# Selective MCL-1 inhibitor ABBV-467 is efficacious in tumor models but is associated with cardiac troponin increases in patients

**DOI:** 10.1038/s43856-023-00380-z

**Published:** 2023-10-25

**Authors:** Junichiro Yuda, Christine Will, Darren C. Phillips, Linu Abraham, Cory Alvey, Abraham Avigdor, Wayne Buck, Lauren Besenhofer, Erwin Boghaert, Dong Cheng, Dan Cojocari, Kelly Doyle, T. Matthew Hansen, Kevin Huang, Eric F. Johnson, Andrew S. Judd, Russell A. Judge, John C. Kalvass, Aaron Kunzer, Lloyd T. Lam, Rachel Li, Ruth L. Martin, Anthony Mastracchio, Mike Mitten, Adam Petrich, Jin Wang, James E. Ward, Haichao Zhang, Xilu Wang, Johannes E. Wolff, Katherine M. Bell-McGuinn, Andrew J. Souers

**Affiliations:** 1 National Cancer Center Hospital East, Kashiwa, Japan; 2 AbbVie Inc, North Chicago, IL USA; 3 Institute of Hematology, Sheba Medical Center, Ramat Gan, Israel; 4 Sackler Faculty of Medicine, Tel Aviv University, Tel Aviv, Israel; 5 Northwestern University, Chicago, IL USA; 6 Present Address: Pleasant Prairie, WI USA; 7 Present Address: Beach Park, IL USA; 8 Present Address: Daiichi Sankyo, Basking Ridge, NJ USA; 9 Present Address: Seagen Inc., Bothell, WA USA; 10 Present Address: Replimmune, Puyallop, WA USA; 11 Present Address: Zionsville, IN USA

**Keywords:** Targeted therapies, Myeloma

## Abstract

**Background:**

MCL-1 is a prosurvival B-cell lymphoma 2 family protein that plays a critical role in tumor maintenance and survival and can act as a resistance factor to multiple anticancer therapies. Herein, we describe the generation and characterization of the highly potent and selective MCL-1 inhibitor ABBV-467 and present findings from a first-in-human trial that included patients with relapsed/refractory multiple myeloma (NCT04178902).

**Methods:**

Binding of ABBV-467 to human MCL-1 was assessed in multiple cell lines. The ability of ABBV-467 to induce tumor growth inhibition was investigated in xenograft models of human multiple myeloma and acute myelogenous leukemia. The first-in-human study was a multicenter, open-label, dose-escalation study assessing safety, pharmacokinetics, and efficacy of ABBV-467 monotherapy.

**Results:**

Here we show that administration of ABBV-467 to MCL-1-dependent tumor cell lines triggers rapid and mechanism-based apoptosis. In vivo, intermittent dosing of ABBV-467 as monotherapy or in combination with venetoclax inhibits the growth of xenografts from human hematologic cancers. Results from a clinical trial evaluating ABBV-467 in patients with multiple myeloma based on these preclinical data indicate that treatment with ABBV-467 can result in disease control (seen in 1 patient), but may also cause increases in cardiac troponin levels in the plasma in some patients (seen in 4 of 8 patients), without other corresponding cardiac findings.

**Conclusions:**

The selectivity of ABBV-467 suggests that treatment-induced troponin release is a consequence of MCL-1 inhibition and therefore may represent a class effect of MCL-1 inhibitors in human patients.

## Plain language summary

Apoptosis is a type of cell death that removes abnormal cells from the body. Cancer cells can have increased levels of MCL-1, a protein that helps cells survive and prevents apoptosis. ABBV-467 is a new drug that blocks the action of MCL-1 (an MCL-1 inhibitor) and could promote apoptosis. In animal models, ABBV-467 led to cancer cell death and delayed tumor growth. ABBV-467 was also studied in a clinical trial in 8 patients with multiple myeloma, a blood cancer. In 1 patient, ABBV-467 treatment prevented the cancer from getting any worse for 8 months. However, in 4 out of 8 patients ABBV-467 increased the levels of troponin, a protein associated with damage to the heart. This concerning side effect may impact the future development of MCL-1 inhibitors as anticancer drugs.

## Abstract (web summary)

Yuda et al. report the development and evaluation of a novel selective MCL-1 inhibitor, ABBV-467. The compound is efficacious in preclinical models but increases in cardiac troponin are seen in a first-in-human study, suggesting potential cardiac toxicity of the drug.

## Introduction

Apoptosis, a form of programmed cell death, is a highly conserved mechanism that plays a critical role in the regulation of tissue homeostasis. The process of intrinsic apoptosis is governed by the B-cell lymphoma 2 (BCL-2) family of proteins, where dynamic binding interactions between pro-death (e.g., BIM, BAX, BAK, NOXA) and prosurvival factors (e.g., BCL-2, BCL-X_L_, MCL-1) ultimately dictate if a cell will undergo programmed cell death^1,2^. Disruption of apoptosis is a hallmark of oncogenesis, tumor maintenance, and chemo-resistance^3^. This can occur by overexpression of prosurvival BCL-2 family proteins, thus making these proteins attractive targets for cancer therapy. Venetoclax is the first BCL-2 family inhibitor to gain market approval for cancer treatment^4^. This molecule works by inhibiting BCL-2, releasing pro-death proteins, and tipping the balance toward apoptosis in cancer cells^5^. The clinical efficacy of venetoclax in hematologic tumors such as chronic lymphocytic leukemia^6,7^ and acute myelogenous leukemia (AML)^8–11^ provides rationale for targeting other prosurvival BCL-2 family proteins.

MCL-1 is a prosurvival protein that mediates malignant cell survival across multiple tumor histologies^12^. *MCL1* gene amplification is frequently found in human cancers, including high incidences in lung and breast cancers^13^. Several key oncogenic pathways lead to increased MCL-1 protein expression through transcriptional or post-transcriptional mechanisms^14^. Studies employing genetic silencing or inducible expression of ligands that inhibit MCL-1 demonstrate its essentiality in the growth of multiple tumor types including multiple myeloma (MM)^15^, AML^16^, and non-Hodgkin lymphoma^17^. Literature data support a role for MCL-1 as a resistance factor to anticancer therapies such as gemcitabine^18^, vincristine, and paclitaxel^19^. In addition, this protein has been identified as a key contributor to both intrinsic and acquired resistance for inhibitors targeting BCL-X_L_^20^ or BCL-2^21^, including venetoclax^22^. Preclinical studies also demonstrated that MCL-1 inhibitors administered as monotherapy or in combination with BCL-2 inhibitors are highly effective in models of MM, AML, and additional hematologic malignancies^22–26^.

The large, hydrophobic, shallow binding groove of MCL-1 has presented substantial challenges for identifying effective small-molecule inhibitors of this protein. Despite several years of investigation toward this goal, MCL-1 inhibitors have only recently entered clinical trials^27,28^. Herein we describe the generation and characterization of the highly potent and selective macrocyclic molecule ABBV-467. In addition, we disclose initial findings from a first-in-human (FIH) trial in patients with relapsed/refractory (R/R) MM (NCT04178902).

In summary, the molecule ABBV-467 is a highly potent and selective MCL-1 inhibitor that demonstrates robust inhibition of tumor growth in in vivo models of hematological malignancies. The macrocyclic compound exhibits a short half-life in humans, a design feature that we implemented as a means of attempting to control the therapeutic index upon administration. Treatment of patients with R/R MM caused troponin increases in 4 of 8 patients. While further data and investigation of this phenomenon are warranted, the results reported herein indicate that troponin release may constitute a predictable side effect of MCL-1 inhibition and could therefore have adverse implications on the future clinical development of this therapeutic class.

## Methods

### Chemistry

Chemical synthesis schemes, experimental procedures, and analytic data for compound 2 and ABBV-467 can be found in the Supplementary Methods.

### X-ray crystallography

#### Protein

The MCL-1 compound structures utilized a maltose-binding protein Mcl-1 fusion construct as follows: 6His-(TEV)-G-[MBP (27-392)]-GS-[Mcl-1 (173-321) K194A, K197A, R201A]. The protein was expressed in E. coli BL21 (DE3) cells. Following cell lysis, the protein was purified using a His tag affinity immobilized metal affinity chromatography column (20 mM 4-[2-hydroxyethyl]-1-piperazineethanesulfonic acid [HEPES], 500 mM NaCl, 0.5 mM tris[2-carboxyethyl]phosphine, pH 7.5) with an elution gradient from 0–300 mM imidazole. The His tag was then removed, the protein dialyzed against buffer (20 mM HEPES, 200 mM NaCl, 0.5 mM tris[2-carboxyethyl]phosphine, pH 7.5) and then run on a Superdex 200 size exclusion chromatography column. Appropriate fractions were collected and then the protein was concentrated to 16 mg/mL. Just prior to crystallization, maltose was added to give 2 mM and glycerol was also added to give 5% (v/v).

#### Crystallization and data collection

Crystals were grown and diffraction data were collected for each compound as follows.

Compound 1: The compound powder was dissolved in dimethyl sulfoxide (DMSO) and compound was then added to the protein to give 0.5 mM compound and 4% (v/v) DMSO. The complex was incubated overnight at 37 °C. Crystals grew using vapor diffusion with the reservoir being 28% (w/v) polyethylene glycol (PEG) 3350, 0.05 M magnesium formate at 17 °C. Crystals were cryoprotected using reservoir solution with 10% (v/v) ethylene glycol and then cryocooled and stored in liquid nitrogen. Diffraction data were collected under cryo conditions at ALBA (Barcelona, Spain).

Compound 2: The compound powder was dissolved in DMSO and compound was then added to the protein to give 5-mM concentration and 4% (v/v) DMSO. The complex was incubated overnight at 37 °C. Crystals grew using vapor diffusion with the reservoir being 24–26% (w/v) PEG 3350, 0.05 M magnesium formate at 17 °C. Crystals were cryoprotected using reservoir solution with 12% (v/v) ethylene glycol and then cryocooled and stored in liquid nitrogen. Diffraction data were collected under gaseous nitrogen (100 K) at the APS beamline 17-ID (Advanced Photon Source, Argonne, IL).

ABBV-467: The compound powder was dissolved in DMSO and compound was then added to the protein to give 4% (v/v) DMSO. The complex was incubated overnight at 37 °C. Crystals grew using vapor diffusion with the reservoir being 25% (w/v) PEG 1500, 0.1 M MIB, pH 6.0 at 17 °C. Crystals were cryoprotected using the reservoir solution and then cryocooled and stored in liquid nitrogen. Diffraction data were collected under gaseous nitrogen (100 K) at the APS beamline 17-ID (Advanced Photon Source, Argonne, IL).

#### Structure solution and refinement

Diffraction intensities were processed using autoPROC^29^ and the structure was solved by molecular replacement using MOLREP^30^ from the CCP4^31^ program suite. Models were rebuilt using COOT^32^ and refined against structure factors using the programs REFMAC5^33^ and autoBUSTER^34^. Figures were prepared using the program PyMOL (Schrodinger, Inc., New York, NY). Data collection and refinement statistics for compounds 1, 2, and ABBV-467 complexed with MCL-1 can be found in Supplementary Table S1. The crystal structures have been deposited to the RCSB Protein Data Bank.

### Time-resolved FRET

Binding affinities for MCL-1, BCL-2, BCL-X_L_, BCL-W, and BCL2-A1 were determined using a time-resolved fluorescence resonance energy transfer (FRET) assay. Test compounds were serially diluted in DMSO starting at 500 mM (20× starting concentration; 100% DMSO followed by a 1:10 dilution of the compound DMSO plate into assay buffer); 10 µL of the compound in 10% DMSO was then transferred into a 384-well plate (low-volume Corning #3673 assay plate). Glutathione S-transferase (GST)-tagged recombinant BCL-2, BCL-X_L_, MCL-1, BCL-W, or BCL2-A1 protein (1 nM each) were mixed with 100 nM Oregon Green™-labeled f-BAK peptide probe and 1 nM Tb-labeled anti-GST antibody were mixed. Then 10 µL of a 2× protein/probe/antibody mix was added to each well of increasing concentrations of test compound in assay buffer (20 mM potassium phosphate, pH 7.6, 50 mM NaCl, 1 mM ethylenediaminetetraacetic acid disodium salt dihydrate, 0.05% pluronic acid F-68, 1 mM DL-dithiothreitol) at room temperature (final concentrations listed in Supplementary Table S2). The samples were then mixed on a shaker for 1 min and incubated for an additional 1 h at room temperature. For each assay plate, a probe/antibody and protein/antibody/probe mixture were included as a negative and a positive control, respectively. Fluorescence was measured on the EnVision™ (Perkin Elmer) using a 340/35-nm excitation filter and 520/525- (F-Bak) and 495/510-nm (Tb-labeled anti-glutathione S-transferase [GST] antibody) emission filters. Dissociation constants were determined using Wang’s equation^35^. The time-resolved FRET assay can be performed in the presence of varying concentrations of human serum to determine apparent half-maximal inhibitory concentration after serum protein binding.

### Determination of caspase-3/7 activation, inner mitochondrial membrane potential (Δψm), and annexin-V positivity by flow cytometry

H929 cells cultured in RPMI 1640 medium (Sigma, St. Louis, MO) supplemented with 10% fetal bovine serum (Gibco, Grand Island, NY) were pretreated with a pan-caspase inhibitor z-VAD-fmk (MP Biomedicals, Solon, OH; 50 µM) for 1 h before the addition of ABBV-467 for a further 6 h. Cells were then washed twice with Dulbecco’s phosphate-buffered saline and resuspended in staining buffer for determination of caspase-3/7 activation, Δψm, and annexin-V positivity using the Intellicyt MultiCyt 4-Plex Kit and high-content flow cytometry (Intellicyt, Albuquerque, NM).

### In vitro cell viability assays

All cell lines were grown and treated in RPMI 1640 or Dulbecco’s modified Eagle medium (Sigma, St. Louis, MO) supplemented with 10% fetal bovine serum (Gibco) or 10% human serum (Sigma) and maintained in a humidified incubator at 5% CO_2_ and 37 °C. DLD-1, MV4-11, NCI-H929 (all ATCC, Manassas, VA, USA) and AMO-1 (DSMZ, Braunschweig, Germany) cells were plated 4000 cells per well in 384-well tissue culture plates and treated with compounds of interest for 24 h at 37 °C, 5% CO_2_ humidified incubator. Each concentration was tested in duplicate at least 3 separate times. Adherent cells were allowed to adhere overnight prior to treatment. Alternatively, AMO-1 or NCI-H929 cells were treated with ABBV-467 for the indicated contact times (1–24 h) at 37 °C in a 5% CO_2_ humidified incubator and washed 3 times with compound-free media prior to incubation for the remainder of a 24-h treatment period. Cell viability was subsequently determined using CellTiter-Glo (Promega, Madison, WI, USA) according to the manufacturer’s instructions. Half maximal effective concentration (EC_50_) and 90% of the maximal effective concentration (EC_90_) values were calculated from the resulting concentration-response curves using non-linear regression analysis.

### Western blot analysis

Parental and BAK-deficient SKBR3 cells were lysed in ice-cold CelLytic™ (Sigma, St. Louis, MO) supplemented with protease (Roche Diagnostics Corporation, Indianapolis, IN) and phosphatase inhibitors (Sigma, St. Louis, MO). Protein concentrations were determined by the BSA assay (Invitrogen, Carlsbad, CA) and 50 µg of protein electrophoresed by SDS Page (Invitrogen). Separated proteins were transferred to nitrocellulose membranes utilizing iBlot^®^ (Invitrogen). Blots were probed with anti-BAK (Abcam, Catalog #ab32371), anti-BAX (Cell Signaling Technology, Catalog #CS2774; Danvers, MA) or GAPDH (Abcam, Catalog #ab110305), followed by IRDye 680RD/800CW-conjugated antibodies (LI-COR Biosciences, Lincoln, NE). Proteins were visualized utilizing the Odyssey^®^ infrared imaging system (LI-COR Biosciences).

### In vivo

All animal studies were conducted in accordance with the guidelines approved by the Institutional Animal Care and Use Committees of AbbVie. The research protocol was approved by In Vivo Pharmacology at AbbVie prior to study initiation but was not publicly registered or disclosed. Female C.B-17 SCID-beige mice (AMO-1, OPM-2, NCI-H929, and OCI-AML2 xenografts) and female NSG mice (MV4-11-Red.Fluc systemic) were purchased from Charles River Laboratories (Wilmington, MA). Animals were housed in a light cycle-controlled room with HEPA filtration at 22 °C. Animals had nesting and climbing cage topper as the primary environmental enrichment. Animals were inoculated subcutaneously in the right flank with 5 × 10^6^ (AMO-1, OPM-2, NCI-H929, and OCI-AML2) or intravenous (IV) with 5 × 10^6^ (MV4-11-Red.Fluc). The inoculation volume (0.2 mL) comprised a 50:50 mixture of cells in growth media and Matrigel (BD Biosciences) for subcutaneous injections and 100% phosphate-buffered saline for IV administration. All cell lines used in this publication were authenticated using IDEXX for STR profiling as well as their IMPACT and human IMPACT panels for pathogen screening. Electronic calipers were used to measure the length and width of each tumor 2 to 3 times per week. Tumor volume was estimated by applying the following equation: volume = length × width^2^/2. All groups remained blinded during measurements and dosing; only veterinary staff were aware of group identity. When tumors reached approximately 200 mm^3^, mice were randomized by match and distributed into treatment and control groups. All xenograft trials were conducted using 5 to 10 mice per group, and all mice were ear tagged and monitored individually throughout the studies. Any animal with a tumor above 2000 mm^3^ or 15% loss in body weight was euthanized and removed from the study. Tumor burden in the systemic MV4-11-Red.Fluc experiment was measured using an IVIS^®^ spectrum from PerkinElmer by taking the total photon flux of the entire dorsal side of an individual mouse. Ten minutes prior to imaging, animals received intraperitoneally 0.2 mL of a 15-mg/mL solution of D-Luciferin (PerkinElmer, Catalog #122799) formulated in 100% phosphate-buffered saline. Animals were anesthetized using 3.5% isoflurane and maintained using 2%. Six days after inoculation, animals were imaged and randomized into vehicle and treatment groups at a total flux of ~1 × 10^8^. A background control mouse that was tumor naive but received D-Luciferin was placed in the upper left corner of each image. The total flux from this animal was subtracted from the total flux of each animal present in the image. ABBV-467 and A-694 were formulated in a mixture of 5% DMSO (dissolved completely before adding other excipients), 10% cremophor EL, and 85% D5W and administered IV; 5-azacitidine was formulated using 100% (vol) sterile water and administered IV; and venetoclax was formulated using 10% (vol) EtOH, 30% (vol) PEG-400, and 60% (vol) Phosal 50 PG and administered orally.

For combination studies, all compounds were administered within 30 min of each other. All study data was captured in Study Director.

### Phase 1 clinical trial

This FIH study (NCT04178902) for ABBV-467 was a multicenter, open-label, dose-escalation study enrolling patients aged ≥18 years who had R/R MM, with the option to enroll R/R AML once recommended phase 2 dose (RP2D) was established. The study design was originally planned to consist of 2 parts: dose escalation and dose expansion, including a minimum of 6 patients treated during dose escalation at what was ultimately to be declared the RP2D. Patients must have received 3 or more prior lines of therapy including at least 1 or more immunomodulatory agents, 1 or more proteasome inhibitors, and 1 or more anti-CD38 monoclonal antibodies. Other eligibility criteria included: Eastern Cooperative Oncology Group performance status ≤2, measurable disease as defined by International Myeloma Working Group (IMWG) consensus criteria^36^, an echocardiogram with an ejection fraction ≥50%, and adequate hematologic, hepatic, and renal function. The prespecified primary objectives were to characterize the safety and toxicity profiles, to establish the RP2D, and to determine the pharmacokinetic (PK) profile of ABBV-467 monotherapy. Safety evaluations included, but were not limited to, adverse event (AE) monitoring, physical examinations, vital sign measurements, electrocardiogram (ECG), cardiac enzyme monitoring, and clinical laboratory testing (hematology and chemistry) as a measure of safety and tolerability for the entire study duration. Parameters used to determine PK included maximum observed plasma concentration (C_max_), terminal elimination half-life (t_1/2_), area under the plasma concentration-time curve (AUC) from time 0 to time of last measurable concentration (AUC_t_), AUC from time 0 to infinity (AUC_0-∞_), and clearance using non-compartmental methods. Secondary objectives were preliminary evaluation of the efficacy of ABBV-467 in R/R MM and R/R AML. Preliminary efficacy endpoints were overall response rate (ORR), clinical benefit rate (CBR), and duration of response (DOR) as evaluated per adapted IMWG^36^ criteria for MM and per adapted International Working Group (IWG)^37^ and European Leukemia Net (ELN)^38^ criteria for AML.

The study was conducted in accordance with the protocol, Operations Manual, International Conference on Harmonisation of Technical Requirements for Registration of Pharmaceuticals for Human Use guidelines, applicable regulations, and guidelines governing clinical study conduct and the ethical principles that have their origin in the Declaration of Helsinki. The study protocol was approved by Sheba Medical Center (IRB number REC-0000040454), Israel and National Cancer Center Institutional Review Board (IRB number REC-0000045267), Japan. Written informed consent was obtained from all patients; the informed consent materials included a note of potential cardiotoxicity as evidenced by class effects of other MCL-1 inhibitors. The first patient on study was recruited on May 19, 2020, and the study completion date was June 22, 2021.

#### Treatment

ABBV-467 was administered as an IV infusion once weekly, for each 28-day cycle over at least a 30-minute period beginning on day 1 of cycle 1. The first infusion was given at half the target dose level. Dosing increased from patient cohort to patient cohort; the escalation was guided by a Bayesian optimal interval design^39^ on the basis of the cumulative number of patients who experienced a dose-limiting toxicity (DLT) at a given ABBV-467 dose level. The DLT observation period was defined as the first treatment cycle (cycle 1). AEs of any grade resulting in discontinuation of study drug or missing any dose as a result of toxicity unexplained by underlying disease will be considered a DLT. Any AE of grade 3 or higher will be considered a DLT, unless toxicity can solely be attributed to the underlying disease, with the following clarifications. A non-hematologic DLT was defined as: grade 3 mucositis, nausea, vomiting, or diarrhea that required total parenteral nutrition, tube feeding, or hospitalization lasting >72 h; any grade 4 non-hematologic laboratory abnormality; and any instance of an AE that met the definition of Hy’s Law. A hematologic DLT was defined as: grade 4 neutropenia lasting >5 days; grade 4 anemia unexplained by underlying disease; febrile neutropenia defined as absolute neutrophil count <1000/mm^3^ with a fever ≥38.3 °C or ≥38 °C for 1 h; grade 4 thrombocytopenia; or grade 3 thrombocytopenia associated with clinically significant bleeding.

#### Study assessments

Blood samples for plasma ABBV-467 concentration analysis were collected on days 1, 2, 3, and 15–17 of cycle 1 as well as on days 1 of cycles for PK analysis. Plasma samples of ABBV-467 were quantified using a validated LC-tandem MS with a lower limit of detection of 3 ng/mL. ABBV-467 PK parameters were calculated using standard non-compartmental approaches with Phoenix, WinNonlin (Version 8.0, Certara). AE severity was graded according to the National Cancer Institute Common Terminology Criteria for Adverse Events v5.0 for up to 30 days after the last dose. Analyses of AEs will include only treatment-emergent AEs. Triplicate ECGs were performed on days 1, 2, 15, and 16 of cycle 1. Creatine phosphokinase and troponin were determined by a central laboratory. An echocardiogram was performed at screening and day 1 of cycle 2 and cycle 6.

See Supplementary Methods for additional details on eligibility criteria and Supplementary Information for the clinical protocol.

### Statistics and reproducibility

For in vivo studies, maximal tumor growth inhibition (TGI) was calculated as the greatest treatment response using the following equation: Maximal TGI = (1 − mean tumor volume of the treated group/mean tumor volume of the vehicle control group) × 100. The tumor growth delay (TGD) percentage was determined as the percentage increase of the median time period for the treatment group to reach an arbitrary tumor volume of 1000 mm^3^ relative to the vehicle control group. A complete tumor regression response was the portion of the population with tumors ≤25 mm^3^ for at least 3 consecutive measurements. A power analysis was conducted using historical data to determine the number of animals required to yield significance of 0.05 at a 30% difference. There were no animals excluded. Data from experiments in vivo were analyzed using the student’s t-test for TGI values and the Mann–Whitney U test for TGD using Excel and GraphPad Prism. Fisher’s exact test was used to probe significance of differences in response frequency.

For in vitro viability assays, mean EC_50_ and EC_90_ values were determined from the resulting dose-response curves from at least 3 independent experiments, and depict the mean ± standard error of the mean.

### Reporting summary

Further information on research design is available in the Nature Portfolio Reporting Summary linked to this article.

## Results

### ABBV-467 is a selective and highly potent MCL-1 inhibitor

Our investigations with earlier MCL-1 inhibitors demonstrated that apoptosis is rapidly induced in MCL-1-dependent cell lines following short-exposure compound incubation^20^, an observation that has been confirmed by other laboratories^24,25^. In addition, given the reported deleterious impact of *mcl1* genetic knockout on various tissue types^40^, we anticipated that compounds neutralizing MCL-1 may have a limited therapeutic index relative to inhibitors of BCL-2^41,42^. Together these data indicated that intermittent IV administration of an MCL-1 inhibitor could induce antitumor activity while allowing for elevated control over patient safety. Thus, we hypothesized that an IV infusion of a potent molecule with a short serum half-life could provide the optimal profile for exploring direct MCL-1 inhibition in human patients.

**Fig. 1:**
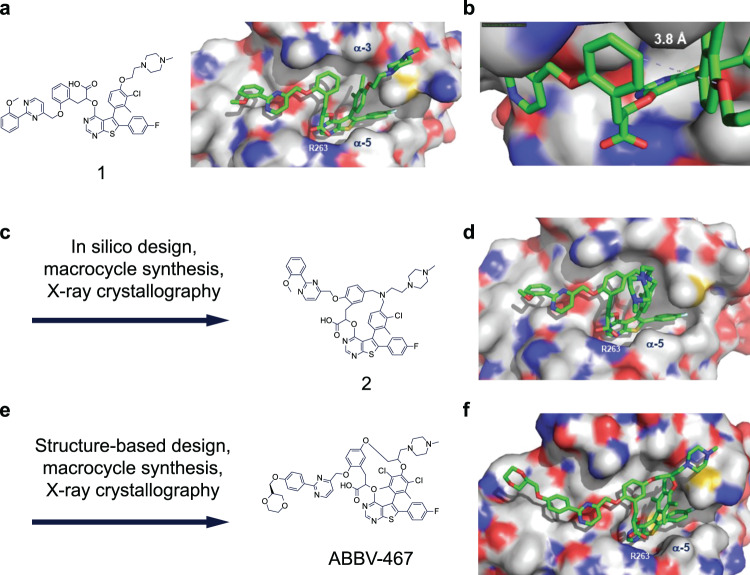
Summary of the structure-based design of macrocyclic MCL-1 inhibitor ABBV-467. **a** Structure of linear compound 1 (MIK665) and X-ray crystal structure of compound 1 in complex with MCL-1 (PDB 8EKX). **b** Magnified view of central binding pose of compound 1 complexed with MCL-1 reveals proximity within 3.9 Å and possible macrocyclization. **c** Design of macrocyclic prototype molecule 2 as inspired by folded binding pose of compound 1 and subsequent in silico-based design with appropriate energy-minimized macrocyclic constraints. **d** X-ray crystal structure of compound 2 in complex with MCL-1 (PDB 8EL0). **e** Multiparameter investigation led to generation of ABBV-467. **f** X-ray crystal structure of ABBV-467 in complex with MCL-1 (PDB 8EL1).

Our efforts began with the generation of a crystal structure of compound 1 (MIK665)^43^ complexed with MCL-1. This compound demonstrated high target affinity as measured in a FRET assay (Table 1). As shown in Fig. 1a, inhibitor 1 makes extensive hydrophobic interactions along the BH3 binding groove of MCL-1 while also forming a hydrogen bond between its pendant carboxylic acid group and the side-chain of R263 in helix 5 of the protein. Although compound 1 is linear in structure, the compact and folded binding pose suggested that a macrocycle constraint could be incorporated to orient the molecule into a highly active conformation (Fig. 1b). Modeling programs BROOD (OpenEye Scientific Software Inc., Santa Fe, NM) and Spark (Cresset Software, Litlington, Cambridgeshire, UK) were then utilized to explore different cyclization strategies, resulting in the design and subsequent preparation of compound 2 (Fig. 1c). The crystal structure of compound 2 complexed with MCL-1 (Fig. 1d) revealed a similar binding pose to that of compound 1, although lesser target affinity was observed in the FRET assay (Table 1). Extensive investigation culminated in the replacement of the nitrogen tether with a carbocyclic linker, incorporation of a symmetric hexa-substituted phenyl core, and extension of the pendant methoxy of compound 2 to provide the highly potent and selective MCL-1 inhibitor ABBV-467 (Fig. 1e, f). This molecule possessed the physicochemical and pharmacokinetic properties consistent with the desired profile of a short-lived IV-administered compound.

**Table 1:** Binding affinity of compounds 1, 2, and ABBV-467 to BCL-2 family proteins, and cellular activity of ABBV-467 and other clinical-stage MCL-1 inhibitors in human tumor cell lines.

	TR-FRET, Ki, nM				
	MCL-1	BCL-2	BCL-X_L_	BCL-W	BCL2-A1
*Binding affinity*					
1 (MIK665)	<0.01	599	>660	>468	>468
2	5.54	>1200	>660	NT	NT
ABBV-467	<0.01	>642	>376	>247	>402

	AMO-1 EC_50_ (nM, 10% FBS)	H929 EC_50_ (nM, 10% FBS)	MV4-11 EC_50_ (nM, 10% FBS)	DLD-1 EC_50_ (nM, 10% FBS)
*Cellular activity*				
ABBV-467	0.16	0.47	3.91	>10,000
MIK665	2.06	4.75	10.87	4750
AMG 176	90.6	195	106	>10,000
AZD5991	22.9	31.7	34.9	>10,000
AMG 397	13.5	13.0	43.6	>10,000

Data are representative of the mean of at least 3 independent experiments. The impact on cell viability was determined by CellTiter-Glo^®^ after 24 h of continuous treatment (see “Methods”).

*BCL-2* B-cell lymphoma 2, *EC*_*50*_ half maximal effective concentration; *FBS* fetal bovine serum, *Ki* dissociation constant, *NT* not tested, *TR-FRET* time-resolved fluorescence resonance energy transfer.

As shown in Table 1, ABBV-467 is a potent binder of human MCL-1 (dissociation constant [Ki] values < 0.010 nM) and demonstrates lesser affinity (Ki values > 200 nM) toward the other BCL-2 family members BCL-X_L_, BCL-2, BCL-W, and BCL2-A1. This high binding affinity to MCL-1 translated to cellular efficacy (Table 1), where cell death at subnanomolar concentrations was observed in tumor cell lines derived from MM (AMO-1, H929) and AML (MV4-11). The concentration of ABBV-467 required to induce cell death was less than that of other clinically relevant MCL-1 inhibitors^24–28^ (Table 1 and Supplementary Figs. S1, S2), reflecting the highly active orientation of this macrocyclic pharmacophore. Importantly, ABBV-467 was inactive in cell lines such as DLD-1, where BCL-X_L_ plays a cooperative role in tumor cell maintenance^44^ (Table 1). In a panel of mouse leukemic cells engineered to be dependent on exogenously expressed human antiapoptotic BCL-2 family members^45^, ABBV-467 showed selective inhibition of the human MCL-1–expressing B-cell acute lymphoblastic leukemia variant (Supplementary Table S3). Additional profiling across a large number of kinases, ion channels, and receptors confirmed the selectivity of ABBV-467 for the target protein (Supplementary Data 1).

**Fig. 2:**
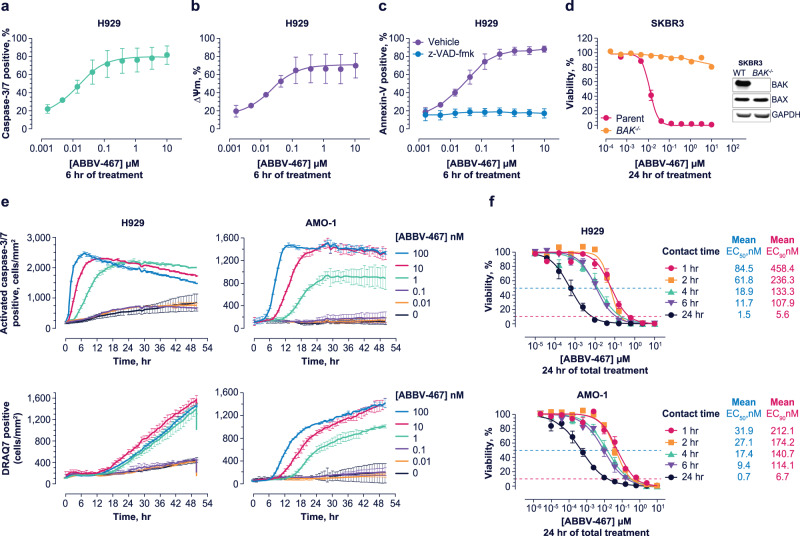
Apoptosis induced by ABBV-467 is mechanism based and induces cell death in MCL-1-dependent cell lines after short target engagement periods. H929 cells were treated with ABBV-467 in the presence or absence of the pan-caspase inhibitor z-VAD-fmk (50 µM) and the impact on caspase-3/7 activation (**a**), mitochondrial membrane potential (ΔΨm; **b**), and apoptosis (**c**) determined. Alternatively, *BAK*-deficient SKBR3 cells were treated with ABBV-467 for 24 h and the impact on cell viability compared with the parental cell line, determined by CellTiter-Glo. Inset depicts a western blot of BAK and BAX expression in SKBR3 cell variants using GAPDH as a loading control. Each of the 3 proteins were probed on separate blots (**d**). Data in (**a**–**d**) are presented as the mean ± standard error of the mean. H929 and AMO-1 cells were treated with ABBV-467 at the indicated concentrations and the impact on caspase-3/7 activation and viability (DRAQ7-positive) cells determined over time by Incucyte^®^. Data are presented as the mean ± standard deviation (**e**). H929 or AMO-1 cells were cultured in media containing 10% human serum and treated with ABBV-467 for the indicated contact times. Cells were subsequently washed 3 times with drug-free media and incubated in drug-free media for the remainder of the 24-h treatment period at 37 °C before cell viability was determined by CellTiter-Glo. Mean EC_50_ and EC_90_ values were determined from the resulting dose-response curves from at least 3 independent experiments, each assessed in quadruplicate, and depict the mean ± standard error of the mean. Presented ABBV-467 dose-response curves in H929 and AMO-1 cells were compiled from at least 3 independent experiments (**f**). EC_50_, half maximal effective concentration; EC_90_, 90% effective concentration; WT, wild-type.

Treatment of H929 cells with ABBV-467 induced the classical hallmarks of apoptosis^46^, including a dose-dependent loss in mitochondrial membrane potential, caspase-3/7 activation, and phosphatidyl serine externalization (Fig. 2a–c). Apoptosis induced by ABBV-467 was abrogated by pan-caspase inhibition with z-VAD-fmk, further highlighting apoptosis as the mode of cell death in this cell line. Moreover, deletion of the BCL-2 effector gene *BAK* in MCL-1-dependent SKBR3 (Fig. 2d, Supplementary Fig. S3) or SU-DH-1 (Supplementary Fig. S4) cells completely inhibited the activity of ABBV-467. Deletion of the genes encoding *BAX* or the BH3-only proteins BIM or NOXA in SKBR3 cells did not impact activity of either compound (Fig. 2d, Supplementary Fig. S5).

To further understand the kinetics of target engagement on cell death, H929 and AMO-1 cells were treated in vitro with ABBV-467 for 1–24 h and the compounds subsequently removed by washing with drug-free media prior to assessing cell death after a total incubation time of 24 h (see Fig. 2e). ABBV-467 treatment for as little as 1 h prior to compound washout was sufficient to induce pervasive cell death (EC_90_) at nanomolar concentrations when assessed 23 h later (see Fig. 2f).

### ABBV-467 inhibits tumor growth of MM xenografts as a monotherapy

**Fig. 3:**
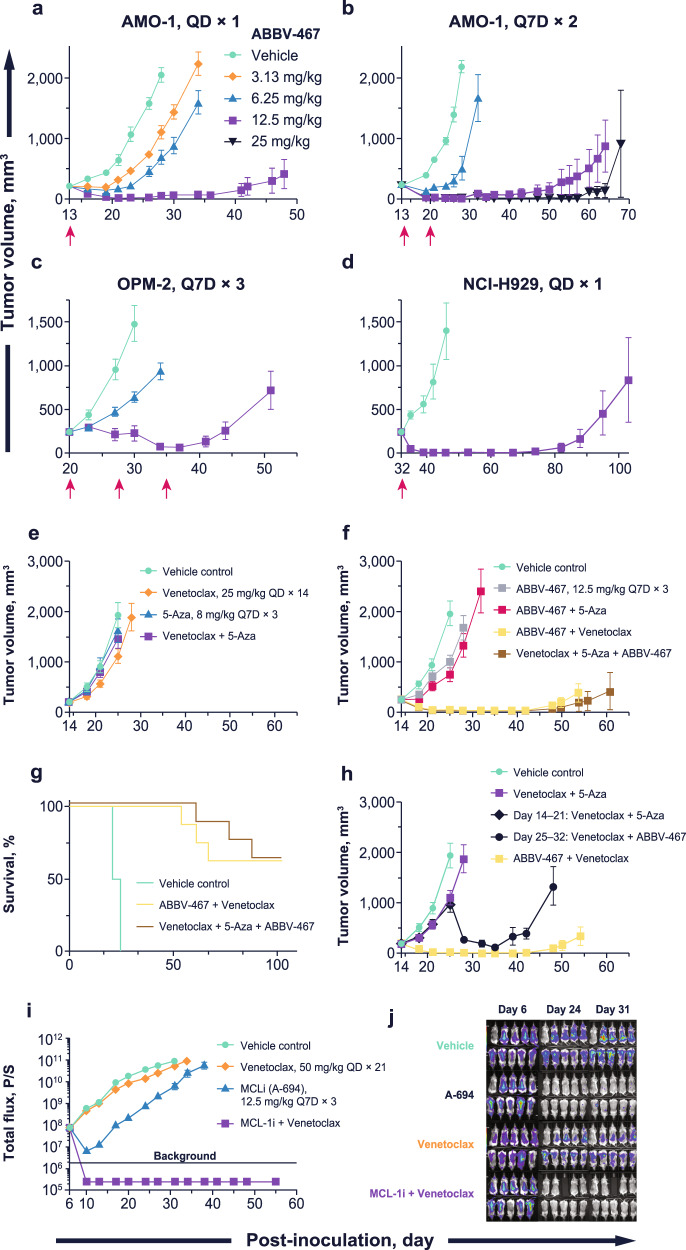
ABBV-467 induces tumor regression in xenograft models of MM and AML when combined with venetoclax. Studies were conducted in SCID/bg mice when tumor volume reached ~200 mm^3^. Each data point represents the group average, with error bars indicating the standard error of the mean. Animals were treated with **a** ABBV-467 on a QD × 1 dosing schedule in AMO-1 xenografts (*n* = 8, 50 total animals), or **b** Q7D × 2 (*n* = 7, 40 total animals). **c** OPM-2 xenografts with ABBV-467 on a Q7D × 3 schedule (*n* = 8, 30 total animals). **d** Single dose in NCI-H929 xenografts (*n* = 5, 20 total animals). **e** OCI-AML2 xenografts treated with venetoclax (QD × 14), 5-azacitidine (Q7D × 3), or in combination (*n* = 8, 100 total animals). **f** In vivo efficacy of ABBV-467 in combination with either venetoclax, 5-azacitidine, or the triple combination. **g** Comparison of survival in mice treated with ABBV-467 + venetoclax or ABBV-467 + venetoclax + 5-azacitidine. **h** In vivo rescue experiment of venetoclax + 5-azacitidine-treated animals. Two groups of animals were treated with venetoclax + 5-azacitidine until the groups reached an average tumor volume of 1000 mm^3^ on day 25. One group continued receiving venetoclax + 5-azacitidine, while the other group switched to venetoclax + ABBV-467. **i** Systemic model of MV4-11 (*FLT3-*ITD*, MLL/AF-4*) transduced with red-shifted *Luciola italica* luciferase under control of the stable UbC promoter (*n* = 9, 60 total animals). The tumor load is given by the amount of light signal detected, expressed as a photon flux from the whole mouse per second. Tumor load is expressed as a function of time in the absence (vehicle) or presence of treatment with A-694 (Q7D × 3, IV), venetoclax (QD × 21, PO) or in combination. Following treatment with the combination, the tumor load dropped below the detectable limits of the instrument, flux of 2e6, noted by the black horizontal line. **j** Representative bioluminescent images of the animals from study (**i**) over time. Animals were size matched on day 6. For days 24 and 31, the mouse in the upper left corner of each image is a negative control (naive mouse injected with D-luciferin). Arrows indicate treatment days. 5-Aza, 5-azacitidine; D, day; *FLT3*-ITD, FMS-like tyrosine kinase-3 internal tandem duplication; IV, intravenous; MCL-1i, MCL-1 inhibitor; PO, oral; Q, every.

In AMO-1 tumor-bearing mice, a single IV administration of ABBV-467 induced significant (*p* < 0.0001, student’s t-test, ϴ = 1.67) and dose-dependent TGI ranging from 46–97% following doses of 3.13, 6.25, or 12.5 mg/kg (Fig. 3a). Complete tumor regression was observed at 20 days post-treatment in mice treated with 12.5 mg/kg ABBV-467, yielding a TGD of 222% (*p* < 0.00012, Mann–Whitney U, ϴ = 1.89). Dosing once weekly for 2 weeks (Q7D × 2) at 6.25 or 12.5 mg/kg increased the efficacy, with the 12.5-mg/kg dose group exhibiting highly durable tumor regression (TGD = 338%, *p* < 0.002, Mann–Whitney U, ϴ = 1.95; Fig. 3b). There were no tolerability issues (no weight loss or deaths) at doses up to 12.5 mg/kg. Administration of 25 mg/kg on a Q7D × 2 schedule was not well-tolerated; thus, 12.5 mg/kg was selected as the highest dose for further studies.

To evaluate in vivo efficacy in MM models of high-risk disease, t(4;14)-translocated OPM-2 xenografts were treated with either 6.25 or 12.5 mg/kg of ABBV-467 once weekly for 3 weeks. IV administration of ABBV-467 induced stasis of OPM-2 after the initial dose of 12.5 mg/kg; 2 additional doses caused tumor regression, with a TGI of 85% (*p* < 0.003, student’s t-test, ϴ = 1.67) and TGD of 335% (*p* < 0.002, Mann–Whitney, ϴ = 1.99, Fig. 3c). There was only a minor growth delay at the 6.25-mg/kg dose after 3 doses and a maximal TGD of 82% (*p* < 0.002, Mann–Whitney, ϴ = 1.866). Given these data, an additional model bearing the t(4;14) translocation (NCI-H929) was treated with ABBV-467 at 12.5 mg/kg. A single dose of ABBV-467 caused complete responses in all mice (Fig. 3d). Tumors remained below 1000 mm^3^ in 40% of the mice until 250 days post-treatment (Supplementary Fig. S6).

### Treatment of AML flank and systemic xenografts in vivo with ABBV-467 or A-694 combined with venetoclax regresses tumors

Given the observed and reported^24,25^ sensitivity of AML cells to MCL-1 inhibition in vitro, the activity of ABBV-467 was evaluated as both monotherapy and with standard-of-care (SOC) agents in AML xenografts in vivo. The SOC regimens, venetoclax (25 mg/kg, QD × 14), azacitidine (8 mg/kg, Q7D × 3), or the combination of both, had no meaningful effect on OCI-AML2 xenograft growth (Fig. 3e). These tumors were also resistant to ABBV-467 as a monotherapy or in combination with 5-azacitidine (Fig. 3f). Significant tumor inhibition was observed when ABBV-467 was combined with either venetoclax (*p* < 0.00001, student’s t-test, ϴ = 1.8) or venetoclax and 5-azacitidine (*p* < 0.00001, student’s t-test, ϴ = 1.8), with each regimen achieving TGI of 99%. Both combination treatments induced complete tumor regression in all tumors for 20 days post-treatment. At 120 days post-inoculation, 62.5% of animals treated with either the doublet or triplet remained tumor free (Fig. 3g). Moreover, both groups had a TGD > 878% (*p* > 0.4, Man-Whitney, ϴ = 0.29) signifying no significant difference between these therapies.

Tumors that failed to respond to venetoclax + 5-azacitidine remained sensitive to venetoclax + ABBV-467. Minor tumor growth inhibition (42–48%) was observed when tumor-bearing mice were treated with venetoclax + 5-azacitidine for 14 days. When average tumor volume reached 1000 mm^3^ (day 25), one group continued with venetoclax + 5-azacitidine therapy, while the other was switched to venetoclax + ABBV-467 (Fig. 3h). While tumors in the former treatment group continued to progress, the venetoclax + ABBV-467 combination caused a significant reduction in tumor volume (1000 mm^3^ to 125 mm^3^; *p* < 0.0006, student’s t-test, ϴ = 1.57). These data suggest that the concomitant MCL-1 and BCL-2 inhibition can impede growth of AML tumors that progress on the SOC venetoclax + 5-azacitidine regimen.

Because AML localized in the bone niche can become resistant to venetoclax therapy^47^, a systemic model of MV4-11 that engrafts in the spine and femur of mice was used to assess venetoclax + MCL-1 inhibitor therapy. MV4-11 cells contain both a mixed-lineage leukemia (*MLL*) fusion as well as an FMS-like tyrosine kinase-3 (*FLT3*) internal tandem duplication (ITD) mutation. The co-occurrence of these mutations can drive full transformation to AML, while the *FLT3-*ITD mutations specifically have been shown to upregulate MCL-1 both in AML leukemic stem cells and cell lines^48^. Venetoclax dosed at 50 mg/kg for 21 days had no antitumor activity; this observation contrasted with previous studies in mice subcutaneously engrafted with MV4-11^49^, highlighting the resistant phenotype of the systemic model. Administration of A-694, a molecule with similar structure and properties to ABBV-467 (see Supplementary Fig. S7 and Supplementary Tables S4, S5), afforded substantial loss of luciferase activity that quickly recovered during the dosing cycle (Fig. 3i). The combination of A-694 + venetoclax reduced luciferase activity below the limits of detection within 7 days of treatment (log transformed TGI of 87%, *p* < 0.000001, log transformed student’s t-test, ϴ = 1.98, Fig. 3i, j). Luciferase activity did not recover by 50 days post-treatment and no cancer-related health issues were observed in the animals.

### ABBV-467 treatment shows evidence of disease control in a patient with MM, associated with cardiac troponin increases in 4 of 8 patients

On the basis of the overall pharmacologic profile of ABBV-467, Investigational New Drug-enabling toxicology studies were executed.

ABBV-467 was profiled in safety pharmacology studies in anesthetized (non-Good Laboratory Practice [GLP]) and telemetry-instrumented conscious (GLP) dog cardiovascular assays (Supplementary Table S6). In both models, cardiovascular function (blood pressure, heart rate, or ECG parameters) was not affected up to micromolar concentrations (conscious study: 6.58 mg/mL; anesthetized study: 16.83 mg/mL).

No direct impact of ABBV-467 on cardiovascular endpoints (anatomic, clinical pathology, and/or function) was observed in multidose GLP toxicity studies in rats or dogs. Due to the lack of any clear impact on cardiovascular function or electrophysiology in safety pharmacology studies or microscopic changes in the heart following repeat dosing of ABBV-467 in toxicity studies, troponin levels were not measured in the preclinical studies per standard protocol.

Once-weekly dosing of ABBV-467 in rats and dogs resulted in microscopic observations of apoptosis/single-cell necrosis^50^ in multiple tissues, with primary target organs including pancreas, liver, gastrointestinal tract, and the hematopoietic system. In lymphoid organs and testes, decreased cellularity and tubular degeneration, respectively, were also observed. In both species, all toxicities appeared to be on-target, on the basis of the nature of the findings (apoptosis/single-cell necrosis), were generally dose dependent in severity and incidence, monitorable, and were considered partially or fully reversible after a 4-week recovery period (Supplementary Table S7). Together with the observations in the cardiovascular safety studies, these data with ABBV-467 demonstrated a safety and tolerability profile consistent with advancement into human trials.

The FIH study (NCT04178902) for ABBV-467 was a multicenter, open-label, dose-escalation trial enrolling adults who had R/R MM. ABBV-467 was administered weekly, on a 28-day cycle with the first infusion given at half the target dose for the dose level.

In total, 8 patients with R/R MM were enrolled between May 2020 and June 2021 (study end) into 3 dose-escalation cohorts: 0.16 mg/kg, *n* = 3; 0.32 mg/kg, *n* = 2; and 0.53 mg/kg, *n* = 3. The dose escalation was primarily guided by a Bayesian optimal interval design using DLT frequency.

Patient demographics are summarized in Supplementary Table S8. Median age was 64 years, the majority (75%) were male, and 75% had International Staging System stage ≥II disease. The enrolled patients had received a median of 7 (range, 6–15) previous lines of systemic therapy that included at least 1 immunomodulatory agent, proteasome inhibitor, and anti-CD38 monoclonal antibody.

### Pharmacokinetics

**Fig. 4:**
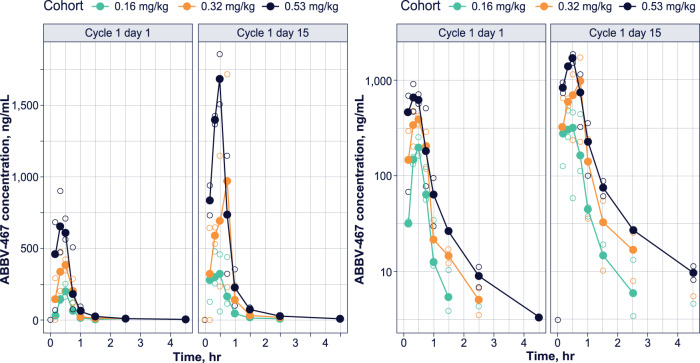
Plasma concentration-time profile of ABBV-467 in patients with MM. Plasma concentration-time profile of half or target dose of ABBV-467 on a linear or log scale. Plasma concentrations were undetectable at time points sampled after 4 h. Open circles are from individual patient concentrations and solid circles represent mean plasma concentrations at the corresponding timepoint. Patients with undetectable or zero concentration values are not displayed on the logarithmic scale. RStudio (Version 1.4) was used to assist in data presentation. MM, multiple myeloma.

Complete concentration-time profiles were determined for the 8 patients across the 3 ABBV-467 dose levels. The mean plasma concentration-time profiles following a 30-minute IV infusion at the various dose levels are presented in Fig. 4 for ABBV-467 in the dose cohorts of 0.16, 0.32, and 0.53 mg/kg on cycle 1 day 1 (half target dose) and cycle 1 day 15 (full target dose), respectively. Pharmacokinetics and exposure of ABBV-467 increased across the dose range of 0.08–0.53 mg/kg (Supplementary Table S9). The peak plasma concentration occurred at the end of infusion and circulating plasma concentrations of ABBV-467 were eliminated 4 h after completion of infusion, with an average half-life of <1 h.

### Safety

The median number of weekly doses for the 8 patients was 4 (range, 2–31). Six patients received more than 3 doses of ABBV-467, while the remaining 2 received 2 or 3 doses, respectively. Any-grade AEs were reported in 7 of 8 (88%) patients. No cardiac disorders of any grade and no grade 5 toxicities were reported in any dose cohort. The most common treatment-emergent AEs (TEAEs) were diarrhea, nausea, increased aspartate aminotransferase, increased troponin I, and increased troponin T (25% each) (Table 2). Five (63%) patients had treatment-related AEs: nausea (*n* = 1), increased levels of alanine aminotransferase (*n* = 1), aspartate aminotransferase (*n* = 2), amylase (*n* = 1), troponin T (*n* = 2), and troponin I (*n* = 2). The occurrence of diarrhea, nausea, and increased aspartate aminotransferase was consistent with predicted toxicology on the basis of preclinical findings.

**Table 2:** Overview of treatment-emergent adverse events (AEs).

	0.16 mg/kg (*n* = 3)	0.32 mg/kg (*n* = 2)	0.53 mg/kg (*n* = 3)	Total (*N* = 8)
Any AE, *n* (%)	2 (67)	2 (100)	3 (100)	7 (88)
Increased troponin I	0	1 (50)	1 (33)	2 (25)
Diarrhea	1 (33)	1 (50)	0	2 (25)
Nausea	2 (67)	0	0	2 (25)
Increased aspartate aminotransferase	0	0	2 (67)	2 (25)
Increased troponin T	1 (33)	0	1 (33)	2 (25)
Anemia	0	1 (50)	0	1 (13)
Febrile neutropenia	1 (33)	0	0	1 (13)
Neutropenia	1 (33)	0	0	1 (13)
Vitreous floaters	1 (33)	0	0	1 (13)
Gastritis	1 (33)	0	0	1 (13)
Malaise	0	1 (50)	0	1 (13)
Increased alanine aminotransferase	0	0	1 (33)	1 (13)
Increased amylase	0	0	1 (33)	1 (13)
Arthralgia	1 (33)	0	0	1 (13)
Arthritis	1 (33)	0	0	1 (13)
Pain in extremity	1 (33)	0	0	1 (13)
Pelvic pain	1 (33)	0	0	1 (13)

The grade 3 TEAEs reported in the 3 patients were febrile neutropenia and neutropenia (1 patient), anemia (1 patient), and troponin I increased (1 patient). One patient experienced a grade 3 serious TEAE of febrile neutropenia and a grade 3 non-serious neutropenia event; both events were assessed as not related to treatment with ABBV-467 by the investigator. A second patient reported a grade 3 non-serious event of anemia that was also assessed as unrelated to ABBV-467 treatment by the investigator and was ongoing at the end of the study. No action was taken with ABBV-467 for either patient. A third patient had a grade 3 non-serious treatment-related TEAE of troponin I elevation that led to discontinuation of ABBV-467 and was classified as a DLT. The remaining 7 patients discontinued treatment with ABBV-467 due to progressive disease (*n* = 6) and decision of physician (*n* = 1).

Troponin elevations occurred in 4 (50%) patients: 1 (12.5%) at dose 0.16 mg/kg, 1 (12.5%) at dose 0.32 mg/kg, and 2 (25%) at dose 0.53 mg/kg (Table 2, Supplementary Data 2). Of these 4 patients, 3 (75%) had a grade 1 troponin elevation and 1 (25%) had a grade 3 troponin elevation. The troponin elevations were detectable at the first blood draw post-infusion (4 h). In addition, the occurrence of grade ≥1 elevated troponin was unrelated to the peak plasma concentration and AUC of ABBV-467 (Supplementary Fig. S8) across the studied dose range. None of these troponin elevations were related to clinical cardiac symptoms, or ECG or echocardiogram changes. The patient with grade 3 troponin I elevations (37.7 ng/L; reference range, 2.3–20 ng/L, 1.9× upper limit of normal) was a 61-year-old man with International Staging System Stage 3 immunoglobulin A kappa MM, a baseline Eastern Cooperative Oncology Group performance status of 2, and no remarkable cardiac history documented. The screening ECG was normal, and the echocardiogram showed an ejection fraction of 50% (the protocol threshold for eligibility) and left ventricular regional wall hypokinesis.

**Fig. 5:**
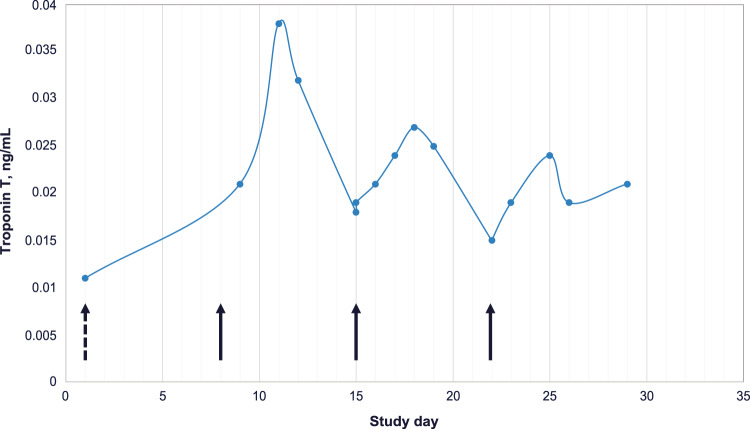
Serum troponin T increase occurred post–ABBV-467 infusion. Serum troponin T release occurred post–ABBV-467 infusion. Representative graph of serum troponin T levels from a single patient on ABBV-467 during cycle 1 of treatment. Troponin T elevations were first identified after the patient received their first full dose of ABB-467. Troponin T remained elevated throughout the first cycle with values ranging from 0.015 to 0.038 ng/mL. Arrows represent the days the patient received ABB-467 (Dash = ½ target dose, Solid = target dose). Target dose = 0.53 mg/kg Q1W. (Reference range: ≤0.014 ng/mL). Q1W, once per week.

The relationship between ABBV-467 infusions and the elevated serum troponin levels can be seen in Fig. 5, a representative troponin time course of a single patient during cycle 1 of treatment. The patient’s screening ECG was recorded as abnormal, with a finding of non-clinically meaningful complete right bundle branch block. The screening echocardiogram was reported as normal with an ejection fraction of 67%. The patient received the first infusion of 0.26 mg/kg ABBV-467 (half the target dose) on cycle 1 day 1. After the first infusion, troponin T values were within normal limits (reference range, ≤0.014 ng/mL). Clinically, the patient remained asymptomatic with no new cardiac findings. On cycle 1 day 8, the patient received the second infusion at the full target dose of ABBV-467 (0.53 mg/kg). Local troponin T was measured on the following day (cycle 1 day 9) and was found to be elevated at 0.021 ng/mL (reference range, <0.014 ng/mL). An AE of cardiac troponin T elevation (grade 1) was recorded. The troponin T values remained elevated from cycle 1 day 9 through cycle 1 day 28 (end of cycle 1), with values ranging from 0.015–0.038 ng/mL and increases recorded after each infusion. The patient remained asymptomatic, as repeat ECG and echocardiogram performed after the troponin elevation remained largely unchanged, with ejection fractions measured between 64–69%.

### Efficacy

On the basis of the observation of elevated troponins in treated patients, AbbVie made the decision to terminate the trial prior to reaching the predicted efficacious dose of ABBV-467. Limited disease responses were available for patients treated on study to determine efficacy of the compound. No objective responses nor clear signs of antitumor activity were observed. However, disease responses for 1 patient suggested disease stabilization, as described below.

**Fig. 6:**
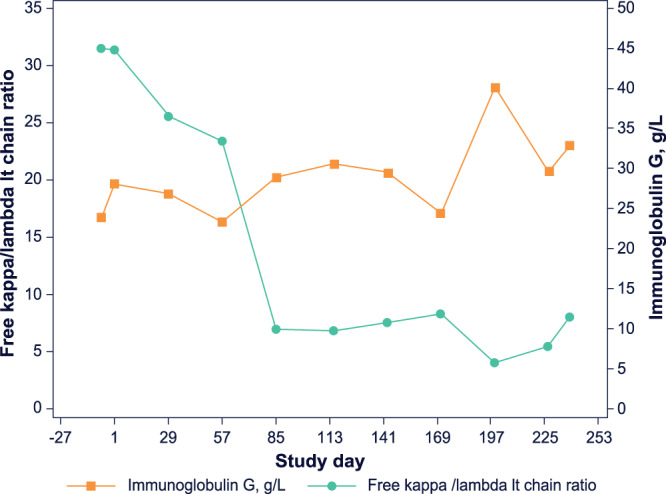
Free kappa/lambda light (lt) chain ratio (turquoise) and immunoglobulin G (orange) levels in patient who achieved disease control while being treated with ABBV-467. Patient had relatively slow clinical course after initiation of ABBV-467 for about 8 months despite treatment history of 10 lines after pretreatment including EPD and D-MPV. Disease control over 8 cycles represented by stable immunoglobulin G level combined with a decrease of the patient’s free lt chain ratio until treatment discontinuation due to PD. D-MPV, dexamethasone, methotrexate, procarbazine, vincristine; EPD, elotuzumab, pomalidomide; PD, progressive disease.

An 83-year-old woman with R/R MM remained on study with stable disease for almost 8 months before she was taken off study drug treatment due to disease progression. She was diagnosed with MM 6 years prior to enrollment, with her disease confirmed before study start as immunoglobulin G kappa subtype. Fluorescence in situ hybridization testing confirmed this patient as having a chromosomal (4;14) translocation and a *1q* amplification, stratifying this patient as high-risk^51^. The patient was enrolled in the 0.16-mg/kg ABBV-467 dose level and received half the target dose of ABBV-467 (0.08 mg/kg) on cycle 1 day 1 and full target dose of 0.16 mg/kg ABBV-467 once weekly beginning from cycle 1 day 8. AEs reported included increased troponin T, diarrhea, gastritis, pain in extremity, arthritis, arthralgia, and nausea, with only the nausea attributed as having a reasonable possibility of being related to study drug (investigator’s opinion). Her stable disease was assessed by IMWG response criteria^36^ including monitoring for changes in immunoglobulin and free light chain (Fig. 6). Interestingly, this patient’s clinical results suggested that ABBV-467 was involved in the disease stabilization of R/R MM for approximately 8 months, even though the patient was refractory to 10 prior treatment regimens, including proteasome inhibitors (ixazomib, bortezomib, and carfilzomib), immunomodulators (lenalidomide and pomalidomide), CD38 monoclonal antibody (daratumumab), SLAMF7 monoclonal antibody (elotuzumab), chemotherapy (melphalan), and corticosteroids.

## Discussion

The prosurvival protein MCL-1 prevents apoptosis by binding to the pro-death BCL-2 family proteins. Overexpression of the MCL-1 protein or amplification of the *MCL1* gene has been observed in multiple tumor types, where it is associated with tumorigenesis and poor prognosis^28^. In addition, MCL-1 has been implicated as a resistance factor to BCL-2 family inhibitors as well as multiple classes of chemotherapeutic agents^27^. These data support the therapeutic potential of inhibitors targeting this prosurvival BCL-2 family member^24^.

The generation of selective BCL-2 family inhibitors has been challenging, at least in part due to the high affinity of the endogenous protein-protein interactions that require disruption and the shallow, hydrophobic binding pockets of prosurvival proteins^52^. MCL-1 has been a particularly recalcitrant target, although excellent progress toward drug-like, potent, and selective inhibitors has recently been made with several structurally distinct compounds now in phase 1 clinical trials^27,28^. The discovery of ABBV-467 adds to this armamentarium.

Exploration of the clinical safety and preliminary efficacy of an MCL-1 inhibitor included several design considerations. As preclinical data revealed a role for MCL-1 in normal tissue maintenance^40^, we envisioned that IV administration would facilitate the careful delivery of an MCL-1 inhibitor in human patients^53^. A molecule with a short half-life was hypothesized to garner additional control of the therapeutic index while not compromising efficacy, given the rapid onset of apoptosis observed with MCL-1 inhibitors in preclinical models^24,25^. Further, we hypothesized that a molecule requiring a low human dose would limit the possibility of undesired pharmacology while expediting the identification of safe IV-based media in the face of challenging chemical properties^54^. These considerations prompted the in silico and structure-based design of ABBV-467. The macrocyclic core structure of ABBV-467 serves to orient the molecule into a highly active conformation; this is evidenced by the reported target affinity and potency against MCL-1-dependent cell lines, as well as the durable in vivo efficacy observed following compound administration. Consistent with published reports of MCL-1 inhibitors, ABBV-467 is highly selective for the target protein.

As previously reported, knockout of *mcl1* in mice results in peri-implantation embryonic lethality^55^. Subsequent mouse studies employing conditional and tissue-specific deletion of *mcl1* indicated that this protein is required for the normal function of multiple tissues. These include cardiomyocytes, where *mcl1* deletion causes morphologic alterations to mitochondria and lethal cardiac dysfunction. Because of the reported impact of *mcl1* deletion within cardiomyocytes in mice^56,57^, particular attention was given to the effects of ABBV-467 treatment on any parameters of cardiovascular function. In repeat-dose studies in rodents and dogs, no deleterious effects on cardiovascular function or heart pathology were observed following administration of ABBV-467. Moreover, in single-dose studies specifically evaluating acute cardiovascular function in dogs, no remarkable effects were observed up to micromolar plasma concentrations. ABBV-467 has substantially reduced affinity for the murine *mcl1* as reported for other inhibitors^24^, while high affinity (Ki <100 pM) was observed to rat, canine and monkey MCL-1 (Supplementary Table S10). These data indicate either that pharmacologic inhibition does not phenocopy the results of genetic *mcl1* ablation in murine cardiomyocytes, or the impact of MCL-1 modulation is not conserved across species. The non-clinical profile of ABBV-467 in rat and dog toxicology/safety pharmacology studies indicated no cardiovascular risk with ABBV-467 and supported the initiation of phase 1 clinical trials. However, considering the published literature on *mcl1* knockout mice, substantial caution was exercised in the phase 1 protocol, with multiple steps taken to facilitate close monitoring of cardiac function in phase 1 patients.

Although the study was terminated by the sponsor with only 8 patients enrolled, there was evidence of disease control in a female patient with high-risk myeloma as stratified by the presence of t(4;14) and *1q* amplification chromosomal abnormalities. This patient was heavily pretreated and refractory to all prior treatment regimens. While on a 0.16-mg/kg dose of ABBV-467, the patient’s disease was stabilized for 8 months, which was substantially longer than for her most recent lines of therapy. It is conceivable that the presence of the *1q* amplification rendered this patient’s disease more susceptible to MCL-1 inhibition even in the face of a low ABBV-467 dose. No other patients had the *1q* amplification at baseline. *MCL1* is one of several genes located on the *1q21* locus, and previous studies have reported greater sensitivity of *1q*-amplified MM patient samples to MCL-1 inhibitors^58^. However, additional studies have failed to identify this association^23^. This discrepancy could be the result of multiple factors, including low sample number or the confounding impact of other co-mutations. More clinical data will be required to understand the relationship between *1q* amplification and sensitivity to MCL-1 inhibitors.

Overall, the results of our phase 1 study demonstrated that ABBV-467 showed linear pharmacokinetics and a manageable safety profile in human patients at the doses studied. However, increased levels of troponin I/T, a diagnostic marker of cardiac toxicity, were observed in 4 (50%) patients, with 1 at each dose level studied. The majority of cases (3 of 4) were mild in severity (grade 1) and normalized over time. One patient did have a grade 3 elevation that resulted in discontinuation of ABBV-467. While this patient had a normal ECG at baseline, the baseline ejection fraction of 50% was just at the threshold of eligibility for trial enrollment; there was also left ventricular regional wall hypokinesis at baseline, which could indicate that the patient may have already been at risk of developing heart disease. After the troponin elevation, the ECG and echocardiogram remained largely unchanged and the ejection fraction was 47%; this small (3%) decrease was not considered clinically meaningful. Of note, a high-sensitivity troponin assay was employed in this study; compared with historical assays, the high-sensitivity assay may have identified troponin level alterations that were previously undetectable in patients^59^. Finally, per standard protocol, troponin levels were not measured in the GLP toxicology studies with ABBV-467 based on the lack of cardiovascular findings in previous repeat dose toxicology and safety pharmacology studies. It is thus unclear if the troponin elevations observed in human patients treated with ABBV-467 would have been predictable from preclinical species assessments.

Concerns about cardiotoxicity in patients who have received MCL-1 inhibitor treatment have recently been reported. In late 2019, the United States Food and Drug Administration placed a clinical hold on a phase 1 study (NCT03465540) of the orally administered MCL-1 inhibitor AMG 397 following cardiac toxicity-related safety signals^60^. While the exact nature of the toxicity was not disclosed, the trial sponsor voluntarily halted a phase 1 study of the related IV-administered compound AMG 176 (NCT02675452)^61^. The latter trial was subsequently reinitiated with several modifications to inclusion/exclusion criteria and cardiac monitoring, including criteria specific to troponin levels as defined by a clinical assay. Similar criteria were subsequently incorporated into an ongoing phase 1 trial evaluating structurally distinct MCL-1 inhibitor AZD5991 (NCT03218683) that was later suspended for undisclosed complications related to safety^62^. Finally, several more recently initiated phase 1 trials evaluating MCL-1 inhibitors (NCT04629443, NCT04702425, NCT05107856) include criteria related to troponin levels.

These reports, along with the clinical data provided in this ABBV-467 FIH study, suggest that pharmacologic inhibition of MCL-1 leads to alteration of troponin levels in human patients through a currently unknown mechanism. In the ABBV-467 FIH clinical study, 7 of 8 (88%) patients reported any-grade AEs, with no cardiac disorders of any grade and no grade 5 toxicities observed in any dose cohort. Diarrhea, nausea, increased aspartate aminotransferase, increased troponin I, and increased troponin T (25% each) were the most common TEAEs. Five (63%) patients reported treatment-related AEs (nausea, increased levels of alanine aminotransferase, aspartate aminotransferase, amylase, troponin T, and troponin I); all were consistent with the mechanism of action of treatment. The absence of vascular findings in our study may indicate a direct effect on cardiomyocytes as opposed to ischemic events. However, the data remain too sparse for definitive conclusions. Additional cardiac monitoring such as baseline and post-baseline cardiac magnetic resonance imaging as a clinical diagnostic tool should be a consideration in future trial protocols using this class of drugs. In addition, patients with heart disease such as cardiac amyloidosis and ischemic heart disease need to be made aware of potential myocardial damage when receiving MCL-1 inhibitors. Consequently, these disease groups should be included in the study exclusion criteria. Similarly, the value of restricting eligibility criteria related to cardiovascular health, prolonging the study drug infusion time, or adding supportive care will need to be assessed in the future.

### Supplementary information

Supplementary Information

Supplementary Data 1

Supplementary Data 2

Supplementary Data 3

Supplementary Data 4

Supplementary Data 5

Supplementary Data 6

Supplementary Data 7

Supplementary Data 8

Supplementary Data 9

Description of Additional Supplementary Files

Reporting Summary

## Data Availability

AbbVie is committed to responsible data sharing regarding the clinical trials we sponsor. This includes access to anonymized, individual and trial-level data (analysis data sets), as well as other information (e.g., protocols and Clinical Study Reports), as long as the trials are not part of an ongoing or planned regulatory submission. This includes requests for clinical trial data for unlicensed products and indications. These clinical trial data can be requested by any qualified researchers who engage in rigorous, independent scientific research, and will be provided following review and approval of a research proposal and Statistical Analysis Plan (SAP) and execution of a Data Sharing Agreement (DSA). Data requests can be submitted at any time and the data will be accessible for 12 months, with possible extensions considered. For more information on the process, or to submit a request, visit the following link: https://vivli.org/ourmember/abbvie/. The atomic coordinates for the structures of Mcl-1 in complex with compound 1, compound 2 and ABBV-467 have been deposited in the Protein Data Bank under the accession codes 8EKX, 8EL0 and 8EL1, respectively. Source data for Table 1 and Figs. 2–6 can be found in Supplementary Data 3–9. All other data are available from the corresponding author on reasonable request. The study protocol can be found in the Supplementary Information.
