# Supplementary material for: Selective MCL-1 inhibitor ABBV-467 is efficacious in tumor models but is associated with cardiac troponin increases in patients
Source: Commun Med (Lond). 2023 Oct 25;3:154. doi: 10.1038/s43856-023-00380-z (PMC10600239; doi:10.1038/s43856-023-00380-z)
Supplement: Supplementary file 1 — Supplementary Information [file 43856_2023_380_MOESM1_ESM.pdf]

Supplementary Information

Selective MCL-1 inhibitor ABBV-467 is efficacious in tumor models but is associated with cardiac troponin increases in patients

Junichiro Yuda[1], Christine Will[2], Darren C. Phillips[2], Linu Abraham[2], Cory Alvey[2], Abraham Avigdor[3,4], Wayne Buck[2], Lauren Besenhofer[2], Erwin Boghaert[2,5], Dong Cheng[2], Dan Cojocari[2], Kelly Doyle[2], T. Matthew Hansen[2], Kevin Huang[2], Eric F. Johnson[2], Andrew S. Judd[2], Russell A. Judge[2], John C. Kalvass[2], Aaron Kunzer[2], Lloyd T. Lam[2], Rachel Li[2], Ruth L. Martin[2], Anthony Mastracchio[2], Mike Mitten[2,6], Adam Petrich[2,7,8], Jin Wang[2], James E. Ward[2,9], Haichao Zhang[2], Xilu Wang[2], Johannes E. Wolff[2,10], Katherine M. Bell-McGuinn[2,11], Andrew J. Souers[2,*]

Affiliations:

[1]National Cancer Center Hospital East, Kashiwa, Japan.

[2]AbbVie Inc., North Chicago, IL, USA.

[3]Institute of Hematology, Sheba Medical Center, Ramat Gan, Israel.

[4]Sackler Faculty of Medicine, Tel Aviv University, Tel Aviv, Israel.

[5]Present address: Pleasant Prairie, WI, USA.

[6]Present address: Beach Park, IL, USA.

[7]Northwestern University, Chicago, IL, USA.

[8]Present address: Daiichi Sankyo, Basking Ridge, NJ, USA.

[9]Present address: Seagen Inc., Bothell, WA, USA.

[10]Present address: Replimmune, Puyallop, WA, USA.

[11]Present address: Zionsville, IN, USA.

Supplementary Methods……………………………………………………………………………………………. 4

Chemistry……. 4

Chemical synthesis of compound 2 and ABBV-467……………………………………………………. 5

NMR and MS spectra for compounds S-1 through S-51 and ABBV-467………………………… 32

Phase 1 clinical trial………………………………………………………………………………………………. 161

Supplementary Tables and Figures…………………………………………………………………………. 164

Supplementary Table S1………………………………………………………………………………………. 164

Supplementary Table S2………………………………………………………………………………………. 164

Supplementary Table S3………………………………………………………………………………………. 165

Supplementary Table S4………………………………………………………………………………………. 166

Supplementary Table S5………………………………………………………………………………………. 166

Supplementary Table S6………………………………………………………………………………………. 167

Supplementary Table S7………………………………………………………………………………………. 169

Supplementary Table S8………………………………………………………………………………………. 171

Supplementary Table S9………………………………………………………………………………………. 172

Supplementary Table S10…………………………………………………………………………………….. 173

Supplementary Fig. S1………………………………………………………………………………………….. 174

Supplementary Fig. S2………………………………………………………………………………………….. 175

Supplementary Fig. S3………………………………………………………………………………………….. 176

Supplementary Fig. S4………………………………………………………………………………………….. 177

Supplementary Fig. S5………………………………………………………………………………………….. 178

Supplementary Fig. S6………………………………………………………………………………………….. 179

Supplementary Fig. S7………………………………………………………………………………………….. 180

Supplementary Fig. S8………………………………………………………………………………………….. 181

Supplementary References…………………………………………………………………………………. 182

Clinical Study Protocol…………………………………………………………………………………………. 183

Supplementary Methods

Chemistry

General. Hydrogen-1 nuclear magnetic resonance spectra were obtained on a Varian UNITY or Inova (500 MHz), Varian UNITY (400 MHz), or Varian UNITY plus or Mercury (300 MHz) instrument. Chemical shifts are reported as values (ppm) downfield relative to tetramethylsilane as an internal standard, with multiplicities reported in the usual manner. Mass spectral analyses were performed on a Finnigan SSQ 7000 GC/MS mass spectrometer using different techniques, including electrospray ionization, desorption chemical ionization, and atmospheric pressure chemical ionization, as specified for individual compounds. Exact mass measurements were performed on a Finnigan FTMS Newstar T70 mass spectrometer. The compound is determined to be "consistent" with the chemical formula if the exact mass measurement is within 5.0 ppm relative mass error of the exact monoisotopic mass.
Analytic liquid chromatography-mass spectrometry was performed on a Finnigan Navigator Mass Spectrometer and Agilent 1100 HPLC system running Xcalibur 1.2 and Open-Access 1.3 software. The mass spectrometer was operated under positive atmospheric pressure chemical ionization conditions. The HPLC system comprised an Agilent Quaternary pump, degasser, column compartment, autosampler, and diode-array detector, with a Sedere Sedex 75 evaporative light-scattering detector. The column used was a Phenomenex Luna Combi-HTS C8(2) 5µm 100Å (2.1 mm × 30 mm), utilizing Method A or Method B, as detailed below. Trifluoroacetic acid method (Method A): a gradient of 10–100% acetonitrile (solvent 1) and 0.1% trifluoroacetic acid in water (solvent 2) was used, at a flow rate of 2 mL/min (0–0.1 min 10% solvent 1, 0.1–2.6 min 10–100% solvent 1, 2.6–2.9 min 100% solvent 1, 2.9–3.0 min 100–10% solvent 1). Ammonium acetate method (Method B): a gradient of 10–100% acetonitrile (solvent 1) and 10 mM NH_4OAc in water (solvent 2) was used, at a flow rate of 1.5 mL/min (0–0.1 min 10% solvent 1, 0.1–3.1 min 10–100% solvent 1, 3.1–3.9 min 100% solvent 1, 3.9–4.0 min 100–10% solvent 1). All final analogues were >95% pure as determined by these methods.

Preparative reverse-phase high-performance liquid chromatography (HPLC) was performed on an automated Gilson HPLC system, using a Symmetry Prep Shield RP18 prep cartridge, 250 mm × 21.20 mm i.d., 10 µm, and a flow rate of 25 mL/min; at 214, 245 nm; mobile phase A, 0.1% trifluoroacetic acid in water; mobile phase B, acetonitrile, using a linear gradient 0–70% of B over 40 minutes, unless otherwise stated.

Chemical synthesis of compound 2 and ABBV-467.

6-iodothieno[2,3-d]pyrimidin-4(3H)-one (S-1). Acetic acid (312 mL), sulfuric acid (9.37 mL), and water (63 mL) were combined with stirring. Thieno[2,3-*d*]pyrimidin-4(3*H*)-one (50 g, 329 mmol), periodic acid (37.4 g, 164 mmol), and iodine (75 g, 295 mmol) were added sequentially, and the mixture became slightly endothermic. A heating mantle was added and the reaction mixture was ramped up to 60°C. Midway through, the temperature climbed to 68–69°C. The heating mantle was removed, and the temperature was maintained at 70°C by self-heating for about 45 minutes. LC-MS indicated a single peak corresponding to the title compound. The reaction mixture was cooled to room temperature. The resulting suspension was filtered, and washed with 5:1 acetic acid: water (three times), and diethyl ether (five times) to provide the title compound, which was used in the next step without further purification (62.7 g, 69%). ^{1}H NMR (400 MHz, dimethyl sulfoxide-d_6) δ ppm 12.80-12.41 (m, 1H), 8.10 (s, 1H), 7.66 (s, 1H). MS (ESI) *m/z* 277.9 (M-H)$^-$. ^{1}H NMR, hydrogen-1 nuclear magnetic resonance; LC-MS, liquid chromatography-mass spectrometry.

4-chloro-6-iodothieno[2,3-d]pyrimidine (S-2). Phosphorous oxychloride (37 mL, 397 mmol) and *N*,*N*-dimethylaniline (11.5 mL, 90 mmol) were combined, and **S-1** (25 g, 90 mmol) was added over a few minutes. The reaction mixture was stirred at about 105°C for 1.5 hours. An aliquot was analyzed by LC-MS, which indicated the reaction mixture was complete. The suspension was cooled to 5–10°C, filtered, and washed with heptanes. The crude filter cake was dumped into ice water with rapid stirring. The mixture was stirred for about 30 minutes, filtered, and washed with additional water (three times) and diethyl ether (three times). The solid was dried on the filter bed overnight to provide the title compound and was used in the next step without further purification (20.5 g, 77%). ^{1}H NMR (400 MHz, dimethyl sulfoxide-d_6) δ ppm 8.89 (s, 1H), 7.95 (s, 1H). MS (ESI) *m/z* 296.8 (M-H)$^-$. ^{1}H NMR, hydrogen-1 nuclear magnetic resonance; LC-MS, liquid chromatography-mass spectrometry.

5-bromo-4-chloro-6-iodothieno[2,3-d]pyrimidine (S-3). S-2 (20.5 g, 69.1 mmol) was taken up in acetonitrile (173 mL) and *N*-bromosuccinimide (13.54 g, 76 mmol) was added followed by tetrafluoroboric acid-dimethyl ether complex (2 mL, 16.4 mmol). While the reaction mixture was stirring, the temperature slowly climbed, reaching 25.5°C after 30 minutes. The reaction mixture was allowed to stir overnight at room temperature. An additional 0.4 equivalents of *N*-bromosuccinimide was added, followed by tetrafluoroboric acid-dimethyl ether complex (2 mL, 16.4 mmol), and the reaction mixture was stirred for an additional 5 hours. The reaction mixture was cooled in an ice bath to about 5°C (internal) and filtered. The solids were washed with acetonitrile (twice) and dried on the filter bed overnight. The title compound was used in the next step without further purification. ^{1}H NMR (400 MHz, dimethyl sulfoxide-d_6) δ ppm 8.93 (s, 1H). MS (ESI) *m/z* 421.0 (M+48)$^-$. ^{1}H NMR, hydrogen-1 nuclear magnetic resonance.

5-bromo-4-chloro-6-(4-fluorophenyl)thieno[2,3-d]pyrimidine (S-4).

(Tris(dibenzylideneacetone)dipalladium(0)) (7.32 g, 7.99 mmol), di-*tert*-butyl(2',4',6'-triisopropyl-[1,1'-biphenyl]-2-yl)phosphine (7.47 g, 17.58 mmol), tripotassium phosphate (181 g, 852 mmol), (4-fluorophenyl)boronic acid (89 g, 639 mmol), and S-3 (200 g, 533 mmol) were combined in a three-neck, 5-L round bottom flask, fitted with a water condenser, thermocouple/J-KEM, overhead stirring, and an argon gas inlet**.** The solids were flushed with argon for 40 minutes. Tetrahydrofuran (1705 mL) and water (426 mL) were combined into a 3-L round bottom flask. The contents were sparged with argon for 30 minutes. The solvent mixture was cannulated into the flask containing the solids. A sharp temperature increase to 37°C was observed. The temperature was set to 64°C (internal), and the reaction mixture was stirred overnight (16 hours) at 64°C under a light positive flow of argon. The reaction mixture was cooled to 38°C, and 200 mL water was added with stirring (overhead). Stirring was continued for 2 hours, and the solids were filtered and washed with water. A second crop was obtained from the filtrate and was combined with the first crop. The combined solids were taken up in hot tetrahydrofuran (2 L), stirred with 20 g thiosilica gel and 20 g charcoal for 30 minutes, and filtered through a pad of diatomaceous earth. The filtrate was concentrated to provide the title compound (145 g, 78 %). ^{1}H NMR (400 MHz, chloroform-*d*) δ ppm 8.86 (s, 1H), 7.75-7.58 (m, 2H), 7.22 (t, 2H). MS (ESI) *m/z* 344.8 (M+H)$^{+}$. ^{1}H NMR, hydrogen-1 nuclear magnetic resonance.

2-methoxybenzimidamide hydrochloride (S-5). A dried 12-L five-necked flask equipped with a mechanical stirrer, a gas inlet with tubing leading to a nitrogen regulator, a gas inlet adapter with tubing leading to a bubbler, and an internal temperature probe (J-KEM controlled) was charged with ammonium chloride (86 g, 1600 mmol). The solid was mixed under nitrogen with anhydrous toluene (2 L). The mixture was cooled to –12.3°C in an ice/methanol bath. To the mixture was added, via cannula, 2.0 M trimethylaluminum in toluene (800 mL, 1600 mmol). Upon addition of the trimethylaluminum, the mixture started to smoke immediately and gas was evolved. The temperature of the reaction mixture rose to a high of –0.4°C during the addition, and the addition took a total of about 60 minutes. After all the trimethylaluminum was added, the mixture was allowed to stir at 20°C for 3 hours. To the mixture was added 2-methoxybenzonitrile (107 g, 800 mmol) as a liquid (had been melted in bath at about 45°C). Once the 2-methoxybenzonitrile was added, the reaction mixture was heated at 90°C overnight with the use of a heating mantle controlled by a J-KEM. The reaction flask was fitted with a vigreux condenser. Thin-layer chromatography in 50% ethyl acetate/heptane indicated a major baseline product. The reaction mixture was cooled to –8.7°C in an ice/methanol bath, and to the cold mixture was added 4 L of methanol, dropwise via an addition funnel. The addition evolved gas and was exothermic. The temperature of the reaction mixture reached a high of 7.9°C, and the addition took a total of about 1 hour. After all the methanol was added, the mixture was allowed to stir for 3 hours at 20°C. The reaction mixture was filtered through filter paper on a benchtop filter. The solids collected were washed with additional methanol (2 L). The filtrate was concentrated. The crude material was mixed with 500 mL of ethyl acetate. The mixture was sonicated for 30 minutes and was stirred for another 30 minutes. The solids were filtered off and washed with more ethyl acetate. The solids collected were air dried for an

hour and then dried under high vacuum for 2 hours to provide the title compound (127 g, 85%). ^{1}H NMR (400 MHz, dimethyl sulfoxide-d_6) δ ppm 9.23 (bs, 2H), 7.69 (bs, 1H), 7.63 (ddd, 1H), 7.55 (dd, 1H), 7.25 (dd, 1H), 7.12 (td, 1H), 3.87 (s, 3H). MS (DCI) *m/z* 151.0 $(M+H)^+$. ^{1}H NMR, hydrogen-1 nuclear magnetic resonance.

4-(dimethoxymethyl)-2-(2-methoxyphenyl)pyrimidine (S-6). An oven-dried 5-L three-neck flask equipped with a mechanical stirrer, nitrogen inlet into a reflux condenser and outlet to a bubbler, and an internal temperature probe (J-KEM controlled) was charged with S-5 (126.9 g, 680 mmol) and (*E*)-4-(dimethylamino)-1,1-dimethoxybut-3-en-2-one (177 g, 1020 mmol). Anhydrous methanol (1360 mL) was added. To the mixture at room temperature under nitrogen was added solid sodium methoxide (257 g, 4759 mmol) in portions over 20 minutes. The temperature of the reaction went up from 18.6°C to 35.7°C during the addition. Once the exotherm stopped, the reaction mixture was heated to 65°C overnight. The reaction mixture was cooled, and concentrated. The residue was mixed with ethyl acetate (800 mL), and water (1 L) was added carefully. The two-phase mixture was sonicated for about 30 minutes to dissolve all the solids. The layers were separated, and organic layer was washed with saturated aqueous NH_4Cl mixture. The combined aqueous extracts were extracted one time with ethyl acetate. The combined organic extracts were washed with brine, dried with Na_2SO_4, filtered, and concentrated. The residue was dissolved in a small amount of dichloromethane (30 mL) and loaded onto a 2.0-L plug of silica in a 3-L Büchner funnel that had been equilibrated with 40% ethyl acetate/heptane. The desired product was eluted with 40% to 50% ethyl acetate/heptane. The fractions containing the desired product were combined and concentrated to provide the title compound (131.4 g, 74%). ^{1}H NMR (500 MHz, dimethyl sulfoxide-d_6) δ ppm 8.93 (d, 1H), 7.54 (dd, 1H), 7.50-7.43 (m, 2H), 7.16 (dd, 1H), 7.06 (td, 1H), 5.31 (s, 1H), 3.76 (s, 3H), 3.38 (s, 6H). MS (DCI) *m/z* 261.0 $(M+H)^+$. ^{1}H NMR, hydrogen-1 nuclear magnetic resonance.

(2-(2-methoxyphenyl)pyrimidin-4-yl)methanol (S-7). A mixture of S-6 (14.7 g, 56.5 mmol) in 110 mL HCl in dioxane (4M mixture) and 110 mL water was heated at 50°C for 14 hours. The mixture was cooled to 0°C, and ground NaOH (17.60 g, 440 mmol) was added in portions. The pH was adjusted to 8 using 10% K_2CO_3 aqueous mixture. $NaBH_4$ (4.27 g, 113 mmol) was added in portions. The mixture was stirred at 0°C for 45 minutes. The mixture was carefully quenched with 150 mL saturated aqueous NH_4Cl and was stirred at 0°C for 30 minutes. The mixture was extracted with ethyl acetate (5 × 150 mL), washed with brine, dried over $MgSO_4$, filtered, and concentrated. The solid residue was triturated in 30 mL ethanol to give a first crop of the title compound. The filtrate was concentrated, and the residue was purified on a silica gel column (120 g, 55–100% ethyl acetate in heptanes, dry load) to give a second crop of the title compound. The first and second crops (9.25 g, 76 %) of the title compound were combined for the subsequent step. ^{1}H NMR (500 MHz, dimethyl sulfoxide-d_6) δ ppm 8.84 (d, 1H), 7.49 (m, 2H), 7.44 (ddd, 1H), 7.13 (dd, 1H), 7.04 (td, 1H), 5.65 (t, 1H), 4.60 (dd, 2H), 3.75 (s, 3H). MS (DCI) *m/z* 217.0 $(M+H)^+$; ^{13}C NMR (101 MHz, DMSO) δ 171.30, 164.82, 157.81, 157.59, 131.30, 130.99, 129.24, 120.55, 115.55, 112.64, 63.93, 56.13, 40.57, 40.36, 40.16, 39.95, 39.74, 39.53, 39.32; HRMS (ESI-QTOF+) $[M+H]^+$ calc. for

$C_{12}H_{13}N_2O_2$ m/z, 217.0972 found 217.0970 (0.6 ppm). ^{1}H NMR, hydrogen-1 nuclear magnetic resonance, ^{13}C NMR, carbon-13 nuclear magnetic resonance, HRMS, high resolution mass spec.

Ethyl 2-acetoxy-3-(2-(benzyloxy)phenyl)acrylate (S-8). A 2-L three-necked round bottom flask equipped with an internal temperature probe was charged with ethyl 2-acetoxy-2-(diethoxyphosphoryl)acetate (86 g, 305 mmol) and anhydrous tetrahydrofuran (1 L) at room temperature under nitrogen gas. To the mixture was added cesium carbonate (100 g, 307 mmol) in one portion. The reaction mixture was stirred for about 20 minutes, and 2-(benzyloxy)benzaldehyde (50 g, 236 mmol) was added as a solid in one portion. The slurry was stirred vigorously overnight at room temperature. Thin-layer chromatography in 10% ethyl acetate/heptane indicted the reaction was about 60–70% complete. Another 0.5 equiv. of ethyl 2-acetoxy-2-(diethoxyphosphoryl)acetate and cesium carbonate were added, and the reaction mixture was stirred overnight. Thin-layer chromatography indicated the reaction mixture was complete. The reaction mixture was cooled to about 0°C in an ice bath, and the reaction mixture was quenched with water (500 mL) in portions. Water was added such that the temperature of the reaction mixture was maintained below 10°C. The reaction mixture was diluted with ethyl acetate (500 mL), and the mixture was stirred for 30 minutes. The mixture was poured into a separatory funnel and was further diluted with ethyl acetate and water to a total volume of 2.6 L. The organic layer was separated, washed with brine, dried with Na_2SO_4, filtered, and concentrated. The residue was dissolved in 2:1 heptane/dichloromethane and was purified on a 2-L silica gel plug equilibrated with 100% heptane. The material was eluted with 5–10% ethyl acetate/heptane. The pure fractions were combined, and the solvents were removed under reduced pressure to provide the title compound. NMR indicated the material was about a 2:1 mix of E and Z isomer (67.7 g, 84%). ^{1}H NMR (501 MHz, dimethyl sulfoxide-d_6) δ ppm 7.71 (m, 2H), 7.50-7.25 (m, 12H), 7.20 (dd, 1H), 7.11 (dd, 0.5H), 7.04 (m, 1H), 6.94 (m, 1H), 5.22 (s, 2H), 5.14 (s, 1H), 4.20 (q, 2H), 4.01 (q, 1H), 2.30 (s, 3H), 2.21 (s, 1.5H), 1.24 (t, 3H), 0.99 (t, 1.5H). MS (ESI) *m/z* 340.8 (M+H ^{1}H NMR, hydrogen-1 nuclear magnetic resonance.

(R)-ethyl 2-acetoxy-3-(2-(benzyloxy)phenyl)propanoate (S-9). (*Z*)-S-8 (1.0 kg, 2.94 mol) in methanol (5.0 L) was degassed with bubbling argon for 30 minutes and then transferred to a 2-gallon Parr stainless steel reactor. The reactor was purged with argon for 30 minutes. At that time, 1,2-bis((2*R*,5*R*)-2,5-diethylphospholano)benzene(cyclooctadiene)rhodium(I) tetrafluoroborate (17.8 g, 21 mmol) was added, and the vessel was sealed and purged further with argon. The vessel was pressurized to 120 psi with hydrogen. The mixture was stirred under 120 psi of hydrogen with no external heating applied. After 70 hours, the reactor was vented and purged four times with argon. High-performance liquid chromatography indicated complete conversion to the desired product. The mixture was transferred to a flask, and the solvents were concentrated. To the residue was added 1:1 heptane/ethyl acetate, and the clear oil turned into a cloudy mix. The flask was swirled, and a sludge crashed out. With the swirling, much of the sludge stuck to the side of the flask. The liquid was poured through a plug of silica (1 L), eluting with 1:1 heptane/ethyl acetate. The filtrate that contained the title

compound was concentrated to provide the title compound (896 g, 89 %). ^{1}H NMR (400 MHz, Chloroform-*d*) δ ppm7.47 (m, 2H), 7.39 (m, 2H), 7.32 (m, 1H), 7.19 (m, 2H), 6.90 (m, 2H), 5.31 (dd, 1H), 5.12 (m, 2H), 4.13 (qq, 2H), 3.35 (dd, 1H), 3.06 (dd, *J* = 13.8, 9.2 Hz, 1H), 2.03 (s, 3H), 1.17 (t, 3H). MS (ESI) *m/z* 360.0 $(M+NH_4)^+$ ^{1}H NMR, hydrogen-1 nuclear magnetic resonance.

(R)-ethyl 2-acetoxy-3-(2-hydroxyphenyl)propanoate (S-10). S-9 (896 g, 2617 mmol) in ethanol (4.3 L) was added to wet 5% palladium on carbon catalyst (399.7 g) in a 2-gallon Parr stainless steel reactor. The reactor was purged with argon, and the mixture was stirred at 600 RPM under 50 psi of hydrogen at 25°C for 12 hours. LC-MS indicated a single peak corresponding to the title compound. The mixture was filtered through filter paper and followed by a 0.2-micron polypropylene membrane. The mixture was concentrated to produce an oil that formed a precipitate upon standing overnight. The precipitate were transferred into a 12-L three-neck round bottom flask equipped with a mechanical stirrer and temperature probe (J-KEM controlled). The solids were mixed in 5 L (about 0.5 M) of heptane. The mixture was heated to about 74°C. To the hot mixture was added isopropyl acetate. The isopropyl acetate was added in 100-mL aliquots up to about 500 mL. The solids were almost all dissolved. Isopropyl acetate was added in 10-mL aliquots until a clear mixture formed. A total of 630 mL of isopropyl acetate was used. The mixture was heated to about 80°C for about 10 minutes. The heat was turned off but the heating mantle was left on. Stirring was slowed to a low rate. The mixture was allowed to cool slowly overnight. The mixture was filtered, and the solids were washed with heptane, and dried for a few hours. The filtrate was concentrated, and the process was repeated on the residue using the same conditions to produce additional title compound. The two batches of title compound were combined (520 g, 79%). Chiral HPLC of the combined material on a Gilson HPLC system using a ChiralPak AD-H column (4.6 mm × 250 mm, 3 μm) and a 5–50% ethanol/heptane gradient over 15 minutes indicated a single peak with a retention time of 8.9 minutes. ^{1}H NMR (400 MHz, dimethyl sulfoxide-d_6) δ ppm 9.53 (s, 1H), 7.06 (m, 2H), 6.79 (m, 1H), 6.71 (td, 1H), 5.11 (dd, *J* = 8.3, 6.0 Hz, 1H), 4.05 (q, 2H), 3.07 (dd, 1H), 2.95 (dd, 1H), 2.00 (s, 3H), 1.09 (t, 3H). MS (DCI) *m/z* 270.0 $(M+NH_4)^+$. ^{1}H NMR, hydrogen-1 nuclear magnetic resonance; HPLC, high-performance liquid chromatography; LC-MS, liquid chromatography-mass spectrometry.

(R)-ethyl 2-acetoxy-3-(5-bromo-2-hydroxyphenyl)propanoate (S-11). A dried 5-L three-neck jacketed flask equipped with a mechanical stirrer and an internal temperature probe controlled by a Huber Ministat 230 Chiller was charged with S-10 (200 g, 793 mmol). To this was added anhydrous tetrahydrofuran (3.3 L) at room temperature under nitrogen. The mixture was cooled to –20.4°C using a chiller. To the cooled mixture was added concentrated sulfuric acid (4.23 mL). The temperature of the reaction rose to –19.8°C. *N*-bromosuccinimide (143 g, 801 mmol) was added in portions over a period of 10 minutes. The temperature rose from –20.3°C to –20.0°C during the addition. The reaction mixture was stirred overnight at –20°C. LC-MS indicated the reaction mixture was about 70% complete. The reaction mixture was warmed to 0°C with the use of the chiller and was stirred for 5 hours at 0°C. LC-MS indicated reaction mixture was >90% complete. The reaction mixture was warmed to 20°C

with use of the chiller. After 1 hour at 20°C, LC-MS showed no sign of starting material and one major product. The reaction mixture was cooled to 0°C with use of the chiller. The reaction mixture was quenched with 500 mL of water, and the temperature rose from 0°C to about 8°C. The reaction mixture was diluted with ethyl acetate (1.0 L), and two-phase mixture was stirred for about 20 minutes. The two-phase mixture was poured into a 6-L separatory funnel. One liter of water was added, the mixture shaken, and the layers were separated. The organic layer was washed with saturated aqueous $NaHCO_3$ mixture and brine. The combined aqueous layers were back-extracted one time with ethyl acetate. The combined organic extracts were dried with Na_2SO_4, filtered, and concentrated. Dichloromethane (300 mL) was added to the residue. The mixture was sonicated for 60 minutes. The solids were filtered, washed with a minimum amount of dichloromethane, and dried for an hour to provide the title compound. The solids that formed in the filtrate were filtered and washed with ethyl acetate. The two batches of solids were combined and dried in a vacuum oven at 50°C for 5 hours to provide the title compound (222 g, 85%). Chiral HPLC of this material on a Gilson HPLC system using a ChiralPak AD-H column (4.6 mm × 250 mm, 3 μM) and a 5–50% ethanol/heptane gradient over 30 minutes indicated a single peak with a retention time of 10.6 minutes. ^{1}H NMR (400 MHz, dimethyl sulfoxide-d_6) δ ppm 9.89 (s, 1H), 7.22 (m, 2H), 6.76 (dt, 1H), 5.11 (dd, 1H), 4.06 (qq, 2H), 3.05 (dd, 1H), 2.97 (dd, 1H), 2.02 (s, 3H), 1.10 (t, 3H). MS (ESI) *m/z* 332.8 $(M+H)^+$. ^{1}H NMR, hydrogen-1 nuclear magnetic resonance; HPLC, high-performance liquid chromatography; LC-MS, liquid chromatography-mass spectrometry.

(R)-ethyl 2-acetoxy-3-(5-bromo-2-((2-(2-methoxyphenyl)pyrimidin-4-yl)methoxy)phenyl)propanoate (S-12). A 2-L three-neck round bottom flask equipped with a temperature probe (J-KEM controlled) and stir bar was charged with S-11 (40 g, 121 mmol) and S-7 (31.3 g, 145 mmol) under nitrogen. The solids were dissolved in anhydrous tetrahydrofuran (604 mL) at room temperature, and the reaction mixture was cooled to 2.3°C in an ice bath. To the mixture was added triphenylphosphine (63.4 g, 242 mmol). After about 15 minutes, (*E*)-N^1,N^1,N^2,N^2-tetramethyldiazene-1,2-dicarboxamide (41.6 g, 242 mmol) was added in one portion. The temperature of the reaction did not rise significantly (temperature maintained at 2.5°C). The reaction mixture was stirred at room temperature overnight. Thin-layer chromatography in 50% ethyl acetate/heptane indicated the starting materials were consumed, and a single major product had formed. The reaction mixture was filtered through a fritted Büchner funnel, and the solids collected were washed with ethyl acetate. The filtrate was concentrated. The residue was dissolved in dichloromethane (150 mL) and loaded on to 2.2 L of silica gel that had been equilibrated in 30% ethyl acetate/heptane in a 3-L fritted Büchner funnel. The title compound was eluted with a gradient of 30–60% ethyl acetate in heptane. The early fractions were pure, but the later fractions were contaminated with triphenylphosphine oxide. The pure fractions were combined and were concentrated to provide the title compound. The impure fractions were combined and concentrated. The residue was dissolved in dichloromethane (50 mL) and purified on a Grace Reveleris® X2 MPLC using a Teledyne Isco RediSep Rf gold 750-g silica gel column, eluting with 30–50% ethyl acetate/heptane. Pure fractions from this column were combined with the pure

material from the earlier column. The solid that resulted was mixed with diethyl ether (50 mL). The mixture was sonicated for 30 minutes and stirred for an additional 10 minutes. The solids were filtered off, washed with diethyl ether, and dried to provide the title compound (55.2 g, 86%). Chiral SFC of this material on a HP/Aurora system using a ChiralCel OD-H column (4.6 mm × 100 mm, 5 μM) and a 5–50% methanol gradient over 10 minutes indicated a single peak with a retention time of 5.0 minutes. ^{1}H NMR (400 MHz, dimethyl sulfoxide-d_6) δ ppm 8.94 (d, 1H), 7.55 (m, 2H), 7.45 (m, 3H), 7.16 (m, 1H), 7.06 (m, 2H), 5.27 (d, 2H), 5.18 (dd, 1H), 4.07 (q, 2H), 3.77 (s, 3H), 3.29 (dd, 1H), 3.13 (dd, 1H), 2.02 (s, 3H), 1.10 (t, 3H). MS (ESI) *m/z* 529.1 $(M+H)^+$. ^{1}H NMR, hydrogen-1 nuclear magnetic resonance; MPLC, medium pressure liquid chromatography; SFC, supercritical fluid chromatography.

(R,E)-ethyl 2-acetoxy-3-(5-(hex-1-en-1-yl)-2-((2-(2-methoxyphenyl)pyrimidin-4-yl)methoxy)phenyl)propanoate (S-13). A 1-L three-neck round bottom flask equipped with a stir bar and an internal temperature probe (J-KEM controlled) was charged with S-12 (41 g, 77 mmol), ((*E*)-hex-1-en-1-ylboronic acid (19.82 g, 155 mmol), palladium(II) acetate (1.74 g, 7.74 mmol), dicyclohexyl(2',6'-dimethoxy-[1,1'-biphenyl]-2-yl)phosphine (*S*Phos) (4.45 g, 10.8 mmol), and CsF (35.3 g, 232 mmol). The flask was sealed with septa, and the solids were sparged for 60 minutes by blowing nitrogen over the solids while stirring. Meanwhile in a separate 500-mL round bottom flask was added anhydrous 1,4-dioxane (620 mL), and the mixture was sparged subsurface with nitrogen for 60 minutes. The sparged solvent was then transferred via cannula to the flask with the solids, and the reaction was stirred at room temperature. The temperature rose steadily and slowly from about 17.4°C to about 33°C. The temperature started to go down after about 5 minutes once the high temperature was reached. LC-MS of the reaction mixture after 30 minutes at room temperature produced a single peak that corresponded to the desired product. The reaction mixture was diluted with ethyl acetate and water, and the two-phased mixture was stirred for about 30 minutes with about 3.8 g (~3.0 equiv. based on moles of palladium) of APDTC (ammonium pyrrolidine dithiocarbamate) palladium scavenger. The mixture was filtered through diatomaceous earth with ethyl acetate washes. The filtrate was poured into a separatory funnel, and the layers were separated. The organic layer was washed with brine. The combined aqueous layers were back-extracted one time with ethyl acetate. The combined organic layers were dried with Na_2SO_4, filtered, and concentrated. The residue was purified on a Grace Reveleris X2 MPLC using a Teledyne Isco RediSep Rf gold 750-g silica gel column, eluting with 30–40% ethyl acetate/heptane. The product containing fractions were combined, and the solvents were concentrated to provide the title compound (43.4 g, 105 %). ^{1}H NMR (400 MHz, dimethyl sulfoxide-d_6) δ ppm 8.93 (d, 1H), 7.55 (m, 2H), 7.47 (ddd, 1H), 7.25 (m, 2H), 7.16 (dd, 1H), 7.05 (m, 2H), 6.31 (m, 1H), 6.14 (dt, 1H), 5.26 (d, 2H), 5.18 (dd, 1H), 4.07 (q, 2H), 3.77 (s, 3H), 3.28 (dd, 1H), 3.11 (dd, 1H), 2.16 (m, 2H), 2.01 (s, 3H), 1.37 (m, 4H), 1.09 (t, 3H), 0.89 (t, 3H). MS (ESI) *m/z* 533.3 $(M+H)^+$. ^{1}H NMR, hydrogen-1 nuclear magnetic resonance; LC-MS, liquid chromatograph-mass spectrometry; MPLC, medium pressure liquid chromatography.

(R)-ethyl 2-acetoxy-3-(5-formyl-2-((2-(2-methoxyphenyl)pyrimidin-4-yl)methoxy)phenyl)propanoate (S-14). A 2-L three-neck round bottom flask equipped with a stir bar and an internal temperature probe (J-KEM controlled) was charged with S-13 (41 g, 77 mmol) and iodobenzene diacetate (57.0 g, 177 mmol). Tetrahydrofuran (733 mL) and water (36.7 mL) were added. To the mixture was added 2,6-lutidine (22.41 mL, 192 mmol), followed by addition of solid osmium tetroxide (249 mg, 0.98 mmol). The temperature of the reaction rose from 19.7°C to 33°C. LC-MS of the mixture after 5 minutes indicated a single product had formed that corresponded to desired product. The reaction mixture was quenched with saturated aqueous sodium thiosulfate (500 mL) and was diluted further with ethyl acetate. The mixture was poured into a separatory funnel, and the layers were separated. The organic layer was washed with aqueous sodium thiosulfate and brine, and the washes were combined with the first thiosulfate wash. The combined thiosulfate washes were back-extracted with dichloromethane, and the dichloromethane extract was combined with the original organic extract. The combined organic extracts were then washed with an aqueous copper sulfate mixture (twice) and brine. The organic extracts were dried with Na_2SO_4, filtered, and concentrated. The residue was purified on a Grace Reveleris X2 MPLC using a Teledyne Isco RediSep Rf gold 750-g silica gel column eluting with 50–60% ethyl acetate/heptane. The product-containing fractions were combined, and concentrated. The residue was dissolved in dichloromethane, and the mixture was loaded onto a plug of silica gel (300 mL-dry loaded) in a 500-mL plastic disposable Büchner funnel. The desired product was eluted with 50% to 60% to 70% ethyl acetate/heptane. The pure fractions were combined and concentrated to provide the title compound (29.7 g, 81 %). Chiral HPLC on a Gilson HPLC system using a CHIRALCEL OD-H column (4.6 mm × 250 mm, 5 μM) and a 20–100% ethanol/heptane gradient over 30 minutes indicated a single peak with a retention time of 29.0 minutes. ^{1}H NMR (400 MHz, dimethyl sulfoxide-d_6) δ ppm 9.89 (s, 1H), 8.95 (d, 1H), 7.87 (dd, 1H), 7.80 (d, 1H), 7.57 (m, 2H), 7.47 (ddd, 1H), 7.32 (d, 1H), 7.16 (dd, 1H), 7.06 (td, 1H), 5.42 (m, 2H), 5.22 (dd, 1H), 4.07 (q, 2H), 3.77 (s, 3H), 3.38 (dd, 1H), 3.22 (dd, 1H), 2.00 (s, 3H), 1.09 (t, 3H). MS (ESI) *m/z* 479.3 $(M+H)^+$. ^{1}H NMR, hydrogen-1 nuclear magnetic resonance; HPLC, high-performance liquid chromatography; LC-MS, liquid chromatograph-mass spectrometry; MPLC, medium pressure liquid chromatography.

(R)-ethyl 3-(5-formyl-2-((2-(2-methoxyphenyl)pyrimidin-4-yl)methoxy)phenyl)-2-hydroxypropanoate (S-15). A 500-mL round bottom flask was charged with S-14 (14.7 g, 30.7 mmol). The solid was mixed with anhydrous ethanol (219 mL). To the mixture at room temperature was added a 21% sodium ethoxide mixture in ethanol (0.573 mL, 1.54 mmol). The reaction mixture was stirred for 3 hours at room temperature. LC-MS indicated a single product had formed that corresponded to the desired product. The reaction mixture was quenched with acetic acid (0.352 mL) and was concentrated. The residue was dissolved in dichloromethane and loaded onto a plug of silica gel (300 mL dry loaded) in a 500-mL plastic disposable fritted Büchner funnel. The desired product was eluted with 50% to 60% to 70% ethyl acetate/heptane. The desired product-containing fractions were combined and concentrated to provide the title compound (9.2 g, 69 %). Chiral HPLC on a Gilson HPLC system using a ChiralCel OD-H column (4.6 mm × 250 mm, 5 μM) and a 10–100% ethanol/heptane gradient

over 20 minutes indicated a single peak with a retention time of 19.2 minutes. ^{1}H NMR (400 MHz, dimethyl sulfoxide-d_6) δ ppm 9.88 (s, 1H), 8.94 (d, 1H), 7.80 (m, 2H), 7.58 (m, 2H), 7.47 (ddd, 1H), 7.29 (d, 1H), 7.17 (dd, 1H), 7.06 (td, 1H), 5.61 (d, 1H), 5.40 (d, 2H), 4.39 (ddd, 1H), 4.07 (q, 2H), 3.77 (s, 3H), 3.23 (dd, 1H), 2.95 (dd, 1H), 1.12 (t, 3H). MS (ESI) *m/z* 437.2 $(M+H)^+$. ^{1}H NMR, hydrogen-1 nuclear magnetic resonance; HPLC, high-performance liquid chromatography; LC-MS, liquid chromatograph-mass spectrometry.

(R)-ethyl 2-((5-bromo-6-(4-fluorophenyl)thieno[2,3-d]pyrimidin-4-yl)oxy)-3-(5-formyl-2-((2-(2-methoxyphenyl)pyrimidin-4-yl)methoxy)phenyl)propanoate (S-16). A 500-mL round bottom flask equipped with a stir bar and temperature probe (J-KEM controlled) was charged with S-15 (9.2 g, 21.1 mmol) and S-4 (7.60 g, 22.1 mmol). Anhydrous *tert*-butanol (162 mL) was added. The mixture was stirred to form a slurry. To the slurry was added cesium carbonate (27.5 g, 84 mmol), and the mixture was heated to 65°C. After 4 hours of heating, thin-layer chromatography in 50% ethyl acetate/heptane indicated one major product with no starting material remaining. The reaction mixture was poured into a combination of saturated aqueous NH_4Cl, brine, and water. The flask was rinsed with ethyl acetate, and more ethyl acetate was added to the aqueous quench. Methanol was added to dissolve most of the solids. The layers were separated, and aqueous layer was extracted one more time with 10% methanol/ ethyl acetate. The combined organic extracts were washed with brine, dried with Na_2SO_4, filtered, and concentrated. The residue was dissolved in dichloromethane and was purified on a Grace Reveleris X2 MPLC using a Teledyne Isco RediSep Rf gold 330-g silica gel column, eluting with 50–70% ethyl acetate in heptane. The pure fractions were collected, and the column was washed with 50–70% ethyl acetate /dichloromethane. The impure fractions were collected from the wash, and they were combined and concentrated. The crude material was purified on a Grace Reveleris X2 MPLC using a Teledyne Isco RediSep Rf gold 220-g silica gel column eluting with 10–30% ethyl acetate/dichloromethane. The product containing fractions from both columns were combined to provide the title compound (11.8 g, 75%). ^{1}H NMR (400 MHz, dimethyl sulfoxide-d_6) δ ppm 9.89 (s, 1H), 8.92 (d, 1H), 8.60 (s, 1H), 8.06 (d, 1H), 7.86 (dd, 1H), 7.73 (m, 2H), 7.61 (d, 1H), 7.44 (m, 4H), 7.33 (d, 1H), 7.11 (d, 1H), 6.99 (t, 1H), 5.78 (dd, 1H), 5.42 (m, 2H), 4.17 (q, 2H), 3.75 (s, 3H), 3.66 (dd, 1H), 3.40 (m, 1H), 1.15 (t, 3H). MS (ESI) *m/z* 743.2 $(M+H)^+$. ^{1}H NMR, hydrogen-1 nuclear magnetic resonance; MPLC, medium pressure liquid chromatography.

2-(4-bromo-2-chlorophenyl)-1,3-dioxane (S-17). A 3-L, three-neck round bottom flask equipped with a Dean-Stark trap and reflux condenser was charged with 4-bromo-2-chlorobenzaldehyde (200 g, 911 mmol), toluene (1519 mL), propane-1,3-diol (110 mL, 1367 mmol) and p-toluenesulfonic acid monohydrate (1.1 g, 4.56 mmol). The reaction mixture was heated to reflux (112°C internal) under Dean-Stark conditions, producing 18 mL of water in about 2 hours. The reaction mixture was cooled to room temperature and poured into saturated aqueous sodium bicarbonate mixture (600 mL) and ethyl acetate (500 mL). The layers were separated, and the aqueous layer was

extracted with ethyl acetate (500 mL, once). The combined organics were dried (anhydrous $MgSO_4$) and treated with charcoal with stirring overnight. The mixture was filtered through a plug of diatomaceous earth and the filtrate was concentrated by rotary evaporation to provide the title compound. The title compound was placed in a vacuum oven overnight at 50°C and was used in the next step without further purification (208 g, 82 %). ^{1}H NMR (400 MHz, chloroform-*d*) δ ppm 7.57 (d, 1H), 7.51 (d, 1H), 7.42 (dd, 1H), 5.74 (s, 1H), 4.29-4.19 (m, 2H), 4.05-3.91 (m, 2H), 2.31-2.13 (m, 1H), 1.43 (dtt, 1H). ^{1}H NMR, hydrogen-1 nuclear magnetic resonance.

2-(4-bromo-2-chloro-3-methylphenyl)-1,3-dioxane (S-18). A five-neck, 5-L round bottom reactor was equipped with overhead stirring, thermocouple/J-KEM, addition funnels, and nitrogen inlet. The assembled reactor was dried with a heat gun under nitrogen. *N*,*N*-diisopropylamine (138 mL, 937 mmol) and tetrahydrofuran (1759 mL) were added to the reactor under a flow of nitrogen. The mixture was cooled to about –76°C (internal) and *n*-butyllithium (369 mL, 923 mmol) was added via addition funnel at a rate necessary to keep the temperature below –68°C. The mixture was stirred at –76°C for 45 minutes to generate a mixture of lithium diisopropylamide (LDA). A tetrahydrofuran (500 mL) mixture of S-17 (208 g, 749 mmol) was added dropwise via addition funnel (over 45 minutes) to the LDA mixture at a rate necessary to keep the temperature below –68°C. The mixture was stirred for 2 hours at –76°C. Iodomethane (70.3 mL, 1124) was added dropwise over 1 hour via addition funnel (very exothermic), and the temperature was kept below –70°C during the addition. The reaction mixture was allowed to warm slowly to room temperature and was stirred overnight. In the morning, water and saturated aqueous ammonium chloride were added along with ethyl acetate (1L). The layers were separated by pump, and the aqueous layer was extracted with ethyl acetate (twice) pumping the top layer into a separatory funnel. The combined organics were dried (anhydrous $MgSO_4$), filtered through diatomaceous earth, and concentrated by rotary evaporation to provide the title compound. GC-MS indicated 11.71 minutes (3%, starting material), 12.82 minutes (8.2%, +Me) and product at 12.5 minutes (88.8%). The material (246 g) was slurried in 550-mL isopropyl alcohol. The mixture was heated to about 80°C. With stirring, the mixture was allowed to cool slowly to room temperature. Copious amounts of solid formed, and the flask was placed in the freezer (–16°C). After 1 hour, the solids were broken up and 400 mL of ice-cold isopropyl alcohol was added. The mixture was slurried and filtered through paper, washing quickly with cold isopropyl alcohol. The solid was allowed to dry on the filter bed and was placed in the vacuum oven for 5 hours (50°C) to provide the title compound (143 g, 65%). ^{1}H NMR (400 MHz, Chloroform-*d*) δ ppm 7.50 (d, 1H), 7.41 (d, 1H), 5.77 (s, 1H), 4.25 (ddd, 2H), 4.01 (td, 2H), 2.53 (s, 3H), 2.34-2.13 (m, 1H), 1.44 (ddt, 1H). MS (ESI) *m/z* 308.0 $(M+NH_4)^+$. ^{1}H NMR, hydrogen-1 nuclear magnetic resonance; GC-MS, gas chromatography-mass spectrometry.

2-(3-chloro-4-(1,3-dioxan-2-yl)-2-methylphenyl)-4,4,5,5-tetramethyl-1,3,2-dioxaborolane (S-19). A three-neck, 5-L round bottom flask fitted with a thermocouple/J-KEM, dry ice acetone bath, overhead stirring, nitrogen inlet and outlets, and addition funnel was charged with S-18 (100 g, 343 mmol) and tetrahydrofuran

(1715 mL) under a positive flow of nitrogen. The mixture was cooled to –76°C (internal) and n-butyllithium (151 mL, 377 mmol) was added dropwise via addition funnel, observing a temperature increase of 5–8°C. The mixture remained clear and colorless and was stirred for 10 minutes at –76°C. 2-Isopropoxy-4,4,5,5-tetramethyl-1,3,2-dioxaborolane (84 mL, 412 mmol) was added dropwise (mixture became exothermic) at such a rate to keep the temperature below –68°C. The mixture was stirred at–76°C for about 30 minutes, warmed to room temperature, and stirred for 3 hours. The reaction mixture was deemed complete by thin-layer chromatography (3:1 heptanes:ethyl acetate). The reaction mixture was concentrated by rotary evaporation. After the volatiles were removed, the water bath was set to 80°C, and the evaporator was switched to high vacuum for 1 hour. Water and ethyl acetate were added to the residue, and the layers were separated. The aqueous layer was extracted with ethyl acetate (once), and the combined organics were dried (anhydrous $MgSO_4$), filtered, and concentrated. The solids were triturated with ice-cold methanol, filtered through paper, and dried on the filter bed and vacuum oven (50°C) to provide the title compound (100 g, 82 %). ^{1}H NMR (400 MHz, dimethyl sulfoxide-d_6) δ ppm 7.59 (d, 1H), 7.45 (d, 1H), 5.76 (s, 1H), 4.14 (ddd, 2H), 3.96 (td, 2H), 2.53 (s, 2H), 2.09-1.94 (m, 1H), 1.50-1.39 (m, 1H), 1.31 (s, 9H). MS (ESI) *m/z* 339.3 $(M+H)^+$. ^{1}H NMR, hydrogen-1 nuclear magnetic resonance.

(R)-ethyl 2-((5-((1S)-3-chloro-4-(1,3-dioxan-2-yl)-2-methylphenyl)-6-(4-fluorophenyl)thieno[2,3-d]pyrimidin-4-yl)oxy)-3-(5-formyl-2-((2-(2-methoxyphenyl)pyrimidin-4-yl)methoxy)phenyl)propanoate (S-20). A 500-mL round bottom flask was charged with S-16 (8.9 g, 11.97 mmol), S-19 (4.86 g, 14.4 mmol), potassium phosphate (7.62 g, 35.9 mmol), and bis(di-*tert*-butyl(4-dimethylaminophenyl)phosphine)dichloropalladium(II) (0.847 g, 1.20 mmol). The flask was sealed, and the solid material was sparged for 60 minutes by blowing nitrogen over the solids with stirring. Separately, in a 250-mL round bottom flask were added tetrahydrofuran (100 mL) and water (25 mL). The mixture was sparged sub-surface with stirring for 60 minutes by bubbling nitrogen through it. The sparged mixture was transferred via cannula to the flask with the solids, and the reaction mixture was stirred overnight at room temperature. LC-MS indicated a single product had formed that corresponded to the desired product. The reaction mixture was diluted with ethyl acetate and water. Ammonium pyrrolidine dithiocarbamate (APDTC, 600 mg, 3 equiv. on the basis of moles of Pd) was added as palladium scavenger, and mixture was stirred for 60 minutes. The mixture was poured into a separatory funnel, and the layers were separated. The organic layer was washed with brine, dried with Na_2SO_4, filtered, and concentrated. The residue was dissolved in dichloromethane and was purified on a Grace Reveleris X2 MPLC using a Teledyne Isco RediSep Rf gold 330-g silica gel column eluting with 20–40% of 25% ethanol in ethyl acetate/heptane. The desired product-containing fractions were combined and concentrated to provide the title compound (9.3 g, 89%). ^{1}H NMR indicated atropisomers in an 8:1 ratio. Analytical HPLC of this material on a HP Agilent instrument using a Thermo Scientific HPLC column (Hypersil Gold AQ, 3.0 um, 150 × 4.6 mm) and a 30-minute gradient run from 10–90% acetonitrile in a trifluoroacetic acid buffer indicated the major atropisomer was 82% of the material with a retention time of 20.2 minutes and the minor atropisomer was 10% of the material with

a retention time of 20.8 minutes. The crude material was carried on in the next step without further purification. ^{1}H NMR (400 MHz, $CDCl_3$) δ 9.83 (s, 1H), 8.90 (d, 1H), 8.57 – 8.49 (m, 1H), 7.77 (dd, 1H), 7.69 (dd, 2H), 7.58 (d, 1H), 7.51 – 7.39 (m, 2H), 7.26 – 7.19 (m, 1H), 7.10 – 7.02 (m, 3H), 6.99 – 6.89 (m, 4H), 5.85 (s, 1H), 5.77 (dd, 1H), 5.43 – 5.23 (m, 3H), 4.30 (dd, 1H), 4.16 – 3.93 (m, 4H), 3.88 (d, 4H), 3.19 (dd, 1H), 2.82 (dd, 1H), 2.23 (dt, 1H), 1.95 (d, 5H), 1.63 (s, 2H), 1.49 – 1.39 (m, 1H), 1.24 (d, 12H), 1.14 (t, 3H). MS (ESI) m/z 875.2 (M+H)+. ^{1}H NMR, hydrogen-1 nuclear magnetic resonance; LC-MS, liquid chromatography-mass spectrometry; MPLC, medium pressure liquid chromatography.

(R)-ethyl 2-((5-((1S)-3-chloro-4-formyl-2-methylphenyl)-6-(4-fluorophenyl)thieno[2,3-d]pyrimidin-4-yl)oxy)-3-(5-formyl-2-((2-(2-methoxyphenyl)pyrimidin-4-yl)methoxy)phenyl)propanoate (S-21). A 100-mL round bottom flask equipped with a stir bar was charged with S-20 (2.98 g, 3.40 mmol). The material was dissolved at room temperature in dichloromethane (6.81 mL). To the mixture was added trifluoroacetic acid (10 mL) and water (0.123 mL). The reaction mixture was stirred overnight at room temperature. Thin-layer chromatography in 20% ethyl acetate/dichloromethane indicated the reaction mixture was complete. The solvents were concentrated with a 50°C bath and house vacuum. The material that resulted was dissolved in ethyl acetate and poured into water. The mixture was diluted further with ethyl acetate and water, and the layers were separated. The organic layer was washed with saturated with aqueous $NaHCO_3$ mixture and brine, dried with Na_2SO_4, filtered, and concentrated. The residue was dissolved in dichloromethane and purified on a Grace Reveleris X2 MPLC using a Grace Reveleris 120-g silica gel column eluting with a 30-minute ramp of 10–30% ethyl acetate/dichloromethane. The desired product-containing fractions were combined, and the solvents were concentrated to provide the title compound (2.18 g, 78 %). ^{1}H NMR indicated an 8 to 1 mixture of atropisomers. Analytical HPLC of this material on a HP Agilent instrument using a Thermo Scientific HPLC column (Hypersil Gold AQ, 3.0 µm, 150 × 4.6 mm) and a 30-minute gradient run from 10–90% acetonitrile in a trifluoroacetic acid buffer indicated the major atropisomer was 87% of the material with a retention time of 19.3 minutes and the minor atropisomer was 12% of the material with a retention time of 19.8 minutes. The crude material was carried on in the next step without further purification. 1H NMR (400 MHz, DMSO) δ 10.28 (d, 1H), 9.93 (d, 2H), 9.76 (d, 1H), 8.96 – 8.88 (m, 4H), 8.55 (d, 2H), 8.08 (d, 2H), 7.88 (tt, 6H), 7.81 – 7.74 (m, 4H), 7.63 (dd, 3H), 7.53 – 7.39 (m, 8H), 7.36 – 7.07 (m, 19H), 7.06 – 6.95 (m, 3H), 5.71 (ddd, 3H), 5.51 – 5.29 (m, 7H), 4.12 (q 5H), 3.99 (q, 2H), 3.75 (d, 11H), 3.64 (dd, J = 13.8, 5.0 Hz, 2H), 3.49 (dd, J = 13.9, 8.1 Hz, 2H), 3.08 – 2.81 (m, 2H), 2.51 (p, 6H), 1.97 (s, 3H), 1.22 (s, 3H), 1.10 (td, , 7H), 1.06 (s, 0H), 0.84 (t, 3H). MS (ESI) *m/z* 817.2 (M+H)+. ^{1}H NMR, hydrogen-1 nuclear magnetic resonance; HPLC, high-performance liquid chromatography; MPLC, medium pressure liquid chromatography.

Ethyl (7R,20S)-18-chloro-1-(4-fluorophenyl)-10-{[2-(2-methoxyphenyl)pyrimidin-4-yl]methoxy}-19-methyl-15-[2-(4-methylpiperazin-1-yl)ethyl]-7,8,15,16-tetrahydro-14H-17,20-etheno-13,9-(metheno)-

6-oxa-2-thia-3,5,15-triazacyclooctadeca[1,2,3-cd]indene-7-carboxylate (S-22). A 250-mL round bottom flask equipped with a stir bar was charged with S-21 (1.96 g, 2.40 mmol) and anhydrous dichloromethane (160 mL) at room temperature under nitrogen. The mixture was cooled to 0°C in an ice bath, and 2-(4-methylpiperazin-1-yl)ethanamine (0.395 mL, 2.64 mmol) was added via a syringe. The mixture was stirred for 25 minutes at 0°C, and sodium triacetoxyborohydride (156 mg, 7.19 mmol) was added as a solid. The reaction mixture was stirred for 15 minutes at 0°C, and powdered activated 3-angstrom molecular sieves were added (1.96 g). The reaction mixture was stirred 2 hours at 0°C, and was allowed to stir and warm slowly to room temperature overnight. LC-MS indicated one major peak with a mass that corresponded to desired product. The reaction mixture was quenched with dichloromethane and water. The layers were separated, and aqueous layer was extracted with dichloromethane and 10% methanol/dichloromethane. The aqueous layer was neutralized with saturated aqueous $NaHCO_3$ mixture and was extracted one more time with 10% methanol/dichloromethane. The combined extracts were washed with saturated aqueous $NaHCO_3$ and brine, dried with Na_2SO_4, filtered, and concentrated. The residue was dissolved in dichloromethane and was purified on a Grace Reveleris X2 MPLC using a Teledyne Isco RediSep Rf gold 750-g silica gel column eluting with a gradient of 0–20% of methanol/dichloromethane over 40 minutes. The mixed fractions were purified on a Grace Reveleris X2 MPLC using a Teledyne Isco RediSep Rf gold 330-g silica gel column eluting with a ramp of 0–15% of methanol/dichloromethane over 40 minutes to collect additional title compound. The material from both columns was combined to provide the title compound (0.975 g, 44 %). ^{1}H NMR (501 MHz, dimethyl sulfoxide-*d*$_6$) δ ppm 8.61 (m, 2H), 7.47 (m, 2H), 7.39 (d, 1H), 7.17 (m, 7H), 7.04 (td, 1H), 6.96 (dd, 1H), 6.67 (d, 1H), 6.51 (d, 1H), 5.84 (dd, 1H), 5.06 (m, 2H), 4.07 (ddq, 2H), 3.90 (d, 1H), 3.75 (s, 3H), 3.68 (dd, 2H), 3.50 (d, 1H), 3.17 (m, 1H), 3.08 (m, 1H), 2.90 (m, 2H), 2.65-2.20 (m, 10H), 2.14 (s, 3H), 1.67 (s, 3H), 1.09 (t, 3H). MS (ESI) *m/z* 928.4 $(M+H)^+$. ^{1}H NMR, hydrogen-1 nuclear magnetic resonance; LC-MS, liquid chromatography-mass spectrometry; MPLC, medium pressure liquid chromatography.

(7R,20S)-18-chloro-1-(4-fluorophenyl)-10-{[2-(2-methoxyphenyl)pyrimidin-4-yl]methoxy}-19-methyl-15-[2-(4-methylpiperazin-1-yl)ethyl]-7,8,15,16-tetrahydro-14H-17,20-etheno-13,9-(metheno)-6-oxa-2-thia-3,5,15-triazacyclooctadeca[1,2,3-cd]indene-7-carboxylic acid (1). A 50-mL round bottom flask equipped with a stir bar was charged with S-22 (1.07 g, 1.15 mmol). The material was dissolved in tetrahydrofuran (5 mL). To the mixture at room temperature was added water (5.00 mL), solid LiOH (0.552 g, 23.1 mmol), and methanol (1 mL). The mixture was stirred overnight at room temperature. LC-MS indicated the reaction mixture was about 60% complete. Another 500 mg of LiOH was added along with another 1 mL of methanol and 2 mL of water. After 6 more hours at room temperature, LC-MS indicated one major peak with a mass that corresponded to desired product. The reaction mixture was diluted with water, and ethyl acetate was added. The cloudy, two-phase mixture was stirred for 10 minutes. The layers were separated. The aqueous layer had a pH of about 9 and was neutralized to pH 7 with saturated aqueous NH_4Cl mixture. The aqueous phase was extracted with ethyl acetate. The combined organic extracts were washed with saturated aqueous NH_4Cl mixture and brine, dried with

Na_2SO_4, filtered, and concentrated. The residue was dissolved in dichloromethane with about 2% methanol and purified on a Grace Reveleris X2 MPLC using a Teledyne Isco RediSep Rf gold 40-g silica gel column eluting with a gradient over 20 minutes of 10–40% methanol/dichloromethane, and then a gradient over 10 minutes of 40–60% methanol/dichloromethane. Most of the desired product eluted during the second gradient. The desired product-containing fractions were combined, and the solvents were concentrated to provide the title compound (0.915 g, 88 %). ^{1}H NMR (501 MHz, dimethyl sulfoxide-d_6) δ ppm 8.54 (m, 2H), 7.46 (m, 2H), 7.38 (d, 1H), 7.26 (d, 1H), 7.15 (m, 4H), 7.03 (m, 3H), 6.90 (dd, 1H), 6.59 (m, 2H), 5.87 (dd, 1H), 5.08 (d, 1H), 4.95 (d, 1H), 3.90-3.30 (m, 5H), 3.74 (s, 3H), 3.26 (dd, 1H), 3.03 (dd, 1H), 2.87 (m, 2H), 2.60-2.40 (m, 10H), 2.25 (s, 3H), 1.55 (s, 3H). MS (ESI) *m/z* 900.42 $(M+H)^+$. ^{1}H NMR, hydrogen-1 nuclear magnetic resonance; LC-MS, liquid chromatography-mass spectrometry; MPLC, medium pressure liquid chromatography.

Thieno[2,3-d]pyrimidin-4(3H)-one (S-23). A mixture of 2-amino-3-cyanothiophene (50 g, 403 mmol) in formic acid (100 mL, 2651 mmol) and H_2SO_4 (22 mL, 413 mmol) was heated in a sealed tube for 2 hours at 100°C. The mixture was cooled to 20°C and was diluted with water (1 L). The resulting precipitate was collected by filtration, washed with water twice (2 × 1 L) and dried under reduced pressure to provide the title compound (50 g, 329 mmol, 95%). ^{1}H NMR (400 MHz, dimethyl sulfoxide-d_6) δ ppm 12.16 (br s, 1H), 8.09 (s, 1H), 7.54 (d, 1H), 7.35 (d, 1H). MS ^{1}H NMR, hydrogen-1 nuclear magnetic resonance.

5,6-diiodothieno[2,3-d]pyrimidin-4(3H)-one (S-24). To an ice-cooled four-neck 2-L flask fit with a mechanical stirrer, reflux condenser, and thermocouple/J-KEM was added acetic acid (160 mL), sulfuric acid (8 mL) and water (80 mL) with stirring. S-23 (40.0 g, 263 mmol), periodic acid (30.0 g, 132 mmol), and iodine (133 g, 524 mmol) were added sequentially and the mixture became slightly endothermic. The ice bucket was removed and a heating mantle was added. The reaction mixture was ramped up to 60°C and was stirred for 20 minutes. The temperature climbed to 95°C. (THIS REACTION SELF-HEATS. EXOTHERMIC; USE CAUTION). The heating mantle was removed and reaction mixture was allowed to cool to room temperature. The resulting suspension was poured into saturated aqueous sodium sulfite solution, filtered, and washed with water. The organic layer was dried under vacuum to provide the title compound (67.7 g, 167 mmol, 63.7%). ^{1}H NMR (400MHz, DMSO-d_6) δ = 12.63 (br s, 1H), 8.15 (s, 1H). MS (ESI) *m/z* 404.8 $(M+H)^+$. ^{1}H NMR, hydrogen-1 nuclear magnetic resonance.

4-chloro-5,6-diiodothieno[2,3-d]pyrimidine (S-25). A 250-mL flask equipped with magnetic stirring, heating mantle, temperature probe, and reflux condenser to a nitrogen bubbler was charged with phosphorus oxychloride (57.3 mL, 615 mmol) and *N*,*N*-dimethylaniline (17.64 mL, 139 mmol). To the mixture was added S-24 (56.22 g, 139 mmol) over 5 minutes. The resulting suspension was heated at 105°C for 30 minutes. After cooling, the resulting material was broken up and transferred to a funnel with heptane. The material was washed with heptane to remove most of the phosphorus oxychloride. The material was slowly scooped into rapidly stirring ice water (600

mL) and stirred for 30 minutes. The material was collected by filtration, washed with water and ether (200 mL), dried over Na_2SO_4, and filtered to provide the title compound, which was used in the next step without further purification (56.2 g, 96%). ^1H NMR (400MHz, DMSO-d_6) δ = 8.90 (s, 1H). MS (DCI/NH3) *m/z* 422.6 $(M+H)^+$. ^1H NMR, hydrogen-1 nuclear magnetic resonance.

4-chloro-5-iodothieno[2,3-d]pyrimidine (S-26). A 500-mL three-neck jacketed flask with magnetic stirring under nitrogen was charged with S-25 (23 g, 77 mmol) and tetrahydrofuran (200 mL). The resulting suspension was cooled to –16°C using a Huber chiller set to –17°C. To the mixture was added *tert*-butylmagnesium chloride (40.8 mL, 2 M in ether) dropwise over 40 minutes, keeping the temperature between –15°C and –16°C. The temperature was slowly raised to 0°C and the mixture was stirred for 30 minutes. The reaction mixture was cooled to –20°C and was quenched by the very slow dropwise addition (initially about 1 drop/minute) of water (23 mL) over 35 minutes, maintaining the temperature at about –20°C, and then slowly warmed to ambient temperature over 1 hour. The stirring was stopped and the supernatant was decanted from the remaining residue. To the residue was added tetrahydrofuran (200 mL). The mixture was stirred briefly, and after standing, the supernatant was decanted from the remaining residue. This was repeated two times. The combined organics were concentrated. The crude material was purified by chromatography on silica gel eluting with isocratic methylene chloride. The title compound was precipitated from a minimum of hot heptanes to give the title compound (12.5 g, 4.2 mmol, 54.8%). ^1H NMR (400MHz, DMSO-d_6) δ = 8.96 (s, 1H), 8.46 (s, 1H). MS (ESI) *m/z* 296.6 $(M+H)^+$. ^1H NMR, hydrogen-1 nuclear magnetic resonance.

4-chloro-5-(4-methoxy-2,6-dimethylphenyl)thieno[2,3-d]pyrimidine (S-27). To a suspension of S-26 (5 g, 16.7 mmol), (4-methoxy-2,6-dimethylphenyl)boronic acid (6.07 g, 33.7 mmol) and cesium carbonate (10.99 g, 33.7 mmol) in degassed toluene (50.0 mL) and water (12.5 mL) was added bis(di-*tert*-butyl(4-dimethylaminophenyl)phosphine)dichloropalladium(II) (597 mg, 0.843 mmol). The mixture was heated to 100°C overnight. After cooling to room temperature, the mixture was diluted with ethyl acetate (200 mL). The organic layer was washed with water and brine, dried over anhydrous sodium sulfate, filtered, and concentrated under vacuum. The residue was purified by silica gel chromatography on a CombiFlash® Teledyne ISCO system eluting with 0–20% ethyl acetate in heptanes to provide the title compound (3.95 g, 12.2 mmol, 73 %). ^1H NMR (501 MHz, $CDCl_3$) δ ppm 8.88 (s, 1H), 7.35 (s, 1H), 6.70 (s, 2H), 3.85 (s, 3H), 1.99 (s, 6H). MS (ESI) *m/z* 305.1 $(M+H)^+$. ^1H NMR, hydrogen-1 nuclear magnetic resonance.

4-chloro-6-iodo-5-(4-methoxy-2,6-dimethylphenyl)thieno[2,3-d]pyrimidine (S-28). To a mixture of diisopropylamine (4.43 mL, 31.1 mmol) in tetrahydrofuran (50 mL) cooled to –78°C was added *n*-butyllithium (10.37 mL, 2.5 M in hexanes) dropwise. The mixture was stirred for 1 minute before S-27 (3.95 g, 12.96 mmol) was added as a mixture in tetrahydrofuran (50 mL). The resulting mixture was stirred at –78°C for 15 minutes. Iodine

(6.58 g, 25.9 mmol) was added in one portion and the mixture was warmed to room temperature. The reaction mixture was quenched with saturated aqueous ammonium chloride mixture (100 mL) and was extracted with ethyl acetate (50 mL × 3). The combined organic layers were washed sequentially with a sodium thiosulfate mixture and brine, dried over anhydrous sodium sulfate, filtered, and concentrated onto silica gel. Purification by flash chromatography on a silica gel column eluting with 0–20% ethyl acetate in heptanes provided crude product, which was triturated with heptanes to obtain the title compound (2.65 g, 6.22 mmol, 48 %). ^{1}H NMR (501 MHz, $CDCl_3$) δ ppm 8.82 (s, 1H), 6.72 (s, 2H), 3.87 (s, 3H), 1.94 (s, 6H). MS (ESI) *m/z* 431.1 $(M+H)^+$. ^{1}H NMR, hydrogen-1 nuclear magnetic resonance.

4-chloro-6-(4-fluorophenyl)-5-(4-methoxy-2,6-dimethylphenyl)thieno[2,3-d]pyrimidine (S-29). To a mixture of S-28 (3.3 g, 7.66 mmol), (4-fluorophenyl)boronic acid (2.144 g) di-*tert*-butyl(2',4',6'-triisopropyl-[1,1'-biphenyl]-2-yl)phosphine (0.179 g, 0.421 mmol) and potassium phosphate tribasic (3.25 g, 15.32 mmol) in degassed tetrahydrofuran (60 mL) and water (15 mL) was added tris(dibenzylideneacetone)dipalladium(0) (0.175 g, 0.192 mmol). The mixture was heated to 60°C overnight. After cooling to room temperature, the mixture was diluted with ethyl acetate (100 mL). The organic layer was washed with brine, dried over anhydrous sodium sulfate, filtered, and concentrated under vacuum. The residue was purified by flash chromatography on a silica gel column eluting with 0–20% ethyl acetate in heptanes to give crude product, which was triturated with heptanes to obtain the title compound (2.36 g, 5.89 mmol, 77 %). ^{1}H NMR (501 MHz, $CDCl_3$) δ ppm 8.84 (s, 1H), 7.31-7.23 (m, 2H), 7.02-6.93 (m, 2H), 6.65 (d, 2H), 3.83 (s, 3H), 1.92 (d, 6H). MS (ESI) *m/z* 399.1 $(M+H)^+$. ^{1}H NMR, hydrogen-1 nuclear magnetic resonance.

4-chloro-5-(3,5-dichloro-4-methoxy-2,6-dimethylphenyl)-6-(4-fluorophenyl)thieno[2,3-d]pyrimidine (S-30). To a suspension of S-29 (2.13 g, 5.34 mmol) in acetonitrile (50 mL) was added *N*-chlorosuccinimide (2.85 g, 21.36 mmol). The mixture was heated to reflux for 1 hour. The mixture was concentrated under vacuum and the residue was redissolved in ethyl acetate (50 mL). The mixture was washed with brine, dried over anhydrous sodium sulfate, filtered, and concentrated under vacuum. The residue was purified by silica gel chromatography on a CombiFlash Teledyne ISCO system eluting with 0–10% ethyl acetate in heptanes to provide the title compound (1.91 g, 4.05 mmol, 76 %). ^{1}H NMR (400 MHz, $CDCl_3$) δ ppm 8.89 (s, 1H), 7.28-7.18 (m, 2H), 7.08- 6.97 (m, 2H), 3.96 (s, 3H), 2.02 (s, 6H). MS (ESI) *m/z* 469.1 $(M+H)^+$. ^{1}H NMR, hydrogen-1 nuclear magnetic resonance.

2,6-dichloro-4-(4-chloro-6-(4-fluorophenyl)thieno[2,3-d]pyrimidin-5-yl)-3,5-dimethylphenol (S-31). To S-30 (5 g, 10.69 mmol) in 1,2-dichloroethane (200 mL) was added aluminum trichloride (4.28 g, 32.1 mmol), and the mixture was heated to 68°C for 6 hours and was cooled to room temperature. Saturated aqueous $NaHCO_3$ (3 mL) was added, and the mixture was stirred for 2 minutes. Saturated aqueous NH_4Cl (15 mL) was added. The mixture was diluted with ethyl acetate and the layers were separated. The aqueous layer was extracted once with

ethyl acetate. The organic layers were combined and washed with water and brine, dried over Na_2SO_4, filtered, and concentrated to provide the title compound (4.80 g, 10.6 mmol, 99%). ^1H NMR (400 MHz, dimethylsulfoxide-d_6) δ ppm 10.10 (br s, 1H), 9.00 (s, 1H), 7.35 (m, 2H), 7.28 (m, 2H), 1.96 (s, 6H). MS (ESI) *m/z* 452.9 $(M-H)^-$; ^{13}C NMR (400 MHz, DMSO-d_6) δ 168.19, 162.93 (d, *J* = 248.7 Hz), 154.22, 153.29, 149.70, 140.82, 135.26, 131.20 (d, *J* = 8.7 Hz), 128.56 (d, *J* = 3.2 Hz), 128.08, 127.79, 126.12, 120.70, 116.71 (d, *J* = 22.0 Hz), 18.43; HMRS (ESI/QTOF): $[M+H]^+$ calc for $C_{20}H_{13}Cl_3FN_2OS$ m/z 452.9793, found 452.9785 (1.7 ppm). ^1H NMR, hydrogen-1 nuclear magnetic resonance, 13C NMR, carbon-13 nuclear magnetic resonance, HRMS, high resolution mass spectrometry.

(R)-3-(allyloxy)propane-1,2-diol (S-32). To a 250-mL round bottom containing (*S*)-4-((allyloxy)methyl)-2,2-dimethyl-1,3-dioxolane (7.08 g, 41.1 mmol) was added methanol (100 mL) and *p*-toluenesulfonic acid monohydrate (0.782 g, 4.1 mmol). The mixture was heated to 50°C for 18 hours, and at 60°C for 4 hours. The mixture was cooled to room temperature, and potassium carbonate (1.704 g, 12.3 mmol) and $MgSO_4$ (5 g) were added. The material was filtered and washed with ethyl acetate. The mixture was concentrated, and the residue was chromatographed on silica gel using 20–80% ethyl acetate in heptanes as the eluent, to provide the title compound (4.25 g, 32.1 mmol, 78 %). ^1H NMR (400 MHz, dimethyl sulfoxide-d_6) δ ppm 5.87 (tdd, 1H), 5.25 (dd, 1H), 5.13 (dd, 1H), 4.62 (d, 1H), 4.46 (t, 1H), 3.94 (ddd, 2H), 3.58 (m, 1H), 3.39 (m, 1H), 3.30 (m, 3H). MS (ESI) *m/z* 133.0 $(M+H)^+$. ^1H NMR, hydrogen-1 nuclear magnetic resonance.

(S)-1-(allyloxy)-3-(bis(4-methoxyphenyl)(phenyl)methoxy)propan-2-ol (S-33). To a mixture of S-32 (2.25 g, 17.0 mmol) and 4,4'-(chloro(phenyl)methylene)bis(methoxybenzene) (DMTrCl) (6.06 g, 17.9 mmol) in dichloromethane (68.1 mL) cooled to 0°C, was added *N*,*N*-diisopropylethylamine (3.27 mL, 18.7 mmol). The mixture was allowed to warm to room temperature and was stirred for 30 minutes. The reaction mixture was quenched with saturated aqueous ammonium chloride mixture (50 mL). The organic layer was washed with brine, dried over anhydrous sodium sulfate, filtered, and concentrated under vacuum. The residue was purified by silica gel chromatography on a CombiFlash Teledyne ISCO system, eluting with 0–50% ethyl acetate in heptanes to provide the title compound (6.2 g, 14.3 mmol, 84%). ^1H NMR (400 MHz, $CDCl_3$) δ ppm 7.45-7.40 (m, 2H), 7.35-7.24 (m, 6H), 7.24-7.17 (m, 1H), 6.86-6.77 (m, 4H), 5.95-5.79 (m, 1H), 5.24 (dq, 1H), 5.17 (dq, 1H), 4.00 (dt, 2H), 3.98-3.91 (m, 1H), 3.78 (s, 6H), 3.55 (dd, 1H), 3.49 (dd, 1H), 3.24-3.16 (m, 2H), 2.40 (bs, 1H). MS (ESI) *m/z* 457.1 $(M+Na)^+$; ^{13}C NMR (101 MHz, $CDCL_3$) δ 158.49, 144.87, 136.04, 134.57, 130.06, 128.15, 127.81, 126.78, 117.11, 113.12, 112.89, 86.10, 77.38, 77.06, 76.74, 72.29, 71.62, 69.97, 64.44, 55.21; HRMS (ESI-QTOF+) $[M+Na]^+$ calc. for $C_{27}H_{30}NaO_5$ m/z 457.1985, found 457.1975 (2.3 ppm); $[\alpha]^{20}_D$ -3.0 (*c* 1.0, $CHCl_3$). ^1H NMR, hydrogen-1 nuclear magnetic resonance, 13C NMR, carbon-13 nuclear magnetic resonance, HRMS, high resolution mass spectrometry.

(R)-5-(4-((1-(allyloxy)-3-(bis(4-methoxyphenyl)(phenyl)methoxy)propan-2-yl)oxy)-3,5-dichloro-2,6-dimethylphenyl)-4-chloro-6-(4-fluorophenyl)thieno[2,3-d]pyrimidine (S-34). Triphenylphosphine (3.47 g,

13.22 mmol), S-31 (3.0 g, 6.61 mmol), and S-33 (4.31 g, 9.92 mmol) were taken up in 18 mL tetrahydrofuran and di-*tert*-butylazodicarboxylate (3.05 g, 13.22 mmol) was added and the reaction was stirred overnight. The material was filtered off and rinsed with 1:1 ether/ethyl acetate, and the organics were concentrated. The crude material was chromatographed on silica gel using 1–40% ethyl acetate in heptanes as eluent to provide the title compound (6.1 g, 6.61 mmol, ~100%). ^{1}H NMR (400 MHz, DMSO-d_6) δ 9.00 (s, 1H), 7.44 – 7.35 (m, 2H), 7.35 – 7.16 (m, 9H), 7.10 (t, *J* = 8.8 Hz, 2H), 6.90 – 6.79 (m, 4H), 5.74 (ddt, *J* = 17.3, 10.5, 5.3 Hz, 1H), 5.20 – 4.99 (m, 2H), 4.56 (t, *J* = 5.0 Hz, 1H), 3.89 – 3.82 (m, 2H), 3.72 (s, 6H), 3.70 (t, *J* = 4.5 Hz, 2H), 3.41 (d, *J* = 5.4 Hz, 2H), 1.98 (d, *J* = 14.2 Hz, 6H); MS (ESI) *m/z* 891.1 $(M+Na)^+$; ^{13}C NMR (101 MHz, Chloroform-*d*) δ 168.33, 163.13 (d, *J* = 251.7 Hz), 158.49, 154.84, 152.71, 151.33, 144.92, 140.88, 136.15, 136.07, 135.49, 134.62, 130.55, 130.46, 130.28, 130.10 (d, *J* = 3.6 Hz), 129.14, 128.33, 128.20, 127.76, 127.65, 126.75, 116.73, 116.29 (d, *J* = 21.9 Hz), 113.18, 113.08, 112.88, 86.39, 81.30, 72.32, 70.76, 62.96, 55.25, 55.21, 18.41, 18.35; HRMS (ESI QTOF+) $[M+H]^+$ calc for $C_{47}H_{41}Cl_3FN_2O_5S$ 869.1780, found 869.1769 (1.3 ppm); $[\alpha]^{20}_D$ +15.0 (*c* 1.0, $CHCl_3$).^{1}H NMR, hydrogen-1 nuclear magnetic resonance, ^{13}C NMR, carbon-13 nuclear magnetic resonance, HRMS, high resolution mass spectrometry

2-(benzyloxy)-5-((tert-butyldimethylsilyl)oxy)benzaldehyde (S-35). A 2-L round bottom flask was charged with 2,5-dihydroxybenzaldehyde (30 g, 217 mmol), imidazole (29.6 g, 434 mmol), and dichloromethane (543 mL). The flask was placed in a water bath and solid *tert*-butylchlorodimethylsilane (32.7 g, 217 mmol) was added. The reaction mixture was stirred at ambient temperature for 15 minutes, at which point thin-layer chromatography indicated complete consumption of starting material. The reaction mixture was poured into a separatory funnel with 200 mL water. The biphasic mixture was shaken, and the layers were separated. The aqueous layer was washed with 100 mL dichloromethane and the organic layers were combined. The organic layer was dried over sodium sulfate, filtered, and concentrated and the material was used in the next step. A 1-L three-necked round bottom flask equipped with an internal temperature probe, a reflux condenser, and a stir bar was charged with 5-((*tert*-butyldimethylsilyl)oxy)-2-hydroxybenzaldehyde (45 g, 178 mmol) in acetone (297 mL). Solid K_2CO_3 (27.1 g, 196 mmol) was added followed by dropwise addition of neat benzyl bromide (21.21 mL). The mixture was stirred at ambient temperature for 10 minutes and heated to 55°C. The reaction mixture was stirred overnight. The reaction mixture was cooled to ambient temperature then poured over cold water (200 mL). The mixture was then transferred to a 1-L separatory funnel. The crude product was extracted with ethyl acetate (3 × 250 mL). The combined organic layers were dried over sodium sulfate, filtered, and concentrated. The crude material was purified by silica gel chromatography over a 330-g column on a Grace Reveleris system (0–5% ethyl acetate/heptanes elution gradient). Fractions containing the desired product were combined, concentrated, and dried under vacuum to obtain the title compound (45 g, 160.6 mmol, 74%). ^{1}H NMR (501 MHz, dimethyl sulfoxide-d_6) δ ppm 10.35 (s, 1H), 7.51-7.47 (m, 2H), 7.42-7.37 (m, 2H), 7.35-7.31 (m, 1H), 7.22 (d, 1H), 7.15 (dd, 1H), 7.11 (d, 1H), 5.21 (s, 2H), 0.93 (s, 9H), 0.16 (s, 6H). MS (ESI) *m/z* 343.21 $(M+H)^+$. ^{1}H NMR, hydrogen-1 nuclear magnetic resonance.

Tert-butyl 2-acetoxy-2-(diethoxyphosphoryl)acetate (S-36). A 3-L jacketed round bottom flask equipped with an overhead stirrer was charged with glyoxylic acid monohydrate (15 g, 163 mmol) and diethyl phosphite (20.82 mL, 161 mmol) and was heated to a 60°C jacket temperature with stirring. The flask headspace was continuously purged with a nitrogen sweep. After stirring overnight, dichloromethane (250 mL) was added, the reaction was cooled to an internal temperature of 5°C, and pyridine (13.05 mL, 161 mmol) was added dropwise. After stirring for 1 hour at the same temperature, acetyl chloride (11.47 mL, 161 mmol) was added dropwise over 20 minutes. The reaction was warmed to 20°C, stirred for 1.5 hours, and cooled to 5°C internal temperature. Pyridine (19.57 mL, 242 mmol) was added slowly. *Tert*-butanol (15.43 mL, 203 mmol) was added in one portion followed by dropwise addition of 2,4,6-tripropyl-1,3,5,2,4,6-trioxatriphosphinane 2,4,6-trioxide (144 mL, 242 mmol, 50% by weight in ethyl acetate) over 20 minutes. After stirring for 1 hour, the reaction was warmed to 20°C and was stirred overnight. The reactor was then cooled to 5°C and 1N aqueous hydrochloric acid (200 mL) was added slowly. The biphasic mixture was stirred for 30 minutes at 20°C, and was poured into a separatory funnel. Dichloromethane (400 mL) and 1N aqueous hydrochloric acid (250 mL) were added and the mixture was separated. The aqueous layer was extracted with dichloromethane (400 mL), and the combined organic layers were washed with a mixture of water (300 mL) and saturated aqueous sodium chloride solution (300 mL). The combined organics were dried over anhydrous magnesium sulfate, filtered, and concentrated under reduced pressure. The crude material was purified by plug filtration on silica gel eluting with 1:1 ethyl acetate/heptanes to give the title compound after concentration under reduced pressure. Semi-pure material was used in next step without further purification. ^{1}H NMR (400 MHz, Chloroform-*d*) δ ppm 5.32 (d, 1H), 4.29-4.18 (m, 4H), 2.21 (s, 3H), 1.37 (tdd, 6H). MS (ESI) *m/z* 255.0 (M–*tert*-butyl+2H)$^+$. ^{1}H NMR, hydrogen-1 nuclear magnetic resonance.

(E)-tert-butyl 2-acetoxy-3-(2-(benzyloxy)-5-((tert-butyldimethylsilyl)oxy)phenyl)acrylate (S-37). An oven dried 2-L three-neck round bottomed flask equipped with overhead stirring was charged with anhydrous lithium chloride (5.55 g, 131 mmol). The flask was purged with a sweep of argon for 10 minutes and anhydrous tetrahydrofuran (350 mL) was added. A solution of S-36 (40.6 g, 131 mmol) in tetrahydrofuran (50 mL) was added. A solution of 1,8-diazabicyclo[5.4.0]undec-7-ene) (19.72 mL, 131 mmol) in tetrahydrofuran (50 mL) was added dropwise. The stirring mixture became cloudy and was cooled in an ice-water bath to an internal temperature of 15°C. A mixture of S-35 (32 g, 93 mmol) in tetrahydrofuran (50 mL) was added over 30 minutes. The reaction was stirred overnight, cooled to an internal temperature of 5°C, and quenched by addition of 1% by weight aqueous citric acid (700 mL). Ethyl acetate (400 mL) was added and the layers were separated. The combined organic layers were washed with saturated aqueous sodium chloride solution (400 mL), dried over anhydrous magnesium sulfate, filtered, and concentrated under reduced pressure. The crude material was purified by flash column chromatography on a Grace Reveleris system using a Teledyne Isco RediSep Gold 330-g column, eluting with a 0–25% ethyl acetate/heptanes gradient to give the title compound in a 9:1 mixture of *E*-and *Z*- isomers (31.2 g, 67%).

E-isomer ^{1}H NMR (501 MHz, Chloroform-*d*) δ ppm 7.39 (ddt, 2H), 7.36 (ddd, 2H), 7.32-7.27 (m, 1H), 6.88 (dd, 1H), 6.85 (d, 1H), 6.76 (d, 1H), 6.71 (ddd, 1H), 5.01 (s, 2H), 2.22 (s, 3H), 1.34 (s, 9H), 0.97 (s, 9H), 0.17 (s, 6H). MS (ESI) *m/z* 515.9 $(M+NH_4)^+$; ^{13}C NMR (101 MHz, CDCL$_3$) δ 169.35, 168.56, 161.05, 151.82, 150.81, 149.26, 148.92, 139.59, 138.07, 137.19, 137.00, 128.51, 128.47, 127.86, 127.77, 127.34, 127.08, 123.63, 123.60, 122.40, 122.32, 121.04, 120.41, 120.21, 113.78, 113.54, 82.18, 82.13, 77.38, 77.06, 76.74, 71.26, 71.21, 31.90, 28.02, 27.64, 25.68, 22.70, 20.66, 20.49, 18.15, 18.11, 14.13, -4.21, -4.45, -4.49, -4.77; HRMS (ESI-QTOF+) $[M+NH_4]^+$ calc. for $C_{28}H_{42}NO_6Si$ m/z 516.2776 found 516.2770 (1.21 ppm) This isomer was assigned *E* by 2D NOE experiments. *Z*-isomer: ^{1}H NMR (501 MHz, Chloroform-*d*) δ ppm 7.74 (s, 1H), 7.45 (ddt, 2H), 7.38 (ddd, 2H), 7.35-7.30 (m, 1H), 7.29-7.26 (m, 1H), 6.83 (d, 1H), 6.79 (dd, 1H), 5.06 (s, 2H), 2.30 (d, 3H), 1.53 (s, 9H), 0.99 (s, 9H), 0.18 (s, 6H). MS (ESI) *m/z* 515.9 $(M+NH_4)^+$. This isomer was assigned *Z* by 2D NMR experiments. ^{1}H NMR, hydrogen-1 nuclear magnetic resonance, ^{13}C NMR, carbon-13 nuclear magnetic resonance, HRMS, high resolution mass spectrometry.

(R)-tert-butyl 2-acetoxy-3-(2-(benzyloxy)-5-((tert-butyldimethylsilyl)oxy)phenyl)propanoate (S-38). A 600-mL stainless steel reactor was charged with (1,2-bis[(2*R*,5*R*)-2,5-diethylphospholano]benzene(1,5-cyclooctadiene)rhodium(I) trifluoromethanesulfonate (1.88 g, 2.6 mmol), followed by a solution of S-37 (34.86 g, 69.9 mmol) in methanol (350 mL). The reactor was purged with nitrogen three times and two times with hydrogen. The mixture was stirred at 1200 RPM under 120 psi of hydrogen with no external heating for 24 hours. The mixture was concentrated under reduced pressure, suspended in 5:1 heptanes/dichloromethane (70 mL), and filtered through a pad of diatomaceous earth. The filtrate was concentrated under reduced pressure and purified on a Grace Reveleris system using a 750-g Teledyne Isco Redisep gold column eluting with an ethyl acetate/heptanes gradient (0–25%). The title compound was concentrated under reduced pressure (31.5 g, 2.6 mmol, 90%). ^{1}H NMR (500 MHz, Chloroform-*d*) δ ppm 7.45 (d, 2H), 7.42-7.34 (m, 2H), 7.34-7.28 (m, 1H), 6.77 (d, 1H), 6.70 (d, 1H), 6.67 (dd, 1H), 5.19 (dd, 1H), 5.05 (d, 1H), 5.01 (d, 1H), 3.29 (dd, 1H), 2.92 (dd, 1H), 2.03 (s, 3H), 1.40 (s, 9H), 0.97 (s, 9H), 0.16 (s, 6H). MS (DCI) *m/z* 518.2 $(M+NH_4)^+$; ^{13}C NMR (101 MHz, CDCL$_3$) δ 170.41, 169.30, 151.32, 149.05, 137.37, 128.48, 127.70, 127.09, 125.93, 123.21, 118.78, 112.45, 81.93, 77.35, 77.03, 76.71, 72.31, 70.45, 32.48, 31.88, 29.02, 27.91, 25.71, 22.69, 20.66, 18.17, 14.11, -4.45, -4.47, -4.73; HRMS (ESI-QTOF+) $[M+Na]^+$ calc. for $C_{28}H_{38}NaO_6Si$ m/z 521.2330, found 521.2323 (1.3 ppm); $[\alpha]^{20}_D$ –0.4 (*c* 0.8, CHCl$_3$). ^{1}H NMR, hydrogen-1 nuclear magnetic resonance, ^{13}C NMR, carbon-13 nuclear magnetic resonance, HRMS, high resolution mass spectrometry.

(R)-tert-butyl 3-(2-(benzyloxy)-5-((tert-butyldimethylsilyl)oxy)phenyl)-2-hydroxypropanoate (S-39). An oven-dried, 250-mL three-neck flask was charged with S-38 (27.46 g, 54.8 mmol). The flask was equipped with a magnetic star bar and rubber septa, and vacuum purged with nitrogen gas twice. Anhydrous ethanol (274 mL) was added, and the mixture was stirred. To the stirring solution was added dropwise sodium ethoxide (21% wt in ethanol, 1.024 mL, 2.74 mmol). The reaction was stirred for 3 hours at ambient temperature and was quenched by addition of acetic acid (0.3 mL). The bulk of the solvents were removed by rotary evaporation, and the material

was diluted with ethyl acetate (300 mL). Saturated aqueous sodium bicarbonate was added (300 mL). The layers were separated, and the aqueous layer was extracted with ethyl acetate (300 mL). The combined organic layers were washed with saturated aqueous sodium chloride, dried over $MgSO_4$, treated with activated charcoal (0.5 g), and stirred for 1 hour before filtering through diatomaceous earth to give the title compound after concentration under reduced pressure (24.0 g, 2.6 mmol, 96%). ^{1}H NMR (400 MHz, chloroform-*d*) δ ppm 7.48-7.42 (m, 2H), 7.42-7.36 (m, 2H), 7.36-7.29 (m, 1H), 6.79 (d, 1H), 6.75 (d, 1H), 6.67 (dd, 1H), 5.10-4.99 (m, 2fH), 4.39 (ddd, 1H), 3.16 (dd, 1H), 2.91 (d, 1H), 2.86 (dd, 1H), 1.41 (s, 9H), 0.99 (s, 9H), 0.18 (s, 6H). MS (DCI) *m/z* 476.2 $(M+NH_4)^+$; ^{13}C NMR (101 MHz, DMSO) δ 173.51, 151.33, 148.71, 137.97, 128.75, 127.99, 127.56, 127.49, 123.33, 118.17, 113.06, 80.23, 70.53, 70.11, 40.60, 40.39, 40.18, 39.97, 39.76, 39.55, 39.34, 35.75, 28.02, 26.01, 18.26, -4.14, -4.20. HRMS (ESI-QTOF+) $[M+Na]^+$ calc. for $C_{26}H_{38}NaO_5Si$ m/z 481.2381, found 481.2369 (2.52 ppm). ^{1}H NMR, hydrogen-1 nuclear magnetic resonance, ^{13}C NMR, carbon-13 nuclear magnetic resonance, HRMS, high resolution mass spectrometry.

Tert-butyl (R)-2-((5-(4-(((R)-1-(allyloxy)-3-(bis(4-methoxyphenyl)(phenyl)methoxy)propan-2-yl)oxy)-3,5-dichloro-2,6-dimethylphenyl)-6-(4-fluorophenyl)thieno[2,3-d]pyrimidin-4-yl)oxy)-3-(2-(benzyloxy)-5-((tert-butyldimethylsilyl)oxy)phenyl)propanoate (S-40). S-34 (14.7 g, 16.9 mmol), S-39 (8.52 g, 18.6 mmol), and cesium carbonate (11.01 g, 16.9 mmol) were added to a three-necked flask equipped with an overhead stirrer and 2.2 g of 4-mm glass beads. *Tert*-butanol (145 mL) was added, and the mixture was heated to 65°C for 3 hours. Additional cesium carbonate (5.50 g, 16.9 mmol) was added, and the reaction was stirred at 65°C overnight. The reaction mixture was cooled and was diluted with ethyl acetate (300 mL). The resulting solution was filtered through diatomaceous earth and washed through with 200 mL ethyl acetate. The mixture was concentrated, taken up in toluene, and purified by silica gel chromatography using 10–30% ethyl acetate in heptanes as eluent to give the title compound (17.1 g, 78%). ^{1}H NMR (500 MHz, dimethylsulfoxide-d_6) δ ppm 8.63 (s, 1H), 7.33 (dd, 4H), 7.32-7.11 (m, 13H), 7.06-6.98 (m, 2H), 6.85 (d, 1H), 6.81-6.74 (m, 4H), 6.63 (dd, 1H), 6.40 (d, 1H), 5.57 (m, 1H), 5.27 (t, 1H), 4.98-4.88 (m, 4H), 4.42 (m, 1H), 3.72-3.63 (m, 10H), 3.55 (m, 2H), 2.60 (dd, 1H), 2.08 (s, 3H), 1.81 (s, 3H), 1.13 (s, 9H), 0.83 (S, 9H), 0.23 (d, 6H). MS (ESI) *m/z* 1293.3 $(M+H)^+$; ^{13}C NMR (101 MHz, DMSO) δ 168.34, 167.02, 162.65, 162.63 (d, J = 248.9 Hz), 158.51, 153.45, 151.35, 150.28, 148.78, 145.25, 137.61, 137.11, 136.11, 135.95, 134.98, 134.68, 132.02, 130.73 (d, J = 8.6 Hz), 130.07, 129.37, 128.95 (d, J = 3.4 Hz), 128.61, 128.07, 127.95, 127.48, 127.37, 127.01, 126.78, 126.60, 124.89, 122.34, 118.80, 116.54 (d, J = 21.9 Hz), 116.36, 113.46, 113.17, 112.96, 86.11, 81.52, 81.28, 73.72, 71.66, 70.08, 69.53, 63.15, 55.33, 32.45, 31.68, 27.67, 25.85, 22.52, 18.58, 18.27, 18.14, 14.31, -4.28, -4.36; HRMS (ESI-QTOF+) $[M+H]^+$ calc. for $C_{73}H_{78}Cl_2FN_2O_{10}S$ 1291.4502, found 1291.4506 (-0.28 ppm); $[\alpha]^{20}_D$ -23.0 (*c* 1.0, $CHCl_3$). ^{1}H NMR, hydrogen-1 nuclear magnetic resonance, ^{13}C NMR, carbon-13 nuclear magnetic resonance, HRMS, high resolution mass spectrometry.

Tert-butyl (R)-2-((5-(4-(((S)-1-(allyloxy)-3-hydroxypropan-2-yl)oxy)-3,5-dichloro-2,6-dimethylphenyl)-6-(4-fluorophenyl)thieno[2,3-d]pyrimidin-4-yl)oxy)-3-(2-(benzyloxy)-5-((tert-

butyldimethylsilyl)oxy)phenyl)propanoate (S-41). S-40 (17.11 g, 13.2 mmol) in dichloromethane (65 mL) and methanol (65 mL) was cooled to 0°C. Formic acid (38 mL, 991 mmol) was added, and the solution was stirred for 15 minutes at 0°C. The mixture was slowly added to 1 L of vigorously stirred saturated aqueous sodium bicarbonate. The resulting mixture was extracted with ethyl acetate (2 × 500 mL). The combined organics were washed with brine (100 mL), dried over Na_2SO_4, filtered, and concentrated. The crude material was purified by silica gel chromatography using 10–30% ethyl acetate in heptanes as eluent to give the title compound (13.0 g, 99%). ^{1}H NMR (500 MHz, dimethylsulfoxide-d_6) δ ppm 8.62 (s, 1H), 7.38 (d, 2H), 7.32-7.24 (m, 6H), 7.19 (t, 2H), 6.88 (d, 1H), 6.65 (dd, 1H), 6.43 (d, 1H), 5.66 (m, 1H), 5.32 (t, 1H), 5.05 (dq, 1H), 4.99 (s, 2H), 4.96 (dq, 1H), 4.80 (t, 1H), 4.37 (m, 1H), 3.77 (m, 2H), 3.68-3.61 (m, 3H), 3.58 (dd, 1H), 2.65 (dd, 1H), 2.07 (s, 3H), 1.81 (s, 3H), 1.16 (s, 9H), 0.88 (s, 9H), 0.07 (d, 6H). MS (ESI) *m/z* 988.9 $(M+H)^+$; ^{13}C NMR (101 MHz, DMSO-d_6) δ 168.39, 162.71, 162.68 (d, *J* = 248.0 Hz), 153.49, 151.33, 150.42, 148.76, 137.66, 135.86, 135.29, 134.59, 131.83, 130.81 (d, *J* = 8.6 Hz), 129.01 (d, *J* = 3.3 Hz), 128.65, 128.01, 127.57, 127.49, 126.55, 125.02, 122.48, 118.75, 118.73, 116.73, 116.46 (d, *J* = 10.6 Hz), 113.00, 83.13, 81.58, 73.75, 71.76, 70.06, 69.79, 60.63, 32.51, 27.70, 25.92, 18.58, 18.24, 18.21, -4.21, -4.29; HRMS (ESI-QTOF+) calc. for $C_{52}H_{60}Cl_2FN_2O_8SSi$ m/z 989.3195, found 989.3201 (-0.55 ppm); $[\alpha]^{20}_{D}$ -42.0 (*c* 1.0, $CHCl_3$). ^{1}H NMR, hydrogen-1 nuclear magnetic resonance, ^{13}C NMR, carbon-13 nuclear magnetic resonance, HRMS, high resolution mass spectrometry.

(R)-tert-butyl 2-((5-(4-(((R)-1-(allyloxy)-3-(tosyloxy)propan-2-yl)oxy)-3,5-dichloro-2,6-dimethylphenyl)-6-(4-fluorophenyl)thieno[2,3-d]pyrimidin-4-yl)oxy)-3-(2-(benzyloxy)-5-((tert-butyldimethylsilyl)oxy)phenyl)propanoate (S-42). S-41 (13.04 g, 13.2 mmol) was dissolved in dichloromethane (125 mL) and cooled to 0°C. *p*-Toluenesulfonyl chloride (3.77 g, 19.8 mmol), and 1,4-diazabicyclo[2.2.2]octane (2.95 g, 36.3 mmol) were added, and the reaction was stirred at 0°C for 30 minutes. The mixture was diluted with 55 mL dichloromethane, and quenched with 55 mL saturated aqueous NH_4Cl. The layers were separated and the organic layer was washed with brine, dried over Na_2SO_4, filtered, and concentrated. The crude material was purified by silica gel chromatography using 10–25% ethyl acetate in heptanes to provide the title compound (14.2 g, 94%). ^{1}H NMR (500 MHz, dimethylsulfoxide-d_6) δ ppm 8.61 (s, 1H), 7.68 (d, 2H), 7.37 (d, 2H), 7.34 (dd, 2H), 7.31-7.22 (m, 6H), 7.18 (t, 2H), 6.86 (d, 1H), 6.63 (dd, 1H), 6.39 (d, 1H), 5.57 (m, 1H), 5.28 (t, 1H), 5.01-4.92 (m, 3H), 4.45 (m, 1H), 4.24 (m, 2H), 3.67 (d, 2H), 3.50 (d, 2H), 2.62 (dd, 1H), 2.43 (dd, 1H), 2.34 (s, 3H), 2.03 (s, 3H), 1.78 (s, 3H), 1.15 (s, 9H), 0.85 (s, 9H), 0.04 (d, 6H). MS (ESI) *m/z* 1145.1 $(M+H)^+$; ^{13}C NMR (126 MHz, DMSO-d_6) δ 168.41, 167.07, 162.75 (d, *J* = 248.3 Hz), 162.69, 153.56, 151.33, 149.47, 148.84, 145.49, 137.69, 137.26, 136.15, 135.00, 134.82, 132.66, 132.50, 130.86 (d, *J* = 8.5 Hz), 130.53, 128.99 (d, *J* = 3.3 Hz), 128.69, 128.09, 128.06, 127.61, 127.26, 126.51, 126.37, 125.01, 122.25, 118.77, 118.73, 116.92, 116.80, 116.63, 113.12, 81.68, 79.21, 73.77, 71.81, 70.18, 69.54, 67.91, 32.33, 27.77, 25.95, 21.51, 18.56, 18.26, 18.24, -4.19, -4.26; HRMS (ESI-QTOF+) $[M+H]^+$ calc. for $C_{59}H_{66}Cl_2FN_2O_{10}S_2$ m/z 143.3284, found 143.3283 (0.1 ppm); $[\alpha]^{20}_{D}$ -26.0 (*c* 1.0, $CHCl_3$). ^{1}H NMR, hydrogen-1

nuclear magnetic resonance, ^{13}C NMR, carbon-13 nuclear magnetic resonance, HRMS, high resolution mass spectrometry.

(R)-tert-butyl 2-((5-(4-(((R)-1-(allyloxy)-3-(tosyloxy)propan-2-yl)oxy)-3,5-dichloro-2,6-dimethylphenyl)-6-(4-fluorophenyl)thieno[2,3-d]pyrimidin-4-yl)oxy)-3-(2-(benzyloxy)-5-hydroxyphenyl)propanoate (S-43). To S-42 (14.15 g, 12.4 mmol) in tetrahydrofuran (120 mL) was added acetic acid (0.779 mL, 13.6 mmol), and tetrabutylammonium fluoride (13.60 mL, 13.6 mmol, 1M in tetrahydrofuran). The reaction mixture was stirred for 20 minutes. The mixture was quenched with 20 mL saturated aqueous sodium bicarbonate solution. The mixture was diluted with 20% ethyl acetate/heptanes (150 mL). The layers were separated, and the organic layer was washed with water and brine, dried over Na_2SO_4, filtered, and concentrated. The crude material was purified by silica gel chromatography using 10–50% ethyl acetate in heptanes to provide the title compound (11.9 g, 93%). ^{1}H NMR (400 MHz, dimethylsulfoxide-*d*$_6$) δ ppm 8.90 (s, 1H), 8.64 (s, 1H), 7.70 (d, 2H), 7.40 (d, 2H), 7.30 (m, 7H), 7.21 (m, 2H), 7.05 (t, 1H), 6.81 (d, 1H), 6.57 (m, 1H), 6.17 (d, 1H), 5.65 (m, 1H), 5.20 (t, 1H), 5.00 (m, 2H), 4.50 (m, 1H), 4.25 (m, 2H), 3.72 (m, 2H), 3.56 (m, 2H), 2.66 (m, 1H), 2.39 (s, 3H), 2.14 (s, 3H), 1.82 (s, 3H), 1.21 (s, 9H). MS (ESI) *m/z* 1030.7 $(M+H)^+$; ^{13}C NMR (101 MHz, DMSO-*d*$_6$) δ 168.60, 167.02, 162.69 (d, *J* = 248.3 Hz), 162.64, 153.49, 151.35, 149.43, 145.48, 137.89, 137.33, 136.23, 134.82, 132.55, 132.35, 130.87 (d, *J* = 8.5 Hz), 130.49, 128.98 (d, *J* = 3.1 Hz), 128.64, 128.03 (d, *J* = 9.4 Hz), 127.53, 127.22, 124.99, 118.70, 117.72, 116.93, 116.76, 116.54, 114.27, 113.48, 81.74, 79.13, 74.10, 71.73, 70.28, 69.42, 67.76, 32.38, 31.67, 27.76, 22.52, 21.47, 18.63, 18.21, 14.34; HRMS (ESI-QTOF+) $[M+H]^+$ calc. for $C_{53}H_{51}Cl_2FN_2NaO_{10}$ m/z 1051.2238, found 1051.2233 (0.55 ppm); $[\alpha]^{20}_D$ +24.0 (*c* 1.0, $CHCl_3$). ^{1}H NMR, hydrogen-1 nuclear magnetic resonance, ^{13}C NMR, carbon-13 nuclear magnetic resonance, HRMS, high resolution mass spectrometry.

Tert-butyl (7R,16R)-10-(benzyloxy)-19,23-dichloro-1-(4-fluorophenyl)-20,22-dimethyl-16-{[(prop-2-en-1-yl)oxy]methyl}-7,8,15,16-tetrahydro-18,21-etheno-13,9-(metheno)-6,14,17-trioxa-2-thia-3,5-diazacyclononadeca[1,2,3-cd]indene-7-carboxylate (S-44). To S-43 (11.88 g, 11.5 mmol) in *N*,*N*-dimethylformamide (1160 mL) was added cesium carbonate (18.79 g, 57.7 mmol) and the reaction was stirred for 2 hours. The solution was poured into water (3600 mL), and the aqueous solution was extracted with ethyl acetate (4 × 300 mL). The combined organics were washed with water (2 × 800 mL), and brine (500 mL), dried over Na_2SO_4, filtered, and concentrated. The crude material was purified by silica gel chromatography using 10–50% ethyl acetate in heptanes to provide the title compound (8.75 g, 88%). ^{1}H NMR (500 MHz, dimethylsulfoxide-*d*$_6$) δ ppm 8.75 (s, 1H), 7.40 (m, 5H), 7.20 (m, 4H), 6.90 (m, 2H), 5.98 (m, 1H), 5.92 (m, 1H), 5.68 (s, 1H), 5.30 (d, 1H), 5.19 (d, 1H), 5.02 (q, 2H), 4.81 (m, 1H), 4.51 (dd, 1H), 4.36 (d, 1H), 4.03 (m, 2H), 3.75 (m, 2H), 3.58 (m, 1H), 2.81 (m, 1H), 2.05 (s, 3H), 1.91 (s, 3H), 1.09 (s, 9H). MS (ESI) *m/z* 857.0 $(M+H)^+$; ^{13}C NMR (126 MHz, DMSO-*d*$_6$) δ 167.94, 167.41, 163.38, 162.67 (d, *J* = 248.1 Hz), 153.27, 151.49, 150.49 (d, *J* = 11.6 Hz), 138.15, 137.73, 135.69, 135.32, 134.90, 131.70, 131.14 (d, *J* = 8.6 Hz), 129.06 (d, *J* = 3.2 Hz), 128.83, 128.45, 128.16, 127.70, 127.46, 125.95 (d, *J* = 9.6 Hz),

121.26, 118.44, 117.13, 116.61, 116.44, 116.02, 113.30, 81.60, 77.66, 73.95, 72.72, 72.03, 70.37, 69.68, 30.08, 27.64, 18.81, 18.64; HRMS (ESI-QTOF+) $[M+H]^+$ calc. for $C_{46}H_{44}Cl_2FN_2O_7S$ 857.2225, found 857.2218 (0.77ppm); $[\alpha]^{20}_{D}$ -82.0 (*c* 1.0, $CHCl_3$ ^{1}H NMR, hydrogen-1 nuclear magnetic resonance, ^{13}C NMR, carbon-13 nuclear magnetic resonance, HRMS, high resolution mass spectrometry.

Tert-butyl (7R,16R)-10-(benzyloxy)-19,23-dichloro-1-(4-fluorophenyl)-16-(hydroxymethyl)-20,22-dimethyl-7,8,15,16-tetrahydro-18,21-etheno-13,9-(metheno)-6,14,17-trioxa-2-thia-3,5-diazacyclononadeca[1,2,3-cd]indene-7-carboxylate (S-45). A solution of S-44 (8.75 g, 10.2 mmol) in tetrahydrofuran (120 mL) and methanol (80 mL) was degassed and flushed with nitrogen three times. Tetrakis(triphenylphosphine)palladium (0) (1.179 g, 1.02 mmol), and then 1,3-dimethylpyrimidine-2,4,6(1*H*,3*H*,5*H*)-trione (3.98 g, 25.5 mmol) were added, and the solution was degassed and flushed with nitrogen once. The reaction mixture was stirred overnight. Pyrrolidine-1-carbodithioic acid, ammonia salt (0.251 g, 1.53 mmol) was added as a palladium scavenger, and the reaction was stirred for 30 minutes. Ethyl acetate (100 mL) was added and the mixture was filtered through diatomaceous earth, washing with more ethyl acetate. The crude material was concentrated and used without further purification (9.08 g, 97%). ^{1}H NMR (500 MHz, dimethylsulfoxide-d_6) δ ppm 8.69 (s, 1H), 7.42 (d, 2H), 7.35 (t, 2H), 7.31-7.25 (m, 1H), 7.19-7.10 (m, 4H), 6.89 (d, 1H), 6.82 (dd, 1H), 5.91 (dd, 1H), 5.62 (d, 1H), 5.05 (t, 1H), 5.02 (d, 1H), 4.95 (d, 1H), 4.64 (m, 1H), 4.43 (dd, 1H), 4.33 (d, 1H), 3.68 (m, 2H), 3.52 (dd, 1H), 2.78 (d, 1H), 2.03 (s, 3H), 1.88 (s, 3H), 1.04 (s, 9H). MS (ESI) *m/z* 819.2 $(M+H)^+$; ^{13}C NMR (126 MHz, DMSO-d_6) δ 167.95, 167.41, 163.39, 162.67 (d, J = 248.1 Hz), 153.26, 151.46, 150.67 (d, J = 13.3 Hz), 138.10, 137.76, 135.61, 134.84, 131.55, 131.12 (d, J = 8.6 Hz), 129.08 (d, J = 3.2 Hz), 128.83, 128.50, 128.16, 127.71, 127.53, 126.08, 125.82, 121.37, 118.46, 116.59, 116.41, 116.19, 113.28, 81.58, 79.42, 74.01, 73.00, 70.36, 61.16, 30.10, 27.64, 18.80, 18.64; HRMS (ESI-QTOF+) $[M+Na]^+$ calc. for $C_{43}H_{39}Cl_2FN_2NaO_7$ m/z 839.1731, found 839.1717 (1.74 ppm); $[\alpha]^{20}_{D}$ -55.0 (*c* 1.0, $CHCl_3$). ^{1}H NMR, hydrogen-1 nuclear magnetic resonance, ^{13}C NMR, carbon-13 nuclear magnetic resonance, HRMS, high resolution mass spectrometry.

Tert-butyl (7R,16S)-10-(benzyloxy)-19,23-dichloro-1-(4-fluorophenyl)-20,22-dimethyl-16-{[(4-methylbenzene-1-sulfonyl)oxy]methyl}-7,8,15,16-tetrahydro-18,21-etheno-13,9-(metheno)-6,14,17-trioxa-2-thia-3,5-diazacyclononadeca[1,2,3-cd]indene-7-carboxylate (S-46). S-45 (8.09 g, 9.89 mmol) in dichloromethane (95 mL) was cooled to 0°C. To the mixture was added *p*-Toluenesulfonyl chloride (4.9 g, 14.8 mmol), and 1,4-diazabicyclo[2.2.2]octane (3.9 g, 19.8 mmol). The reaction was stirred at 0°C for 1 hour. The mixture was diluted with 50 mL dichloromethane and quenched with 50 mL saturated aqueous NH_4Cl. Water (50 mL) was added and the layers were separated. The organic layer was washed with brine, dried over Na_2SO_4, filtered, and concentrated. The crude material was purified by silica gel chromatography using 10–35% ethyl acetate in heptanes to provide the title compound (8.04 g, 84%). ^{1}H NMR (500 MHz, dimethylsulfoxide-d_6) δ ppm 8.71 (s, 1H), 7.81 (d, 2H), 7.45 (d, 2H), 7.42 (d, 2H), 7.36 (td, 2H), 7.32-7.28 (m, 1H), 7.20 (td, 2H), 7.17-7.13 (m, 2H), 6.90 (d, 1H), 6.83 (dd, 1H), 5.93

(dd, 1H), 5.62 (d, 1H), 5.05 (d, 1H), 4.97 (d, 1H), 4.86 (m, 1H), 4.45-4.35 (m, 3H), 4.26 (d, 1H), 3.50 (dd, 1H), 2.80 (d, 1H), 2.38 (s, 3H), 2.00 (s, 3H), 1.88 (s, 3H), 1.06 (s, 9H). MS (ESI) *m/z* 971.2 $(M+H)^+$; ^{13}C NMR (101 MHz, DMSO-d_6) δ 167.95, 163.28, 162.66 (d, *J* = 248.2 Hz), 153.24, 151.53, 150.10, 149.65, 145.56, 138.21, 137.68, 135.70, 134.96, 132.53, 131.93, 131.13 (d, *J* = 8.6 Hz), 130.62, 128.99 (d, *J* = 3.3 Hz), 128.81, 128.30, 128.17, 128.14, 127.67, 127.25, 125.83 (d, *J* = 4.5 Hz), 120.87, 116.67, 116.45, 115.81, 113.14, 81.57, 75.81, 73.86, 71.06, 70.27, 70.06, 30.14, 27.61, 21.49, 18.76, 18.61; HRMS (ESI-QTOF+) $[M+H]^+$ calc. for $C_{50}H_{46}Cl_2FN_2O_9S_2$ m/z 971.2000, found 971.2004 (-0.4 ppm); $[\alpha]^{20}_D$ -52.0 (*c* 1.0, $CHCl_3$). ^1H NMR, hydrogen-1 nuclear magnetic resonance, ^{13}C NMR, carbon-13 nuclear magnetic resonance, HRMS, high resolution mass spectrometry.

Tert-butyl (7R,16R)-10-(benzyloxy)-19,23-dichloro-1-(4-fluorophenyl)-20,22-dimethyl-16-[(4-methylpiperazin-1-yl)methyl]-7,8,15,16-tetrahydro-18,21-etheno-13,9-(metheno)-6,14,17-trioxa-2-thia-3,5-diazacyclononadeca[1,2,3-cd]indene-7-carboxylate (S-47). To an ambient solution of S-46 (2.98 g, 3.07 mmol) in *N*,*N*-dimethylformamide (10 mL) was added 1-methylpiperazine (10.20 mL). The reaction was heated to 40°C for 24 hours. Another 2 mL of 1-methyl-piperazine was added and the reaction was heated at 35°C overnight. The reaction was cooled to room temperature, and the solvents were removed by rotary evaporation. The crude material was cooled in an ice bath, stirred, and diluted sequentially with ethyl acetate (100 mL) and water (100 mL). The layers were separated, and the aqueous layer was extracted with additional ethyl acetate (2 × 100 mL). The combined organics were washed with brine (2 × 100 mL), dried over anhydrous sodium sulfate, filtered, and concentrated under reduced pressure. The residue was diluted with toluene (5 mL) and was purified by normal-phase MPLC (Biotage® Isolera, 100 g Biotage® Ultra SiO_2 column), eluting with a gradient of 0–6% methanol in dichloromethane to provide the title compound (2.16 g, 78%). ^1H NMR (500 MHz, dimethylsulfoxide-d_6) δ ppm 8.74 (s, 1H), 7.41 (m, 2H), 7.39 (m, 2H), 7.35 (m, 1H), 7.20 (m, 4H), 6.90 (m, 1H), 6.81 (m, 1H), 6.00 (m, 1H), 5.67 (s, 1H), 5.02 (q, 2H), 4.75 (m, 1H), 4.44 (m, 2H), 3.60 (m, 1H), 3.58 (m, 1H), 2.80 (m, 1H), 2.48 (m, 3H), 2.40 (m, 4H), 2.30 (m, 4H), 2.15 (s, 3H), 2.08 (s, 3H), 1.89 (s, 3H), 1.09 (s, 9H). MS (ESI) *m/z* 899.4 $(M+H)^+$; ^{13}C NMR (126 MHz, DMSO-d_6) δ 168.00, 167.43, 163.33, 162.70 (d, *J* = 248.2 Hz), 153.25, 151.59, 150.18, 149.69, 145.59, 138.25, 137.71, 135.72, 135.03, 132.60, 131.98, 131.16 (d, *J* = 8.6 Hz), 130.64, 129.03 (d, *J* = 3.3 Hz), 128.84, 128.35, 128.19, 128.17, 127.69, 127.31, 125.89 (d, *J* = 9.1 Hz), 120.89, 118.42, 116.65, 116.48, 115.83, 113.23, 81.62, 75.87, 73.92, 71.08, 70.35, 70.07, 30.22, 27.64, 21.52, 18.76, 18.63; HRMS (ESI-QTOF+) $[M+H]^+$ calc. for $C_{48}H_{50}Cl_2FN_4O_6S$ m/z 899.2807, found 899.2799 (0.8 ppm); $[\alpha]^{20}_D$ -80.0 (*c* 1.0, $CHCl_3$). ^1H NMR, hydrogen-1 nuclear magnetic resonance; MPLC, medium pressure liquid chromatography, ^{13}C NMR, carbon-13 nuclear magnetic resonance, HRMS, high resolution mass spectrometry.

Tert-butyl (7R,16R)-19,23-dichloro-1-(4-fluorophenyl)-10-hydroxy-20,22-dimethyl-16-[(4-methylpiperazin-1-yl)methyl]-7,8,15,16-tetrahydro-18,21-etheno-13,9-(metheno)-6,14,17-trioxa-2-thia-3,5-diazacyclononadeca[1,2,3-cd]indene-7-carboxylate (S-48). S-47 (1.943 g, 2.16 mmol) in tetrahydrofuran (11 mL)

was added to 5% Pd/C (1.801 g) in a 20-mL Barnstead Hast C pressure reactor. The reactor was purged with argon gas. The mixture was stirred at 1600 rpm under 50 psi of hydrogen at 25°C. After 17.3 hours, the reaction was vented. The mixture was filtered through a filter funnel with a polyethylene frit packed with diatomaceous earth. The mixture was concentrated, and the crude material was taken up in ether and a small amount of dichloromethane. The mixture was filtered through diatomaceous earth, washing with ether/dichloromethane. The solvent was removed on a rotovap, and the material was placed on high vacuum overnight to provide the title compound (1.61 g, 92%). ^{1}H NMR (500 MHz, dimethylsulfoxide-d_6) δ ppm 9.11 (s, 1H), 8.72 (s, 1H), 7.20 (m, 4H), 6.67 (m, 2H), 5.96 (m, 1H), 5.50 (s, 1H), 4.69 (m, 1H), 4.41 (m, 1H), 4.37 (m, 1H), 3.54 (dd, 1H), 3.58 (m, 1H), 2.62 (m, 2H), 2.22-2.50 (m, 9H), 2.18 (s, 6H), 1.88 (s, 3H), 1.09 (s, 9H). MS (ESI) *m/z* 811.2 $(M+H)^+$; ^{13}C NMR (101 MHz, Chloroform-*d*) δ 168.48, 167.53, 163.43, 162.65 (d, *J* = 250.2 Hz), 152.58, 150.69, 150.15, 149.71, 138.26, 135.18 (d, *J* = 9.3 Hz), 131.55, 130.43 (d, *J* = 8.3 Hz), 128.90 (d, *J* = 4.6 Hz), 127.40, 123.72, 121.41, 116.31, 116.07, 115.85, 115.82, 81.87, 76.33, 74.14, 59.20, 55.00, 53.56, 45.89, 30.48, 27.57, 18.61, 18.54; HRMS (ESI-QTOF+) $[M+H]^+$ calc. for $C_{41}H_{44}ClFNO_6S$ m/z 809.2337, found 809.2329 (1.04 ppm); $[\alpha]^{20}_{D}$ -126.0 (*c* 1.0, $CHCl_3$). ^{1}H NMR, hydrogen-1 nuclear magnetic resonance, ^{13}C NMR, carbon-13 nuclear magnetic resonance, HRMS, high resolution mass spectrometry.

(R)-2-(4-((1,4-dioxan-2-yl)methoxy)phenyl)-4,4,5,5-tetramethyl-1,3,2-dioxaborolane (S-49). (*S*)-(1,4-dioxan-2-yl)methanol (160 mg, 1.35 mmol) was dissolved in dichloromethane (6 mL). The mixture was cooled to 0°C. Triethylamine (0.217 mL, 1.56 mmol) was added. Methanesulfonyl chloride (0.116 mL¸ 1.49 mmol) was then added dropwise. The mixture was allowed to warm to room temperature. After 2 hours, saturated aqueous sodium bicarbonate (3 mL) was added. The layers were separated, and the organic portion was washed with brine (5 mL). The aqueous portions were combined and back-extracted with dichloromethane (10 mL). The organic portions were combined and dried over anhydrous sodium sulfate and filtered. The solvent was removed under vacuum. To this material was added 4-(4,4,5,5-tetramethyl-1,3,2-dioxaborolan-2-yl)phenol (200 mg, 0.91 mmol) and *N*,*N*-dimethylformamide (5 mL). Cesium carbonate (592 mg, 1.82 mmol) was added, and the mixture was heated to 90°C for 16 hours. The mixture was cooled, and saturated aqueous ammonium chloride (2 mL) was added. The mixture was diluted with ethyl acetate (20 mL) and washed with water (10 mL) twice. The organic portion was washed with brine (10 mL) and dried on anhydrous sodium sulfate. After filtration, the mixture was concentrated under vacuum and was purified by flash column chromatography on silica gel using a 10–30% gradient of ethyl acetate in heptanes to provide the title compound. ^{1}H NMR (500 MHz, dimethylsulfoxide-d_6) δ ppm 7.60 (d, 2H), 6.94 (d, 2H), 3.98 (d, 2H), 3.88-3.74 (m, 3H), 3.68-3.59 (m, 2H), 3.52-3.46 (m, 1H), 3.42-3.37 (m, 1H), 1.27 (s, 12H). MS (ESI) *m/z* 221.3 (M-*tert*-butyl carboxylate)$^+$; ^{13}C NMR (101 MHz, DMSO) δ 160.96, 136.21, 113.93, 83.33, 73.17, 67.34, 67.26, 65.81, 65.76, 24.65; HRMS (ESI-QTOF+) [M+Na]+ calc. for $C_{17}H_{25}BNaO_5$ m/z 342.1724, found 342.1719 (1.44 ppm); $[\alpha]^{20}_{D}$ +8.0 (*c* 1.0, $CHCl_3$). ^{1}H NMR, hydrogen-1 nuclear magnetic resonance, ^{13}C NMR, carbon-13 nuclear magnetic resonance, HRMS, high resolution mass spectrometry.

(R)-(2-(4-((1,4-dioxan-2-yl)methoxy)phenyl)pyrimidin-4-yl)methanol (S-50). S-49 (138 mg, 0.43 mmol) and (2-bromopyrimidin-4-yl)methanol (94 mg, 0.50 mmol) were dissolved in 1,4-dioxane (2 mL). Aqueous sodium carbonate (2 M, 0.65 mL) was added. The mixture was degassed and flushed with nitrogen three times. Dichloro[1,1′-bis(diphenylphosphino)ferrocene]palladium (II) dichloromethane adduct (35 mg, 0.043 mmol) was added, and the mixture was degassed and flushed with nitrogen once. The mixture was stirred at 75°C for 16 hours. The mixture was cooled, diluted with ethyl acetate (10 mL), washed with water (10 mL), washed with brine (10 mL), and dried over anhydrous sodium sulfate. The mixture was concentrated and purified by flash column chromatography on silica gel using a 30–70% gradient of ethyl acetate in heptanes to provide the title compound. ^{1}H NMR (500 MHz, dimethylsulfoxide-d_6) δ ppm 8.81 (d, 1H), 8.33 (d, 2H), 7.42 (d, 1H), 7.07 (d, 2H), 5.65 (t, 1H), 4.61 (d, 2H), 4.04 (d, 2H), 3.92-3.76 (m, 3H), 3.69-3.61 (m, 2H), 3.54-3.48 (m, 1H), 3.45-3.40 (m, 1H). MS (ESI) *m/z* 303.2 (M+H)$^+$; ^{13}C NMR (101 MHz, DMSO) δ 160.96, 136.21, 113.93, 83.33, 73.17, 67.34, 67.26, 65.81, 65.76, 24.65; HRMS (ESI-QTOF+) [M+H]$^+$ calc. for $C_{16}H_{19}N_2O_4$ m/z 303.1339, found 303.1334 (1.89 ppm); $[\alpha]^{20}_{D}$ +14.0 (*c* 1.0, $CHCl_3$). ^{1}H NMR, hydrogen-1 nuclear magnetic resonance, ^{13}C NMR, carbon-13 nuclear magnetic resonance, HRMS, high resolution mass spectrometry.

Tert-butyl (7R,16R)-19,23-dichloro-10-{[2-(4-{[(2R)-1,4-dioxan-2-yl]methoxy}phenyl)pyrimidin-4-yl]methoxy}-1-(4-fluorophenyl)-20,22-dimethyl-16-[(4-methylpiperazin-1-yl)methyl]-7,8,15,16-tetrahydro-18,21-etheno-13,9-(metheno)-6,14,17-trioxa-2-thia-3,5-diazacyclononadeca[1,2,3-cd]indene-7-carboxylate (S-51). To an ambient solution of S-50 (112 mg, 0.370 mmol), S-48 (200 mg, 0.247 mmol), and triphenylphosphine (194 mg, 0.741 mmol) in a solvent mixture of toluene/THF (1/1, 1.2 mL) was added (E)-N1,N1,N2,N2-tetramethyldiazene-1,2-dicarboxamide (128 mg, 0.741 mmol), and the reaction was heated to 50°C. A precipitate formed, and the mixture was filtered through a 0.45-μm syringe filter and was directly purified by MPLC (Biotage Isolera, 10 g ultra SiO2), eluting with a gradient of 0–6% MeOH in dichloromethane to give the desired product, a foam (200 mg, 74%). ^{1}H NMR (500 MHz, dimethylsulfoxide-d_6) δ ppm 8.82 (d, 1H), 8.71 (s, 1H), 8.30 (d, 2H), 7.41 (d, 1H), 7.21-7.12 (m, 4H), 7.04 (d, 2H), 6.91 (d, 1H), 6.80 (dd, 1H), 6.03 (dd, 1H), 5.65 (d, 1H), 5.16 (q, 2H), 4.70 (q, 1H), 4.47-4.34 (m, 2H), 4.03-3.99 (m, 3H), 3.86 (m, 1H), 3.81 (dd, 1H), 3.74 (dd, 1H), 3.70-3.56 (m, 3H), 3.51-3.45 (m, 1H), 3.41 (dd, 1H), 2.87 (d, 1H), 2.67-2.57 (m, 2H), 2.46-2.21 (m, 7H), 2.11 (bs, 3 H), 2.07 (s, 3H), 1.86 (s, 3H), 1.03 (s, 9H). MS (ESI) *m/z* 1093.1 (M+H)$^+$; ^{13}C NMR (101 MHz, DMSO) δ 167.46, 166.97, 166.20, 162.87, 162.78, 162.20 (d, *J* = 248.2 Hz), 160.71, 158.68, 158.11, 152.81, 150.37, 150.28, 150.21, 137.76, 135.30, 134.31, 131.13, 130.67 (d, *J* = 8.6 Hz), 129.62, 129.48, 128.60, 127.91, 126.96, 125.55, 125.38, 120.91, 117.96, 116.10 (d, *J* = 22.0 Hz), 115.48, 115.28, 114.49, 112.61, 81.22, 76.23, 73.20, 69.78, 67.59, 67.36, 65.85, 65.79, 58.09, 54.66, 53.31, 45.63,37.90, 35.82, 29.57, 27.16, 18.41, 18.19; HRMS (ESI-QTOF+) [M+H]$^+$ calc. for $C_{57}H_{60}Cl_2FN_6O_9S$ m/z 1093.3498, found 1093.3492 (0.58 ppm); $[\alpha]^{20}_{D}$ -42.0 (*c* 1.0, $CHCl_3$). ^{1}H NMR, hydrogen-1 nuclear magnetic resonance; MPLC, medium pressure

liquid chromatography, ^{13}C NMR, carbon-13 nuclear magnetic resonance, HRMS, high resolution mass spectrometry.

(7R,16R)-19,23-dichloro-10-{[2-(4-{[(2R)-1,4-dioxan-2-yl]methoxy}phenyl)pyrimidin-4-yl]methoxy}-1-(4-fluorophenyl)-20,22-dimethyl-16-[(4-methylpiperazin-1-yl)methyl]-7,8,15,16-tetrahydro-18,21-etheno-13,9-(metheno)-6,14,17-trioxa-2-thia-3,5-diazacyclononadeca[1,2,3-cd]indene-7-carboxylic acid (ABBV-467). To an ambient solution of S-51 (200 mg, 0.183 mmol) in dichloromethane (1.5 mL) was added trifluoroacetic acid (1.500 mL). The reaction was stirred at room temperature for 6 hours, and concentrated to an oil. The oil was diluted in dichloromethane (5 mL) and concentrated under reduced pressure twice. The residue was dissolved in 10 mL dichloromethane and washed with 5 mL saturated aqueous bicarbonate solution. The organic layer was dried with anhydrous sodium sulfate, filtered, and concentrated to a yellow foam. The residue was purified by RP-HPLC on a Gilson 2020 system (Phenomenex Luna 250 × 50 mm, 70 mL/min) eluting with a gradient of 5–100% acetonitrile in water containing 0.1% ammonium acetate over 30 minutes to give the title compound (134 mg, 71%). ^{1}H NMR (500 MHz, dimethylsulfoxide-d_6) δ ppm 9.42 (bs, 1H), 8.84 (d, 1H), 8.76 (s, 1H), 8.32 (d, 2H), 7.44 (d, 1H), 7.21-7.15 (m, 4H), 7.08 (d, 2H), 6.91 (d, 1H), 6.83 (dd, 1H), 6.28 (m, 1H), 5.79 (d, 1H), 5.21 (q, 2H), 4.93 (m, 1H), 4.51-4.42 (m, 2H), 4.05 (m, 2H), 3.92-3.88 (m, 1H), 3.87 (dd, 1H), 3.78 (dd, 1H), 3.71-3.62 (m, 3H), 3.53 (m, 1H), 3.24 (m, 4H), 3.12-2.91 (m, 6H), 2.89-2.81 (m, 2H), 2.80 (s, 3H), 1.99 (s, 3H), 1.96 (s, 3H). MS (ESI) *m/z* 1037.1 $(M+H)^+$; ^{13}C NMR (101 MHz, DMSO) δ 171.00, 166.86, 166.50, 163.58, 162.73, 162.15 (d, *J* = 248.1 Hz), 160.71, 158.17, 152.99, 150.05, 149.98, 149.87, 137.07, 135.04, 134.70, 131.16, 130.67 (d, *J* = 8.6 Hz), 129.70, 129.52, 128.79 (d, *J* = 3.3 Hz), 127.64, 127.43, 127.30, 124.96, 119.49, 118.25, 116.03 (d, *J* = 21.8 Hz), 115.55, 114.53, 112.72, 111.99, 75.89, 73.25, 71.53, 69.59, 67.62, 67.39, 65.90, 65.84, 57.67, 53.51, 51.87, 43.93, 30.24, 18.31, 18.25; HRMS (ESI-QTOF+): $[M+H]^+$ calc. for $C_{53}H_{52}Cl_2FN_6O_9S$ m/z 1037.2872, found 1037.2869 (0.32 ppm); $[\alpha]^{20}_{D}$ -132.0 (*c* 1.0, $CHCl_3$) ^{1}H NMR, hydrogen-1 nuclear magnetic resonance; RP-HPLC, reverse phase high-performance liquid chromatography, ^{13}C NMR, carbon-13 nuclear magnetic resonance, HRMS, high resolution mass spectrometry.

NMR and MS spectra for compounds S-1 through S-51 and ABBV-467.

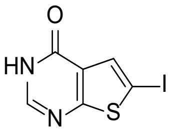

6-iodothieno[2,3-d]pyrimidin-4(3H)-one (S-1).

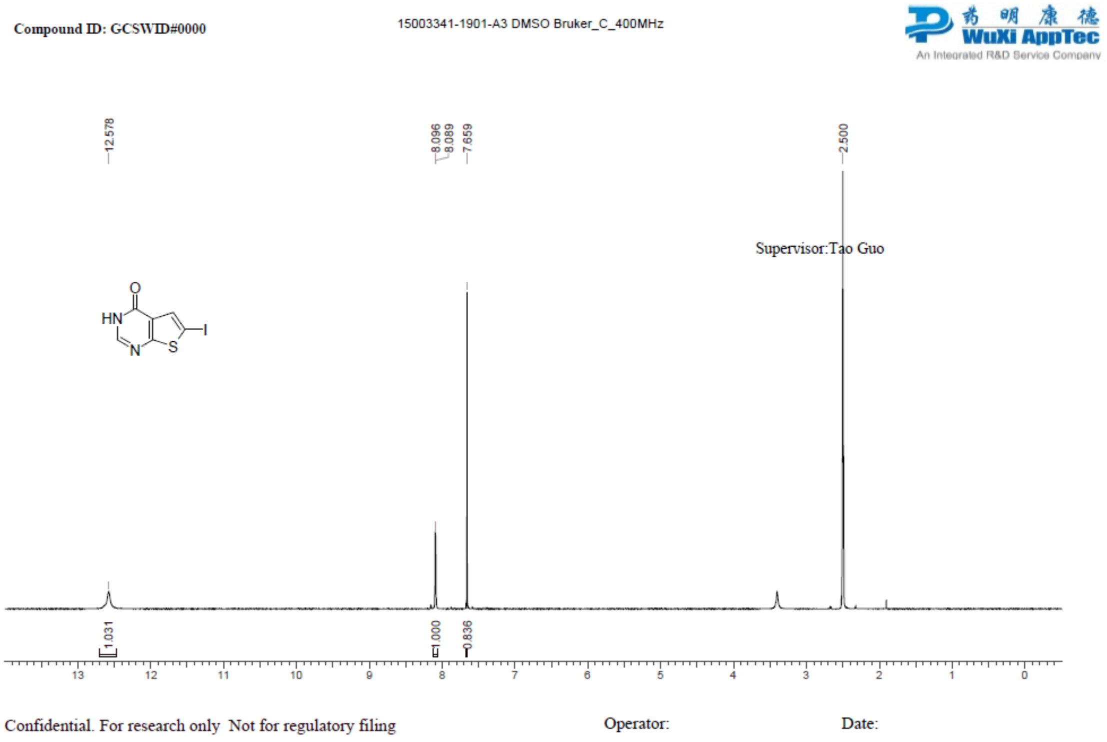

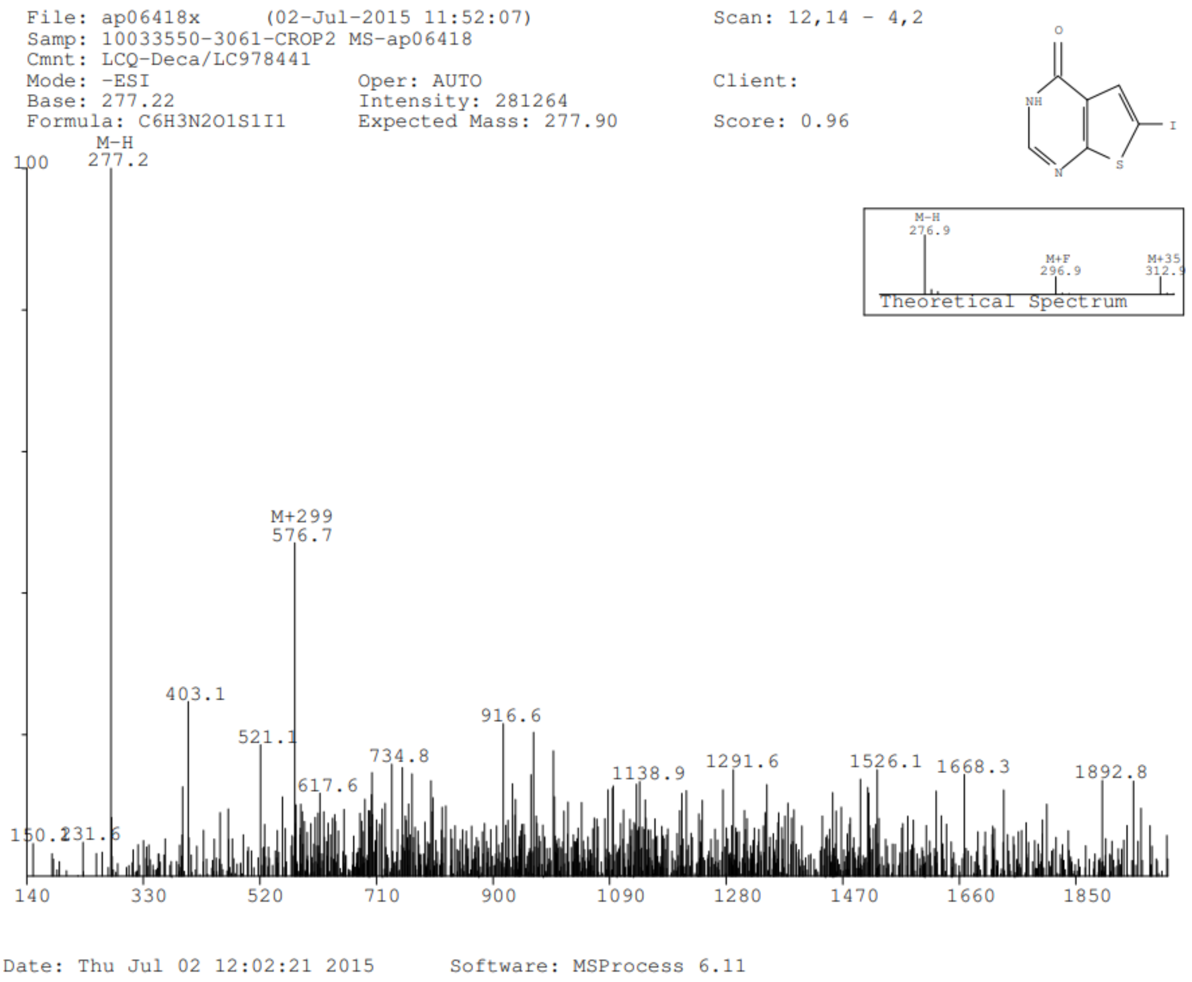

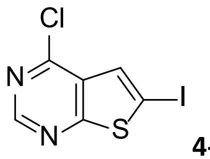

4-chloro-6-iodothieno[2,3-d]pyrimidine (S-2).

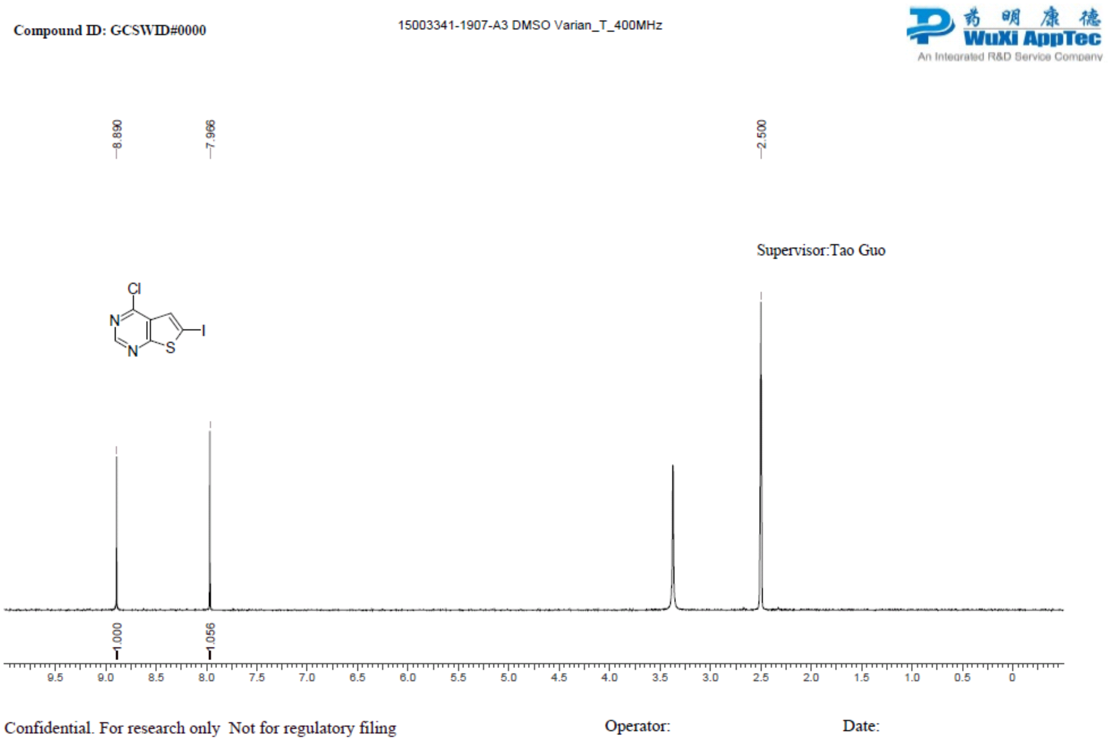

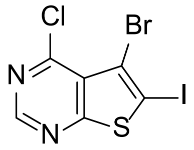

5-bromo-4-chloro-6-iodothieno[2,3-d]pyrimidine (S-3).

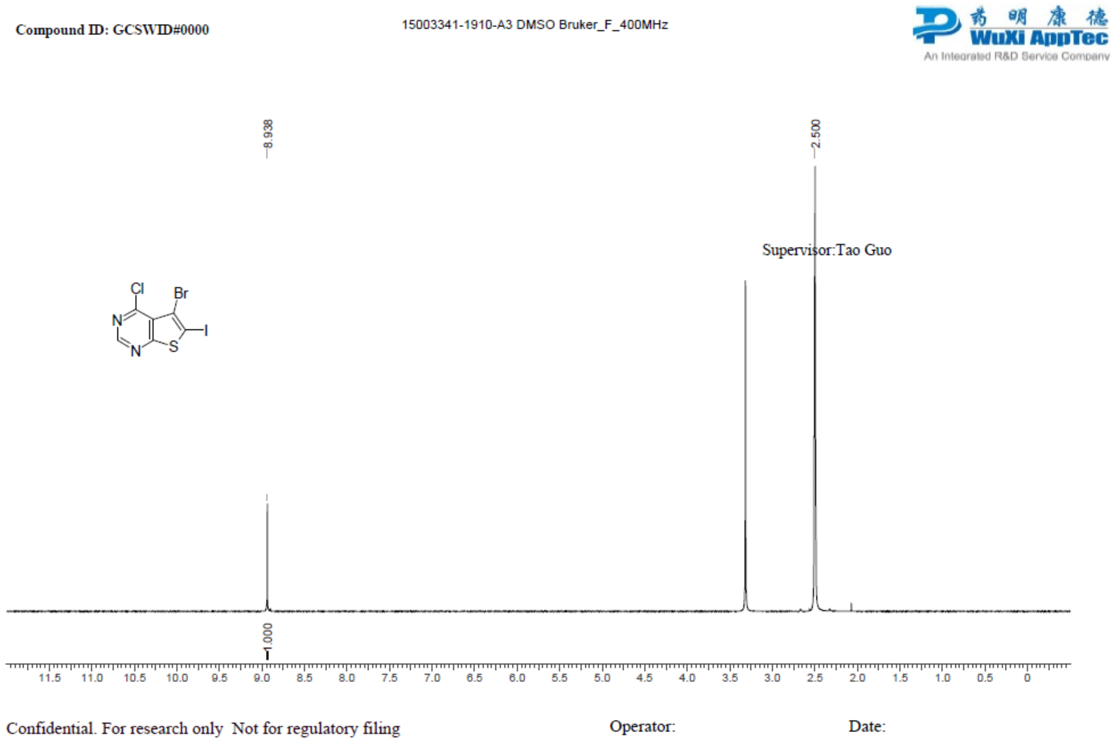

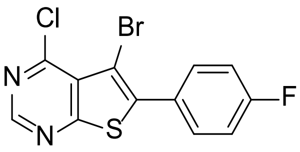

5-bromo-4-chloro-6-(4-fluorophenyl)thieno[2,3-d]pyrimidine (S-4).

abbvie

10033550-3253-FIN in CDCL3 BC#2387 $10
mrs400

Acq: VnmrJ VERSION 3.2 REVISION A/mrs400
Proc: VnmrJ VERSION 3.2 REVISION A/i600

Chemist: MATTHEW HANSEN
Solvent and water peaks subtracted
Experiment: s2pul

nmr3313561

May 13 2016

ID=91.30
P =88.00

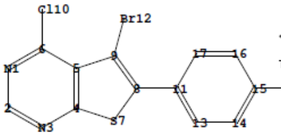

HoseCodePredictions

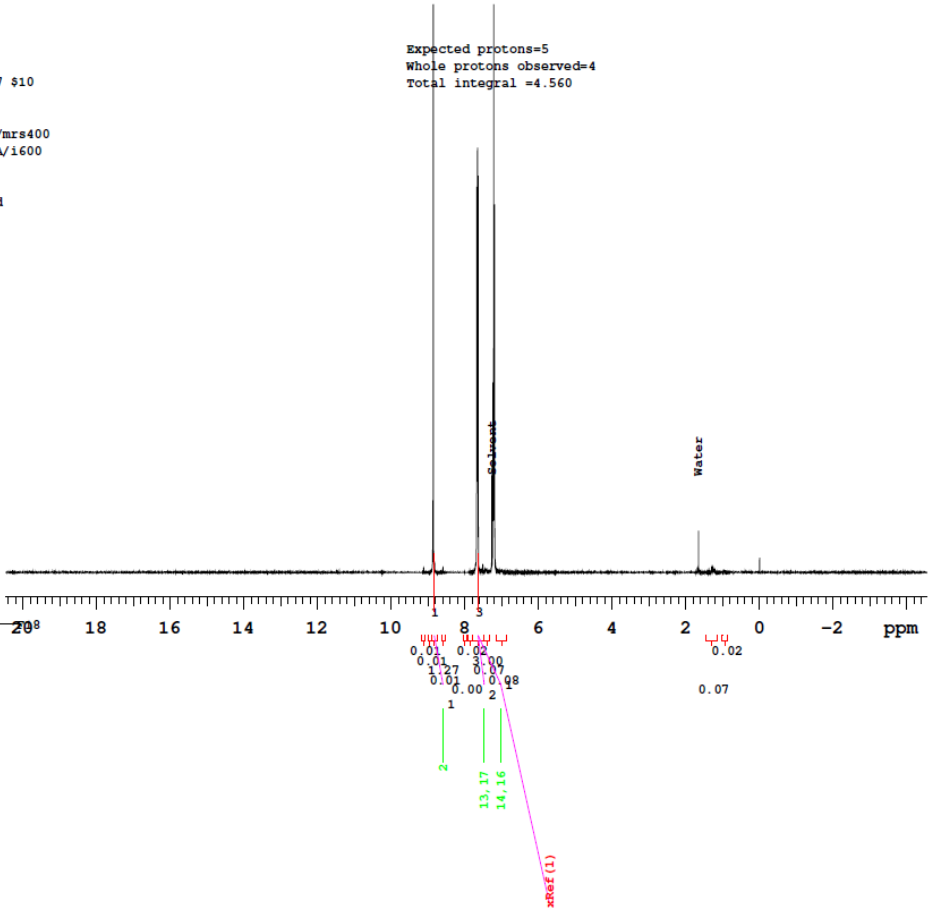

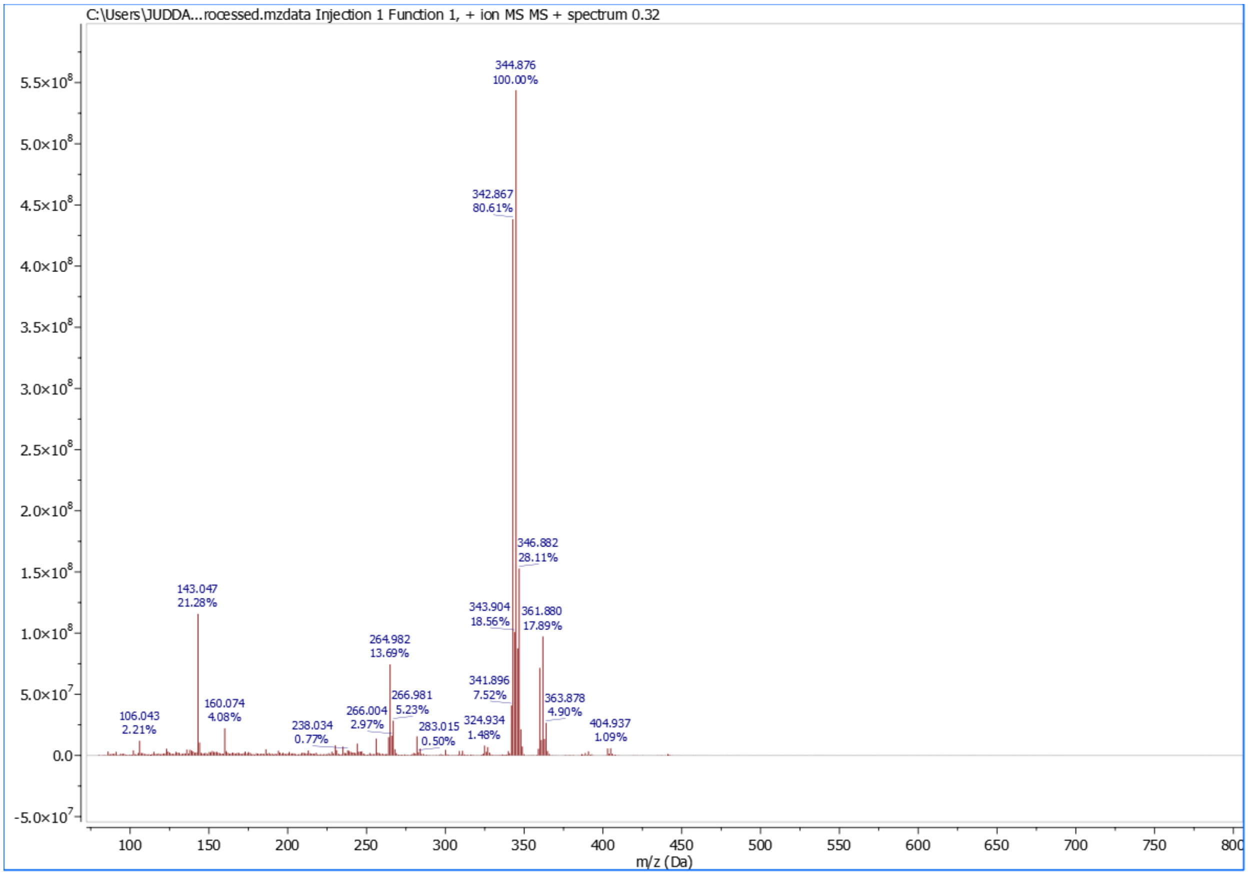

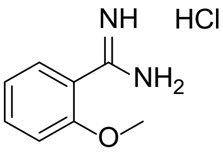

2-methoxybenzimidamide hydrochloride (S-5).

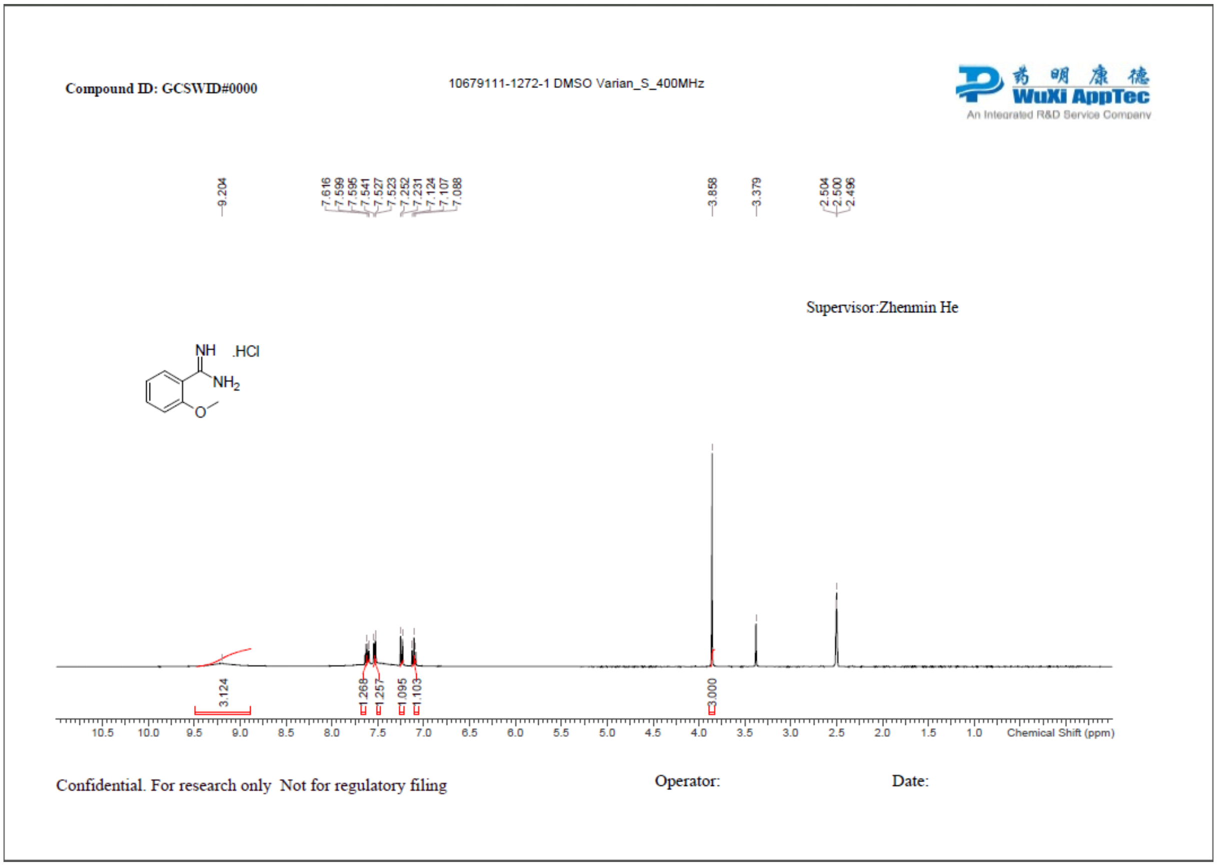
Compound ID: GCSWID#0000
10679111-1272-1 DMSO Varian_S_400MHz
WuXi AppTec
An Integrated R&D Service Company
9.204
7.616 7.599 7.595 7.541 7.527 7.523 7.252 7.231 7.124 7.107 7.088
3.858
3.379
2.504 2.500 2.496
Supervisor:Zhenmin He
NH .HCl
NH2
O
3.124
1.268 1.257 1.095 1.103
3.000
10.5 10.0 9.5 9.0 8.5 8.0 7.5 7.0 6.5 6.0 5.5 5.0 4.5 4.0 3.5 3.0 2.5 2.0 1.5 1.0 Chemical Shift (ppm)
Confidential. For research only Not for regulatory filing
Operator:
Date:

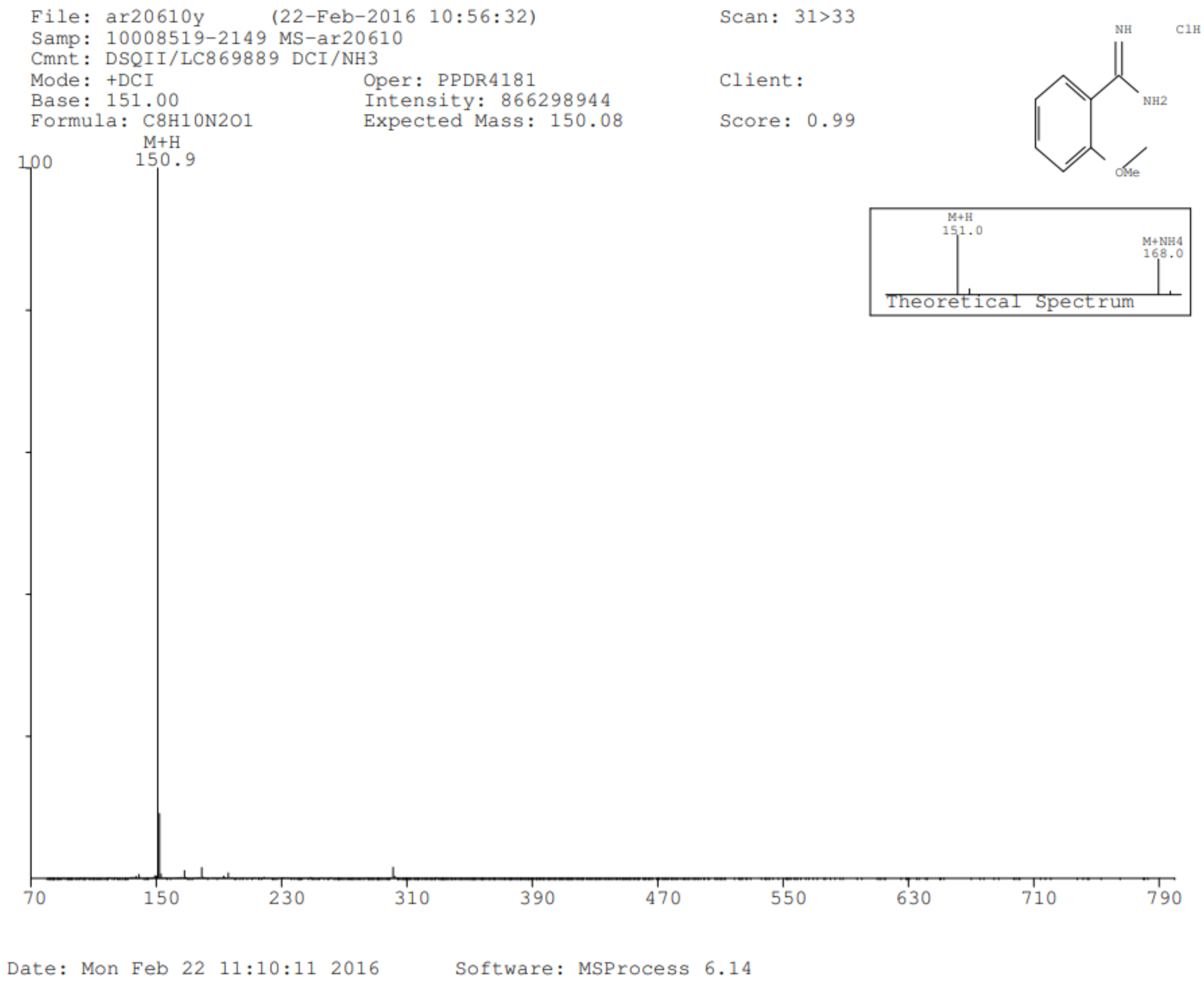

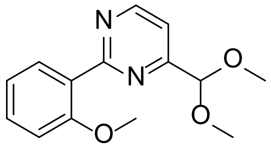

4-(dimethoxymethyl)-2-(2-methoxyphenyl)pyrimidine (S-6).

Compound ID: GCSWID#0000

10679111-1279-1 CDCl3 Varian_S_400MHz

WuXi AppTec
An Integrated R&D Service Company

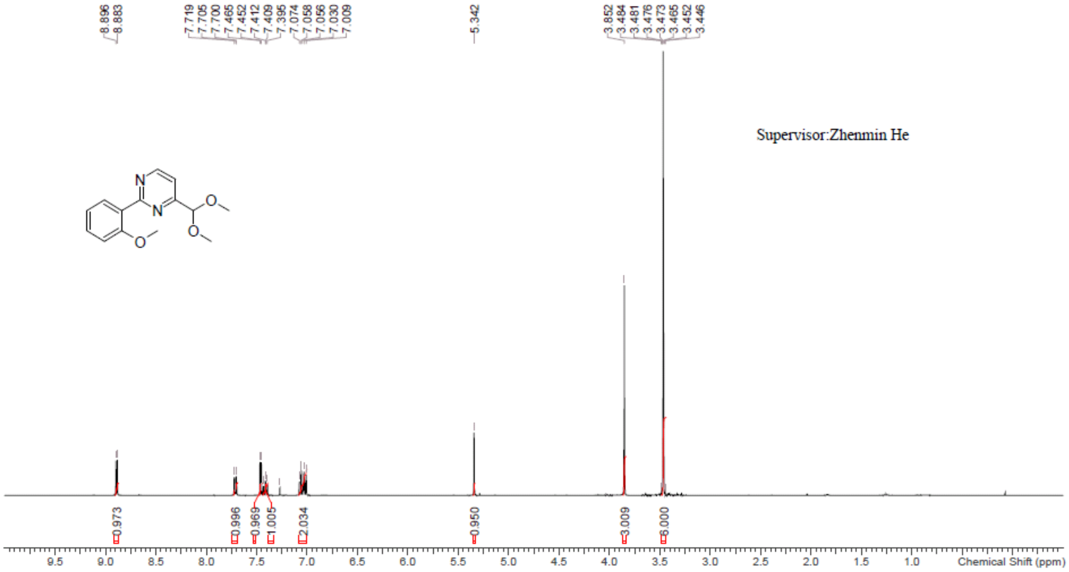

Confidential. For research only Not for regulatory filing

Operator:

Date:

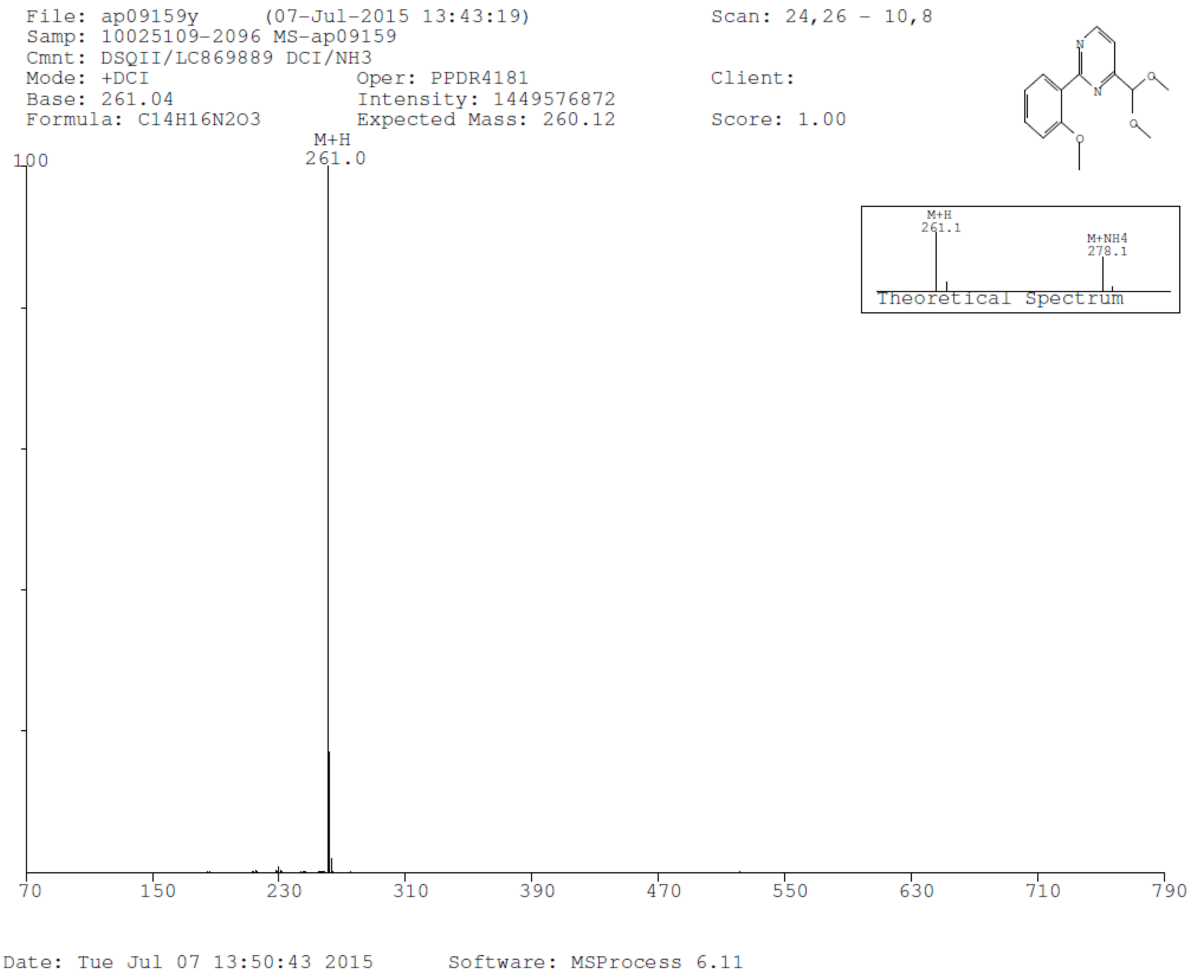

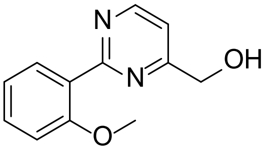

(2-(2-methoxyphenyl)pyrimidin-4-yl)methanol (S-7)..

Compound ID: RSU-0428

10679111-1284-1 DMSO Varian_S_400MHz

WuXi AppTec
An Integrated R&D Service Company

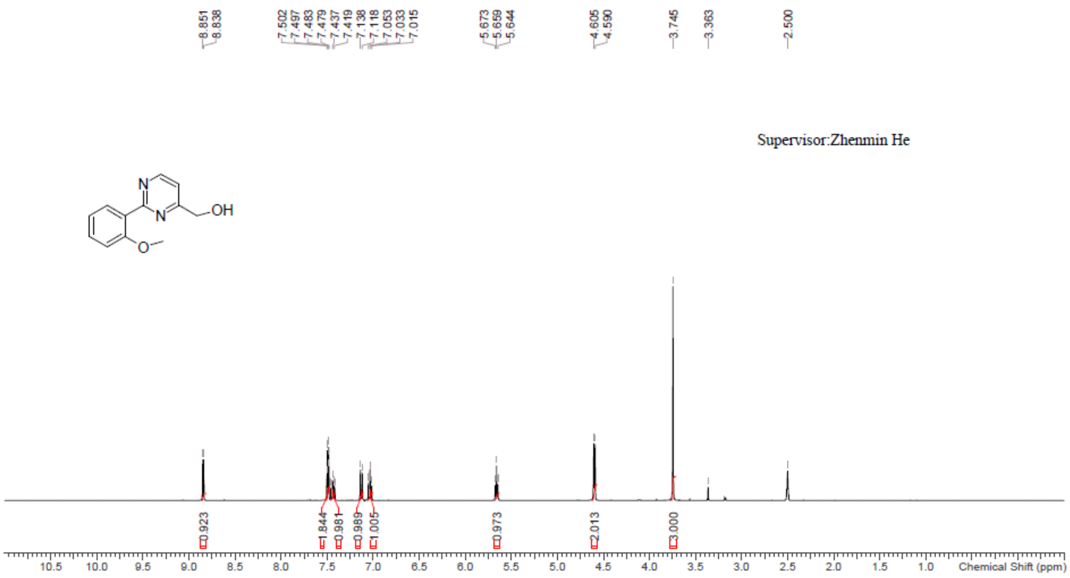

Confidential. For research only Not for regulatory filing

Operator:

Date:

Compound Spectrum SmartFormula Report

Analysis Info

Analysis Name	D:\Data\External\Abbvie\Brady\03192018\Abbvie-Brady-O_37_01_1893.d	Acquisition Date	3/19/2018 10:36:03 AM	
Method	MethodSet-FIA-MS-C1-30_A1-70_B1-2min-03192018.m	Operator	Demo User	
Sample Name	Abbvie-Brady-O	Instrument	impact II	1825265.10104
Comment				

Acquisition Parameter

Source Type	ESI	Ion Polarity	Positive	Set Nebulizer	0.4 Bar
Focus	Active	Set Capillary	4500 V	Set Dry Heater	200 °C
Scan Begin	100 m/z	Set End Plate Offset	-500 V	Set Dry Gas	4.0 l/min
Scan End	3000 m/z	Set Charging Voltage	2000 V	Set Divert Valve	Source
		Set Corona	0 nA	Set APCI Heater	0 °C

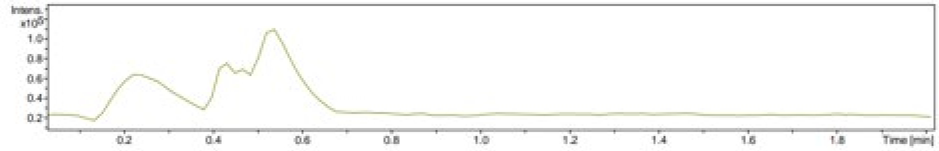

+MS, 0.5-0.5min #27-29

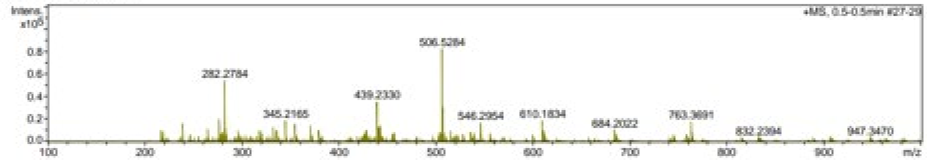

Meas. m/z	z	Adduct	Ion Formula	m/z	Sum Formula	|err| [mDa]	err [ppm]	N-Rule	Score
217.0970	1+	M+H	C12H13N2O2	217.0972	C12H12N2O2	0.1	0.6	ok	100.00

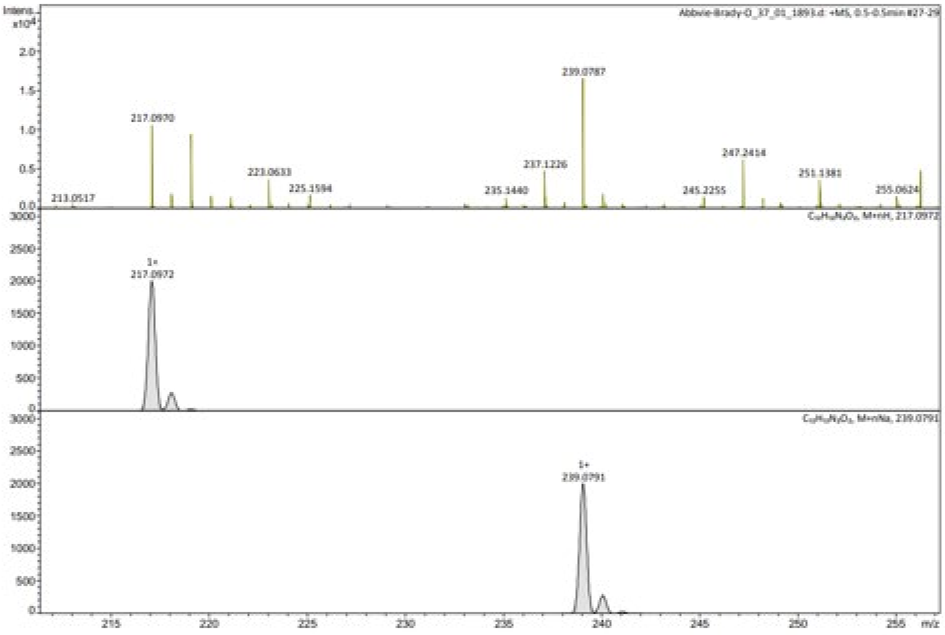

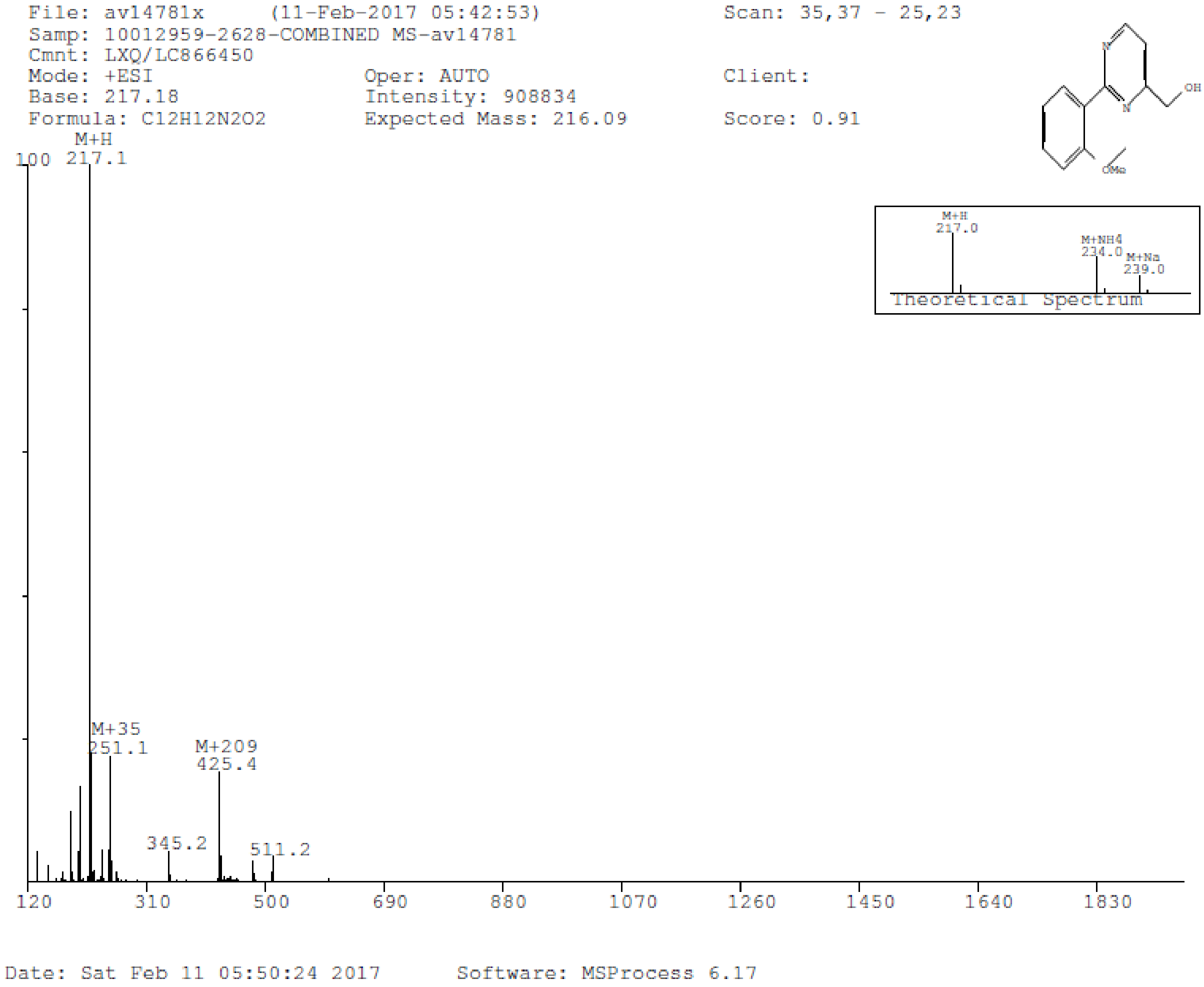

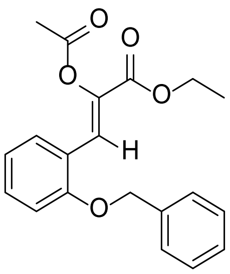

Ethyl 2-acetoxy-3-(2-(benzyloxy)phenyl)acrylate (S-8).

abbvie

10025373-1932-B in CDCL3 $15 BC762 1mg
Temp = 27 C
C20H20O5
v501

Acq: VnmrJ VERSION 3.2 REVISION A/v501
Proc: VnmrJ VERSION 3.2 REVISION A/coffee

Chemist: ROBERT MANTEI
Experiment: s2pul

nmr3338676

Jul 15 2016

Expected protons=20
Whole protons observed=19
Total integral =19.220

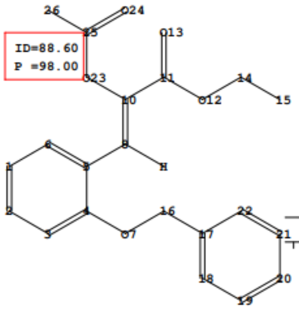

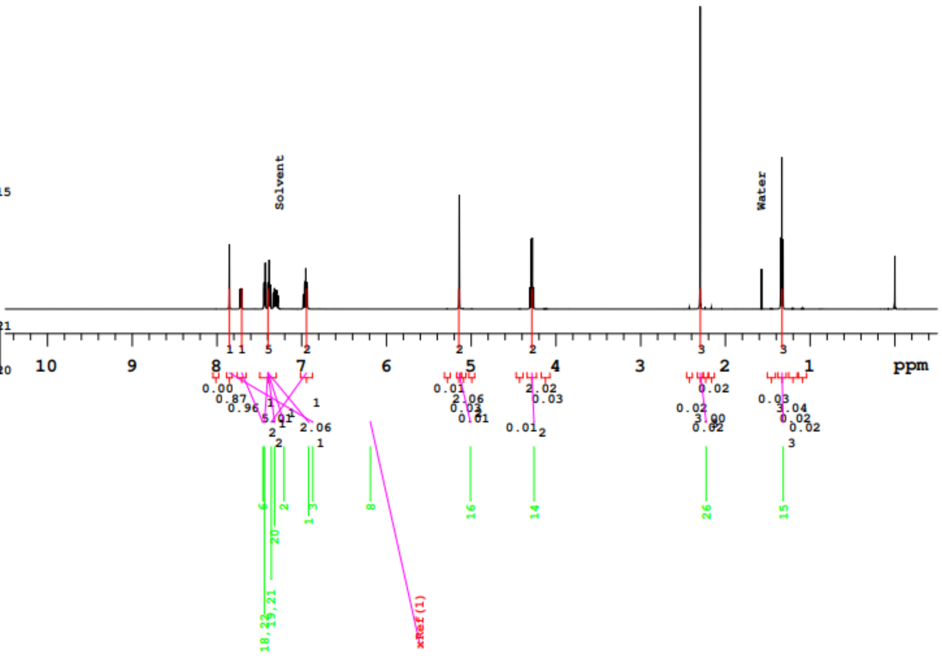

HoseCodePredictions

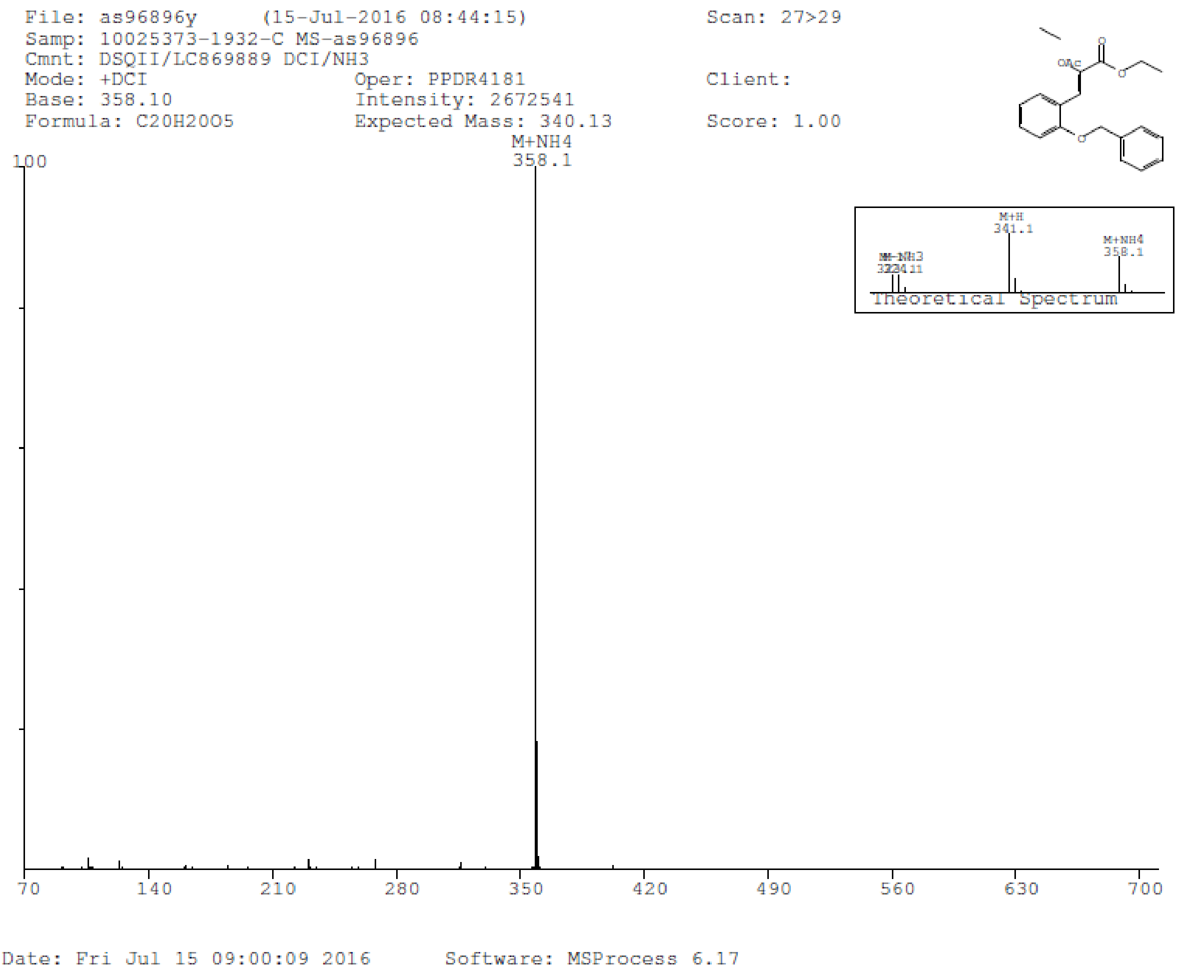

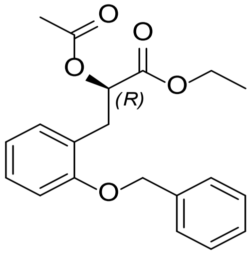

(R)-ethyl 2-acetoxy-3-(2-(benzyloxy)phenyl)propanoate (S-9).

abbvie

15006350-372-1 in CDCL3 $19 BC1008 4.28mg
Temp = 26 C
C20H22O5
jr400

Acq: VnmrJ VERSION 3.2 REVISION A/jr400
Proc: VnmrJ VERSION 3.2 REVISION A/coffee

Chemist: GREG STORER
Experiment: s2pul

nmr3339599

Jul 19 2016

Expected protons=22
Whole protons observed=19
Total integral =19.849

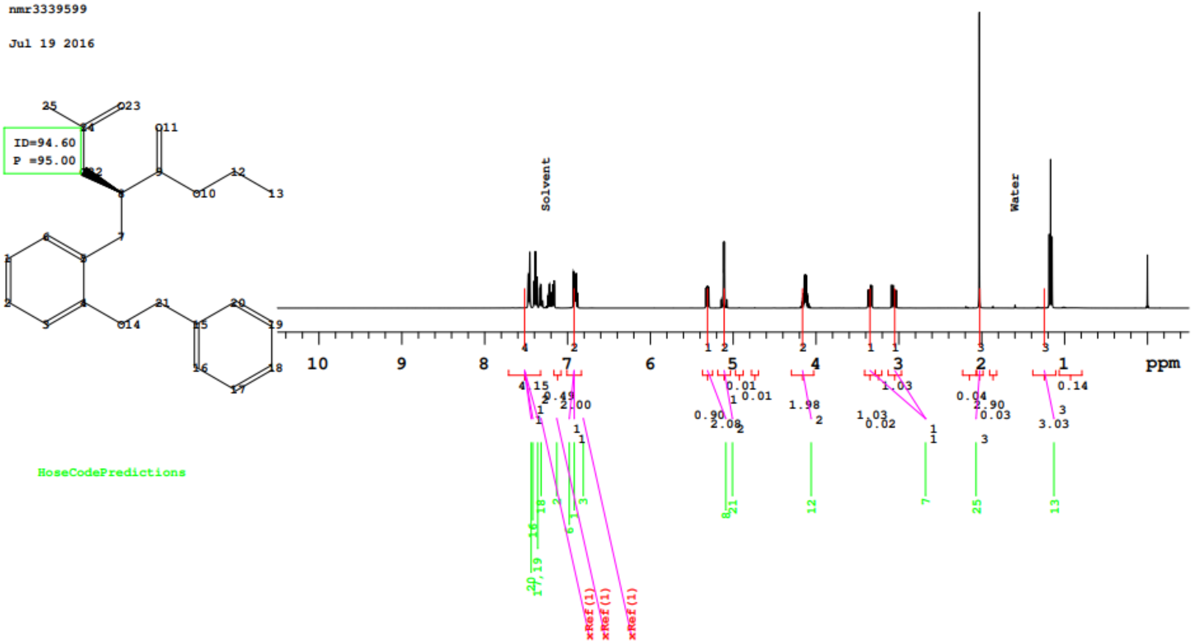

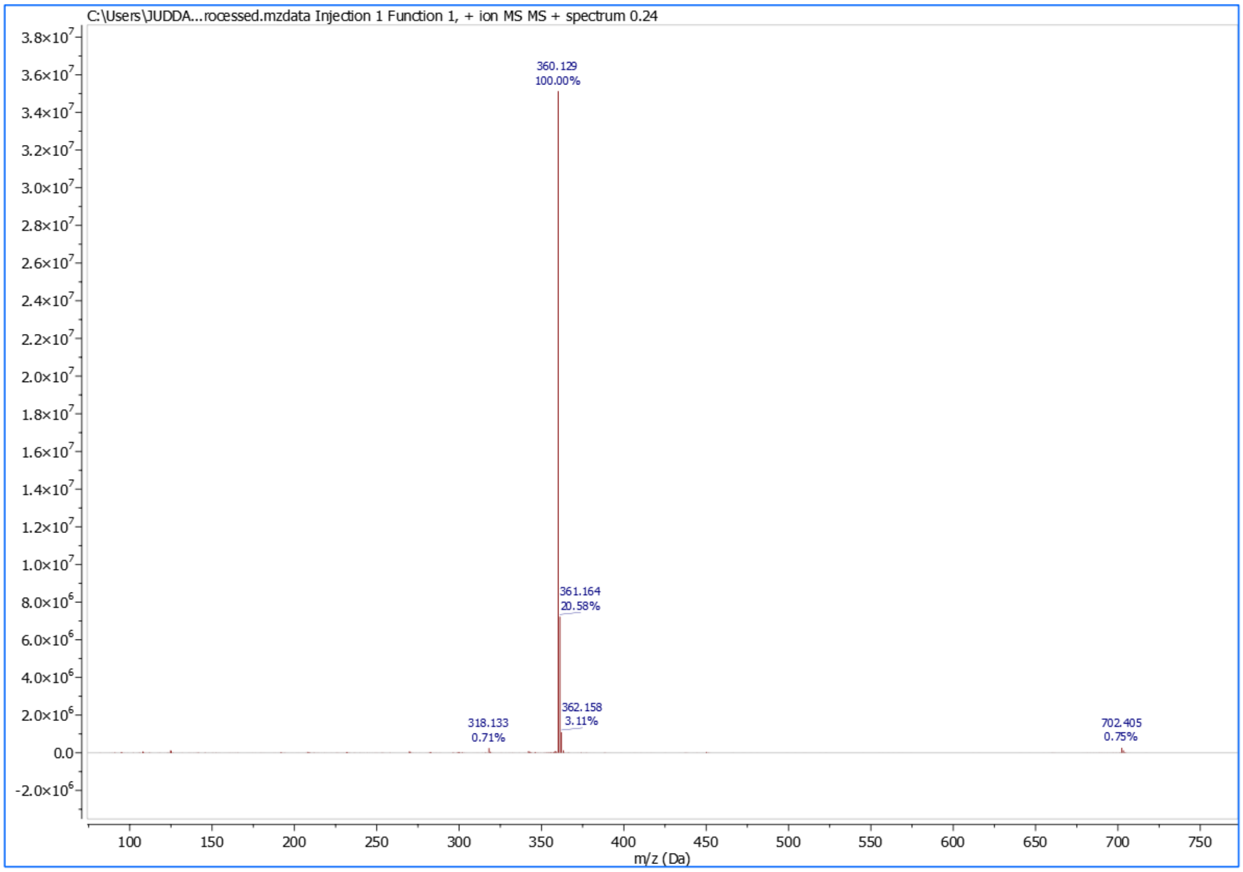

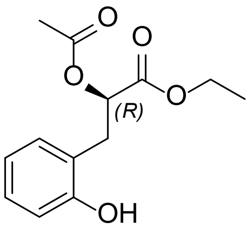

(R)-ethyl 2-acetoxy-3-(2-hydroxyphenyl)propanoate (S-10).

abbvie

10033550-3285-FINAL in CDCL3 BC#2433 $25
mrs400

Acq: VnmrJ VERSION 3.2 REVISION A/mrs400
Proc: VnmrJ VERSION 3.2 REVISION A/i600

Chemist: MATTHEW HANSEN
Solvent and water peaks subtracted
Experiment: s2pul

nmr3341189

Jul 25 2016

Expected protons=16
Whole protons observed=16
Total integral =16.369

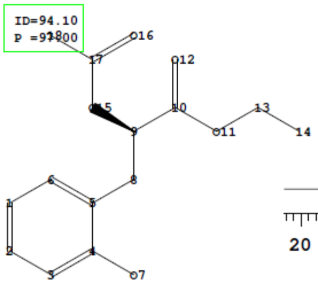

HoseCodePredictions

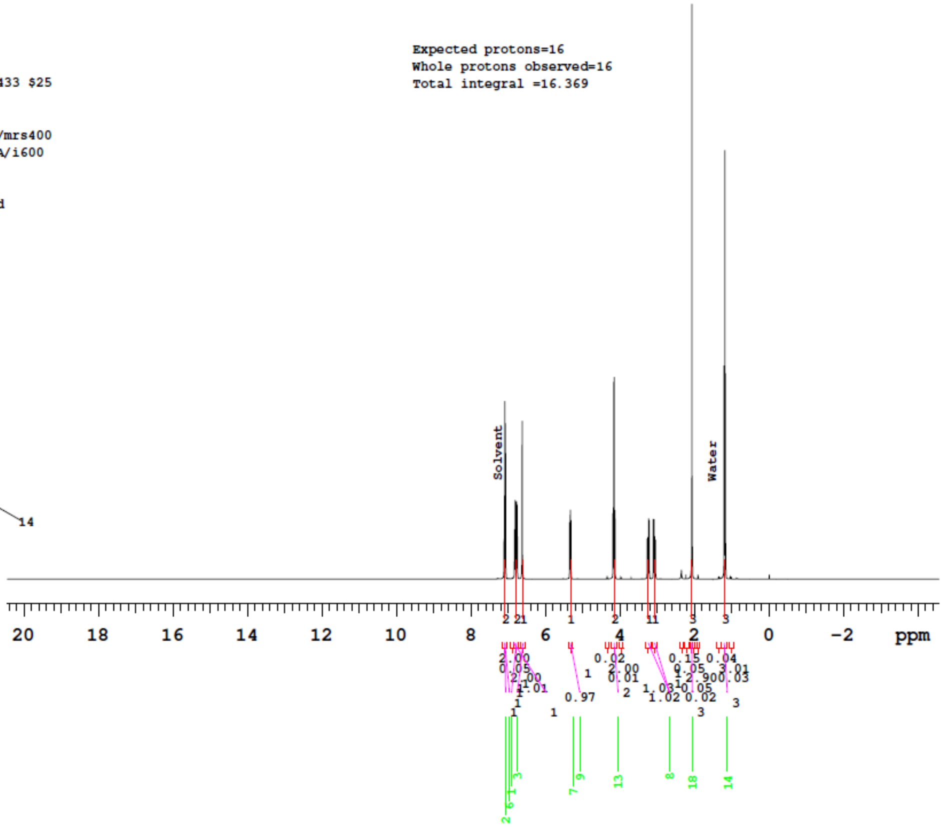

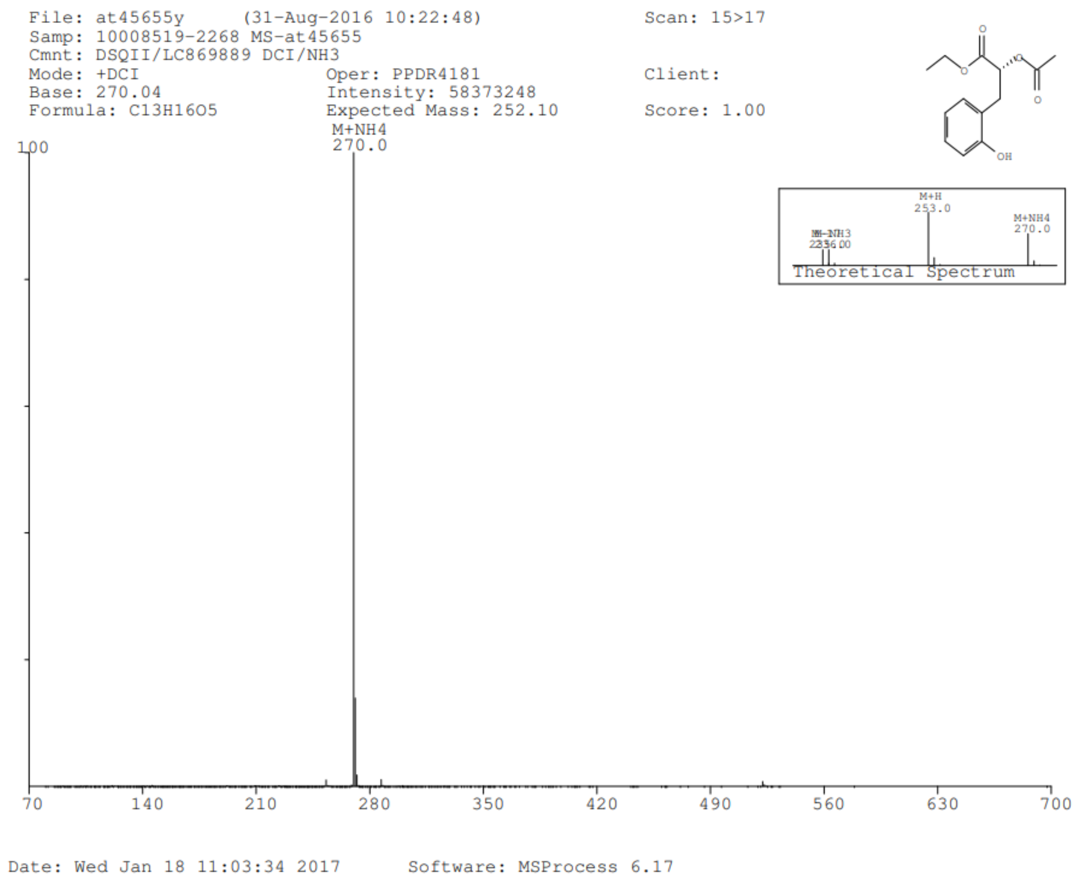

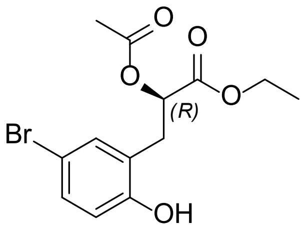

(R)-ethyl 2-acetoxy-3-(5-bromo-2-hydroxyphenyl)propanoate (S-11).

abbvie

10008519-2284-FIRSTDCMWASH in DMSO $01 BC400 5mg
Temp = 27 C
C13H15O5Br1
v501

Acq: VnmrJ VERSION 3.2 REVISION A/v501
Proc: VnmrJ VERSION 3.2 REVISION A/coffee

Chemist: BRYAN SORENSEN
Experiment: s2pul

nmr3353178

Sep 1 2016

Expected protons=15
Whole protons observed=15
Total integral =15.147

ID=96.50
P =97.00

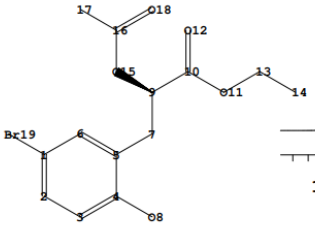

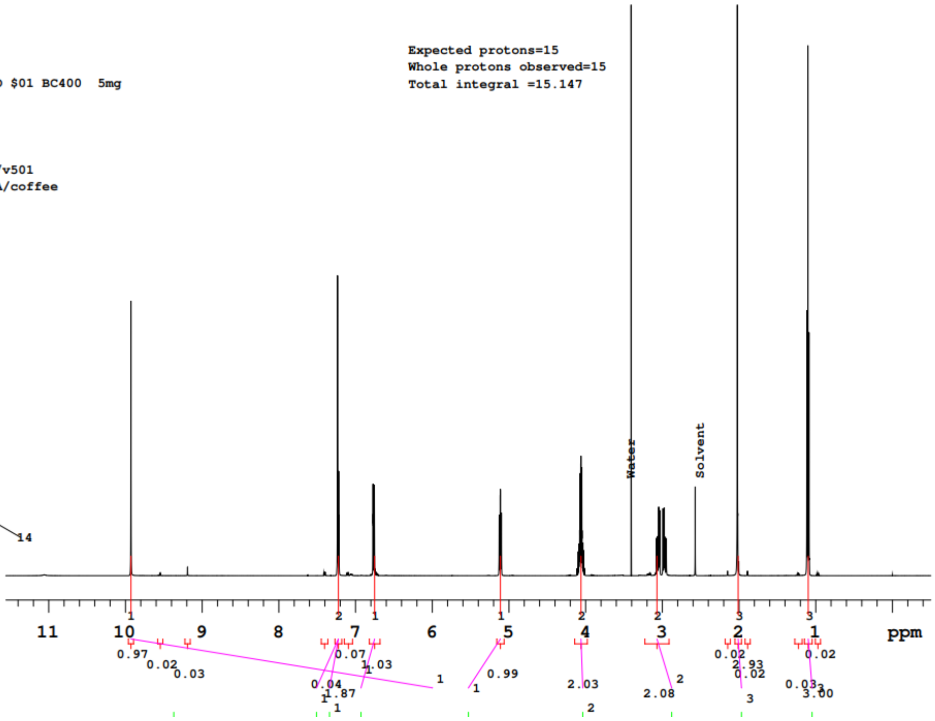

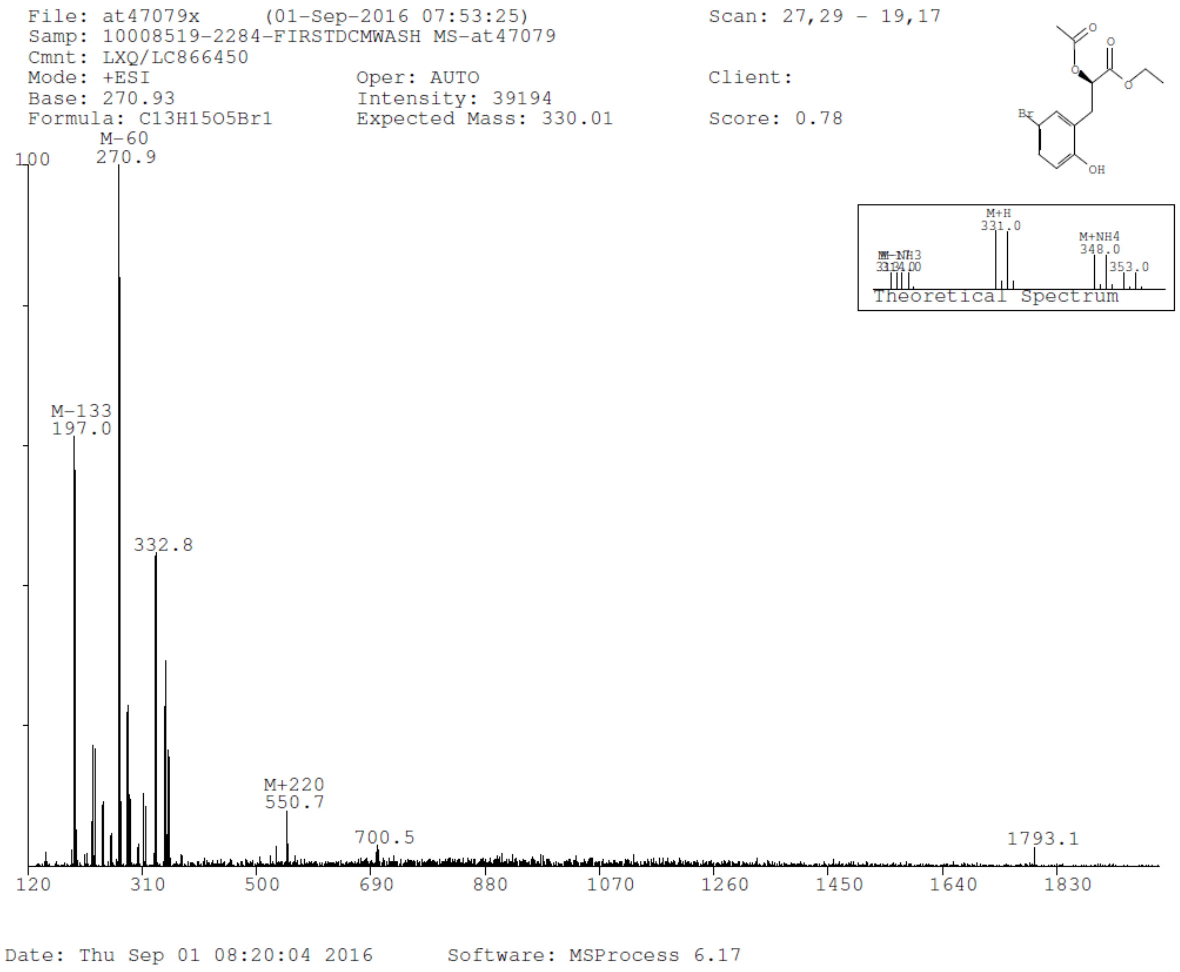

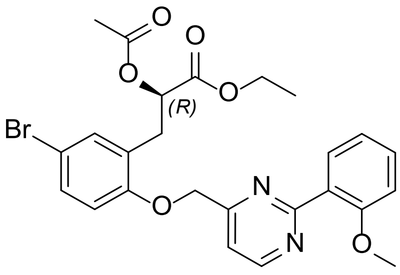

(R)-ethyl 2-acetoxy-3-(5-bromo-2-((2-(2-methoxyphenyl)pyrimidin-4-yl)methoxy)phenyl)propanoate (S-12).

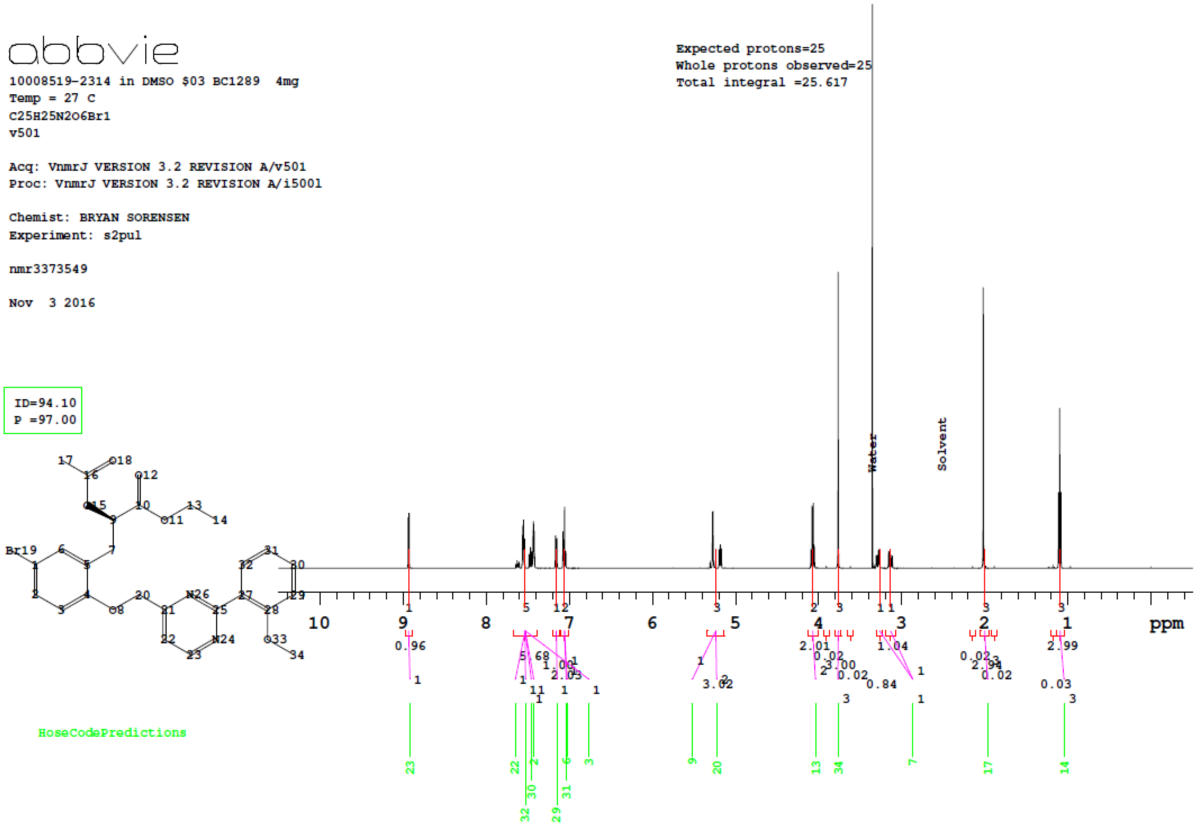
abbvie
10008519-2314 in DMSO $03 BC1289 4mg
Temp = 27 C
C25H25N2O6Br1
v501
Acq: VnmrJ VERSION 3.2 REVISION A/v501
Proc: VnmrJ VERSION 3.2 REVISION A/i5001
Chemist: BRYAN SORENSEN
Experiment: s2pul
nmr3373549
Nov 3 2016
Expected protons=25
Whole protons observed=25
Total integral =25.617
ID=94.10
P =97.00
Water
Solvent
HoseCodePredictions
ppm

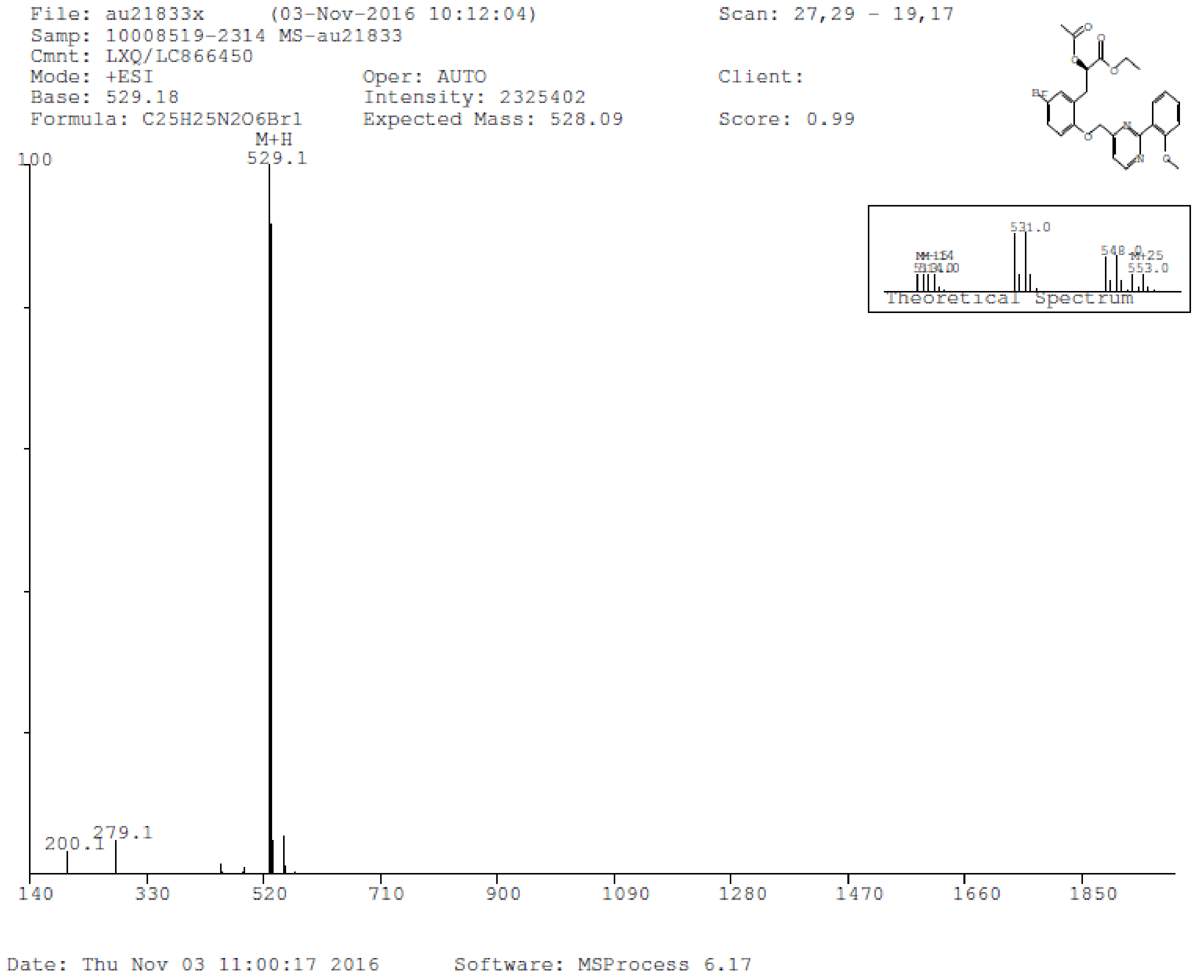

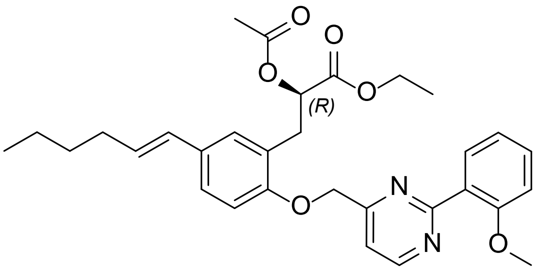

(R,E)-ethyl 2-acetoxy-3-(5-(hex-1-en-1-yl)-2-((2-(2-methoxyphenyl)pyrimidin-4-yl)methoxy)phenyl)propanoate (S-13).

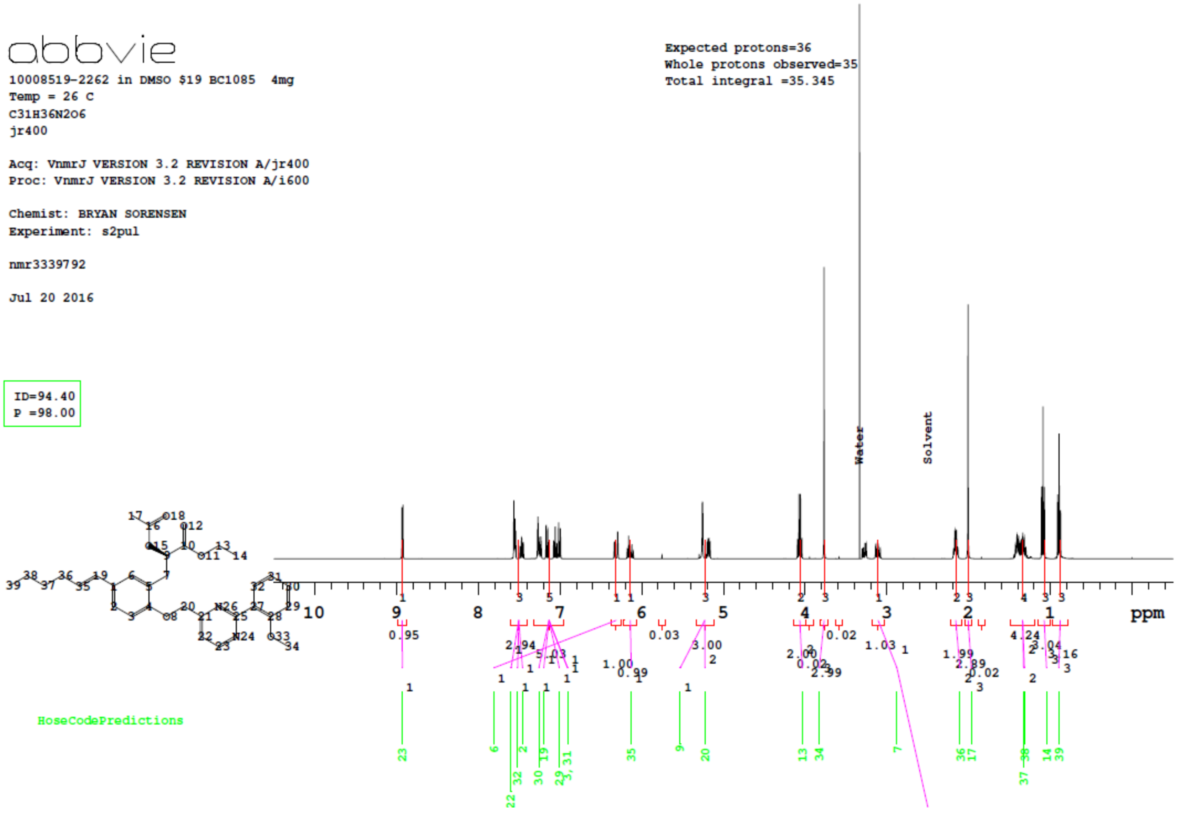
abbvie
10008519-2262 in DMSO $19 BC1085 4mg
Temp = 26 C
C31H36N2O6
jr400
Acq: VnmrJ VERSION 3.2 REVISION A/jr400
Proc: VnmrJ VERSION 3.2 REVISION A/i600
Chemist: BRYAN SORENSEN
Experiment: s2pul
nmr3339792
Jul 20 2016
ID=94.40
P =98.00
HoseCodePredictions
Expected protons=36
Whole protons observed=35
Total integral =35.345
Water
Solvent
ppm

File: at00570x (20-Jul-2016 06:04:12) Scan: 27,29 - 11,9
Samp: 10008519-2262 MS-at00570
Cmnt: LXQ/LC866450
Mode: +ESI Oper: AUTO Client:
Base: 533.35 Intensity: 4754555
Formula: C31H36N2O6 Expected Mass: 532.26 Score: 1.00

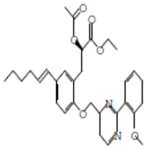

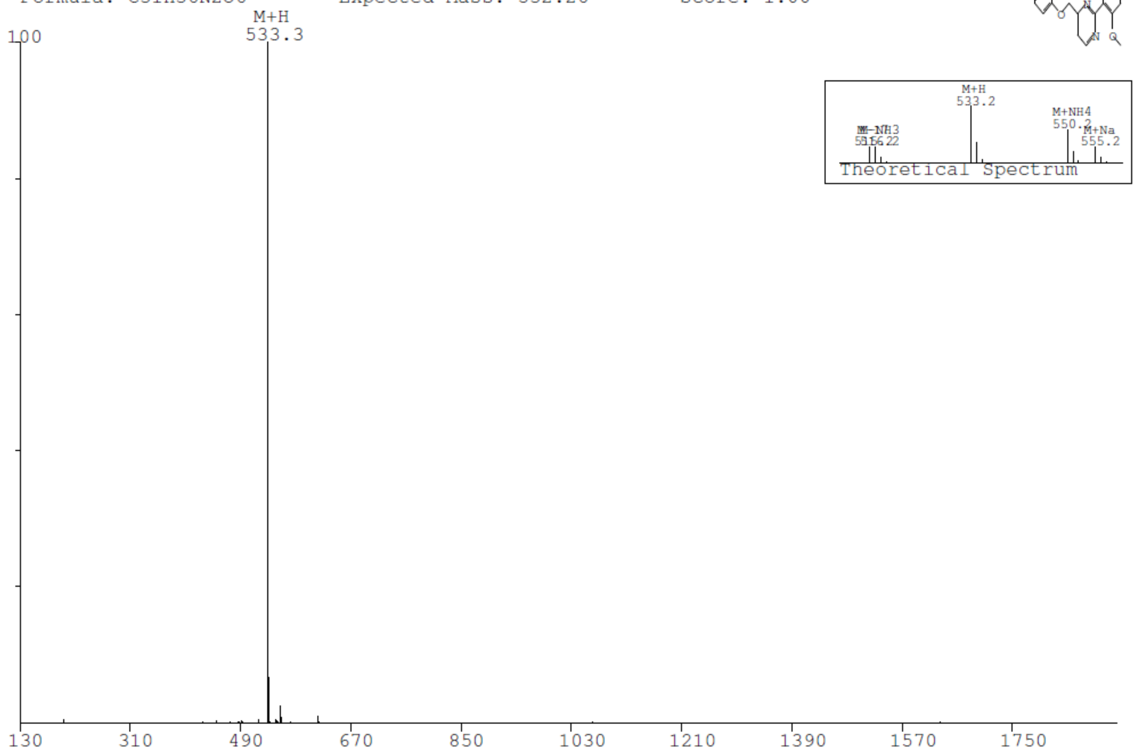

Date: Wed Jul 20 06:20:26 2016 Software: MSProcess 6.17

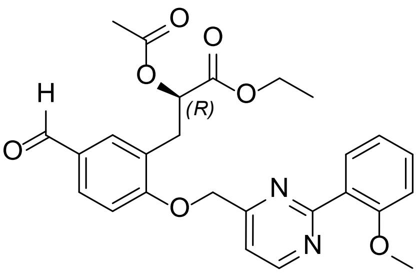

(R)-ethyl 2-acetoxy-3-(5-formyl-2-((2-(2-methoxyphenyl)pyrimidin-4-yl)methoxy)phenyl)propanoate (S-14).

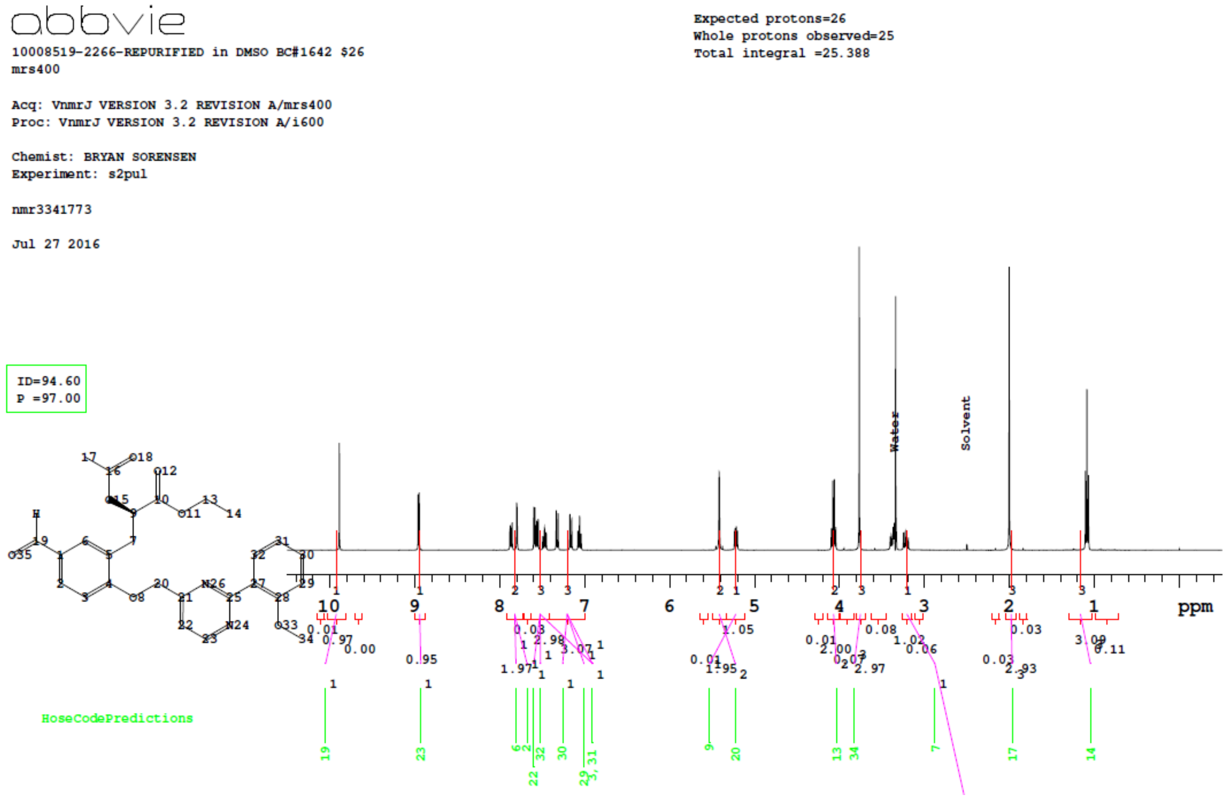

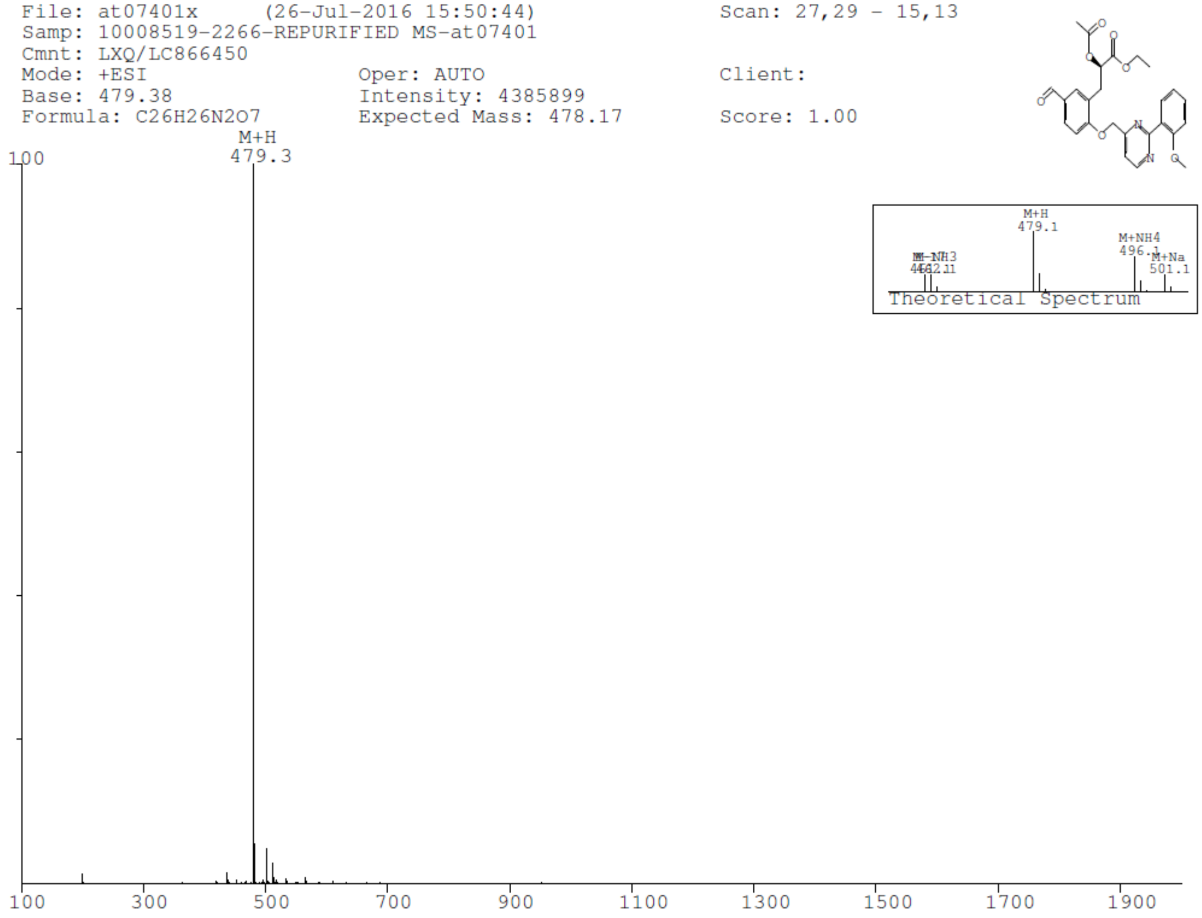

Date: Wed Jul 27 05:34:32 2016 Software: MSProcess 6.17

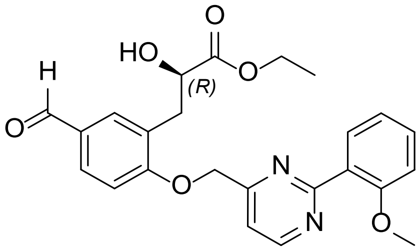

(R)-ethyl 3-(5-formyl-2-((2-(2-methoxyphenyl)pyrimidin-4-yl)methoxy)phenyl)-2-hydroxypropanoate (S-15).

abbvie

10008519-2221 in DMSO BC#146 $19
mrs400

Acq: VnmrJ VERSION 3.2 REVISION A/mrs400
Proc: VnmrJ VERSION 3.2 REVISION A/coffee

Chemist: BRYAN SORENSEN
Experiment: s2pul

nmr3315576

May 19 2016

Expected protons=24
Whole protons observed=24
Total integral =24.708

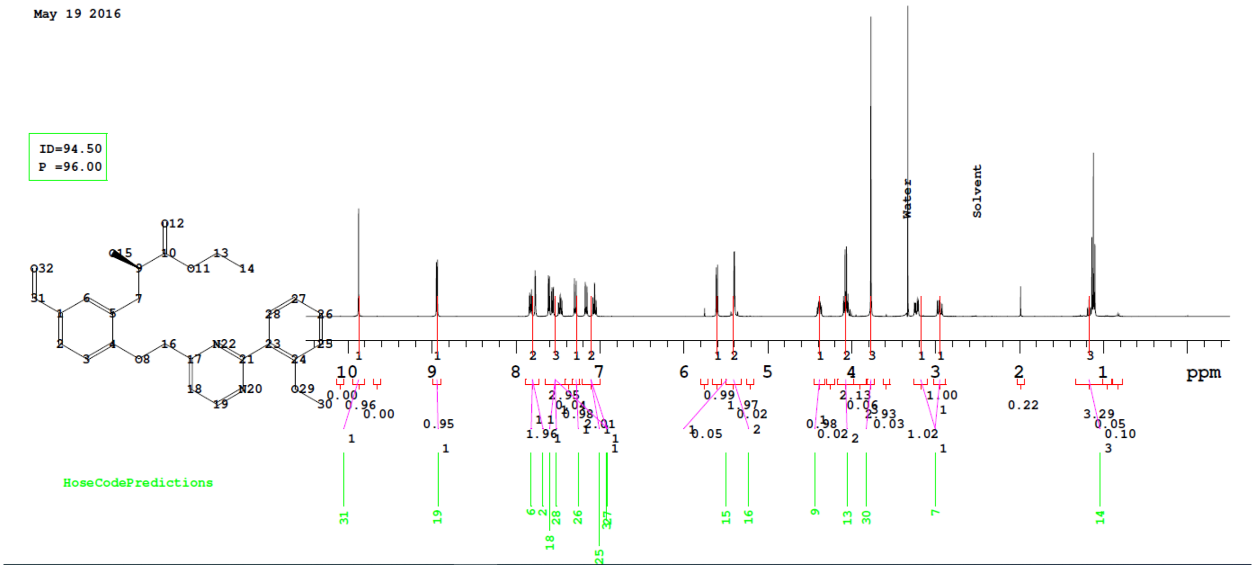

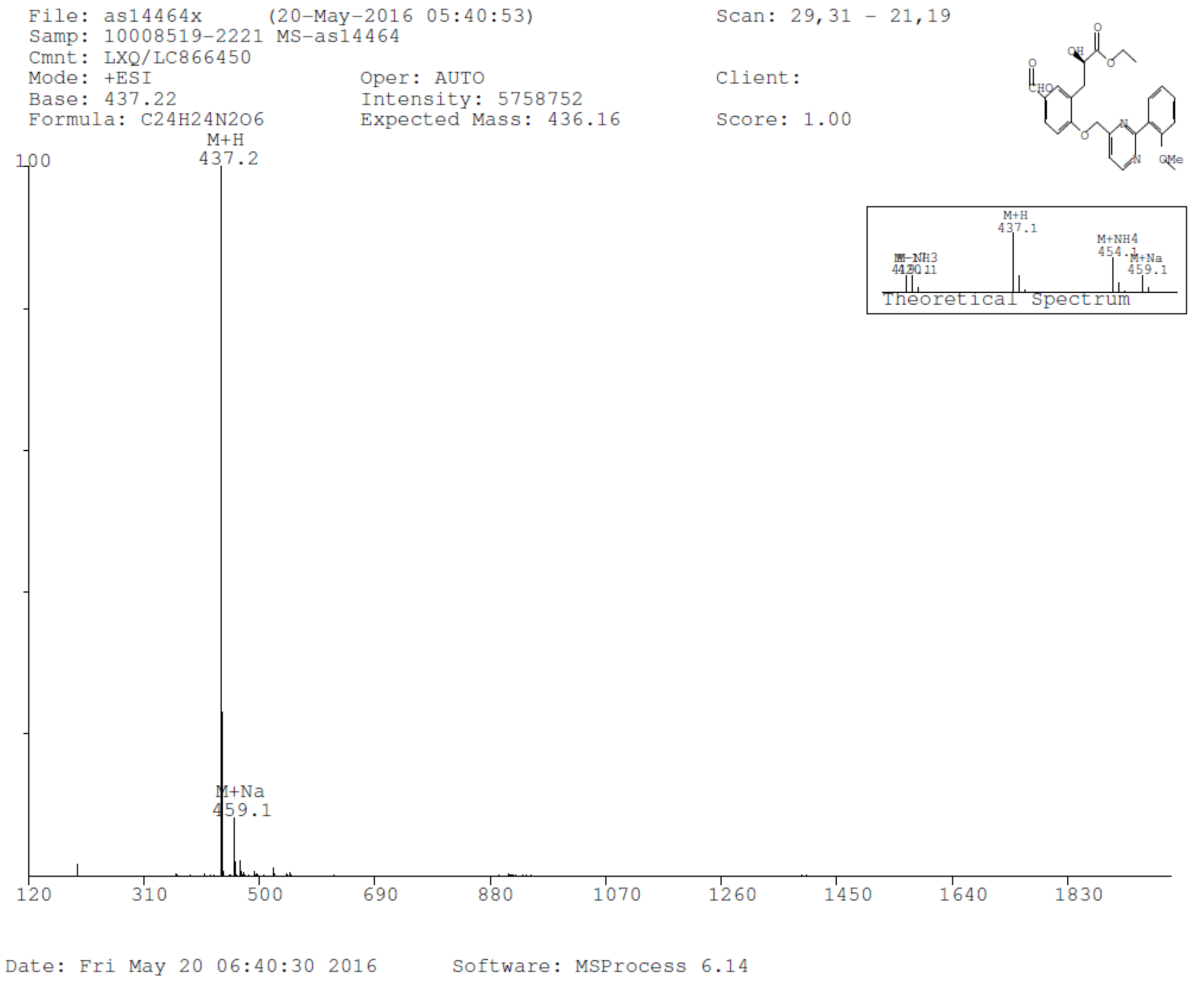

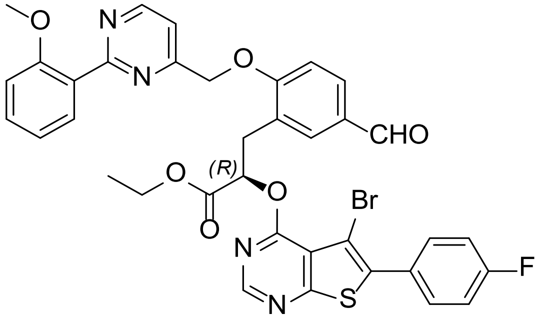

(R)-ethyl 2-((5-bromo-6-(4-fluorophenyl)thieno[2,3-d]pyrimidin-4-yl)oxy)-3-(5-formyl-2-((2-(2-methoxyphenyl)pyrimidin-4-yl)methoxy)phenyl)propanoate (S-16).

abbvie

10008519-2272 in DMSO $10 BC876 4mg
Temp = 26 C
C36H28N4O6F1S1Br1
jr400

Acq: VnmrJ VERSION 3.2 REVISION A/jr400
Proc: VnmrJ VERSION 3.2 REVISION A/i600

Chemist: BRYAN SORENSEN
Experiment: s2pul

nmr3346293

Aug 10 2016

Expected protons=28
Whole protons observed=27
Total integral =27.090

ID=95.60
P =98.00

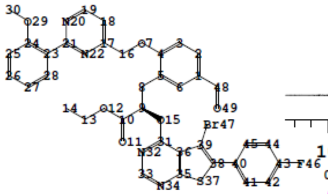

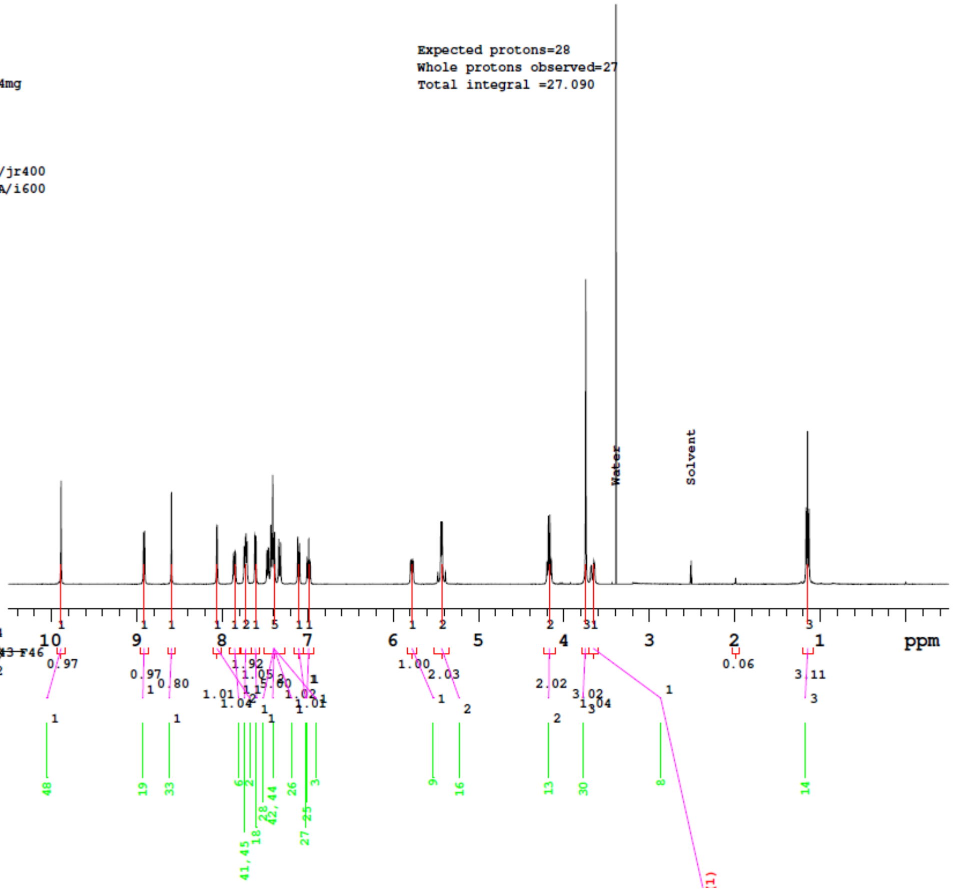

HoseCodePredictions

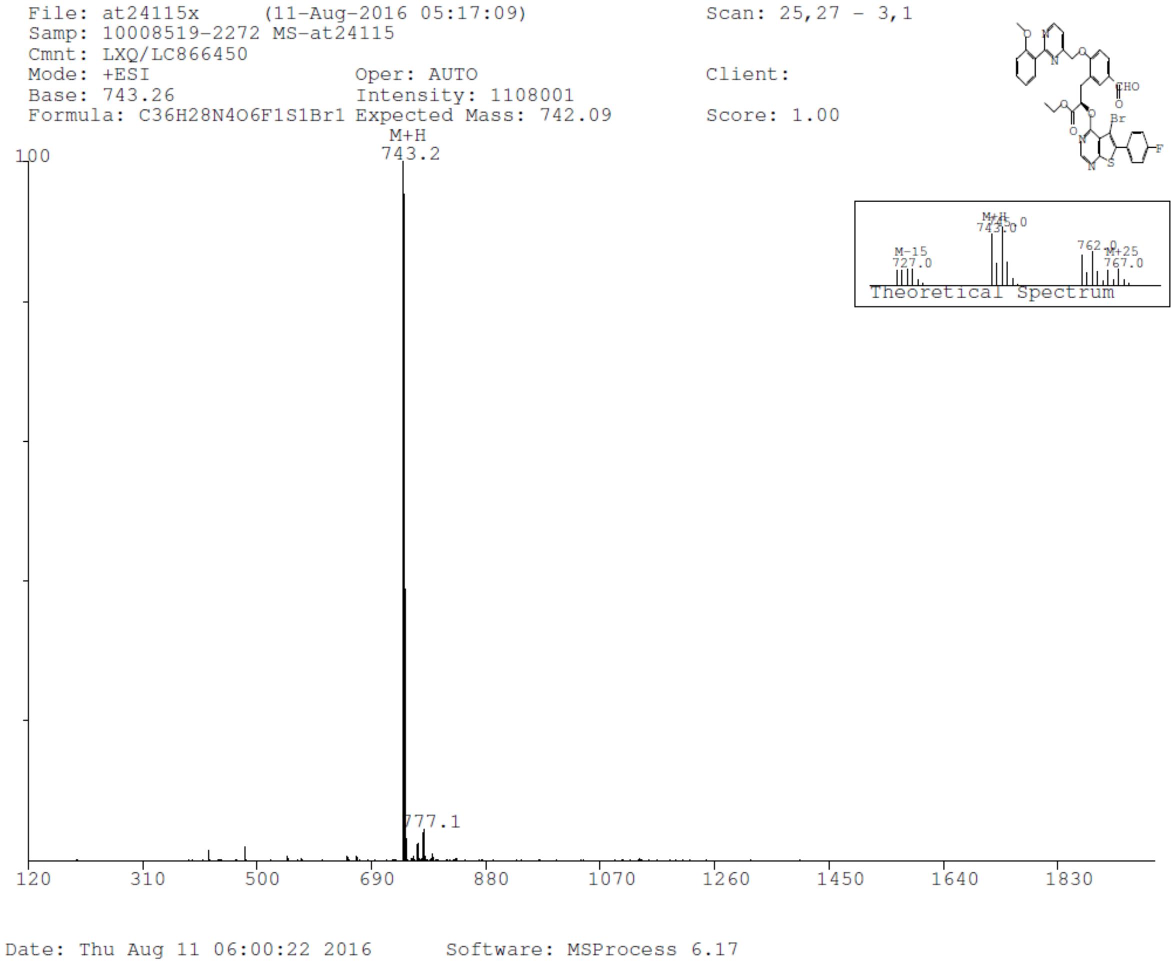

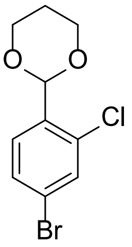

2-(4-bromo-2-chlorophenyl)-1,3-dioxane (S-17).

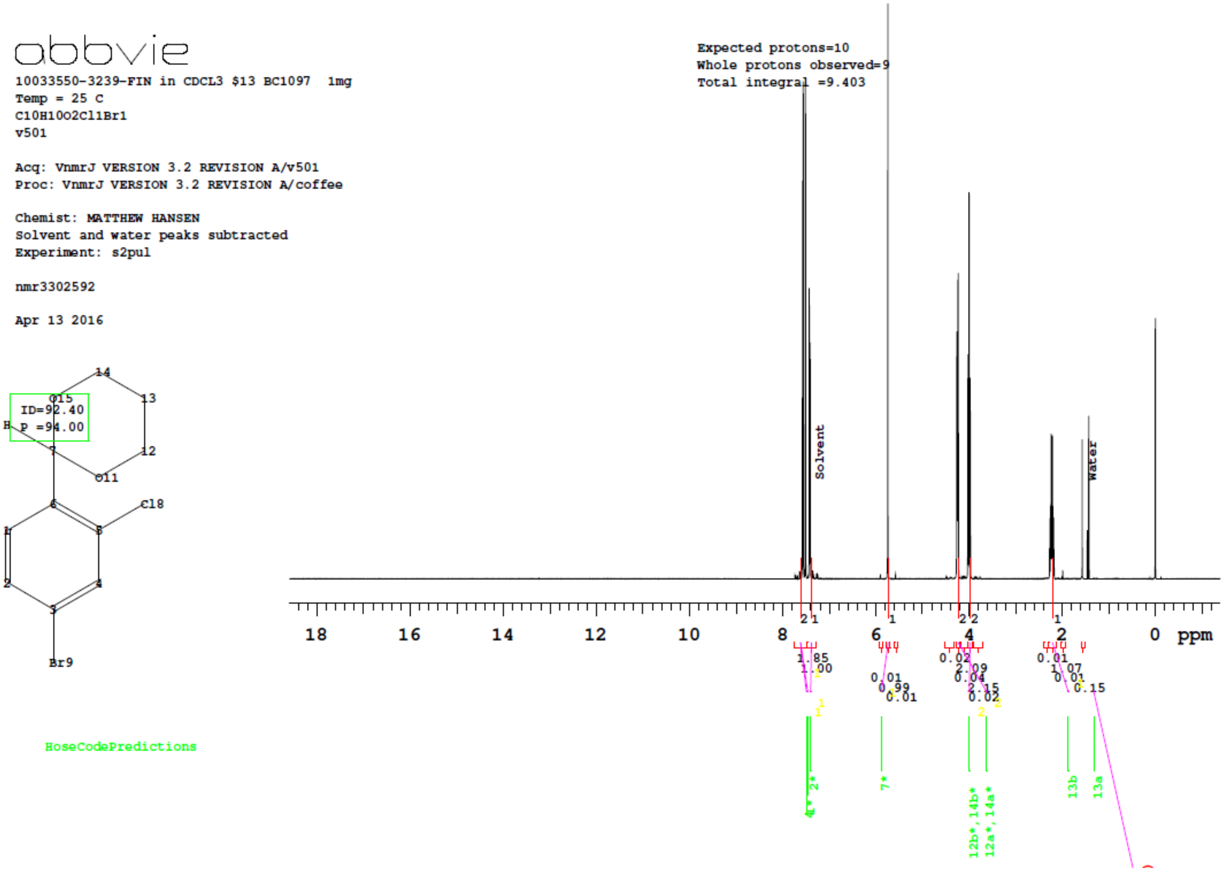

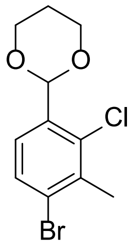

2-(4-bromo-2-chloro-3-methylphenyl)-1,3-dioxane (S-18).

abbvie

10033550-3284-FINAL in CDCL3 BC#2429 $21
mrs400

Acq: VnmrJ VERSION 3.2 REVISION A/mrs400
Proc: VnmrJ VERSION 3.2 REVISION A/i600

Chemist: MATTHEW HANSEN
Solvent and water peaks subtracted
Experiment: s2pul

nmr3340442

Jul 21 2016

Expected protons=12
Whole protons observed=12
Total integral =12.161

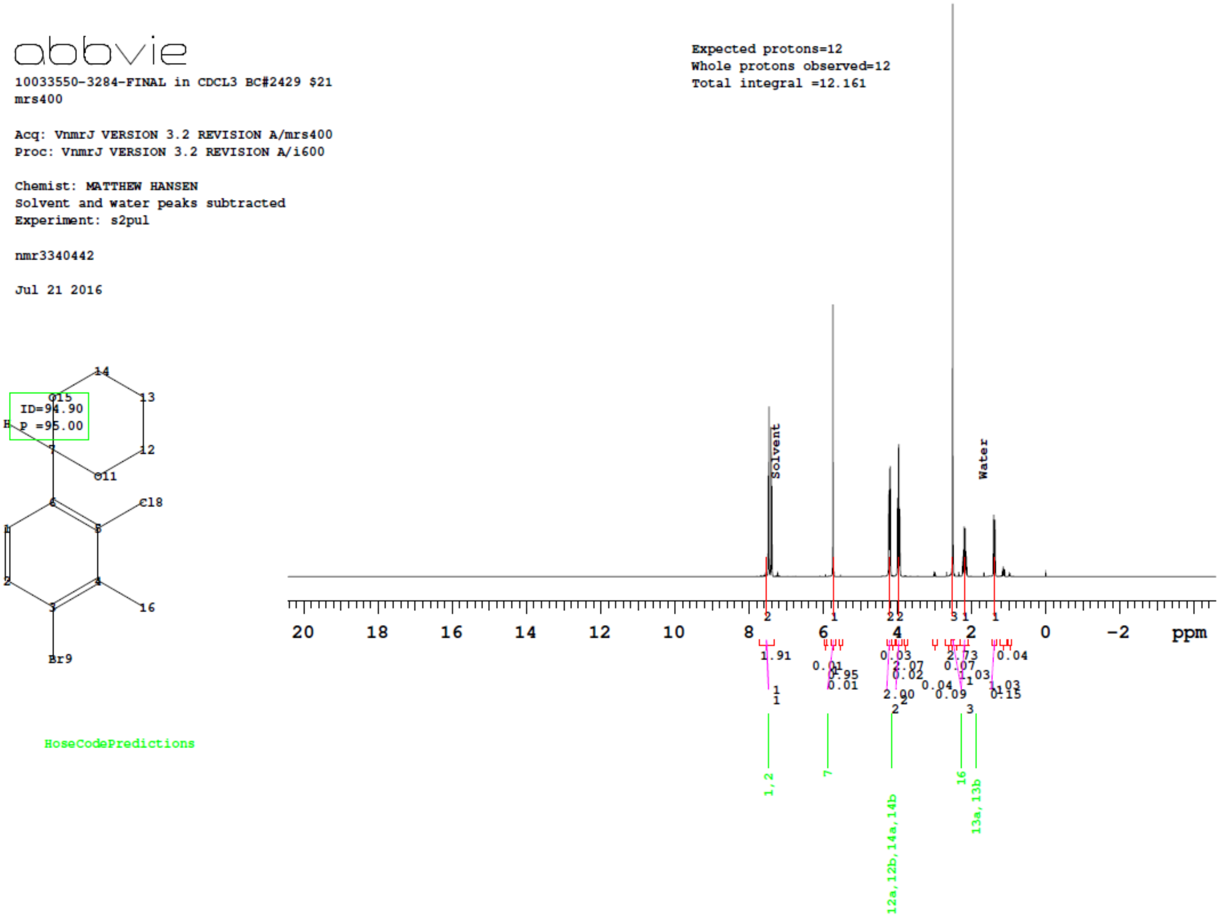

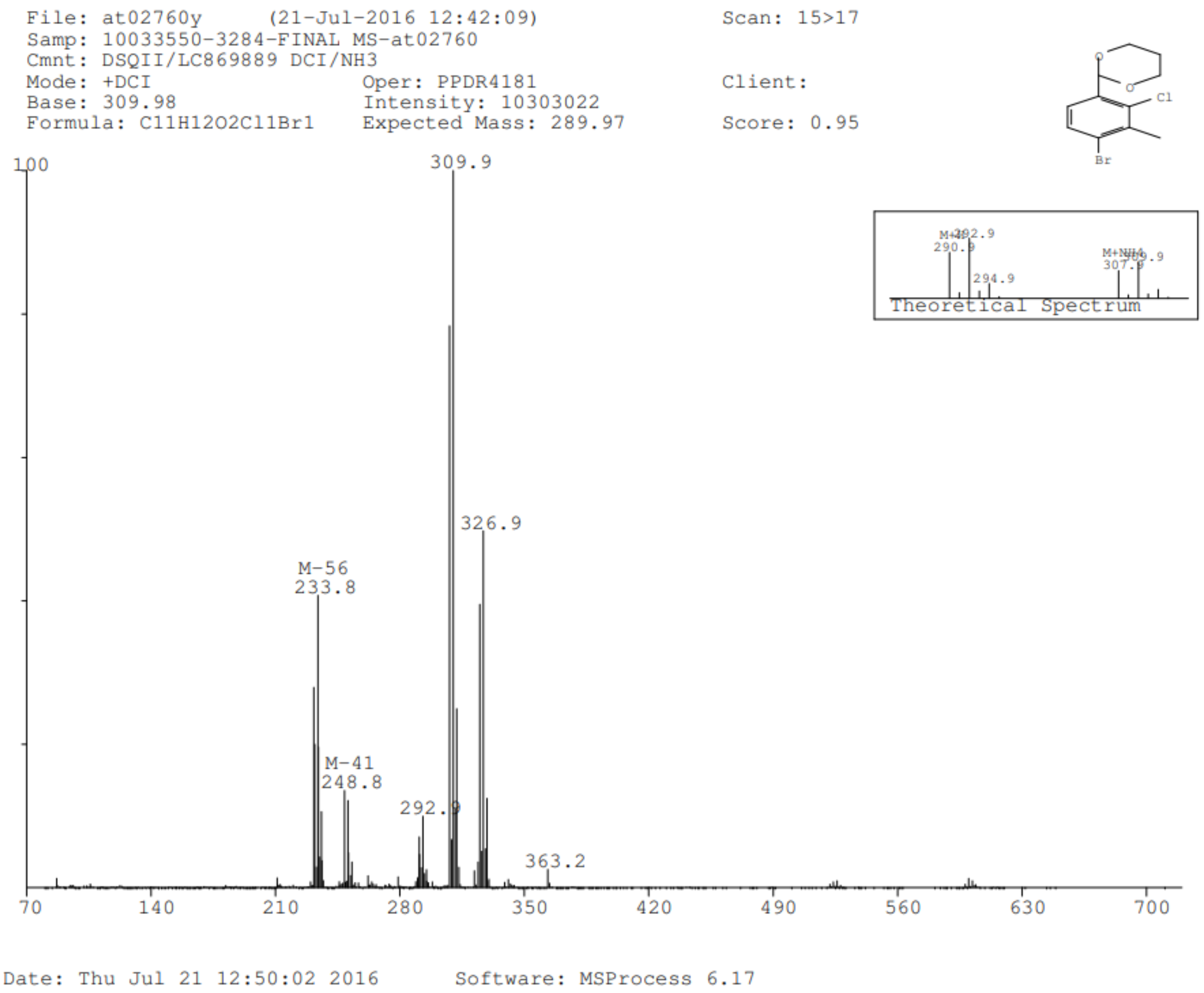

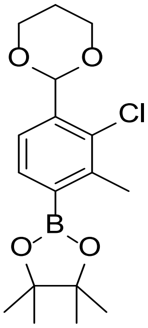

2-(3-chloro-4-(1,3-dioxan-2-yl)-2-methylphenyl)-4,4,5,5-tetramethyl-1,3,2-dioxaborolane (S-19).

10033550-3286-FIN-1 in CDCL3 $26 BC1615 1mg
Temp = 26 C
C17H24B1O4Cl1
jr400

Acq: VnmrJ VERSION 3.2 REVISION A/jr400
Proc: VnmrJ VERSION 3.2 REVISION A/i600

Chemist: MATTHEW HANSEN
Solvent and water peaks subtracted
Experiment: s2pul

nmr3341638

Jul 26 2016

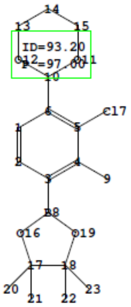

HoseCodePredictions

Expected protons=24
Whole protons observed=24
Total integral =24.113

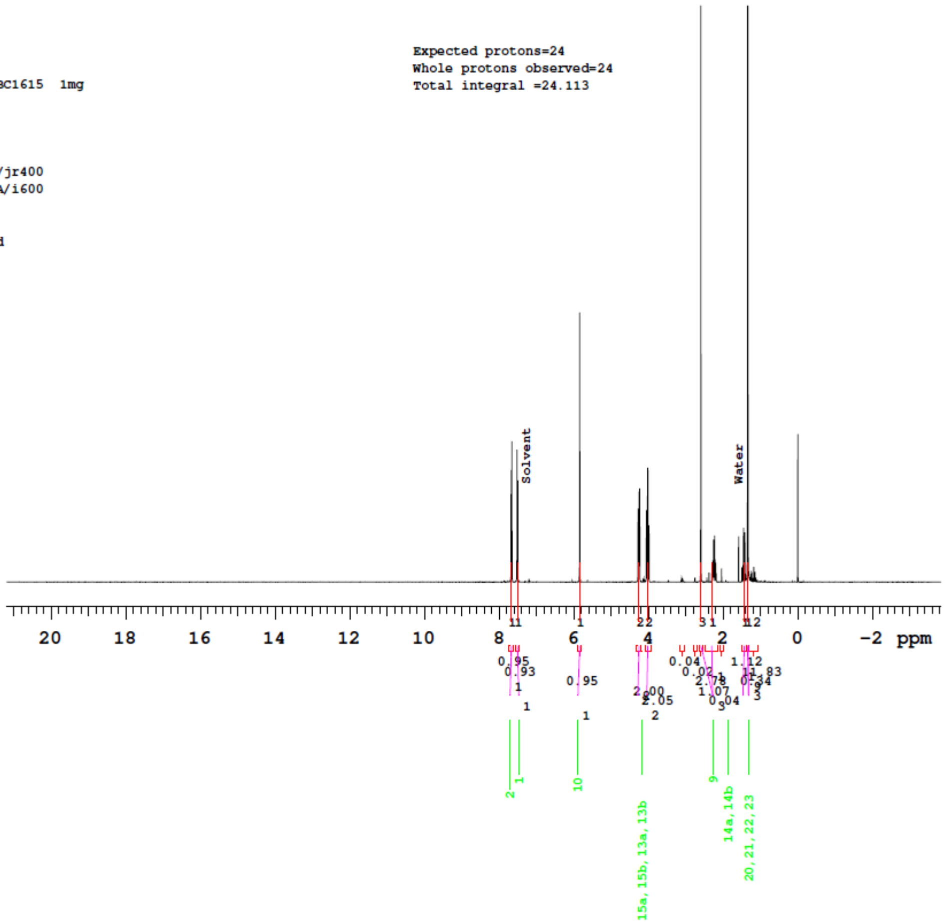

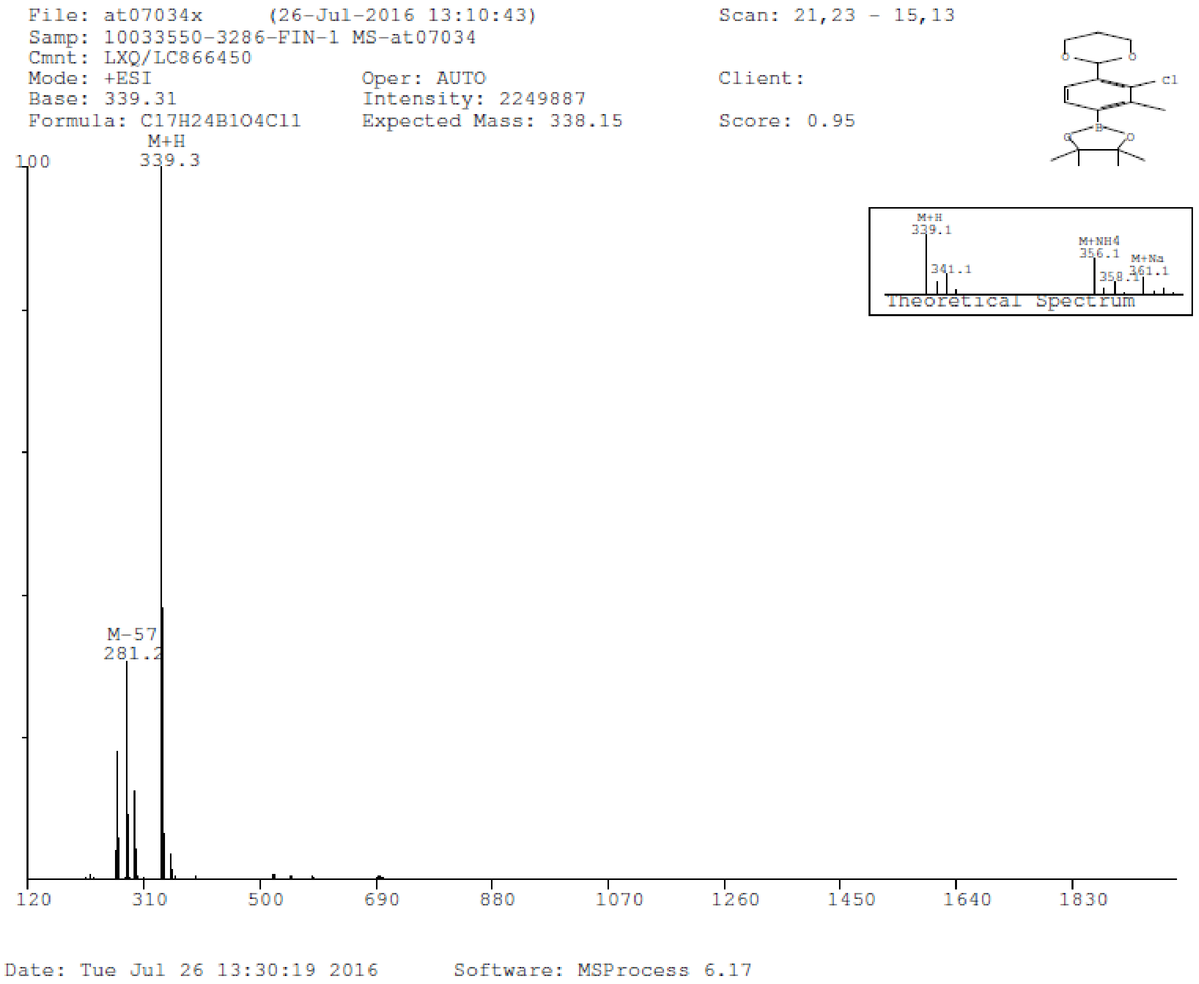

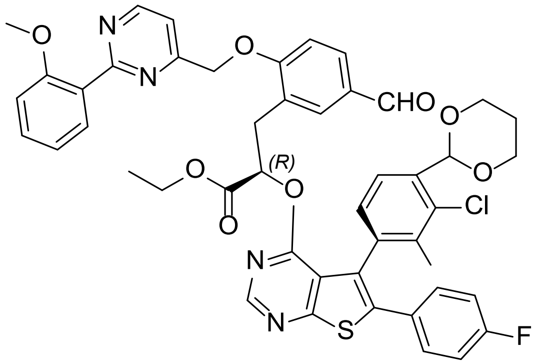

(R)-ethyl 2-((5-((1S)-3-chloro-4-(1,3-dioxan-2-yl)-2-methylphenyl)-6-(4-fluorophenyl)thieno[2,3-d]pyrimidin-4-yl)oxy)-3-(5-formyl-2-((2-(2-methoxyphenyl)pyrimidin-4-yl)methoxy)phenyl)propanoate (S-20).

abbvie

10008519-2224 in DMSO $25 BC534 4mg
Temp = 26 C
C47H40N4O8F1S1Cl1
jr400

Acq: VnmrJ VERSION 3.2 REVISION A/jr400
Proc: VnmrJ VERSION 3.2 REVISION A/i600

Chemist: BRYAN SORENSEN
Experiment: s2pul

nmr3317014

May 25 2016

Expected protons=40
Whole protons observed=41
Total integral =42.327

ID=88.50
P =92.00

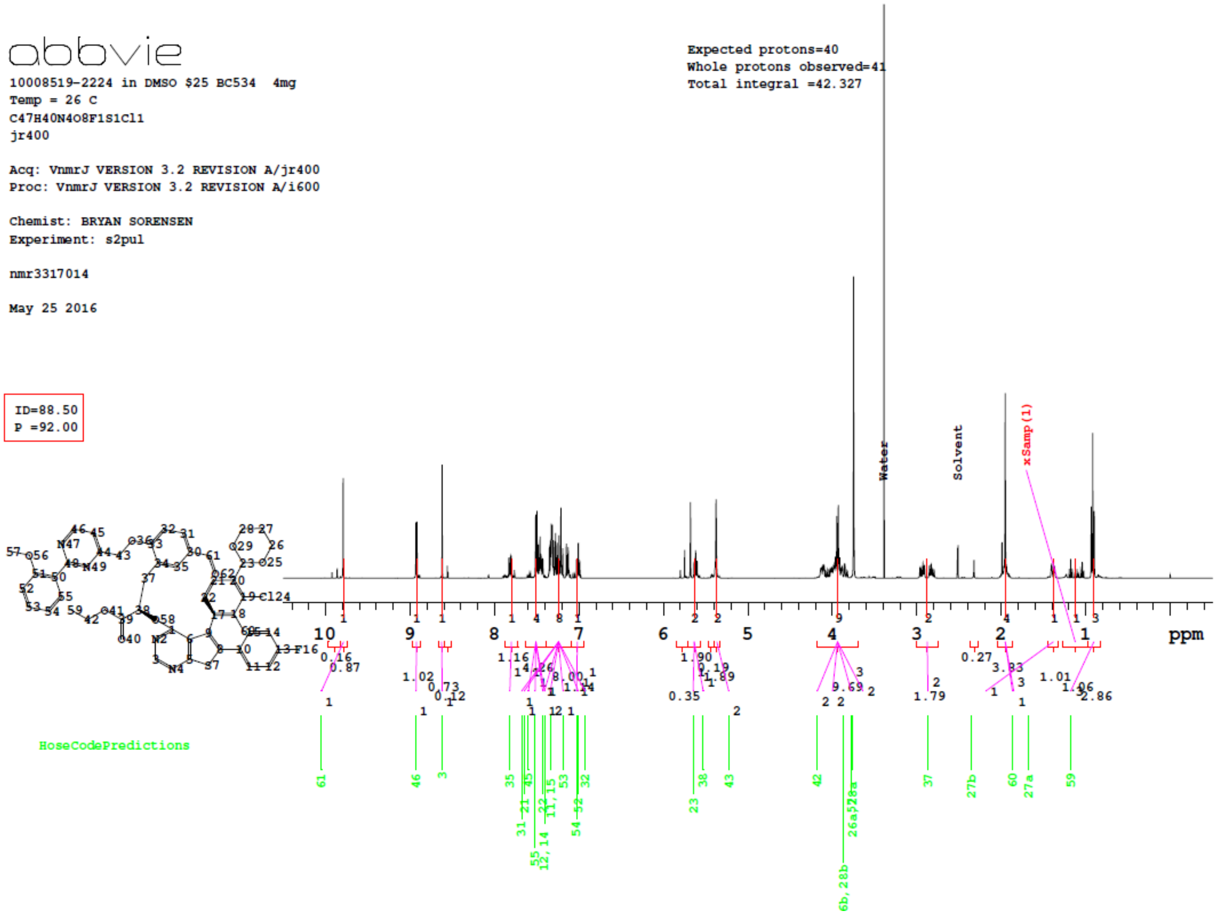

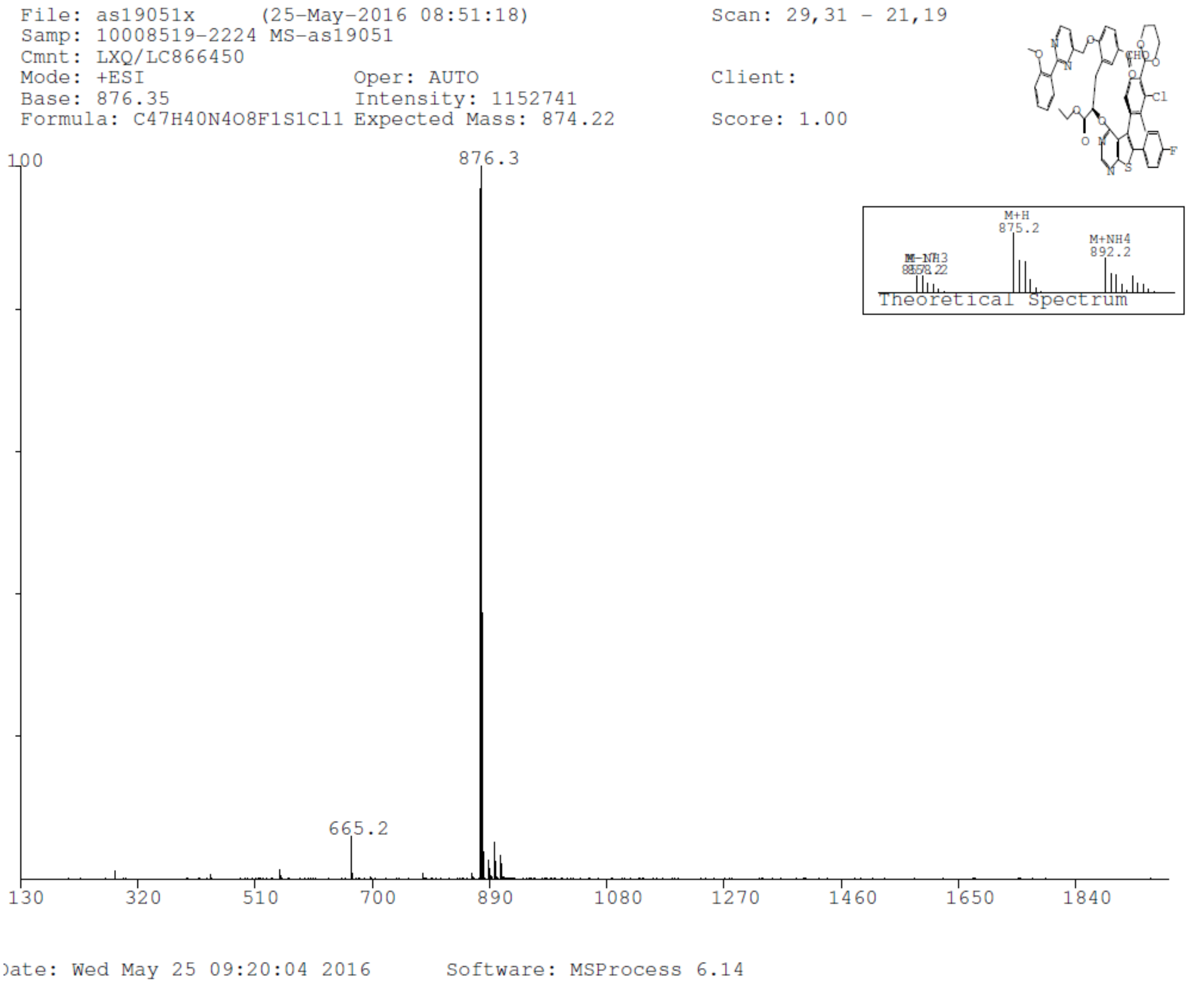

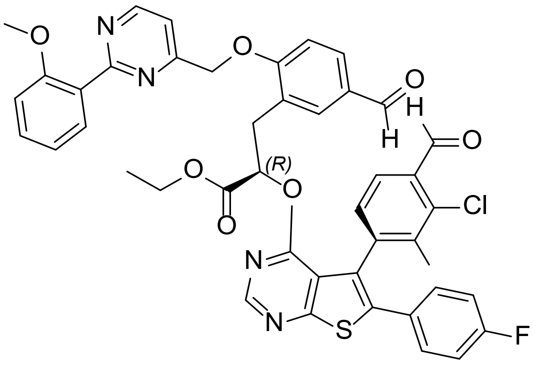

(R)-ethyl 2-((5-((1S)-3-chloro-4-formyl-2-methylphenyl)-6-(4-fluorophenyl)thieno[2,3-d]pyrimidin-4-yl)oxy)-3-(5-formyl-2-((2-(2-methoxyphenyl)pyrimidin-4-yl)methoxy)phenyl)propanoate (S-21).

abbvie

10008519-2212 in DMSO BC#1501 $12
mrs400

Acq: VnmrJ VERSION 3.2 REVISION A/mrs400
Proc: VnmrJ VERSION 3.2 REVISION A/ui5001

Chemist: BRYAN SORENSEN
Experiment: s2pul

nmr3312925

May 12 2016

Expected protons=34
Whole protons observed=36
Total integral =37.602

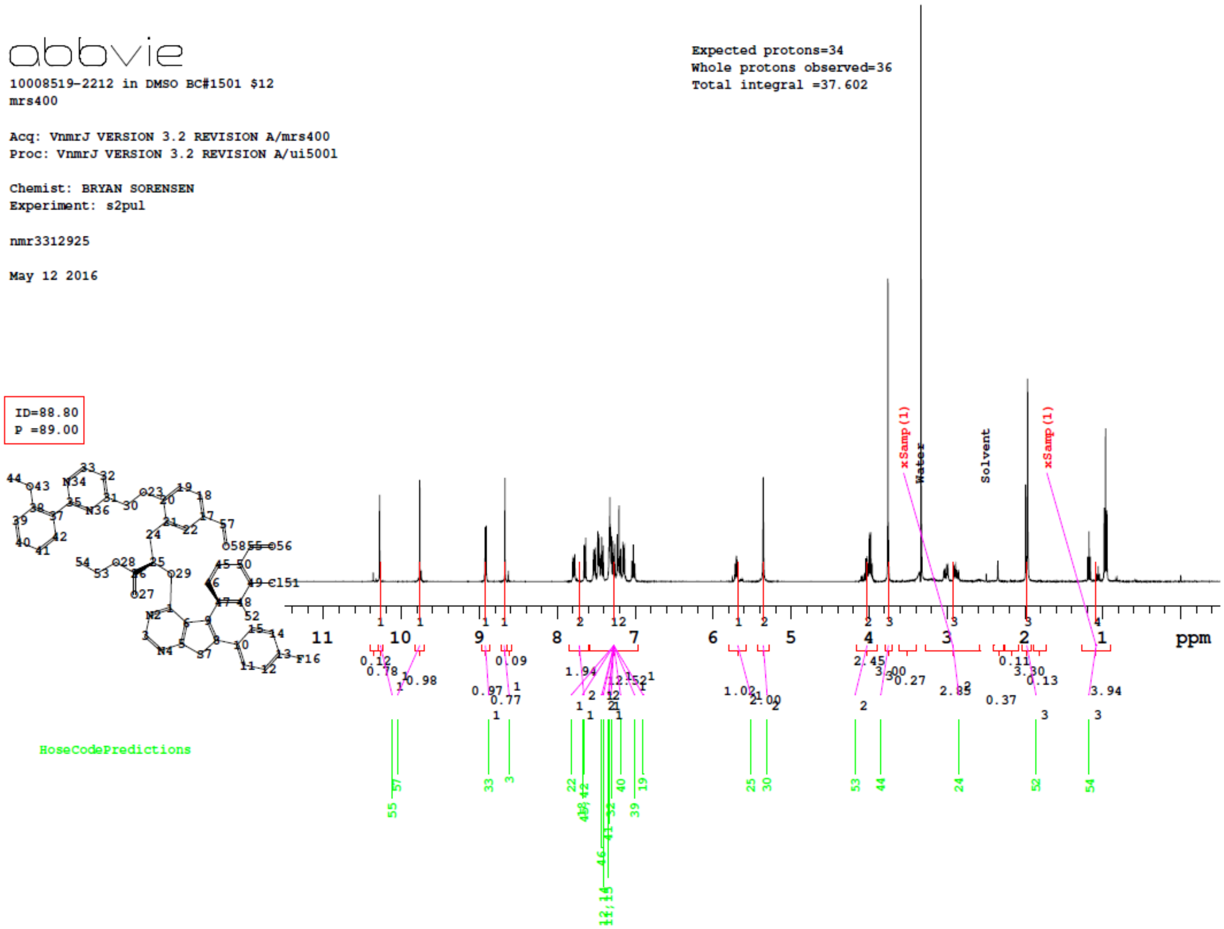

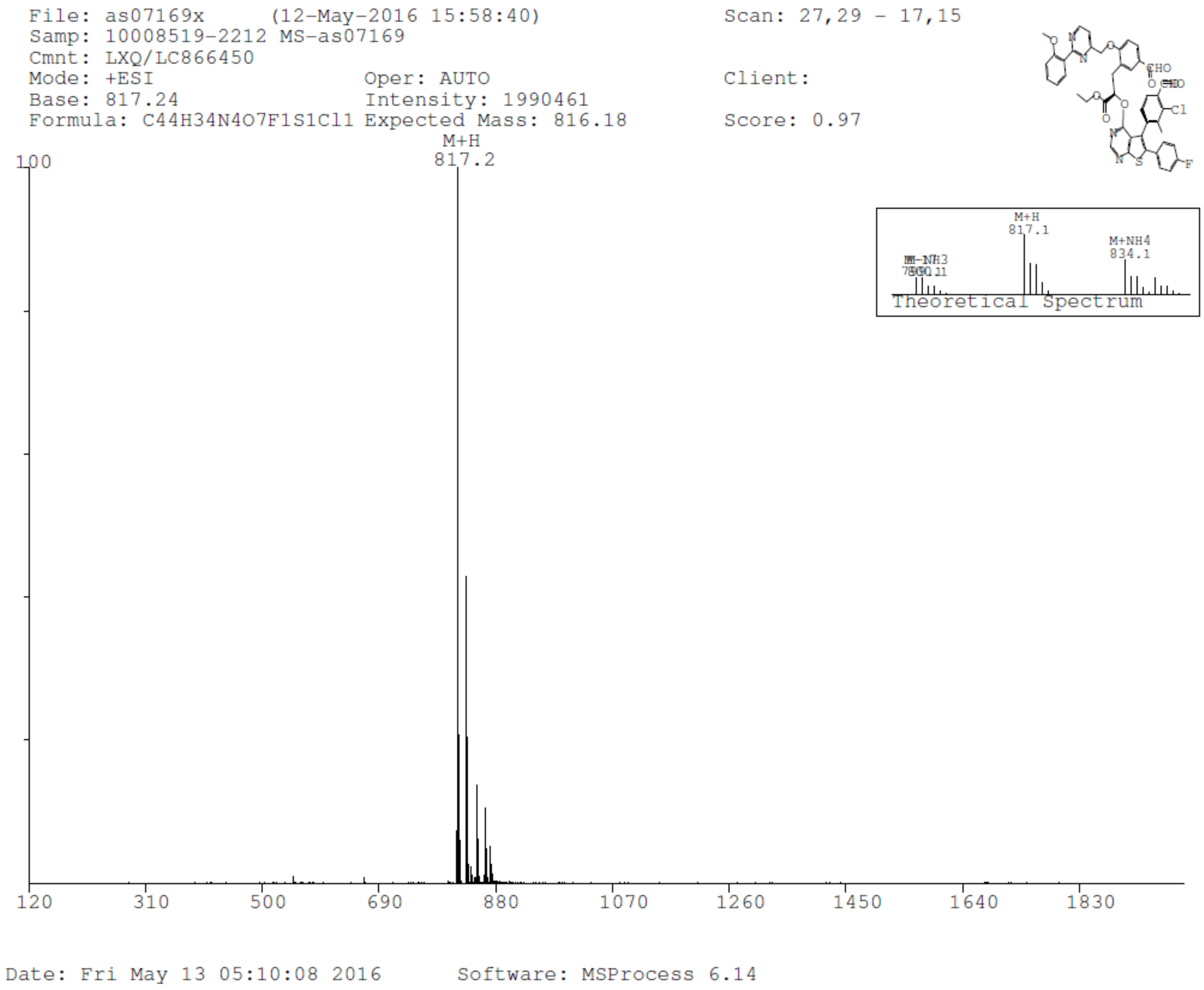

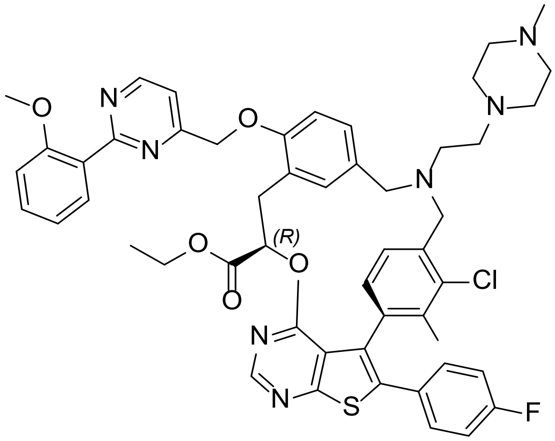

Ethyl (7R,20S)-18-chloro-1-(4-fluorophenyl)-10-{[2-(2-methoxyphenyl)pyrimidin-4-yl]methoxy}-19-methyl-15-[2-(4-methylpiperazin-1-yl)ethyl]-7,8,15,16-tetrahydro-14H-17,20-etheno-13,9-(metheno)-6-oxa-2-thia-3,5,15-triazacyclooctadeca[1,2,3-cd]indene-7-carboxylate (S-22

abbvie

10698574-1537-FINAL in DMSO BC#6994 $17
j500

Acq: VnmrJ VERSION 3.2 REVISION A/j5001
Proc: VnmrJ VERSION 3.2 REVISION A/borat

Chemist: ROB RISI
Experiment: s2pul

nmr3314407

May 17 2016

Expected protons=51
Whole protons observed=48
Total integral =48.997

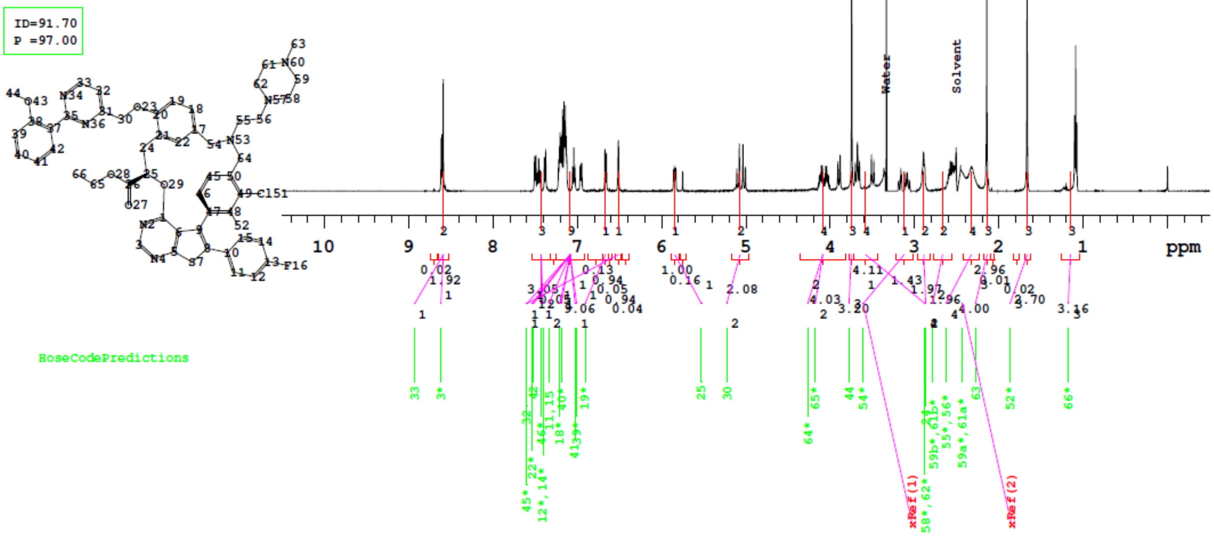

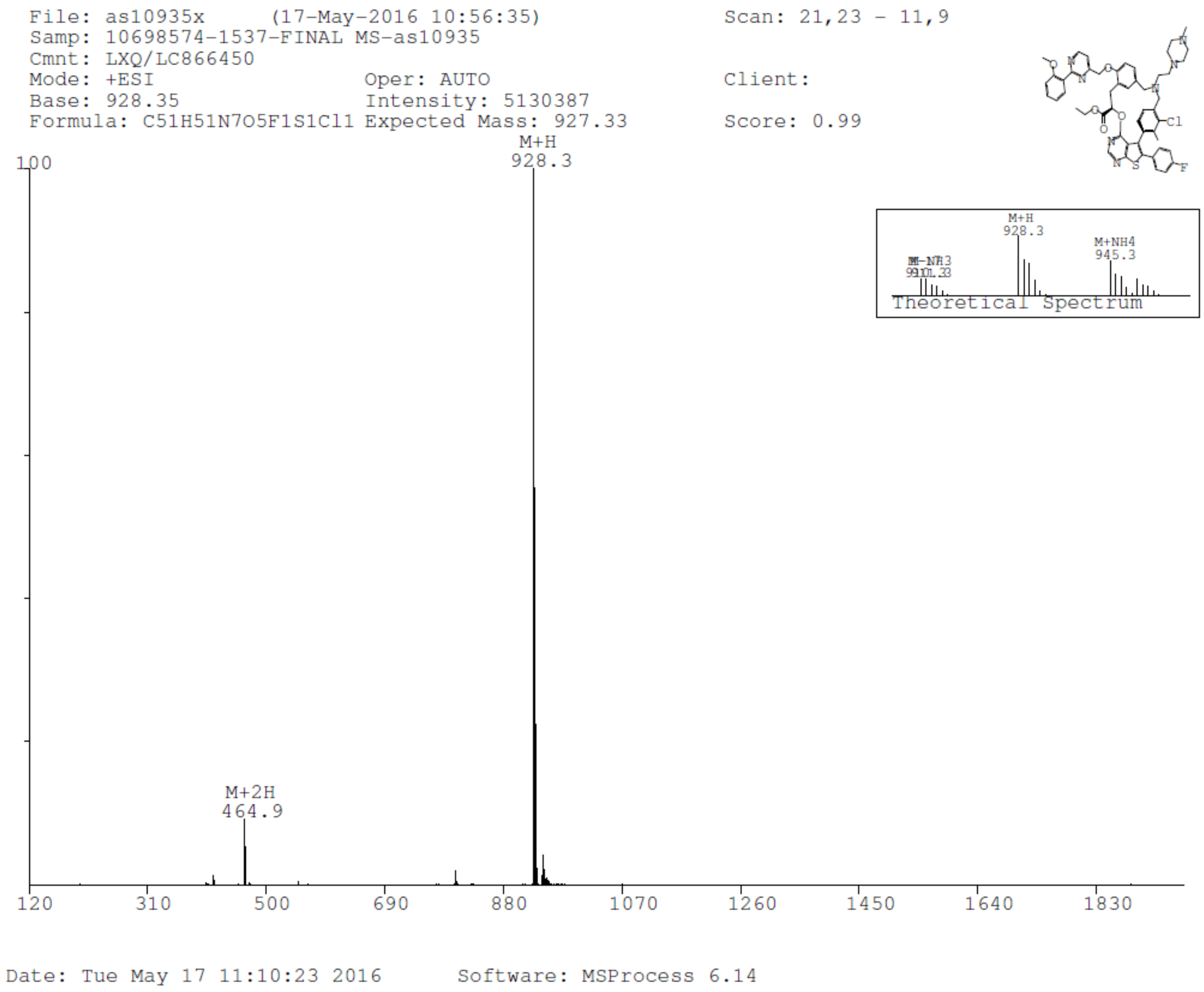

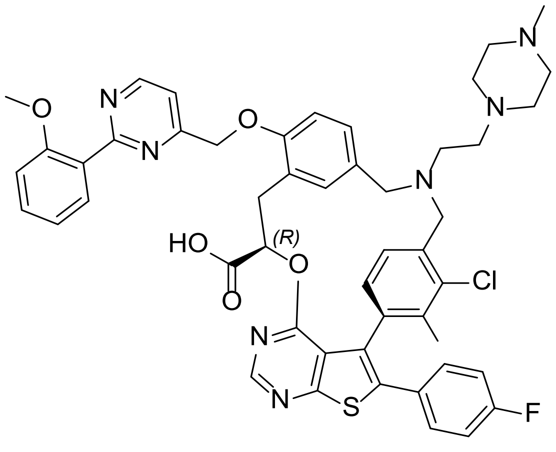

(7R,20S)-18-chloro-1-(4-fluorophenyl)-10-{[2-(2-methoxyphenyl)pyrimidin-4-yl]methoxy}-19-methyl-15-[2-(4-methylpiperazin-1-yl)ethyl]-7,8,15,16-tetrahydro-14H-17,20-etheno-13,9-(metheno)-6-oxa-2-thia-3,5,15-triazacyclooctadeca[1,2,3-cd]indene-7-carboxylic acid (1).

abbvie

10008519-2218 in DMSO $18 BC1934 4mg
Temp = 27 C
C49H47N7O5F1S1Cl1
v501

Acq: VnmrJ VERSION 3.2 REVISION A/v501
Proc: VnmrJ VERSION 3.2 REVISION A/i5001

Chemist: BRYAN SORENSEN
Experiment: s2pul

nmr3314878

May 18 2016

Expected protons=47
Whole protons observed=38
Total integral =39.005

ID=83.70
P =95.00

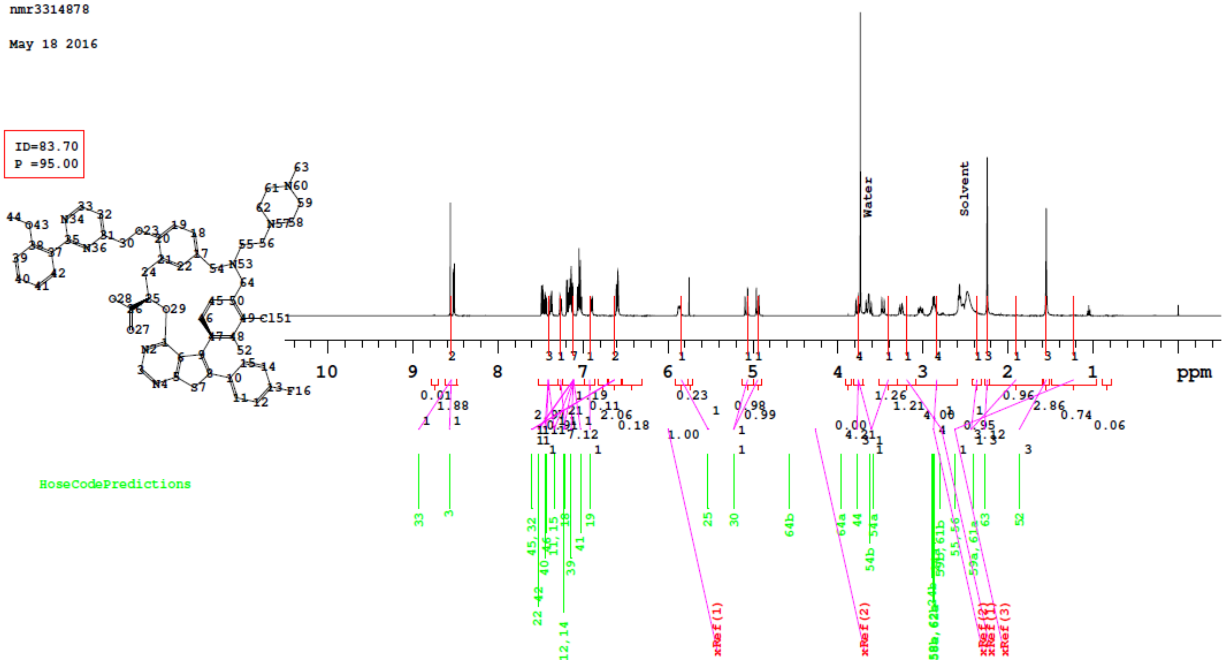

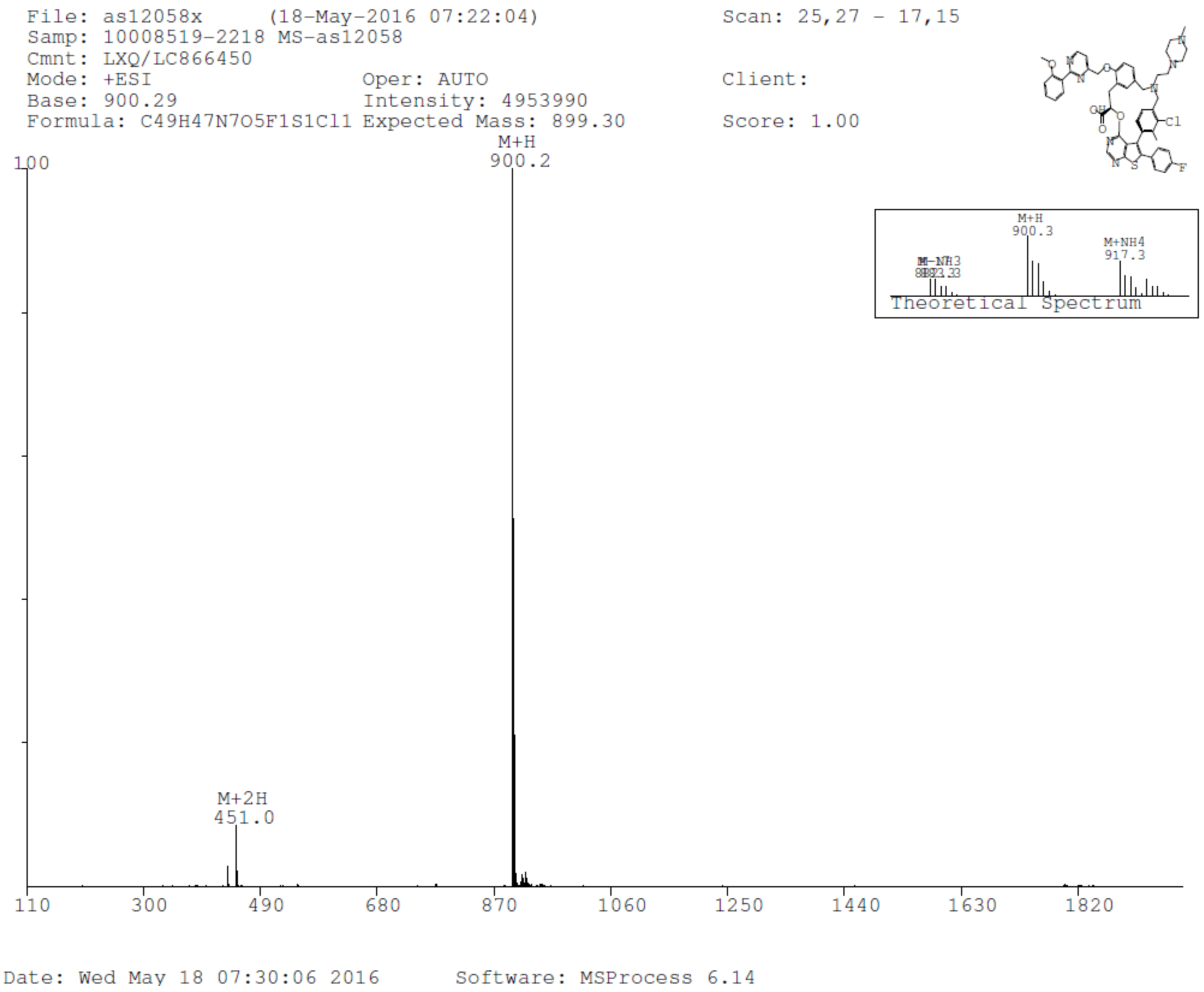

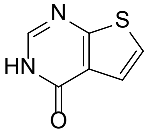

Thieno[2,3-d]pyrimidin-4(3H)-one (S-23).

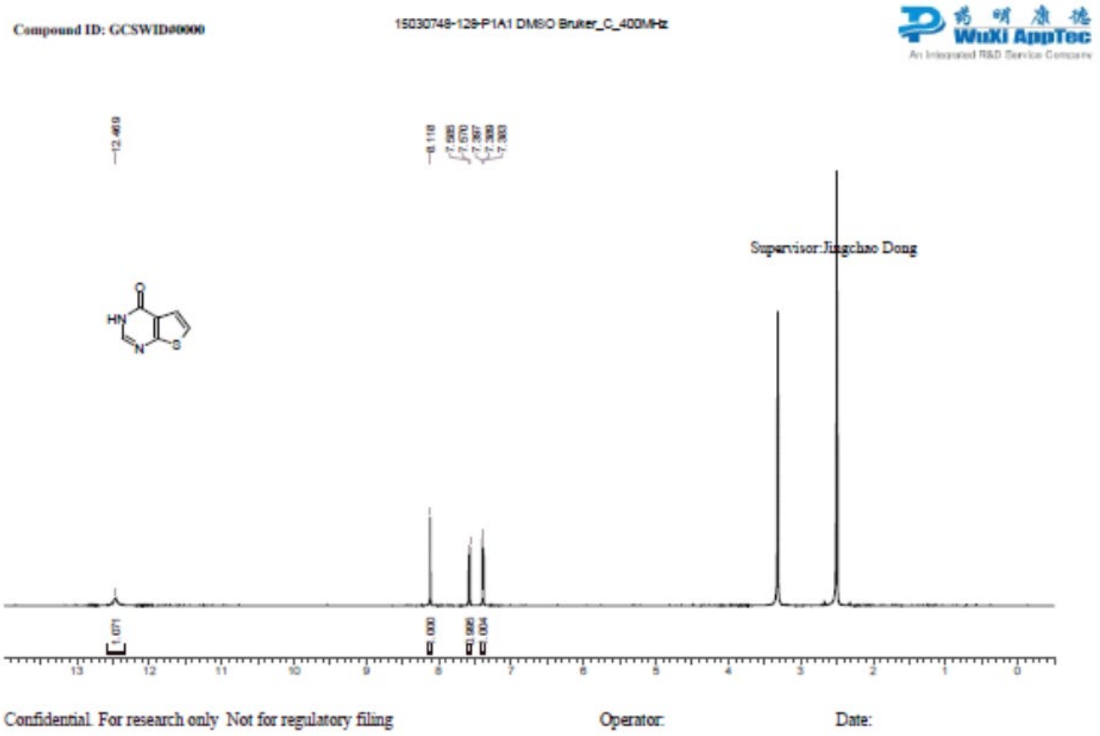

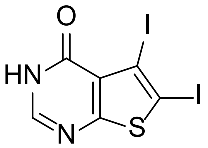

5,6-diiodothieno[2,3-d]pyrimidin-4(3H)-one (S-24)..

Compound ID: GCSWID#0000

15042986-0252-500 DMSO Bruker_E_400MHz

WuXi AppTec
An Integrated R&D Service Company

Supervisor:Tao Guo

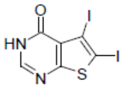

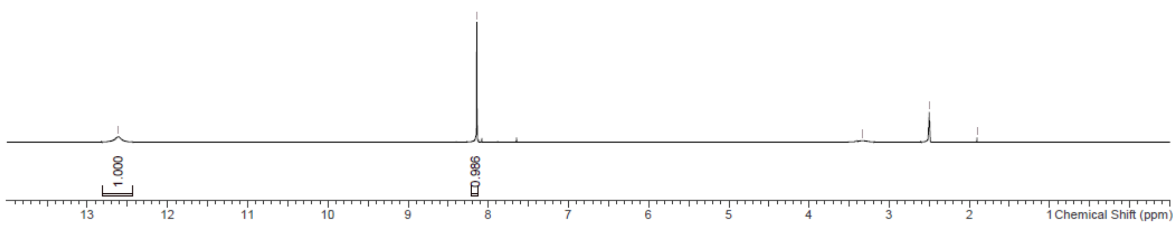

Confidential. For research only Not for regulatory filing

Operator:

Date:

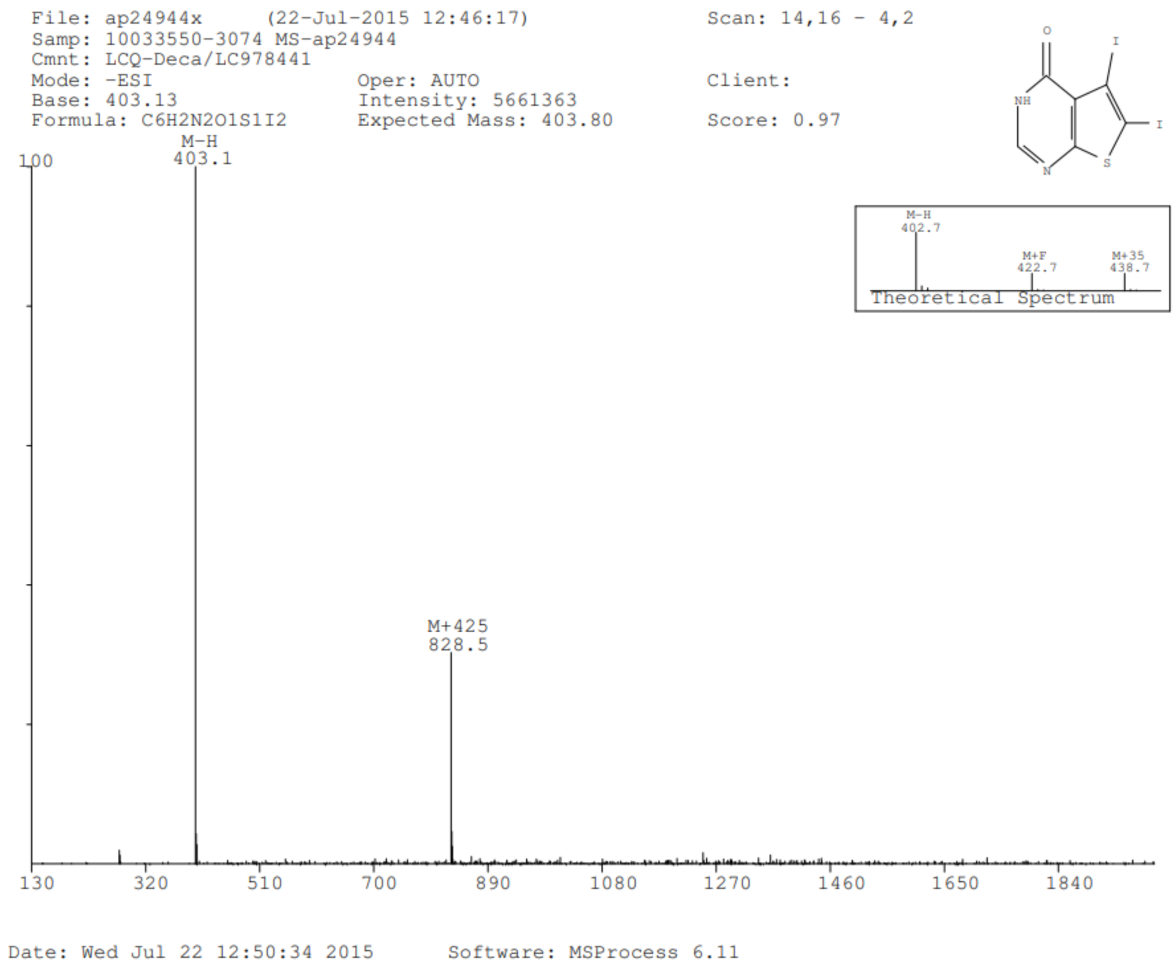

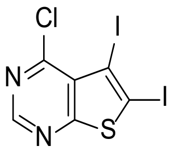

4-chloro-5,6-diiodothieno[2,3-d]pyrimidine (S-25

Compound ID: RSU-0694_4

15042986-0268-0 DMSO Bruker_C_400MHz

Supervisor:Tao Guo

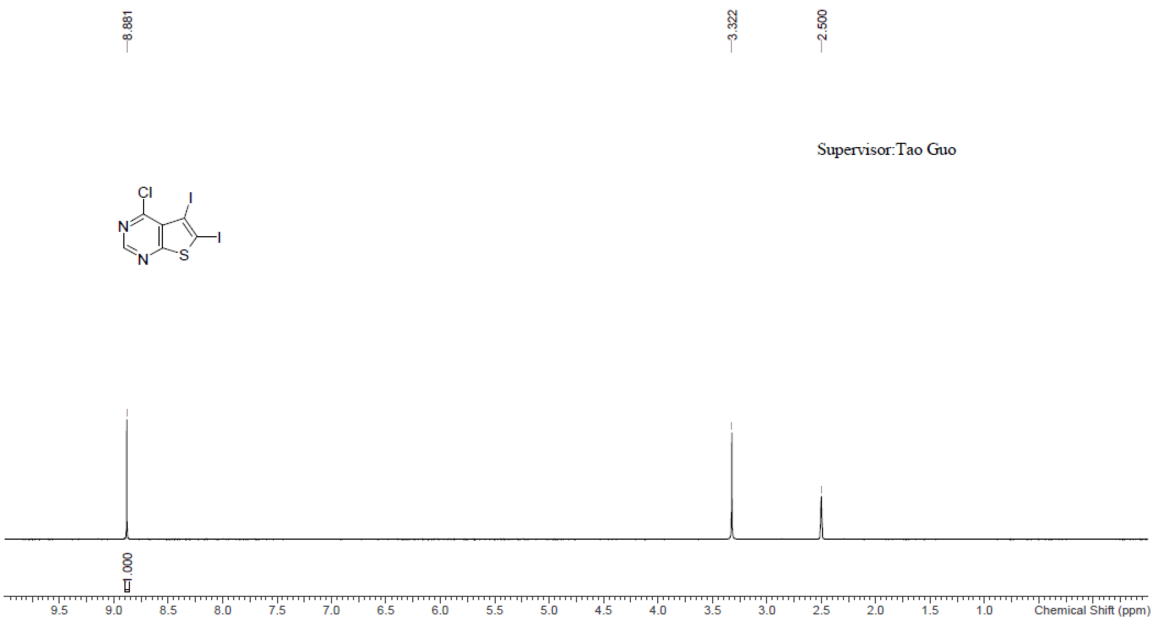

Confidential. For research only Not for regulatory filing

Operator:

Date:

LCMS REPORT

Compound ID	:	RSU-0666_4 ->
Sample ID	:	15042986-0113-0
Injection Date	:	3. Apr. 2018
Acq Operator	:	TF0001
Location	:	P2-C-09
Inj. Vol.	:	0.20 uL
Acq Method	:	D:\METHOD\WUXIAB25.M
Data Filename	:	D:\DATA\180402K-D\2CI-0801.D
Instrument	:	LCMS-K

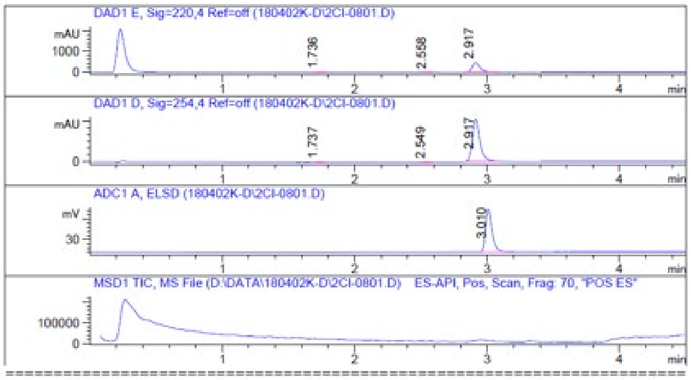

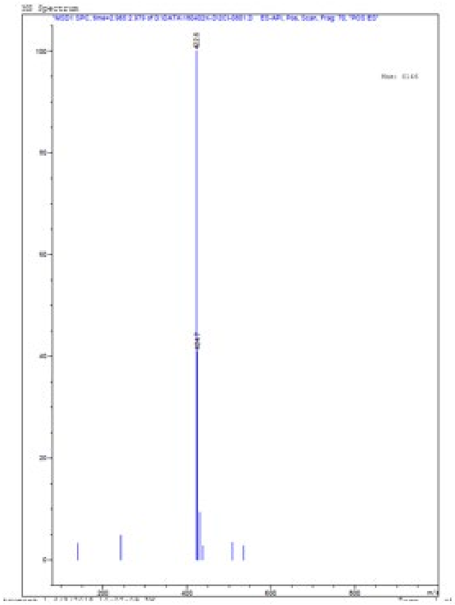

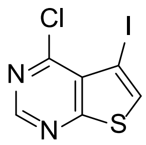

4-chloro-5-iodothieno[2,3-d]pyrimidine (S-26).

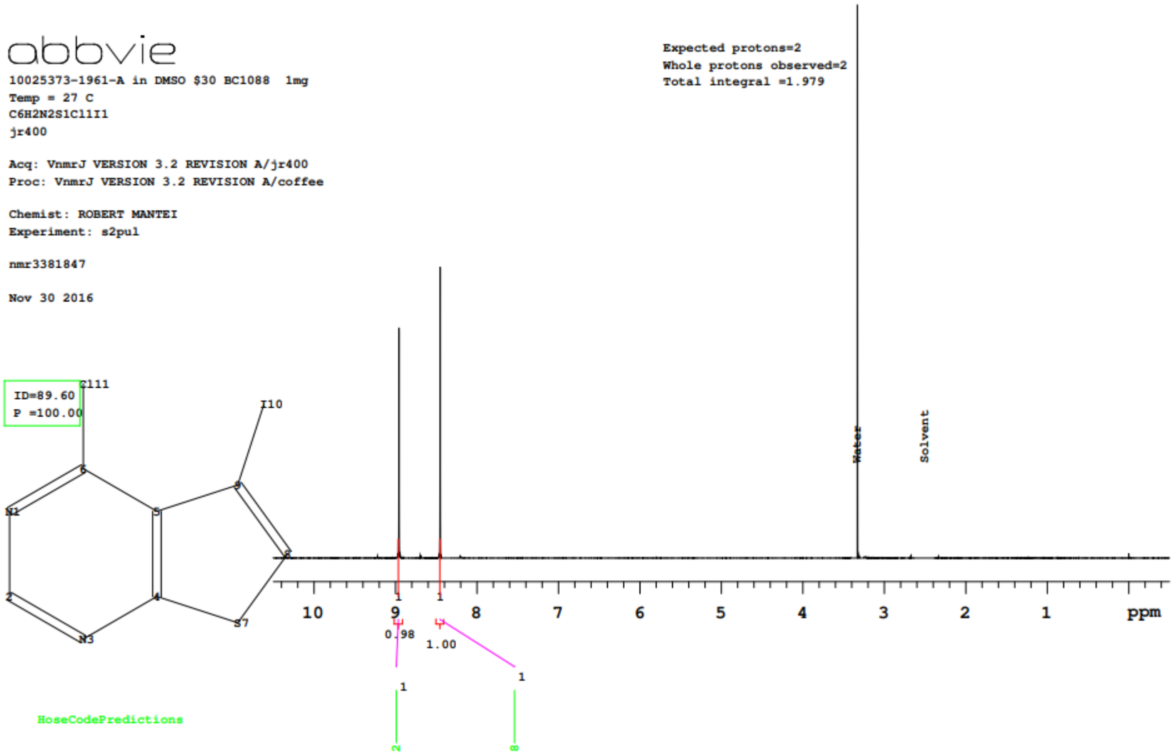

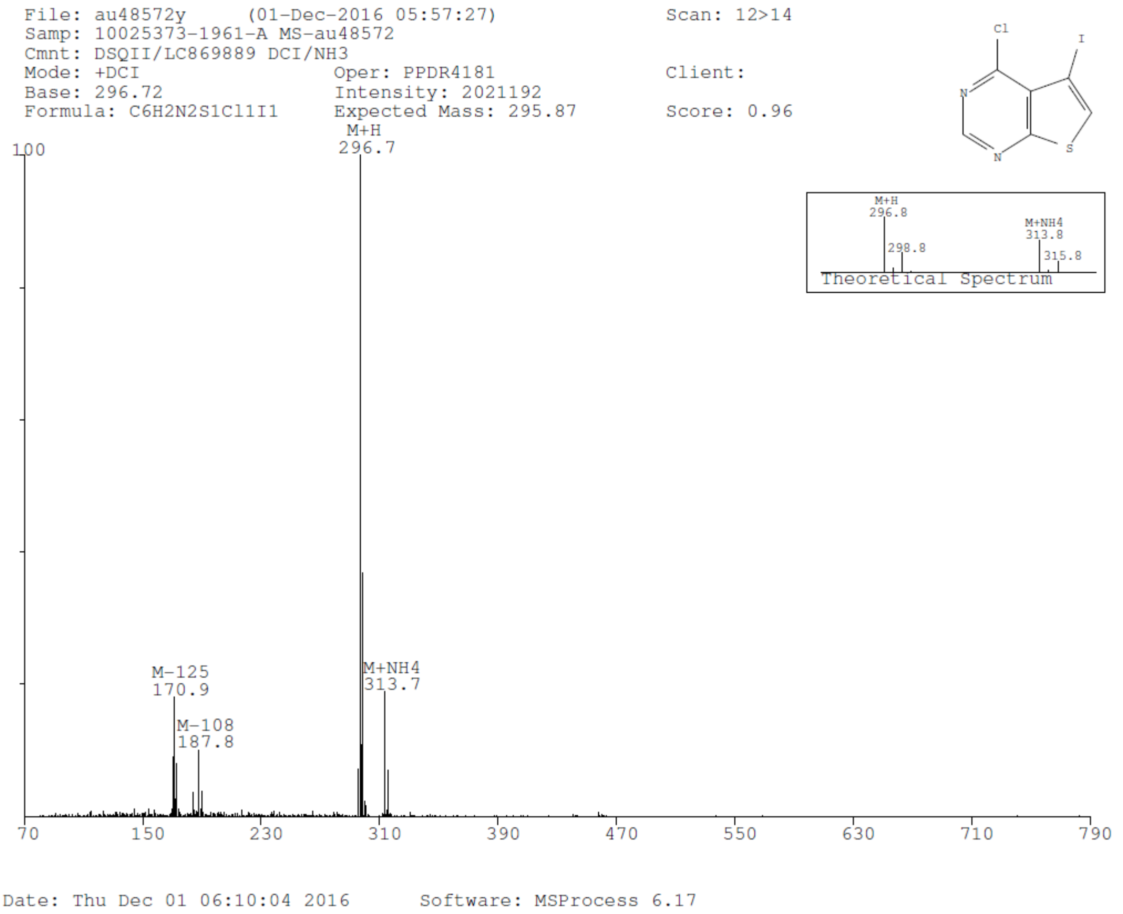

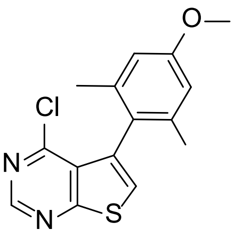

4-chloro-5-(4-methoxy-2,6-dimethylphenyl)thieno[2,3-d]pyrimidine (S-27).

Compound ID:

15002679-1075-P1B DMSO Varian_Y_400MHz

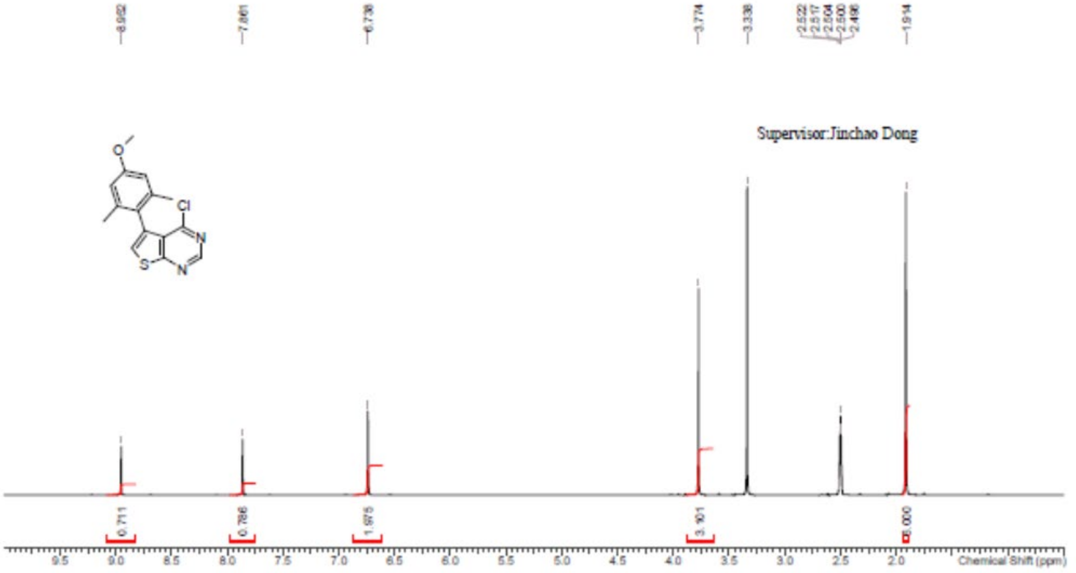

Operator: Date:

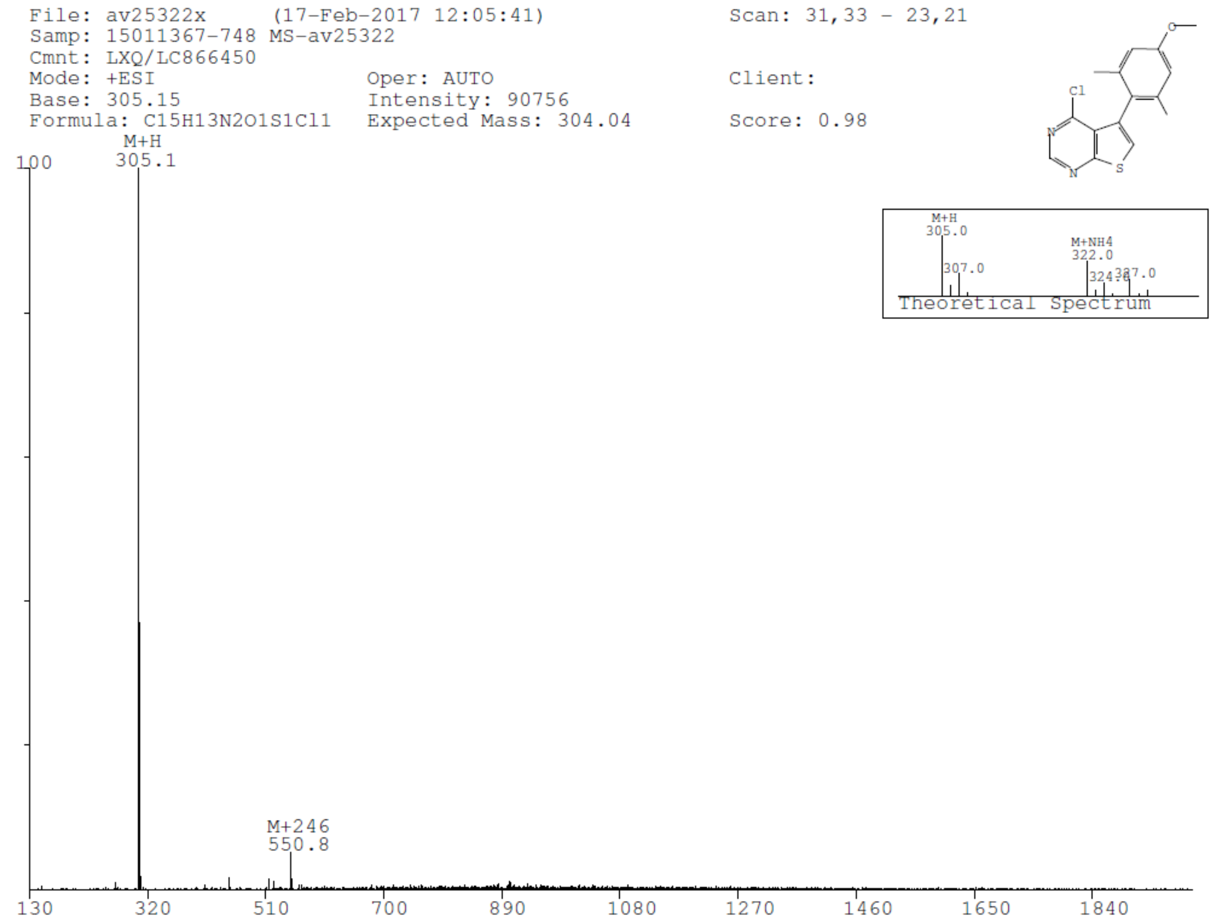

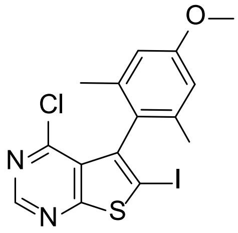

4-chloro-6-iodo-5-(4-methoxy-2,6-dimethylphenyl)thieno[2,3-d]pyrimidine (S-28).

15002679-939-P1A CDCl3 Varian_Y_400MHz

Supervisor:Jingchao Dong

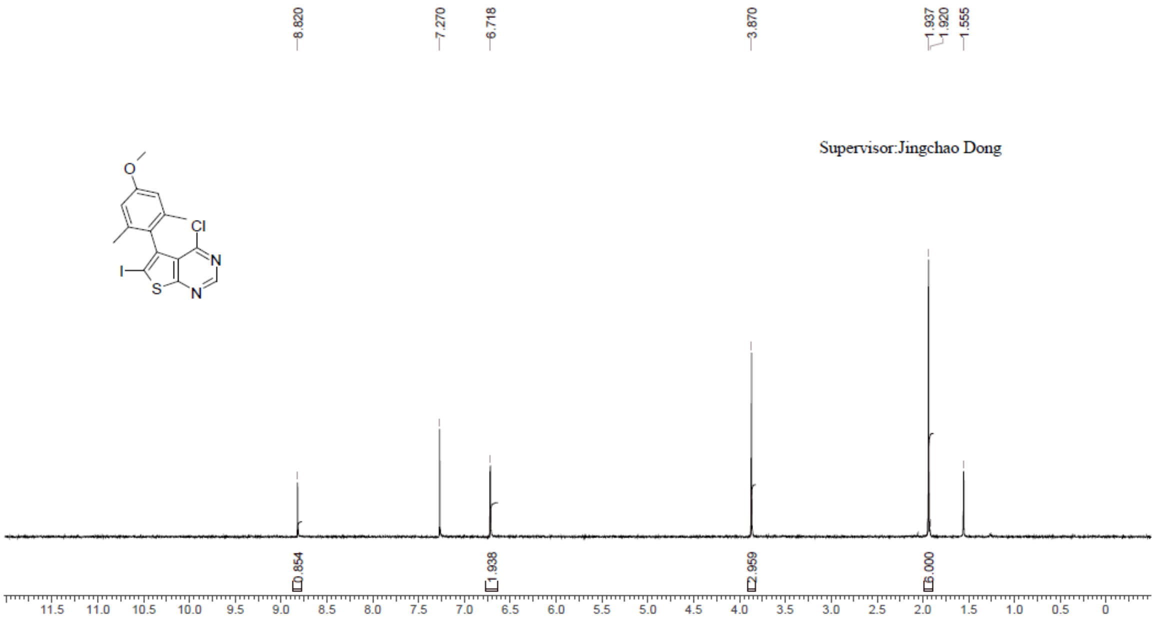

Confidential. For research only Not for regulatory filing Operator: Date:

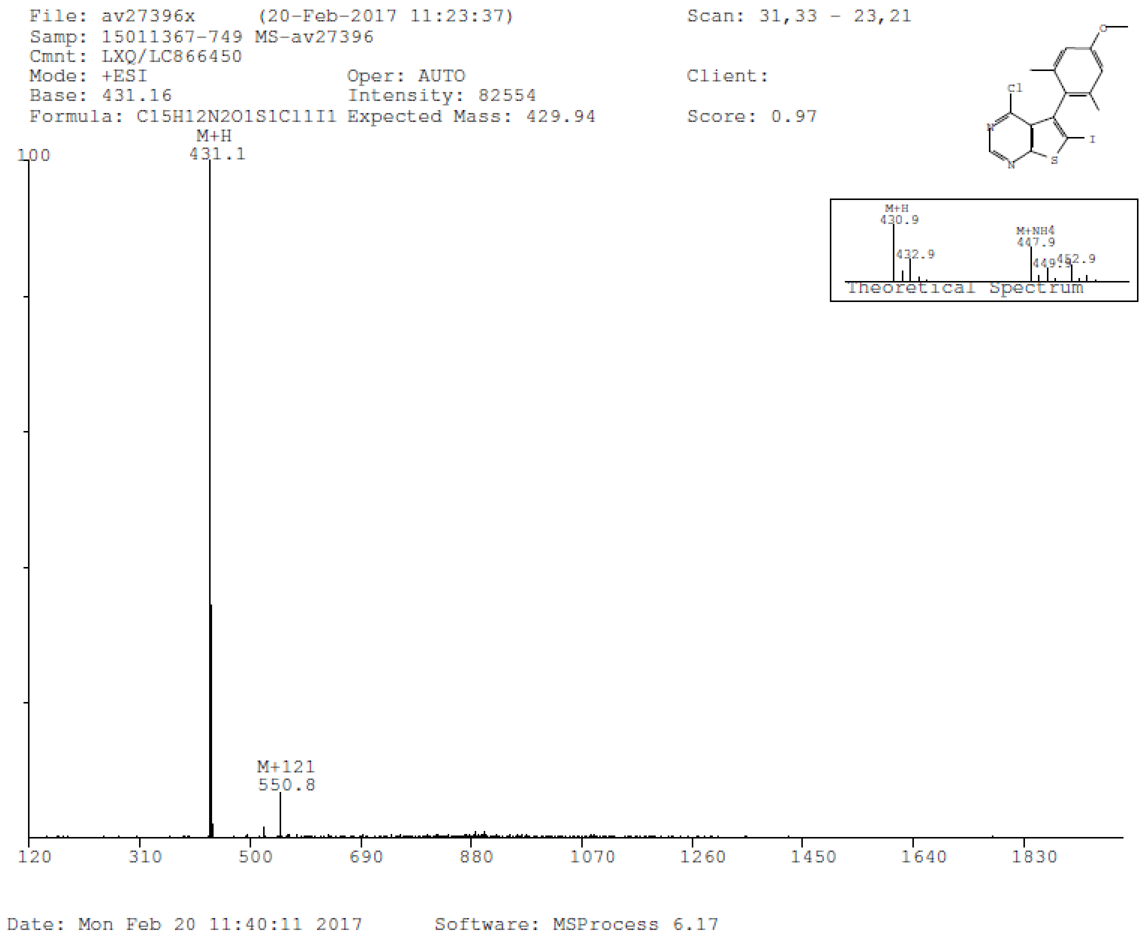

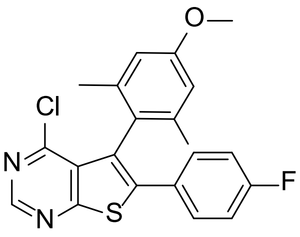

4-chloro-6-(4-fluorophenyl)-5-(4-methoxy-2,6-dimethylphenyl)thieno[2,3-d]pyrimidine (S-29).

Compound ID: WuXi1406_18

15002679-942-P1A CDCl3 Varian_S_400MHz

WuXi AppTec
An Integrated R&D Service Company

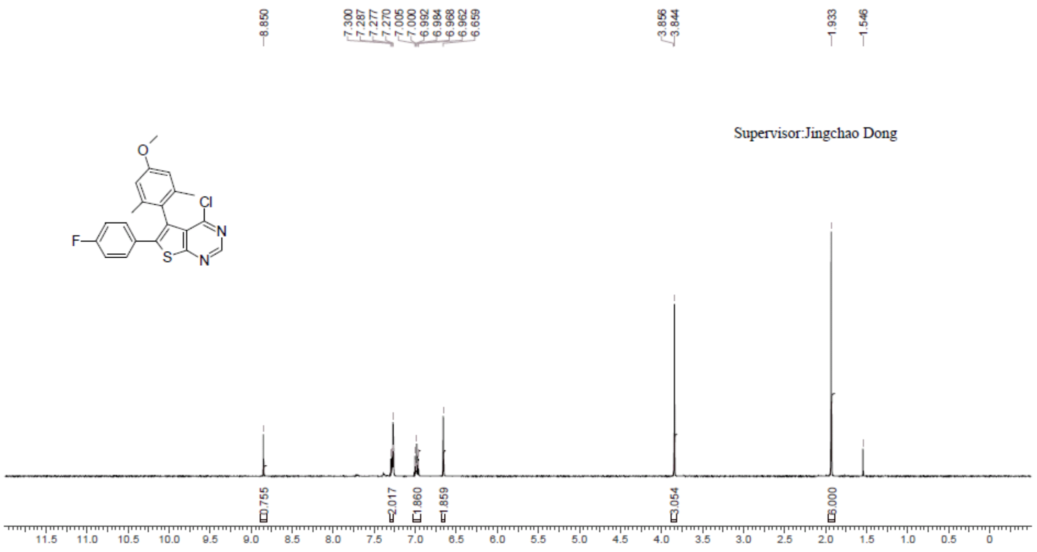

Confidential. For research only Not for regulatory filing   Operator: Date:

```
File: av32878x      (23-Feb-2017 12:44:39)              Scan: 29,31 - 3,1
Samp: 15011367-756 MS-av32878
Cmnt: LXQ/LC866450
Mode: +ESI                  Oper: AUTO                  Client:
Base: 399.15                Intensity: 82918
Formula: C21H16N2O1F1S1Cl1 Expected Mass: 398.07        Score: 0.97
```

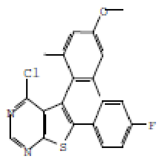

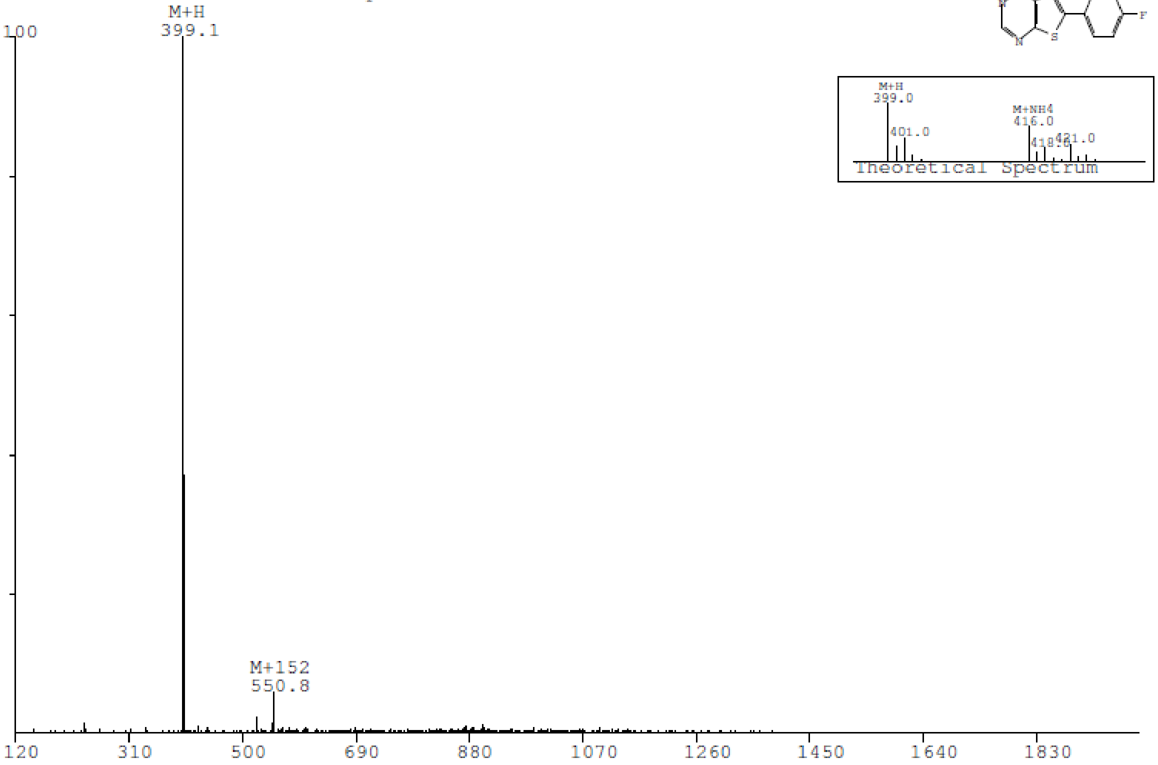

Date: Thu Feb 23 13:03:06 2017   Software: MSProcess 6.17

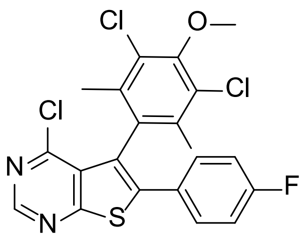

4-chloro-5-(3,5-dichloro-4-methoxy-2,6-dimethylphenyl)-6-(4-fluorophenyl)thieno[2,3-d]pyrimidine (S-30).

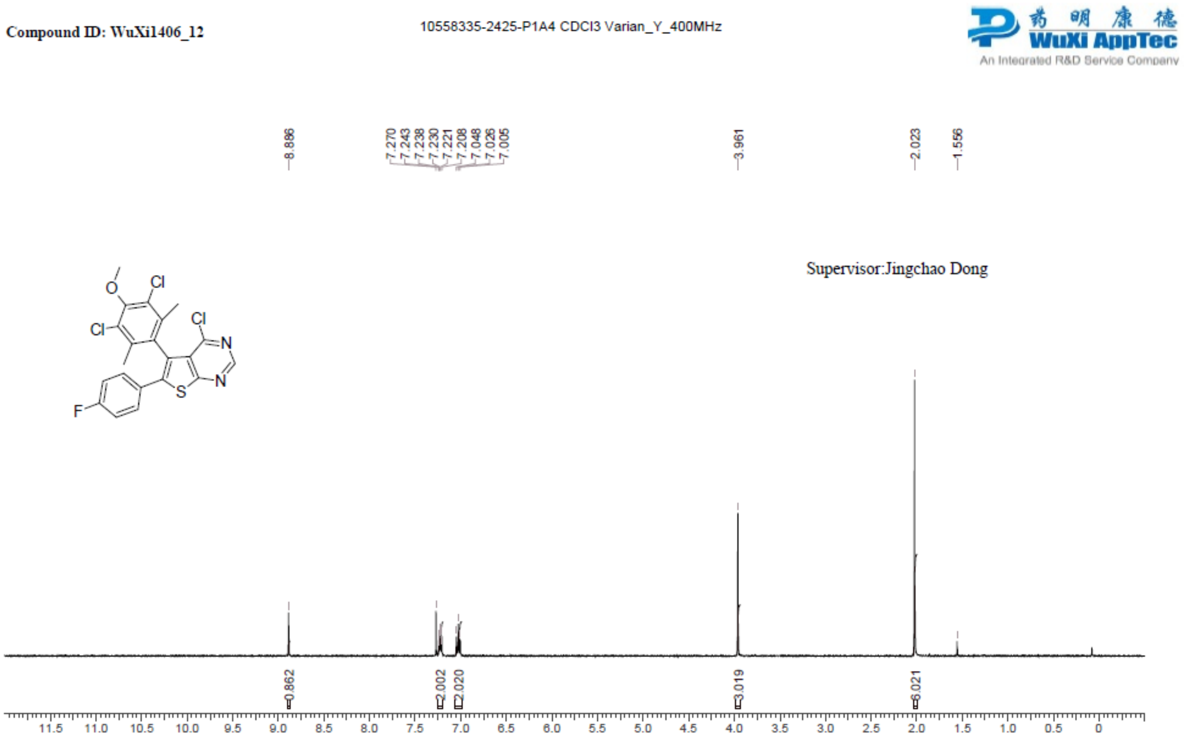

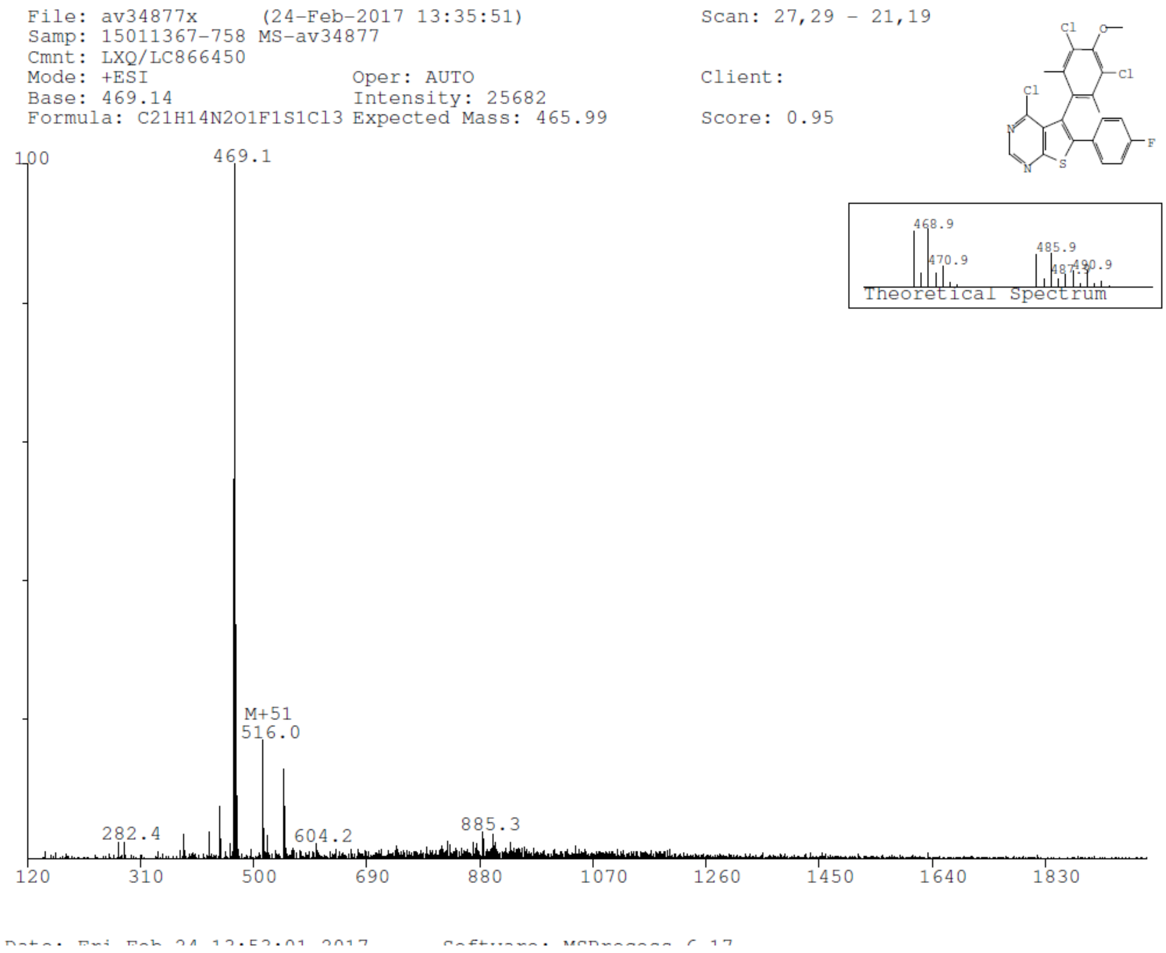

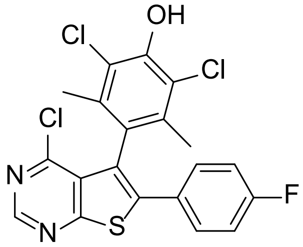

2,6-dichloro-4-(4-chloro-6-(4-fluorophenyl)thieno[2,3-d]pyrimidin-5-yl)-3,5-dimethylphenol (S-31).

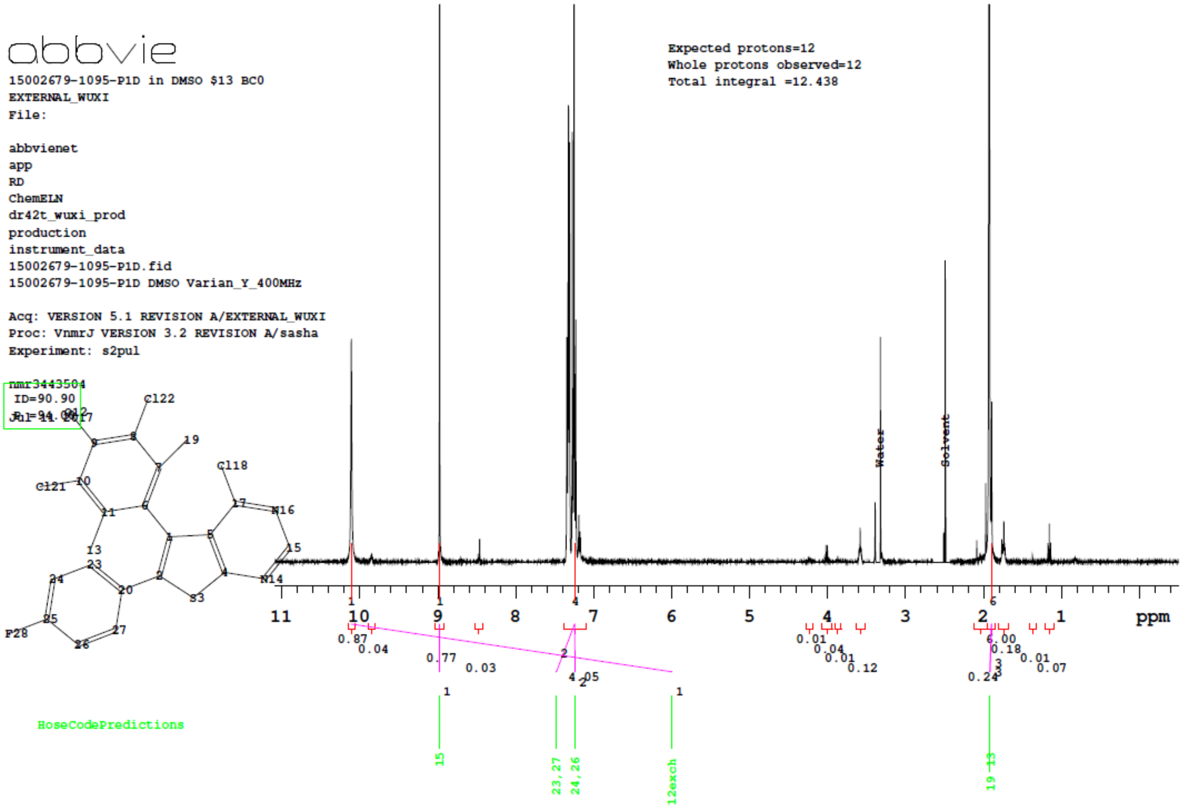
abbvie
15002679-1095-P1D in DMSO $13 BC0
EXTERNAL_WUXI
File:
abbvienet
app
RD
ChemELN
dr42t_wuxi_prod
production
instrument_data
15002679-1095-P1D.fid
15002679-1095-P1D DMSO Varian_Y_400MHz
Acq: VERSION 5.1 REVISION A/EXTERNAL_WUXI
Proc: VnmrJ VERSION 3.2 REVISION A/sasha
Experiment: s2pul
nmr3443504
ID=90.90
Jul 11 2017
Expected protons=12
Whole protons observed=12
Total integral =12.438
Water
Solvent
ppm
HoseCodePredictions
12exch

Compound Spectrum SmartFormula Report

Analysis Info

Analysis Name	D:\Data\External\Abbvie\Brady\03192018\Abbvie-Brady-G_30_01_1886.d	Acquisition Date	3/19/2018 10:15:59 AM
Method	MethodSet-FIA-MS-C1-30_A1-70_B1-2min-03192018.m	Operator	Demo User
Sample Name	Abbvie-Brady-G	Instrument	impact II 1825265.10104
Comment			

Acquisition Parameter

Source Type	ESI	Ion Polarity	Positive	Set Nebulizer	0.4 Bar
Focus	Active	Set Capillary	4500 V	Set Dry Heater	200 °C
Scan Begin	100 m/z	Set End Plate Offset	-500 V	Set Dry Gas	4.0 l/min
Scan End	3000 m/z	Set Charging Voltage	2000 V	Set Divert Valve	Source
		Set Corona	0 nA	Set APCI Heater	0 °C

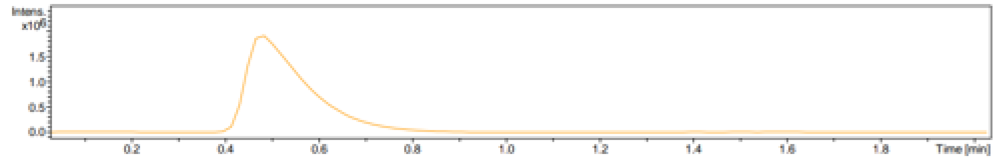

+MS, 0.6-0.7min #34-40

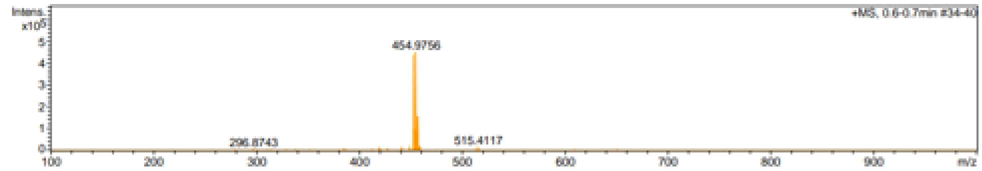

Meas. m/z	z	Adduct	Ion Formula	m/z	Sum Formula	|err| [mDa]	err [ppm]	N-Rule	Score
452.9785	1+	M+H	C20H13Cl3FN2OS	452.9793	C20H12Cl3FN2OS	0.8	1.7	ok	100.00

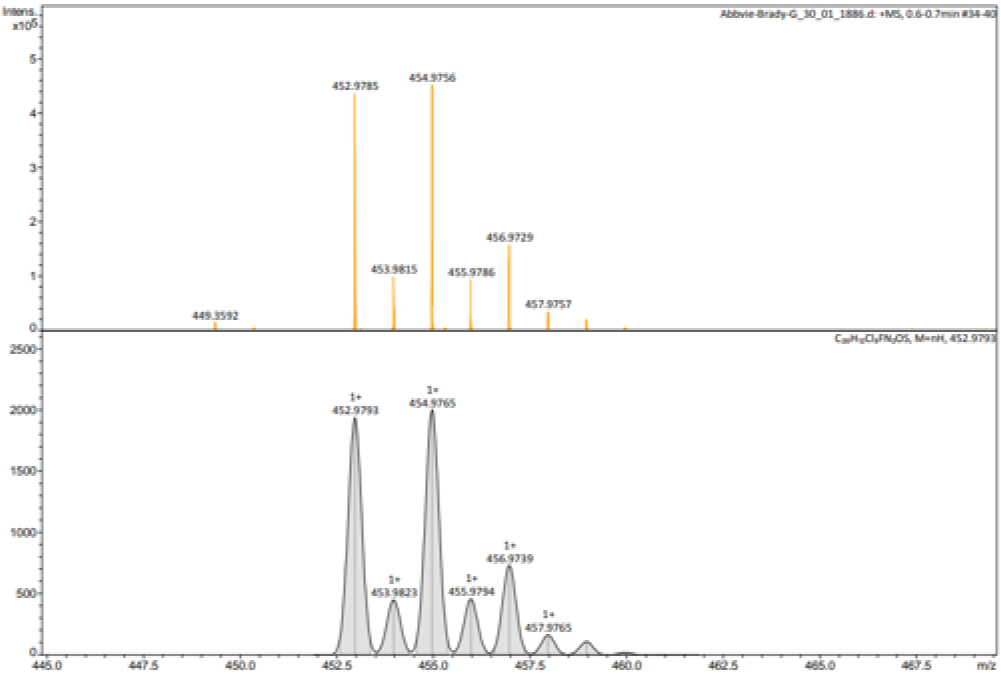

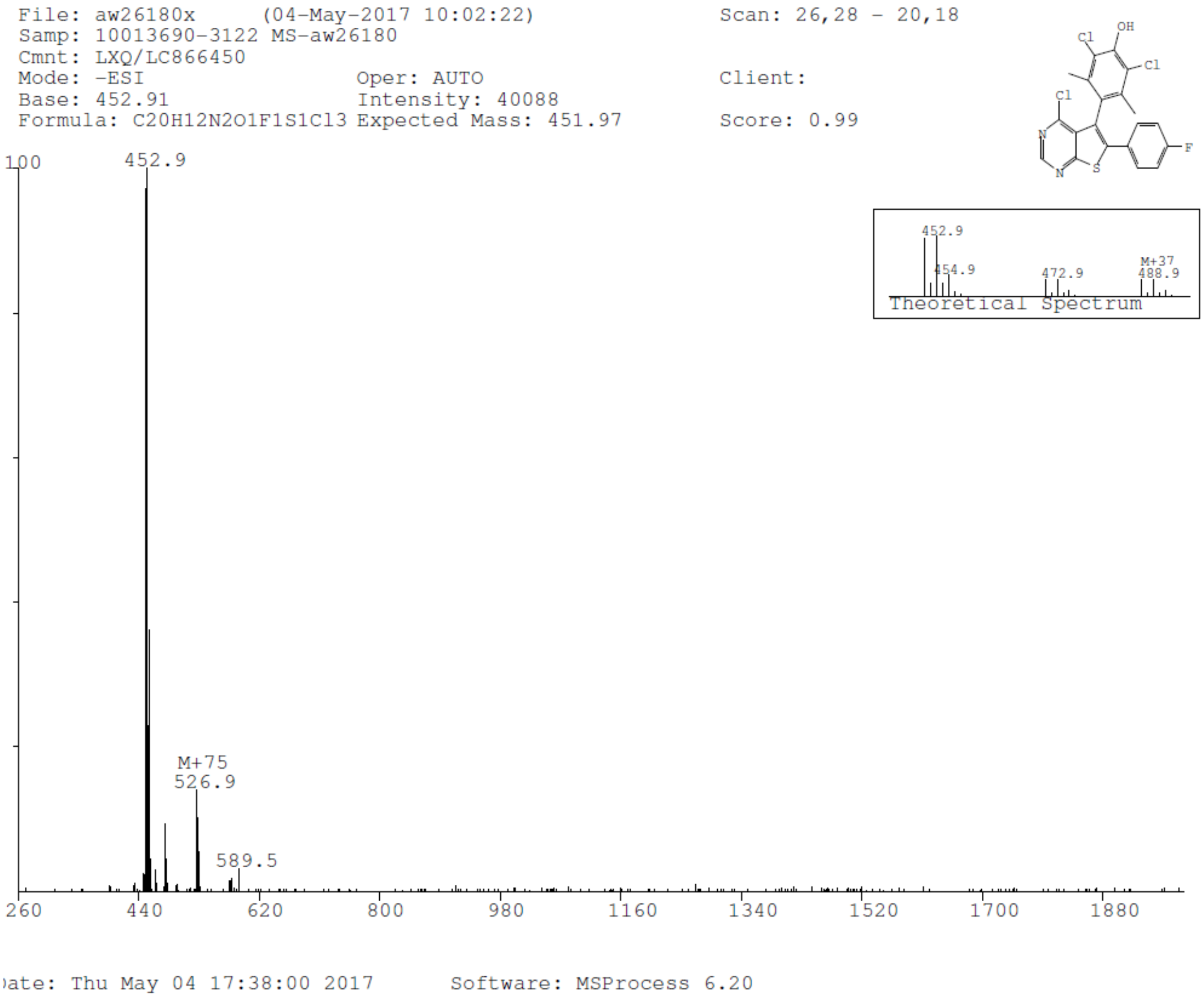

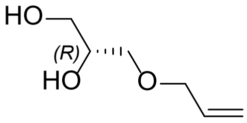

(R)-3-(allyloxy)propane-1,2-diol (S-32).

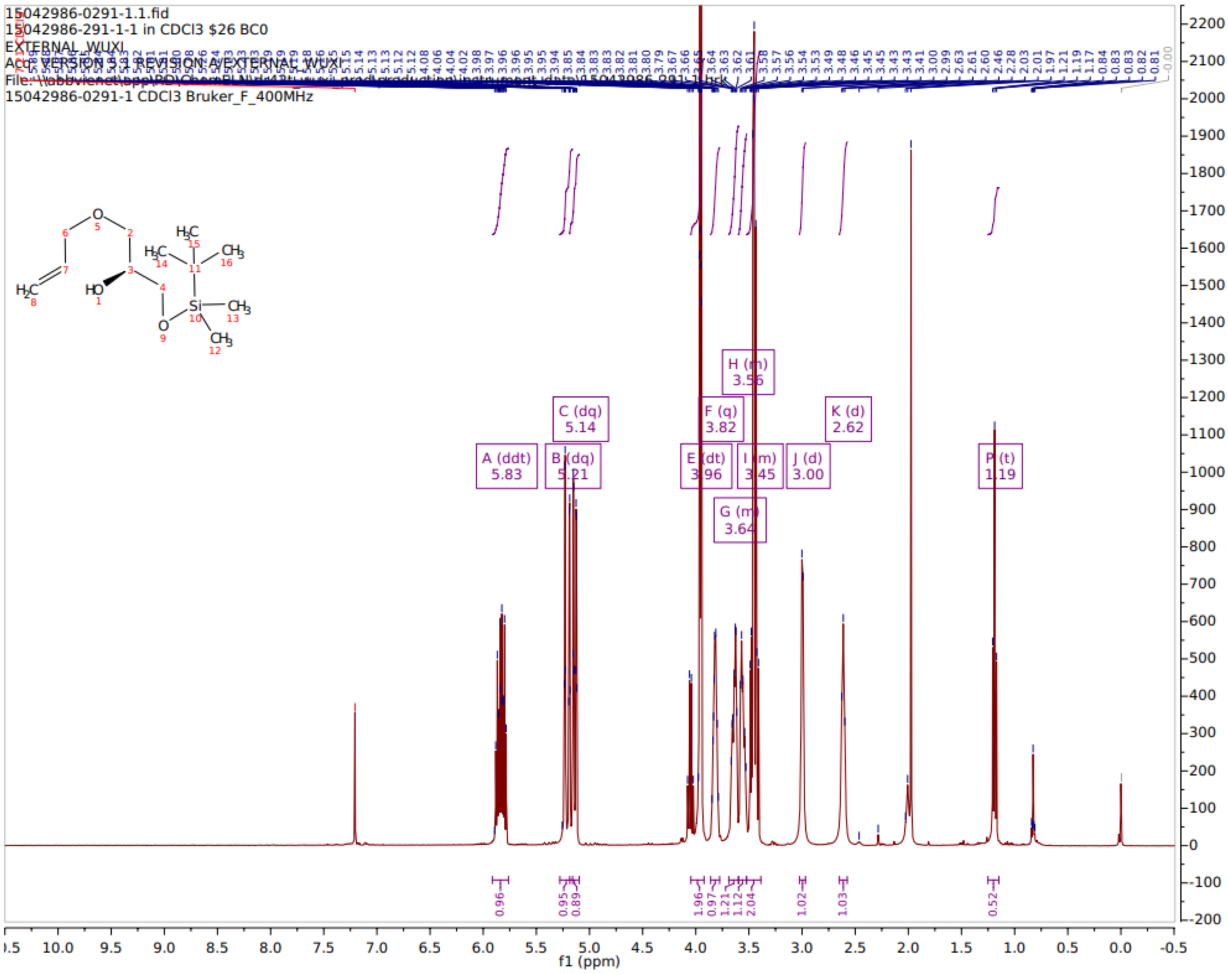

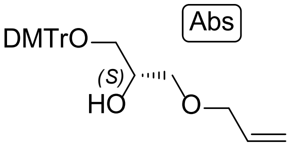

(S)-1-(allyloxy)-3-(bis(4-methoxyphenyl)(phenyl)methoxy)propan-2-ol (S-33).

abbvie

15006715-802-A in CDCL3 $08 BC2650 10mg
Temp = 27 C
C27H30O5
mrs400

Acq: VnmrJ VERSION 3.2 REVISION A/mrs400
Proc: VnmrJ VERSION 3.2 REVISION A/ui5001

Chemist: PATRICK BRADY
Experiment: s2pul

nmr3434552

Jun 8 2017

Expected protons=30
Whole protons observed=24
Total integral =25.997

ID=83.90
P =87.00

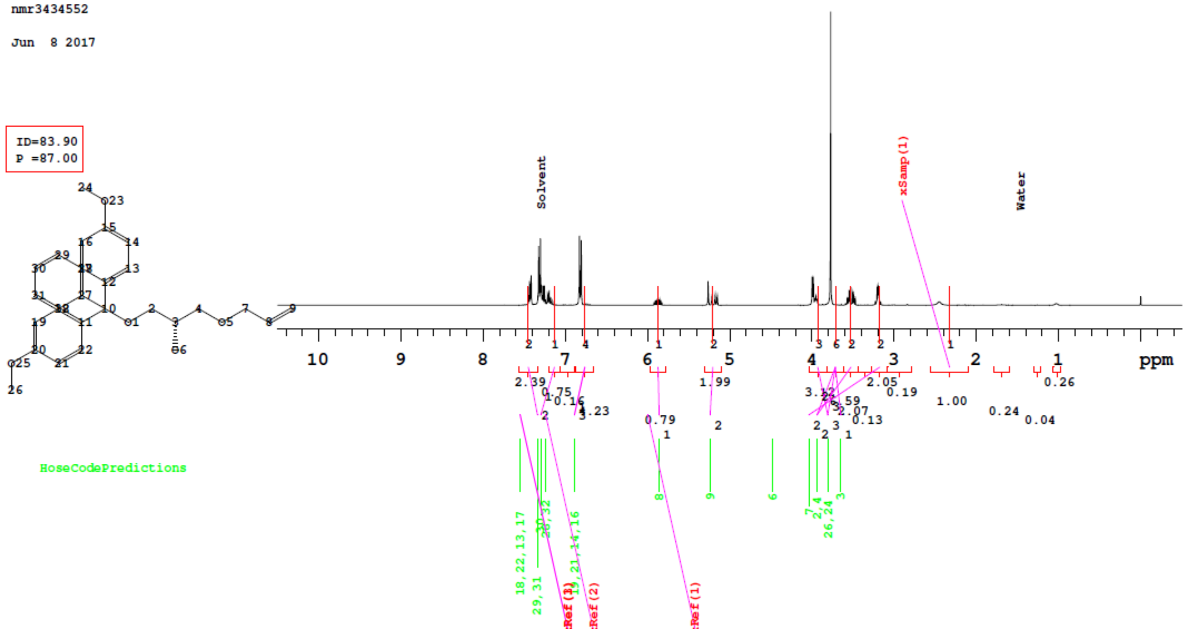

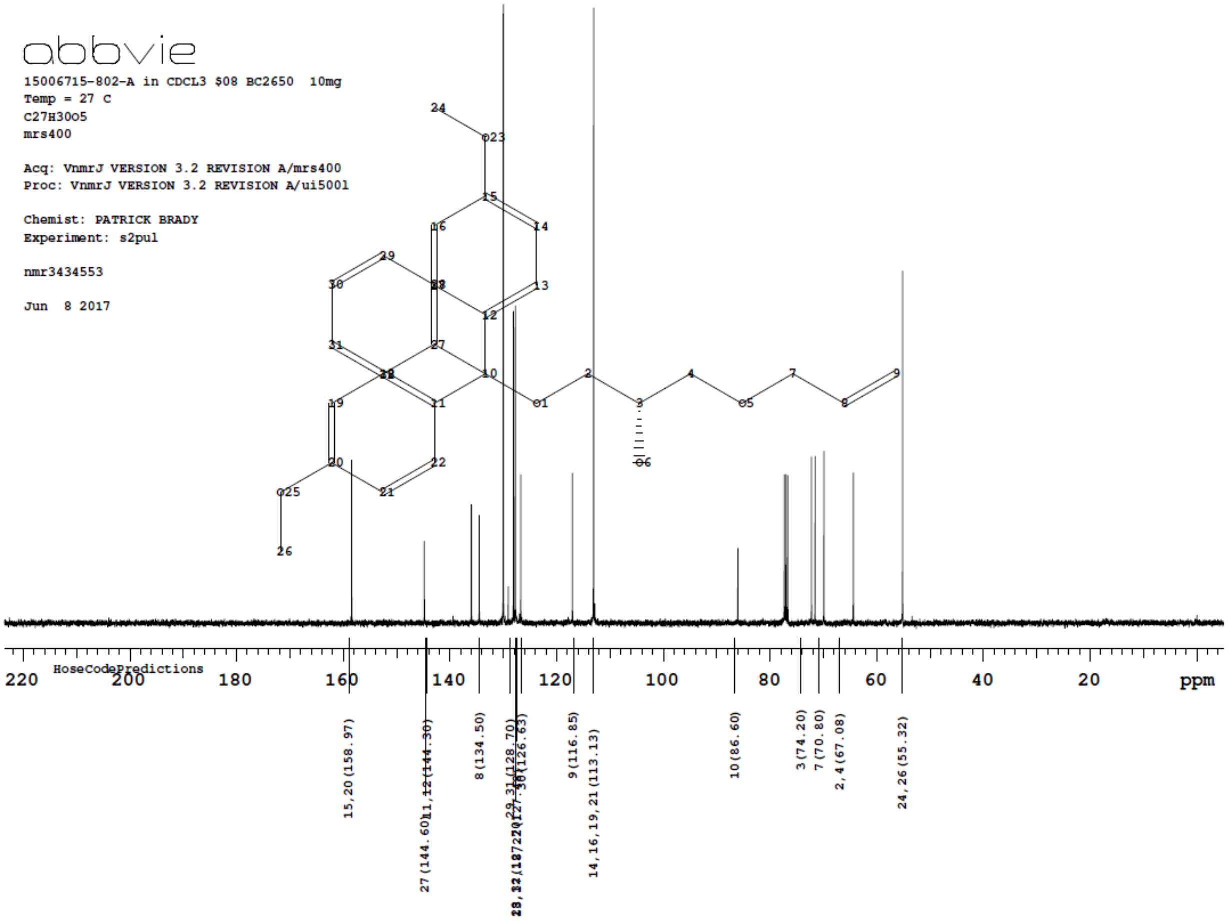
abbvie
15006715-802-A in CDCL3 $08 BC2650 10mg
Temp = 27 C
C27H30O5
mrs400
Acq: VnmrJ VERSION 3.2 REVISION A/mrs400
Proc: VnmrJ VERSION 3.2 REVISION A/ui5001
Chemist: PATRICK BRADY
Experiment: s2pul
nmr3434553
Jun 8 2017
HoseCodePredictions
220 200 180 160 140 120 100 80 60 40 20 ppm
15,20(158.97)
27(144.60)
8(134.50)
9(116.85)
14,16,19,21(113.13)
10(86.60)
3(74.20)
7(70.80)
2,4(67.08)
24,26(55.32)

Compound Spectrum SmartFormula Report

Analysis Info

Analysis Name	D:\Data\External\Abbvie\Brady\03192018\Abbvie-Brady-R_40_01_1896.d	Acquisition Date	3/19/2018 10:44:38 AM	
Method	MethodSet-FIA-MS-C1-30_A1-70_B1-2min-03192018.m	Operator	Demo User	
Sample Name	Abbvie-Brady-R	Instrument	impact II	1825265.10104
Comment				

Acquisition Parameter

Source Type	ESI	Ion Polarity	Positive	Set Nebulizer	0.4 Bar
Focus	Active	Set Capillary	4500 V	Set Dry Heater	200 °C
Scan Begin	100 m/z	Set End Plate Offset	-500 V	Set Dry Gas	4.0 l/min
Scan End	3000 m/z	Set Charging Voltage	2000 V	Set Divert Valve	Source
		Set Corona	0 nA	Set APCI Heater	0 °C

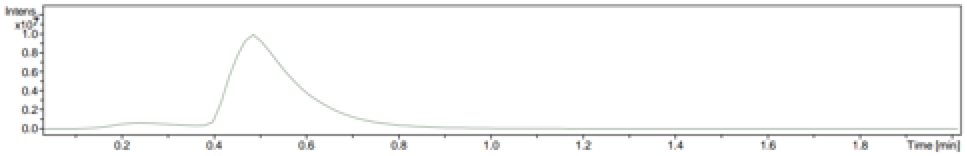

+MS, 0.2-0.3min #10-16

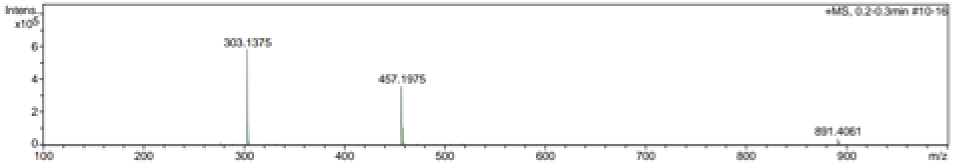

Meas. m/z	z	Adduct	Ion Formula	m/z	Sum Formula	|err| [mDa]	err [ppm]	N-Rule	Score
457.1975	1+	M+Na	C27H30NaO5	457.1985	C27H30O5	1.1	2.3	ok	100.00

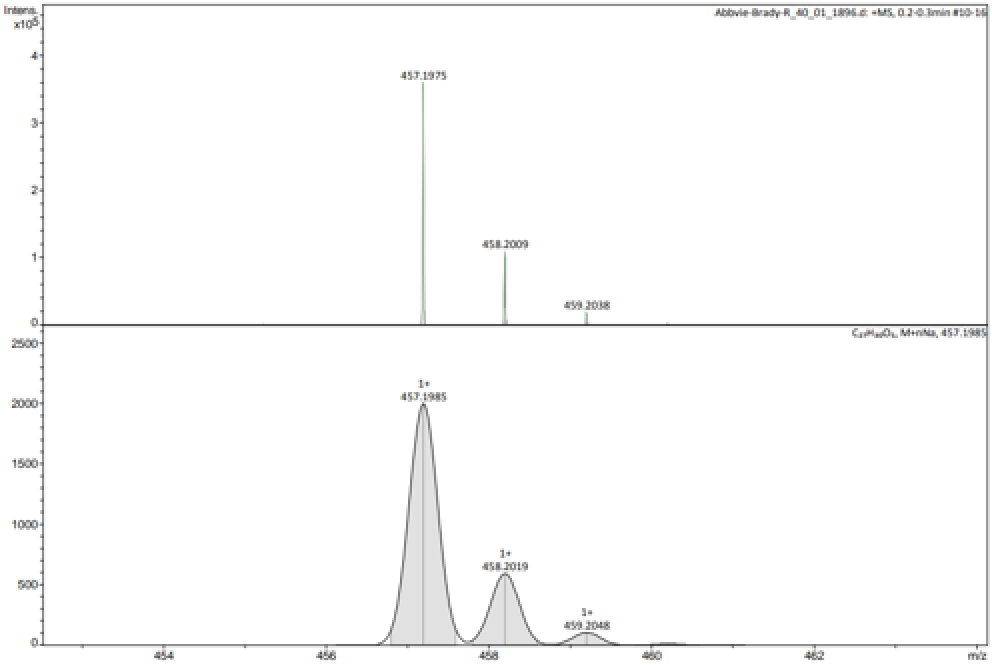

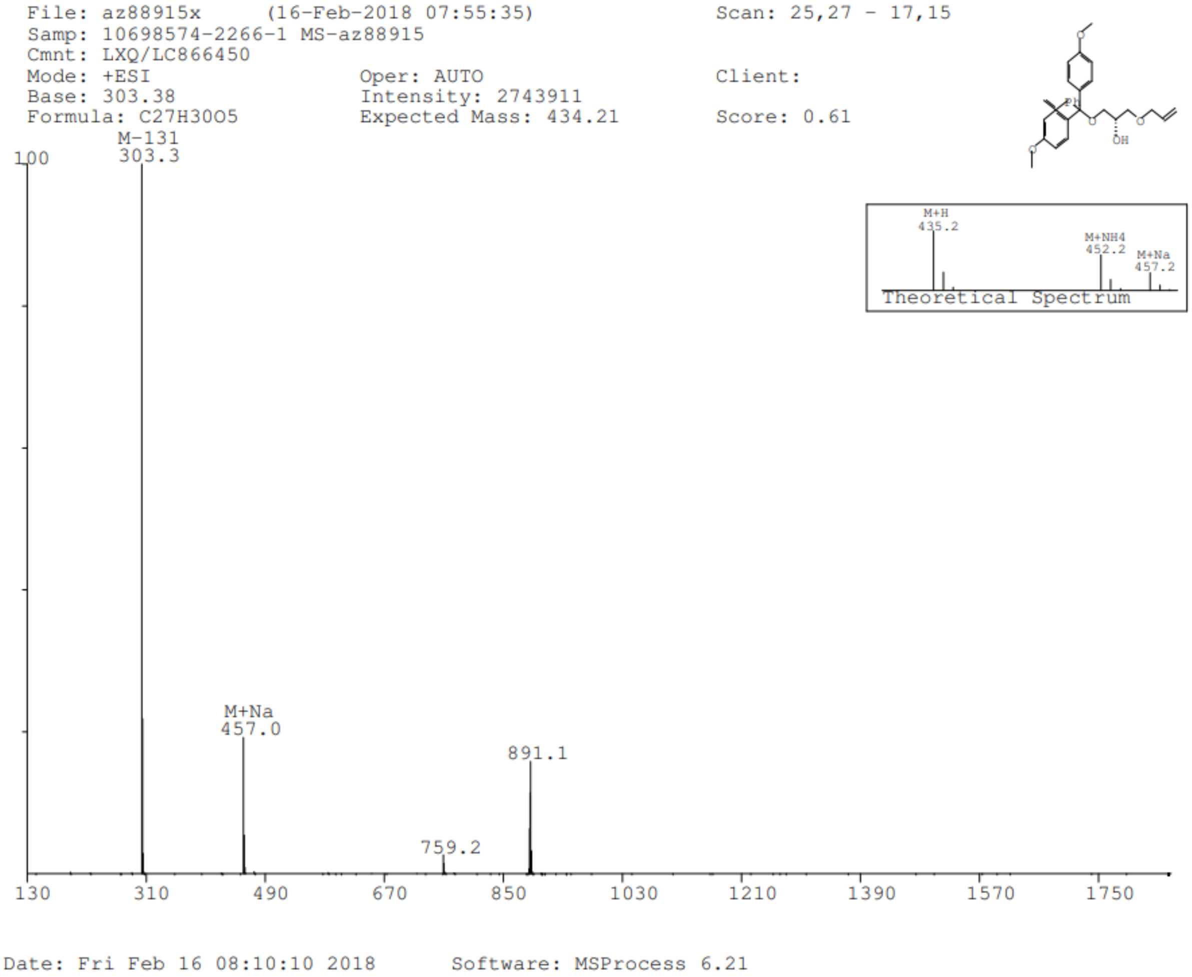

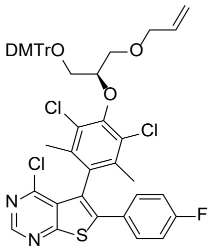

(R)-5-(4-((1-(allyloxy)-3-(bis(4-methoxyphenyl)(phenyl)methoxy)propan-2-yl)oxy)-3,5-dichloro-2,6-dimethylphenyl)-4-chloro-6-(4-fluorophenyl)thieno[2,3-d]pyrimidine (S-34).

10033550-3413 in DMSO $21 BC789 1mg
Temp = 27 C
C47H40N2O5F1S1Cl3
jr400

Acq: VnmrJ VERSION 3.2 REVISION A/jr400
Proc: VnmrJ VERSION 3.2 REVISION A/ui5001

Chemist: MATTHEW HANSEN
Solvent and water peaks subtracted
Experiment: s2pul

nmr3446106

Jul 21 2017

Expected protons=40
Whole protons observed=39
Total integral =40.267

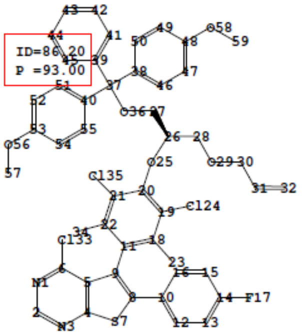

HoseCodePredictions

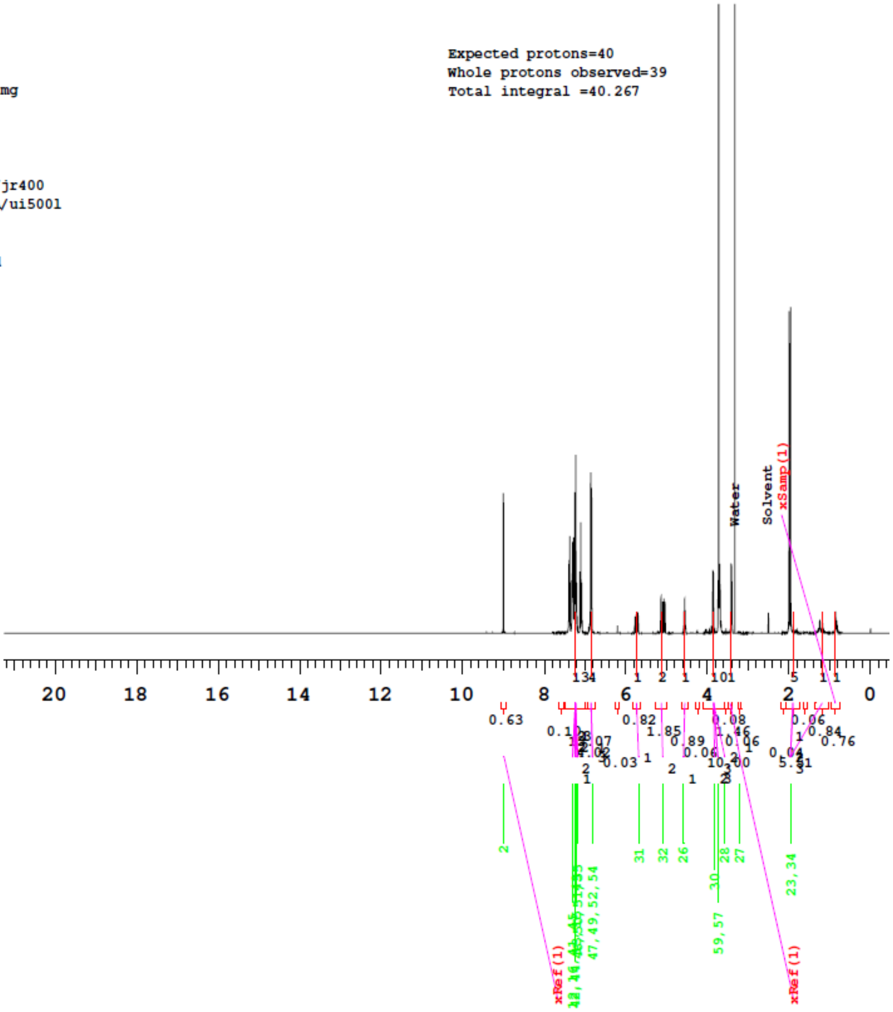

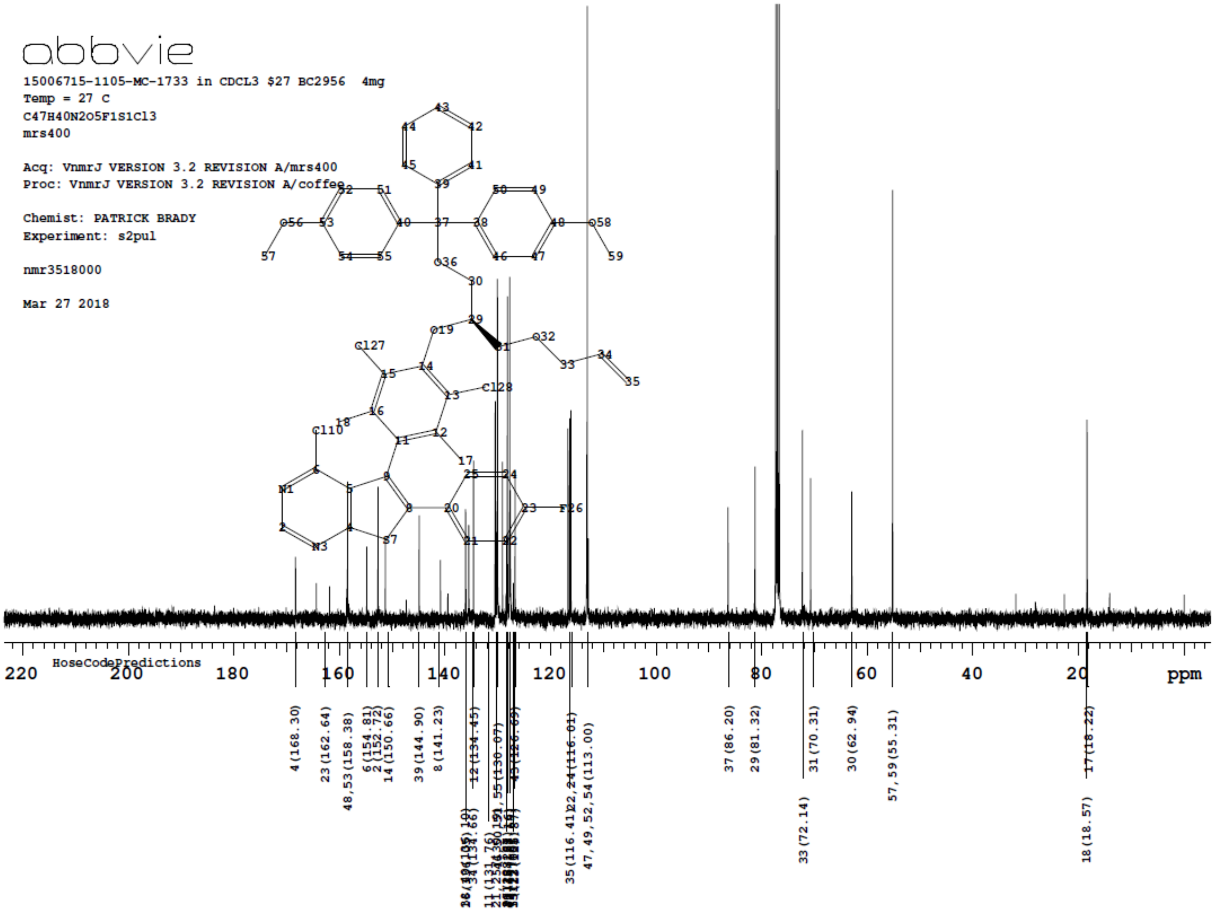
abbvie
15006715-1105-MC-1733 in CDCL3 $27 BC2956 4mg
Temp = 27 C
C47H40N2O5F1S1Cl3
mrs400
Acq: VnmrJ VERSION 3.2 REVISION A/mrs400
Proc: VnmrJ VERSION 3.2 REVISION A/coffee
Chemist: PATRICK BRADY
Experiment: s2pul
nmr3518000
Mar 27 2018
HoseCodePredictions
220
200
180
160
140
120
100
80
60
40
20
ppm
4(168.30)
23(162.64)
48,53(158.38)
6(154.81)
2(152.72)
14(150.66)
39(144.90)
8(141.23)
12(134.45)
37(86.20)
29(81.32)
33(72.14)
31(70.31)
30(62.94)
57,59(55.31)
17(18.22)
18(18.57)
47,49,52,54(113.00)

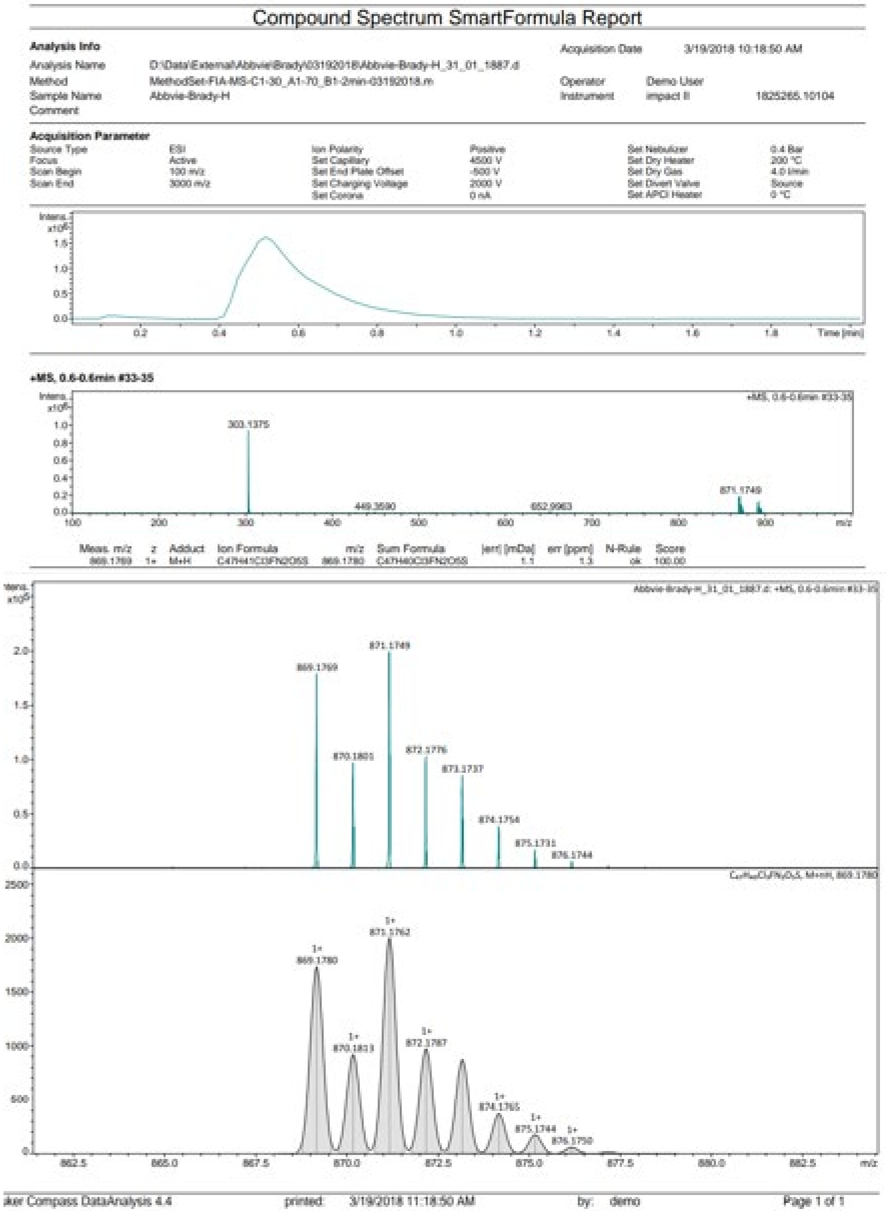

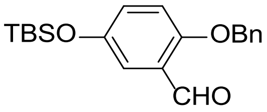

2-(benzyloxy)-5-((tert-butyldimethylsilyl)oxy)benzaldehyde (S-35).

abbvie

10008519-2328-CRUDE in DMSO BC#1740 $16
mrs400

Acq: VnmrJ VERSION 3.2 REVISION A/mrs400
Proc: VnmrJ VERSION 3.2 REVISION A/i600

Chemist: BRYAN SORENSEN
Experiment: s2pul

nmr3392079

Jan 16 2017

Expected protons=34
Whole protons observed=24
Total integral =24.563

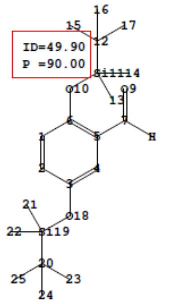

HoseCodePredictions

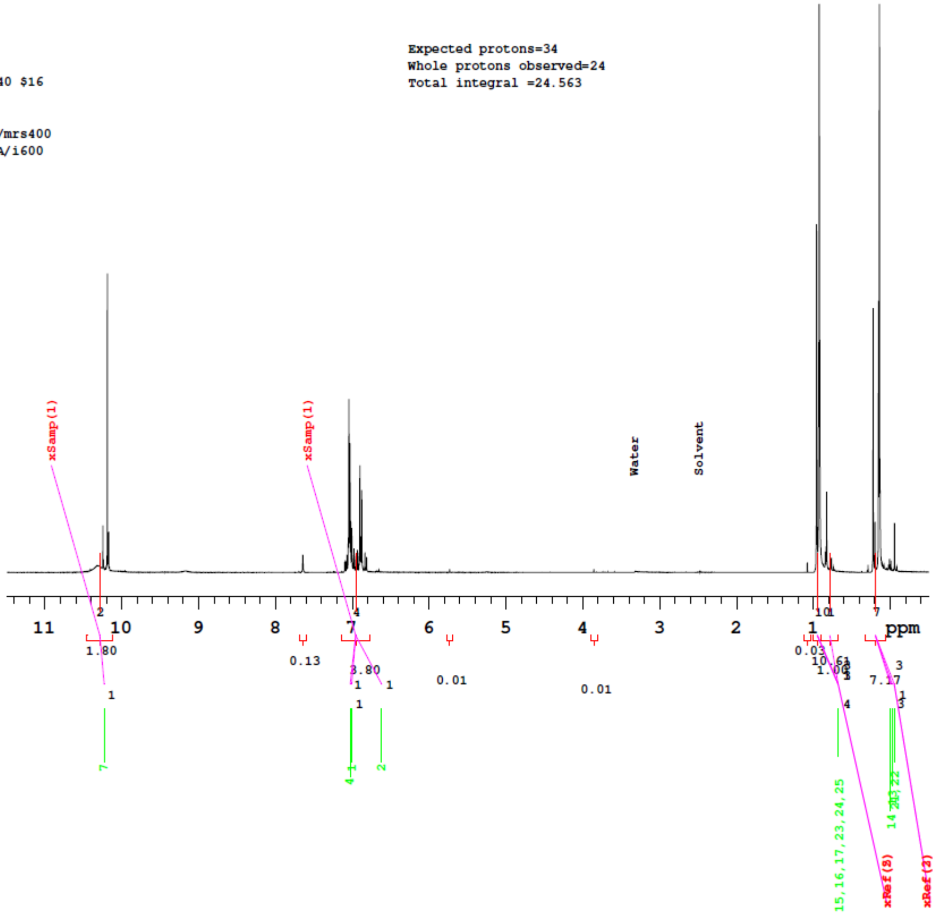

File: au86153x (17-Jan-2017 06:24:44) Scan: 29,31 - 23,21
Samp: 10008519-2328-CRUDE MS-au86153
Cmnt: LXQ/LC866450
Mode: +ESI Oper: AUTO Client:
Base: 367.19 Intensity: 267659
Formula: C19H34O3Si2 Expected Mass: 366.20 Score: 0.96

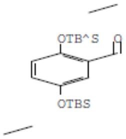

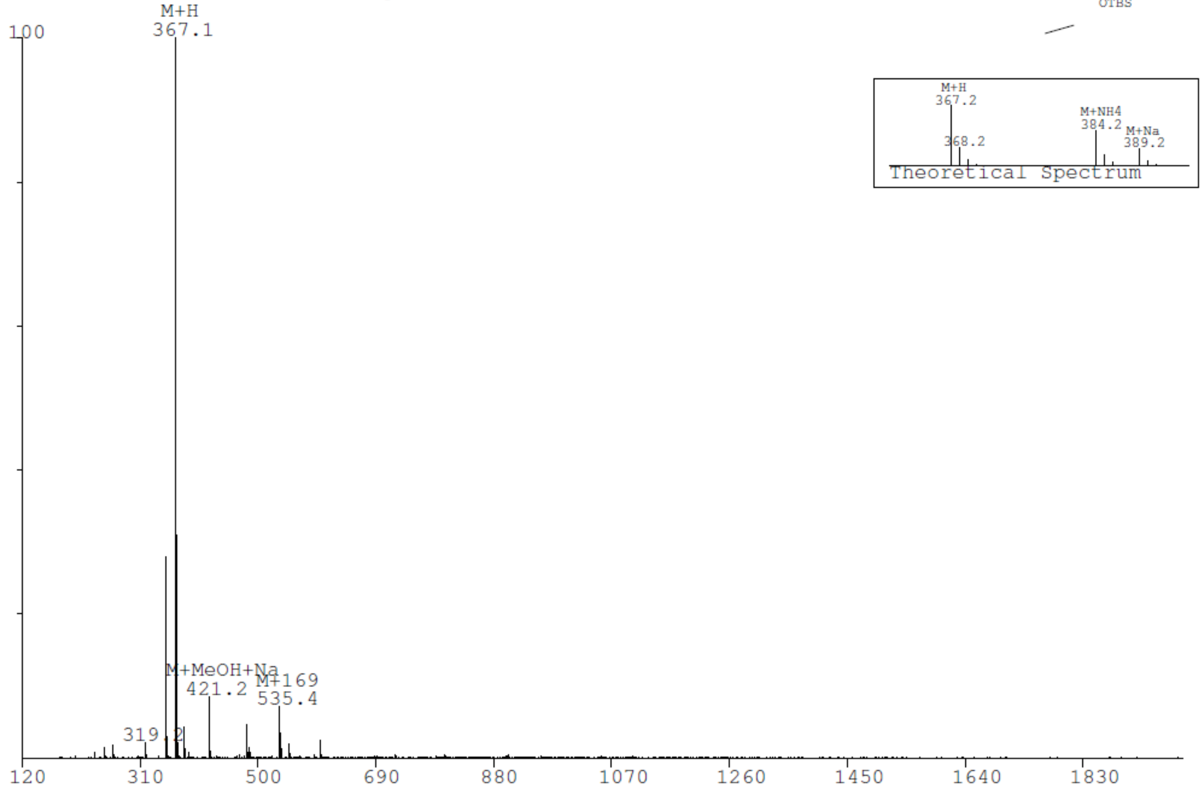

Date: Tue Jan 17 06:40:18 2017 Software: MSProcess 6.17

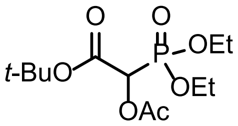

Tert-butyl 2-acetoxy-2-(diethoxyphosphoryl)acetate (S-36).

abbvie

15006715-688-B in CDCL3 $24 BC6118 20mg
Temp = 27 C
C12H23O7P1
mrs400

Acq: VnmrJ VERSION 3.2 REVISION A/mrs400
Proc: VnmrJ VERSION 3.2 REVISION A/m3001

Chemist: PATRICK BRADY
Experiment: s2pul

nmr3474821

Oct 24 2017

Expected protons=23
Whole protons observed=14
Total integral =15.807

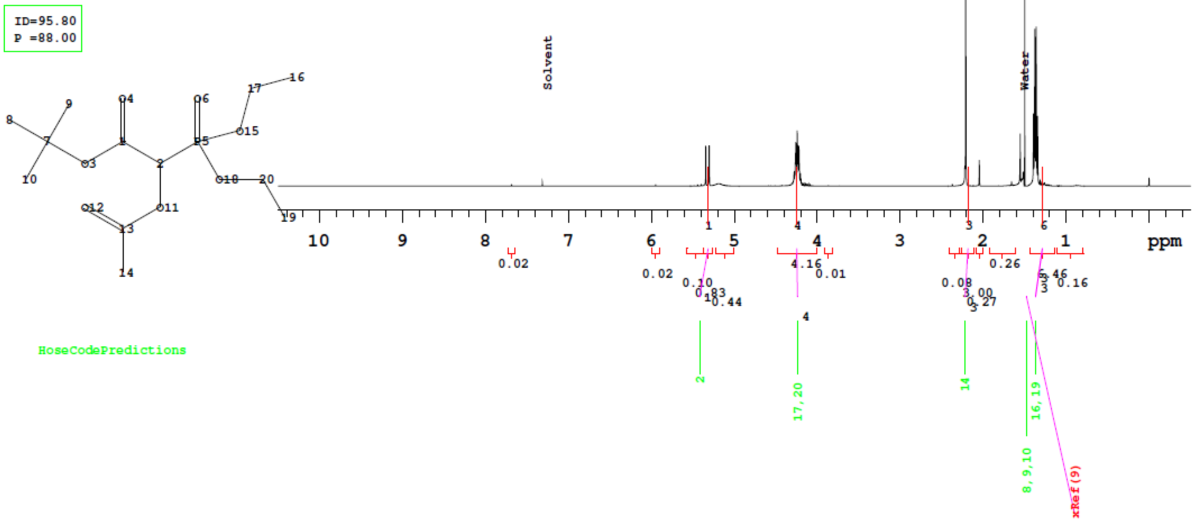

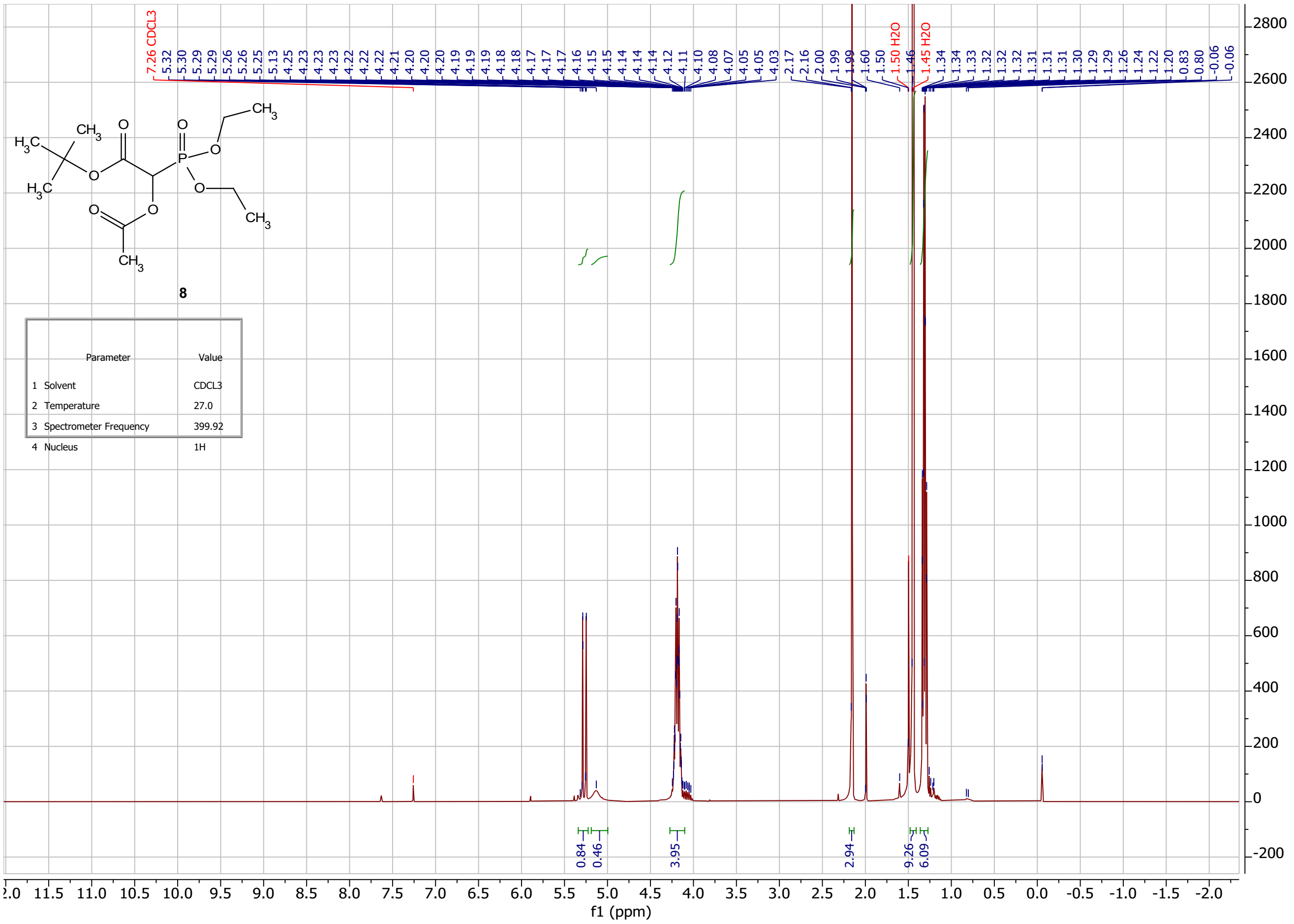

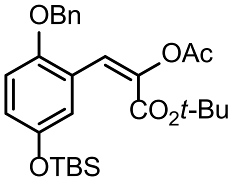

(E)-tert-butyl 2-acetoxy-3-(2-(benzyloxy)-5-((tert-butyldimethylsilyl)oxy)phenyl)acrylate (S-37).

abbvie

10012959-2654-COMBINED in CDCL3 $10 BC6132 4mg
Temp = 27 C
C28H38O6Si1
mrs400

Acq: VnmrJ VERSION 3.2 REVISION A/mrs400
Proc: VnmrJ VERSION 3.2 REVISION A/ui5001

Chemist: ALAN FLORJANCIC
Experiment: s2pul

nmr3426310

May 10 2017

Expected protons=38
Whole protons observed=32
Total integral =33.646

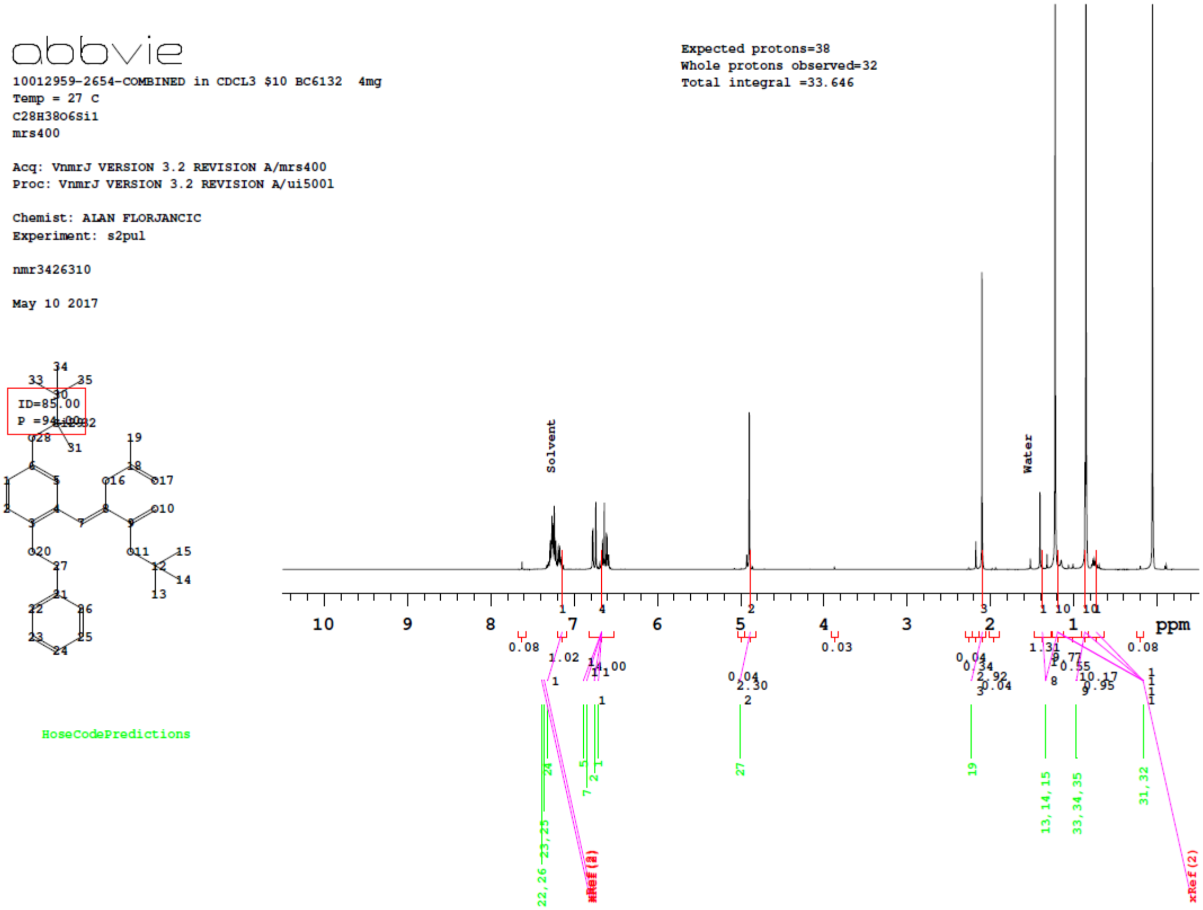

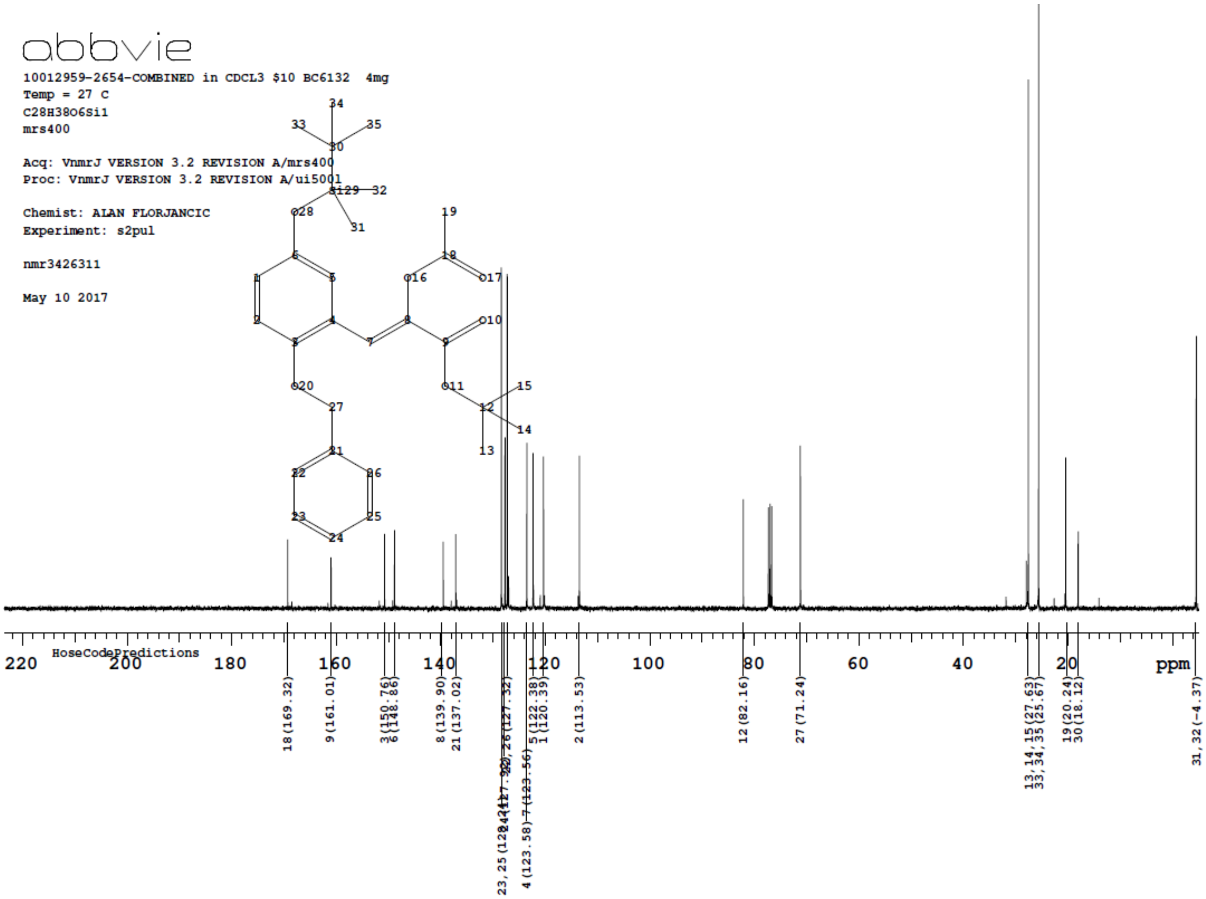
abbvie
10012959-2654-COMBINED in CDCL3 $10 BC6132 4mg
Temp = 27 C
C28H38O6Si1
mrs400
Acq: VnmrJ VERSION 3.2 REVISION A/mrs400
Proc: VnmrJ VERSION 3.2 REVISION A/ui5001
Chemist: ALAN FLORJANCIC
Experiment: s2pul
nmr3426311
May 10 2017
HoseCodePredictions
ppm
18(169.32)
9(161.01)
3(150.76)
6(148.86)
8(139.90)
21(137.02)
5(122.38)
1(120.39)
2(113.53)
12(82.16)
27(71.24)
13,14,15(27.63)
33,34,35(25.67)
19(20.24)
30(18.12)
31,32(-4.37)

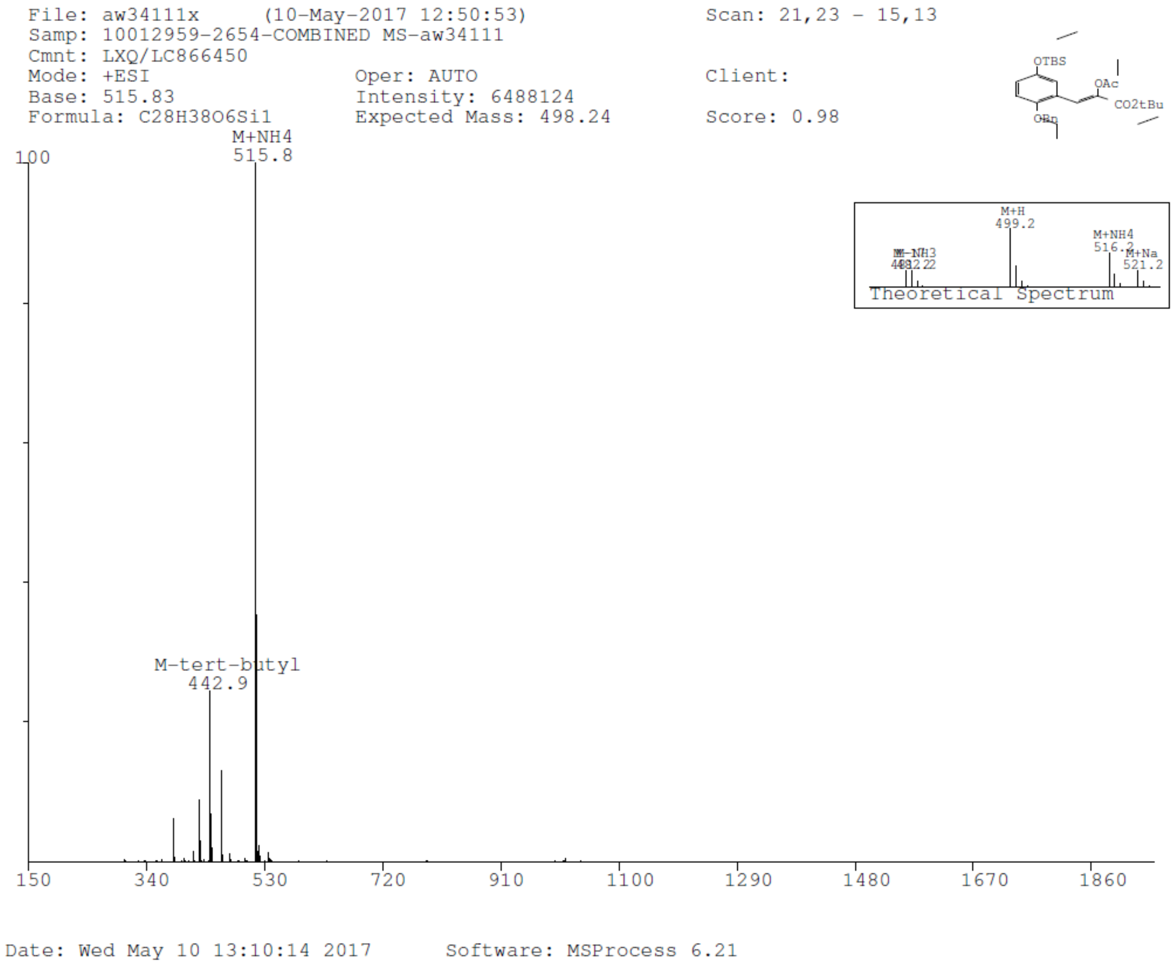

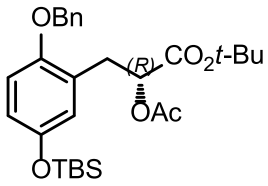

(R)-tert-butyl 2-acetoxy-3-(2-(benzyloxy)-5-((tert-butyldimethylsilyl)oxy)phenyl)propanoate (S-38).

abbvie

10012959-2656-LOT1 in CDCL3 $17 BC1138 3mg
Temp = 27 C
C28H40O6Si1
v501

Acq: VnmrJ VERSION 3.2 REVISION A/v501
Proc: VnmrJ VERSION 3.2 REVISION A/ui5001

Chemist: ALAN FLORJANCIC
Experiment: s2pul

nmr3428210

May 17 2017

Expected protons=40
Whole protons observed=33
Total integral =33.971

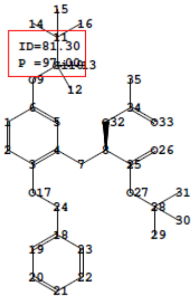

HoseCodePredictions

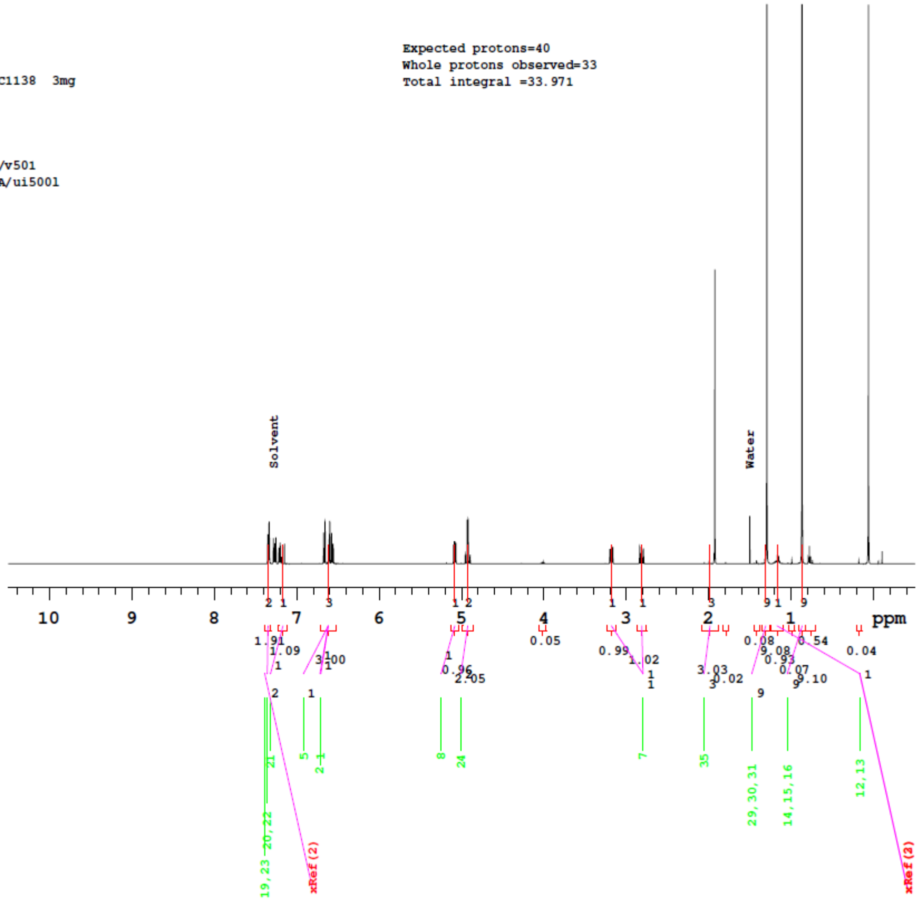

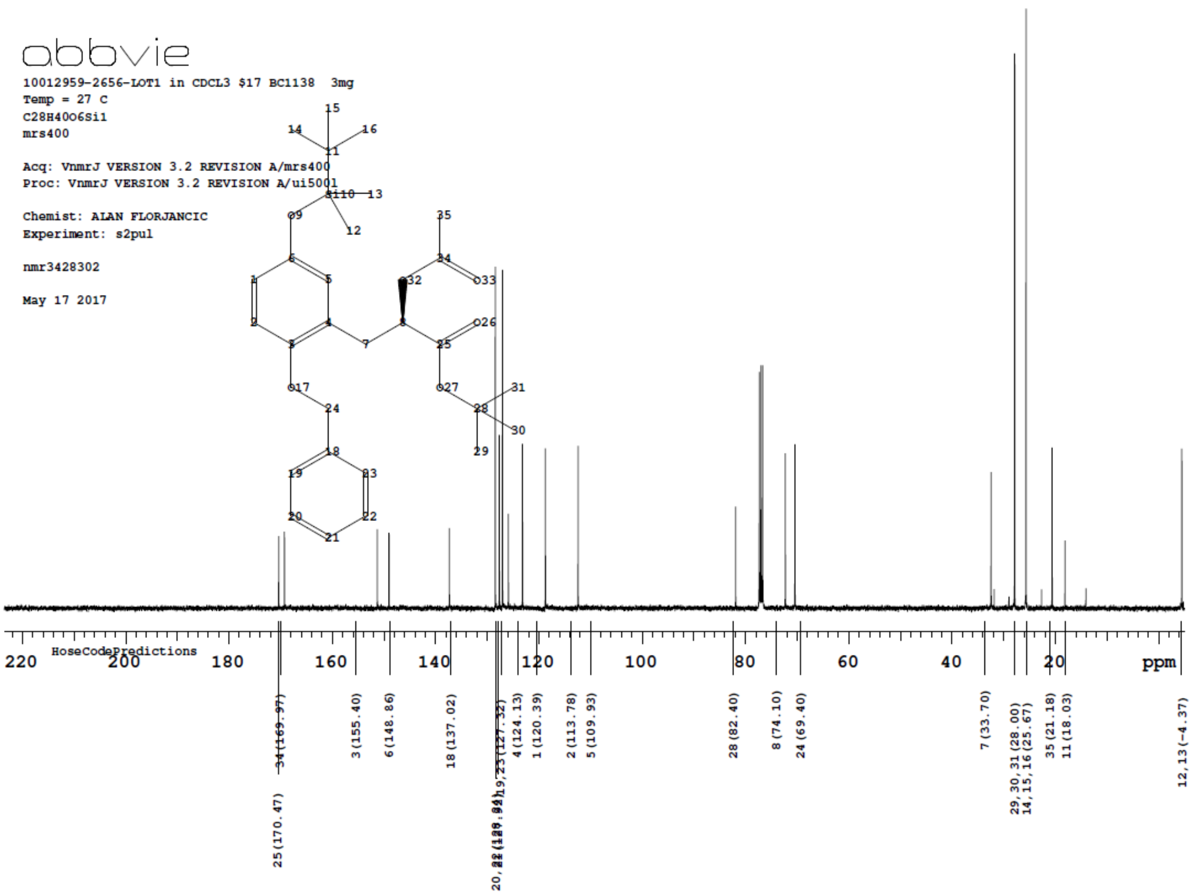
abbvie
10012959-2656-LOT1 in CDCL3 $17 BC1138 3mg
Temp = 27 C
C28H40O6Si1
mrs400
Acq: VnmrJ VERSION 3.2 REVISION A/mrs400
Proc: VnmrJ VERSION 3.2 REVISION A/ui5001
Chemist: ALAN FLORJANCIC
Experiment: s2pul
nmr3428302
May 17 2017
HoseCodePredictions
220 200 180 160 140 120 100 80 60 40 20 ppm
34(169.97)
25(170.47)
3(155.40)
6(148.86)
18(137.02)
4(124.13)
1(120.39)
2(113.78)
5(109.93)
28(82.40)
8(74.10)
24(69.40)
7(33.70)
29,30,31(28.00)
14,15,16(25.67)
35(21.18)
11(18.03)
12,13(-4.37)

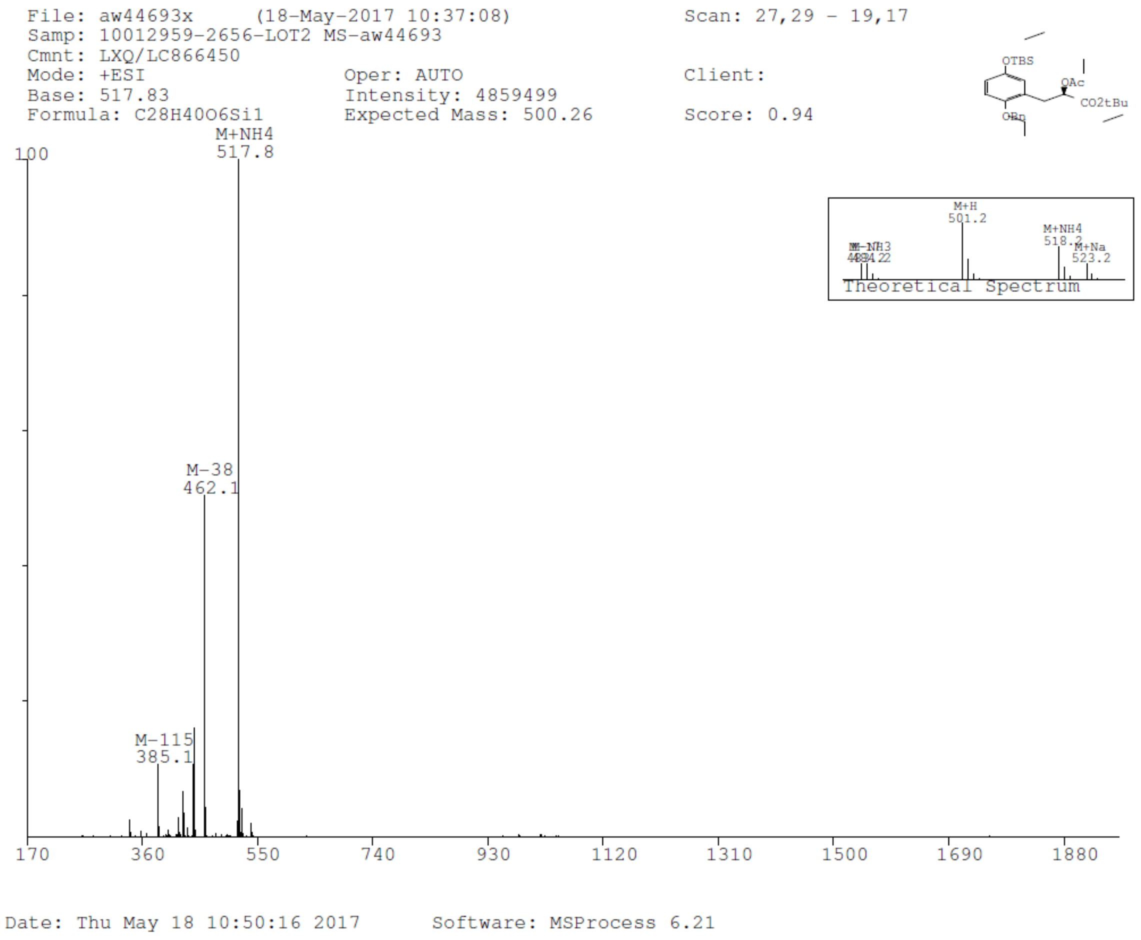

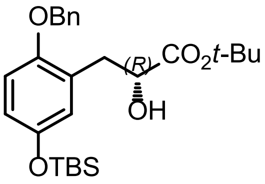

(R)-tert-butyl 3-(2-(benzyloxy)-5-((tert-butyldimethylsilyl)oxy)phenyl)-2-hydroxypropanoate (S-39

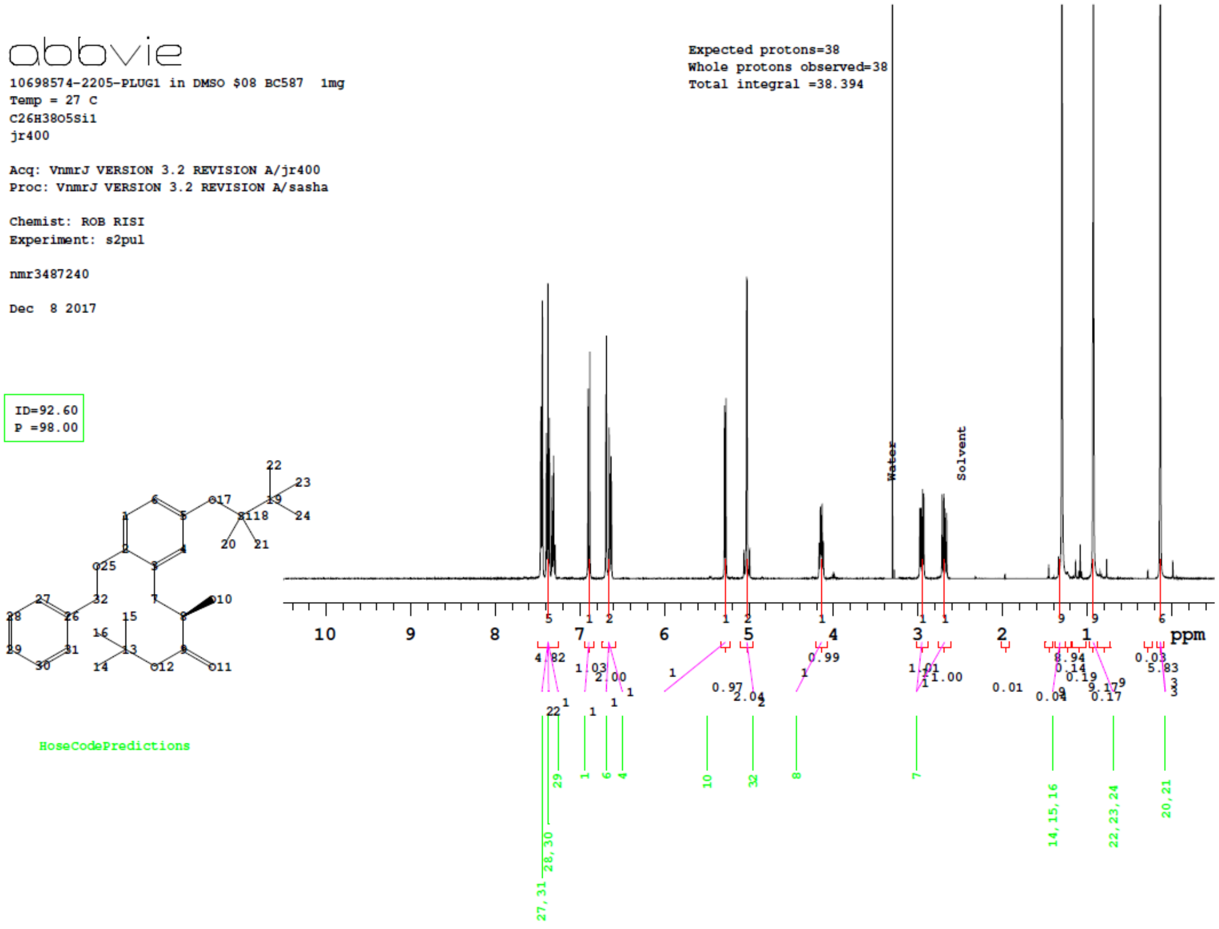
abbvie
10698574-2205-PLUG1 in DMSO $08 BC587 1mg
Temp = 27 C
C26H38O5Si1
jr400
Acq: VnmrJ VERSION 3.2 REVISION A/jr400
Proc: VnmrJ VERSION 3.2 REVISION A/sasha
Chemist: ROB RISI
Experiment: s2pul
nmr3487240
Dec 8 2017
ID=92.60
P =98.00
HoseCodePredictions
Expected protons=38
Whole protons observed=38
Total integral =38.394
Water
Solvent
ppm

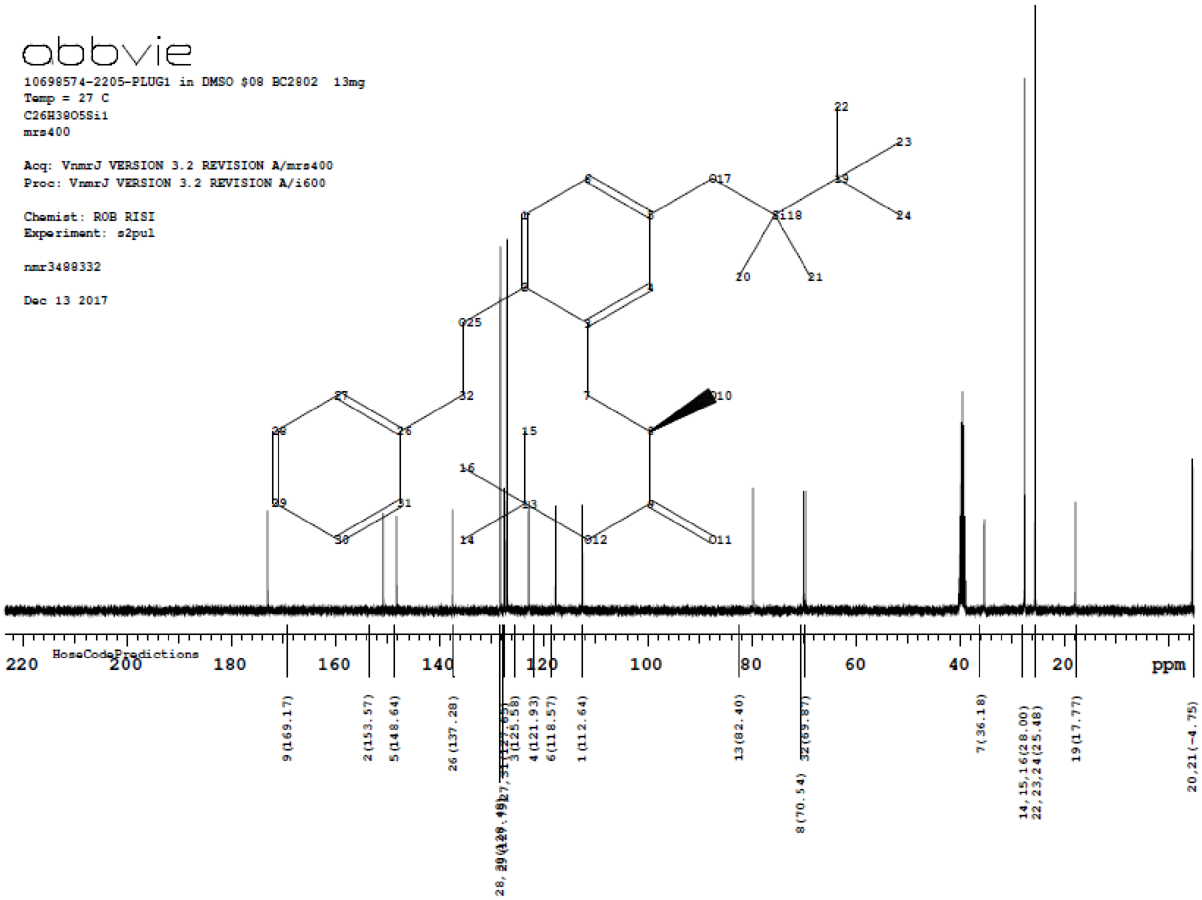

10698574-2205-PLUG1 in DMSO $08 BC2802 13mg
Temp = 27 C
C26H38O5Si1
mrs400
Acq: VnmrJ VERSION 3.2 REVISION A/mrs400
Proc: VnmrJ VERSION 3.2 REVISION A/i600
Chemist: ROB RISI
Experiment: s2pul
nmr3488332
Dec 13 2017
HoseCodePredictions
ppm
9(169.17)
2(153.57)
5(148.64)
26(137.28)
3(125.58)
4(121.93)
6(118.57)
1(112.64)
13(82.40)
32(69.87)
8(70.54)
7(36.18)
14,15,16(28.00)
22,23,24(25.48)
19(17.77)
20,21(-4.75)

**Sample Name**	AB	**Position**	P2-B2	**Instrument Name**	Instrument 1	**User Name**	
**Inj Vol**	0.1	**InjPosition**		**SampleType**	Sample	**IRM Calibration Status**	Success
**Data Filename**	AB.d	**ACQ Method**	IMSERC_ESI_Pos_Main_	**Comment**		**Acquired Time**	5/1/2018 5:32:44 PM

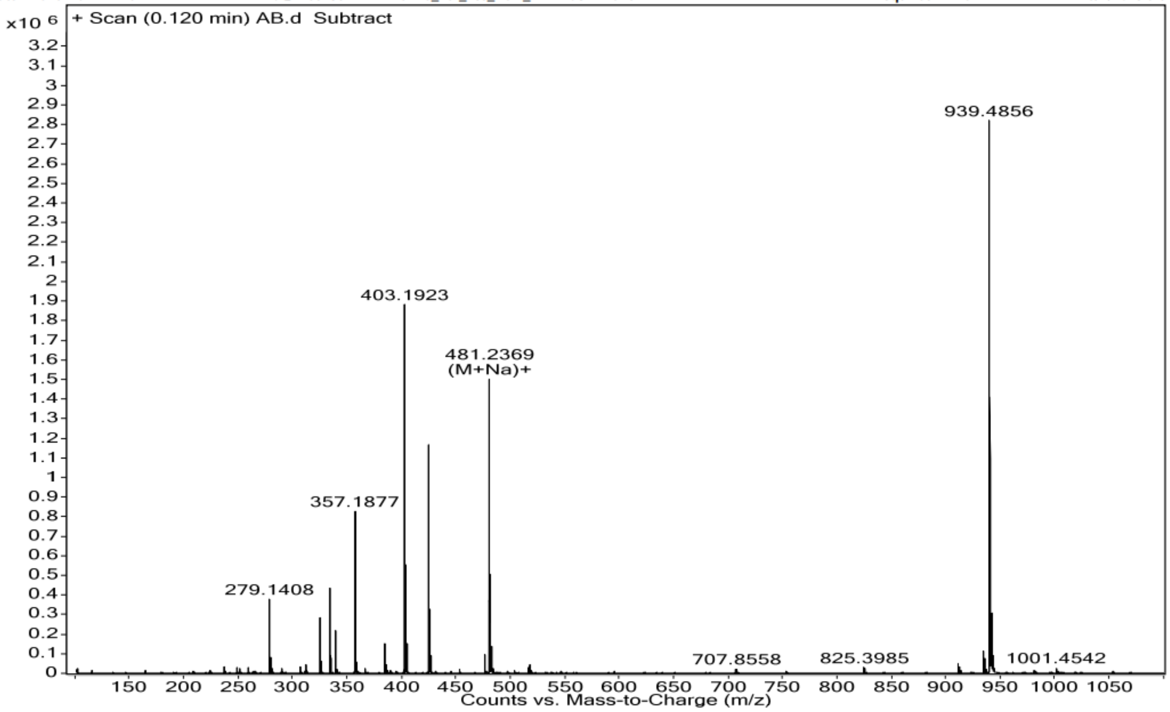

**Sample Name**	AB	**Position**	P2-B2	**Instrument Name**	Instrument 1	**User Name**	
**Inj Vol**	0.1	**InjPosition**		**SampleType**	Sample	**IRM Calibration Status**	Success
**Data Filename**	AB.d	**ACQ Method**	IMSERC_ESI_Pos_Main_	**Comment**		**Acquired Time**	5/1/2018 5:32:44 PM

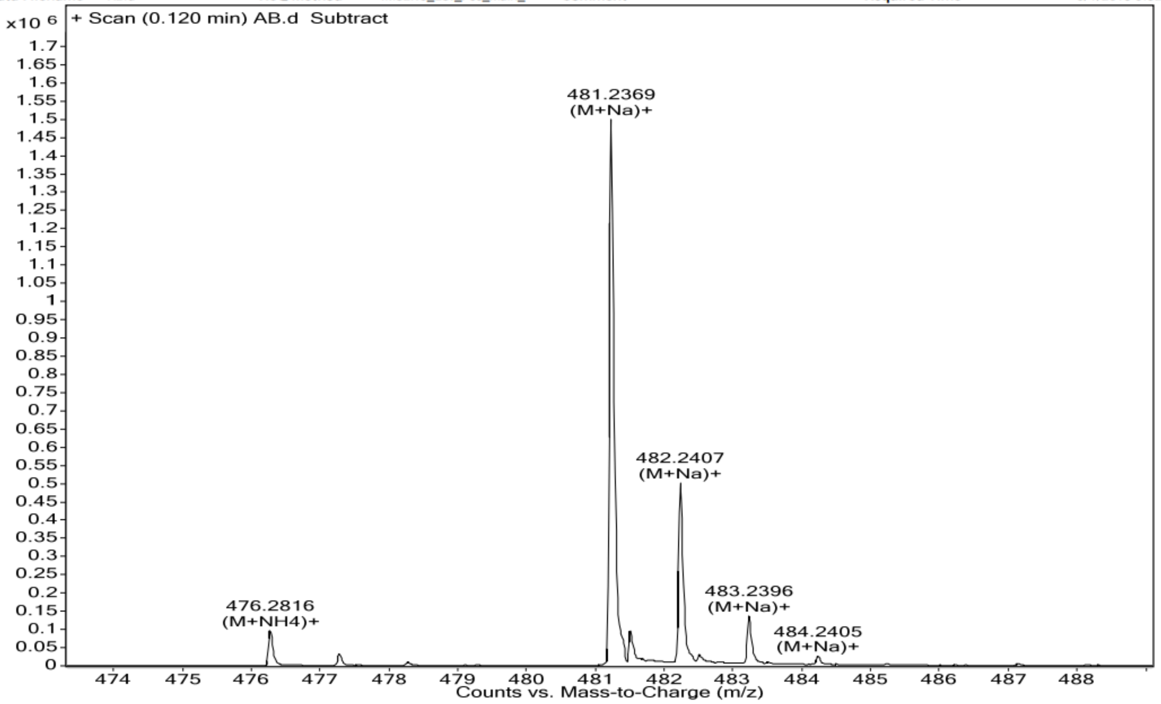

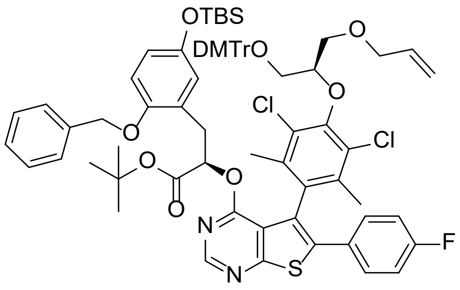

Tert-butyl (R)-2-((5-(4-(((R)-1-(allyloxy)-3-(bis(4-methoxyphenyl)(phenyl)methoxy)propan-2-yl)oxy)-3,5-dichloro-2,6-dimethylphenyl)-6-(4-fluorophenyl)thieno[2,3-d]pyrimidin-4-yl)oxy)-3-(2-(benzyloxy)-5-((tert-butyldimethylsilyl)oxy)phenyl)propanoate (S-40).

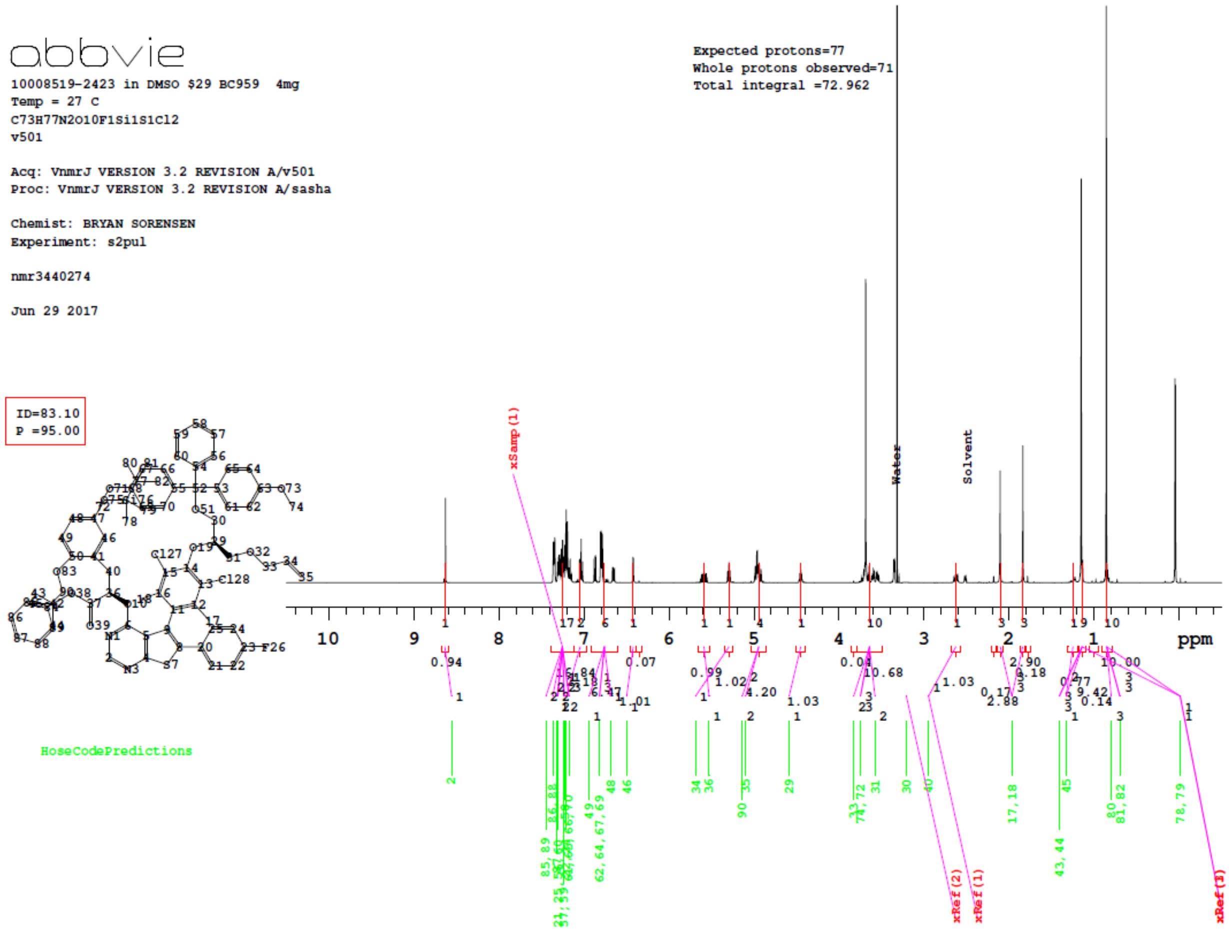

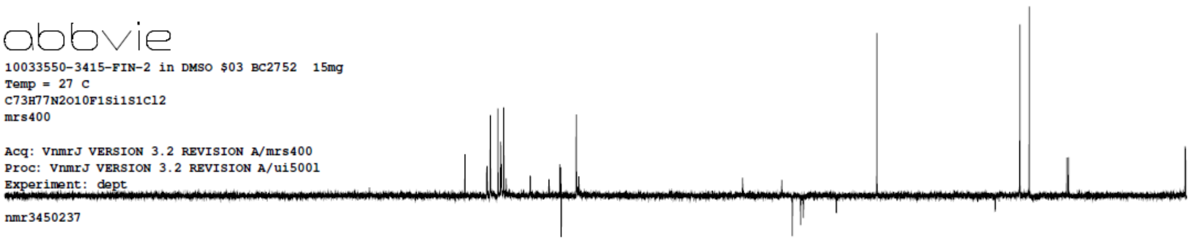
abbvie
10033550-3415-FIN-2 in DMSO $03 BC2752 15mg
Temp = 27 C
C73H77N2O10F1Si1S1Cl2
mrs400
Acq: VnmrJ VERSION 3.2 REVISION A/mrs400
Proc: VnmrJ VERSION 3.2 REVISION A/ui5001
Experiment: dept
nmr3450237
Aug 3 2017

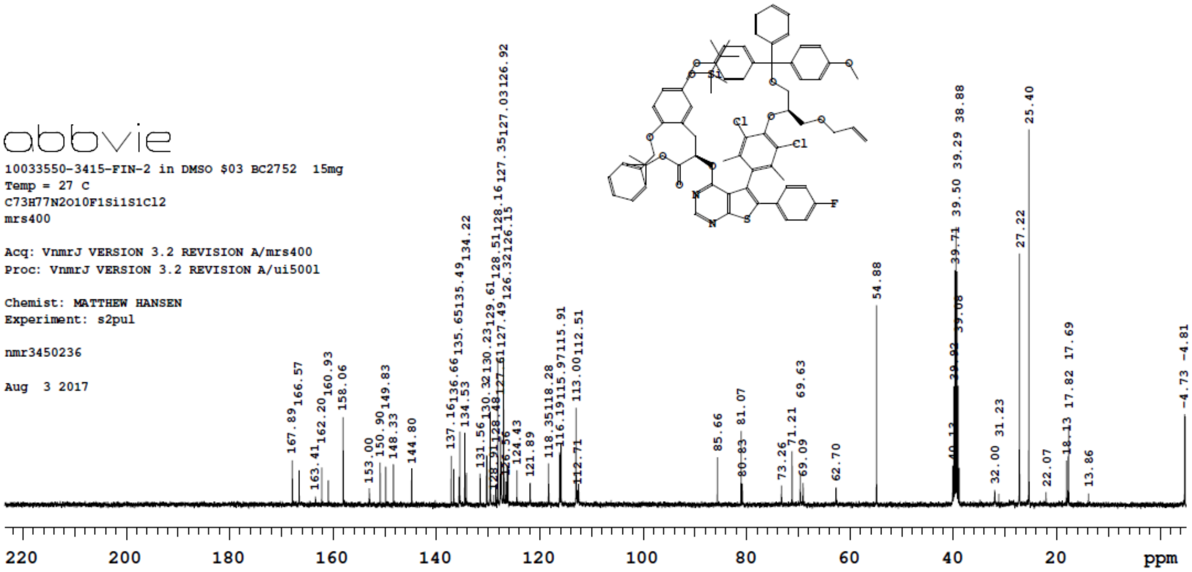
abbvie
10033550-3415-FIN-2 in DMSO $03 BC2752 15mg
Temp = 27 C
C73H77N2O10F1Si1S1Cl2
mrs400
Acq: VnmrJ VERSION 3.2 REVISION A/mrs400
Proc: VnmrJ VERSION 3.2 REVISION A/ui5001
Chemist: MATTHEW HANSEN
Experiment: s2pul
nmr3450236
Aug 3 2017
Cl
Cl
F
N
N
S
Si
ppm
220
200
180
160
140
120
100
80
60
40
20

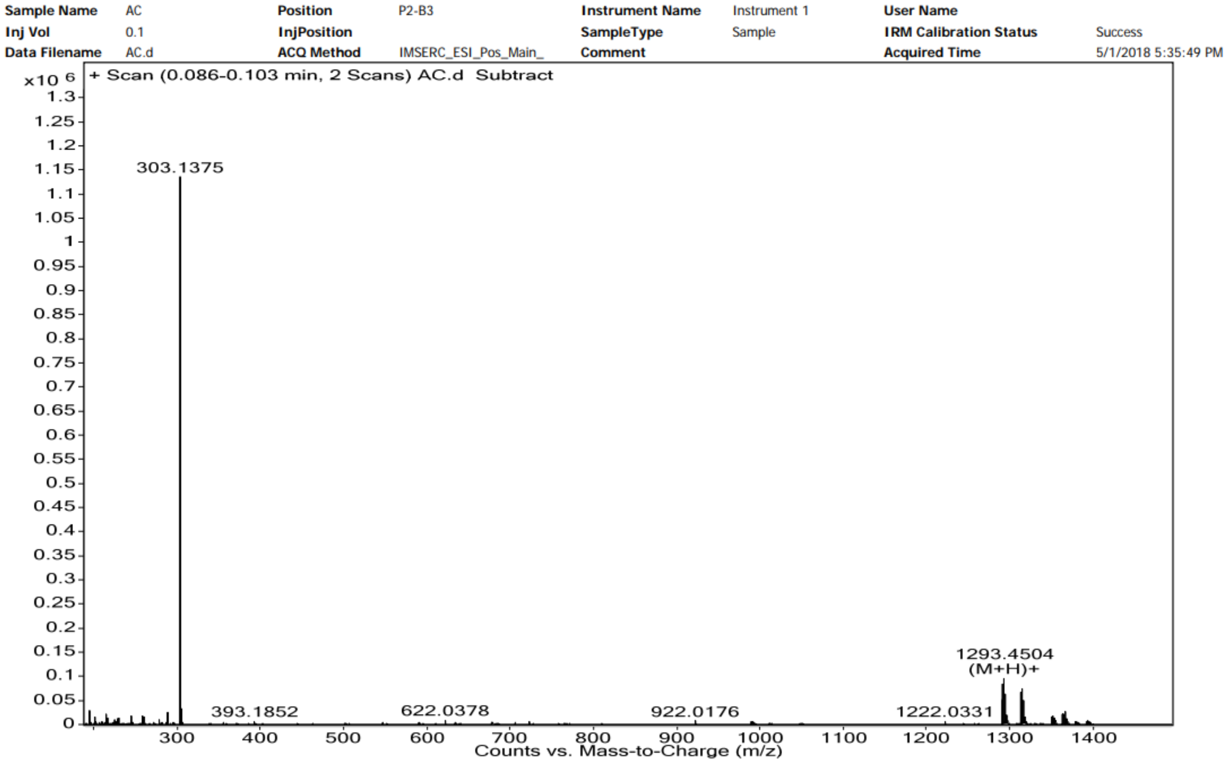
Sample Name AC
Inj Vol 0.1
Data Filename AC.d
Position P2-B3
InjPosition
ACQ Method IMSERC_ESI_Pos_Main_
Instrument Name Instrument 1
SampleType Sample
Comment
User Name
IRM Calibration Status Success
Acquired Time 5/1/2018 5:35:49 PM
+ Scan (0.086-0.103 min, 2 Scans) AC.d Subtract
x10 6
303.1375
393.1852
622.0378
922.0176
1222.0331
1293.4504
(M+H)+
Counts vs. Mass-to-Charge (m/z)

Sample Name	AC	Position	P2-B3	Instrument Name	Instrument 1	User Name	
Inj Vol	0.1	InjPosition		SampleType	Sample	IRM Calibration Status	Success
Data Filename	AC.d	ACQ Method	IMSERC_ESI_Pos_Main_	Comment		Acquired Time	5/1/2018 5:35:49 PM

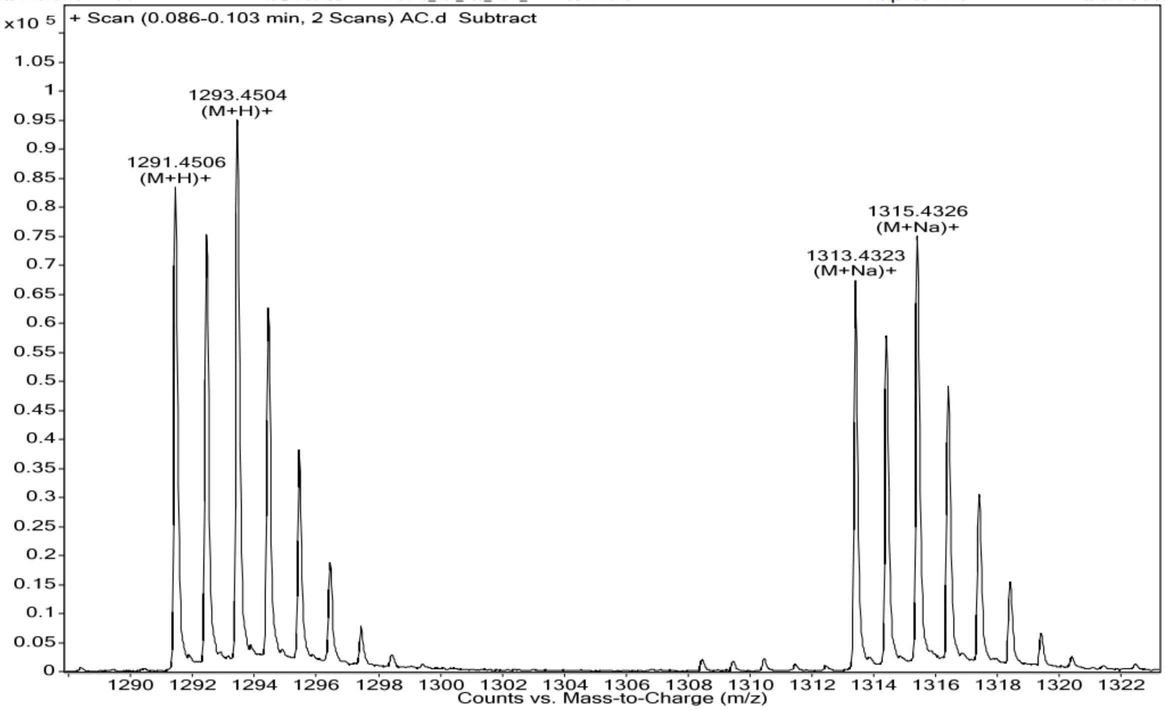

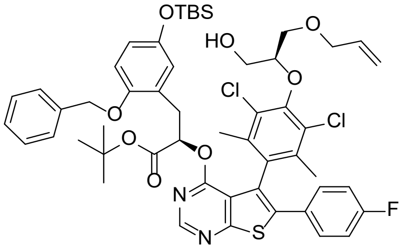

Tert-butyl (R)-2-((5-(4-(((S)-1-(allyloxy)-3-hydroxypropan-2-yl)oxy)-3,5-dichloro-2,6-dimethylphenyl)-6-(4-fluorophenyl)thieno[2,3-d]pyrimidin-4-yl)oxy)-3-(2-(benzyloxy)-5-((tert-butyldimethylsilyl)oxy)phenyl)propanoate (S-41).

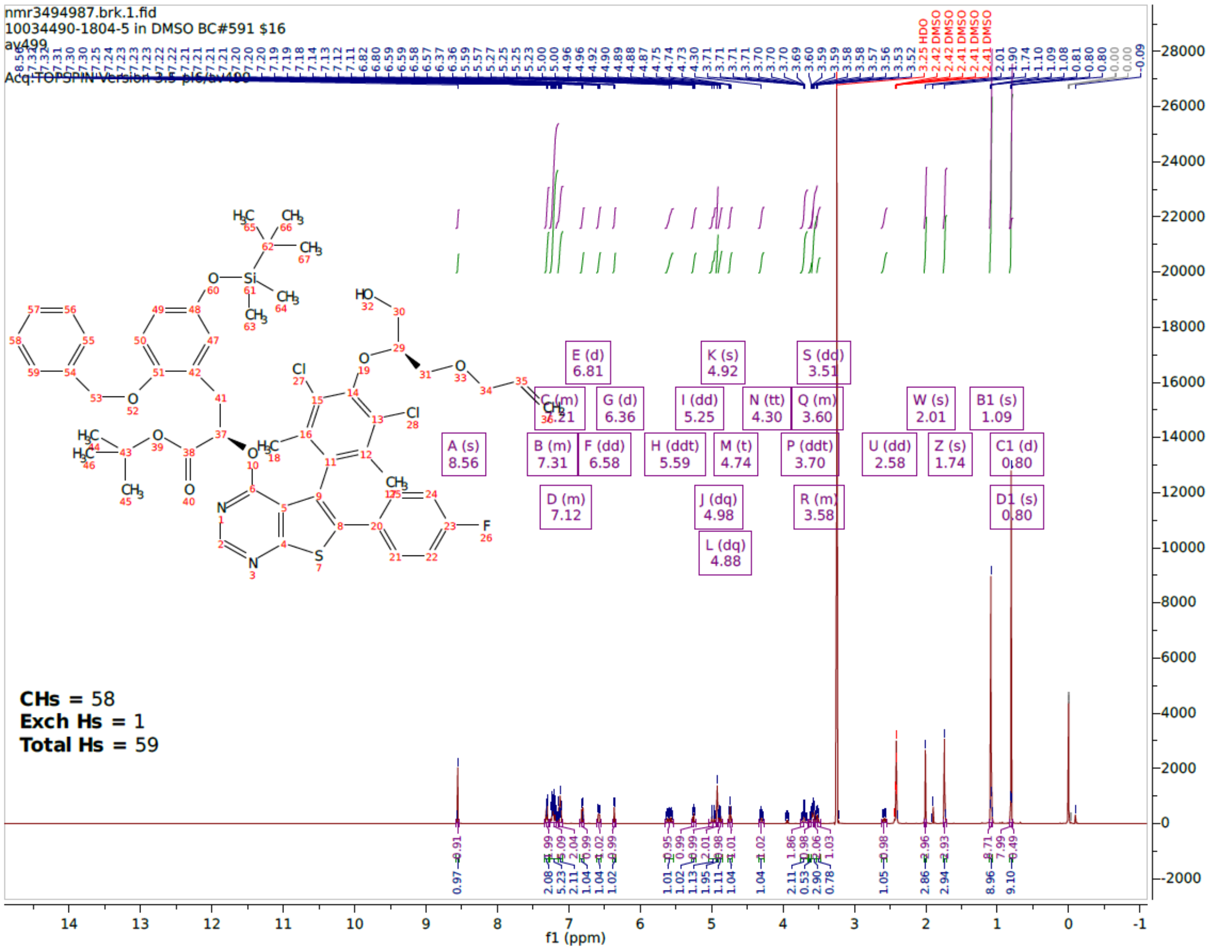
nmr3494987.brk.1.fid
10034490-1804-5 in DMSO BC#591 $16
av499
Acq:TOPSPIN Version 3.5 pl6/av400
CHs = 58
Exch Hs = 1
Total Hs = 59
A (s) 8.56
B (m) 7.31
C (m) 7.21
D (m) 7.12
E (d) 6.81
F (dd) 6.58
G (d) 6.36
H (ddt) 5.59
I (dd) 5.25
J (dq) 4.98
K (s) 4.92
L (dq) 4.88
M (t) 4.74
N (tt) 4.30
P (ddt) 3.70
Q (m) 3.60
R (m) 3.58
S (dd) 3.51
U (dd) 2.58
W (s) 2.01
Z (s) 1.74
B1 (s) 1.09
C1 (d) 0.80
D1 (s) 0.80
f1 (ppm)

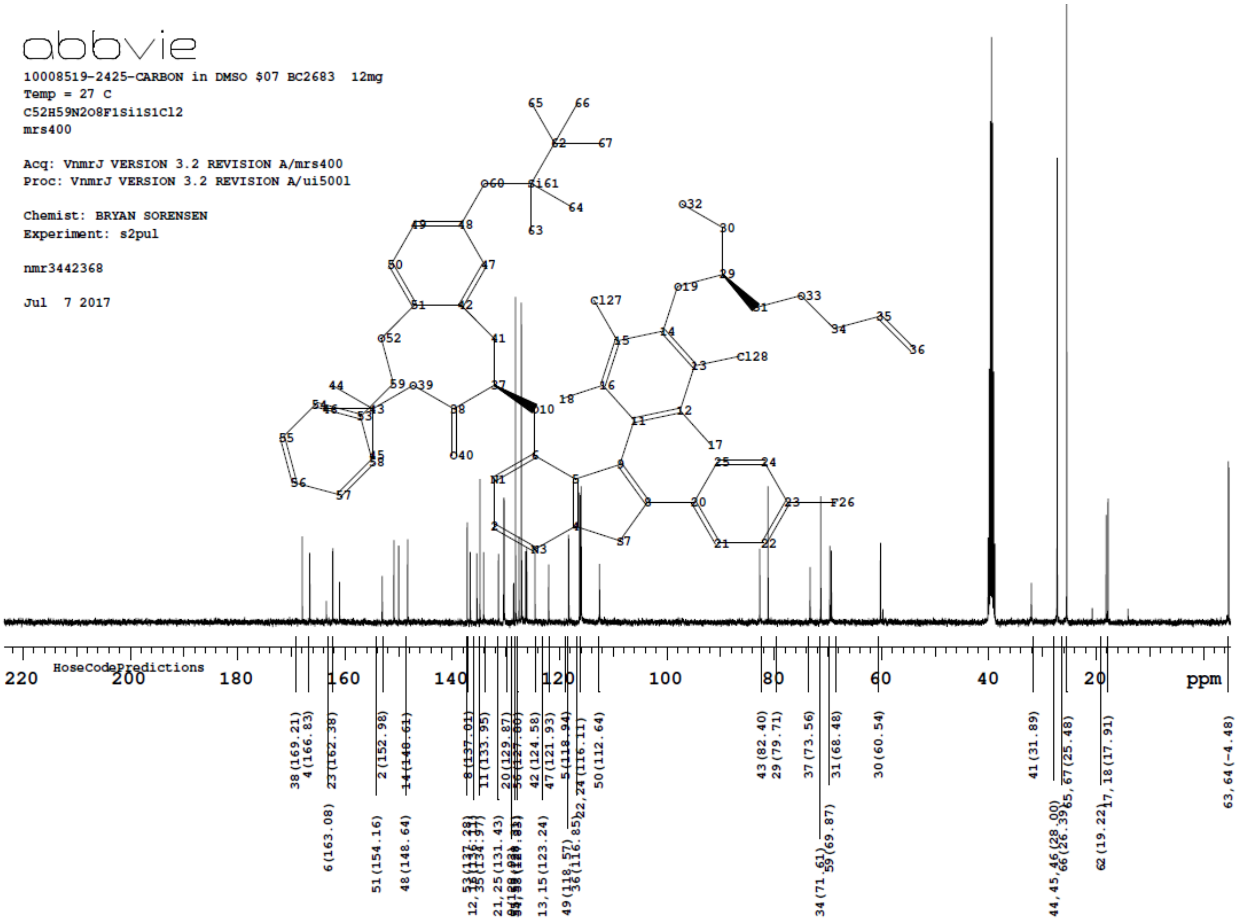
abbvie
10008519-2425-CARBON in DMSO $07 BC2683 12mg
Temp = 27 C
C52H59N2O8F1Si1S1Cl2
mrs400
Acq: VnmrJ VERSION 3.2 REVISION A/mrs400
Proc: VnmrJ VERSION 3.2 REVISION A/ui5001
Chemist: BRYAN SORENSEN
Experiment: s2pul
nmr3442368
Jul 7 2017
HoseCodePredictions
ppm

**Sample Name**	AD	**Position**	P2-B4	**Instrument Name**	Instrument 1	**User Name**	
**Inj Vol**	0.1	**InjPosition**		**SampleType**	Sample	**IRM Calibration Status**	Success
**Data Filename**	AD.d	**ACQ Method**	IMSERC_ESI_Pos_Main_	**Comment**		**Acquired Time**	5/1/2018 5:38:51 PM

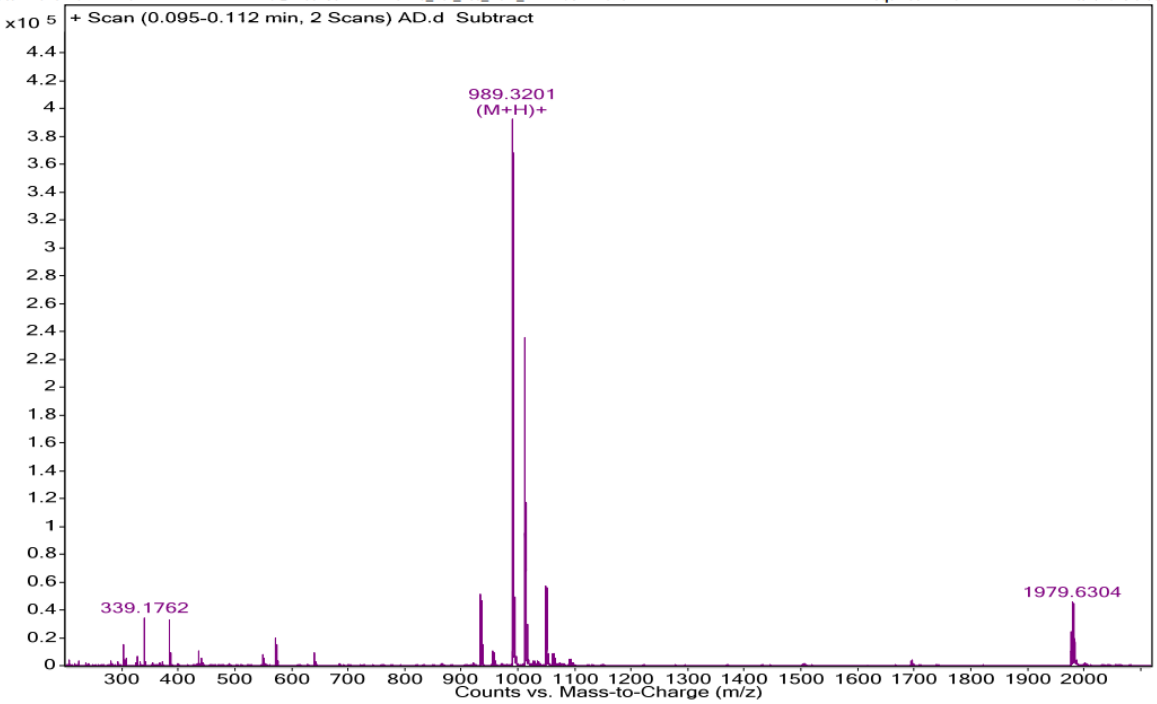

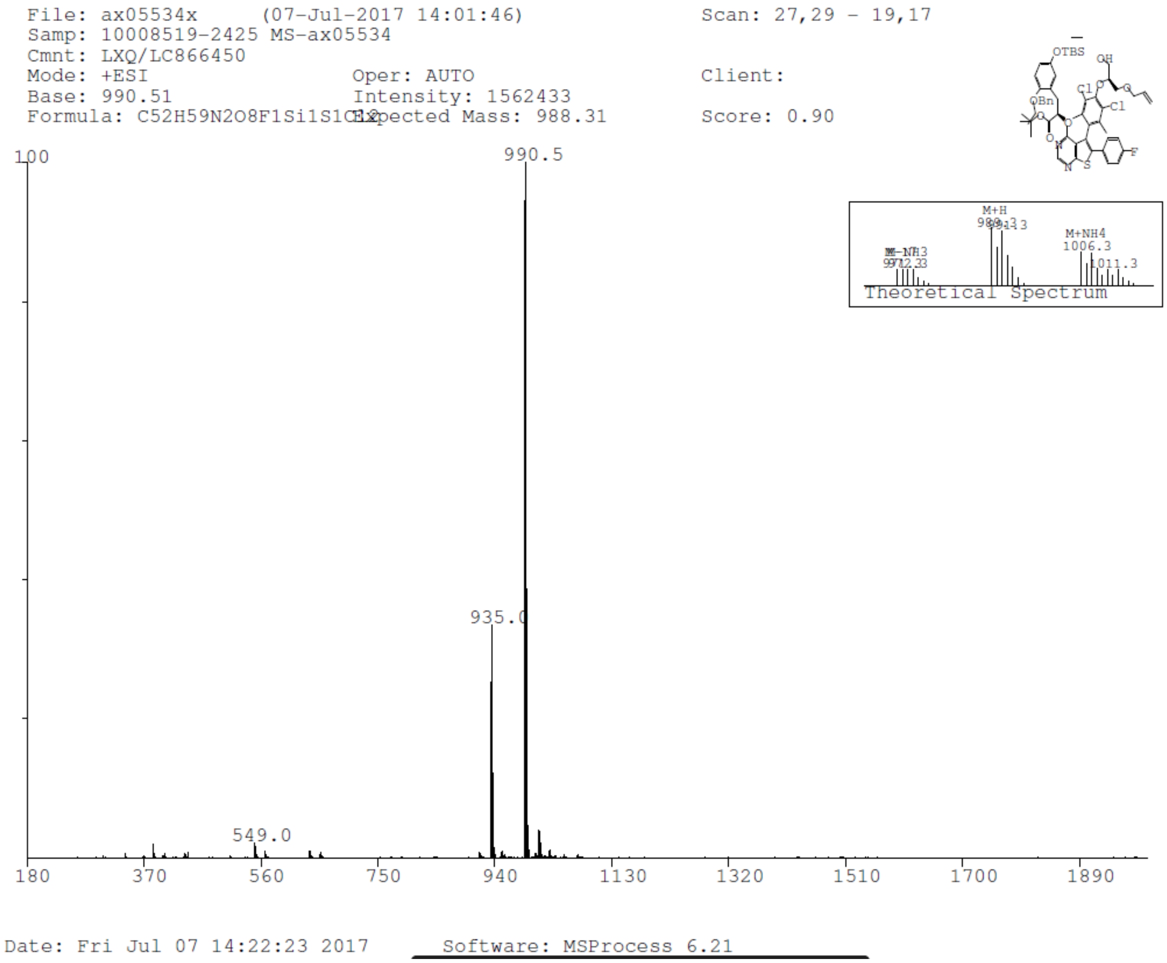

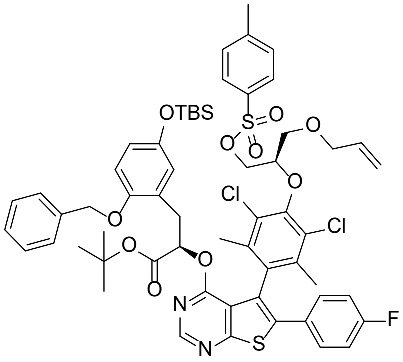

(R)-tert-butyl 2-((5-(4-(((R)-1-(allyloxy)-3-(tosyloxy)propan-2-yl)oxy)-3,5-dichloro-2,6-dimethylphenyl)-6-(4-fluorophenyl)thieno[2,3-d]pyrimidin-4-yl)oxy)-3-(2-(benzyloxy)-5-((tert-butyldimethylsilyl)oxy)phenyl)propanoate (S-42).

abbvie

10034490-1805-2 in DMSO $25 BC1641 2mg
Temp = 27 C
C59H65N2O10F1Si1S2Cl2
mrs400

Acq: VnmrJ VERSION 3.2 REVISION A/mrs400
Proc: VnmrJ VERSION 3.2 REVISION A/i600

Chemist: MICHAEL CURTIN
Experiment: s2pul

nmr3498209

Jan 26 2018

ID=86.00
P =97.00

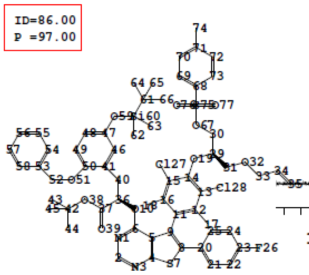

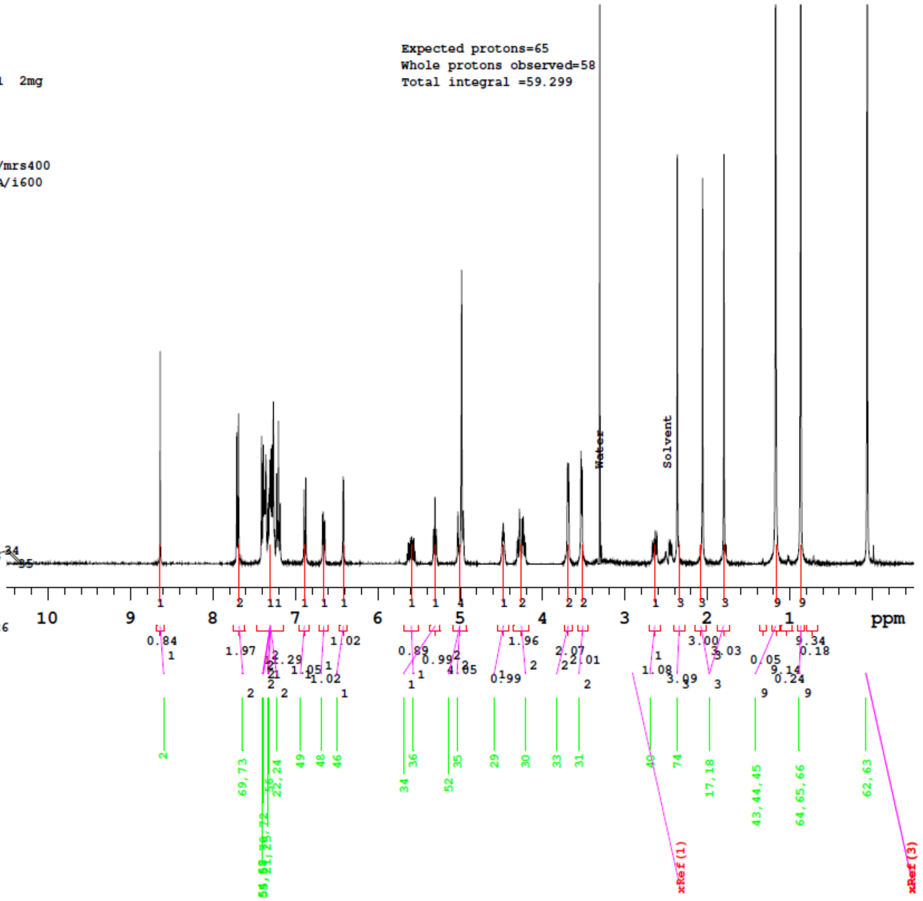

HoseCodePredictions

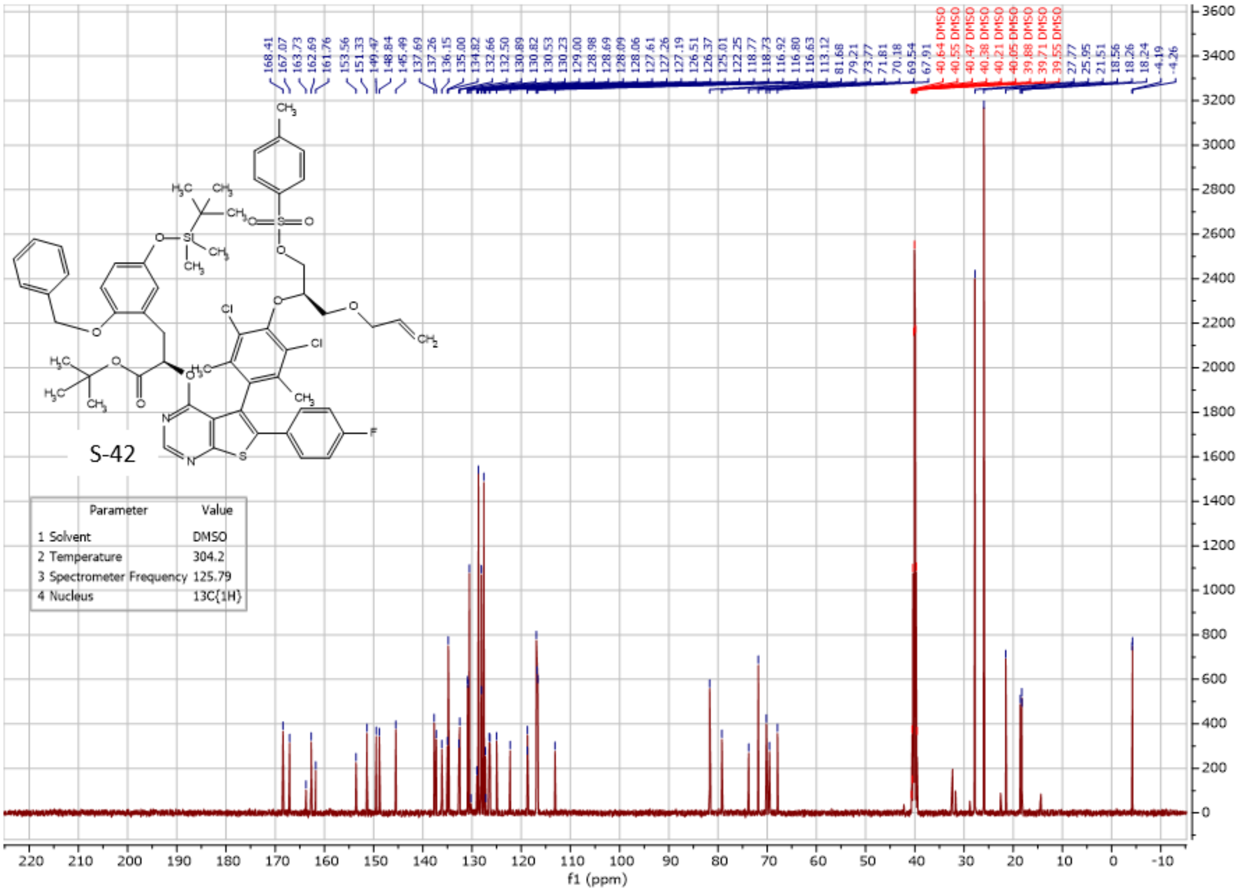

**Sample Name**	AE	**Position**	P2-B5	**Instrument Name**	Instrument 1	**User Name**	
**Inj Vol**	0.1	**InjPosition**		**SampleType**	Sample	**IRM Calibration Status**	Success
**Data Filename**	AE.d	**ACQ Method**	IMSERC_ESI_Pos_Main_	**Comment**		**Acquired Time**	5/1/2018 5:41:54 PM

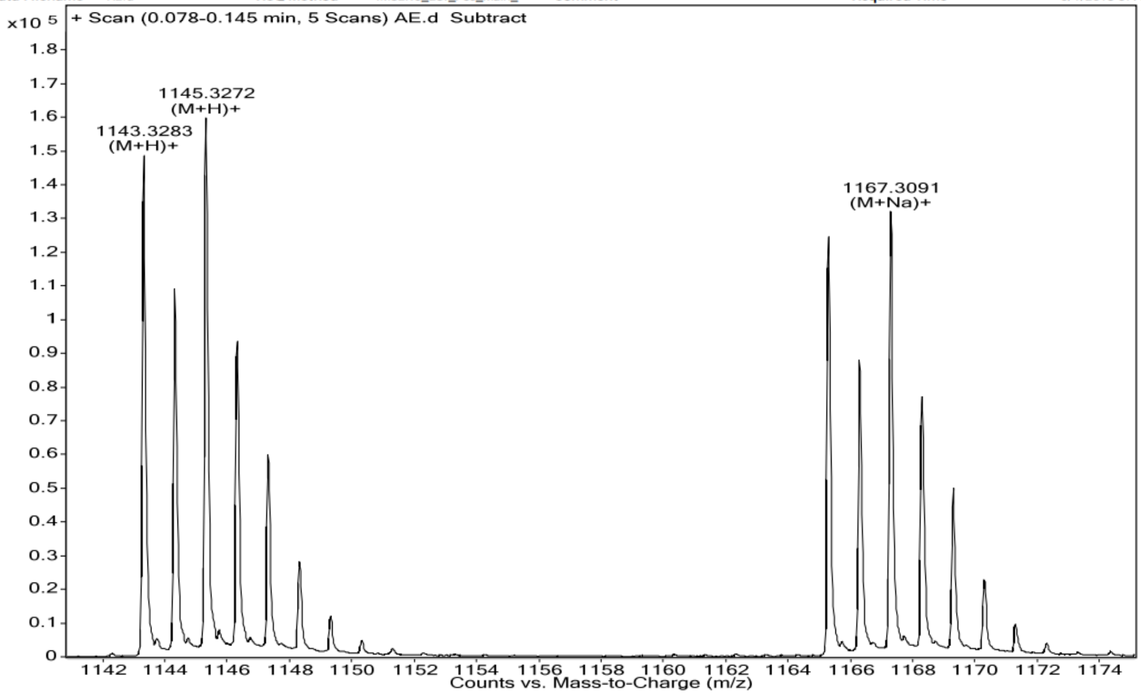

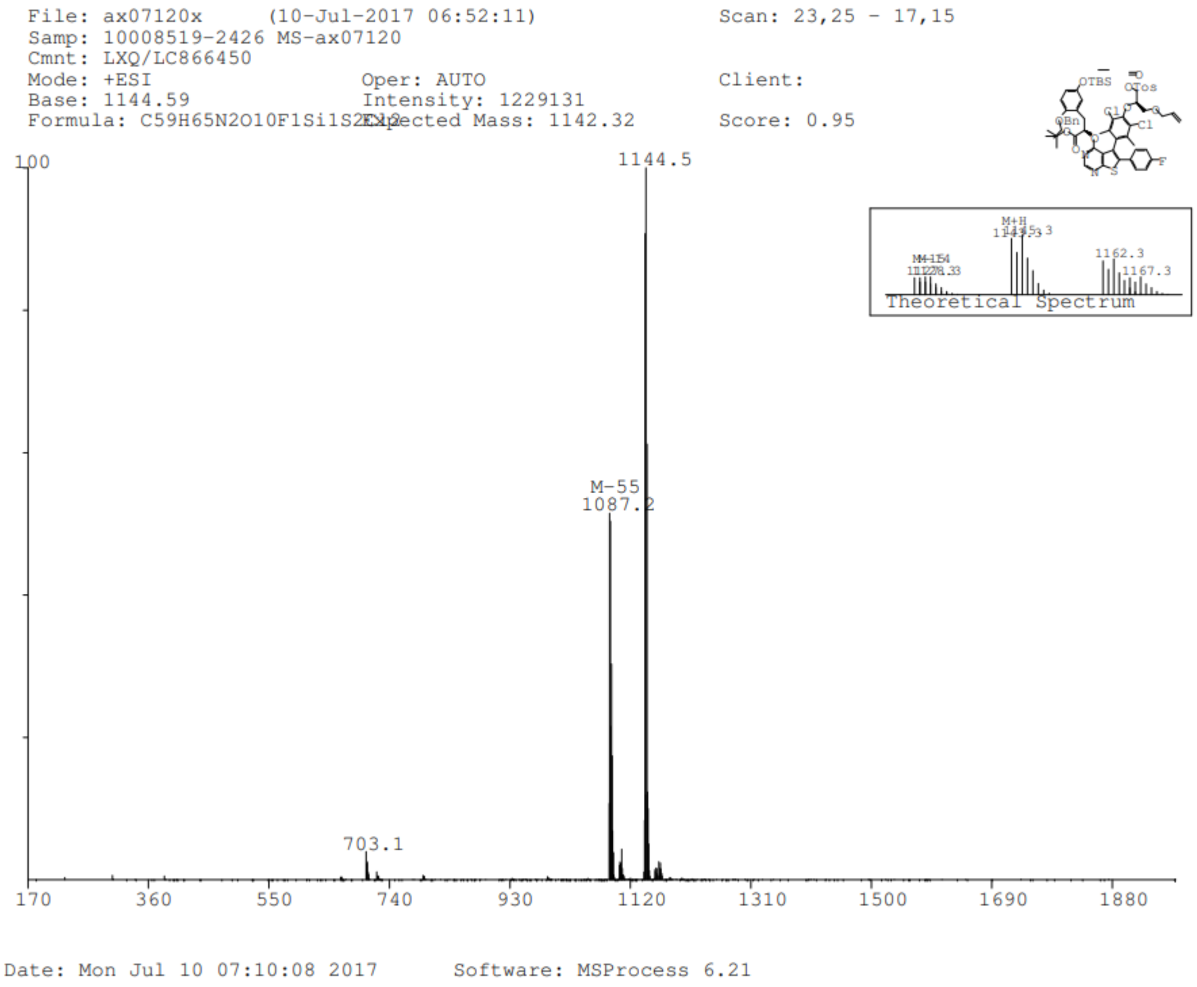

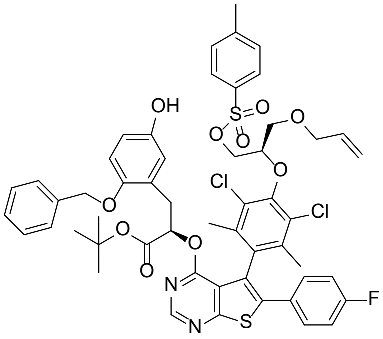

(R)-tert-butyl 2-((5-(4-(((R)-1-(allyloxy)-3-(tosyloxy)propan-2-yl)oxy)-3,5-dichloro-2,6-dimethylphenyl)-6-(4-fluorophenyl)thieno[2,3-d]pyrimidin-4-yl)oxy)-3-(2-(benzyloxy)-5-hydroxyphenyl)propanoate (S-43).

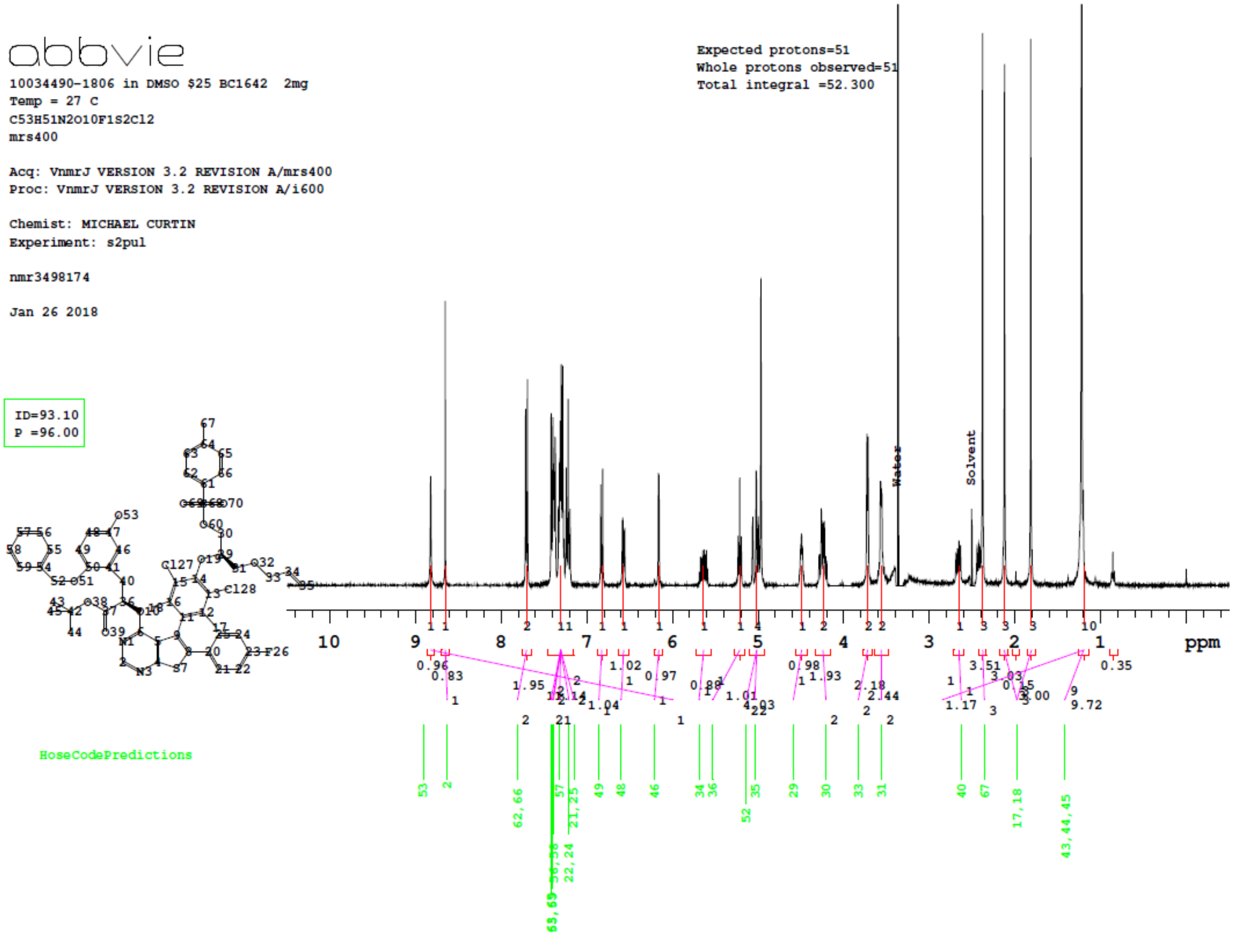
abbvie
10034490-1806 in DMSO $25 BC1642 2mg
Temp = 27 C
C53H51N2O10F1S2Cl2
mrs400
Acq: VnmrJ VERSION 3.2 REVISION A/mrs400
Proc: VnmrJ VERSION 3.2 REVISION A/i600
Chemist: MICHAEL CURTIN
Experiment: s2pul
nmr3498174
Jan 26 2018
ID=93.10
P =96.00
Expected protons=51
Whole protons observed=51
Total integral =52.300
Water
Solvent
ppm
HoseCodePredictions

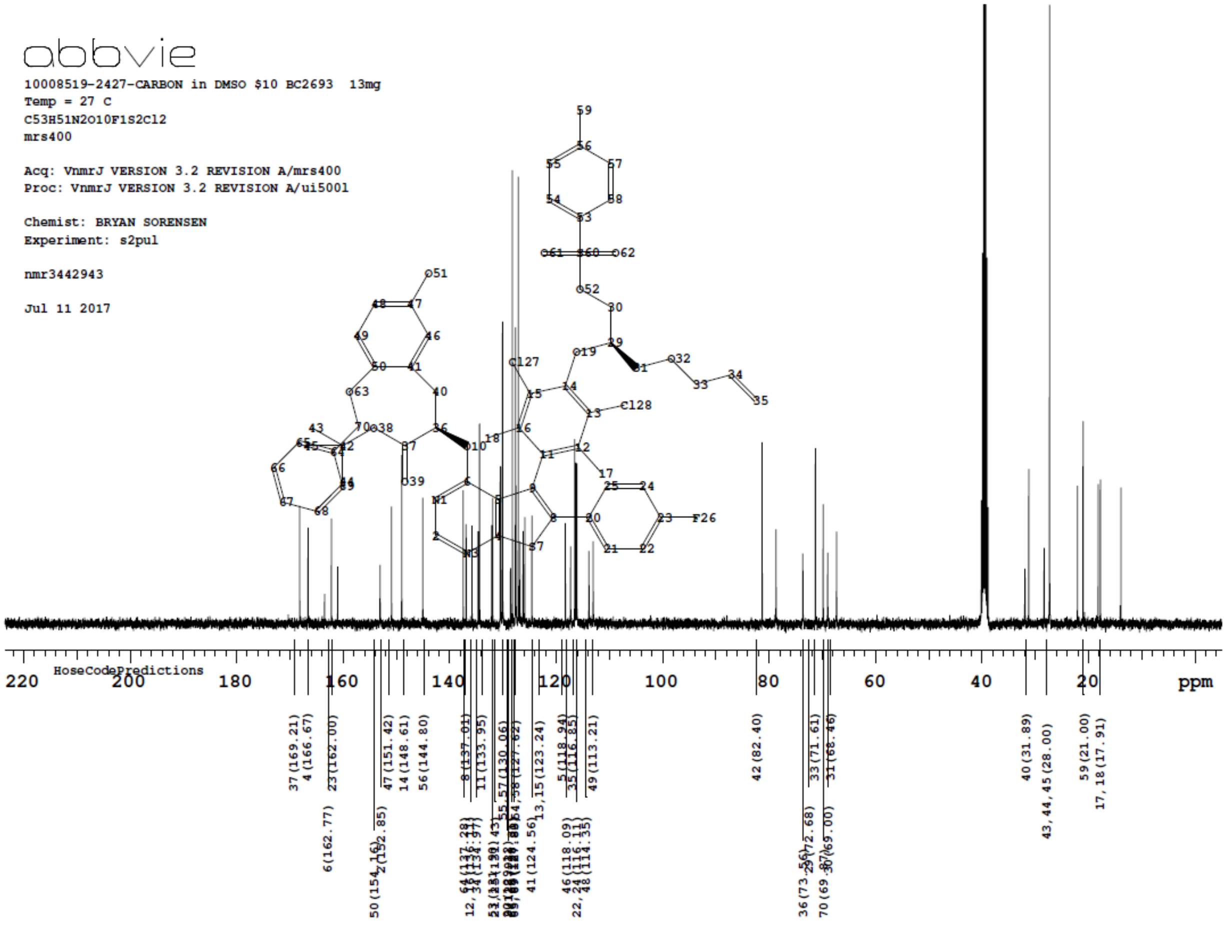
abbvie
10008519-2427-CARBON in DMSO $10 BC2693 13mg
Temp = 27 C
C53H51N2O10F1S2Cl2
mrs400
Acq: VnmrJ VERSION 3.2 REVISION A/mrs400
Proc: VnmrJ VERSION 3.2 REVISION A/ui5001
Chemist: BRYAN SORENSEN
Experiment: s2pul
nmr3442943
Jul 11 2017
HoseCodePredictions
ppm

**Sample Name**	AF	**Position**	P2-B6	**Instrument Name**	Instrument 1	**User Name**	
**Inj Vol**	0.1	**InjPosition**		**SampleType**	Sample	**IRM Calibration Status**	Success
**Data Filename**	AF.d	**ACQ Method**	IMSERC_ESI_Pos_Main_	**Comment**		**Acquired Time**	5/1/2018 5:44:57 PM

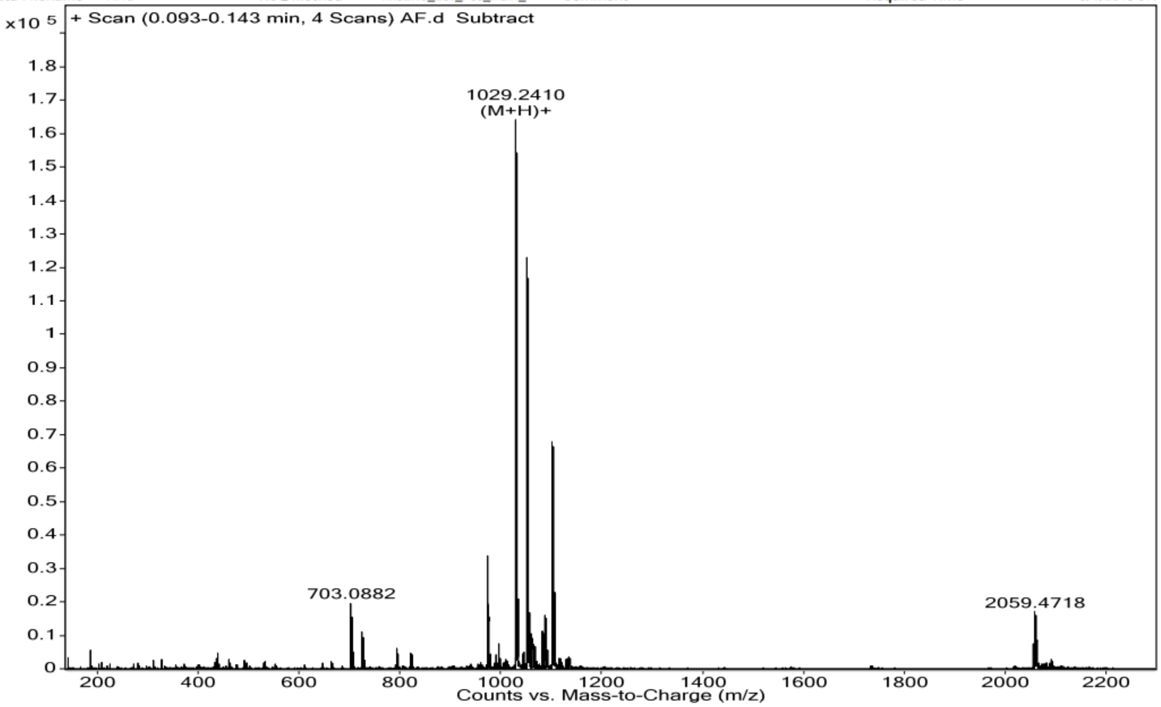

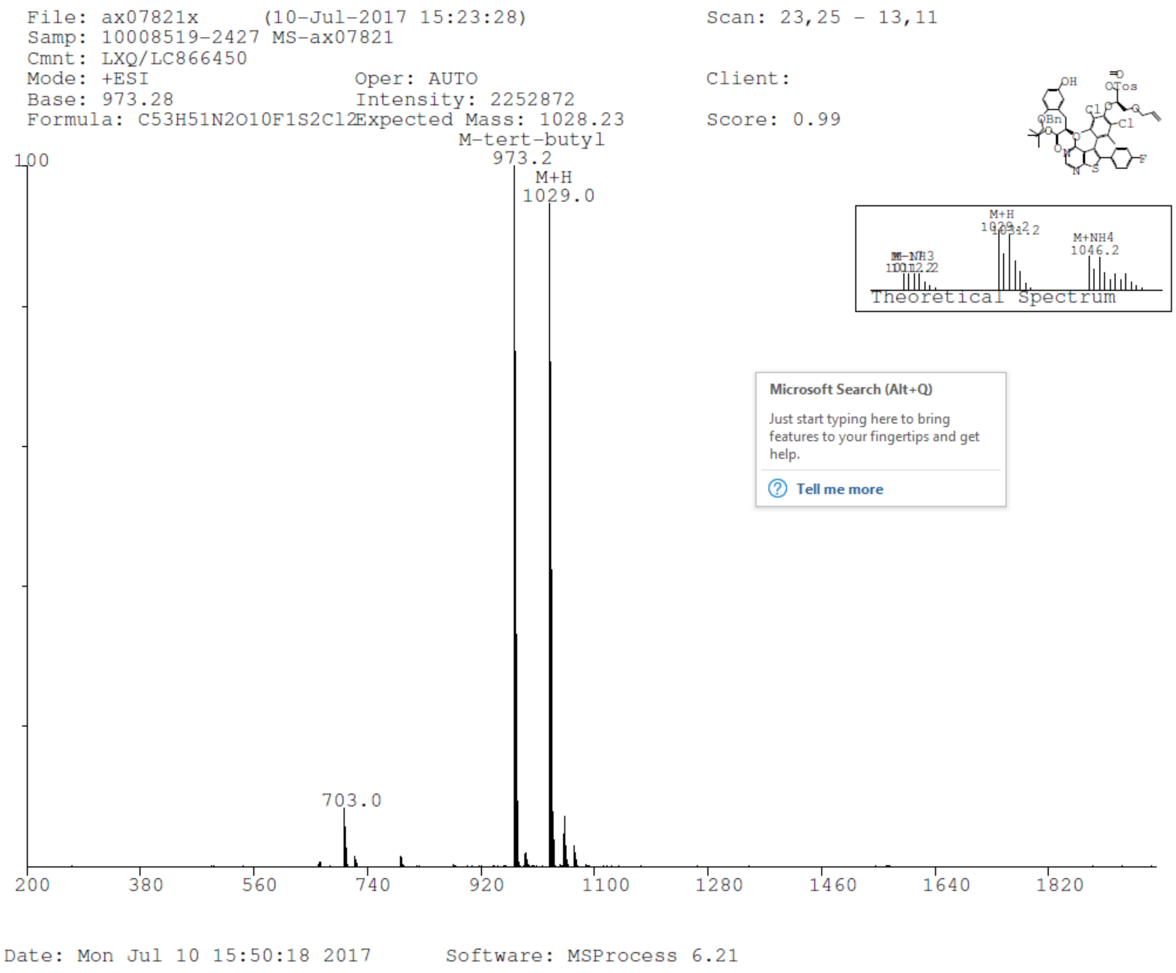

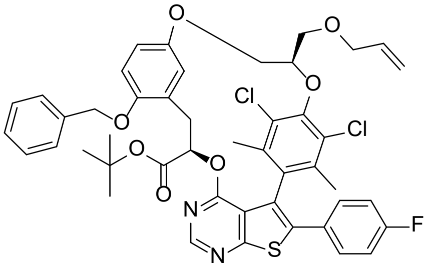

Tert-butyl (7R,16R)-10-(benzyloxy)-19,23-dichloro-1-(4-fluorophenyl)-20,22-dimethyl-16-{[(prop-2-en-1-yl)oxy]methyl}-7,8,15,16-tetrahydro-18,21-etheno-13,9-(metheno)-6,14,17-trioxa-2-thia-3,5-diazacyclononadeca[1,2,3-cd]indene-7-carboxylate (S-44).

abbvie

10034490-1807 in CDCL3 $31 BC233 2mg
Temp = 27 C
C46H43N2O7F1S1Cl2
v501

Acq: VnmrJ VERSION 3.2 REVISION A/v501
Proc: VnmrJ VERSION 3.2 REVISION A/i600

Chemist: MICHAEL CURTIN
Experiment: s2pul

nmr3500082

Feb 1 2018

Expected protons=43
Whole protons observed=43
Total integral =43.668

ID=86.70
P =89.00

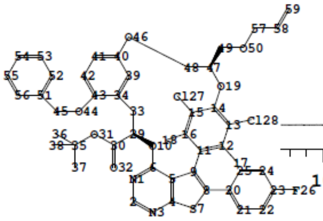

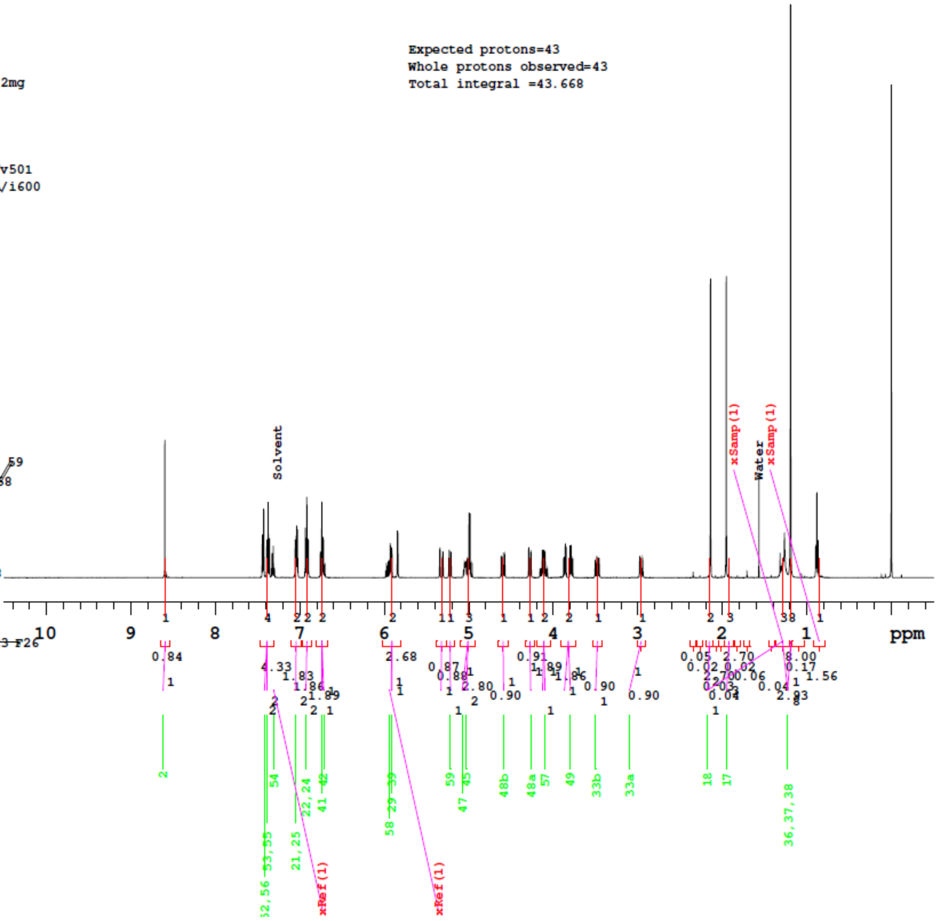

HoseCodePredictions

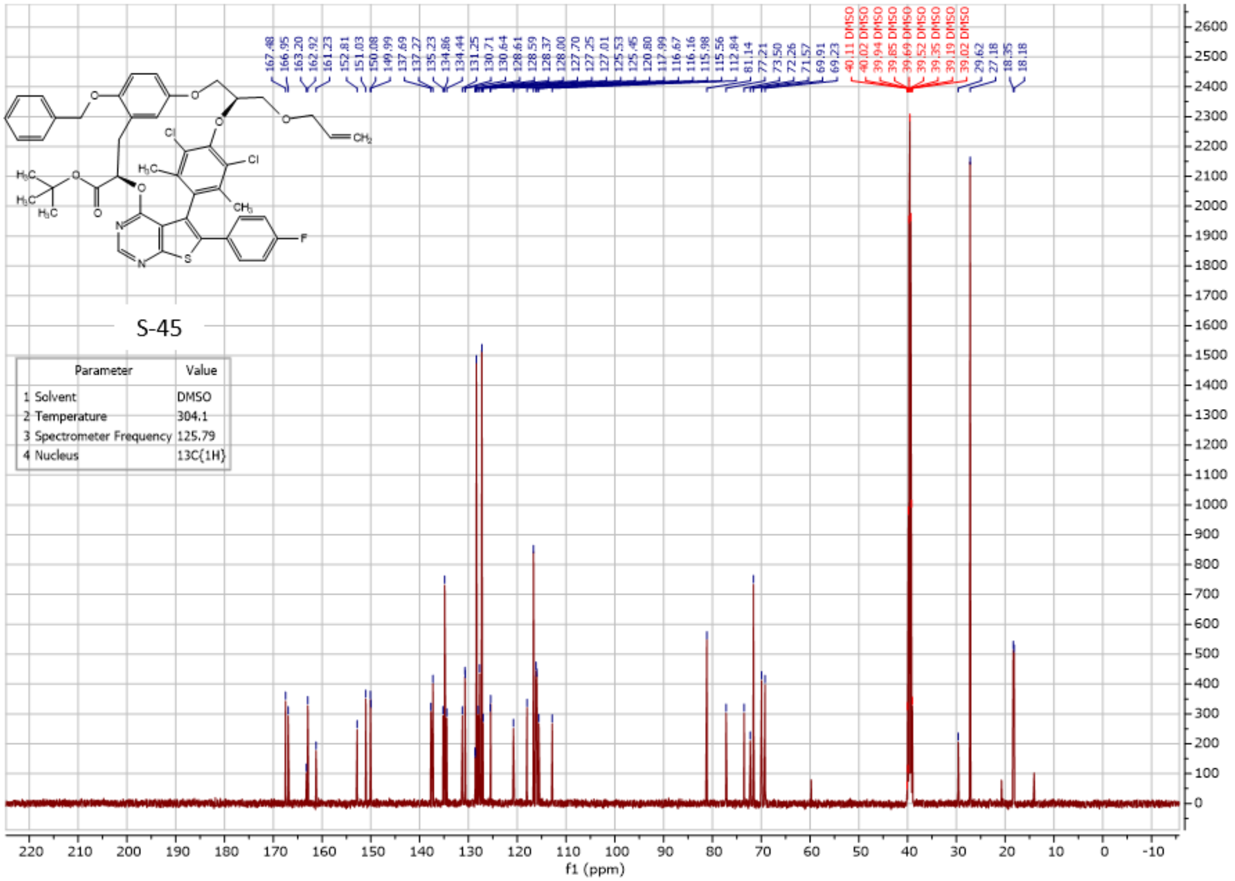

S-45

Parameter	Value
1 Solvent	DMSO
2 Temperature	304.1
3 Spectrometer Frequency	125.79
4 Nucleus	13C{1H}

**Sample Name**	AG	**Position**	P2-B7	**Instrument Name**	Instrument 1	**User Name**	
**Inj Vol**	0.1	**InjPosition**		**SampleType**	Sample	**IRM Calibration Status**	Success
**Data Filename**	AG.d	**ACQ Method**	IMSERC_ESI_Pos_Main_	**Comment**		**Acquired Time**	5/1/2018 5:48:00 PM

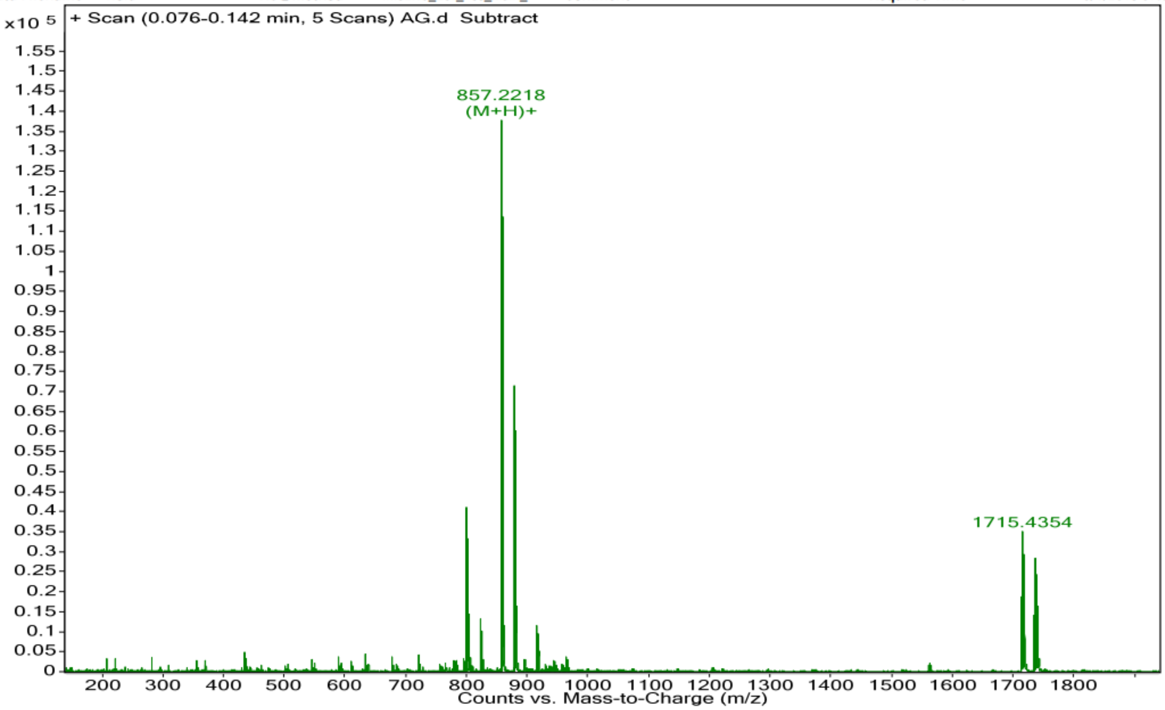

File: ax09929x (12-Jul-2017 06:12:56) Scan: 25,27 - 15,13
Samp: 10008519-2428 MS-ax09929
Cmnt: LXQ/LC866450
Mode: +ESI Oper: AUTO Client:
Base: 857.15 Intensity: 1613639
Formula: C46H43N2O7F1S1Cl2 Expected Mass: 856.22 Score: 0.70

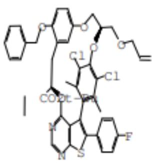

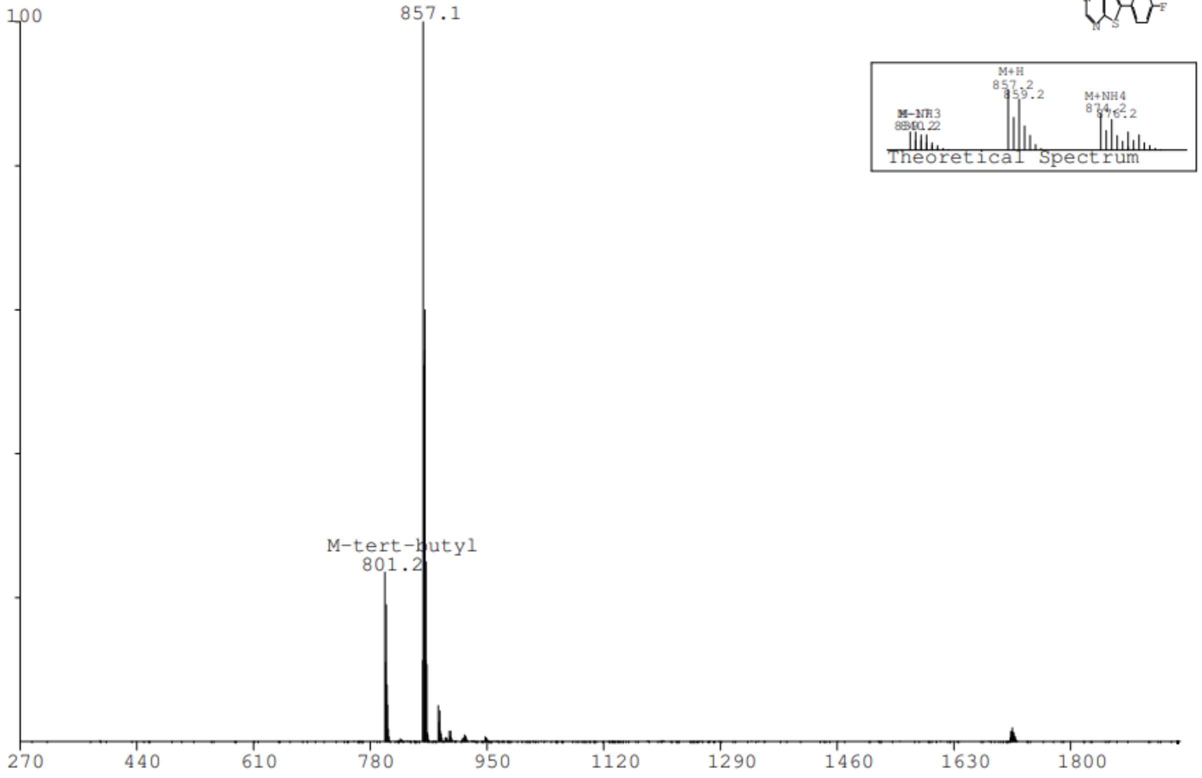

Date: Wed Jul 12 06:30:18 2017 Software: MSProcess 6.21

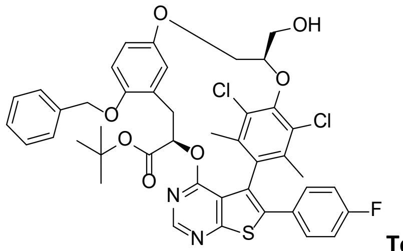

Tert-butyl (7R,16R)-10-(benzyloxy)-19,23-dichloro-1-(4-fluorophenyl)-16-(hydroxymethyl)-20,22-dimethyl-7,8,15,16-tetrahydro-18,21-etheno-13,9-(metheno)-6,14,17-trioxa-2-thia-3,5-diazacyclononadeca[1,2,3-cd]indene-7-carboxylate (S-45).

abbvie

10034490-1808 in CDCL3 $31 BC234 2mg
Temp = 27 C
C43H39N2O7F1S1Cl2
v501

Acq: VnmrJ VERSION 3.2 REVISION A/v501
Proc: VnmrJ VERSION 3.2 REVISION A/i600

Chemist: MICHAEL CURTIN
Experiment: s2pul

nmr3500079

Feb 1 2018

Expected protons=39
Whole protons observed=37
Total integral =38.532

ID=75.70
P =81.00

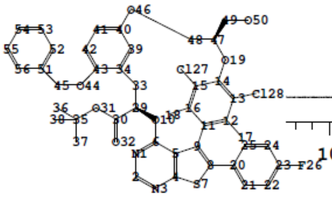

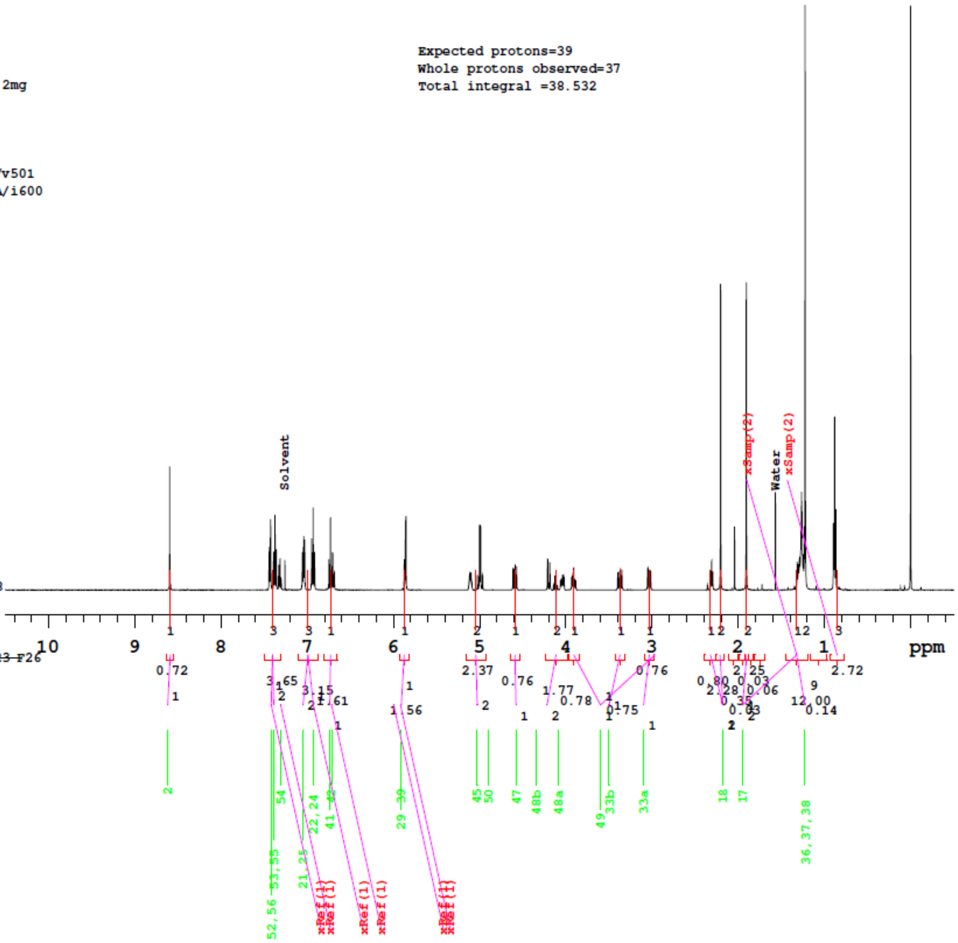

HoseCodePredictions

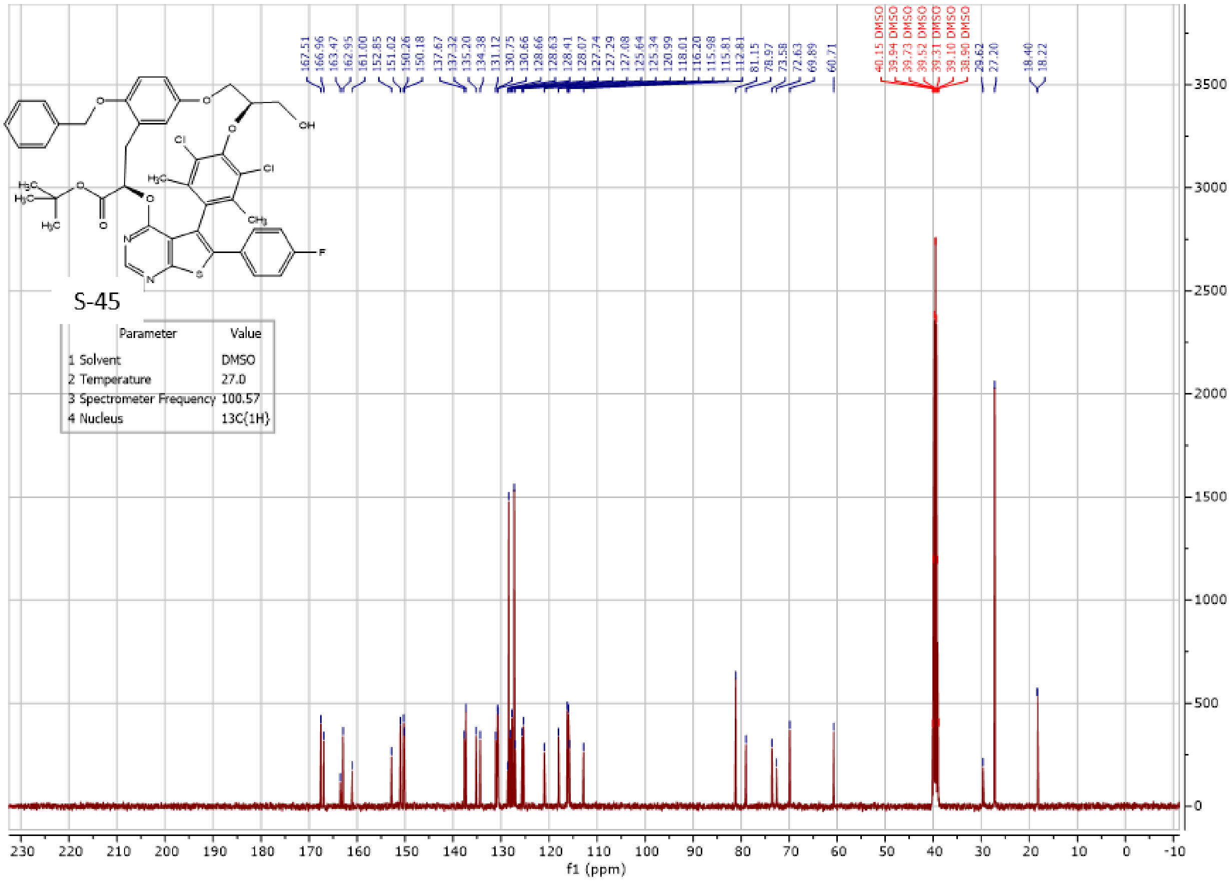

S-45

	Parameter	Value
1	Solvent	DMSO
2	Temperature	27.0
3	Spectrometer Frequency	100.57
4	Nucleus	13C{1H}

**Sample Name**	AH	**Position**	P2-B8	**Instrument Name**	Instrument 1	**User Name**	
**Inj Vol**	0.1	**InjPosition**		**SampleType**	Sample	**IRM Calibration Status**	Success
**Data Filename**	AH.d	**ACQ Method**	IMSERC_ESI_Pos_Main_	**Comment**		**Acquired Time**	5/1/2018 5:51:00 PM

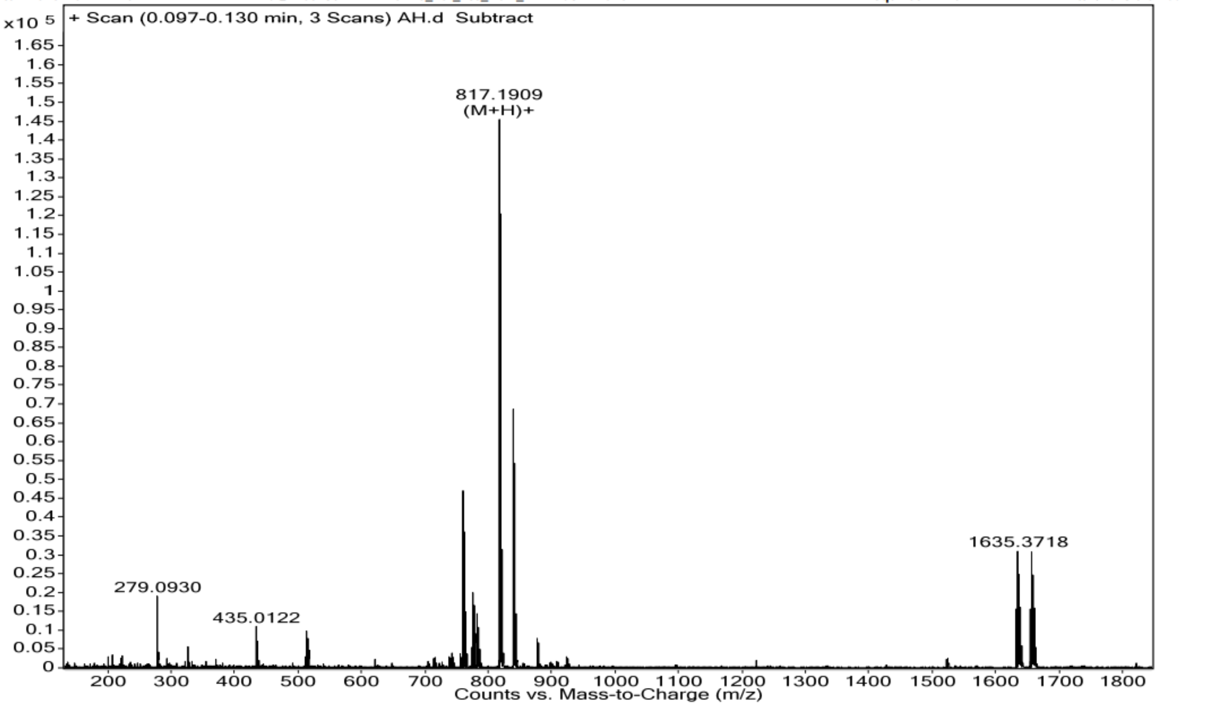

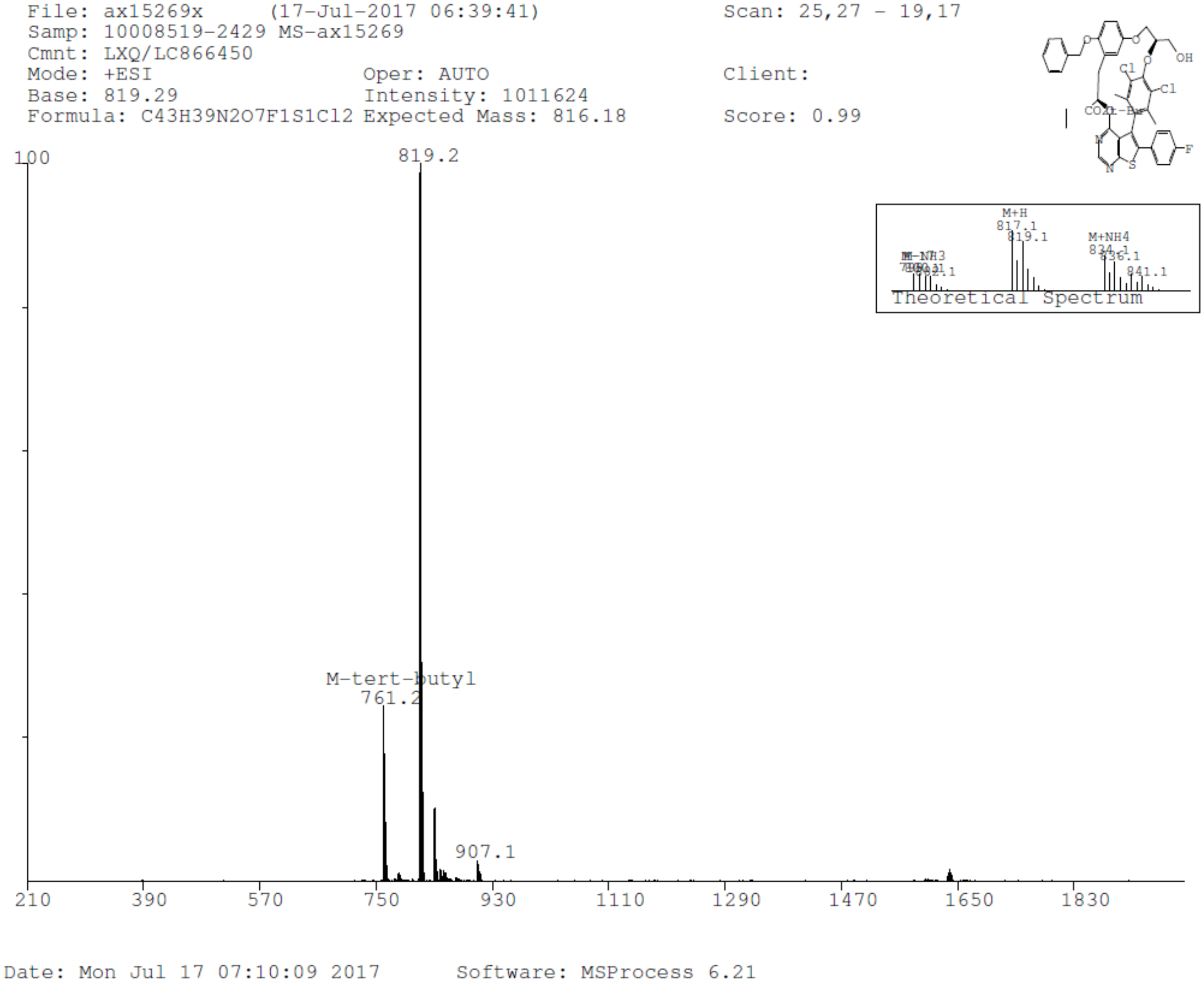

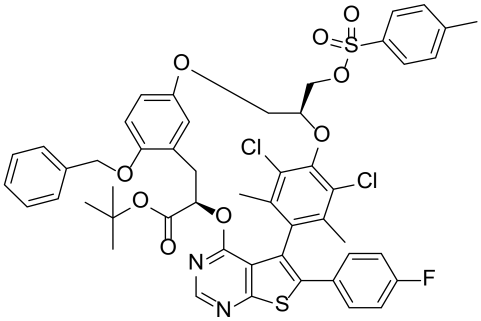

Tert-butyl (7R,16S)-10-(benzyloxy)-19,23-dichloro-1-(4-fluorophenyl)-20,22-dimethyl-16-{[(4-methylbenzene-1-sulfonyl)oxy]methyl}-7,8,15,16-tetrahydro-18,21-etheno-13,9-(metheno)-6,14,17-trioxa-2-thia-3,5-diazacyclononadeca[1,2,3-cd]indene-7-carboxylate (S-46).

abbvie

10034490-1809 in CDCL3 $31 BC235 2mg
Temp = 27 C
C50H45N2O9F1S2Cl2
v501

Acq: VnmrJ VERSION 3.2 REVISION A/v501
Proc: VnmrJ VERSION 3.2 REVISION A/i600

Chemist: MICHAEL CURTIN
Experiment: s2pul

nmr3500092

Feb 1 2018

Expected protons=45
Whole protons observed=46
Total integral =46.131

ID=84.50
P =89.00

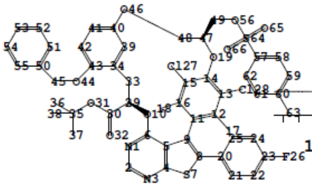

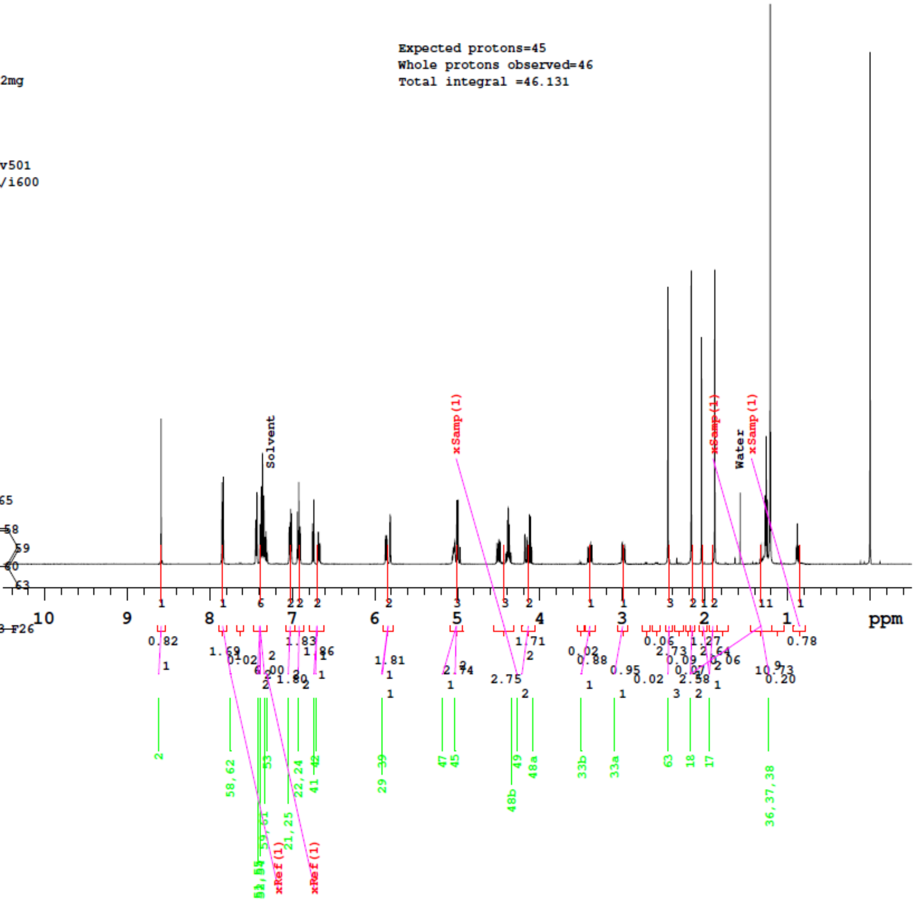

HoseCodePredictions

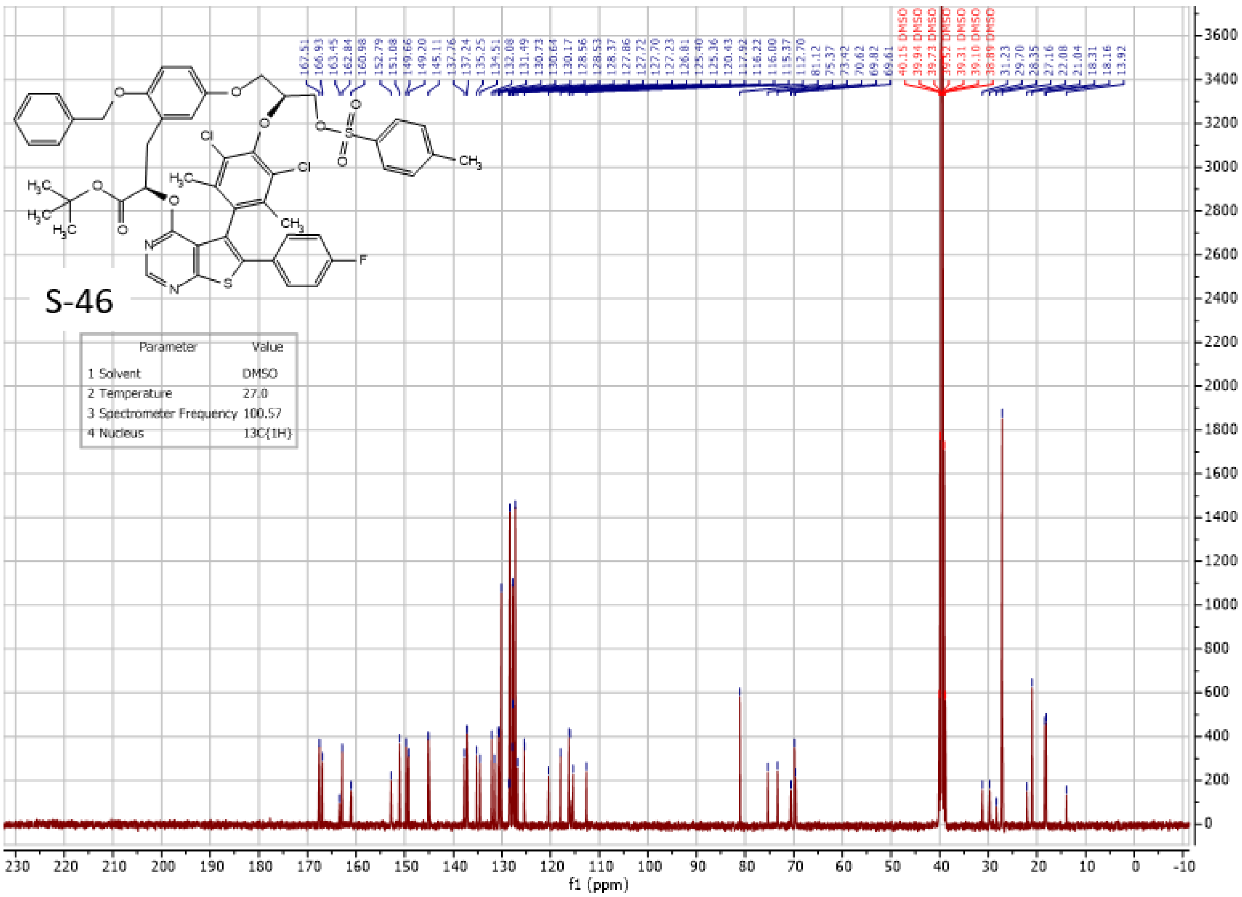

**Sample Name**	AI	**Position**	P2-B9	**Instrument Name**	Instrument 1	**User Name**	
**Inj Vol**	1	**InjPosition**		**SampleType**	Sample	**IRM Calibration Status**	Success
**Data Filename**	AI.d	**ACQ Method**	IMSERC_ESI_Pos_Main_	**Comment**		**Acquired Time**	5/4/2018 2:23:55 PM

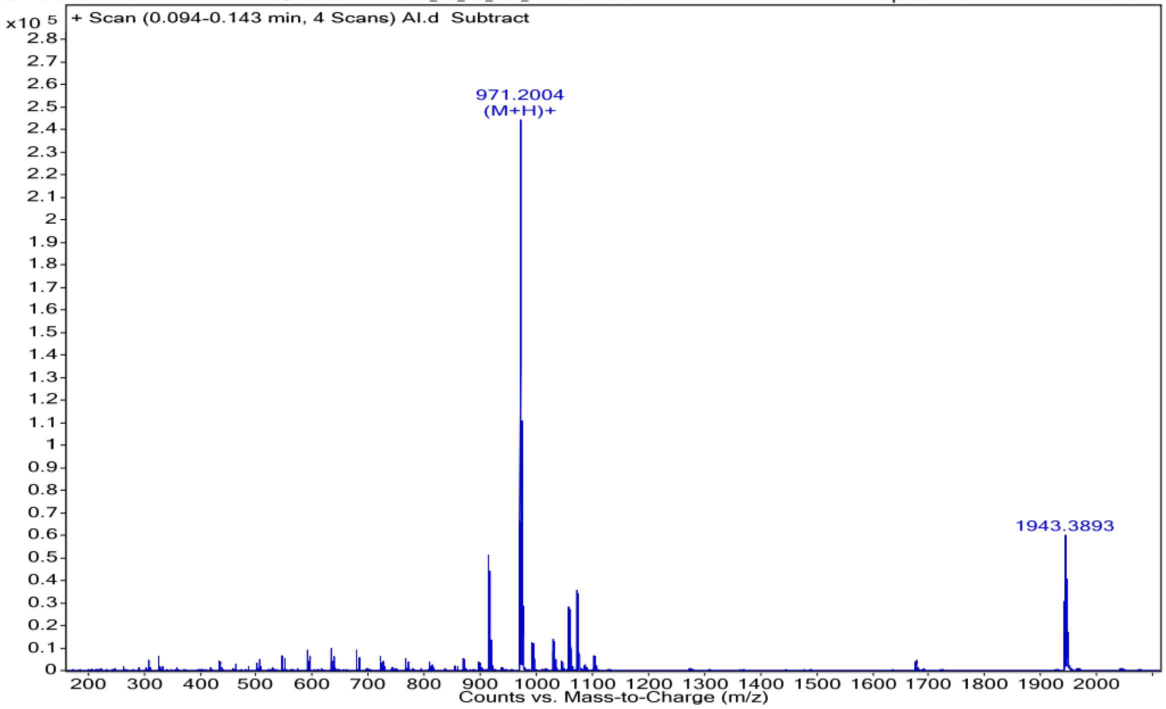

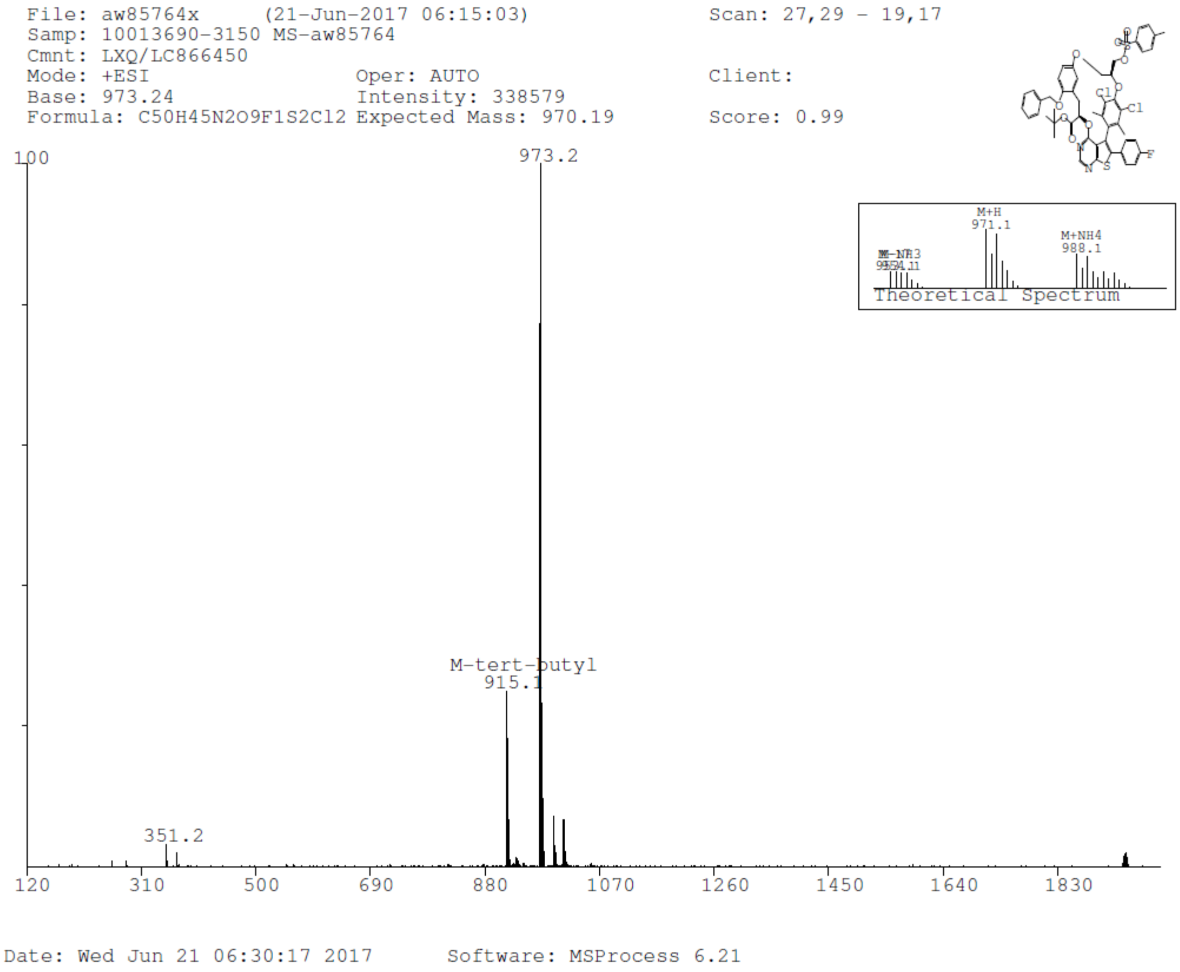

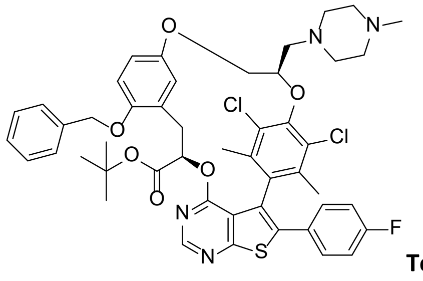

Tert-butyl (7R,16R)-10-(benzyloxy)-19,23-dichloro-1-(4-fluorophenyl)-20,22-dimethyl-16-[(4-methylpiperazin-1-yl)methyl]-7,8,15,16-tetrahydro-18,21-etheno-13,9-(metheno)-6,14,17-trioxa-2-thia-3,5-diazacyclononadeca[1,2,3-cd]indene-7-carboxylate (S-47).

abbvie

10034490-1811 in DMSO $06 BC836 2mg
Temp = 27 C
C48H49N4O6F1S1Cl2
jr400

Acq: VnmrJ VERSION 3.2 REVISION A/jr400
Proc: VnmrJ VERSION 3.2 REVISION A/coffee

Chemist: MICHAEL CURTIN
Experiment: s2pul

nmr3501806

Feb 7 2018

ID=89.90
P =95.00

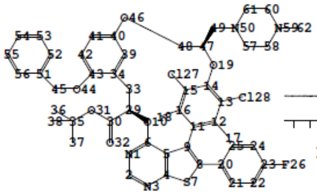

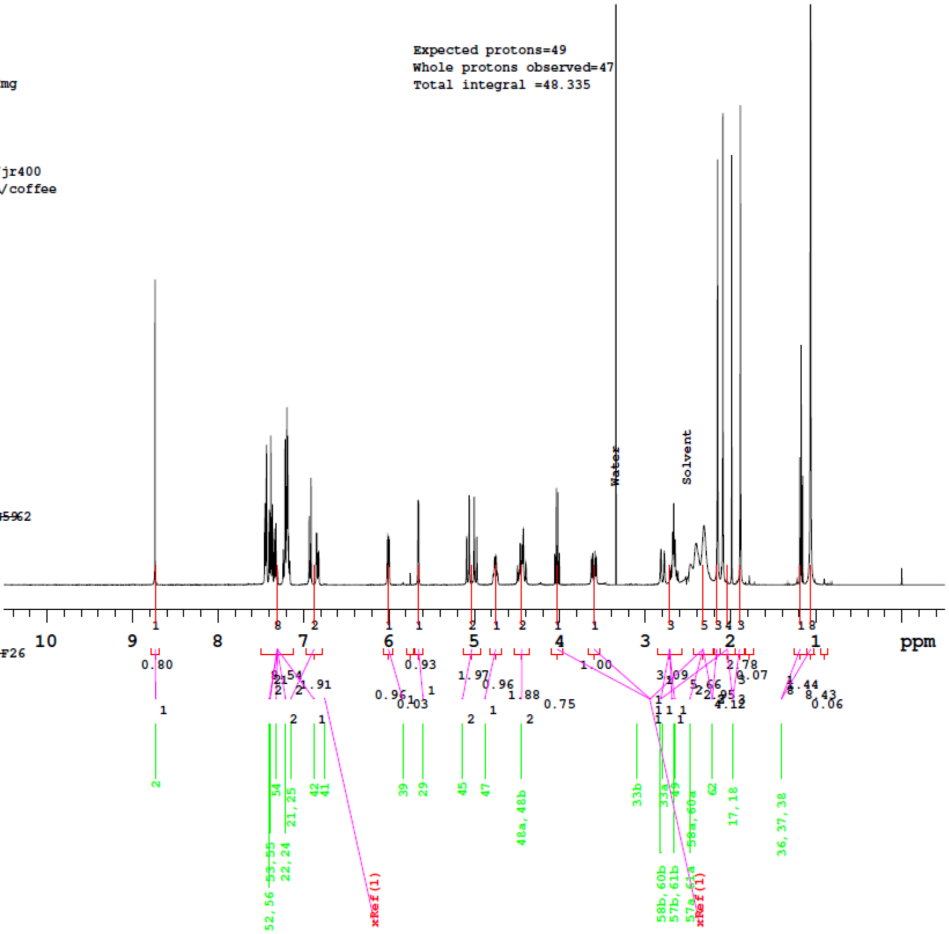

HoseCodePredictions

abbvie

10008519-2431-CARBON in DMSO BC#2700 $17
av500

Acq:TOPSPIN Version 3.5-pl5/av500

Chemist: BRYAN SORENSEN
Experiment: zg0pg

nmr3444910

Jul 17 17

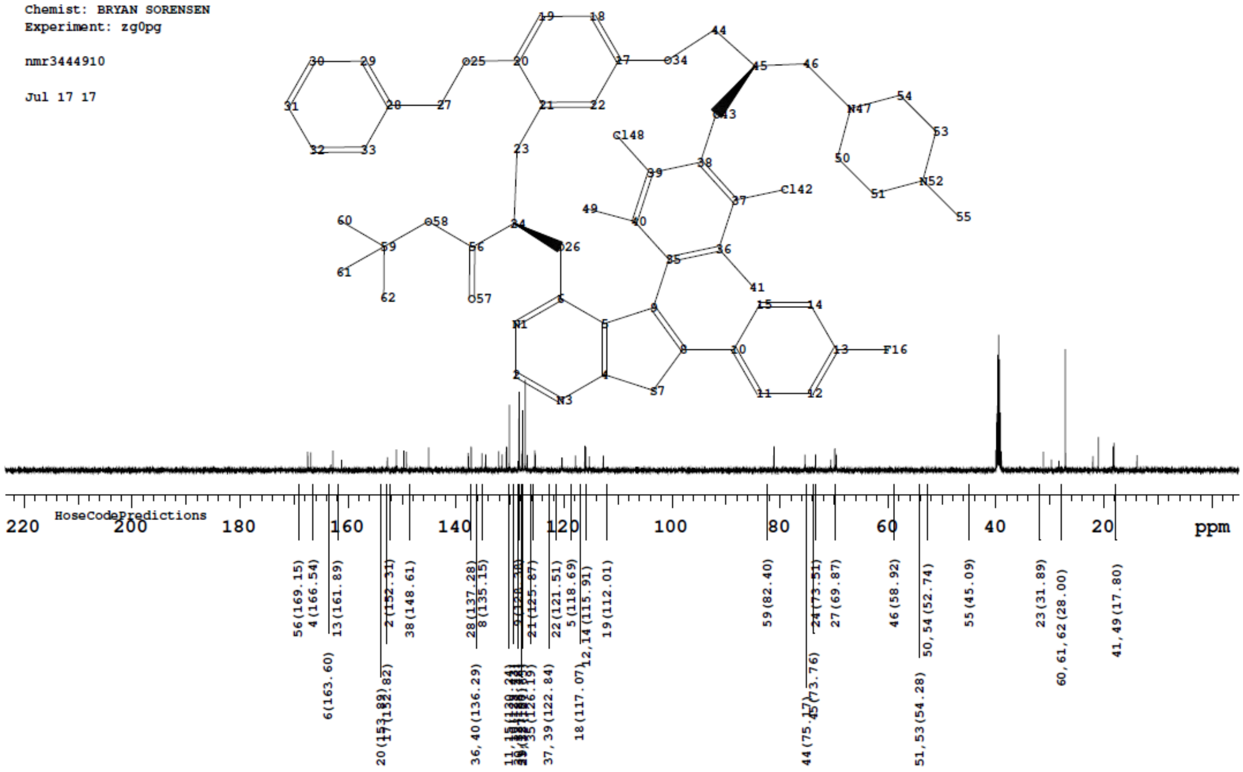

Sample Name	AJ	Position	P2-C1	Instrument Name	Instrument 1	User Name	
Inj Vol	0.1	InjPosition		SampleType	Sample	IRM Calibration Status	Success
Data Filename	AJ.d	ACQ Method	IMSERC_ESI_Pos_Main_	Comment		Acquired Time	5/1/2018 5:57:52

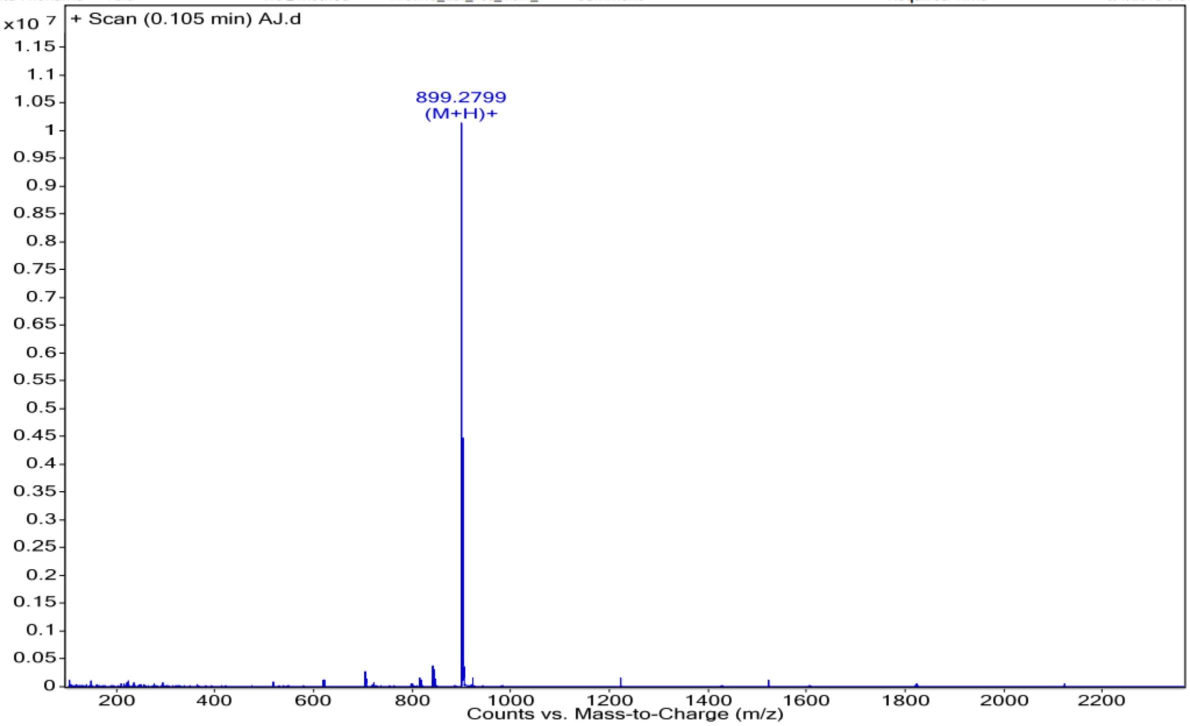

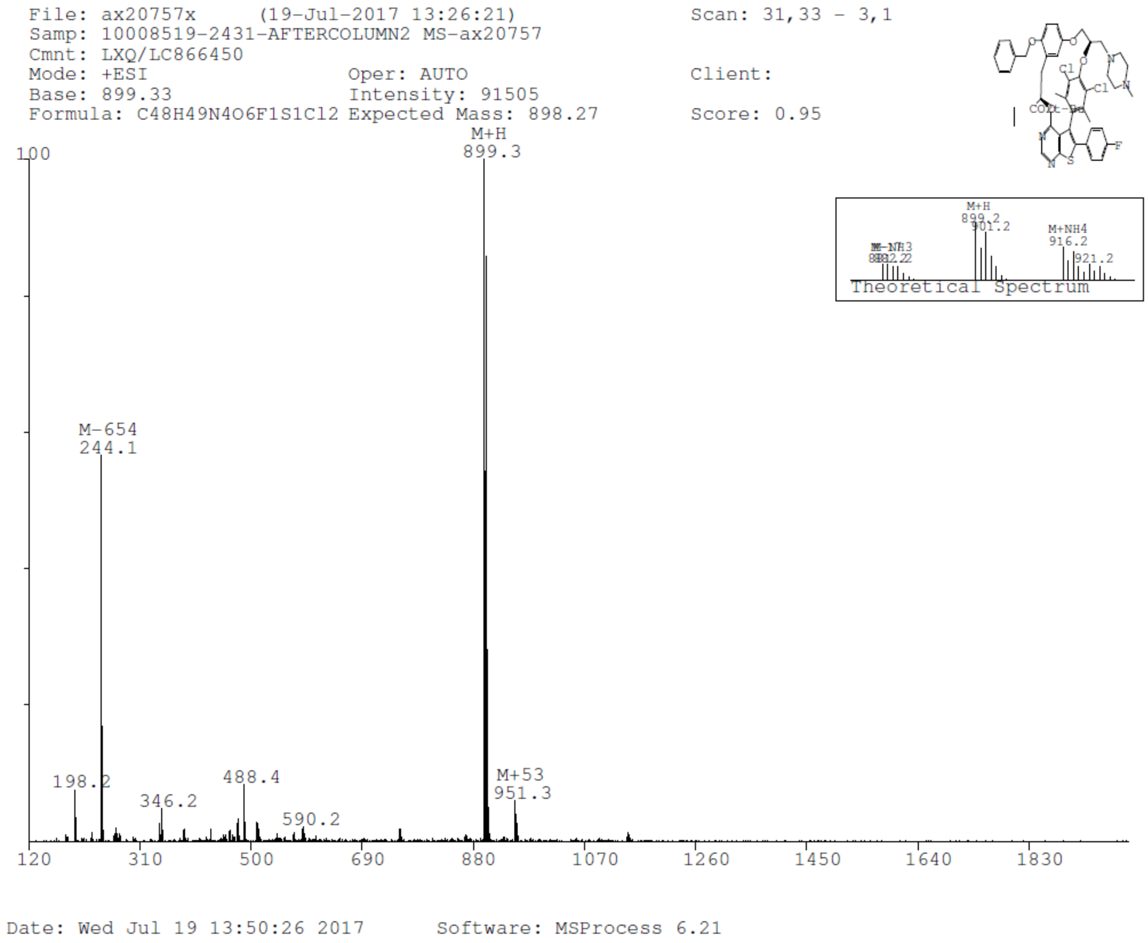

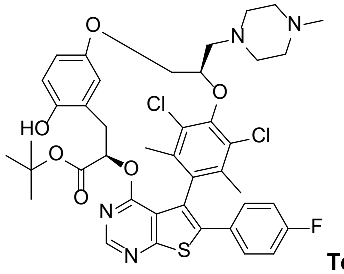

Tert-butyl (7R,16R)-19,23-dichloro-1-(4-fluorophenyl)-10-hydroxy-20,22-dimethyl-16-[(4-methylpiperazin-1-yl)methyl]-7,8,15,16-tetrahydro-18,21-etheno-13,9-(metheno)-6,14,17-trioxa-2-thia-3,5-diazacyclononadeca[1,2,3-cd]indene-7-carboxylate (S-48

abbvie

10008519-2433 in CDCL3 $20 BC692 6mg
Temp = 27 C
C41H43N4O6F1S1Cl2
jr400

Acq: VnmrJ VERSION 3.2 REVISION A/jr400
Proc: VnmrJ VERSION 3.2 REVISION A/m3001

Chemist: BRYAN SORENSEN
Experiment: s2pul

nmr3446059

Jul 21 2017

Expected protons=43
Whole protons observed=40
Total integral =42.825

ID=84.10
P =93.00

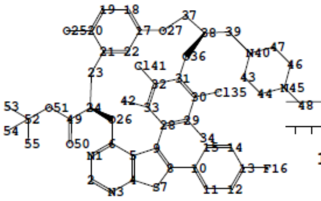

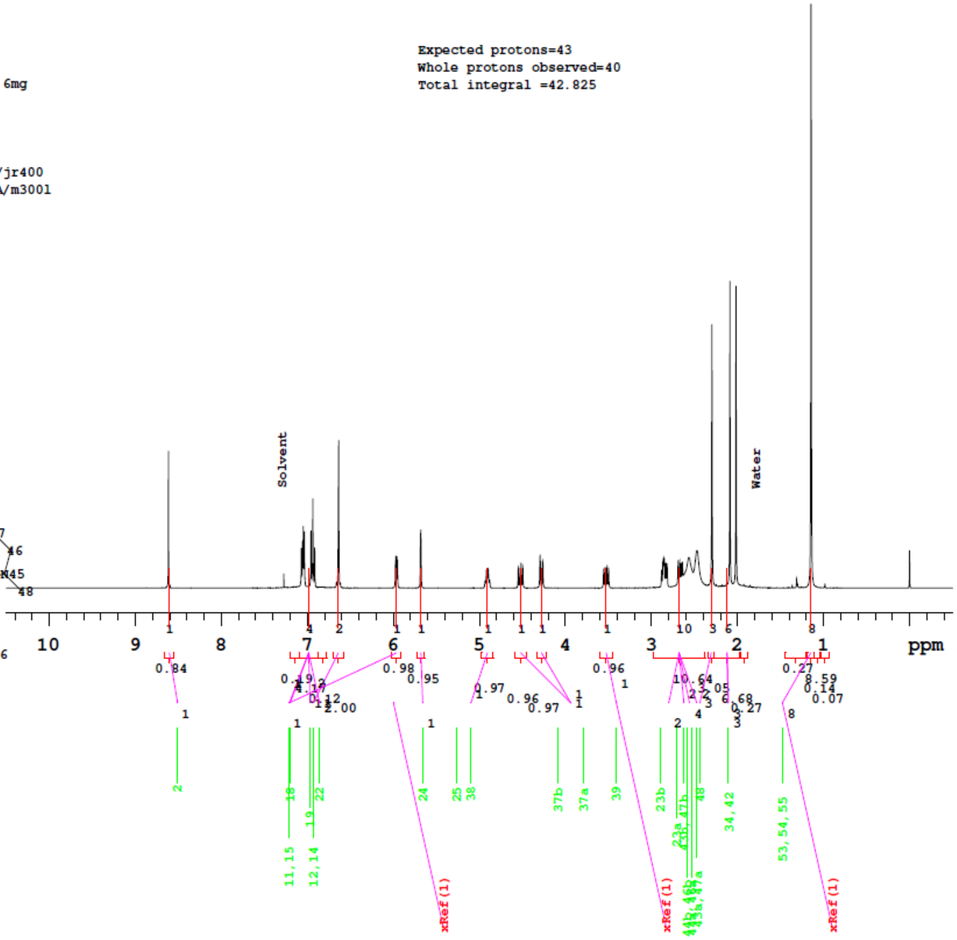

HoseCodePredictions

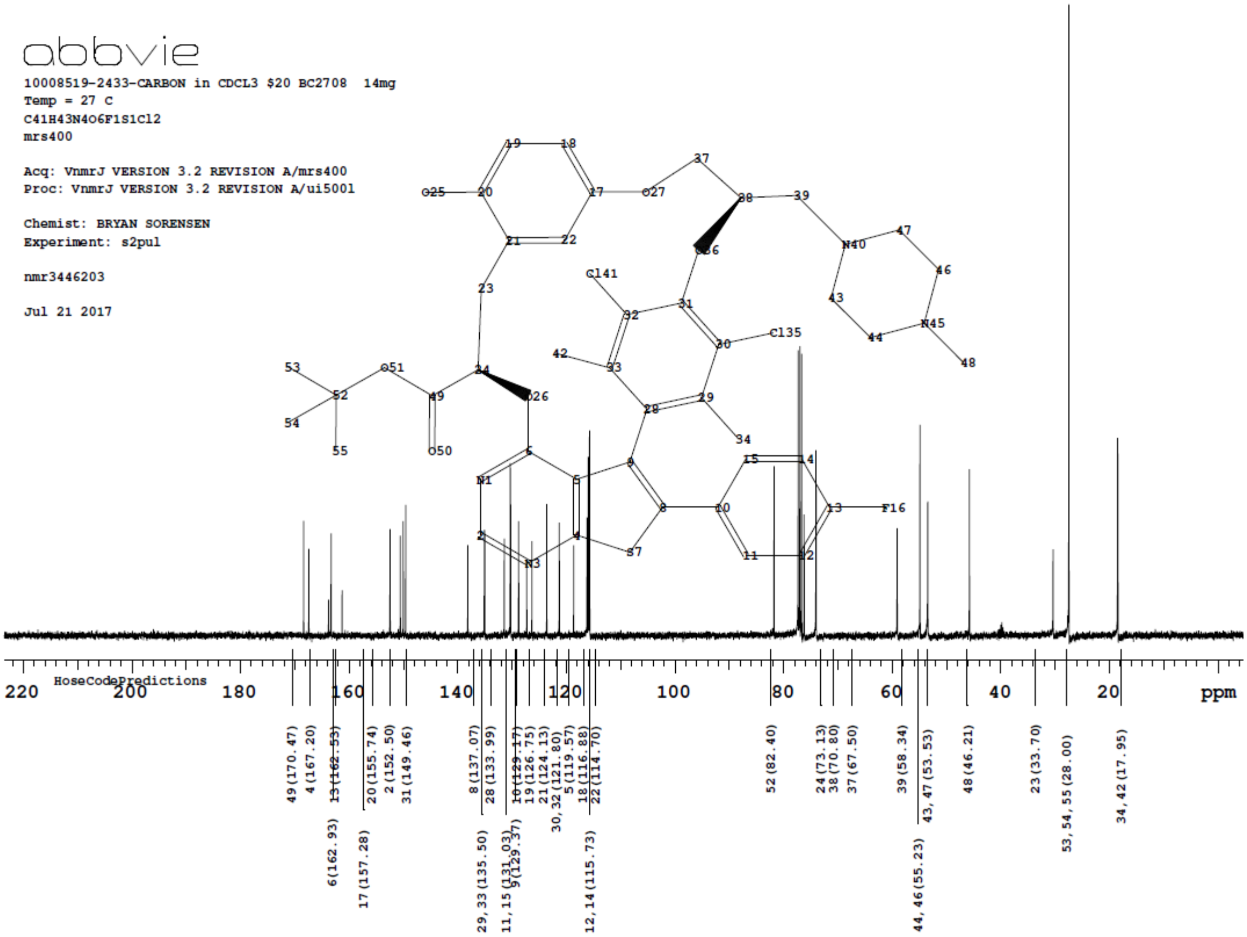
abbvie
10008519-2433-CARBON in CDCL3 S20 BC2708 14mg
Temp = 27 C
C41H43N4O6F1S1Cl2
mrs400
Acq: VnmrJ VERSION 3.2 REVISION A/mrs400
Proc: VnmrJ VERSION 3.2 REVISION A/ui5001
Chemist: BRYAN SORENSEN
Experiment: s2pul
nmr3446203
Jul 21 2017
HoseCodePredictions
ppm
49(170.47)
4(167.20)
6(162.93)
13(162.53)
17(157.28)
20(155.74)
2(152.50)
31(149.46)
8(137.07)
29,33(135.50)
28(133.99)
11,15(131.03)
10(129.17)
9(129.37)
19(126.75)
21(124.13)
30,32(121.80)
5(119.57)
18(116.88)
12,14(115.73)
22(114.70)
52(82.40)
24(73.13)
38(70.80)
37(67.50)
39(58.34)
44,46(55.23)
43,47(53.53)
48(46.21)
23(33.70)
53,54,55(28.00)
34,42(17.95)

**Sample Name**	AK	**Position**	P2-C2	**Instrument Name**	Instrument 1	**User Name**	
**Inj Vol**	0.1	**InjPosition**		**SampleType**	Sample	**IRM Calibration Status**	Success
**Data Filename**	AK.d	**ACQ Method**	IMSERC_ESI_Pos_Main_	**Comment**		**Acquired Time**	5/1/2018 6:00:56 PM

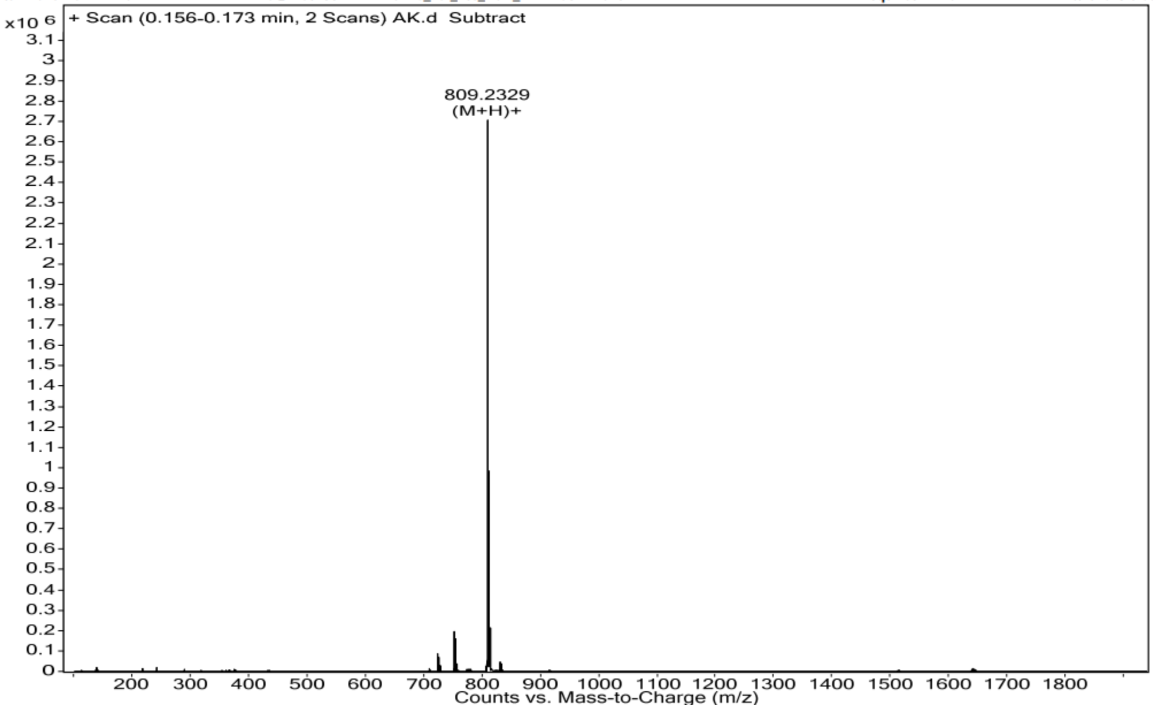

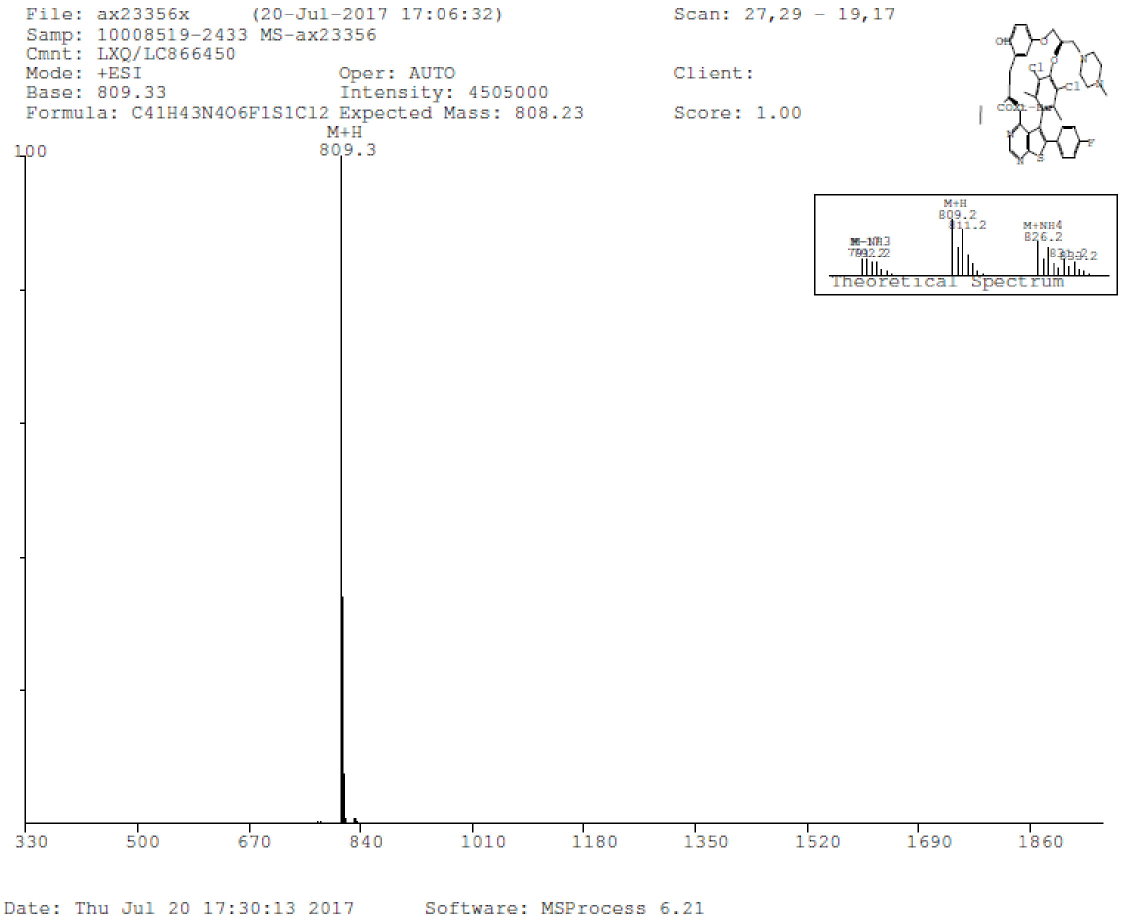

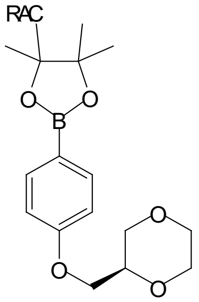

(R)-2-(4-((1,4-dioxan-2-yl)methoxy)phenyl)-4,4,5,5-tetramethyl-1,3,2-dioxaborolane (S-49).

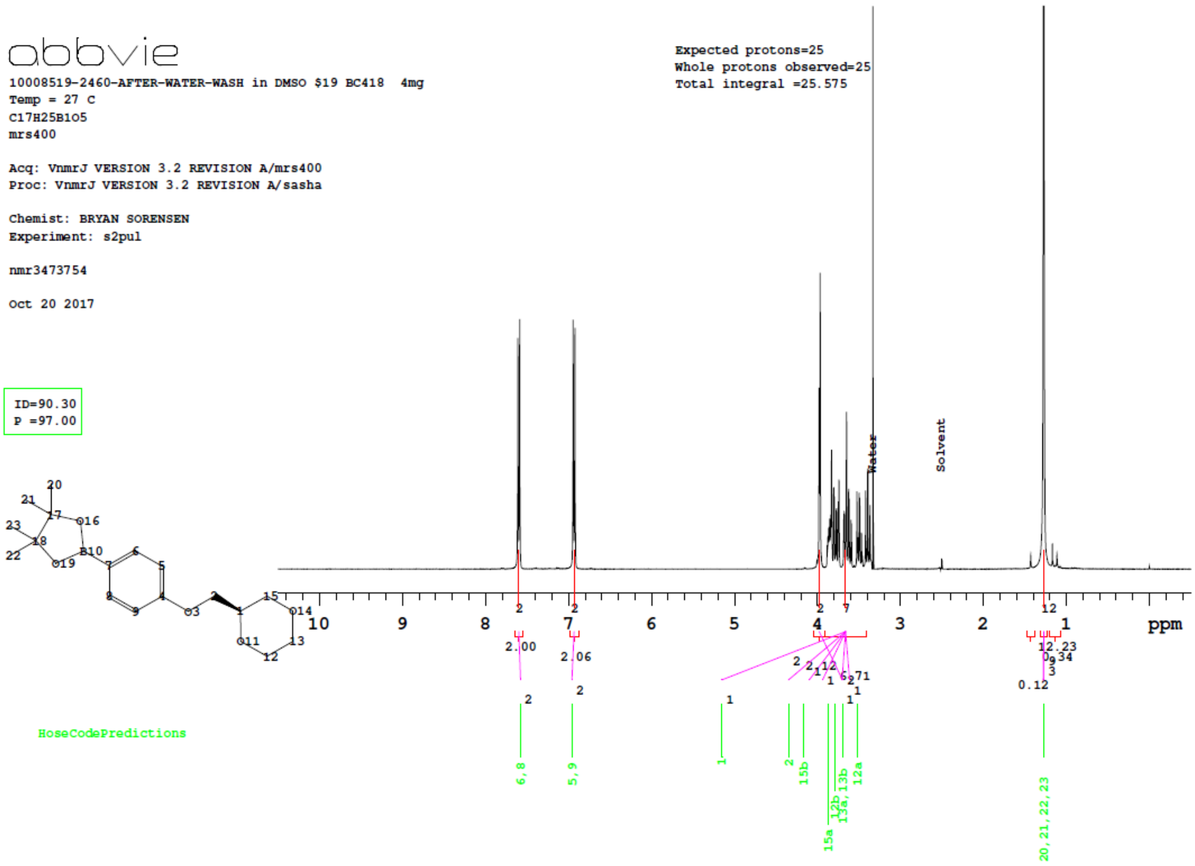
abbvie
10008519-2460-AFTER-WATER-WASH in DMSO $19 BC418 4mg
Temp = 27 C
C17H25B1O5
mrs400
Acq: VnmrJ VERSION 3.2 REVISION A/mrs400
Proc: VnmrJ VERSION 3.2 REVISION A/sasha
Chemist: BRYAN SORENSEN
Experiment: s2pul
nmr3473754
Oct 20 2017
ID=90.30
P =97.00
Expected protons=25
Whole protons observed=25
Total integral =25.575
Water
Solvent
HoseCodePredictions
ppm
2.00
2.06
0.12
6,8
5,9
15b
12a
15a
20,21,22,23

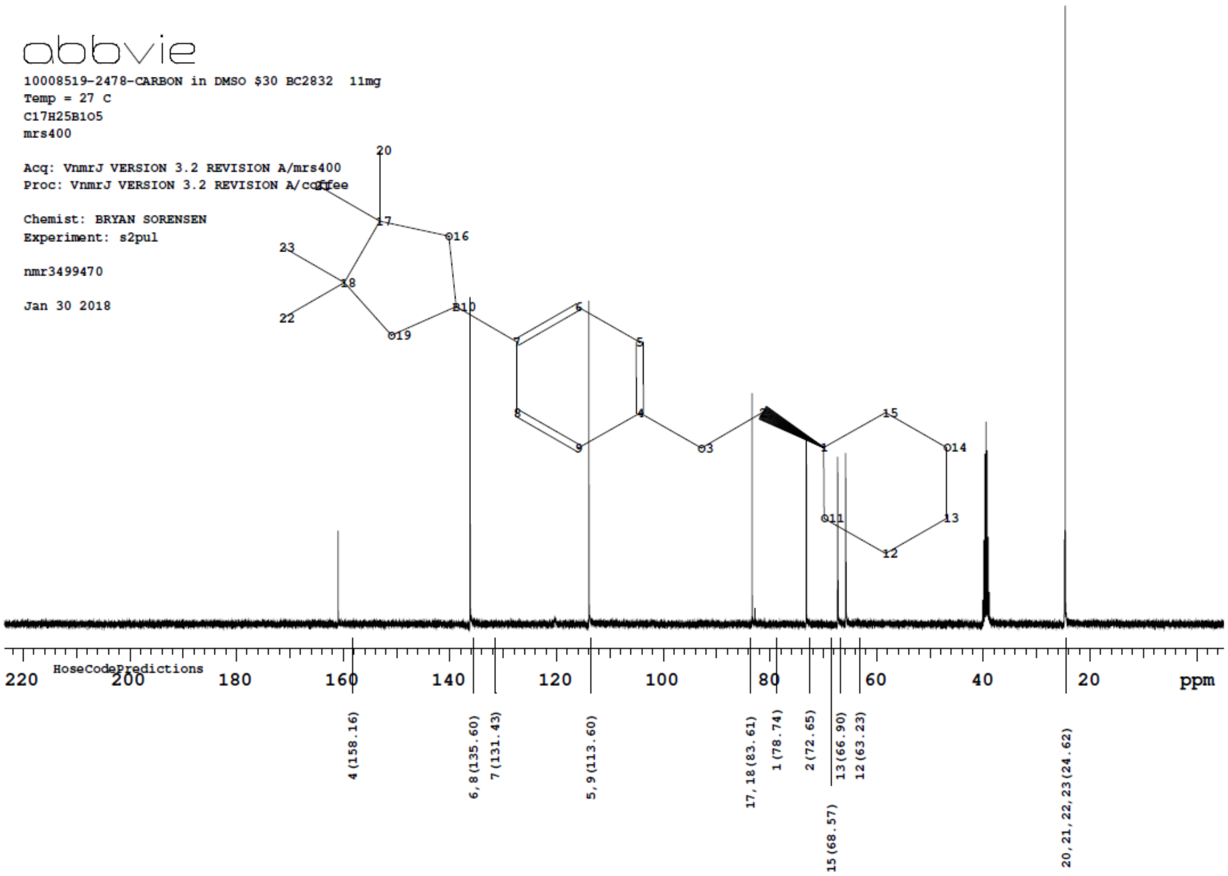

10008519-2478-CARBON in DMSO $30 BC2832 11mg
Temp = 27 C
C17H25BIO5
mrs400
Acq: VnmrJ VERSION 3.2 REVISION A/mrs400
Proc: VnmrJ VERSION 3.2 REVISION A/coffee
Chemist: BRYAN SORENSEN
Experiment: s2pul
nmr3499470
Jan 30 2018
HoseCodePredictions
4 (158.16)
6,8 (135.60)
7 (131.43)
5,9 (113.60)
17,18 (83.61)
1 (78.74)
2 (72.65)
15 (68.57)
13 (66.90)
12 (63.23)
20,21,22,23 (24.62)
ppm

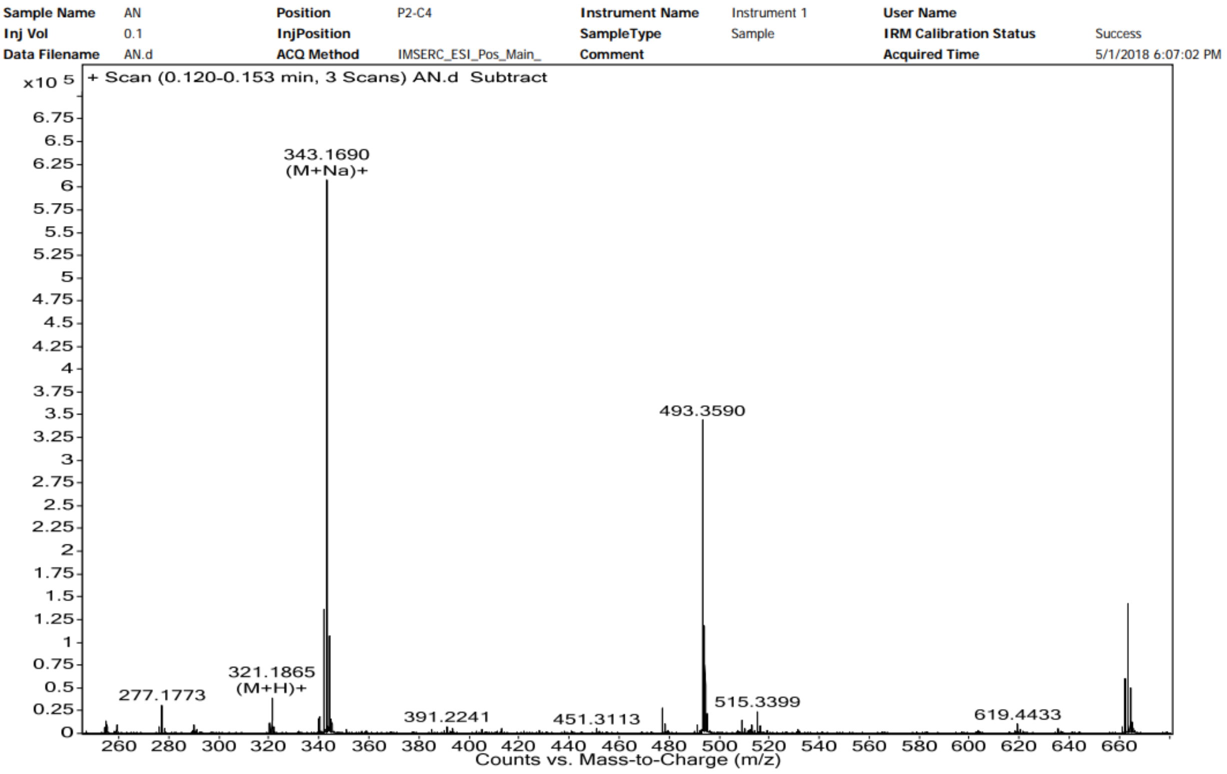

Sample Name AN
Inj Vol 0.1
Data Filename AN.d
Position P2-C4
InjPosition
ACQ Method IMSERC_ESI_Pos_Main_
Instrument Name Instrument 1
SampleType Sample
Comment
User Name
IRM Calibration Status Success
Acquired Time 5/1/2018 6:07:02 PM
+ Scan (0.120-0.153 min, 3 Scans) AN.d Subtract
343.1690
(M+Na)+
493.3590
321.1865
(M+H)+
277.1773
391.2241
451.3113
515.3399
619.4433
Counts vs. Mass-to-Charge (m/z)

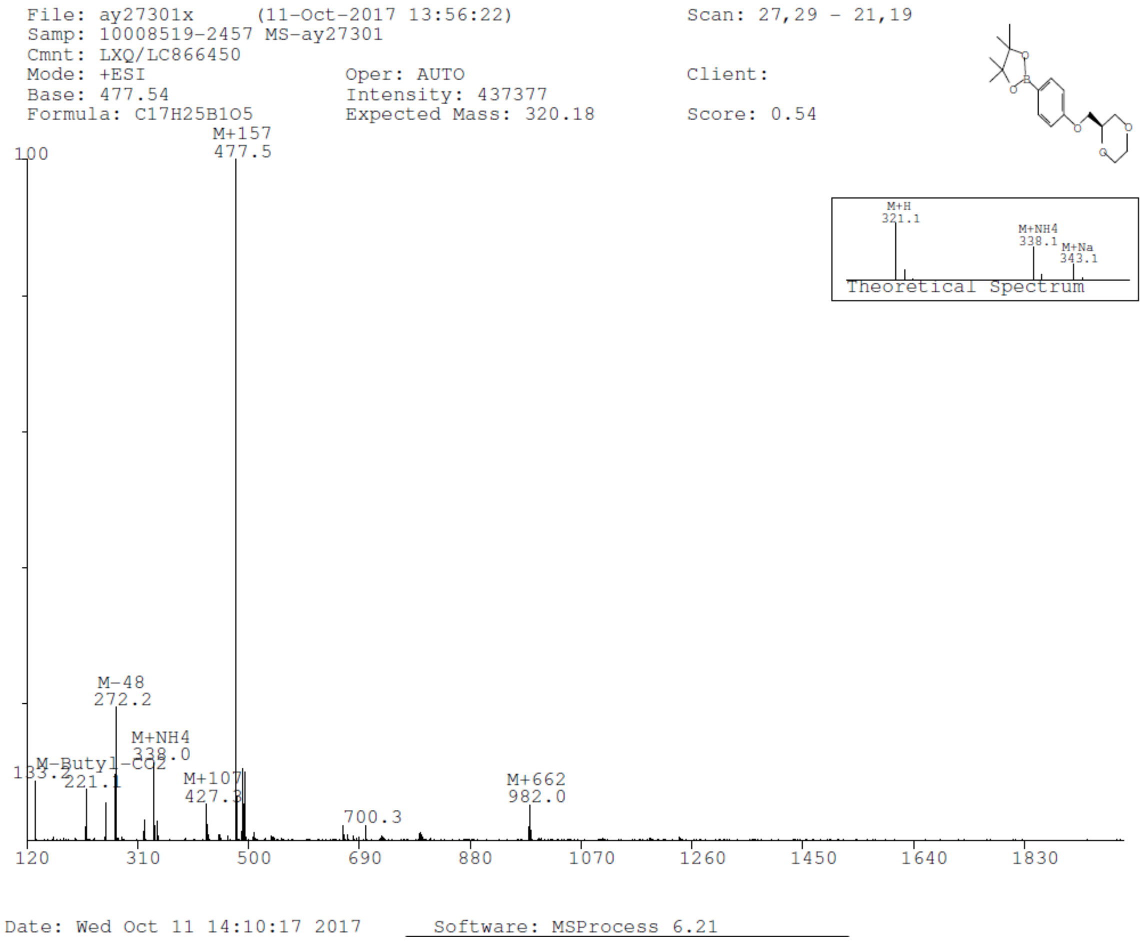

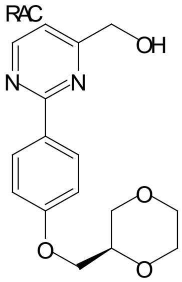

(R)-(2-(4-((1,4-dioxan-2-yl)methoxy)phenyl)pyrimidin-4-yl)methanol (S-50).

abbvie

10008519-2459 in DMSO $13 BC1788 3mg
Temp = 27 C
C16H18N2O4
mrs400

Acq: VnmrJ VERSION 3.2 REVISION A/mrs400
Proc: VnmrJ VERSION 3.2 REVISION A/m3001

Chemist: BRYAN SORENSEN
Experiment: s2pul

nmr3471935

Oct 13 2017

Expected protons=18
Whole protons observed=18
Total integral =18.377

ID=82.20
P =92.00

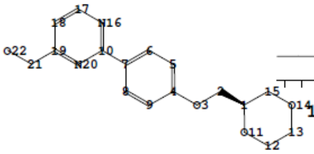

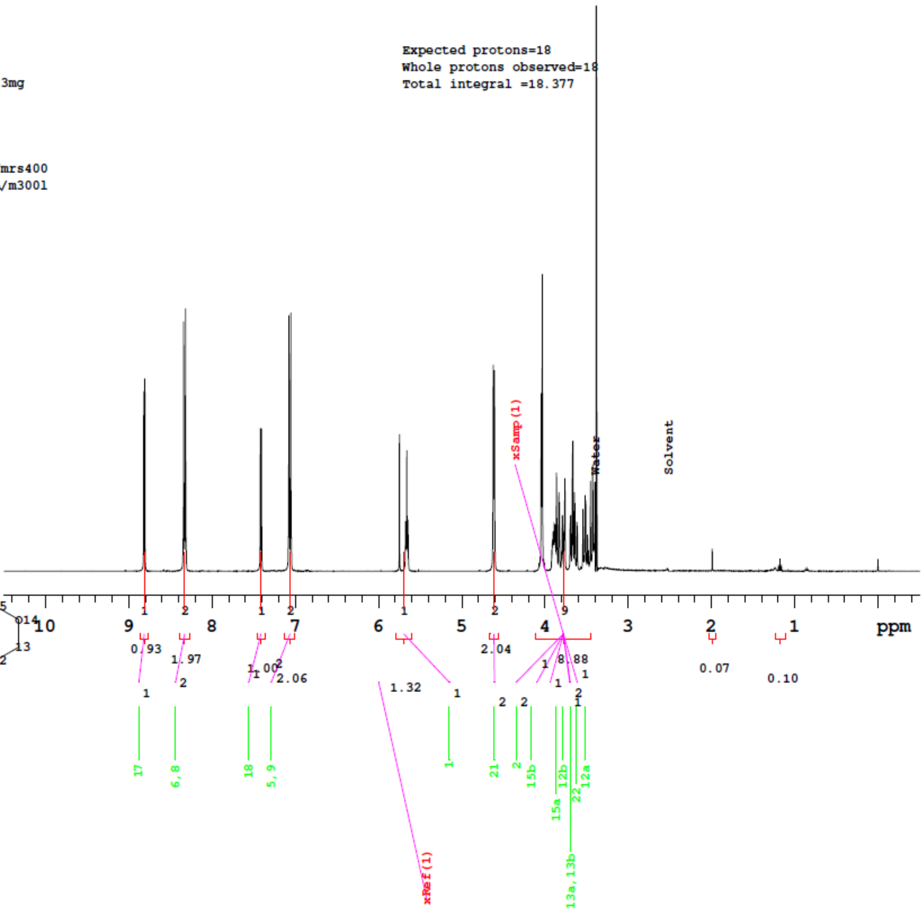

HoseCodePredictions

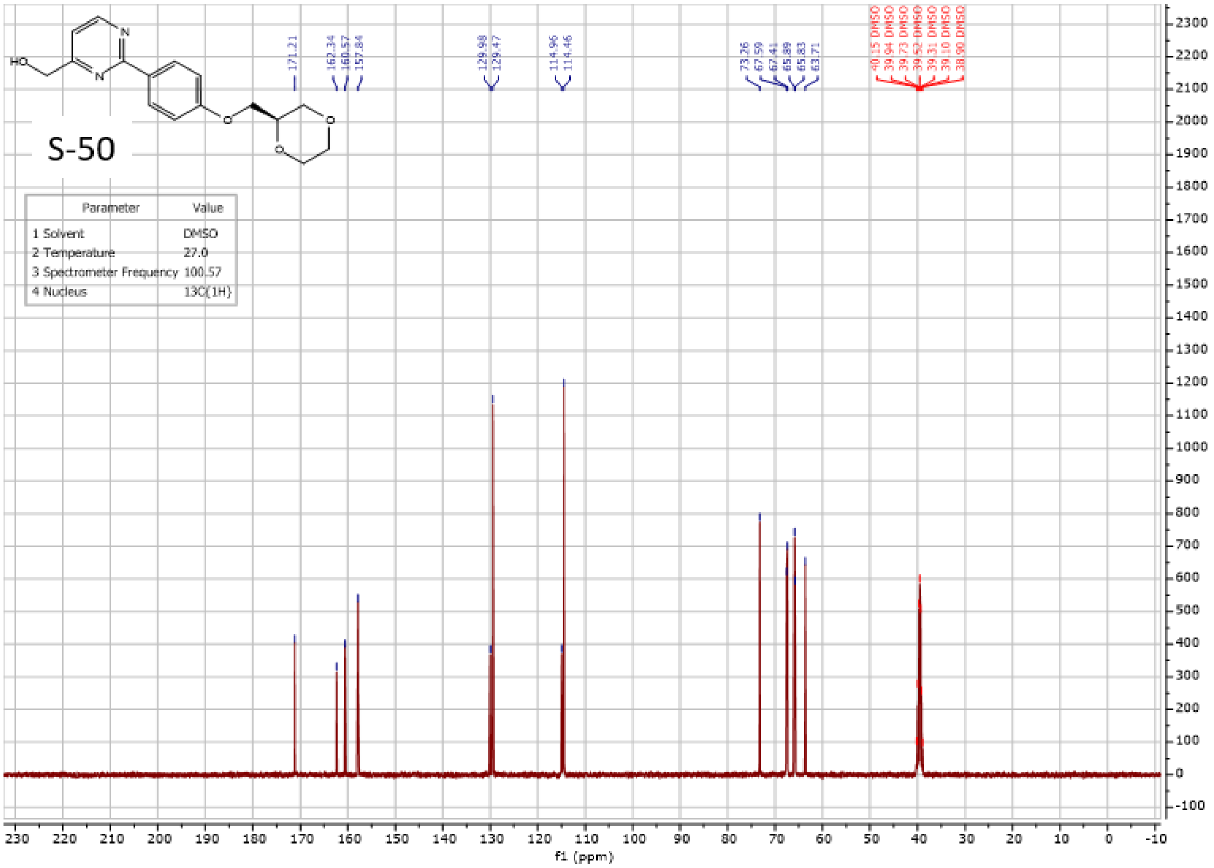

S-50
Parameter
Value
1 Solvent
DMSO
2 Temperature
27.0
3 Spectrometer Frequency
100.57
4 Nucleus
13C{1H}
171.21
162.34
160.57
157.84
129.98
129.47
114.96
114.46
73.26
67.59
67.41
65.89
65.83
63.71
f1 (ppm)

**Sample Name**	AO	**Position**	P2-C5	**Instrument Name**	Instrument 1	**User Name**	
**Inj Vol**	0.1	**InjPosition**		**SampleType**	Sample	**IRM Calibration Status**	Success
**Data Filename**	AO.d	**ACQ Method**	IMSERC_ESI_Pos_Main_	**Comment**		**Acquired Time**	5/1/2018 6:10:04 PM

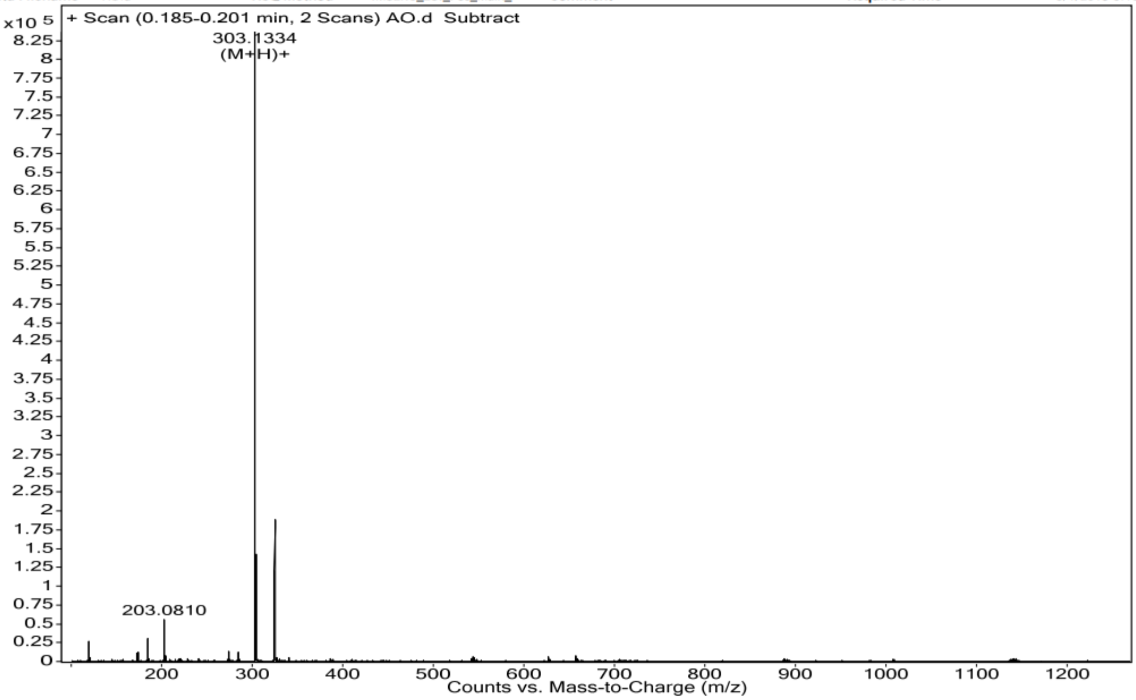

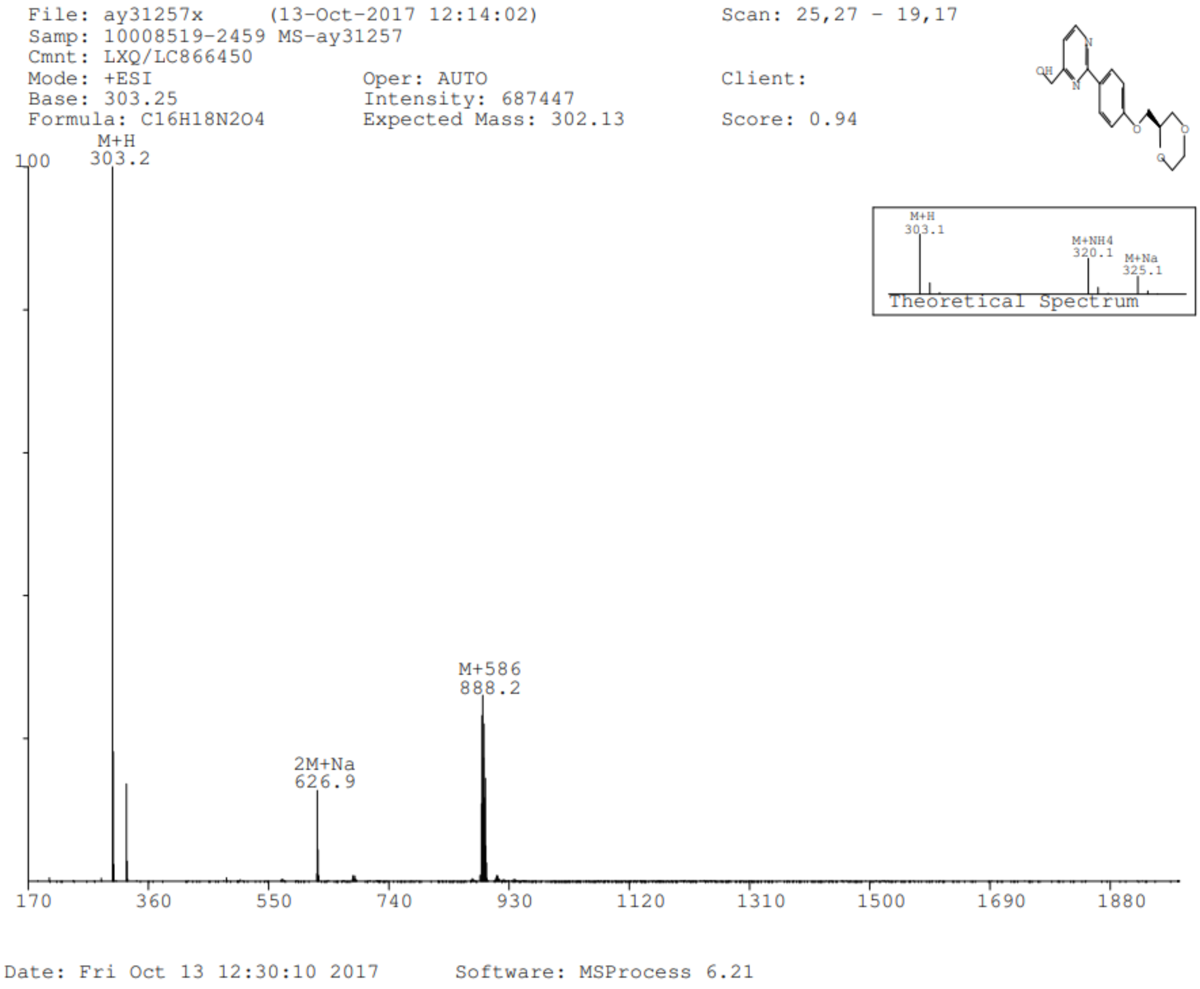

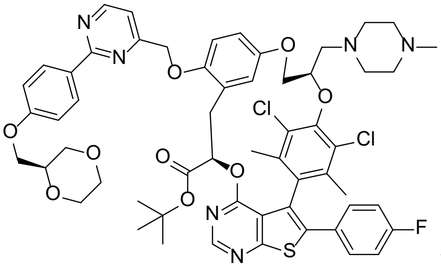

Tert-butyl (7R,16R)-19,23-dichloro-10-{[2-(4-{[(2R)-1,4-dioxan-2-yl]methoxy}phenyl)pyrimidin-4-yl]methoxy}-1-(4-fluorophenyl)-20,22-dimethyl-16-[(4-methylpiperazin-1-yl)methyl]-7,8,15,16-tetrahydro-18,21-etheno-13,9-(metheno)-6,14,17-trioxa-2-thia-3,5-diazacyclononadeca[1,2,3-cd]indene-7-carboxylate (S-51).

abbvie

10008519-2462 in DMSO $20 BC504 5mg
Temp = 27 C
C57H59N6O9F1S1Cl2
jr400

Acq: VnmrJ VERSION 3.2 REVISION A/jr400
Proc: VnmrJ VERSION 3.2 REVISION A/sasha

Chemist: BRYAN SORENSEN
Experiment: s2pul

nmr3473918

Oct 20 2017

Expected protons=59
Whole protons observed=58
Total integral =62.008

ID=81.70
P =88.00

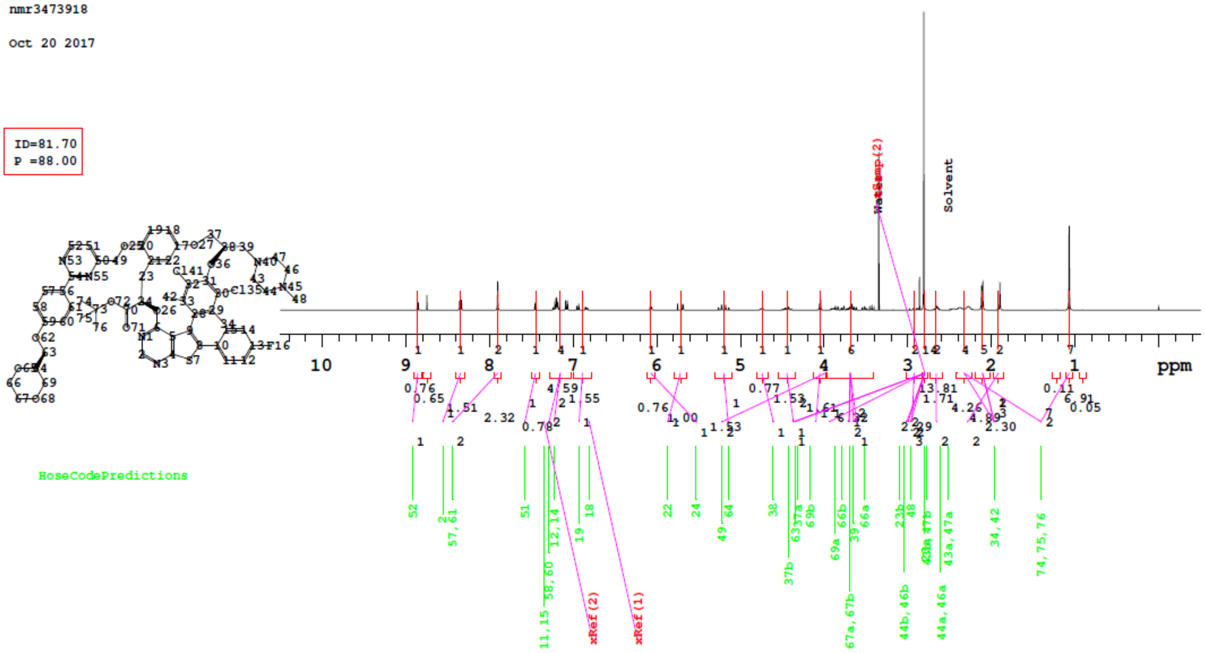

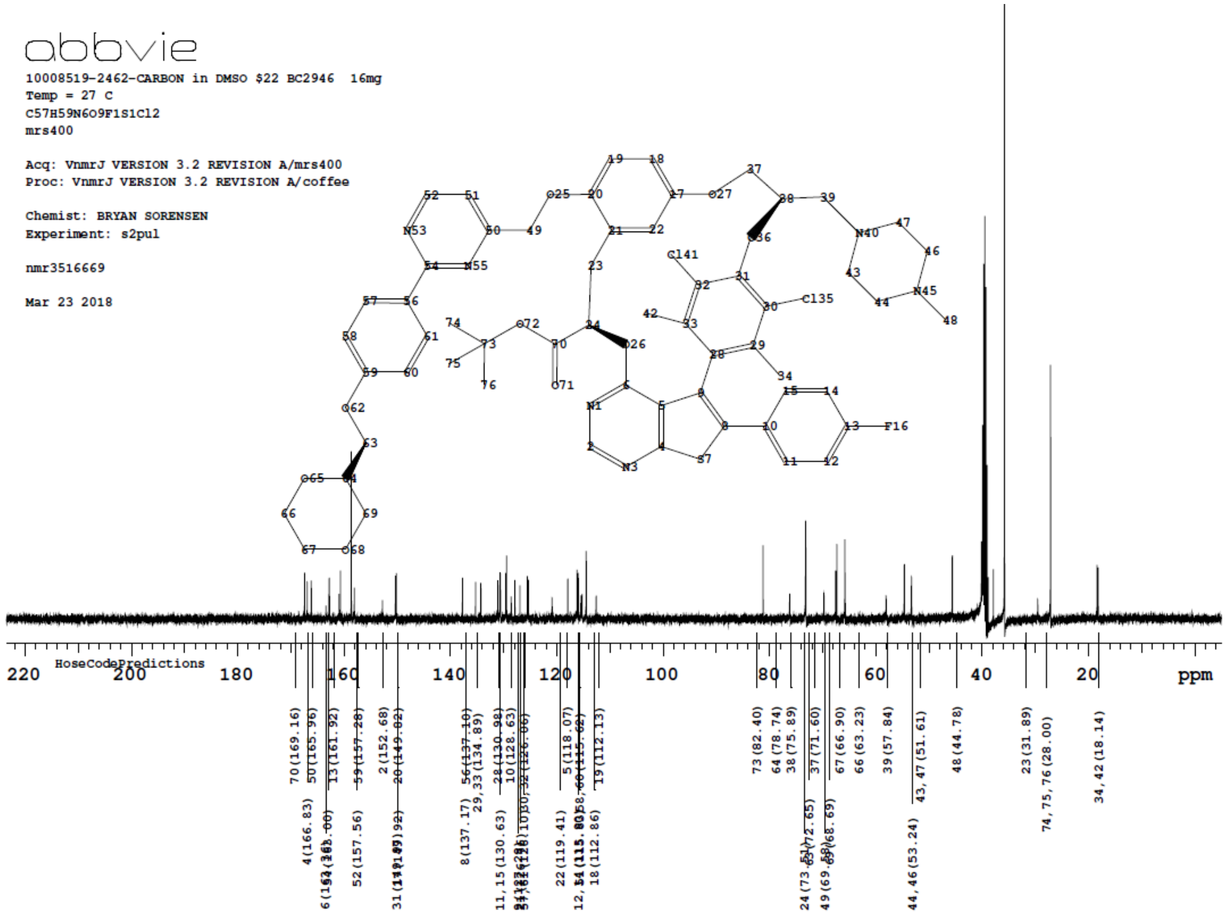
abbvie
10008519-2462-CARBON in DMSO $22 BC2946 16mg
Temp = 27 C
C57H59N6O9F1S1Cl2
mrs400
Acq: VnmrJ VERSION 3.2 REVISION A/mrs400
Proc: VnmrJ VERSION 3.2 REVISION A/coffee
Chemist: BRYAN SORENSEN
Experiment: s2pul
nmr3516669
Mar 23 2018
HoseCodePredictions
220 200 180 160 140 120 100 80 60 40 20 ppm
70(169.16)
50(165.96)
4(166.83)
13(161.92)
59(157.28)
52(157.56)
2(152.68)
20(149.82)
8(137.17)
56(137.10)
29,33(134.89)
28(130.98)
11,15(130.63)
10(128.63)
22(119.41)
5(118.07)
19(112.13)
18(112.86)
73(82.40)
64(78.74)
38(75.89)
37(71.60)
67(66.90)
66(63.23)
39(57.84)
43,47(51.61)
44,46(53.24)
48(44.78)
23(31.89)
74,75,76(28.00)
34,42(18.14)

**Sample Name**	AP	**Position**	P2-C6	**Instrument Name**	Instrument 1	**User Name**	
**Inj Vol**	0.1	**InjPosition**		**SampleType**	Sample	**IRM Calibration Status**	Success
**Data Filename**	AP.d	**ACQ Method**	IMSERC_ESI_Pos_Main_	**Comment**		**Acquired Time**	5/1/2018 6:13:08 PM

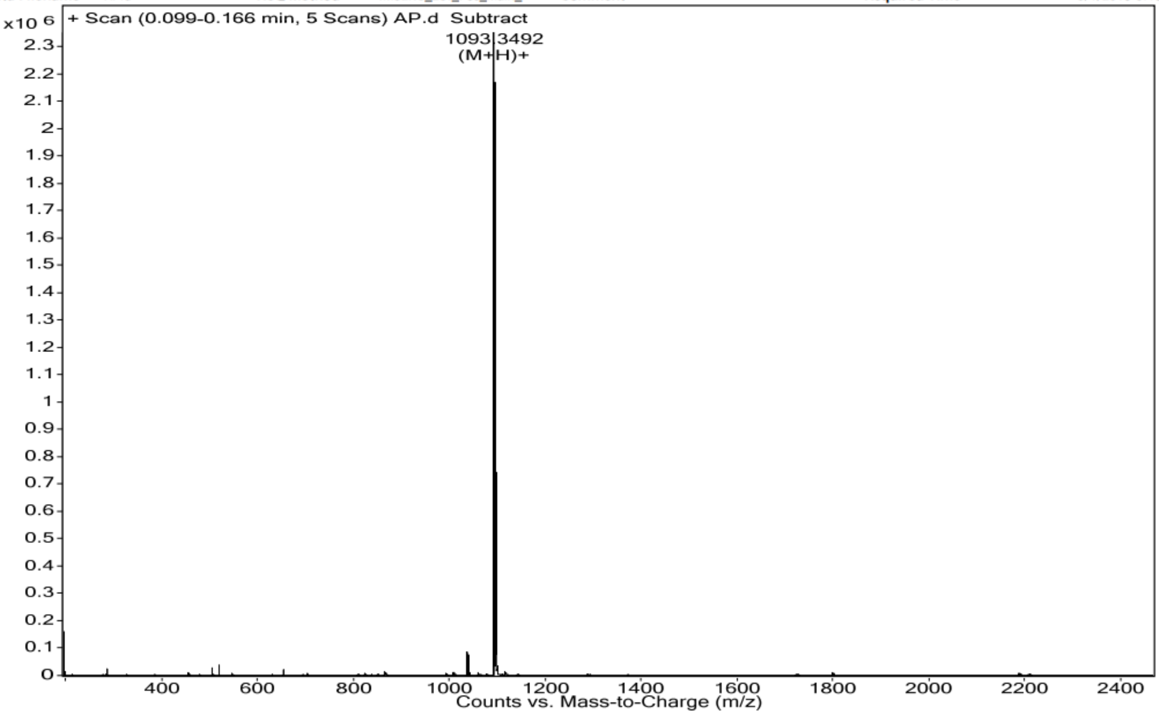

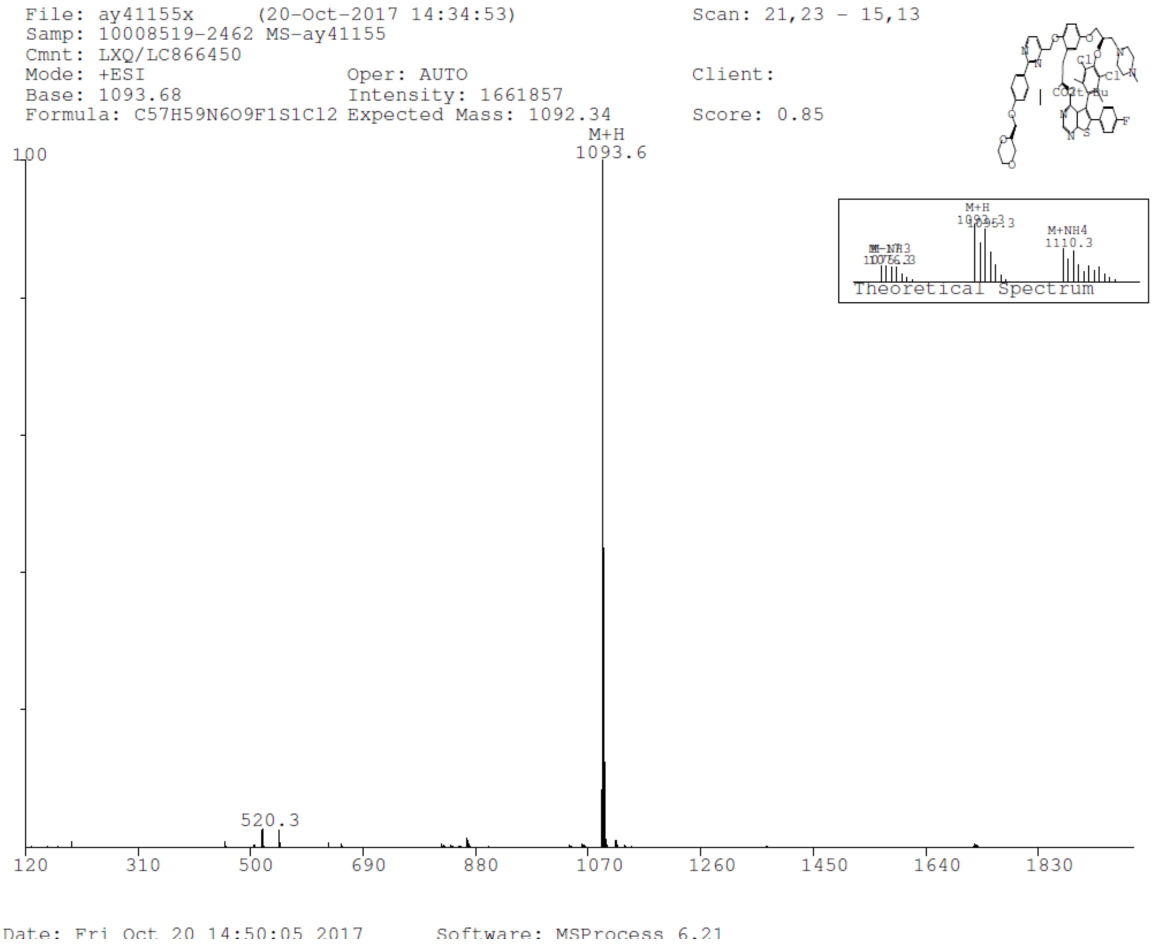

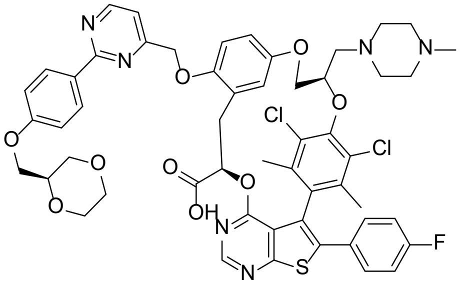

(7R,16R)-19,23-dichloro-10-{[2-(4-{[(2R)-1,4-dioxan-2-yl]methoxy}phenyl)pyrimidin-4-yl]methoxy}-1-(4-fluorophenyl)-20,22-dimethyl-16-[(4-methylpiperazin-1-yl)methyl]-7,8,15,16-tetrahydro-18,21-etheno-13,9-(metheno)-6,14,17-trioxa-2-thia-3,5-diazacyclononadeca[1,2,3-cd]indene-7-carboxylic acid (ABBV-467).

abbvie

10008519-2466-2-DAYS-HIGH-VAC-AT-60C in DMSO $02 BC1602 4mg
Temp = 27 C
C53H51N6O9F1S1Cl2
mrs400

Acq: VnmrJ VERSION 3.2 REVISION A/mrs400
Proc: VnmrJ VERSION 3.2 REVISION A/i600

Chemist: BRYAN SORENSEN
Experiment: s2pul

nmr3477483

Nov 2 2017

ID=87.70
P =95.00

Expected protons=51
Whole protons observed=49
Total integral =51.227

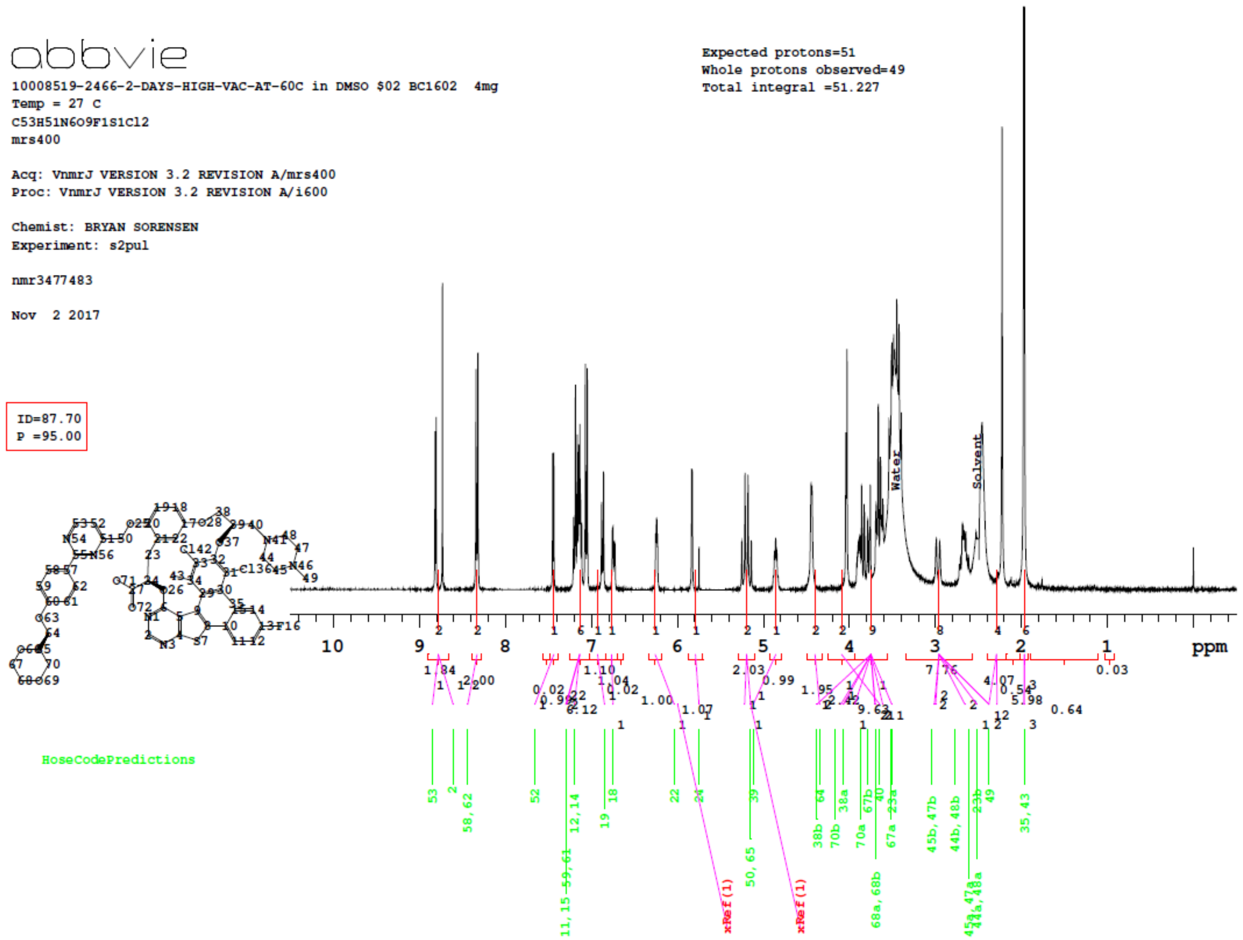

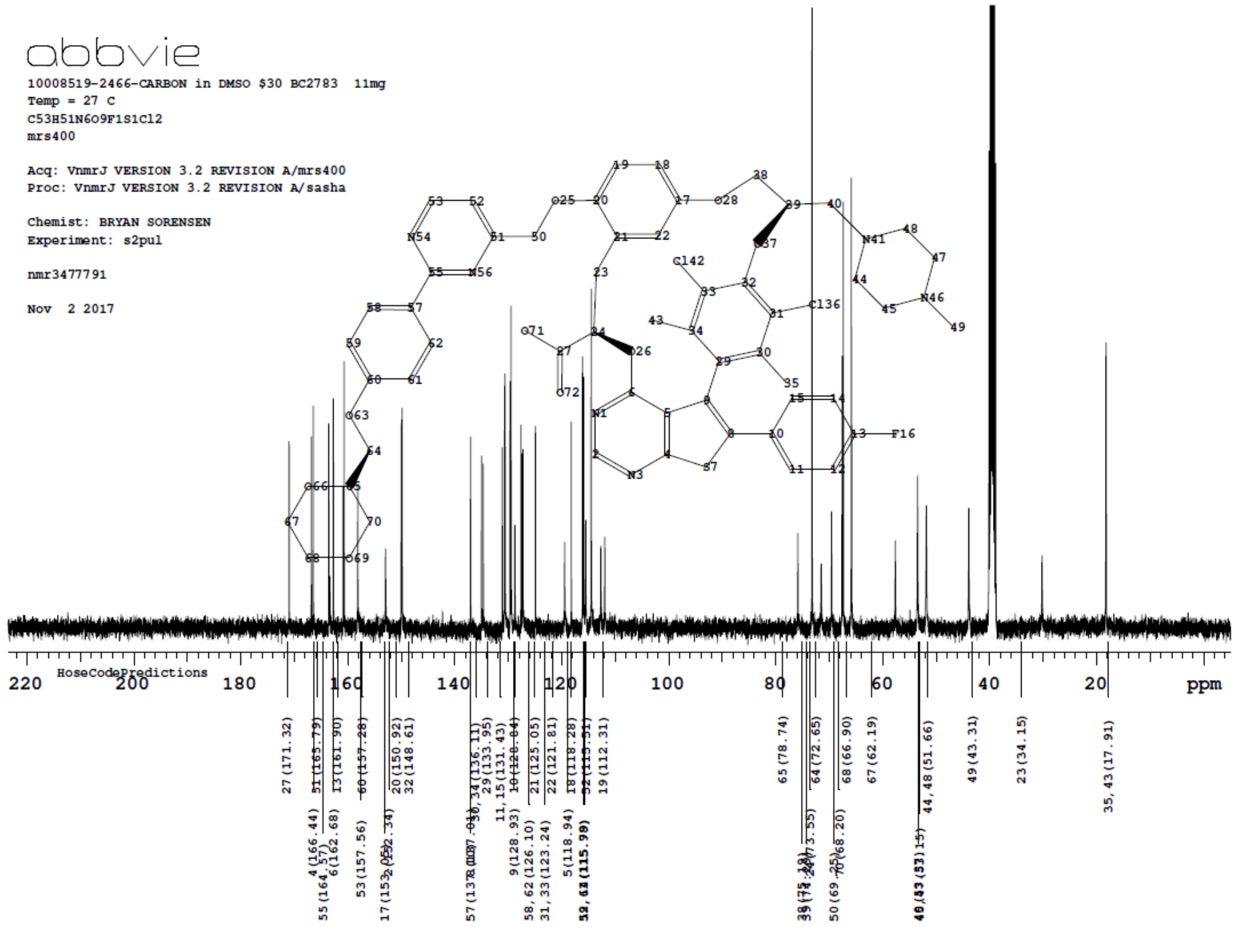
abbvie
10008519-2466-CARBON in DMSO $30 BC2783 11mg
Temp = 27 C
C53H51N6O9F1S1Cl2
mrs400
Acq: VnmrJ VERSION 3.2 REVISION A/mrs400
Proc: VnmrJ VERSION 3.2 REVISION A/sasha
Chemist: BRYAN SORENSEN
Experiment: s2pul
nmr3477791
Nov 2 2017
HoseCodePredictions
ppm

**Sample Name**	AQ	**Position**	P2-C7	**Instrument Name**	Instrument 1	**User Name**	
**Inj Vol**	0.1	**InjPosition**		**SampleType**	Sample	**IRM Calibration Status**	Success
**Data Filename**	AQ.d	**ACQ Method**	IMSERC_ESI_Pos_Main_	**Comment**		**Acquired Time**	5/1/2018 6:16:10 PM

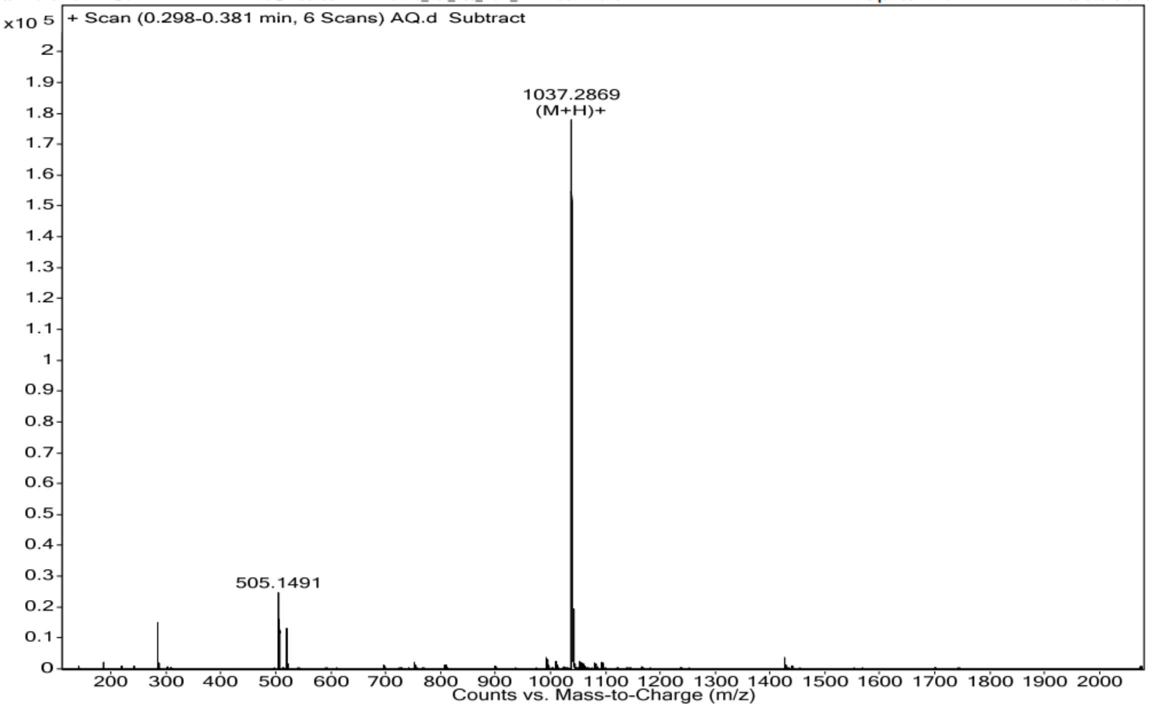

File: ay51238x (30-Oct-2017 07:38:05) Scan: 25,27 - 19,17
Samp: 10008519-2466-AFTER-DRYING MS-ay51238
Cmnt: LXQ/LC866450
Mode: +ESI Oper: AUTO Client:
Base: 1037.44 Intensity: 2130650
Formula: C53H51N6O9F1S1Cl2 Expected Mass: 1036.28 Score: 0.95

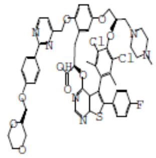

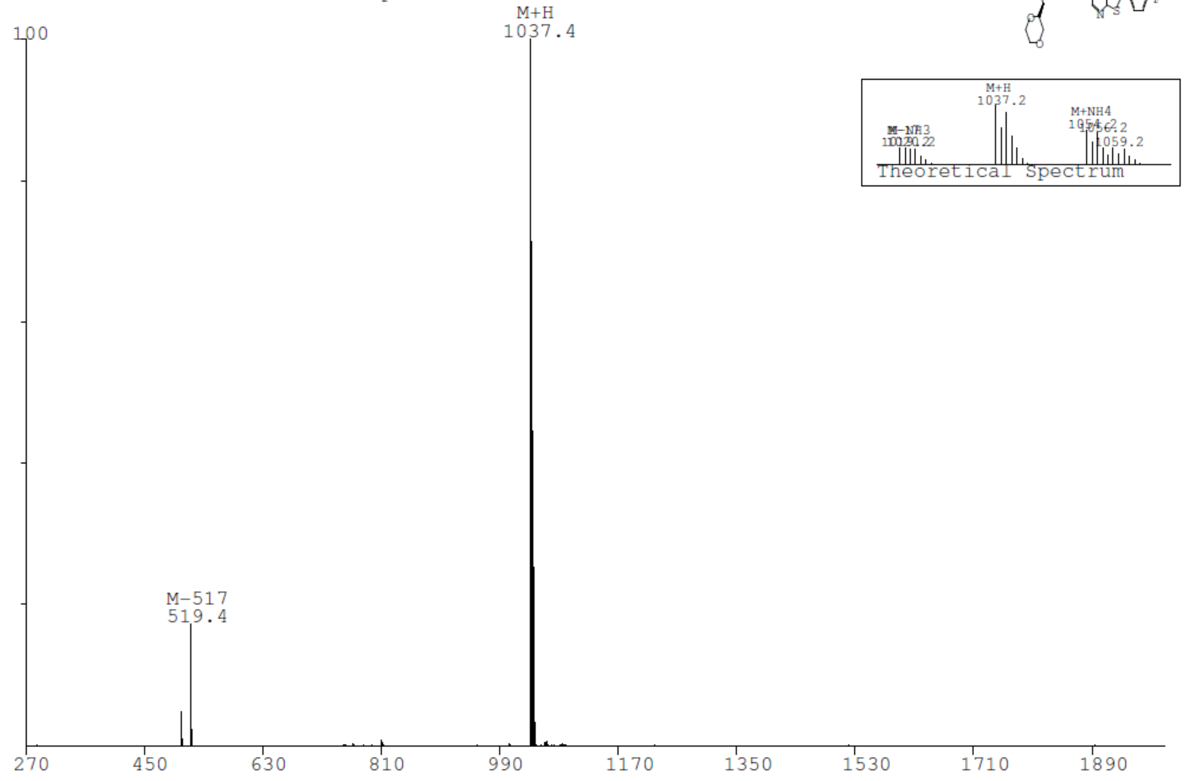

Date: Mon Oct 30 08:10:06 2017 Software: MSProcess 6.21

Phase 1 clinical trial

Inclusion/exclusion criteria. Subjects must meet all of the following criteria in order to be included in the study. Anything other than a positive response to the questions below will result in exclusion from study participation.

Consent

1. Subjects or their legally authorized representative must voluntarily sign and date an informed consent, approved by an independent ethics committee (IEC)/institutional review board (IRB), prior to the initiation of any screening or study-specific procedures.

Demographic and Laboratory Assessments

2. At least 18 years old.
3. Eastern Cooperative Oncology Group (ECOG) performance status 0, 1, or 2.
4. Laboratory values meeting the following criteria during the screening period prior to first dose of study drug:
    a. For subjects with AML, the following hematologic parameter requirements are not applicable. For subjects with MM, subjects must have adequate hematologic function, defined as follows:
    b. Absolute neutrophil count ≥ 1000/mm3;
    c. Platelet count ≥ 75,000/mm3;
    d. Hemoglobin ≥ 8 g/dL;
    e. Serum alanine aminotransferase (ALT) ≤ 2.5 x upper limit of normal (ULN);
    f. Serum aspartate aminotransferase (AST) ≤ 2.5 x ULN;
    g. Total bilirubin ≤ 1.5 x ULN (subject with documented Gilbert's syndrome may have a total bilirubin >1.5 x ULN);
    h. Amylase ≤ 2 x ULN;
    i. Lipase ≤ 2 x ULN;
    j. Creatinine clearance (CrCl) ≥ 60 mL/min, measured by 24-hour urine collection or calculated using the Cockcroft-Gault formula.
5. Echocardiogram with ejection fraction ≥ 50% and no other clinically significant finding that would increase the subject's susceptibility to cardiac toxicity.

6. No clinically relevant or significant ECG abnormalities, including ECG with QT interval corrected for heart rate (QTc) using Fridericia's formula (QTcF) > 450 msec (males) or > 470 msec (females).
7. Willingness and ability to comply with procedures required in this protocol.

Disease Activity

8. Documented diagnosis of MM (Parts A and B only).
9. For MM subjects only: measurable disease, defined as at least 1 of the following:
    a. Serum monoclonal protein ≥ 1 g/dL
    b. Urine M-protein ≥ 200 mg/24 hours
    c. Serum immunoglobulin free light chain (FLC) ≥ 10 mg/dL (100 mg/L), provided serum FLC ratio is abnormal
10. For MM subjects only: Subjects who have relapsed after or are refractory or intolerant to all established MM therapies that are both known to provide clinical benefit, and locally available. At least 3 prior lines of therapy must have been administered (Appendix E), including 1 or more immunomodulatory agents (e.g., lenalidomide or pomalidomide), 1 or more proteasome inhibitors (e.g., bortezomib or carfilzomib), and 1 or more anti-CD38 monoclonal antibodies (e.g., daratumumab).
11. Documented diagnosis of AML (Parts C and D only; subjects with APL are excluded throughout the study).
12. AML subjects only: Total white blood cell count < 25,000/mm3 (Note: hydroxyurea is permitted to meet this criterion).
13. For AML subjects only: failure to respond to, and/or relapse or progression after, at least 1 prior line of therapy, including all available standard therapies (per assessment of local investigator). Where applicable, this could include prior exposure to approved inhibitors of FLT3, IDH1, and IDH2.
14. No autologous stem cell transplant within 90 days prior to start of ABBV-467.
15. No allogeneic stem cell transplant within 180 days prior to start of ABBV-467.
16. No clinically significant graft versus host disease requiring ongoing systemic therapy.

Subject History

17. No unresolved clinically significant non-hematologic toxicity of ≥ Grade 2 from prior anticancer therapy.
18. No history of acute or chronic pancreatitis.

19. No significant unresolved liver disease. Subjects with hepatitis C who have received definitive treatment and are considered cured will be eligible.
20. No known history of hepatitis B or HIV infection. Testing for hepatitis B and HIV are not required as part of Screening.
21. No major surgery within 4 weeks of study treatment.
22. No history of central nervous system involvement by underlying disease.
23. No history of plasma cell leukemia.

Statistical analysis. Efficacy and safety analyses were performed on the all-treated population, which comprises all subjects who received at least 1 dose of study drug. The PK population is defined as subjects who received at least 1 dose of study drug and have at least 1 valid post-baseline PK assessment; PK analyses were based on the PK population.

Supplementary Tables and Figures

**Supplementary Table S1: X-ray diffraction statistics for the 3 compound complexes**			
	MCL-1: Compound 1	MCL-1: Compound 2	MCL-1: ABBV-467
PDB code	8EKX	8EL0	8EL1
Data collection			
Space group	P 21 21 2	P 21 21 2	P 1 21 1
Cell dimensions	a, b, c (Å): 97.36 136.89 38.61<br>α, β, γ (deg): 90.00, 90.00, 90.00	a, b, c (Å): 97.99 136.94 38.39<br>α, β, γ (deg): 90.00, 90.00, 90.00	a, b, c (Å): 111.38 79.62 137.85<br>α, β, γ (deg): 90.00, 90.22, 90.00
Mol/ASU	1	1	4
Resolution (Å)	1.55	1.92	2.41 (Staraniso)
Completeness (%)	99.8	99.8	47.3 (sphere)/87.9 (ellipsoidal)
Redundancy	6.4	5.6	3.3
I/σI	24.7	17.5	10.3
Rmerge (%)	3.6	5.8	6.8
Refinement			
R-work/R-free (%)	17.6/20.2	18.3/21.2	19.2/28.2
RMSD bonds (Å)	0.008	0.008	0.009
RMSD angles (°)	0.871	0.857	1.011
No. non-hydrogen atoms			
Protein	4020	4020	15474
Other	85	87	380
Water	550	391	183
B-factors ($Å^2$)	21.9	33.4	52.2
Ramachandran favored (%)	98.8	98.5	93.4
Ramachandran allowed (%)	1.2	1.5	6.1
Ramachandran disallowed (%)	0.0	0.0	0.5
PDB, protein data bank; RMSD, root-mean-square deviation.			

**Supplementary Table S2: TR-FRET assay protein/probe/antibody mixes**					
Protein	Probe	[Protein], nM	[Probe], nM	Antibody	[Antibody], nM
GST-BCL-X_L	F-Bak (GQVGRQLAIIGDK(6-FAM)INR-amide)	1	100	Tb-anti-GST	1
GST-BCL-2	F-Bak (GQVGRQLAIIGDK(6-FAM)INR-amide)	1	100	Tb-anti-GST	1
BCL-2, B-cell lymphoma 2; TR-FRET, time-resolved fluorescence resonance energy transfer.					

**Supplementary Table S3: Cellular potency and selectivity of ABBV-467 in engineered murine B-cell ALL cell lines**					
	B-Cell ALL Lines (EC_{50}, nM)				
	BCL-XL	BCL-2	BCL-w	BCL2A1	MCL-1
ABBV-467	>3,000	>3,000	>3,000	2,970*	20.3

Murine p185[+] B-ALL cells selectively expressing individual human anti–BCL-2 family members (*36*) were treated with ABBV-467 for 24 hours in RPMI-1640 media (Life Technologies, CA) containing 10% fetal bovine serum, 55 µM 2-mercaptoethanol, 2 mM glutamine, and 1% penicillin/streptomycin and the impact on cell viability determined using CellTiter-Glo. Each EC_{50} was calculated from the resulting dose-response curves. Data are presented as the mean of three independent experiments, except *(n=2). Where an EC_{50} could not be accurately determined, activity was defined as >3,000 nM.

ALL, acute lymphoblastic leukemia; BCL-2, B-cell lymphoma 2; EC_{50}, half maximal effective concentration.

**Supplementary Table S4: Binding affinity of compounds A-694 and ABBV-467 to BCL-2 family proteins**					
	**TR-FRET, Ki, nM**				
	MCL-1	BCL-2	BCL-X_L	BCL-W	BCL2-A1
A-694	<0.01	>447	>214	>127	267
ABBV-467	<0.01	>642	>376	>247	>402

BCL-2, B-cell lymphoma 2; Ki, dissociation constant; TR-FRET, time-resolved fluorescence resonance energy transfer.

**Supplementary Table S5: Cellular activity of A-694 and ABBV-467 in human tumor cell lines**				
	**AMO-1**<br>**EC_{50} (nM, 10% FBS)**	**H929**<br>**EC_{50} (nM, 10% FBS)**	**MV4;11**<br>**EC_{50} (nM, 10% FBS)**	**DLD-1**<br>**EC_{50} (nM, 10% FBS)**
**A-694**	**0.2**	**0.4**	**0.3**	**>1,000**
**ABBV-467**	**0.1**	**0.4**	**0.3**	**>1,000**

EC_{50}, half maximal effective concentration; FBS, fetal bovine serum.

**Supplementary Table S6: Major findings in safety pharmacology cardiovascular studies conducted with ABBV-467**				
Species/Strain	Route of Administration	Concentrations or Doses	Sex and N per Group	Results
hERG (human ether-a-go-go-related gene) current	In vitro	0.56, 2.23, 8.59, 20.3 µg/mL	N=7	hERG tail current IC_{50} >20.3 µg/mL (22% block)
Anesthetized dog Beagle aged 8-12 months	IV	1, 3, and 10 mg/kg	Male N=4	No effects up to a plasma concentration of 16.83 µg/mL on<br>• Blood pressure<br>• Heart rate<br>• Contractility<br>• Systemic vascular resistance<br>• Cardiac output<br>• QTcV<br>• QRS<br>• PR interval<br>At 67.23 mg/mL, an increase in heart rate and decrease in systemic vascular resistance were observed
Conscious dog beagle (aged 8-12 months at implantation; up to 4 years of age)	IV	0, 0.3, 1, and 3 mg/kg	Male N=5–6[a]	No effects on blood pressure, heart rate, or ECG parameters at any dose (plasma concentration of 6.53 µg/mL at 3-mg/kg dose)

ECG, electrocardiogram; IC_{50}, half maximal inhibitory concentration; IV, intravenous; QTcV, QT interval corrected according to the Van de Water formula. No animals or data were excluded from analysis. Studies were approved by the animal use committee of Abbvie, Lake County, IL. Healthy dogs, as determined by veterinary physical examination and clinical pathology, were selected for study based on availability without explicit randomization. Conscious dog telemetry utilized a Latin square design to randomize for dose order effects. The same 6 dogs received all doses. Power analysis has shown a group size of 6 can statistically detect an approximately 20% alteration in measured parameters with α=0.05. Dose groups were not blinded in any study. Statistical analysis of telemeterized data utilized paired sample t-test with Bonferroni correction. Anesthetized cardiovascular parameters were within 15% of control mean for mean arterial blood pressure, heart rate, systemic vascular resistance, and cardic output and within 10% of control mean for dP/dt (contractility), and within 10 msec of control QT; these criteria are considered biologically relevant but do not constitute a statistical analysis. Comparison was made to vehicle control data from 6 dogs collected within the preceding 3 month period. Detailed methods for the anesthetized dog study were previously published in J Pharmacol Toxicol Methods (2018) 91:27[1]. Detailed methods for the conscious telemetery study were previously published in J Pharmacol Toxicol Methods (2021) 111:107109[2]. Detailed data are Abbvie internal, not publically available at the time of this publication.

a. One dog in the conscious telemetery study did not receive its final dose (0.3 mg/kg) and the replicate number for 0.3 mg/kg, only, is instead N=5. This animal had received the 3 mg/kg dose the previous week and was excluded from further testing for animal welfare concerns to provide additional recovery time from gastrointestinal clinical signs.

Supplementary Table S7: Major findings in toxicology studies conducted with ABBV-467*			
Study	N and Sex per Group	Dosage (mg/kg/dose)	Primary target organ toxicities
4-week (5-dose) IV infusion with a 4-week recovery period in Sprague-Dawley rat (Crl:CD®(SD)) aged 10 weeks at initiation of dosing	• 10/sex (main)<br>• 5/sex for recovery (control, mid, high)	0, 10, 30, 60, 100	Effects observed in<br>• Pancreas – single-cell necrosis, increased amylase and lipase<br>• Liver – increased ALT/AST, multinucleated hepatocytes<br>• Bone marrow – decreased cellularity, red and white blood<br>• Gastrointestinal tract – epithelial degeneration and regeneration<br>• Lymphoid system – lymphoid cell depletion and/or necrosis in multiple lymphoid tissues<br>• Testes – degeneration/atrophy of seminiferous tubules<br>• Multiple other tissues – single-cell necrosis
4-week (5-dose) IV infusion with a 4-week recovery period in beagle dog aged 8-8.5 months at initiation of dosing	• 4/sex (main)<br>• 2/sex for recovery (control, mid, high)	0, 1, 3, 10	Mortality at 10 mg/kg after 1 or 2 doses (days 3–10) attributed to on-target effects in gastrointestinal tract, pancreas, and bone marrow<br>Effects observed in<br>• Pancreas – single-cell necrosis, acinar atrophy/necrosis, and increased amylase and lipase<br>• Liver – profound increases in ALT and AST, hepatocyte necrosis, bile cholestasis<br>• Bone marrow – decreased cellularity, red and white blood<br>• Gastrointestinal tract – necrosis and/or hemorrhage<br>• Lymphoid system – decreased circulating lymphocytes, lymphoid cell necrosis in multiple lymphoid tissues<br>• Testes – degeneration/atrophy of seminiferous tubules<br>• Multiple other tissues – single-cell necrosis

*Standard toxicology studies were conducted with ABBV-467 including one single-dose study in rats, 2-week (3-dose) dose range-finding studies, and 4-week (5-dose) with recovery GLP studies in rats and dogs. For all studies, test item was administered once weekly by 30-minute IV infusion. Standard GLP endpoints were assessed including in-life (body weights, clinical observations, eye exams), electrocardiograms (dogs only), clinical pathology (hematology, clinical chemistry, and coagulation) and anatomic pathology (macroscopic and microscopic observations and organ weights). All findings were considered dose dependent and partially to fully reversible. Study design was generally in compliance with FDA Redbook Guidance IV.C.3.a. and IV.C.3.b. sections on short-term toxicity studies with rodents and non-rodents, respectively (viz. https://fda.gov/regulatory-information/search-fda-guidance-documents/redbook-2000-ivc3a-short-term-toxicity-studies-rodents)[3]. Studies were approved by the animal use committee of Charles River Laboratories, Mattawan, MI. No animals or data were excluded from analysis. Healthy dogs, as determined by veterinary physical examination and clinical pathology, were randomized between dose groups using laboratory information management software. Dose groups were not

blinded. Dunnett's test was used for statistical comparisons against control in the laboratory information management software. Historical data analysis indicates parameter distribution is sufficiently normally distributed for the test used. No specific modifications were adopted in study execution to control for dose order or other confounders. Detailed data are Abbvie internal, not publically available at the time of this publication. ALT, alanine aminotransferase; AST, aspartate aminotransferase; GLP, Good Laboratory Practice; IV, intravenous.

Supplementary Table S8: Demographic characterization of patients in dose-escalation cohort				
	0.16 mg/kg (N=3)	0.32 mg/kg (N=2)	0.53 mg/kg (N=3)	Total (N=8)
Age, years, n (%)				
<65	1 (33)	2 (100)	1 (33)	4 (50)
≥65	2 (67)	0	2 (67)	4 (50)
Sex, n (%)				
Female	1 (33)	1 (50)	0	2 (25)
Male	2 (67)	1 (50)	3 (100)	6 (75)
Race, n (%)				
White	2 (67)	1 (60)	2 (67)	5 (63)
Black or African American	0	0	0	0
Asian	1 (33)	1 (50)	1 (33)	3 (38)
Other/multiple	0	0	0	0
Mean weight, kg (SD)	62.7 (20.7)	65.75 (13)	58.8 (12.0)	62.0 (14.0)
Mean height, cm (SD)	162.9 (20.3)	164.5 (9.3)	172.5 (13.1)	166.9 (14.2)
ECOG PS				
0	1 (33)	1 (50)	1 (33)	3 (38)
1	2 (67)	1 (50)	0	3 (38)
2	0	0	2 (67)	2 (25)
MM ISS staging, n (%)				
I	1 (33)	1 (50)	0	2 (25)
II	1 (33)	1 (50)	2 (67)	4 (50)
III	1 (33)	0	1 (33)	2 (25)
Plasmacytoma(s) at screening, n (%)				
Yes	0	1 (50)	2 (67)	3 (38)
No	3 (100)	1 (50)	1 (33)	5 (63)
Number of prior systemic therapies, median (range)	9 (6–10)	11 (7–15)	7 (7–7)	7 (6–15)
Prior transplants, n (%)				
Yes	2 (67)	2 (100)	0	4 (50)
No	1 (33)	0	3 (100)	4 (50)
Prior radiation therapy, n (%)				
Yes	0	1 (50)	1 (33)	2 (25)
No	3 (100)	1 (50)	2 (67)	6 (75)
Prior transfusion, n (%)				
Yes	2 (67)	0	0	2 (25)
No	1 (33)	2 (100)	3 (100)	6 (75)
ECOG, Eastern Cooperative Oncology Group; MM, multiple myeloma; ISS, International Staging System; PS, performance status; SD, standard deviation.				

**Supplementary Table S9: PK parameters of ABBV-467**							
Dose Level (mg/kg)	N*	T_{max}*,† (min)	C_{max}* (ng/mL)	AUC_t* (ng•h/mL)	AUC_{inf}* (ng•h/mL)	CL* (mL/h/kg)	$t_{1/2}$*,‡ (min)
Cycle 1 day 1							
0.16§	3	30.0 (30.0, 30.0)	201 (204, 22)	101 (102, 16)	103 (104, 16)	778 (784, 17)	16.1 (6.51)
0.32§	2	20.0, 30.0	344, 474	159, 306	167, 309	957, 517	50.8, 34.4
0.53§	3	30.0 (20.0, 30.0)	732 (742, 20)	428 (428, 2)	435 (435, 2)	610 (610, 2)	55.5 (4.66)
Cycle 1 day 15							
0.16	3	30.0 (20.0, 45.0)	406 (412, 20)	251 (252, 12)	256 (257, 10)	625 (627, 10)	33.6 (20.7)
0.32	2	20.0, 45.0	1720, 646	346, 933	940, 350	340, 914	23.0, 54.3
0.53	2	30.0, 30.0	1510, 1860	921, 1280	933, 1290	568, 410	63.3, 64.1

*Individual values are presented where N=2. †T_{max} values are presented as median (min, max). ‡Harmonic mean (pseudo SD). §Only half of this dose was given on cycle 1 day 1.
$AUC_{inf,}$ area under the plasma concentration-time curve from time 0 to infinity; $AUC_{t,}$ area under the plasma concentration-time curve from time 0 to last measurable time t; CL, clearance; $C_{max,}$ maximum observed plasma concentration; PK, pharmacokinetic; SD, standard deviation; $t_{1/2,}$ terminal phase elimination half-life; ,T_{max}, time to C_{max}, peak time.

**Supplementary Table S10: MCL-1 ortholog information and ABBV-467 affinity across species**				
	**Mouse** *Mus musculus*	**Rat** *Rattus norvegicus*	**Dog** *Canis lupus familiaris*	**Monkey** *Macaca sp.*
Protein name	Mcl1	Mcl1	MCL1	MCL1
Amino acid homology to human sequence	80.7%	82.7%	87.7%	97.1%
TR-FRET, Ki, µM*	0.001 131	0.000 081	0.000 039	0.000 030

**mcl1* cDNAs for mouse (NM_008562, AA152-308), rhesus monkey (iso4, NM_001108937, AA 171-327), dog (NM_001003016, AA171-327), rat (NM_021846, AA151-307) were PCR amplified and cloned into *E. coli* expression vector pGEX-6P-1 vector and the recombinant proteins were expressed in *E.coli* BL21(DE3)-T1R and purified by affinity chromatography using GST column. TR-FRET assays were performed using the following conditions: 100 nM F-BAK peptide, 1 nM of mouse mcl-1 protein or 0.67 nM of rat protein, or 0.5 nM of dog protein, or 0.5 nM of rhesus monkey protein.

Amino acid sequence homology determined with NCBI BLAST (http://blast.ncbi.nlm.nih.gov). *M. mulatta* used instead of *M. fasciularis* for homology due to incomplete sequence information for the latter.

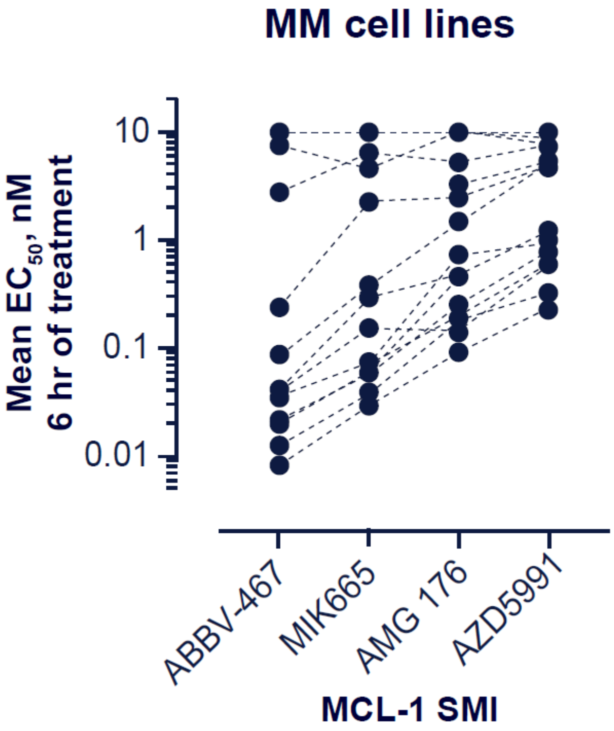

Supplementary Fig. S1: Cellular potency of ABBV-467 and other clinical-stage MCL-1 inhibitors in multiple myeloma (MM) cell lines in vitro. A panel of human MM cell lines were treated with ABBV-467, MIK665, AMG 176, or AZD5991 for 6 hours in media containing 10% FBS, and the impact on cell viability compared with the parental cell line determined by CellTiter-Glo. Mean EC_{50} values were calculated from the dose-response curves of three independent experiments. Dotted lines connect individual MM cell lines across. EC_{50}, half maximal effective concentration; FBS, fetal bovine serum; SMI, small molecule inhibitor.

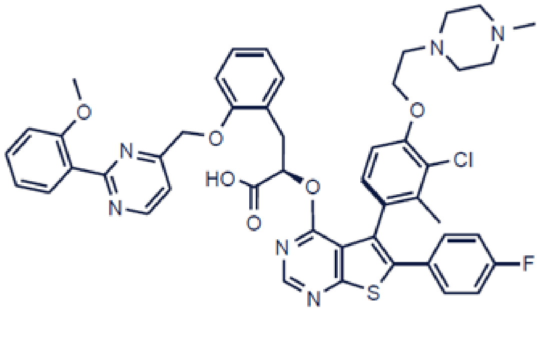

1 (MIK665)

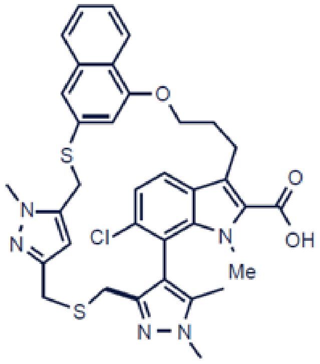

AZD5591

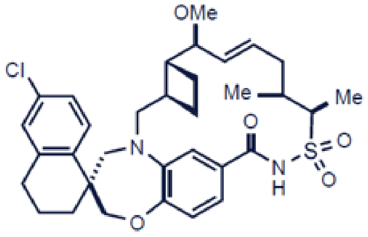

AMG 176

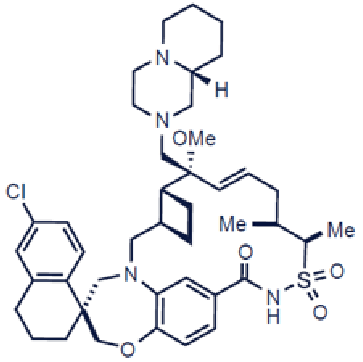

AMG 397

	FRET Ki (nM)*				
	MCL-1	BCL-2	BCL-XL	BCL2-A1	BCL-W
**MIK665**	0.044	541	>660	>468	>2280
**AZD5991**	0.044	541	>660	>468	>468
**AMG 176**	0.25	90.4	322	168	177
**AMG 397**	0.45	9.93	61.3	NA	NA

*See Methods; data derived from at least two independent experiments.

Supplementary Fig. S2: Chemical structures of other MCL-1 inhibitors that have been evaluated in the clinic and table of binding affinity (Ki) to BCL-2 family proteins. BCL-2, B-cell lymphoma 2; FRET, fluorescence resonance energy transfer; NA, not available.

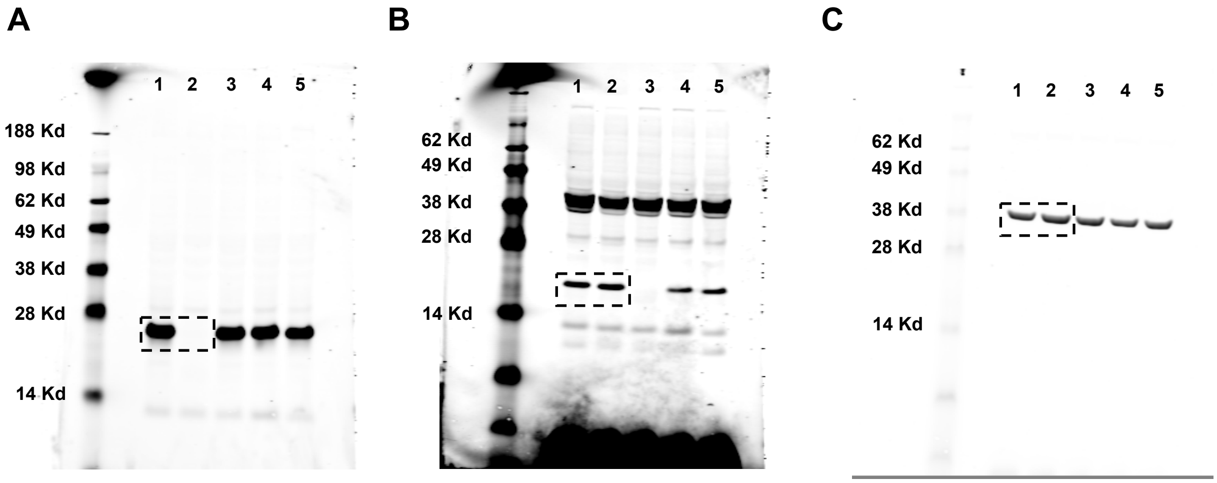

Supplementary Fig. S3: Uncropped images for western blots featured in the insert of Figure 2D. Parental (Lane 1), *BAK*-deficient (Lane 2), *BAX*-deficient (Lane 3), *BCL2L11*-deficient (Lane 4) & *PMAIP1*-deficient (Lane 5) SKBR3 cells were assessed for expression of BAK (A), BAX (B) and GAPDH (C) by western blot. Each of these 3 proteins were probed on separate membranes. Broken box marks the approximate borders of the final cropped images employed in the insert of Figure 2D.

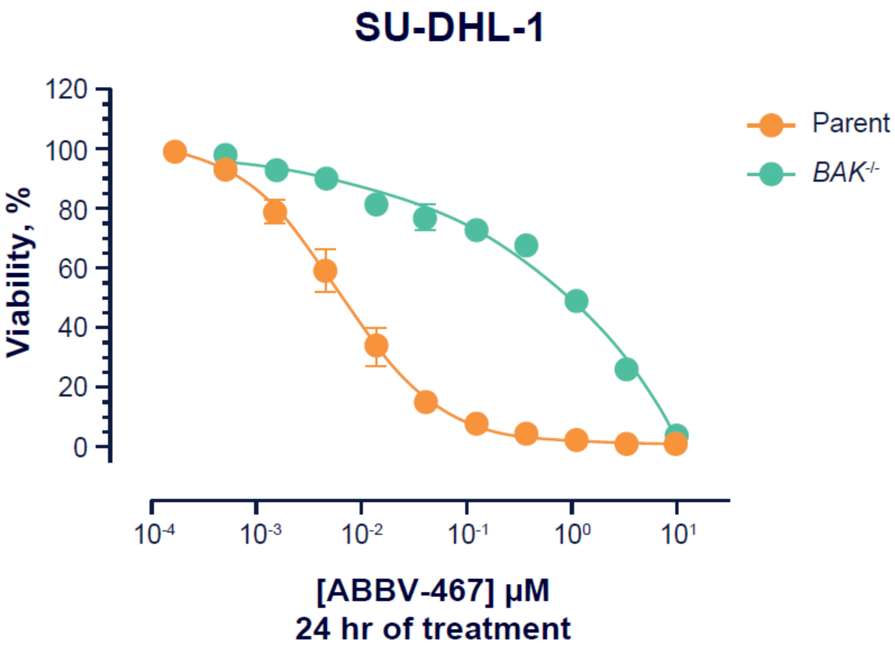

Supplementary Fig. S4: BAK is required for ABBV-467 activity in SU-DHL-1 cells. *BAK*-deficient SU-DHL-1 cells were treated with ABBV-467 for 24 hr at the indicated concentrations and the impact on cell viability compared to the parental cell line determined by CellTiter-Glo. N=3.

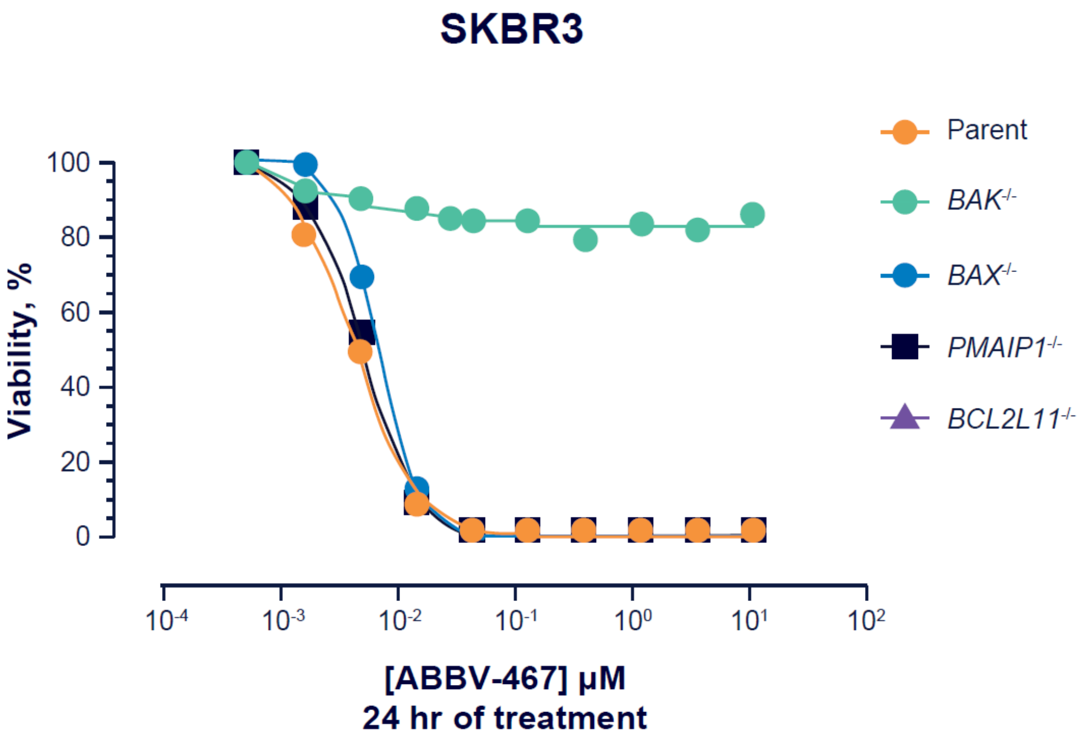

Supplementary Fig. S5: SKBR3 cells deficient in *BAX, BAK*, *PMAIP1* (gene encoding the protein NOXA), *BCL2L11* (gene encoding the protein BIM) were treated with ABBV-467 for 24 hours and the impact on cell death compared to the parental cell line determined by CellTiter-Glo™.

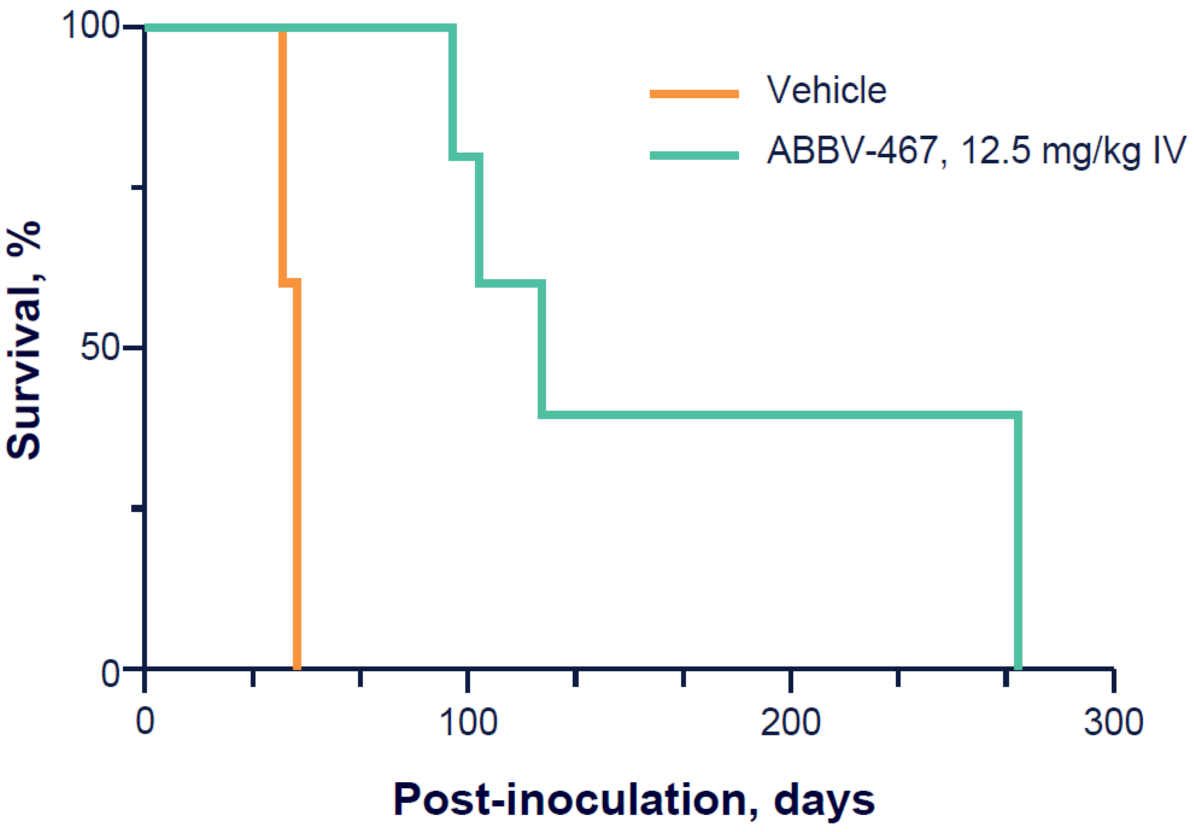

Supplementary Fig. S6: Survival of mice bearing H929 tumor xenografts following a single dose of ABBV-467. NCI-H929 xenografts dosed at a single dose of 12.5 mg/kg (n=5); Kaplan-Meier curve of the NCI-H929 xenograft study comparing vehicle to ABBV-467-treated group. To stay within IACUC guidelines, animals above a tumor volume 2,000 mm^3, with tumor ulcerations, or in poor health were removed from the study. IACUC, Institutional Animal Care and Use Committee; IV, intravenous.

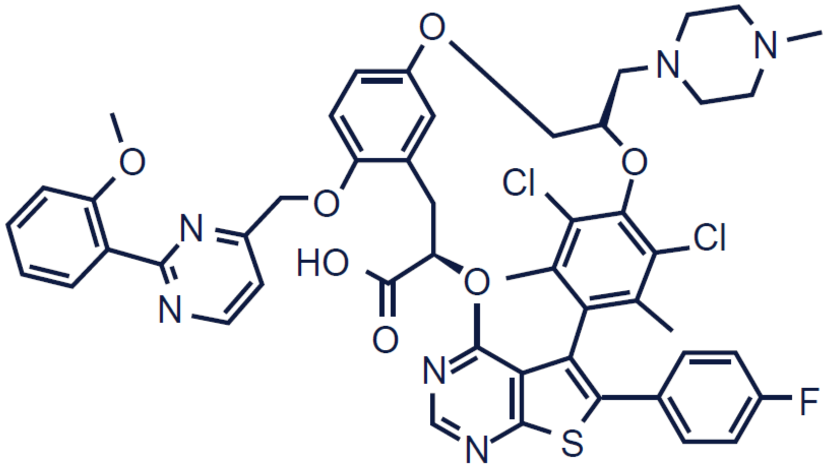

A-694

Supplementary Fig. S7: Chemical structure of A-694, a potent and selective MCL-1 inhibitor.

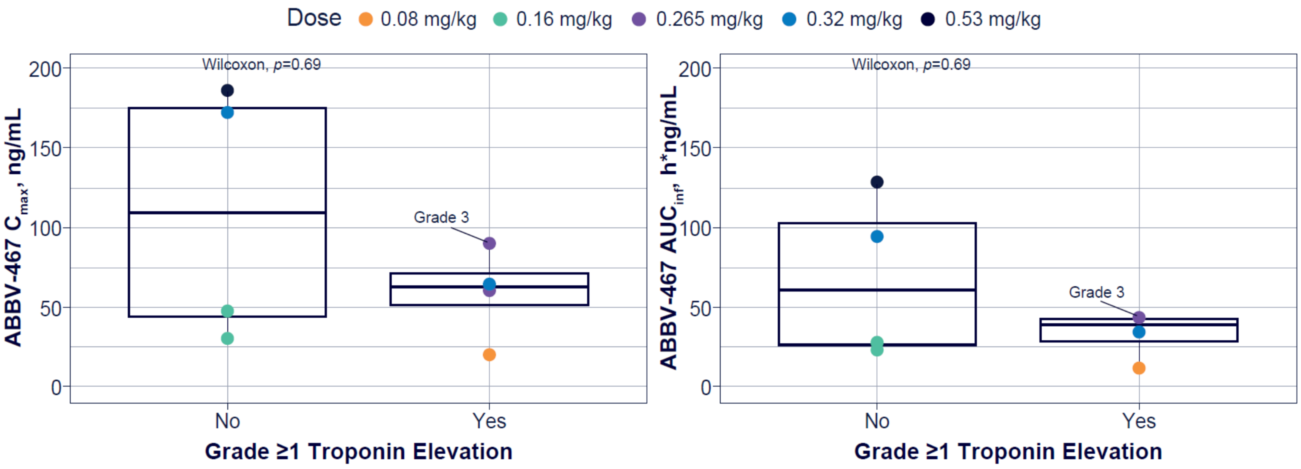

Supplementary Fig. S8: Grade 1 or higher troponin elevations by C_{max} or AUC_{inf}. Since ABBV-467 was undetectable 8 hours post-infusion, the last available measurements estimating C_{max} or AUC_{inf} of ABBV-467 was correlated to the occurrence of troponin elevations across the studied dose range. AUC_{inf}, area under the plasma concentration-time curve from time 0 to infinity; C_{max}, maximum observed plasma concentration.

Supplementary References

1. Koshman, Y. E., Herzberg, B. R., Seifert, T. R., Polakowski, J. S., Mittelstadt, S. W. The evaluation of drug-induced changes in left ventricular function in pentobarbital-anesthetized dogs. *J. Pharmacol. Toxicol. Methods* **91**, 27-35 (2018).

2. Koshman, Y. E., *et al*. Automated blood sampling in canine telemetry studies: Enabling enhanced assessments of cardiovascular liabilities and safety margins. *J. Pharmacol. Toxicol. Methods* **111**, 107109 (2021).

3. Toxicological Principles for the Safety Assessment of Food Ingredients. *U.S. Food and Drug Administration* http://https://www.fda.gov/regulatory-information/search-fda-guidance-documents/guidance-industry-and-other-stakeholders-redbook-2000#TOC (2023).

abbvie

Protocol for Study M19-025

Multiple Myeloma and Acute Myeloid Leukemia: A First-in-Human Study of an MCL-1 Inhibitor

VERSION:	3.0	DATE:	29 April 2020
SPONSOR:	AbbVie Inc.*	NUMBER OF SITES:	25
ABBVIE INVESTIGATIONAL PRODUCT:	ABBV-467	EudraCT:	2018-003744-24

FULL TITLE: A First-in-Human Study of the MCL-1 Inhibitor, ABBV-467

Incorporating Versions 1.0, 1.1 (Japan-only), 2.0, and 3.0.

PRINCIPAL INVESTIGATOR(S): Investigator information on file at AbbVie.

SPONSOR/EMERGENCY MEDICAL CONTACT:*

[redacted]
AbbVie Inc.
[redacted]
1 North Waukegan Road
North Chicago, IL 60064

Office: [redacted]
Mobile:
Email:

EMERGENCY 24-hour Number: +1 973-784-6402

*The specific contact details of the AbbVie legal/regulatory entity (person) within the relevant country are provided within the clinical trial agreement with the Investigator/Institution and in the Clinical Trial Application with the Competent Authority. Additional study contact information can be found in the Operations Manual (Appendix J).

CONFIDENTIAL INFORMATION
No use or disclosure outside AbbVie is permitted without prior written authorization from AbbVie.

abbvie

TABLE OF CONTENTS

1 SYNOPSIS 5

2 INTRODUCTION 9

2.1 BACKGROUND AND RATIONALE 9

2.2 BENEFITS AND RISKS TO SUBJECTS 9

3 OBJECTIVES AND ENDPOINTS 10

3.1 PRIMARY ENDPOINTS 10

3.2 SECONDARY ENDPOINTS 10

3.3 ADDITIONAL EFFICACY ENDPOINTS 11

3.4 BIOMARKER RESEARCH ENDPOINTS 11

4 INVESTIGATIONAL PLAN 11

4.1 OVERALL STUDY DESIGN AND PLAN 11

4.2 DISCUSSION OF STUDY DESIGN 14

5 STUDY ACTIVITIES 16

5.1 ELIGIBILITY CRITERIA 16

5.2 CONTRACEPTION RECOMMENDATIONS 19

5.3 PROHIBITED MEDICATIONS AND THERAPY 20

5.4 PRIOR AND CONCOMITANT THERAPY 20

5.5 WITHDRAWAL OF SUBJECTS FROM TREATMENT AND DISCONTINUATION OF STUDY 21

5.6 FOLLOW-UP FOR SUBJECT WITHDRAWAL FROM STUDY TREATMENT OR FROM STUDY 23

5.7 STUDY DRUG 23

5.8 RANDOMIZATION/DRUG ASSIGNMENT 24

5.9 PROTOCOL DEVIATIONS 24

6 SAFETY CONSIDERATIONS 24

6.1 COMPLAINTS AND ADVERSE EVENTS 24

6.2 TOXICITY MANAGEMENT 29

6.3 DOSE-LIMITING TOXICITY 34

7 STATISTICAL METHODS & DETERMINATION OF SAMPLE SIZE 35

7.1 Statistical and Analytical Plans 35
7.2 Definition for Analysis Populations 35
7.3 Statistical Analyses for Efficacy 35
7.4 Statistical Analyses for Pharmacokinetics 36
7.5 Statistical Analyses for Safety 36
7.6 Multiplicity Adjustment and Overall Type I Error Control 37
7.7 Sample Size Determination 37

8 ETHICS 38
8.1 Independent Ethics Committee/Institutional Review Board 38
8.2 Ethical Conduct of the Study 38
8.3 Subject Confidentiality 38

9 SOURCE DOCUMENTS AND CASE REPORT FORM COMPLETION 38

10 DATA QUALITY ASSURANCE 39

11 COMPLETION OF THE STUDY 39

12 REFERENCES 39

LIST OF TABLES

TABLE 1. DOSE DE-ESCALATION/ESCALATION DECISION RULES 13

TABLE 2. PROPOSED DOSE LEVELS 13

TABLE 3. STUDY DRUG INFORMATION 24

TABLE 4. AML-SPECIFIC ABBV-467 DOSE MODIFICATIONS FOR DRUG-RELATED HEMATOLOGIC TOXICITIES 30

TABLE 5. EXACT 1-SIDED 80% CONFIDENCE INTERVALS FROM VARIOUS RESPONSE RATES 38

LIST OF FIGURES

FIGURE 1. STUDY DIAGRAM 12

LIST OF APPENDICES

APPENDIX A. STUDY SPECIFIC ABBREVIATIONS AND TERMS 41

APPENDIX B. RESPONSIBILITIES OF THE INVESTIGATOR 44

APPENDIX C. LIST OF PROTOCOL SIGNATORIES 45

APPENDIX D. ACTIVITY SCHEDULE 46

APPENDIX E. DEFINITION OF LINES OF THERAPY (MULTIPLE MYELOMA ONLY) 49

APPENDIX F. DEFINITIONS OF LABORATORY AND CLINICAL TUMOR LYSIS SYNDROME 50

APPENDIX G. RECOMMENDATIONS FOR INITIAL MANAGEMENT OF ELECTROLYTE IMBALANCES AND PREVENTION OF TUMOR LYSIS SYNDROME 51

APPENDIX H. MONITORING AND MANAGEMENT OF DRUG-INDUCED LIVER INJURY 55

APPENDIX I. PROTOCOL SUMMARY OF CHANGES 57

APPENDIX J. OPERATIONS MANUAL 59

CONFIDENTIAL INFORMATION
No use or disclosure outside AbbVie is permitted without prior written authorization from AbbVie.

abbvie

1 SYNOPSIS

Title: A First-in-Human Study of MCL-1 Inhibitor ABBV-467	
**AbbVie Inc.**	**Protocol Number: M19-025**
**Name of Study Drug: ABBV-467**	**Phase of Development: 1**
**Background and Rationale:**	Although remarkable advances in multiple myeloma (MM) have emerged with the introduction of several new, more effective, and less toxic therapies, it remains an incurable disease. Treatment paradigms for MM include immunomodulatory agents, proteasome inhibitors, monoclonal antibodies, and corticosteroids, often in 2- or 3-drug combinations. However, the majority of patients will eventually relapse after, or become refractory to, all available therapies, in which case prognosis is poor. There remains a clear unmet need to identify agents with novel mechanisms of action for use alone and in combination with existing standards of care, to improve outcomes for patients with MM.<br>Acute myeloid leukemia (AML) is a heterogenous disease characterized by high unmet need. Current treatment strategies are still largely based on combination chemotherapy and stem cell transplantation, but the toxicities associated with this approach limits its utility in elderly and unfit patients, and outcomes for all patients with relapsed/refractory (R/R) AML are poor. As therapeutic progress in AML has been modest, opportunities for novel agents offering improved efficacy, reduced toxicity, or both, remain.<br>Myeloid cell leukemia-1 (MCL-1), a member of antiapoptotic BCL-2 family proteins, is a key regulator of cell survival and homeostasis. Frequent overexpression of MCL-1 has been observed in multiple cancer types where it acts as a resistance factor to various chemotherapeutic and targeted agents. Therapeutic targeting of induced myeloid leukemia cell differentiation protein (MCL-1), alone and in combination with other standard-of-care agents, enhances apoptotic death in preclinical MM and AML models, and offers a novel approach to treating these hematologic malignancies with progressive morbidity and eventual mortality.<br>The first-in-human study will evaluate the safety and tolerability of ABBV-467 in subjects with either R/R MM or R/R AML. ABBV-467 is a high-affinity MCL-1 inhibitor that exhibits robust preclinical efficacy in MM and AML models. The pharmacology, toxicology, and pharmacokinetic (PK) profiles of ABBV-467 have been adequately characterized to support an acceptable rationale and risk/benefit profile for treating adult subjects with MM or AML with ABBV-467 within the context of a clinical trial.
**Objectives and Endpoints:**	<u>Primary Objectives</u><br>• To characterize the safety and toxicity profiles of ABBV-467 in subjects with R/R MM and R/R AML<br>• To determine the recommended Phase 2 dose (RP2D) of ABBV-467 in subjects with R/R MM and R/R AML<br>• To determine the PK of ABBV-467<br><u>Secondary Objective</u><br>• To preliminarily evaluate the efficacy of ABBV-467 in R/R MM and R/R AML

	Safety evaluations include, but are not limited to, adverse event (AE) monitoring, physical examinations, vital sign measurements, electrocardiogram (ECG), cardiac enzyme monitoring, and clinical laboratory testing (hematology and chemistry) as a measure of safety and tolerability for the entire study duration.<br>Pharmacokinetic parameters to be determined for ABBV-467 will include maximum observed concentration (C_{max}), half-life ($t_{1/2}$), area under the concentration-time curve from time 0 to time of last measurable concentration (AUC_t), area under the concentration-time curve (AUC) from time 0 to infinity ($AUC_{0-\infty}$), and clearance (CL) using noncompartmental methods.<br>For subjects with R/R MM, efficacy will be evaluated based on adapted International Myeloma Working Group criteria. For subjects with R/R AML, efficacy will be evaluated based on adapted International Working Group and European Leukemia Net criteria.
**Investigators:**	Multicenter: Investigator information is on file at AbbVie.
**Study Sites:**	Up to 25 sites in 7 countries including but not limited to: United States, Australia, France, Israel, Japan, Spain, and Taiwan.
**Study Population and Number of Subjects to be Enrolled:**	Eligible subjects will have R/R MM or R/R AML for which few established therapies are available. A total of approximately 108 subjects with R/R MM or R/R AML will be enrolled into this first-in-human study. This trial will consist of 2 parts: dose escalation and dose expansion. Including a minimum of 6 subjects treated during dose escalation at what is ultimately declared the RP2D, it is estimated that 30 subjects will be treated in each disease indication during dose escalation and approximately 20 response-evaluable subjects (with a maximum of 24 subjects enrolled total) will be enrolled into each of the dose expansion cohorts.
**Investigational Plan:**	This is a Phase 1, first-in-human, open-label, dose escalation and dose expansion study evaluating ABBV-467 in subjects with R/R MM and R/R AML. Dosing will be weekly, on a 28-day cycle, though alternative dosing frequencies may be studied if supported by the totality of safety, efficacy, PK, and/or pharmacodynamic (PD) data.<br>Dose escalation will be guided by a Bayesian optimal interval (BOIN) design based on the cumulative number of subjects who experience a dose-limiting toxicity (DLT) in a given ABBV-467 dose level. A minimum of 2 - 3 subjects will be treated at each dose level.<br>Subjects with R/R MM (Part A) will receive escalating doses of ABBV-467 to identify a pharmacologically and/or clinically active and tolerable dose based on review of the totality of safety, efficacy, PK and PD data. This dose will be used to commence dose escalation in R/R AML (Part C), where provisional dose de-escalation will also be permitted. In order to identify the optimal biological dose (OBD) and/or select the RP2D, safety, tolerability, efficacy, PK, and PD data will be reviewed on an ongoing basis during dose escalation in R/R MM (Part A) and R/R AML (Part C). This may result in distinct RP2D between MM and AML.<br>Subjects with R/R MM (Part B) and R/R AML (Part D) will receive the ABBV-467 at the RP2D determined in Parts A and C, respectively. At the joint discretion of the sponsor and the investigators, subjects may be prospectively selected for Parts B and D based on any of the following:<br>1. Only those with prior exposure to a BCL-2 inhibitor<br>2. Only those naïve to prior BCL-2 inhibitor

abbvie

	3. Irrespectively of prior BCL-2 inhibitor exposure<br>Standard statistical, clinical, and laboratory procedures will be utilized in this study. All efficacy measurements are standard for assessing disease activity in subjects with hematologic malignancies. All clinical and laboratory procedures are also standard and generally accepted.
**Key Eligibility Criteria:**	Adults who meet the following key eligibility criteria:<br>• Documented diagnosis of MM (Parts A and B only)<br>• For MM subjects only: measurable disease, defined as at least 1 of the following:<br>• Serum monoclonal protein ≥ 1 g/dL<br>• Urine M-protein ≥ 200 mg/24 hours<br>• Serum immunoglobulin free light chain (FLC) ≥ 10 mg/dL (100 mg/L), provided serum FLC ratio is abnormal<br>• For MM subjects only: subjects who have relapsed after or are refractory or intolerant to all established MM therapies that are both known to provide clinical benefit, and locally available. At least 3 prior lines of therapy must have been administered, including 1 or more immunomodulatory agents (e.g., lenalidomide or pomalidomide), 1 or more proteasome inhibitors (e.g., bortezomib or carfilzomib), and 1 or more anti-CD38 monoclonal antibodies (e.g., daratumumab).<br>• Documented diagnosis of AML (Parts C and D only; subjects with APL are excluded throughout the study).<br>• AML subjects only: Total white blood cell count < 25,000/mm^3 (Note: hydroxyurea is permitted to meet this criterion).<br>• For AML subjects only: failure to respond to, and/or relapse or progression after, at least 1 prior line of therapy, including all available standard therapies (per assessment of local investigator). Where applicable, this includes prior exposure to approved inhibitors of FLT3, IDH1, and IDH2.<br>• Eastern Cooperative Oncology Group performance status of 0, 1, or 2.<br>• For subjects with AML, the following hematologic parameter requirements are not applicable. For subjects with MM, subjects must have adequate hematologic function, defined as follows:<br>• Absolute neutrophil count ≥ 1000/mm^3;<br>• Platelet count ≥ 75,000/mm^3;<br>• Hemoglobin ≥ 8 g/dL;<br>• Serum alanine aminotransferase (ALT) ≤ 2.5 × upper limit of normal (ULN);<br>• Serum aspartate aminotransferase (AST) ≤ 2.5 × ULN;<br>• Total bilirubin ≤ 1.5 × ULN (subject with documented Gilbert's syndrome may be allowed total bilirubin >1.5 × ULN);<br>• Amylase ≤ 2 × ULN;<br>• Lipase ≤ 2 × ULN;<br>• Creatinine clearance ≥ 60 mL/min, measured by 24-hour urine collection or calculated using the Cockcroft-Gault formula.

CONFIDENTIAL INFORMATION
No use or disclosure outside AbbVie is permitted without prior written authorization from AbbVie.

	• Echocardiogram with ejection fraction ≥ 50% and no other clinically significant finding that would increase the subject's susceptibility to cardiac toxicity<br>• No prior exposure to any targeted MCL-1 inhibitor.<br>• No antineoplastic therapy (including any cytotoxic, targeted, and/or investigational therapy; but not including corticosteroids), within 28 days or 5 half-lives, whichever is shorter, prior to the first dose of study drug and through the last dose of study drug(s).<br>• No autologous stem cell transplant within 90 days prior to start of ABBV-467.<br>• No allogeneic stem cell transplant within 180 days prior to start of ABBV-467.<br>• No history of acute or chronic pancreatitis.<br>• No significant unresolved liver disease.<br>• No known history of hepatitis B or HIV infection
**Study Drug and Duration of Treatment:**	ABBV-467 will be administered as an IV infusion once weekly for each 28-day cycle.<br>Subjects will continue ABBV-467 therapy until disease progression, withdrawal of consent, or unacceptable toxicity. No subject will receive ABBV-467 for longer than 24 months within the context of this study.
**Date of Protocol Synopsis:**	29 April 2020

2 INTRODUCTION

2.1 Background and Rationale

Why Is This Study Being Conducted

Although remarkable advances in multiple myeloma (MM) have emerged with the introduction of several new, more effective, and less toxic therapies, it remains an incurable disease. Treatment paradigms for MM include immunomodulatory agents, proteasome inhibitors, monoclonal antibodies, and corticosteroids, often in 2- or 3-drug combinations. However, the majority of patients will eventually relapse after, or become refractory to, all available therapies, in which case prognosis is poor. There remains a clear unmet need to identify agents with novel mechanisms of action for use alone and in combination with existing standards of care, to improve outcomes for patients with MM.

Acute myeloid leukemia (AML) is a heterogenous disease characterized by high unmet need.[1] Current treatment strategies are still largely based on combination chemotherapy and stem cell transplantation, but the toxicities associated with this approach limits its utility in elderly and unfit patients, and outcomes for all patients with relapsed/refractory (R/R) AML are poor.[2] As therapeutic progress in AML has been modest, opportunities for novel agents offering improved efficacy, reduced toxicity, or both, remain.

Myeloid cell leukemia-1 (MCL-1), a member of antiapoptotic B-cell lymphoma-2 (BCL-2) family proteins, is a key regulator of cell survival and homeostasis.[3] Frequent overexpression of MCL-1 has been observed in multiple cancer types where it acts as a resistance factor to various chemotherapeutic and targeted agents. Therapeutic targeting of MCL-1, alone and in combination with other standard-of-care agents, enhances apoptotic death in preclinical MM and AML models, and offers a novel approach to treating these hematologic malignancies with progressive morbidity and eventual mortality.

ABBV-467 is a small molecule that potently and selectively binds and inhibits MCL-1. The first-in-human study will evaluate the safety and tolerability of ABBV-467 in subjects with either R/R MM or R/R AML. Based on the monotherapy data from this study, combination therapy with additional compounds may be explored in the future.

2.2 Benefits and Risks to Subjects

ABBV-467 is a high-affinity MCL-1 inhibitor that exhibits robust preclinical efficacy in MM and AML models. The pharmacology, toxicology, and pharmacokinetic (PK) profiles of ABBV-467 have been adequately characterized to support an acceptable rationale and risk/benefit profile for treating adult subjects with MM or AML with ABBV-467 within the context of a clinical trial.

For further details, please see findings from completed studies, including safety data in the current ABBV-467 Investigator's Brochure.[4]

CONFIDENTIAL INFORMATION
No use or disclosure outside AbbVie is permitted without prior written authorization from AbbVie.

3 OBJECTIVES AND ENDPOINTS

Primary

The primary objectives of this study are:

- To characterize the safety and toxicity profiles of ABBV-467 in subjects with:
  - R/R MM.
  - R/R AML.
- To determine the recommended Phase 2 dose (RP2D) of ABBV-467 in subjects with:
  - R/R MM.
  - R/R AML.
- To determine the PK of ABBV-467.

Secondary

The secondary objective of this study is:

- To preliminarily evaluate the efficacy of ABBV-467 in R/R MM and R/R AML.

3.1 Primary Endpoints

Safety evaluations include, but are not limited to, adverse event (AE) monitoring, physical examinations, vital sign measurements, electrocardiogram (ECG), cardiac enzyme monitoring, and clinical laboratory testing (hematology and chemistry) as a measure of safety and tolerability for the entire study duration.

Pharmacokinetic parameters to be determined for ABBV-467 will include maximum observed plasma concentration (C_{max}), terminal elimination half-life ($t_{1/2}$), area under the plasma concentration-time curve from time 0 to time of last measurable concentration (AUC_t), area under the plasma concentration-time curve (AUC) from time 0 to infinity ($AUC_{0-\infty}$), and clearance (CL) using noncompartmental methods.

3.2 Secondary Endpoints

Multiple Myeloma

For MM, efficacy will be evaluated per adapted International Myeloma Working Group (IMWG)[5] criteria:

- Overall response rate (ORR), defined as partial response (PR) + very good partial response (VGPR) + complete remission (CR) + stringent complete response (sCR).
- Clinical benefit rate (CBR), defined as minimal response (MR) + PR + VGPR + CR + sCR.

Duration of response (DOR), defined as the time between date of first response and the first occurrence of progression or death from any cause, whichever occurs first.

No use or disclosure outside AbbVie is permitted without prior written authorization from AbbVie.

Acute Myeloid Leukemia

For AML, efficacy will evaluated per adapted International Working Group (IWG)[6] and European Leukemia Net (ELN)[7] criteria:

- Composite complete remission (CRc), comprised of CR + CR with incomplete blood count recovery (CRi).
- CR + CR with partial hematologic recovery (CRh).
- ORR, comprised of CRc + PR.
- DOR, as defined above.

3.3 Additional Efficacy Endpoints

Overall survival will be assessed as an exploratory efficacy endpoint of the study.

3.4 Biomarker Research Endpoints

Biospecimens (whole blood, plasma, bone marrow aspirate, and/or bone marrow core biopsies) will be collected at specified time points (Appendix D) throughout the study to evaluate known and/or novel disease-related or drug-related biomarkers in circulation or at tissue sites. The types of biomarkers to be analyzed may include, but are not limited to, nucleic acids, proteins, lipids, and/or metabolites, either free or in association with specific cell types.

Types of biomarker analyses may include, but are not limited to, determination of MCL-1, BCL-2, and BCL-X_L expression or functional dependence, depth of responses as measured by MRD techniques, and assessment of mutation status in MM and AML. These assessments may be explored in the context of MM, AML, or related conditions and/or ABBV-467 or drugs of similar classes. This research may be exploratory in nature and the results may not be included with the clinical study report. Biomarker samples will be collected and analyzed from all subjects, unless precluded by local regulations or restrictions.

Further details regarding the biomarker research collection time points are located in Section 3.8 of the Operations Manual (Appendix J).

4 INVESTIGATIONAL PLAN

4.1 Overall Study Design and Plan

This is a Phase 1, first-in-human, open-label, dose escalation and dose expansion study evaluating ABBV-467 in subjects with R/R MM and R/R AML; the schematic of the study is shown in Figure 1. Further details regarding study procedures are located in the Operations Manual. See Section 5 for information regarding eligibility criteria. Dosing will be weekly, on a 28-day cycle, though alternative

dosing frequencies may be studied if supported by the totality of safety, efficacy, PK, and/or pharmacodynamic (PD) data.

Subjects will continue ABBV-467 therapy until disease progression, unacceptable toxicity, or withdrawal of consent. No subject will receive ABBV-467 for longer than 24 months within the context of this study.

Figure 1. Study Diagram

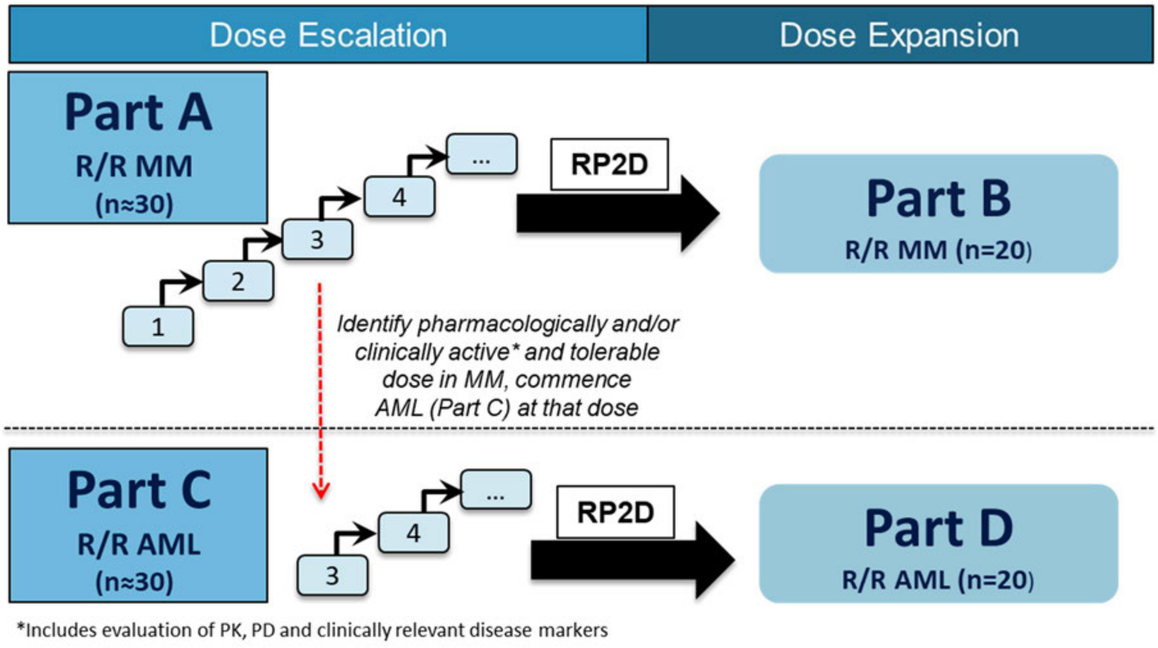

AML = acute myeloid leukemia; MM = multiple myeloma; PD = pharmacodynamic; PK = pharmacokinetic; RP2D = recommended Phase 2 dose; R/R = relapsed/refractory

Note: Part C will be initiated after a pharmacologically and/or clinically active and tolerable dose is determined in Part A. The expansion component will target enrollment of 20 response-evaluable subjects (with a maximum of 24 subjects enrolled).

Dose Escalation Guidelines

The goal of the dose escalation is to find the RP2D. The dose-escalation stage allows characterization of safety, tolerability, efficacy, PK, and PD data for each dose level while respecting the de-escalation and dose-elimination boundaries specified by the Bayesian optimal interval (BOIN) design.[8] To maintain subject safety, all dose escalation parts will be guided by BOIN design based on the cumulative number of subjects who experience a dose-limiting toxicity (DLT) in a given ABBV-467 dose level. De-escalation and dose elimination will occur when instructed by the BOIN rules set forth in Table 1. However, if the BOIN rules recommend dose escalation or dose de-escalation, the escalation or de-escalation decision will be made in consideration of all available information. No new subjects would be added to the dose escalation stage after a maximum of 12 subjects enrolled in a single dose level.

Dose escalation decisions require a minimum of 2 DLT-evaluable subjects in dose levels 1 and 2, and a minimum of 3 DLT-evaluable subjects for all subsequent dose levels. For this study, the DLT observation period is defined as the first treatment cycle (Cycle 1, 28 days). Adverse events occurring after the DLT

CONFIDENTIAL INFORMATION
No use or disclosure outside AbbVie is permitted without prior written authorization from AbbVie.

observation period will also be reviewed and may be taken into consideration for ensuring dose escalation decisions. At least 6 subjects will be treated during dose escalation at what is ultimately declared the disease specific RP2D prior to commencing dose expansion at the same dose.

The target toxicity rate is 0.25. A true toxicity rate of 0.6 × the target rate or lower is deemed potentially subtherapeutic such that dose escalation is indicated and a true toxicity rate of 1.4 × the target rate or higher is deemed overly toxic such that de-escalation is indicated. For non-informative priors, the prespecified optimal escalation and de-escalation boundaries for the observed toxicity rate are 0.197 and 0.298, respectively. Hence, if the observed toxicity rate at the current dose level is ≤ 0.197, then the dose is escalated for the next cohort. If the observed toxicity rate is ≥ 0.298, then the dose is de-escalated for the next cohort. Furthermore, if there is a higher than 95% posterior probability that the true toxicity rate associated with the current dose level is above the target toxicity rate, then that dose level and higher dose levels will be eliminated. These boundaries produce the following decision table based on the cumulative number of subjects who experience a DLT at current dose level (Table 1).

Table 1. Dose De-escalation/Escalation Decision Rules

	# of DLT-Evaluable Subjects at Current Dose Level										
**Action**	**2**	**3**	**4**	**5**	**6**	**7**	**8**	**9**	**10**	**11**	**12**
Escalate if # of subjects with DLT ≤	0	0	0	0	1	1	1	1	1	2	2
Stay at current dose level if # of subjects with DLT =	-	-	1	1	-	2	2	2	2	3	3
De-escalate if #of subjects with DLT ≥	1	1	2	2	2	3	3	3	3	4	4
Eliminate if # of subjects with DLT ≥	NA	3	3	3	4	4	4	5	5	6	6

DLT = dose-limiting toxicity; NA = not applicable

Dose escalation increments are based on a modified Fibonacci sequence that yield proportionally smaller increases at higher doses,[9] as shown in Table 2. At the joint discretion of the investigators and the sponsor additional interval dose levels may also be explored but will continue to follow identical de-escalation/escalation decision rules. If the BOIN rules recommend dose de-escalation, the next dose evaluated could be an intermediate dose not exceeding the current dose. If the BOIN rules recommend dose escalation, the next dose evaluated could be an intermediate dose not exceeding the next dose level in the modified Fibonacci sequence, chosen in consideration of all available information.

Table 2. Proposed Dose Levels

**Dose Level**	1	2	3	[redacted]
**Dose (mg/kg)**	0.16	0.32	0.53	

Throughout all parts of the study, ABBV-467 will be administered with an abbreviated dose ramp-up, in which 50% of the target dose will be administered on Cycle 1 Day 1 (C1D1), and full target dose thereafter (weekly until disease progression, unacceptable toxicity, or withdrawal of consent).

For each dose level, the second subject will not be dosed until at least 48 hours have elapsed since the initial dose for the first subject at that dose level.

Part A and Part C

Subjects with R/R MM (Part A) will receive escalating doses of ABBV-467 as described above. Once a pharmacologically and/or clinically active and tolerable dose is identified based on review of totality of safety, efficacy, PK and PD data, this dose will be used to commence dose escalation in R/R AML (Part C), where provisional dose de-escalation will also be permitted.

In order to be considered DLT evaluable, a subject must either:

1. receive each of planned doses *and* at least 80% of the total planned dose of ABBV-467 for Cycle 1; or
2. experience a DLT after receipt of at least one dose of ABBV-467 during the DLT evaluation period.

Selection of doses used in the study, including the optimal biological dose (OBD) and RP2D, can be found in Section 4.2.

Expansion Components

Dose expansion for subjects with R/R MM (Part B) or R/R AML (Part D) will commence after the RP2D is identified and the initial safety and efficacy data of ABBV-467 are evaluated as favorable by the Sponsor with input from the investigators. A cumulative review of safety, tolerability, efficacy, PK, and PD data collected during dose escalation will occur prior to initiating dose expansion.

Subjects with R/R MM (Part B) and R/R AML (Part D) will receive the ABBV-467 at the RP2D determined in Parts A and C, respectively. Expansion may occur at multiple dose levels and/or schedules. The expansion component will target enrollment of 20 response-evaluable subjects (with a maximum of 24 subjects enrolled regardless of response evaluability) per cohort to further evaluate the safety profile, and preliminarily evaluate efficacy, of ABBV-467 at RP2D.

At the joint discretion of the sponsor and the investigators, subjects may be prospectively selected for Parts B and D based on any of the following:

1. Only those with prior exposure to a BCL-2 inhibitor.
2. Only those naïve to prior BCL-2 inhibitor.
3. Irrespectively of prior BCL-2 inhibitor exposure.

4.2 Discussion of Study Design

Choice of Control Group

Not applicable.

No use or disclosure outside AbbVie is permitted without prior written authorization from AbbVie.

Appropriateness of Measurements

Standard PK, statistical, clinical, and laboratory procedures will be utilized in this study. All efficacy measurements in this study are standard for assessing disease activity in subjects with MM and AML. All clinical and laboratory procedures in this study are standard and generally accepted.

Suitability of Subject Population

Subjects with MM whose disease has relapsed after, or was refractory to, treatment with standard therapies (immunomodulatory agents, proteasome inhibitors, anti-CD38 monoclonal antibodies, alone or in combination) have limited options and poor prognoses. In addition, subjects with AML that have relapsed after 1 or more lines of treatment, including all locally approved therapies, face a dire prognosis, with survival measured in months or even weeks. These subjects are broadly considered ideal candidates for novel treatment approaches, and are therefore, the subject population chosen for this study.

Selection of Doses in the Study

The starting dose of ABBV-467 is based on the International Conference on Harmonisation of Technical Requirements for Registration of Pharmaceuticals for Human Use (ICH) S9 guidance (ICH 2010). The dog is considered the appropriate species for calculating human starting dose. [redacted]

Full pharmacology and toxicology data for ABBV-467 are provided in the ABBV-467 Investigator's Brochure.[4]

5 STUDY ACTIVITIES

5.1 Eligibility Criteria

Subjects must meet all of the following criteria in order to be included in the study. Anything other than a positive response to the questions below will result in exclusion from study participation.

Consent

- 1. Subjects or their legally authorized representative must voluntarily **sign and date an informed consent**, approved by an independent ethics committee (IEC)/institutional review board (IRB), prior to the initiation of any screening or study-specific procedures.

Demographic and Laboratory Assessments

- 2. At least 18 years old.
- 3. Eastern Cooperative Oncology Group (ECOG) performance status 0, 1, or 2.
- 4. **Laboratory values** meeting the following criteria during the screening period prior to first dose of study drug:
  - For subjects with AML, the following hematologic parameter requirements are not applicable. For subjects with MM, subjects must have adequate hematologic function, defined as follows:
    - Absolute neutrophil count ≥ 1000/mm^3;
    - Platelet count ≥ 75,000/mm^3;
    - Hemoglobin ≥ 8 g/dL;
  - Serum alanine aminotransferase (ALT) ≤ 2.5 × upper limit of normal (ULN);
  - Serum aspartate aminotransferase (AST) ≤ 2.5 × ULN;
  - Total bilirubin ≤ 1.5 × ULN (subject with documented Gilbert's syndrome may have a total bilirubin >1.5 × ULN);
  - Amylase ≤ 2 × ULN;
  - Lipase ≤ 2 × ULN;
  - Creatinine clearance (CrCl) ≥ 60 mL/min, measured by 24-hour urine collection or calculated using the Cockcroft-Gault formula.
- 5. Echocardiogram with ejection fraction ≥ 50% and no other clinically significant finding that would increase the subject's susceptibility to cardiac toxicity.
- 6. No clinically relevant or significant ECG abnormalities, including ECG with QT interval corrected for heart rate (QTc) using Fridericia's formula (QTcF) > 450 msec (males) or > 470 msec (females).
- 7. Willingness and ability to comply with procedures required in this protocol.

No use or disclosure outside AbbVie is permitted without prior written authorization from AbbVie.

Disease Activity

- 8. Documented diagnosis of MM (Parts A and B only).
- 9. For MM subjects only: measurable disease, defined as at least 1 of the following:
  - Serum monoclonal protein ≥ 1 g/dL
  - Urine M-protein ≥ 200 mg/24 hours
  - Serum immunoglobulin free light chain (FLC) ≥ 10 mg/dL (100 mg/L), provided serum FLC ratio is abnormal
- 10. For MM subjects only: Subjects who have relapsed after or are refractory or intolerant to all established MM therapies that are both known to provide clinical benefit, and locally available. At least 3 prior lines of therapy must have been administered (Appendix E), including 1 or more immunomodulatory agents (e.g., lenalidomide or pomalidomide), 1 or more proteasome inhibitors (e.g., bortezomib or carfilzomib), and 1 or more anti-CD38 monoclonal antibodies (e.g., daratumumab).
- 11. Documented diagnosis of AML (Parts C and D only; subjects with APL are excluded throughout the study).
- 12. AML subjects only: Total white blood cell count < 25,000/mm^3 (Note: hydroxyurea is permitted to meet this criterion).
- 13. For AML subjects only: failure to respond to, and/or relapse or progression after, at least 1 prior line of therapy, including all available standard therapies (per assessment of local investigator). Where applicable, this could include prior exposure to approved inhibitors of FLT3, IDH1, and IDH2.
- 14. No autologous stem cell transplant within 90 days prior to start of ABBV-467.
- 15. No allogeneic stem cell transplant within 180 days prior to start of ABBV-467.
- 16. No clinically significant graft versus host disease requiring ongoing systemic therapy.

Subject History

- 17. No unresolved clinically significant non-hematologic toxicity of ≥ Grade 2 from prior anticancer therapy.
- 18. No history of acute or chronic pancreatitis.
- 19. No significant unresolved liver disease. Subjects with hepatitis C who have received definitive treatment and are considered cured will be eligible.
- 20. No known history of hepatitis B or HIV infection. Testing for hepatitis B and HIV are not required as part of Screening.
- 21. No major surgery within 4 weeks of study treatment.
- 22. No history of central nervous system involvement by underlying disease.
- 23. No history of plasma cell leukemia.

- 24. No history of clinically significant medical conditions or any other reason that the investigator determines would interfere with the subject's participation in this study or would make the subject an unsuitable candidate to receive study drug.
- 25. No history of other malignancies, with the following exceptions:
  - No known active disease present within 3 years prior to first dose of study treatment, and felt to be at low risk of recurrence by the treating investigator;
  - Adequately treated in situ carcinoma without evidence of disease;
  - Basal cell carcinoma of the skin or localized squamous cell carcinoma of the skin without evidence of disease;
- 26. No history of an allergic reaction or significant sensitivity to constituents of the study drug (and its excipients) and/or other products in the same class.

Contraception

- 27. For all females of child-bearing potential; a **negative serum pregnancy test** at the Screening Visit and a negative urine pregnancy test at baseline prior to the first dose of study drug.
- 28. Female subjects of childbearing potential must practice at least 1 protocol-specified **method of birth control**, that is effective from C1D1 through at least 185 days after the last dose of study drug (refer to Section 5.2).
- 29. Female must not be **pregnant, breastfeeding (even if breastfeeding is interrupted), or considering becoming pregnant** during the study or for approximately 185 days after the last dose of study drug.
- 30. **If male**, and subject is **sexually active with female partner(s) of childbearing potential**, he must agree, from C1D1 through 95 days after the last dose of study drug, to practice the protocol-specified contraception (refer to Section 5.2).
- 31. Male must not be considering **fathering a child or donating sperm** during the study or for approximately 95 days after the last dose of study drug.

Concomitant Medications

- 32. Subject has no prior exposure to any targeted MCL-1 inhibitor.
- 33. Subject must not have been treated with **antineoplastic therapy** (including cytotoxic, targeted, and/or investigational therapy; but not including corticosteroids), within 28 days or 5 half-lives, whichever is shorter, prior to first dose of study drug and through the last dose of study drug(s).
- 34. No anticipation of the use of prohibited medications during study participation (see Section 5.3 for additional prohibited medications or foods).
- 35. Subject must not have received any live vaccine within 2 weeks before the first dose of study drug or be expected to need a live vaccination during study participation including at least 4 weeks after the last dose of study drug.

CONFIDENTIAL INFORMATION
No use or disclosure outside AbbVie is permitted without prior written authorization from AbbVie.

abbvie

5.2 Contraception Recommendations

Contraception Requirements for Females

Subjects must follow the following contraceptive guidelines as specified:

- <u>Females, Non-Childbearing Potential</u>

  Females do not need to use birth control during or following study drug treatment if considered of non-childbearing potential due to meeting any of the following criteria:

  - Postmenopausal, age > 55 years with no menses for 12 or more months without an alternative medical cause.
  - Postmenopausal, age ≤ 55 years with no menses for 12 or more months without an alternative medical cause and a follicle-stimulating hormone level > 40 IU/L.
  - Permanently surgically sterile (bilateral oophorectomy, bilateral salpingectomy, or hysterectomy).

- <u>Females, of Childbearing Potential</u>

  Females of childbearing potential must avoid pregnancy while taking study drug(s) and for at least 185 days after the last dose of study drug. Females must commit to 1 of the following methods of birth control:

  - Combined (estrogen and progestogen containing) hormonal birth control (oral, intravaginal, transdermal, injectable) associated with inhibition of ovulation initiated at least 30 days prior to study Baseline Day 1, in combination with a barrier method(s) as drug-drug interactions with ABBV-467 with the hormonal contraception are unknown.
  - Progestogen-only hormonal birth control (oral, injectable, implantable) associated with inhibition of ovulation initiated at least 30 days prior to study Baseline Day 1, in combination with a barrier method(s) as drug-drug interactions with ABBV-467 with the hormonal contraception are unknown.
  - Bilateral tubal occlusion/ligation (can be via hysteroscopy, provided a hysterosalpingogram confirms success of the procedure).
  - Intrauterine device (IUD).
  - Intrauterine hormone-releasing system (IUS).
  - Vasectomized partner (provided the partner has received medical confirmation of the surgical success of the vasectomy and is the sole sexual partner of the trial subject).
  - Practice true abstinence, defined as: Refraining from heterosexual intercourse when this is in line with the preferred and usual lifestyle of the subject (periodic abstinence [e.g., calendar, ovulation, symptothermal, post-ovulation methods] and withdrawal are not acceptable).
  - If required per local practices, male or female condom with or without spermicide or cap, diaphragm or sponge with spermicide should be used in addition to 1 of the birth control methods listed above (excluding true abstinence).

Contraception recommendations related to use of concomitant therapies prescribed should be based on the local label.

Contraception Requirements for Males

Male subjects who are sexually active with a female partner of childbearing potential, must agree **to use condoms, even if the male subject has undergone a successful vasectomy**, from C1D1 through at least 95 days after the last dose of study drug:

- His female partner(s) must also use at least 1 of the following methods of birth control:
  - Combined (estrogen and progestogen containing) hormonal birth control (oral, intravaginal, transdermal, injectable) associated with inhibition of ovulation initiated at least 30 days prior to study Baseline Day 1.
  - Progestogen-only hormonal birth control (oral, injectable, implantable) associated with inhibition of ovulation initiated at least 1 month prior to study Baseline Day 1.
  - Bilateral tubal occlusion/ligation (can be via hysteroscopy, provided a hysterosalpingogram confirms success of the procedure).
  - IUD.
  - IUS.

5.3 Prohibited Medications and Therapy

No anticancer agents, radiation, anticancer medicinal/herbal remedies, investigational agents, anticancer corticosteroid therapy, or anticancer hormonal therapy may be taken while the subject is in the treatment phase of the study. However, during the dose escalation part of the study only, localized radiation therapy to a site of pre-existing and/or symptomatic disease may be permitted. Following approval from the sponsor, a subject may initiate or continue with protocol therapy without interruption during the course of palliative radiation therapy if the investigator believes that the risk of excessive bone marrow suppression or other toxicity is acceptable, and it is in the best interest of the subject.

Extramedullary sites of MM or AML identified at baseline that are surgically resected or in any radiated fields should be excluded from further response assessments.

Use of radiotherapy or surgical intervention must be recorded on the electronic case report form (eCRF).

Medications known to prolong the QT interval (Operations Manual Section 7.6) are prohibited.

5.4 Prior and Concomitant Therapy

Any medication or vaccine (including over the counter or prescription medicines, vitamins, and/or other supplements) that the subject is receiving at the time of enrollment or receives during the study must be recorded through the follow-up visit.

CONFIDENTIAL INFORMATION
No use or disclosure outside AbbVie is permitted without prior written authorization from AbbVie.

Best supportive care and treatment in accordance with standard practice (antiemetics, antibiotics, transfusions, oxygen therapy, nutritional support, palliative treatment for pain or cough, etc.) will be given as appropriate to each subject. Any questions regarding concomitant or prior therapy should be raised to the AbbVie emergency contact. Information regarding potential drug interactions with ABBV-467 can be located in the ABBV-467 Investigator's Brochure.

Medications that may be required during the first 15 days of Cycle 1 to reduce the risk of tumor lysis syndrome (TLS) include the following:

- Allopurinol or other appropriate TLS risk-reducing agent required through Day 15 of Cycle 1.
- Adequate fluids to ensure dehydration prevention (note: per institutional standards while using medical judgment; refer to Appendix G).

In addition, use of hydroxyurea is recommended as needed to maintain a white blood cell count of < 25,000/µL during Cycle 1 of the study.

MM subjects only: In dose escalation, hematopoietic growth factors and transfusion support should not be given during the DLT evaluation period, but are otherwise permitted at the investigator's discretion, in accordance with local or institutional guidelines and/or practice patterns.

The following medications are to be used with caution during the study (see Operations Manual Section 7.5 for examples of each):

- P-glycoprotein inhibitors.
- Breast cancer resistance protein inhibitors.
- OATP1B1/B3 inhibitors.
- OATP1B1/1B3 substrates.
- Strong CYP3A4 inhibitors.

Subjects who are receiving corticosteroids chronically (> 3 months) for non-oncological purposes may continue at a dose that does not exceed 5 mg per day of prednisone or equivalent.

5.5 Withdrawal of Subjects from Treatment and Discontinuation of Study

Discontinuation of Individual Subjects from Treatment

A subject may voluntarily withdraw or be withdrawn from study drug at any time for reasons including, but not limited to, the following:

- Clinically significant abnormal laboratory results or AEs, which rule out continuation of the study drug, as determined by the investigator or the AbbVie Therapeutic Area Medical Director (TA MD).
- An AE that results in a study drug hold of > 28 days.

- Subject experiences progressive disease per IMWG criteria (MM) or IWG and ELN criteria (AML).
- The investigator believes it is in the best interest of the subject.
- The subject requests withdrawal from study drug treatment.
- Eligibility criteria violation was noted after the subject started study drug and continuation of the study drug would place the subject at risk.
- Introduction of prohibited medications or dosages and continuation of the study drug would place the subject at risk.
- The subject becomes pregnant while on study drug.
- Subject is significantly noncompliant with study procedures, which would put the subject at risk for continued participation in the trial.
- Subject is lost to follow-up.
- Study is terminated by the sponsor.

To minimize missing data for efficacy and safety assessments, any subject who discontinues study drug treatment should return for the Final Visit, the 30-Day Safety Follow-Up Visit, and continue to be followed for progression (if discontinued for reasons other than progression) and survival-related endpoints, unless s/he has withdrawn informed consent. Subjects should be advised on the continued scientific importance of their data even if they discontinue treatment with study drug early.

Discontinuation of Individual Subjects from Study

A subject may voluntarily withdraw or be withdrawn from the study at any time for reasons including, but not limited to, the following:

- The subject requests withdrawal from the study.
- Lost to follow-up.
- Study terminated by the sponsor.
- Death.

Final Visit procedures as described in Appendix D should be performed when a subject discontinues from the study.

For subjects to be considered lost to follow-up, reasonable attempts must be made to obtain information on the subject's final status. At a minimum, 2 telephone calls must be made, and 1 certified letter must be sent and documented in the subject's source documentation.

AbbVie may terminate this study prematurely, either in its entirety or at any site for reasonable cause provided that written notice is submitted in advance of the intended termination. The investigator may also stop the study at his/her site if he/she has safety concerns. If AbbVie terminates the study for safety reasons, AbbVie will promptly notify the investigator.

5.6 Follow-Up for Subject Withdrawal from Study Treatment or from Study

For any subject who withdraws informed consent, the procedures outlined for the Final Visit should be completed at the subject's last visit. In addition, if subject is willing, a 30-day follow-up phone call after the last dose of study drug may be completed to ensure all treatment-emergent AE/serious adverse events (SAEs) have been resolved to a ≤ Grade 1 or baseline level or, in the opinion of the investigator, the event is unlikely to resolve.

All attempts must be made to determine the date of the last study drug dose and the primary reason for discontinuation of study drug or study participation. The information will be recorded on the appropriate eCRF page. However, these procedures should not interfere with the initiation of any new treatments or therapeutic modalities that the investigator feels are necessary to treat the subject's condition. Following discontinuation of study drug, the subject will be treated in accordance with the investigator's clinical judgment, irrespective of whether or not the subject decides to continue participation in the study.

In the event a subject withdraws consent from the clinical study, biomarker research will continue unless the subject explicitly requests analysis to be stopped. When AbbVie is informed that the subject has withdrawn and no longer wishes biomarker research to continue, samples will not be analyzed, no new biomarker analysis data will be collected for the withdrawn subject or added to the existing data or database(s). A subject may withdraw consent for optional biomarker research at any time and remain in the clinical study. Data generated from clinical study and/or optional biomarker research before subject withdrawal of consent, will remain part of the study results.

5.7 Study Drug

ABBV-467, manufactured by AbbVie, should be administered as an intravenous (IV) infusion via a central venous catheter (CVC) (e.g., peripherally inserted central catheter line, port line) once weekly (QW), for each 28-day cycle over at least a 30-minute period beginning on C1D1. For any particular dose level below 1.4 mg/kg, a peripheral IV may be used if/when CVC placement is unavailable or contraindicated (CVC use is mandatory for any dose 1.4 mg/kg or higher due to possibly increased risk of adverse local/skin reactions). Subjects receiving study drug via peripheral IV should be monitored closely for extravasation and/or local irritation (which, if observed, should be reported to Sponsor). Doses will be determined by dose-escalation decision rules; the starting dose is 0.16 mg/kg. ABBV-467 can be administered with or without food. The first dose of ABBV-467 on C1D1 will be administered at half the targeted dose for a given subject; subsequent doses will be administered at the full target dose. After completion of Cycle 1, ABBV-467 doses may be delayed for up to 1 day due to scheduling conflicts; delays longer than 1 day should result in the dose being skipped. If there is a less than 10% weight change from weight on C1D1, no dose adjustment is necessary.

During preparation of the dose for infusion and during infusion, the solution must be protected from light. As the phototoxic potential of ABBV-467 is unknown, subjects will be advised to limit sunlight exposure during participation in the trial.

CONFIDENTIAL INFORMATION
No use or disclosure outside AbbVie is permitted without prior written authorization from AbbVie.

Study drug will be administered at the site with appropriate medical support available to manage infusion reactions if they occur. The study site personnel will document compliance.

AbbVie will provide instructions for drug preparation. AbbVie will not supply drugs other than ABBV-467.

Study drug information is presented in Table 3.

Table 3. Study Drug Information

Investigational Product	Manufacturer	Mode of Administration	Dosage Form	Strength
ABBV-467	AbbVie	Intravenous	Concentrate for Solution for Infusion	[redacted]

Study drug will only be used for the conduct of this study.

Additional information on the investigational product is provided in the Operations Manual, Section 6.

5.8 Randomization/Drug Assignment

This is an open-label study; there is no randomization. As they are enrolled in the study, subjects will be assigned unique identification numbers. At the time of screening, assignment of unique subject numbers and dose assignments will be done via interactive response technology. For subjects who rescreen, the screening number assigned at the initial screening visit should be used.

5.9 Protocol Deviations

The investigator is responsible for complying with all protocol requirements, written instructions, and applicable laws regarding protocol deviations. Protocol deviations are prohibited except when necessary to eliminate an immediate hazard to study subjects. If a protocol deviation occurs (or is identified), the investigator is responsible for notifying IEC/IRB, regulatory authorities (as applicable), and AbbVie.

6 SAFETY CONSIDERATIONS

6.1 Complaints and Adverse Events

Complaints

A complaint is any written, electronic, or oral communication that alleges deficiencies related to the physical characteristics, identity, quality, purity, potency, durability, reliability, safety, effectiveness, or performance of a product/device. Complaints associated with any component of this investigational product must be reported to AbbVie.

CONFIDENTIAL INFORMATION
No use or disclosure outside AbbVie is permitted without prior written authorization from AbbVie.

Product Complaint

A product complaint is any complaint related to the biologic or drug component of the product or to the medical device component(s).

For a product this may include, but is not limited to, damaged/broken product or packaging, product appearance whose color/markings do not match the labeling, labeling discrepancies/inadequacies in the labeling/instructions (e.g., printing illegible), missing components/product, device not working properly, or packaging issues.

Product complaints concerning the investigational product and/or device must be reported to AbbVie within 24 hours of the study site's knowledge of the event. Product complaints occurring during the study will be followed up to a satisfactory conclusion.

Medical Complaints/Adverse Events and Serious Adverse Events

An AE is defined as any untoward medical occurrence in a subject or clinical investigation subject administered a pharmaceutical product and which does not necessarily have a causal relationship with this treatment. An AE can therefore be any unfavorable and unintended sign (including an abnormal laboratory finding), symptom, or disease temporally associated with the use of a medicinal (investigational) product, whether or not the event is considered causally related to the use of the product.

Such an event can result from use of the drug as stipulated in the protocol or labeling, as well as from "special situations," such as accidental or intentional overdose, medication error, occupational or accidental exposure, off-label use, drug abuse, drug misuse, all which must be reported whether associated with an AE or not. Any worsening of a pre-existing condition or illness is considered an AE. Worsening in severity of a reported AE should be reported as a new AE. Laboratory abnormalities and changes in vital signs are considered to be adverse events only if they result in discontinuation from the study, necessitate therapeutic medical intervention, meets protocol-specific criteria (see Section 6.2 regarding toxicity management), and/or if the investigator considers them to be AEs.

The investigators will monitor each subject for clinical and laboratory evidence of AEs on a routine basis throughout the study. All AEs will be followed to a satisfactory conclusion.

An elective surgery/procedure scheduled to occur during a study will not be considered an AE if the surgery/procedure is being performed for a pre-existing condition and the surgery/procedure has been pre planned prior to study entry. However, if the pre-existing condition deteriorates unexpectedly during the study (e.g., surgery performed earlier than planned), then the deterioration of the condition for which the elective surgery/procedure is being done will be considered an AE.

If any of the following AEs are reported, then the following supplemental report must be completed.

Adverse Event	Supplemental eCRF
**All AEs that meet DLT criteria, per protocol Section 6.3, Dose-Limiting Toxicity**	Dose-Limiting Toxicity eCRF
**Discontinuation or interruption of study drug due to a hepatic-related AE**<br>**A hepatic-related SAE**<br>**Clinically significant hepatic AEs**<br>**ALT or AST ≥ 3 × ULN along with total bilirubin > 2 × ULN**	Hepatic AE eCRF
**All AEs of infusion related reaction regardless of causality**	Infusion Related Reaction eCRF
**AEs of overdose, abuse, off-label use, misuse, medication error, occupational exposure**	Special Safety Situations eCRF
**Per protocol Section 6.2, Toxicity Management**	Tumor Lysis Syndrome eCRF

AE = adverse event; eCRF = electronic case report form; SAE = serious adverse event; ULN = upper limit of normal

If an AE, whether associated with study drug or not, meets any of the following criteria, it is to be reported to AbbVie clinical pharmacovigilance as a SAE within 24 hours of the site being made aware of the SAE (refer to Section 4.3 of the Operations Manual for reporting details and contact information):

**Death of Subject**	An event that results in the death of a subject.
**Life-Threatening**	An event that, in the opinion of the investigator, would have resulted in immediate fatality if medical intervention had not been taken. This does not include an event that would have been fatal if it had occurred in a more severe form.
**Hospitalization or Prolongation of Hospitalization**	An event that results in an admission to the hospital for any length of time or prolongs the subject's hospital stay. This does not include an emergency room visit or admission to an outpatient facility.
**Congenital Anomaly**	An anomaly detected at or after birth, or any anomaly that results in fetal loss.
**Persistent or Significant Disability/Incapacity**	An event that results in a condition that substantially interferes with the activities of daily living of a study subject. Disability is not intended to include experiences of relatively minor medical significance such as headache, nausea, vomiting, diarrhea, influenza, and accidental trauma (e.g., sprained ankle).

**Important Medical Event Requiring Medical or Surgical Intervention to Prevent Serious Outcome**	An important medical event that may not be immediately life-threatening or result in death or hospitalization, but based on medical judgment may jeopardize the subject and may require medical or surgical intervention to prevent any of the outcomes listed above (i.e., death of subject, life threatening, hospitalization, prolongation of hospitalization, congenital anomaly, or persistent or significant disability/incapacity). Additionally, any elective or spontaneous abortion or stillbirth is considered an important medical event. Examples of such events include allergic bronchospasm requiring intensive treatment in an emergency room or at home, blood dyscrasias or convulsions that do not result in inpatient hospitalization, or the development of drug dependency or drug abuse.

All AEs reported from the time of study drug administration until 30 days after discontinuation of study drug administration will be collected, whether solicited or spontaneously reported by the subject. In addition, study procedure-related serious and nonserious AEs will be collected from the time the subject signs the study-specific informed consent.

The following definitions will be used for Serious Adverse Reactions (SAR) and Suspected Unexpected Serious Adverse Reaction (SUSAR):

**SAR**	Defined as all noxious and unintended responses to an IMP related to any dose administered that result in death, are life-threatening, require inpatient hospitalization or prolongation of existing hospitalization, result in persistent or significant disability or incapacity, or are a congenital anomaly or birth defect.
**SUSAR**	A suspected SAR: refers to individual SAE case reports from clinical trials where a causal relationship between the SAE and the IMP was suspected by either the sponsor or the investigator, is not listed is the applicable Reference Safety Information, and meets one of the following serious criteria: results in death, is life-threatening, requires hospitalization or prolongation of an existing hospitalization, results in persistent or significant disability or incapacity, or is a congenital anomaly or birth defect. All individually reported SARs are considered suspected.

AbbVie will be responsible for Suspected Unexpected Serious Adverse Reactions (SUSAR) reporting for the Investigational Medicinal Product in accordance with global and local requirements.

Adverse events will be monitored throughout the study to identify any of special interest that may indicate a trend or risk to subjects.

Adverse Event Severity and Relationship to Study Drug

The investigators will rate the severity of each AE according to the National Cancer Institute (NCI) Common Terminology Criteria for Adverse Events (CTCAE), version 5.0. If a reported AE increases in

severity, the initial AE should be given an outcome date and a new AE must be reported to reflect the change in severity. The dates on the AEs cannot overlap. For all reported SAEs that increase in severity, the supplemental eCRFs also need to be updated to reflect any changes due to the increase in severity.

For AEs not captured by the NCI CTCAE, the following should be used:

**Grade 1**	The AE is transient and easily tolerated by the subject (mild).
**Grade 2**	The AE causes the subject discomfort and interrupts the subject's usual activities (moderate).
**Grade 3**	The AE causes considerable interference with the subject's usual activities and may be incapacitating (moderate to severe).
**Grade 4**	The AE is life-threatening and requires urgent intervention (severe).
**Grade 5**	The AE resulted in death of the subject (severe).

The investigator will use the following definitions to assess the relationship of the AE to the use of study drug:

**Reasonable Possibility**	After consideration of factors including timing of the event, biologic plausibility, clinical judgment, and potential alternative causes, there is sufficient evidence (information) to suggest a causal relationship.
**No Reasonable Possibility**	After consideration of factors including timing of the event, biologic plausibility, clinical judgment, and potential alternative causes, there is insufficient evidence (information) to suggest a causal relationship.

Pregnancy

While not an AE, pregnancy in a study subject must be reported to AbbVie within 24 hours after the site becomes aware of the pregnancy. Subjects who become pregnant during the study must be discontinued (Section 5.5). If a pregnancy occurs in a study subject or in the partner of a study subject, information regarding the pregnancy and the outcome will be collected.

In the event of pregnancy occurring in a subject's partner during the study, written informed consent from the partner must be obtained prior to collection of any such information. AbbVie will provide a separate consent form for this purpose. Pregnancy in a subject's partners will be collected from the date of the first dose through 90 days following the last dose of study drug.

The pregnancy outcome of an elective or spontaneous abortion, stillbirth or congenital anomaly is considered a SAE and must be reported to AbbVie within 24 hours after the site becomes aware of the event.

6.2 Toxicity Management

ABBV-467 Dosing Modifications for Toxicity

After Cycle 1, doses of ABBV-467 can be delayed up to 1 day due to toxicity; delays any longer than 1 day should result in the dose being skipped. If the Day 8 dose is skipped or delayed, the subject should resume dosing on Day 15 (provided that any toxicities requiring dose delay or interruption have resolved), and so on. If the Day 22 dose is skipped or delayed, the subject should complete the 28-day cycle on schedule and resume dosing at the start of the next cycle (provided that any toxicities requiring dose delay or interruption have resolved). If the ABBV-467 Day 1 dose of any cycle is delayed, then the start of the cycle should be delayed until the next dose of ABBV-467 is administered.

For any subject who experiences Grade 3/4 toxicity which is attributed to ABBV-467, the dose will be held until the toxicity resolves to Grade 1 or lower, or to baseline if the toxicity was > Grade 1 at the time of study entry. Upon toxicity resolution, the subject may restart study drug at 75% of that subject's starting dose (i.e., a 25% dose reduction). Following similar guidelines, subsequent dose reductions to 50% of the starting dose, and then a 25% of the starting dose, may be implemented. Dose reductions below 25% of the subject's starting dose are not allowed.

In addition, subjects with AML who experience CR or PR with hematologic recovery after receiving ABBV-467 and then experience Grade > 3 hematologic AE should undergo a bone marrow evaluation to determine if cytopenias are due to drug toxicity or recurrent disease.

Study drug interruptions for events that are considered not related to the study drug (e.g., underlying cancer, planned surgical procedures or acute viral illnesses), should not necessitate a dose reduction. The timing of dose resumption should be at the discretion of the Investigator.

Subjects experiencing a delay longer than 28 days due to an AE(s) related to study treatment should be permanently discontinued from study treatment. Subjects experiencing a delay longer than 28 days unrelated to study treatment must be discussed with the AbbVie TA MD or designee.

AML-Specific Dose Modifications for Drug-Related Hematologic Toxicities

If a subject with AML experiences hematologic toxicity during the study, the dose of ABBV-467 should be modified as indicated in Table 4.

Table 4. AML-Specific ABBV-467 Dose Modifications for Drug-Related Hematologic Toxicities

Treatment Cycle	Efficacy Assessments	Hematology Results	Action	Modifications
After Cycle 1[a]	CRi<br>MLFS	Incomplete count recovery	Delay upcoming cycle	ABBV-467 should be interrupted to allow for ANC recovery from Day 29 until ANC ≥ 500/µL or up to 14 days (by Day 42), whichever is earlier
After Cycle 2	CRi<br>MLFS	New Grade 4 neutropenia lasting for more than 1 week (unless due to the underlying disease)	Delay upcoming cycle	ABBV-467 dosing should be interrupted once cycle is completed until ANC is ≥ 500/µL (unless medically necessary to interrupt within cycle)
After Cycle 3	CR<br>CRi<br>MLFS	Subjects requiring interruption or delay of study drug administration for cytopenias (neutropenia/thrombocytopenia)	Reduce per-cycle ABBV-467 duration	ABBV-467 should be administered Days 1, 8, and 15 (but not Day 22) of each subsequent 28-day cycle

AML = acute myeloid leukemia; ANC = absolute neutrophil count; CR = complete remission; CRi = complete remission with incomplete blood count recovery; MLFS = morphologic leukemia-free state; PLT = platelet

a. Resistant disease or partial remission: there is no delay of next cycle. Once these subjects achieve CRi/MLFS, follow steps above in the sequence listed. CRi (ANC < 1,000/µL or PLT < 100,000/µL); MLFS (ANC < 1,000/µL & PLT < 100,000/µL).

Tumor Lysis Syndrome

Subjects with MM with high tumor burden (e.g., high bone marrow plasma cell infiltration, plasma cell leukemia, or bulky plasmacytomas), rapidly increasing M-protein or light chains or high proliferative activity, plasmablastic morphology, or compromised renal function (CrCl < 50 mL/minute) may be at higher risk of developing TLS.[10] There is a potential risk for TLS in subjects with AML, especially in those with elevated leukocyte count, circulating blasts, elevated pretreatment lactate dehydrogenase levels, renal dysfunction, and dehydration.[11,12] To mitigate the risk for TLS all subjects enrolled into the study will need TLS prophylaxis and monitoring. For subjects at higher risk for TLS, additional mitigation measures with more intensive laboratory monitoring and intervention should be implemented. Prophylactic reductions of potassium, inorganic phosphorus or uric acid above normal range are recommended prior to beginning study treatment and to be continued based on the ongoing risk of TLS. Please reference Appendix F for additional information on the evidence of TLS and Appendix G for initial management of electrolyte abnormalities and prevention of TLS.

During dose escalation, each subject should be hospitalized the night prior to C1D1 for hydration, treatment with uric acid-reducing agents, and monitoring. Tumor lysis syndrome laboratories will be obtained at 3 different time points: immediately prior to the first dose, 6 to 8 hours after the first dose, and approximately 24 hours after the first dose (on C1D2).

CONFIDENTIAL INFORMATION
No use or disclosure outside AbbVie is permitted without prior written authorization from AbbVie.

The subject may be discharged once the laboratory results on C1D2 are reviewed (approximately 24 hours after first dose) and confirm the absence of TLS. If discharged since first dose, subjects will be re-hospitalized the night prior to C1D8 for hydration, treatment with uric acid-reducing agents, and monitoring.

Tumor lysis syndrome prophylaxis and management for the second dose of ABBV-467 will be managed in the same manner as the first dose described above. Subjects who experience clinical or laboratory TLS, or other significant toxicity following the first or second dose of ABBV-467 should discuss the safety monitoring plan for subsequent doses of ABBV-467 with the AbbVie TA MD.

The Sponsor will review the totality of clinical and laboratory data on an ongoing basis and may provide additional recommendations regarding the need for subject monitoring, including either removing the need for, or extending required duration of, any hospitalization.

Below are the minimum requirements for TLS prophylaxis and management for all subjects enrolled into the study. All other prophylaxis and monitoring procedures for TLS will be implemented as per regional guidelines/institutional standards:

- All subjects must receive uric acid reducing agent, adequate oral and IV hydration as tolerated while monitoring the fluid status of the subject prior to and during the first cycle of treatment. The uric acid reducing agent, type of fluids and the rate of infusion will be determined by the investigator based on regional standards or institutional guidelines.
- TLS chemistry tests to be drawn (calcium, inorganic phosphorus, potassium, uric acid, and creatinine) on the first day of dosing and each day of a new dose at 0 (within 4 hours prior to dosing) and 6 – 8 hours post dose.
- Additional laboratory assessments may be performed, per investigator discretion, post-dose and up to 48 hours, if clinically indicated.

Abnormal chemistry tests should be corrected promptly. If a subject meets criteria for clinically significant laboratory or clinical TLS (Appendix F), institutional guidelines or recommendations in Appendix G should be followed, no additional doses should be administered until resolution.[13] For continued dosing, monitor for evidence of TLS during study treatment, and manage abnormalities of serum creatinine, and electrolytes promptly.

Hepatic Laboratory Abnormalities

Subjects should be monitored closely for hepatic-related laboratory abnormalities and AEs. In instances of new observations of hepatic laboratory abnormalities (i.e., ALT and/or AST > 1- < 3 × ULN and/or total bilirubin < 2 × ULN), clinical chemistries should be re-tested within 48 to 72 hours.

An algorithm for monitoring and management of drug-induced liver injury in subjects with normal or elevated baseline ALT and/or AST (defined as the average of 2 screening measurements) is available in Appendix H and should be used for study drug-related toxicity management. Guidelines should be followed for all subjects regardless of baseline liver function laboratory values. Hy's law is defined as ALT/AST and bilirubin in the normal reference range prior to study drug dosing, with subsequent development of ALT or AST ≥ 3 × ULN along with total bilirubin > 2 × ULN, without findings of cholestasis

(elevated serum ALP) and with no alternative explanation.[14] Any suspected cases of Hy's Law will require clinical review and confirmation.

Any increase in ALT and/or AST that is ≥ 3 × ULN but < 5 × ULN, the dose will be held until the toxicity resolves to Grade 1 or lower, or to baseline if the toxicity was > Grade 1 at the time of study entry. Upon toxicity resolution, the subject may restart study drug at a dose that is at least 33% lower than the previous dose. For ALT and/or AST increase ≥ 5 × ULN study drug may be restarted only if another etiology is identified and liver enzymes return to baseline.

In the event of hepatic abnormalities, documentation which captures relevant clinical signs and symptoms should be completed by the site in the source records and corresponding eCRF(s). In addition, for hepatic abnormalities, this should include imaging as clinically indicated (e.g., abdominal ultrasound, endoscopic retrograde cholangiopancreatography) to exclude alternative etiologies of liver injury (with full reports made available).

Pancreatic Enzyme Abnormalities and/or Pancreatitis

Any subject with pancreas enzyme (amylase and/or lipase) elevations, and/or clinical evidence of pancreatitis, should be promptly evaluated by the treating investigator. Treatment-emergent elevations in excess of 2 - 3 × ULN should trigger urgent evaluation, particularly when accompanied by other clinical signs and/or symptoms of pancreatitis.[15]

The following are guidelines for dose reduction, delay, and discontinuation of ABBV-467 in the setting of pancreatic abnormalities:

- ***Elevations in amylase/lipase:*** If isolated elevation of enzymes without signs or symptoms of pancreatitis, perform evaluation of pancreatitis by clinical assessment, abdominal computed tomography with contrast, and/or MRCP, consider other causes for elevated amylase/lipase. Additional grade-specific recommendations include:
    - Grade 3 increase in amylase and/or lipase (>2.0 - 5.0 × ULN with signs or symptoms of pancreatitis; > 5.0 × ULN regardless of signs/symptoms): ABBV-467 should be immediately interrupted, and the sponsor notified. At joint discretion of the investigator and sponsor, the subject may resume treatment, but only after the toxicity resolves to Grade 1 or lower, or to baseline if the toxicity was > Grade 1 at the time of study entry. In such instances, ABBV-467 should be resumed at a dose that is at least 33% lower than the previous dose.
    - Grade 4 increase in amylase and/or lipase (> 5.0 × ULN with signs and/or symptoms of pancreatitis): ABBV-467 should be immediately and permanently discontinued, and the sponsor notified.
- ***Pancreatitis:*** For any instance of pancreatitis (regardless of grade), ABBV-467 should be immediately held, and the sponsor notified:
    - Grade 2 (enzyme and/or radiographic findings in absence of either clear signs/symptoms of pancreatitis or need for intervention): At joint discretion of the investigator and sponsor, the subject may resume treatment, but only after the toxicity resolves to Grade 1 or lower. In such instances, ABBV-467 should be resumed at a dose that is at least 33% lower than the previous dose.

- Grade 3 - 4 (signs and/or symptoms of pancreatitis with medical intervention required): ABBV-467 should be immediately and permanently discontinued, and the sponsor notified.

Pancreatitis will be graded based on the criteria in the table below, adapted from CTCAE v5.0.

MedDRA SOC	CTCAE Term	Grade 1	Grade 2	Grade 3	Grade 4
Gastrointestinal disorders	Pancreatitis	-	Enzyme elevation; radiologic findings only	Severe pain; vomiting; medical intervention indicated (e.g., analgesia, nutritional support)	Life-threatening consequences; urgent intervention indicated

CTCAE = Common Terminology Criteria for Adverse Events; MedDRA = Medical Dictionary for Regulatory Activities; SOC = system organ class

In addition to the above, all local standards of care should be followed with respect to diagnostic and therapeutic interventions, including bowel rest, imaging, endoscopy, and/or gastroenterology consult.

Cardiovascular Toxicity

Due to MCL-1 expression on cardiac myocytes, there is a possibility that ABBV-467 may lead to cardiovascular toxicity including vital sign abnormalities (e.g., blood pressure, heart rate), chest pain, ECG abnormalities, and/or laboratory findings consistent with cardiac AEs.

Monitoring for cardiac toxicity will be performed as specified in the Protocol, Appendix D, and the Operations Manual, and additional assessments may be performed as clinically indicated. If any such parameters (ECG, echocardiography, laboratory parameters, and/or clinical signs) suggest occurrence of cardiotoxicity, then a cardiology consult should be initiated and the TA MD should be informed and consulted. If the AE appears valid and there is no alternative cause, then ABBV-467 should be promptly interrupted, and the TA MD consulted prior to any resumption.

Infusion Reactions and/or Allergic Reactions

Although the risk of infusion and/or allergic reactions with ABBV-467 is felt to be low, all subjects should be monitored for treatment-related AEs during all infusions, including measurement of vital signs every 30 minutes as specified in Operations Manual Section 3.11. For the initial 2 ABBV-467 infusions, subjects should remain at the site for monitoring for at least 2 hours following the completion of the 30 – 60-minute infusion (3 hours total). For subsequent infusions, post infusion monitoring is not required; however, vital signs should still be taken prior to start of infusion and every 30 minutes thereafter while the subject remains in the infusion area. Longer observation periods and more frequent vital sign evaluations are warranted in subjects who experience infusion reactions. ABBV-467 will be discontinued in the setting of Grade 4 infusion-related reactions.

If a Grade 2, Grade 3, or Grade 4 allergic reaction or infusion reaction is observed, ALL of the following measures are required:

- Immediate interruption of ABBV-467 treatment and discussion with the AbbVie TA MD before receiving any further treatment with ABBV-467.

- Appropriate medical therapy including epinephrine, corticosteroids, IV antihistamines, bronchodilators, and oxygen. Subjects should be carefully observed until the complete resolution of all signs and symptoms.
- Blood draw approximately 2 hours after the first sign of a reaction for the following laboratory tests: C3a, C5 complement functional assay, Immunoglobulin E level, and serum tryptase level.
- Collect unscheduled ABBV-467 PK samples.

6.3 Dose-Limiting Toxicity

Dose-limiting toxicity for dose escalation purposes will be determined on events that occur during the first 28-day cycle.

Any AE of Grade 3 or higher will be considered a DLT, unless toxicity can solely be attributed to the underlying disease, with the following clarifications. Adverse events of any grade resulting in discontinuation of study drug will be considered a DLT. Missing any dose as a result of toxicity unexplained by the underlying disease will also be considered a DLT.

Non-hematologic DLTs (regardless of attribution)

- Grade 3 mucositis, nausea, vomiting, or diarrhea will only be considered a DLT if they require total parenteral nutrition, tube feeding, or prolonged hospitalization (> 72 hours).
- Any Grade 4 non-hematologic laboratory abnormality.
- Any instance of an AE that meets the definition of Hy's Law.[14]

Hematologic DLTs (regardless of attribution)

For subjects with MM, hematologic DLTs are defined as:

- Grade 4 neutropenia lasting for > 5 days.
- Grade 4 anemia unexplained by underlying disease.
- Febrile neutropenia defined as ANC < 1000/mm^3 with a fever ≥ 38.3°C or ≥ 38°C for 1 hour.
- Grade 4 thrombocytopenia.
- Grade 3 thrombocytopenia associated with clinically significant bleeding.

For subjects with AML, hematologic DLTs are as follows:

- ANC < 500/mm^3 and/or platelet < 25,000/mm^3 at either/both of the following time points (and only in the setting of a bone marrow response assessment showing < 5% blast count):
  - Day 42 from first dose of ABBV-467
  - 14 days beyond cessation of ABBV-467

For AML subjects with evidence of myelosuppression at 28 days, the DLT period will be 42 days or 14 days after bone marrow biopsy showing < 5% blast count (whichever is later). NOTE: In any instance in which bone marrow response assessment is either undocumented, or consistent with ≥ 5% blast count, cytopenias should be attributed to persistence of AML, rather than study drug (and therefore should not be considered DLT).

The DLT evaluation period will be 28 days irrespective of dose frequency (should other treatment schedules be explored).

Subjects who do not complete all of the scheduled doses of ABBV-467 or who do not receive at least 80% of the planned total dose during the DLT evaluation period, due to reasons other than occurrence of DLT(s) will be considered non-evaluable for DLT, and may be replaced to ensure the accrual of the minimum number of subjects to adequately assess tolerability (other subjects who withdraw consent or refuse further follow-up will be replaced). All safety information including information on AEs, etc., observed in subjects who are excluded from DLT evaluation will be taken into consideration to determine the next dose level.

7 STATISTICAL METHODS & DETERMINATION OF SAMPLE SIZE

7.1 Statistical and Analytical Plans

Complete and specific details of the statistical analysis will be described and fully documented in the Statistical Analysis Plan (SAP). The SAP will be finalized prior to the database lock. The statistical analyses will be performed using SAS (SAS Institute Inc., Cary, North Carolina, USA).

7.2 Definition for Analysis Populations

Efficacy and safety analyses will be performed on the all-treated population which comprises all subjects who receive at least 1 dose of study drug. The PK population is defined as subjects who received at least 1 dose of study drug and have at least 1 valid post-baseline PK data; PK analyses will be based on the PK population.

Details on the analysis population definitions will be provided in the SAP.

7.3 Statistical Analyses for Efficacy

Efficacy analysis will be performed separately for subjects with R/R MM and R/R AML.

Efficacy for R/R MM subjects will include analyses of ORR (PR + VGPR + CR + sCR), DOR, and CBR.

Efficacy for R/R AML subjects will include analyses of CRc rate (CR + CRi), CRh, ORR (CRc + PR), and DOR.

Exploratory efficacy analysis includes overall survival (OS) for both R/R MM and R/R AML.

For Parts A and C, efficacy data will be listed for each dose level.

CONFIDENTIAL INFORMATION
No use or disclosure outside AbbVie is permitted without prior written authorization from AbbVie.

For Parts B and D, efficacy analyses will be performed by cohort for the all-treated population. For binary endpoints (such as ORR, CBR, CRc, CRh), the estimated proportion and the associated 2-sided 90% Clopper-Pearson (exact) confidence intervals (CIs) will be provided for each cohort. DOR will be defined as the time between date of first response and the first occurrence of progression or death from any cause, whichever occurs first. Only responders will be included in the analysis of DOR. DOR for subjects without DOR events at the time of analysis will be censored at the date of last disease assessment. If a subject receives a new anti-cancer therapy, DOR will be censored at the date of last disease assessment prior to the start of the new therapy. DOR will be summarized using the Kaplan-Meier method. The median DOR and its associated 2-sided 90% CIs will be provided for each cohort. For R/R MM subjects, DOR will be analyzed among subjects who achieved PR, VGPR, CR or sCR. For R/R AML subjects, DOR will be analyzed among subjects who achieved CRc or PR. OS will be defined as the time from the date of the first dose of study treatment to the date of death. For subjects who are not deceased, the data will be censored at the last known alive date. OS will be summarized using the Kaplan-Meier method. The median OS and its associated 2-sided 90% CIs will be provided.

Additional details on the efficacy analyses are provided in the SAP.

7.4 Statistical Analyses for Pharmacokinetics

Plasma concentrations and PK parameter values for ABBV-467 will be tabulated for each subject, and dose level. Summary statistics will be computed for each sampling time and each PK parameter.

Details on the statistical analyses for PK will be provided in the SAP.

7.5 Statistical Analyses for Safety

Safety analyses will be performed on the all-treated subjects. Subjects will be assigned to a treatment group based on the treatment received. The safety of ABBV-467 will be assessed by evaluation of study drug exposure, AEs, SAEs, and deaths, as well as changes in laboratory and vital sign parameters.

Analysis of Adverse Events

Analyses of AEs will include only treatment-emergent events. The number and percentage of subjects experiencing treatment-emergent AEs will be tabulated according to the primary Medical Dictionary for Regulatory Activities (MedDRA) system organ class (SOC) and MedDRA preferred term. Subjects reporting more than 1 AE for a given MedDRA preferred term will be counted only once for that term. Subjects reporting more than one type of AE within a MedDRA SOC will be counted only once for that SOC. Subjects reporting more than one type of AE will be counted only once in the overall total.

SAEs, drug-related AEs, AEs leading to death, dose interruption and dose modification as applicable, study treatment discontinuation, and study discontinuation will be summarized using the same methods as described above.

Analysis of Laboratory Data

Baseline values and changes from baseline will be summarized for each scheduled post-baseline visit for laboratory data as applicable. If more than one measurement exists for a subject on a particular day, an

arithmetic average will be calculated. This average will be that subject's measurement for that day. For subjects that do not have any post-baseline measurements, only their baseline values will be summarized.

Descriptive statistics will include the mean, standard deviation, and median for baseline, and mean, standard deviation, median, minimum, and maximum for change from baseline for each scheduled post-baseline visit.

Analysis of Vital Signs

Baseline values and changes from baseline will be summarized for each scheduled post-baseline visit for vital signs data as applicable using methods similar to the analysis of laboratory data.

Details on the statistical analyses for safety will be provided in the SAP.

7.6 Multiplicity Adjustment and Overall Type I Error Control

Not applicable.

7.7 Sample Size Determination

A total of approximately 108 subjects will be enrolled in this study. In the dose-escalation component, the number of subjects will depend upon the occurrences of DLT events as specified in the allocation rules for BOIN design. Including a minimum of 6 subjects treated during dose escalation at what is ultimately declared the RP2D, it is estimated that approximately 30 subjects each will be treated in Part A and Part C (approximately 60 total).

The expansion component will target enrollment of 20 response-evaluable subjects (with a maximum of 24 subjects enrolled regardless of response evaluability) each into Parts B and D (up to 48 enrolled total) to further evaluate the safety profile, and preliminarily evaluate efficacy, of ABBV-467 at RP2D. Subjects will be considered response-evaluable if they have received at least 1 dose of ABBV-467 and have died, or exited the study due to progressive disease, or had at least 1 post-baseline response assessment per the indication-specific response criteria. Subjects who die or exit for progressive disease, prior to their first baseline assessment, will be considered to have not responded to the therapy.

With a sample size of 24 subjects, the probability to observe at least 1 occurrence of AE is ≥ 92% if the true incidence of the respective AE rate is ≥ 10%.

No formal hypothesis testing is planned for this first-in-human study; preliminary assessment of efficacy is a secondary objective of this study. Table 5 provides the lower bound of a one-sided 80% CI corresponding to various observed response rates assuming a total of 20 response-evaluable subjects in each cohort. For example, if a 25% response rate is observed, the lower bound of a one-sided exact CI will exclude 15% (or lower) rates of response, providing some preliminary assessment of efficacy.

Table 5. Exact 1-Sided 80% Confidence Intervals from Various Response Rates

Cohort Size (n)	# of Responders	Observed Response Rate[a]	Exact 80% Confidence Interval
20	5	25%	(0.158, 1)
	6	30%	(0.201, 1)
	7	35%	(0.245, 1)
	8	40%	(0.291, 1)
	10	50%	(0.384, 1)

AML = acute myeloid leukemia; CR = complete remission; CRi = complete remission with incomplete blood count recovery; MM = multiple myeloma; ORR = overall response rate

a. CR/CRi for AML cohort; ORR for MM cohort.

Note: Cohort size refers to the number of response-evaluable subjects.

8 ETHICS

8.1 Independent Ethics Committee/Institutional Review Board

The protocol, informed consent form(s), recruitment materials, and all subject materials will be submitted to the IEC/IRB for review and approval. Approval of both the protocol and the informed consent form(s) must be obtained before any subject is enrolled. Any amendment to the protocol will require review and approval by the IEC/IRB before the changes are implemented to the study. In addition, all changes to the consent form(s) will be IEC/IRB approved.

8.2 Ethical Conduct of the Study

The study will be conducted in accordance with the protocol, Operations Manual, ICH guidelines, applicable regulations, and guidelines governing clinical study conduct and the ethical principles that have their origin in the Declaration of Helsinki. Responsibilities of the investigator are specified in Appendix B.

8.3 Subject Confidentiality

To protect subjects' confidentiality, all subjects and their associated samples will be assigned numerical study identifiers or "codes." No identifiable information will be provided to AbbVie.

9 SOURCE DOCUMENTS AND CASE REPORT FORM COMPLETION

The investigator is responsible for ensuring the accuracy, completeness, legibility, and timeliness of the data reported. All source documents should be attributable, legible, contemporaneous, original,

CONFIDENTIAL INFORMATION
No use or disclosure outside AbbVie is permitted without prior written authorization from AbbVie.

accurate, and complete to ensure accurate interpretation of data. Clinical site monitoring is conducted to ensure that the rights and well-being of human subjects are protected, that the reported trial data are accurate, complete, and verifiable, and that the conduct of the trial is in compliance with the currently approved protocol, ICH Good Clinical Practice (GCP), and applicable local regulatory requirement(s).

10 DATA QUALITY ASSURANCE

AbbVie will ensure that the clinical trial is conducted with a quality management system that will define quality tolerance limits in order to ensure human subject protection and reliability of study results. Data will be generated, documented, and reported in compliance with the protocol, ICH GCP, and applicable regulatory requirements.

11 COMPLETION OF THE STUDY

The end-of-study is defined as the date of the last subject's last visit or date of the last follow-up contact, whichever is later.

12 REFERENCES

1. Saultz JN, Garzon R. Acute myeloid leukemia: A concise review. J Clin Med. 2016;5(3):33.
2. Kurosawa S, Yamaguchi T, Miyawaki S, et al. Prognostic factors and outcomes of adult patients with acute myeloid leukemia after first relapse. Haematologica. 2010;95(11):1857-64.
3. Xiang W, Yang CY, Bai L. MCL-1 inhibition in cancer treatment. Onco Targets Ther. 2018;11:7301-14.
4. AbbVie. ABBV-467 Investigator's Brochure Edition 1. 2019.
5. Kumar S, Paiva B, Anderson KC, et al. International Myeloma Working Group consensus criteria for response and minimal residual disease assessment in multiple myeloma. Lancet Oncol. 2016;17(8):e328-e346.
6. Cheson BD, Bennett JM, Kopecky KJ, et al; International Working Group for Diagnosis, Standardization of Response Criteria, Treatment Outcomes, and Reporting Standards for Therapeutic Trials in Acute Myeloid Leukemia. Revised recommendations of the International Working Group for Diagnosis, Standardization of Response Criteria, Treatment Outcomes, and Reporting Standards for Therapeutic Trials in Acute Myeloid Leukemia. J Clin Oncol. 2003;21(24):4642-9.
7. Döhner H, Estey E, Grimwade D, et al. Diagnosis and management of AML in adults: 2017 ELN recommendations from an international expert panel. Blood. 2017;129(4):424-47.
8. Liu S, Yuan Y. Bayesian Optimal Interval Designs for Phase I Clinical Trials. J R Stat Soc Ser C. 2015;64:507-23.
9. Penel N, Kramar A. What does a modified-Fibonacci dose-escalation actually correspond to? BMC Med Res Methodol. 2012;12(103):1-5.

10. Fassas AB, Desikan KR, Siegel D, et al. Tumour lysis syndrome complicating high-dose treatment in patients with multiple myeloma. Br J Haematol. 1999;105(4):938-41.

11. Montesinos P, Lorenzo I, Martín G, et al. Tumor lysis syndrome in patients with acute myeloid leukemia: identification of risk factors. Haematologica. 2008;93(1):67-74.

12. Mato AR, Riccio BE, Qin L, et al. A predictive model for the detection of tumor lysis syndrome during AML induction therapy. Leuk Lymphoma. 2006;47(5):877-83.

13. Coiffier B, Altman A, Pui CH, et al. Guidelines for the management of pediatric and adult tumor lysis syndrome: an evidence-based review. J Clin Oncol. 2008;26(16):2767-78.

14. Hy's law is defined as ALT/AST and bilirubin in the normal reference range prior to study drug dosing, with subsequent development of ALT or AST > 3 × ULN along with total bilirubin > 2 × ULN, without findings of cholestasis [elevated serum ALP] and with no alternative explanation. FDA guidance on this matter may be found at: http://www.fda.gov/downloads/Drugs/Guidances/UCM174090.pdf.

15. Crockett SD, Wani S, Gardner TB, et al. American Gastroenterological Association Institute Guideline on Initial Management of Acute Pancreatitis. Gastroenterology. 2018;154(4):1096-101.

16. Rajkumar SV, Harousseau JL, Durie B, et al. Consensus recommendations for the uniform reporting of clinical trials: report of the International Myeloma Workshop Consensus Panel 1. Blood. 2011;117(18):4691-5.

17. Howard SC, Jones DP, Pui CH. The tumor lysis syndrome. N Engl J Med. 2011;364(19):1844-54.

18. Paiva B, Gutierrez NC, Rosinol L, et al. High-risk cytogenetics and persistent minimal residual disease by multiparameter flow cytometry predict unsustained complete response after autologous stem cell transplantation in multiple myeloma. Blood. 2012;119(3):687-91.

19. Zamagni E, Nanni C, Mancuso K, et al. PET/CT improves the definition of complete response and allows to detect otherwise unidentifiable skeletal progression in multiple myeloma. Clin Cancer Res. 2015;21(19):4384-90.

CONFIDENTIAL INFORMATION
No use or disclosure outside AbbVie is permitted without prior written authorization from AbbVie.

APPENDIX A. STUDY SPECIFIC ABBREVIATIONS AND TERMS

Abbreviation	Definition
AE	adverse event
ALT	alanine aminotransferase
AML	acute myeloid leukemia
AST	aspartate aminotransferase
AUC	area under the plasma concentration-time curve
AUC_t	area under the plasma concentration-time curve from time 0 to time of last measurable concentration
BCL-2	B-cell lymphoma-2
BCL-X_L	B-cell lymphoma - extra large
BOIN	Bayesian optimal interval
C	Cycle
CBR	clinical benefit rate
CI	confidence interval
CL	clearance
C_{max}	maximum observed plasma concentration
CR	complete remission
CRc	composite complete remission
CrCl	creatinine clearance
CRh	complete remission with partial hematologic recovery
CRi	complete remission with incomplete blood count recovery
CT	computed tomography
CTCAE	Common Terminology Criteria for Adverse Events
CVC	central venous catheter
D	Day
DLT	dose-limiting toxicity
DOR	duration of response
ECG	electrocardiogram
ECOG	Eastern Cooperative Oncology Group
eCRF	electronic case report form
ELN	European Leukemia Net
FFPE	formalin-fixed paraffin-embedded

abbvie

Abbreviation	Definition
FLC	free light chain
GCP	Good Clinical Practice
HED	human equivalent dose
HNSTD	highest non-severely toxic dose
ICH	International Conference on Harmonisation of Technical Requirements for Registration of Pharmaceuticals for Human Use
IEC	Independent Ethics Committee
IMP	Investigational Medicinal Product
IMWG	International Myeloma Working Group
IRB	Institutional Review Board
IRT	interactive response technology
IUD	intrauterine device
IUS	Intrauterine hormone-releasing system
IV	intravenous
IWG	International Working Group
MCL-1	myeloid cell leukemia-1
MedDRA	Medical Dictionary for Regulatory Activities
MM	multiple myeloma
MRD	minimal residual disease
MRI	magnetic resonance imaging
MTD	maximum tolerated dose
NCI	National Cancer Institute
OBD	optimal biological dose(s)
ORR	overall response rate
PD	pharmacodynamic
PET	positron emission tomography
PG	pharmacogenetics
PK	pharmacokinetic
PR	partial response
RP2D	recommended Phase 2 dose
R/R	relapsed/refractory
SAE	serious adverse event
SAP	statistical analysis plan

Abbreviation	Definition
sCR	stringent complete response
SOC	system organ class
SPEP	serum protein electrophoresis
SUSAR	suspected unexpected serious adverse reaction
TA MD	Therapeutic Area Medical Director
TLS	tumor lysis syndrome
ULN	upper limit of normal
UPEP	urine protein electrophoresis
VGPR	very good partial response

APPENDIX B. RESPONSIBILITIES OF THE INVESTIGATOR

Protocol M19-025: A First-in-Human Study of the MCL-1 Inhibitor, ABBV-467

Protocol Date: 29 April 2020

Clinical research studies sponsored by AbbVie are subject to the International Conference on Harmonisation of Technical Requirements for Registration of Pharmaceuticals for Human Use (ICH) Good Clinical Practices (GCP) and local regulations and guidelines governing the study at the site location. In signing the Investigator Agreement, the investigator is agreeing to the following:

1. Conducting the study in accordance with ICH GCP, the applicable regulatory requirements, current protocol and operations manual, and making changes to a protocol only after notifying AbbVie and the appropriate Institutional Review Board (IRB)/Independent Ethics Committee (IEC), except when necessary to protect the subject from immediate harm.
2. Personally conducting or supervising the described investigation(s).
3. Informing all subjects, or persons used as controls, that the drugs are being used for investigational purposes and complying with the requirements relating to informed consent and ethics committees (e.g., IEC or IRB) review and approval of the protocol and its amendments.
4. Reporting complaints that occur in the course of the investigation(s) to AbbVie.
5. Reading the information in the Investigator's Brochure/safety material provided, including the instructions for use and the potential risks and side effects of the investigational product(s).
6. Informing all associates, colleagues, and employees assisting in the conduct of the study about their obligations in meeting the above commitments.
7. Maintaining adequate and accurate records of the conduct of the study, making those records available for inspection by representatives of AbbVie and/or the appropriate regulatory agency, and retaining all study-related documents until notification from AbbVie.
8. Maintaining records demonstrating that an ethics committee reviewed and approved the initial clinical protocol and all of its amendments.
9. Reporting promptly, all changes in the research activity and all unanticipated problems involving risks to human subjects or others, to the appropriate individuals (e.g., coordinating investigator, institution director) and/or directly to the ethics committees and AbbVie.
10. Providing direct access to source data documents for study-related monitoring, audits, IEC/IRB review, and regulatory inspection(s).

Signature of Principal Investigator Date

Name of Principal Investigator (printed or typed)

CONFIDENTIAL INFORMATION
No use or disclosure outside AbbVie is permitted without prior written authorization from AbbVie.

APPENDIX C. LIST OF PROTOCOL SIGNATORIES

Name	Title	Functional Area
[redacted]		Clinical Program Development
		Medical Writing
		Oncology Early Development
		Early Oncology Statistics
		Early Oncology Statistics
		Pharmacokinetics
		Oncology Early Development
		Clinical Program Development

CONFIDENTIAL INFORMATION
No use or disclosure outside AbbVie is permitted without prior written authorization from AbbVie.

APPENDIX D. ACTIVITY SCHEDULE

The following table shows the required activities for this study. The individual activities are described in detail in the Operations Manual Section 3.

Study Activities Table

Activity	Screening (Day −21 to Day −1)	Cycle 1, Day 1	Cycle 1, Days 2 - 5	Cycle 1, Days 8, 15, 22 (± 1 day)	Cycle 1, Days 16 & 17	Day 1 of Each Cycle (± 1 day)	Days 8, 15, 22 of Each Cycle (± 1 day)	Final Visit	30-day F-U Visit (± 7 days)	Post-Treatment Follow-Up (every 3 months ± 7 days)
**INTERVIEWS & QUESTIONNAIRES**										
Informed consent	✓									
Eligibility criteria	✓	✓								
Medical/oncology history	✓	✓								
Adverse event assessment	✓	✓	✓	✓	✓	✓	✓	✓	✓	
Prior/concomitant therapy	✓	✓	✓	✓	✓	✓	✓	✓	✓	
**LOCAL LABS & EXAMS**										
Single 12-lead ECG	✓							✓		
Echocardiogram	✓					✓ C2 & C6 only				
Cytogenetic Testing	✓									
Vital Signs	✓	✓	✓	✓		✓	✓	✓	✓	
ECOG Performance Status	✓	✓				✓		✓	✓	
Targeted Physical Examination	✓	✓				✓		✓	✓	
Height (screening only) and Weight	✓	✓				✓		✓	✓	
TLS Prophylaxis		✓	✓	✓						
TLS Chemistry Panel		✓	✓ D2 only	✓						
Urine Pregnancy test		✓				✓		✓		
Serum Pregnancy test	✓									
Viral serologies	✓									
Coagulation/Urinalysis	✓							✓		
**CENTRAL LABS**										
Hematology/Chemistry	✓	✓	✓	✓		✓	✓	✓	✓	

No use or disclosure outside AbbVie is permitted without prior written authorization from AbbVie.

Activity	Screening (Day −21 to Day −1)	Cycle 1, Day 1	Cycle 1, Days 2 - 5	Cycle 1, Days 8, 15, 22 (± 1 day)	Cycle 1, Days 16 & 17	Day 1 of Each Cycle (± 1 day)	Days 8, 15, 22 of Each Cycle (± 1 day)	Final Visit	30-day F-U Visit (± 7 days)	Post-Treatment Follow-Up (every 3 months ± 7 days)
Triplicate ECG		✓	✓ D2 only	✓ D15 only	✓ D16 only					
Creatine phosphokinase and troponin		✓	✓ D2 & D3 only	✓ D15 only	✓	✓ C2 – C8 only	✓ D15 of C2, C4, C6, C8 only			
Serum β2 microglobulin	✓									
ABBV-467 Blood PK Samples		✓	✓ D2 & D3 only	✓ D15 only	✓	✓ C2, C4, C6, C8 only				
AML subjects (expansion only): ABBV-467 Urine PK Samples		✓	✓ D2 & D3 only							
Peripheral Blood biomarker samples	✓	✓	✓ D2 only		✓ D16 only	✓ C2, C3, C4; every 3 cycles		✓		
AML subjects: Bone marrow aspirate and biopsy for biomarker research	✓					C2, then every 3 cycles		✓		
MM subjects: Bone marrow aspirate and biopsy for biomarker research	✓					✓ C5 only		✓		
Optional biomarker samples (DNA and RNA) (taken if consent given)		✓				✓ C3 only		✓		
**DISEASE ASSESSMENT**										
AML subjects: Bone Marrow Aspirate and Biopsy for Response Assessment	✓	✓				C2, then every 3 cycles		✓		
AML subjects: Response Assessment						C2, then every 3 cycles		✓		
MM Subjects: Serum protein immunofixation	✓	✓				✓		✓		
MM Subjects: Serum protein electrophoresis	✓	✓				✓		✓		

Activity	Screening (Day −21 to Day −1)	Cycle 1, Day 1	Cycle 1, Days 2 - 5	Cycle 1, Days 8, 15, 22 (± 1 day)	Cycle 1, Days 16 & 17	Day 1 of Each Cycle (± 1 day)	Days 8, 15, 22 of Each Cycle (± 1 day)	Final Visit	30-day F-U Visit (± 7 days)	Post-Treatment Follow-Up (every 3 months ± 7 days)
MM Subjects: Serum quantitative immunoglobulins	✓	✓				✓		✓		
MM Subjects: Serum free light chains	✓	✓				✓		✓		
MM Subjects: Urine protein immunofixation	✓	✓				✓		✓		
MM Subjects: Urine protein electrophoresis	✓	✓				✓		✓		
MM Subjects: Skeletal survey/ Assessment of lytic disease	✓	As clinically indicated								
MM Subjects: Plasmacytoma evaluation	✓	As clinically indicated								
MM Subjects: Bone marrow aspirate for response assessment	✓	✓				✓		✓		
MM Subjects: Bone marrow core biopsy for response assessment	✓									
MM subjects: Response assessment						✓		✓		
Disease Status									✓	✓
Survival status									✓	✓
Subsequent anticancer therapy									✓	✓
**℞ TREATMENT**										
Hospitalization/Inpatient Monitoring (Dose Escalation Only)		✓	✓ D2 Only	✓ D8 Only						
Administer ABBV-467 (Once weekly for every 28-day cycle)		✓		✓		✓	✓			

AML = acute myeloid leukemia; C = Cycle; D = Day; ECG = electrocardiogram; ECOG = Eastern Cooperative Oncology Group; F-U = Follow-Up; MM = multiple myeloma; PK = pharmacokinetic; TLS = tumor lysis syndrome

APPENDIX E. DEFINITION OF LINES OF THERAPY (MULTIPLE MYELOMA ONLY)

According to the IMWG Consensus panel on uniform reporting criteria in clinical trial,[16] A line of therapy is defined as 1 or more cycles of a planned treatment regimen. This may consist of 1 or more planned cycles of single-agent therapy or combination therapy, as well as a sequence of treatments administered in a planned manner. For example, a planned induction, followed by autologous stem cell transplantation, followed by maintenance is considered 1 line of therapy. A new line of therapy starts when a planned course of therapy is modified to include other treatment agents (alone or in combination) as a result of disease progression, relapse or toxicity, or when a planned period of observation is interrupted by the need for additional treatment for the disease. Modification of drug doses or resuming therapy after holding will not be considered a new line of therapy provided that there was no evidence of disease progression per IMWG response criteria.

APPENDIX F. DEFINITIONS OF LABORATORY AND CLINICAL TUMOR LYSIS SYNDROME

**Metabolic Abnormality**	**Criteria for Classification of Laboratory Tumor Lysis Syndrome**	**Criteria for Classification of Clinical Tumor Lysis Syndrome**
Hyperuricemia	Uric acid > 8.0 mg/dL (475.8 µmol/L)	Not applicable
Hyperphosphatemia	Phosphorus > 4.5 mg/dL (1.5 mmol/L)	Not applicable
Hyperkalemia	Potassium > 6.0 mmol/L	Cardiac dysrhythmia or sudden death probably or definitely caused by hyperkalemia
Hypocalcemia	Corrected calcium < 7.0 mg/dL (1.75 mmol/L) or ionized calcium < 1.12 mg/dL (0.3 mmol/L)[a]	Cardiac dysrhythmia, sudden death, seizure, neuromuscular irritability (tetany, paresthesias, muscle twitching, carpopedal spasm, Trousseau's sign, Chvostek's sign, laryngospasm, or bronchospasm), hypotension, or heart failure probably or definitely caused by hypocalcemia
Acute kidney injury[b]	Not applicable	Increase in the serum creatinine level of 0.3 mg/dL (26.5 µmol/L) or the presence of oliguria, defined as an average urine output < 0.5 mL/kg/hr for 6 hours

TLS = tumor lysis syndrome

a. The corrected calcium level in mg/dL = measured calcium level in mg/dL + 0.8 × (4-albumin in g/dL).

b. Acute kidney injury, unless attributable to another cause, represents clinical TLS even if criteria for laboratory TLS are not satisfied.

Note: In laboratory TLS, 2 or more metabolic abnormalities must be present during the same 24-hour period within 3 days before the start of therapy or up to 7 days afterward. Not directly or probably attributable to therapeutic agent.

Note: Clinical TLS requires the presence of Laboratory TLS plus 1 or more findings from the Clinical TLS column.

Source: Howard et al, 2011[17]

No use or disclosure outside AbbVie is permitted without prior written authorization from AbbVie.

abbvie

APPENDIX G. RECOMMENDATIONS FOR INITIAL MANAGEMENT OF ELECTROLYTE IMBALANCES AND PREVENTION OF TUMOR LYSIS SYNDROME

Initial Dosing for All Subjects

- Within the first 24 hours after the first dose of ABBV-467, if any laboratory criteria below are met, the investigator should be notified. No additional of ABBV-467 doses should be administered until resolution. A rapidly rising serum potassium is a medical emergency.
- Nephrology (or other acute dialysis service) should be available (per institutional standards to ensure emergency dialysis is available) for any subject hospitalized prophylactically or in response to laboratory changes.
- IV fluids (e.g., D5 1/2 normal saline) should be initiated at a rate of at least 1 mL/kg/hr rounded to the nearest 10 mL (target 150 - 200 mL/hr; not < 50 mL/hr). Modification of fluid rate should also be considered for individuals with specific medical needs.
- Monitor for symptoms or signs of TLS (e.g., fever, chills, tachycardia, nausea, vomiting, diarrhea, diaphoresis, hypotension, muscle aches, weakness, paresthesias, mental status changes, confusion, seizures). If any clinical features are observed, recheck potassium, phosphorus, uric acid, calcium and creatinine within 1 hour STAT.
- Vital signs should be taken at time of all blood draws or any intervention if TLS is clinically suspected.
- The management recommendations below focus on the minimum initial responses required. If a diagnosis of TLS is established, ongoing intensive monitoring and multi-disciplinary management will be per institutional protocols.

Ongoing Dosing for All Subjects

- Management of electrolyte changes from last value at intervals > 24 hours after either the first dose is as below. Note: If any laboratory criteria below are met, no additional doses should be administered until resolution.
- For potassium, admit subject (if not already hospitalized) for any increase ≥ 1.0 mmol/L (1.0 mEq/L), or any level > ULN.
- Refer to the management guidelines for electrolyte changes observed within the first 24 hours after either the first dose or dose escalation (see below table).
- If a smaller potassium increase is observed that does not meet the criteria for admission above, recheck potassium, phosphorus, uric acid, calcium and creatinine in 24 hours and confirm no evidence of tumor lysis prior to further dosing.
- For uric acid, calcium, phosphorus and creatinine, refer to the management guidelines for electrolyte changes observed within the first 24 hours after either the first dose or dose escalation.

CONFIDENTIAL INFORMATION
No use or disclosure outside AbbVie is permitted without prior written authorization from AbbVie.

Abnormality	Management Recommendations
**Hyperkalemia (including rapidly rising potassium)**	
Potassium ≥ 0.5 mmol/L increase from prior value (even if potassium WNL)	• Recheck potassium, phosphorus, uric acid, calcium and creatinine in 1 hour STAT. If further ≥ 0.2 mmol/L increase in potassium, but still < ULN, manage as per potassium ≥ ULN. Otherwise recheck in 1 hour.<br>• Resume per protocol testing if change in potassium is < 0.2 mmol/L, and potassium < ULN, and no other evidence of tumor lysis.<br>• At discretion of investigator, may recheck prior to hospitalization. If stable or decreased, and still WNL, hospitalization is at the discretion of the investigator. Potassium, phosphorus, uric acid, calcium and creatinine must be rechecked within 24 hours.
Potassium > ULN	• Perform STAT ECG and commence telemetry.<br>• Nephrology (or other acute dialysis service) notification with consideration of initiating dialysis.<br>• Administer Kayexalate 60 g (or Resonium A 60 g).<br>• Administer furosemide 20 mg IV × 1.<br>• Administer calcium gluconate 100 – 200 mg/kg IV slowly if there is ECG/telemetry evidence of life-threatening arrhythmias.<br>• Recheck potassium, phosphorus, uric acid, calcium and creatinine in 1 hour STAT.<br>• If potassium < ULN 1 hour later, repeat potassium, phosphorus, uric acid, calcium and creatinine 1, 2 and 4 hours, if no other evidence of tumor lysis.
Potassium ≥ 6.0 mmol/L (6.0 mEq/L) and/or symptomatic (e.g., muscle cramps, weakness, paresthesias, nausea, vomiting, diarrhea)	• Perform STAT ECG and commence telemetry.<br>• Nephrology (or other acute dialysis service) assessment with consideration of initiating dialysis.<br>• Administer Kayexalate 60 g (or Resonium A 60 g).<br>• Administer furosemide 20 mg IV × 1.<br>• Administer insulin 0.1 U/kg IV + D25 2 mL/kg IV.<br>• Administer sodium bicarbonate 1 – 2 mEq/kg IV push.<br>• If sodium bicarbonate is used, rasburicase should not be used as this may exacerbate calcium phosphate precipitation.<br>• Administer calcium gluconate 100 – 200 mg/kg IV slowly if there is ECG/telemetry evidence of life-threatening arrhythmias. Do not administer in same IV line as sodium bicarbonate.<br>• Recheck potassium, phosphorus, uric acid, calcium and creatinine every hour STAT.

Abnormality	Management Recommendations
**Hyperuricemia**	
Uric acid ≥ 8.0 mg/dL (476 µmol/L)	• Consider rasburicase (prior to rasburicase administration please refer to local label for tests to be performed, contraindications and precautions. Dosing is per institutional guidelines).<br>◦ If rasburicase is used, sodium bicarbonate should not be used as this may exacerbate calcium phosphate precipitation.<br>• Recheck potassium, phosphorus, uric acid, calcium and creatinine in 1 hour STAT.
Uric acid ≥ 10 mg/dL (595 µmol/L)<br>or<br>Uric acid ≥ 8.0 mg/dL (476 µmol/L) with 25% increase and creatinine increase ≥ 0.3 mg/dL (≥ 0.027 mmol/L) from pre-dose level	• Administer rasburicase (prior to rasburicase administration please refer to local label for tests to be performed, contraindications and precautions. Dosing is per institutional guidelines).<br>◦ If rasburicase is used, sodium bicarbonate should not be used as this may exacerbate calcium phosphate precipitation.<br>• Notify nephrology (or other acute dialysis service).<br>• Recheck potassium, phosphorus, uric acid, calcium and creatinine in 1 hour STAT.<br>• If uric acid < 8.0 mg/dL 1 hour later, repeat potassium, phosphorus, uric acid, calcium and creatinine 2 and 4 hours later, if no other evidence of tumor lysis.
**Hypocalcemia**	
Calcium ≤ 7.0 mg/dL (1.75 mmol/L)<br>and<br>Subject symptomatic (e.g., muscle cramps, hypotension, tetany, cardiac arrhythmias)	• Administer calcium gluconate 50 – 100 mg/kg IV slowly with ECG monitoring.<br>• Telemetry.<br>• Recheck potassium, phosphorus, uric acid, calcium and creatinine in 1 hour STAT.<br>• If calcium normalized 1 hour later, repeat potassium, phosphorus, uric acid, calcium and creatinine 2 and 4 hours later, if no other evidence of tumor lysis.<br>• Calculate corrected calcium and check ionized calcium if albumin low.
**Hyperphosphatemia**	
Phosphorus ≥ 5.0 mg/dL (1.615 mmol/L) with ≥ 0.5 mg/dL (0.16 mmol/L) increase	• Administer a phosphate binder (e.g., aluminum hydroxide, calcium carbonate, sevelamer hydroxide, or lanthanum carbonate).<br>• Nephrology (or other acute dialysis service) notification (dialysis required for phosphorus ≥ 10 mg/dL).<br>• Recheck potassium, phosphorus, uric acid, calcium and creatinine in 1 hour STAT.<br>• If phosphorus < 5.0 mg/dL 1 hour later, repeat potassium, phosphorus, uric acid, calcium and creatinine 2 and 4 hours later, if no other evidence of tumor lysis.

CONFIDENTIAL INFORMATION
No use or disclosure outside AbbVie is permitted without prior written authorization from AbbVie.

Abnormality	Management Recommendations
**Creatinine**	
Increase ≥ 25% from baseline	• Start or increase rate of IV fluids.<br>• Recheck potassium, phosphorus, uric acid, calcium and creatinine in 1 – 2 hours STAT.

ECG = electrocardiogram; IV = intravenous; STAT = statum (immediately); ULN = upper limit of normal; WNL = within normal limits

APPENDIX H. MONITORING AND MANAGEMENT OF DRUG-INDUCED LIVER INJURY

Hepatic abnormalities will be graded based on the criteria in the table below, adapted from CTCAE v5.0.

MedDRA SOC	CTCAE Term	Grade 1	Grade 2	Grade 3	Grade 4	Grade 5
Investigations	ALT increased	> ULN – 3 × ULN if baseline was normal; 1.5 - 3 × baseline if baseline was abnormal	> 3 - 5 × ULN if baseline was normal; > 3.0 - 5.0 × baseline if baseline was abnormal	> 5 - 20 × ULN if baseline was normal; > 5 - 20 × baseline if baseline was abnormal	> 20 × ULN if baseline was normal; > 20 × baseline if baseline was abnormal	-
Investigations	AST increased	> ULN - 3 × ULN if baseline was normal; 1.5 - 3 × baseline if baseline was abnormal	> 3 - 5 × ULN if baseline was normal; > 3 - 5 × baseline if baseline was abnormal	> 5 - 20 × ULN if baseline was normal; > 5 – 20 × baseline if baseline was abnormal	> 20 × ULN if baseline was normal; > 20 × baseline if baseline was abnormal	-

ALT = alanine aminotransferase; AST = aspartate aminotransferase; CTCAE = Common Terminology Criteria for Adverse Events; MedDRA = Medical Dictionary for Regulatory Activities; SOC = system organ class; ULN = upper limit of normal

An algorithm for monitoring and management of drug-induced liver injury in subjects with normal or elevated baseline ALT (defined as the average of 2 screening measurements) is presented in the table below.

No use or disclosure outside AbbVie is permitted without prior written authorization from AbbVie.

Treatment-Emergent ALT and/or AST	Treatment-Emergent TBL and/or INR	Symptoms[a]	Action	Assessment/Management
> 1 - < 3 × ULN	Normal<br>(TBL < 2 × baseline in subjects with Gilbert syndrome)	Absent	• Continue treatment; or consider holding treatment for concerning lab value trend<br>• Monitor LFTs every 3 - 5 days<br>• Follow-up for symptoms	• Rule out viral etiology, hepatic dysfunction related to underlying malignancy, and/or other drug-induced transaminase elevations<br>• Consider ultrasound<br>• Consider MRCP if ultrasound non-diagnostic<br>• Limit/discontinue hepatotoxic medications (assess acetaminophen, dietary supplement, and alcohol use)
≥ 3 - < 5 × ULN	Normal<br>(TBL < 2 × baseline in subjects with Gilbert syndrome)	Absent	• Hold treatment<br>• Monitor LFTs every 3 - 5 days<br>• Restart study drug at ≥ 33% dose reduction if LFTs return to baseline or < 3 × ULN (permanently discontinue treatment if LFTs become abnormal with treatment at reduced dose)	
≥ 3 - < 5 × ULN	> 2 × ULN and/or INR > 2.0	Absent or present	• Permanently discontinue treatment	• Consider inpatient evaluation<br>• Monitor LFTs every 1 - 2 days<br>• Consider hepatology consultation
≥ 5 × ULN	Any	Absent or present	• Permanently discontinue treatment unless another etiology is identified and liver enzymes return to baseline	

ALT = alanine aminotransferase; AST = aspartate aminotransferase; INR = international normalized ratio; LFT = liver function test; MRCP = magnetic resonance cholangiopancreatography; TBL = total bilirubin; ULN = upper limit of normal

a. Symptoms are defined as any constellation of right upper quadrant pain, swelling, and/or tenderness; marked fatigue; jaundice; nausea and/or vomiting; rash; eosinophilia; asterixis; and/or other symptoms that are consistent with symptomatic liver disease in the opinion of the treating investigator.

No use or disclosure outside AbbVie is permitted without prior written authorization from AbbVie.

APPENDIX I. PROTOCOL SUMMARY OF CHANGES

Previous Protocol Versions

Protocol	Date
Version 1.0	10 September 2019
Version 1.1 (Japan only)	07 October 2019
Version 2.0	25 October 2019

The purpose of this Version is to respond to regulatory agency feedback and make additional updates, including the following:

- Added exclusion of subjects with known history of hepatitis B and HIV infection.

  Rationale: *To ensure subject safety.*

- Broadened eligibility criteria for MM subjects.

  Rationale: *To broaden the study population that can enroll in the study based on measurable disease as defined by IMWG consensus criteria.*

- Revised collection windows for PK time points.

  Rationale: *Since triplicate ECG are collected in conjunction with (and prior to) PK sample collection, the larger windows of PK timepoints provides added flexibility for PK collection and minimizes the likelihood of protocol deviations.*

- Revisions to clarify that TLS chemistry samples are only required during Cycle 1.

  Rationale: *Updated for clarity.*

- Revised collection of weight, ECOG, and physical exam to be only on Day 1 of every cycle.

  Rationale: *Correction.*

- Added response assessment for AML subjects to Day 1 of Cycle 2, every 3 cycles thereafter and final visit.

  Rationale: *Correction.*

- Revised "MM: subsequent myeloma therapy" to "Subsequent anticancer therapy" so as to collect this information for all subjects.

  Rationale: *Correction.*

- Added language to state that if subject's weight is within 10% of weight at C1D1, no dose adjustment is necessary.

  Rationale: *Updated for clarity.*

- Removed 3-day window for vital sign collection to ensure that vital signs measurements are collected on the appropriate visit day.

  Rationale: *To ensure subject safety.*

- Removed triplicate ECG at screening and added a second time point on C1D1.

  Rationale: *To capture variability in triplicate ECG reading at baseline.*

- Added troponin and creatine phosphokinase collection at Day 1 of all cycles through Cycle 8 (instead of just even cycles).

  Rationale: *To ensure the safety of subjects.*

- Clarified that disease status should be collected for all subjects who discontinue study drug treatment for reasons other than disease progression.

  Rationale: *Updated for clarity.*

- Clarified that IMWG serum and urine labs do not need to be collected on C1D1 if Screening labs were collected within 7 days of C1D1.

  Rationale: *To ease the burden on the subject.*

- Revised follow-up call timing to be every 3 months instead of every 30 days.

  Rationale: *To ease the burden on the subject.*

- Modified response-evaluable definition within sample size justification, removed reference to statistical analysis for adverse events of special interest, and added information regarding overall survival analyses.

  Rationale: *To clarify planned statistical analyses.*

- Added allowance for peripheral IV to be used for study drug administration for doses below 1.4 mg/kg.

  Rationale: *To allow for study drug administration via peripheral IV (at doses below 1.4 mg/kg) if CVC is unavailable or contraindicated.*

- Updates were made to the Safety Considerations section based updated AbbVie template.

  Rationale: *For consistency with company standards.*

- Updated wording of Toxicity Management sections to further clarify study drug action requirements/guidance and/or Sponsor notification.

  Rationale: For additional clarity.

- Included a list of medications known to prolong the QT interval to the Operations Manual appendix.

  Rationale: *To ensure safety of subjects.*

- Removed 7-day window for biomarker samples.

  Rationale: *Correction.*

In addition, minor clerical errors were corrected and edits for consistency within the document were made.

APPENDIX J. OPERATIONS MANUAL

Operations Manual for Clinical Study Protocol M19-025

Multiple Myeloma and Acute Myeloid Leukemia: A First-in-Human Study of an MCL-1 Inhibitor

SPONSOR:	AbbVie Inc.	ABBVIE INVESTIGATIONAL PRODUCT:	ABBV-467

FULL TITLE: A First-in-Human Study of the MCL-1 Inhibitor, ABBV-467

No use or disclosure outside AbbVie is permitted without prior written authorization from AbbVie.

1 CONTACTS

**Sponsor/ Emergency Medical Contact**	[redacted]<br>AbbVie Inc<br>[redacted]<br>1 North Waukegan Road<br>North Chicago, IL 60064<br><br>**<u>EMERGENCY 24-hour Number:</u>**<br>**+1 (973) 784-6402**	Office: [redacted]<br>Mobile:<br>Email:
**Safety Concerns**	AbbVie Oncology Safety Team<br>Bldg. AP30-3<br>1 North Waukegan Road<br>North Chicago, IL 60064	Phone: (847) 935-2609<br>Email:<br>SafetyManagement_Oncology@abbvie.com
**SAE Reporting outside of RAVE**	Email:<br>PPDINDPharmacovigilance@abbvie.com	Fax: +1 (847) 938-0660
**Protocol Deviations and Product complaints**	[redacted]<br>Study Project Manager I<br>AbbVie Inc.<br>[redacted]<br>1 North Waukegan Road<br>North Chicago, IL 60064	Phone: [redacted]<br>Fax:<br>Email:
**Certified Clinical Lab**	<u>For sites in the United States</u><br>Covance Central Laboratory Services L.P.<br>8211 SciCor Drive<br>Indianapolis, IN 46214, USA<br><u>For sites in Europe:</u><br>Covance<br>Rue Moise-Marcinhes 7<br>1217 Meyrin/Geneva-CH, Switzerland<br><u>For sites in Asia Pacific:</u><br>1 International Business Park<br>The Synergy, #01-01<br>Singapore 609917, Singapore<br><u>For sites in Japan:</u><br>CB Lab c/o BML General Laboratory<br>1361-1 Matoba<br>Kawagoe-shi<br>Saitama 350-1101, Japan	<u>For sites in the United States</u><br>Phone: +1 866-762-6209 (Toll free)<br>+1 317-271-1200 (Local calls)<br>Fax: +1 317-273-4030<br><u>For site in Europe</u>:<br>Phone: +41 58 822 79 01<br>Fax: +41 58 822 75 21<br><br><u>For sites in Asia Pacific:</u><br>Phone: +65-6560-8793<br>Fax: +65-6565-5901<br><br><u>For sites in Japan</u><br>Phone: 0120 123 905 (Toll free)<br>+81 3 6837 9536 (Direct line)<br>Fax: +81 3 5250 0360

CONFIDENTIAL INFORMATION
No use or disclosure outside AbbVie is permitted without prior written authorization from AbbVie.

**Bioanalytical Lab**	Bioanalysis<br>AbbVie Inc.<br>Dept. R46W Bldg. AP13A<br>1 North Waukegan Road<br>North Chicago, IL 60064	Phone:<br>Fax:	(847) 937-0889<br>(847) 938-9898
**Triplicate ECG**	[redacted]<br>AbbVie Inc.<br>[redacted]<br>480 South US Route 45<br>Grayslake, IL 60030<br>Email: cscsoperations@abbvie.com	Phone:<br>Fax:	[redacted]

CONFIDENTIAL INFORMATION
No use or disclosure outside AbbVie is permitted without prior written authorization from AbbVie.

abbvie

TABLE OF CONTENTS

1 CONTACTS 2

2 PROTOCOL ACTIVITIES BY VISIT 7

2.1 Individual Treatment Period Visit Activities 7

2.2 Individual Post-Treatment Period Visit Activities 12

3 STUDY PROCEDURES 14

3.1 Study Subject Information and Informed Consent 14

3.2 Medical/Oncology History 15

3.3 Concomitant Medication 15

3.4 Adverse Event Assessment 16

3.5 Eastern Cooperative Oncology Group Performance Status 16

3.6 Cytogenetic Testing 16

3.7 Pharmacokinetic Sampling 17

3.8 Biomarker Research Sampling 18

3.9 12-Lead Electrocardiogram 22

3.10 Echocardiogram 24

3.11 Vital Signs 24

3.12 Targeted Physical Examination 24

3.13 Height and Weight 25

3.14 Tumor Lysis Syndrome Prophylaxis 25

3.15 Clinical Laboratory Tests 25

3.16 Disease Assessments 30

3.17 Hospitalization/Inpatient Monitoring 34

3.18 Dispense Study Drug 34

3.19 Subject Withdrawal from Study 34

4 SAFETY MANUAL 34

4.1 Methods and Timing of Safety Assessment 34

4.2 Recording Data and Analyses of Safety Findings 35

CONFIDENTIAL INFORMATION

No use or disclosure outside AbbVie is permitted without prior written authorization from AbbVie.

4.3 Reporting Adverse Events and Intercurrent Illnesses 35

5 COUNTRY-SPECIFIC REQUIREMENTS 36

5.1 Japan-Specific Information 36

5.2 SUSAR Reporting 40

6 STUDY DRUG 40

6.1 Treatments Administered 40

6.2 Packaging and Labeling 40

6.3 Method of Assigning Subjects to Treatment Groups 41

6.4 Selection and Timing of Dose for Each Subject 41

6.5 Preparation/Reconsitution of Dosage Form 41

7 APPENDICES 42

7.1 HEPATITIS B VIRUS TESTING GUIDELINES FOR ELIGIBILTY 42

7.2 NATIONAL COMPREHENSIVE CANCER NETWORK RISK CATEGORIZATION: GUIDELINES FOR AML (Version 1.2019) 43

7.3 INTERNATIONAL MYELOMA WORKING GROUP RESPONSE CRITERIA 44

7.4 INTERNATIONAL WORKING GROUP RESPONSE CRITERIA FOR ACUTE MYELOID LEUKEMIA 47

7.5 INHIBITORS AND SUBSTRATES TO BE USED WITH CAUTION 49

7.6 GUIDE OF EXCLUDED MEDICATION THAT AFFECT QT INTERVAL 50

LIST OF TABLES

TABLE 1. ABBV-467 PHARMACOKINETIC BLOOD SAMPLING TIMES 17

TABLE 2. ABBV-467 AML EXPANSION COHORT URINE COLLECTION TIMES 17

TABLE 3. SAMPLING TIMES FOR BIOMARKERS - SUBJECTS WITH MULTIPLE MYELOMA 19

TABLE 4. SAMPLING TIMES FOR BIOMARKERS - SUBJECTS WITH AML 20

TABLE 5. ABBV-467 TRIPLICATE ELECTROCARDIOGRAM TIMING 23

TABLE 6. CENTRAL CLINICAL LABORATORY TESTS 27

TABLE 7. LOCAL CLINICAL LABORATORY TESTS 28

CONFIDENTIAL INFORMATION

No use or disclosure outside AbbVie is permitted without prior written authorization from AbbVie.

TABLE 8. TLS CHEMISTRY PANEL SAMPLING TIMES 29

TABLE 9. CREATINE PHOSPHOKINASE AND TROPONIN SAMPLING TIMES 29

LIST OF FIGURES

FIGURE 1. GUIDELINES FOR THE PREVENTION OF HEPATITIS B VIRUS REACTIVATION IN SUBJECTS RECEIVING IMMUNOSUPPRESSIVE THERAPY OR CHEMOTHERAPY 37

CONFIDENTIAL INFORMATION
No use or disclosure outside AbbVie is permitted without prior written authorization from AbbVie.

2 PROTOCOL ACTIVITIES BY VISIT

2.1 Individual Treatment Period Visit Activities

This section presents a list of activities performed during each visit, organized by visit. The dot pattern on the upper right indicates the place of the visit in the overall Treatment Period Activity Schedule.

Activities are grouped by category (Interview, Exam, etc.). Further information about each activity is provided in Section 3.

SCREENING (Days –21 to –1):

INTERVIEWS & QUESTIONNAIRES	• Informed consent<br>• Eligibility criteria<br>• Medical/oncology history	• Adverse event assessment<br>• Prior/concomitant medications
LOCAL LABS & EXAMS	• Single 12-Lead ECG<br>• Cytogenetic testing<br>• Vital Signs<br>• ECOG Performance status<br>• Targeted physical examination	• Height and weight<br>• Serum pregnancy test<br>• Viral serologies<br>• Coagulation/urinalysis<br>• Echocardiogram
CENTRAL LABS	• Hematology/chemistry<br>• Serum β2 microglobulin	• Peripheral blood biomarker samples<br>• BM aspirate and biopsy biomarker samples
DISEASE ASSESSMENT	• AML: BM aspirate and biopsy for response assessments<br>• MM: Serum protein immunofixation, serum protein electrophoresis, serum quantitative immunoglobulins, serum FLC, urine protein immunofixation, urine protein electrophoresis	• MM: skeletal survey/assessment of lytic disease, plasmacytoma evaluation, BM aspirate and core biopsy for response assessment

AML = acute myeloid leukemia; BM = bone marrow; ECG = electrocardiogram; ECOG = Eastern Cooperative Oncology Group; FLC = free light chain; MM = multiple myeloma

NOTES: -For AML and MM: BM biopsy and aspirate biomarker samples to be split from samples taken for disease assessments.

No use or disclosure outside AbbVie is permitted without prior written authorization from AbbVie.

CYCLE 1, DAY 1

INTERVIEW	• Confirm eligibility criteria<br>• Medical/oncology history	• Adverse event assessment<br>• Prior/concomitant therapy
LOCAL LABS AND EXAMS	• Vital signs<br>• ECOG performance status<br>• Targeted physical examination<br>• Weight	• TLS prophylaxis<br>• TLS chemistry panel<br>• Urine pregnancy test
CENTRAL LABS	• Hematology/chemistry<br>• Triplicate ECG<br>• Creatine phosphokinase and troponin<br>• Blood PK samples<br>• Urine PK samples	• Peripheral blood biomarker samples (predose and postdose)<br>• Optional biomarker samples (if consent given)
DISEASE ASSESSMENT	• AML: BM aspirate and biopsy for response assessments<br>• MM: Serum protein immunofixation, serum protein electrophoresis, serum quantitative immunoglobulins, serum FLC; urine protein immunofixation, urine protein electrophoresis	• MM: skeletal survey/assessment of lytic disease, plasmacytoma evaluation (as clinically indicated)<br>• MM: BM aspirate for response assessment
TREATMENT	• Hospitalization/inpatient monitoring (Dose Escalation only)<br>• ABBV-467 administration (Once weekly for every 28-day cycle)	

AML = acute myeloid leukemia; BM = bone marrow; C1D1 = Cycle 1 Day 1; ECG = electrocardiogram; ECOG = Eastern Cooperative Oncology Group; DLT = dose-limiting toxicity; FLC = free light chain; MM = multiple myeloma; PK = pharmacokinetic; TLS = tumor lysis syndrome

NOTES:

-Urine pregnancy test must be obtained if it has been > 7 days since obtaining the serum pregnancy results at screening.

-Urine PK samples are taken for AML expansion cohort only (Part D).

-BM aspirate/biopsy only required at Day 1 if not performed during screening; see Section 3.16 for further details regarding required disease assessments.

-For MM: serum and urine samples for response assessments do not need to be collected if the Screening samples were collected within 7 days of C1D1.

-The ABBV-467 dose on C1D1 will be administered at half the target dose for a given subject.

-Subjects enrolled in dose escalation cohorts in Japan will be hospitalized for the duration of the DLT period (see Section 5.1).

-See Table 8 for TLS chemistry panel sampling times

CONFIDENTIAL INFORMATION

No use or disclosure outside AbbVie is permitted without prior written authorization from AbbVie.

Cycle 1, Days 2 - 5:

INTERVIEW	• Adverse event assessment	• Prior/concomitant medications
LOCAL LABS AND EXAMS	• Vital signs	• TLS prophylaxis<br>• TLS chemistry panel (Day 2 only)
CENTRAL LABS	• Hematology/chemistry<br>• Triplicate ECG (Day 2 only)<br>• Creatine phosphokinase and troponin (Days 2 & 3 only)<br>• Blood PK samples (Days 2 & 3 only)	• Urine PK samples (Days 2 & 3 only)<br>• Peripheral blood biomarker sample (Day 2 only)
DISEASE ASSESSMENT	• MM: skeletal survey/assessment of lytic disease, plasmacytoma evaluation (as clinically indicated)	
TREATMENT	• Hospitalization/inpatient monitoring (Dose Escalation, Day 2 only)	

AML = acute myeloid leukemia; DLT = dose-limiting toxicity; ECG = electrocardiogram; ECOG = Eastern Cooperative Oncology Group; MM = multiple myeloma; PK = pharmacokinetic; TLS = tumor lysis syndrome

NOTES: -Urine PK samples are taken on Cycle 1, Days 2 and 3 for AML expansion cohort only (Part D).

-Subjects enrolled in dose escalation cohorts in Japan will be hospitalized for the duration of the DLT period (see Section 5.1).

-See Table 8 for TLS chemistry sampling time points.

No use or disclosure outside AbbVie is permitted without prior written authorization from AbbVie.

Cycle 1, Days 8, 15, 22 (± 1 day):

INTERVIEW	• Adverse event assessment	• Prior/concomitant medications
LOCAL LABS AND EXAMS	• Vital signs	• TLS prophylaxis<br>• TLS chemistry panel
CENTRAL LABS	• Hematology/chemistry<br>• Creatine phosphokinase and troponin (Day 15 only)	• Triplicate ECG (Day 15 only)<br>• Blood PK Samples (Day 15 only)
DISEASE ASSESSMENT	• MM: skeletal survey/assessment of lytic disease, plasmacytoma evaluation (as clinically indicated)	
TREATMENT	• Hospitalization/inpatient monitoring (Dose Escalation, Day 8 only)<br>• ABBV-467 administration (Once weekly for every 28-day cycle)	

DLT = dose-limiting toxicity; ECG = electrocardiogram; MM = multiple myeloma; PK = pharmacokinetic; TLS = tumor lysis syndrome

NOTE: -Subjects enrolled in dose escalation cohorts in Japan will be hospitalized for the duration of the DLT period (see Section 5.1).

-See Table 8 for TLS chemistry sampling time points.

Cycle 1, Days 16 & 17

INTERVIEW	• Adverse event assessment	• Prior/concomitant medications
CENTRAL LABS	• Triplicate ECG (Day 16 only)<br>• Creatine phosphokinase and troponin<br>• Blood PK Samples	• Peripheral blood biomarker sample (Day 16 only)
DISEASE ASSESSMENT	• MM: skeletal survey/assessment of lytic disease, plasmacytoma evaluation (as clinically indicated)	

DLT = dose-limiting toxicity; ECG = electrocardiogram; MM = multiple myeloma; PK = pharmacokinetic

NOTE: -Subjects enrolled in dose escalation cohorts in Japan will be hospitalized for the duration of the DLT period (see Section 5.1).

CONFIDENTIAL INFORMATION

No use or disclosure outside AbbVie is permitted without prior written authorization from AbbVie.

Day 1 of Each Cycle (± 1 day):

INTERVIEW	• Adverse event assessment	• Prior/concomitant medications
LOCAL LABS AND EXAMS	• Vital signs<br>• ECOG performance status<br>• Targeted physical examination<br>• Weight	• Urine pregnancy test<br>• Echocardiogram (Cycle 2 and Cycle 6 only)
CENTRAL LABS	• Hematology/chemistry<br>• Creatine phosphokinase and troponin (Cycles 2 – 8 only)<br>• Blood PK Samples (Cycles 2, 4, 6, & 8 only)<br>• Peripheral blood biomarker samples (Cycles 2, 3, 4, and every 3 cycles thereafter)	• AML: BM aspirate and biopsy biomarker samples (Cycle 2 and every 3 cycles thereafter)<br>• MM: BM aspirate and biopsy biomarker samples (Cycle 5 only)<br>• Optional biomarker samples (Cycle 3 only, if consent given)
DISEASE ASSESSMENT	• AML: BM aspirate and biopsy for response assessments (Cycle 2 and every 3 cycles thereafter)<br>• AML: Response assessment (Cycle 2 and every 3 cycles thereafter)	• MM: Serum protein immunofixation, serum protein electrophoresis, serum quantitative immunoglobulins, serum FLC, urine protein immunofixation, urine protein electrophoresis<br>• MM: skeletal survey/assessment of lytic disease, plasmacytoma evaluation (as clinically indicated)<br>• MM: BM aspirate for response assessment, response assessment
TREATMENT	• ABBV-467 administration (Once weekly for every 28-day cycle)	

AML = acute myeloid leukemia; BM = bone marrow; CR = complete remission; ECOG = Eastern Cooperative Oncology Group; FLC = free light chain; MM = multiple myeloma; PK = pharmacokinetic; PR = partial response; sCR = stringent complete response

NOTES: After screening, surveillance echocardiogram is required within approximately 7 days of Cycles 2 and 6 for all subjects. In addition, echocardiogram should be obtained at any time during the study, for any subjects presenting with treatment-emergent signs, symptoms, or clinical findings that warrant an echocardiogram, as determined by the investigator or AbbVie TA MD. See Section 3.10 for further detail.

-MM: BM samples for response assessments to be taken at any time of suspected CR to confirm sCR/CR, and at 12, 18, and 24 months post confirmation of sCR/CR; aspirate is optional at the time of disease progression. Plasmacytoma evaluations should be performed to confirm PR/CR or disease progression, and otherwise as clinically indicated (approximately every 12 to 16 weeks).

-BM biopsy and aspirate biomarker samples to be split from samples taken for disease assessments.

-See Section 3.16 for further details regarding required disease assessments.

CONFIDENTIAL INFORMATION
No use or disclosure outside AbbVie is permitted without prior written authorization from AbbVie.

-After completion of Cycle 1, ABBV-467 doses may be delayed for up to 1 day due to scheduling conflicts; delays longer than 1 day should result in the dose being skipped.

Days 8, 15, and 22 of Each Cycle (± 1 day):

INTERVIEW	• Adverse event assessment	• Prior/concomitant medications
LOCAL LABS AND EXAMS	• Vital signs	
CENTRAL LABS	• Hematology/chemistry	• Creatine phosphokinase and troponin (Day 15 of Cycles 2, 4, 6, & 8 only)
DISEASE ASSESSMENT	• MM: skeletal survey/assessment of lytic disease, plasmacytoma evaluation (as clinically indicated)	
TREATMENT	• ABBV-467 administration (Once weekly for every 28-day cycle)	

MM = multiple myeloma

NOTES: -After completion of Cycle 1, ABBV-467 doses may be delayed for up to 1 day due to scheduling conflicts; delays longer than 1 day should result in the dose being skipped.

2.2 Individual Post-Treatment Period Visit Activities

This section presents a list of activities performed during each visit, organized by visit. The dot pattern on the upper right indicates the place of the visit in the overall Post-Treatment Period Activity Schedule.

Activities are grouped by category (Interview, Exam, etc.). Further information about the activities is provided in Section 3.

CONFIDENTIAL INFORMATION
No use or disclosure outside AbbVie is permitted without prior written authorization from AbbVie.

Final Visit:

INTERVIEW	• Adverse event assessment	• Prior/concomitant medications
LOCAL LABS AND EXAMS	• Single 12-lead ECG<br>• Vital signs<br>• ECOG performance status<br>• Targeted physical examination	• Weight<br>• Urine pregnancy test<br>• Coagulation/urinalysis
CENTRAL LABS	• Hematology/chemistry<br>• Peripheral blood biomarker samples	• BM aspirate and biopsy biomarker samples<br>• Optional biomarker samples (if consent given)
DISEASE ASSESSMENT	• AML: BM aspirate and biopsy for response assessments<br>• AML: response assessment<br>• MM: Serum protein immunofixation, serum protein electrophoresis, serum quantitative immunoglobulins, serum FLC, urine protein immunofixation, urine protein electrophoresis	• MM: skeletal survey/assessment of lytic disease, plasmacytoma evaluation (as clinically indicated)<br>• MM: BM aspirate for response assessment, response assessment

AML = acute myeloid leukemia; BM = bone marrow; ECG = electrocardiogram; ECOG = Eastern Cooperative Oncology Group; FLC = free light chain; MM = multiple myeloma

NOTES: -Final Visit procedures should be performed when a subject discontinues from the study.

-Final visit ECG may be obtained within ± 2 days of visit.

-See Section 3.16 for further details regarding required disease assessments.

-For AML and MM: BM biopsy and aspirate biomarker samples to be split from samples taken for disease assessments. See Section 3.8 for further details on biomarker sample requirements.

CONFIDENTIAL INFORMATION

No use or disclosure outside AbbVie is permitted without prior written authorization from AbbVie.

Follow-Up Visit (30 days post last dose, ± 7 days):

INTERVIEW	• Adverse event assessment	• Prior/concomitant medications
LOCAL LABS AND EXAMS	• Vital signs<br>• ECOG performance status	• Targeted physical examination<br>• Weight
CENTRAL LAB	• Hematology/chemistry	
DISEASE ASSESSMENT	• Disease status<br>• Survival status	• Subsequent anticancer therapy

ECOG = Eastern Cooperative Oncology Group

Post Treatment Follow-Up (Every 3 months post Follow-Up Visit, ± 7 days):

DISEASE ASSESSMENT	• Disease Status<br>• Survival status	• Subsequent anticancer therapy

NOTES: -For subjects who discontinue treatment for reasons other than disease progression, disease status will be collected until progressive disease or subject initiates new anticancer therapy, whichever occurs first.

-Post treatment follow-up (including survival status collection) does not require an on-site visit. Required visit information may be collected via phone call, subject medical record review, or public information sources, as appropriate per local regulations.

3 STUDY PROCEDURES

3.1 Study Subject Information and Informed Consent

The investigator or his/her representative will explain the nature of the study to the subject and answer all questions regarding this study. Prior to any study-related screening procedures being performed on the subject or any medications being discontinued by the subject in order to participate in this study, the informed consent statement will be reviewed, signed, and dated by the subject or their legally authorized representative, the person who administered the informed consent, and any other signatories according to local requirements. A copy of the signed informed consent will be given to the subject and the original will be placed in the subject's medical record. An entry must also be made in the subject's dated source documents to confirm that informed consent was obtained prior to any study-related procedures and that the subject received a signed copy.

An electronic informed consent (eConsent) system will be used in this trial. Where permitted by local regulation, study staff and participants will use electronic signature (e-signature) directly in the patient-facing tablet that is provided to the sites by the eConsent vendor. In cases where e-signature is not permitted, a print to sign option will be available. No data will be stored on the tablets; all data collected using the tablets is encrypted and kept in unalterable databases.

Information regarding benefits for subjects and information regarding provisions for treating and/or compensating subjects who are harmed as a consequence of participation in the study can be found in the informed consent form.

Optional biomarker research samples will only be collected if the subject has voluntarily signed and dated a written consent form describing the research. The written consent may be part of the main consent form. If the subject does not consent to providing optional samples, the subject will still be allowed to participate in the study.

3.2 Medical/Oncology History

A complete medical history including demographics will be taken at screening. This detailed history will include a detailed oncology history with date of diagnosis, method of diagnosis, any surgical procedures and treatments administered (including dates and type of modality and treatment results such as tumor response and severe AEs), dates and results of most recent previous disease assessments, and present signs and symptoms. The history will also include previous cardiac symptoms, cardiac diagnoses, and cardiac procedures as well as previous acute kidney injury and present signs of chronic renal failure. All medication presently used, regardless if those are prescriptions drugs or over-the-counter medications, will be documented.

On Cycle 1 Day 1, additional medical/oncology history that is observed after signing of the informed consent but prior to initial study drug administration and not considered related to study-required procedures will be recorded in the subject's medical history. This updated medical/oncology history will serve as the baseline for clinical assessment.

3.3 Concomitant Medication

On Cycle 1 Day 1, additional prior/concomitant medications taken or reported after signing of the informed consent but prior to initial study drug administration and not considered related to study-required procedures will be recorded in the subject's file.

If a subject reports taking any over-the-counter or prescription medications, vitamins, and/or other supplements or if administration of any medication becomes necessary from 4 weeks before the screening visit through 30 days after last dose of study drug, the name of the medication, dosage information including dose, route, and frequency, date(s) of administration including start and end dates, and reason for use must be recorded on the appropriate eCRF.

Subjects should receive full supportive care during study participation (except for prohibited medications listed in the Protocol Section 5.3), including but not limited to transfusion of blood products, fluid and electrolyte replacement, cytokines, antifungals, and antibiotics when appropriate as

CONFIDENTIAL INFORMATION

No use or disclosure outside AbbVie is permitted without prior written authorization from AbbVie.

treatment or as prophylaxis. Subjects who use oral contraceptives, hormone-replacement therapy, or other maintenance therapy should continue their use.

General guidelines regarding prohibited, cautionary, and allowed medications are summarized in Protocol Section 5.3 and Section 5.4.

The AbbVie TA MD identified in Section 1 may be contacted if there are any questions regarding concomitant or prior therapy(ies).

3.4 Adverse Event Assessment

Please refer to Section 4.2 and Protocol Section 6.1.

3.5 Eastern Cooperative Oncology Group Performance Status

For all subjects, the ECOG performance status will be performed as outlined in Section 2.

It is recommended, when possible, that a subject's performance status be assessed by the same person throughout the study. ECOG performance status will be assessed as follows:

Grade	Description
0	Fully active, able to carry on all predisease performance without restriction.
1	Restricted in physically strenuous activity but ambulatory and able to carry out work of a light or sedentary nature, e.g., light housework, office work.
2	Ambulatory and capable of all self-care but unable to carry out any work activities. Up and about more than 50% of waking hours.
3	Capable of only limited self-care, confined to bed or chair more than 50% of waking hours.
4	Completely disabled. Cannot carry on any self-care. Totally confined to bed or chair.
5	Dead

For all study visits after Cycle 1, ECOG performance status may be performed within 3 days before or after the scheduled visit.

3.6 Cytogenetic Testing

Cytogenetic (chromosomal) analysis may be performed centrally from diagnostic bone marrow (preferred) or from peripheral blood if adequate number of circulating blasts (> 10^9/L) are present. Historic cytogenetic data will be accepted if done within 1 month prior to screening.

Sites should perform local cytogenetics and/or fluorescence in situ hybridization analysis as per institutional guidelines.

No use or disclosure outside AbbVie is permitted without prior written authorization from AbbVie.

3.7 Pharmacokinetic Sampling

Blood and urine samples for PK will be collected for analysis of ABBV-467 concentration at the visits specified in Section 2.1.

These blood and urine samples may also be used for other exploratory analysis.

Intensive blood sampling will be performed on Cycle 1 Days 1, 2, 3, 15, 16, and 17; and sparse sampling will be performed on Day 1 of Cycles 2, 4, 6 and 8 (see Table 1).

Table 1. ABBV-467 Pharmacokinetic Blood Sampling Times

Cycle 1		Cycles 2, 4, 6, 8
**Days 1 – 3**	**Days 15 – 17**	**Day 1**
0 (pre-infusion), 10, and 20 minutes post start-of-infusion,[a] EOI, and 0.25, 0.5, 1, 2, 4, 8, 24, and 48 hours post-EOI	0 (pre-infusion), 10, and 20 minutes post start-of-infusion,[a] EOI, and 0.25, 0.5, 1, 2, 4, 8, 24 and 48 hours post-EOI	EOI

EOI = end of infusion

a. If the infusion duration is lengthened beyond 30 minutes within the study, the 10 and 20 minute post start-of-infusion samples should be increased accordingly (e.g., for a 60-minute planned infusion, the 10 and 20 minute samples should be collected at 20 and 40 minutes, respectively post start-of-infusion instead).

Note: All samples will be shipped to central laboratory for processing.

Note: Pre-infusion samples need to be collected within 1 hour prior to start of infusion. 10, and 20 minutes samples post-start of infusion need to be collected within ± 5 minutes of nominal time. End of infusion (EOI) samples need to be collected within 3 minutes prior to EOI. Samples up to 1 hour post-EOI need to be collected within ± 5 minutes of nominal time, samples > 1 hour and up to 5 hour post-EOI to be collected within ± 10 minutes of nominal time. All other samples > 5 hours post-EOI to be collected within ± 30 minutes of the nominal time.

The timing of blood collections will take priority over all other scheduled study activities except for dose administration and triplicate ECG. The date and time (to the nearest minute) of each blood sample collection will be recorded. The date and time (start time and end time) of each ABBV-467 infusion will be recorded to the nearest minute.

Urine PK samples will be taken on Cycle 1, Days 1, 2, and 3 for AML expansion cohort only (Part D; see Table 2).

Table 2. ABBV-467 AML Expansion Cohort Urine Collection Times

Cycle 1		
**Day 1**	**Day 2**	**Day 3**
0 hours (pre-infusion),[a] 0 through 8 hours post-EOI	8 through 24 hours post-EOI	24 through 48 hours post-EOI

EOI = end of infusion

a. Void sample to be collected within 1 hour prior to start-of-infusion.

Note: All samples will be shipped to central laboratory for processing.

CONFIDENTIAL INFORMATION
No use or disclosure outside AbbVie is permitted without prior written authorization from AbbVie.

The data and start and stop times (to the nearest minute) of each urine collection interval will be recorded. To ensure complete urine collection, subjects will be instructed to void into a container at the conclusion of each collection interval. For subjects with urine catheters, a fresh urine sample will be obtained. The urine sample will be thoroughly mixed, and the volume will be measured and recorded to the nearest 1 mL.

Handling/Processing of Samples

Specific instructions for collection of blood samples and subsequent preparation and storage of the plasma samples for the assay of ABBV-467 will be in the laboratory manual provided by the central laboratory.

Specific instructions for collection and storage of urine samples for the assay of ABBV-467 will be in the laboratory manual provided by the central laboratory.

Disposition of Samples

The frozen plasma and urine samples for the pharmacokinetic assays of ABBV-467 will be packed in dry ice sufficient to last during transport and transferred from the study site to the central laboratory.

The central laboratory will then ship the ABBV-467 samples to AbbVie following separately provided instructions.

Measurement Method

Plasma and urine concentrations of ABBV-467 will be determined by the Bioanalysis Department at AbbVie using validated methods. Plasma concentration of possible metabolite(s) may be determined with validated or non-validated methods.

3.8 Biomarker Research Sampling

Biospecimens (whole blood, plasma, bone marrow aspirate, and/or bone marrow core) will be collected to support the biomarker research objectives of the study. Table 3 and Table 4 provide the schedule of biomarker research sample collections for subjects with MM and AML, respectively. Assessment may include, but are not limited to, biomarkers related to the pathway (s) targeted by the study drug, or those believed to be related to the disease(s) being studied. The information learned from analyzing these samples may be used to investigate factors influencing response to treatment, scientific questions related to MM and AML, and/or in the development of new therapies and diagnostic tests.

Research performed using biomarker samples from this study will be exploratory in nature, may be conducted at non-GCP compliant laboratories, will not be provided to subjects enrolled in the study, and may not be included in the clinical study report. Additional analyses may be performed based upon data availability.

The collection of samples for the assessment of pharmacogenomics (PG) is optional. For each collection, 6.5 mL of blood will be drawn (total of 19.5 mL during the study). The results of PG research are exploratory in nature, cannot be used to make treatment decisions, and will not be shared with the study site or subjects.

CONFIDENTIAL INFORMATION
No use or disclosure outside AbbVie is permitted without prior written authorization from AbbVie.

All biomarker samples should be labeled and shipped as outlined in the study-specific laboratory manual. AbbVie (companies working with AbbVie) will store these samples in a secure storage space with adequate measures to protect confidentiality. The samples may be retained while research on ABBV-467, or drugs in these classes, or this disease and related conditions continues, but for no longer than 20 years after study completion, or per local requirement.

Biomarker sampling times, both optional and mandatory, are listed in Table 3 (MM) and Table 4 (AML).

Table 3. Sampling Times for Biomarkers - Subjects with Multiple Myeloma

Samples[a]	Biomarker	Screen	Cycle 1 Day 1	Cycle 1 Days 2 - 5	Cycle 1 Day 16	Day 1 of each cycle	Cycle 2 Day 1 and Day 1 of Every 3rd Cycle Thereafter	Final Visit[b]
**Mandatory Peripheral Blood Collection**								
Peripheral Blood	Plasma Markers	X				C4D1 and every 3 cycles thereafter		X
Peripheral Blood	Cell Processing Tube		X (predose and 4 hours postdose)					
Peripheral Blood	Flow Cytometry		X	C1D2 only	X	C2D1, C3D1, C4D1, and every 3 cycles thereafter		X
Peripheral Blood	Viable PBMC		X	C1D2 only	X	C2D1, C3D1, C4D1, and every 3 cycles thereafter		X
**Mandatory Bone Marrow Collection**								
Bone marrow aspirate	FISH	X						X
Bone marrow aspirate	Translational Research	X					C5D1 only	X

Samples[a]	Biomarker	Screen	Cycle 1 Day 1	Cycle 1 Days 2 - 5	Cycle 1 Day 16	Day 1 of each cycle	Cycle 2 Day 1 and Day 1 of Every 3rd Cycle Thereafter	Final Visit[b]
Bone marrow aspirate	MRD	X		C5D1, at Time of suspected CR, and 12, 18, and 24 months post-CR				
Bone marrow aspirate	Mutational Profiling	X					C5D1 only	X
Bone marrow biopsy	IHC (only if standard of care)	X						X[c]
Bone marrow aspirate	CD138	X					C5D1 only	X
**Optional Peripheral Blood Collection**								
Peripheral Blood	PG		Predose			Cycle 3 only		X

C = Cycle; CR = complete remission; D = Day; FISH = fluorescence in situ hybridization; IHC = immunohistochemistry; MRD = minimal residual disease; PBMC = peripheral blood mononuclear cell; PG = pharmacogenetics

a. Mandatory blood and bone marrow collections will be taken on the same day and at the same time.

b. Bone marrow aspirate and biopsy will also be collected if progressive disease is suspected.

c. Bone marrow biopsy is optional at the Final Visit for subjects with MM.

Table 4. Sampling Times for Biomarkers - Subjects with AML

Samples[a]	Biomarker	Screen	Cycle 1 Day 1	Cycle 1 Days 2 - 5	Cycle 1 Day 16	Day 1 of each cycle	Cycle 2 Day 1 and Day 1 of Every 3rd Cycle Thereafter	Final Visit[b]
**Mandatory Peripheral Blood Collection**								
Peripheral Blood	Plasma Markers	X				C4D1 and every 3 cycles thereafter		X
Peripheral Blood	Translational Research	X				X		X

CONFIDENTIAL INFORMATION

No use or disclosure outside AbbVie is permitted without prior written authorization from AbbVie.

Samples[a]	Biomarker	Screen	Cycle 1 Day 1	Cycle 1 Days 2 - 5	Cycle 1 Day 16	Day 1 of each cycle	Cycle 2 Day 1 and Day 1 of Every 3rd Cycle Thereafter	Final Visit[b]
Peripheral Blood	Flow Cytometry		X	C1D2 only	X	C2D1, C3D1, C4D1, and every 3 cycles thereafter		X
Peripheral Blood	Mutational Profiling	X				Cycle 2 only		X
Peripheral Blood	Cell Processing Tube		X (predose and 4 hours postdose)					
Peripheral Blood	Viable PBMC		X	C1D2 only	X	C2D1, C3D1, C4D1, and every 3 cycles thereafter		X
**Mandatory Bone Marrow Collection**								
Bone marrow aspirate	Mutational Profiling	X					C2D1 only	X
Bone marrow aspirate	MRD	X					X	X
Bone marrow aspirate	Translational Research	X					X	X
Bone marrow aspirate	BCL-2 family members	X					C2D1 only	X
Bone marrow biopsy	IHC (only if standard of care)	X						X
**Optional Peripheral Blood Collection**								
Peripheral Blood	PG		Predose			Cycle 3 only		X

BCL-2 = B-cell lymphoma-2; C = Cycle; D = Day; IHC = immunohistochemistry; MRD = minimal residual disease; PBMC = peripheral blood mononuclear cell; PG = pharmacogenetics

CONFIDENTIAL INFORMATION

No use or disclosure outside AbbVie is permitted without prior written authorization from AbbVie.

a. Mandatory blood and bone marrow collections will be taken on the same day and at the same time.

b. Bone marrow aspirate and biopsy will also be collected if progressive disease is suspected.

3.9 12-Lead Electrocardiogram

Single ECG

A single 12-lead ECG will be performed at the designated study visits as specified in Section 2. If triplicate ECGs are planned for the visit, then the first replicate of the triplicate ECGs may be reviewed for safety (single local ECGs are also acceptable for this purpose). The Final Visit ECG may be obtained within ± 2 days of the visit.

ECGs will be acquired after the subject has been resting in a supine or semi-recumbent position for at least 5 minutes without postural changes. The posture during ECG collection must be consistent for all subjects within a facility. Subjects will be instructed to remain completely stationary (no talking, laughing, deep breathing, sleeping, or swallowing) for approximately 10 seconds during the ECG recording. While ECGs are being acquired, subjects and staff are prohibited from having devices (e.g., cellular telephones, fans, heaters, etc.) that emit electrical interference in the room.

When an ECG is scheduled at the same time as a blood collection, the ECG will be obtained prior to the blood collection. ECGs occurring near meals will take place prior to meals. ECGs will be recorded after the subject has been in a supine or semi-recumbent position for at least 5 minutes.

The ECGs will be evaluated by an appropriately trained physician at the site ("local reader"). The local reader from the site will sign and date all ECG tracings and will provide his/her global interpretation as a written comment on the tracing using the following categories:

- Normal ECG
- Abnormal ECG – not clinically significant
- Abnormal ECG – clinically significant
- Unable to evaluate

If the global interpretation of the ECG is abnormal (NCS or CS) then the local reader will provide further information (e.g., sinus bradycardia, arrhythmia). The QTcF will be calculated and documented for all single ECGs.

Only the local reader's evaluation of the ECG will be collected and documented in the subject's source folder and eCRF. The automatic machine reading (i.e., machine-generated measurements and interpretation that are automatically printed on the ECG tracing) will not be collected.

Triplicate ECG

Twelve-lead resting ECGs in triplicate approximately 2 minutes apart are collected in ABBV-467 monotherapy dose escalation and expansion phases (Parts A, B, C, and D) at the time points specified in Table 5.

CONFIDENTIAL INFORMATION
No use or disclosure outside AbbVie is permitted without prior written authorization from AbbVie.

Table 5. ABBV-467 Triplicate Electrocardiogram Timing

Cycle 1	
**Days 1 - 2**	**Days 15 - 16**
0.5 hour pre-infusion, 0 (pre-infusion), EOI, and 0.25, 0.5, 1, 2, 4, 8, and 24 hours post-EOI	0 (pre-infusion), EOI, and 0.25, 0.5, 1, 2, 4, 8, and 24 hours post-EOI

ECG = electrocardiogram; EOI = end of infusion; PK = pharmacokinetics

Note: Triplicate ECGs will be collected in conjunction with blood sampling for PK. When occurring in conjunction, triplicate ECG can be collected up to 10 minutes prior to blood sampling and procedures performed as close to scheduled time as possible.

On the days of triplicate ECG readings, the first ECG of each triplicate may be used for the safety ECG reading (single local ECGs are also acceptable for this purpose). Any ECG collected pre-infusion on Cycle 1 Day 1 may serve as the baseline for clinical assessment. An average of the 2 pre-infusion triplicate ECGs on Cycle 1 Day 1 (0.5 hours pre-infusion; 0 hours pre-infusion) will be used as the quantitative baseline. ECGs occurring near meals or blood draws will take place prior to meals or blood draws. All electronic ECG data collected in triplicate will be transferred to and evaluated by electronic ECG/AbbVie BIOsignal System (eECG/ABBIOS), which uses an automated signal analysis algorithm to measure predefined ECG intervals (RR, PR, QT, and QRS duration). The eECG/ABBIOS analysis process includes a rigorous signal quality review using criteria that are pre-defined, objective, and evidence-based to mitigate factors that are known to introduce errors into automated ECG interval measurements. All ECGs will be transferred for manual verification and adjudication by an expert Over Reader who has experience in analyzing electronic ECG signals. The expert Over Reader will inspect each triplicate ECG and evaluate the accuracy of the interval measurements generated by eECG/ABBIOS. Based upon this manual verification and adjudication, the Over Reader will either exclude the ECG from analysis, or retain it for analysis, in which case, the Over Reader may adjust or confirm the measurements obtained by ABBIOS. The measurements obtained from the Over Reader will supersede those initially obtained by eECG/ABBIOS. The data provided by eECG/ABBIOS will be entered into the database and summarized. In addition to the Over Reader, a Central Reader who will be an appropriately qualified cardiologist will independently review each triplicate ECG to assess T and U wave morphology in eECG/ABBIOS.

Consultation with a cardiologist is recommended for any potentially concerning clinical findings, including, but not limited to, cardiac symptoms, ECG abnormalities, and/or elevated troponin levels. Investigators are also encouraged to discuss any such findings and to raise any questions/concerns on the matter with the TA MD.

The original ECG tracing and the central reader's interpretation, each with the investigator's signature and date, will be retained in the subject's records at the study site as source documents.

The data provided by eECG/ABBIOS after completion of the process detailed above will be entered into the database and summarized. Annotated electronic ECGs may be loaded in the Food and Drug Administration (FDA) ECG Warehouse after T- and U-wave morphology and interval analyses are complete.

CONFIDENTIAL INFORMATION
No use or disclosure outside AbbVie is permitted without prior written authorization from AbbVie.

3.10 Echocardiogram

Echocardiograms will be administered as specified in Section 2.

Due to high MCL-1 expression on cardiac myocytes and the potential association between ABBV-467 and cardiotoxicity, baseline and surveillance echocardiography is required for all subjects. A surveillance echocardiogram is required within approximately 7 days of Cycles 2 and 6. In addition, echocardiograms should be obtained at any time during the study for any subject presenting with any treatment-emergent signs, symptoms, or clinical findings that warrant an echocardiogram, as determined by the investigator and/or the AbbVie TA MD.

The echocardiogram findings will be documented as follows:

- normal echocardiogram
- abnormal echocardiogram – not clinically significant
- abnormal echocardiogram – clinically significant

And

- drop in ejection fraction by ≥ 10% (e.g., 65% at screening to 55% at follow-up)
- drop in ejection fraction ≤ 40%
- other clinically significant finding (please specify)

In addition to the ejection fraction classification above, the presence or absence of pericardial effusion and any other clinically significant findings will be documented at each echocardiogram obtained during study participation (including screening).

3.11 Vital Signs

Vital sign determinations of systolic and diastolic blood pressure, pulse rate, respiratory rate, and body temperature will be obtained at visits as specified in Section 2. Blood pressure and pulse rate should be measured after the subject has been sitting for at least 3 minutes and before study drug administration.

Vital signs should be assessed every 30 minutes during the ABBV-467 infusion, including prior to start, after 30 minutes of infusion, and upon conclusion of infusion but before leaving the site. Serial vital signs should be collected on all days that treatment is administered. On required visit days without ABBV-467 infusion (i.e., Final Visit), vital signs only need to be collected once.

3.12 Targeted Physical Examination

A targeted physical examination will be performed at Screening and Day 1. The examination performed on Day 1 will serve as the baseline physical examination for the entire study. Physical examination

CONFIDENTIAL INFORMATION

No use or disclosure outside AbbVie is permitted without prior written authorization from AbbVie.

abnormalities noted at the Baseline Visit prior to the first dose of study drug should be recorded in the subject's medical history.

The targeted physical exam should be based on symptoms and physical manifestations of disease and should be performed as part of the AE assessment.

Any significant physical examination findings after the first dose will be recorded as AEs. All findings, whether related to an AE or part of each subject's medical history, will be captured on the appropriate eCRF page.

For all study visits after Cycle 1, the targeted physical examination may be performed within 3 days before or after the scheduled visit.

3.13 Height and Weight

Height will be measured at screening only. Body weight will be measured at scheduled visits as specified in Section 2.1. The subject will wear lightweight clothing and no shoes during weighing.

3.14 Tumor Lysis Syndrome Prophylaxis

Subjects with MM with high tumor burden (e.g., high bone marrow plasma cell infiltration, plasma cell leukemia, or bulky plasmacytomas), rapidly increasing M-protein or light chains or high proliferative activity, plasmablastic morphology, or compromised renal function (CrCl < 50 mL/minute) may be at higher risk of developing TLS. There is a potential risk for TLS in subjects with AML, especially in those with elevated leukocyte count, circulating blasts, elevated pretreatment lactate dehydrogenase levels, renal dysfunction, and dehydration (see Protocol Section 6.2). To mitigate the risk for TLS all subjects enrolled into the study will need TLS prophylaxis and monitoring. For subjects at higher risk for TLS, additional mitigation measures with more intensive laboratory monitoring and intervention should be implemented. Prophylactic reductions of potassium, inorganic phosphorus, or uric acid above normal range are recommended prior to beginning study treatment and to be continued based on the ongoing risk of TLS.

Please reference Protocol Appendix F for additional information on the evidence of TLS and Protocol Appendix G for initial management of electrolyte abnormalities and prevention of TLS.

3.15 Clinical Laboratory Tests

A certified central laboratory will be utilized to process and provide results for the clinical laboratory tests as specified in Table 6. A certified local reference laboratory may perform laboratory testing for immediate subject management; however, split or concurrent samples must be drawn and sent to the central laboratory for analysis. Data from the central laboratory will be used for data analysis. In the case of a treatment decision made during an unscheduled visit based on results obtained from a local laboratory, the significant lab values must be collected in the eCRF. Laboratory reference ranges will be obtained prior to the initiation of the study. Other local laboratory values should not be captured in the eCRF.

CONFIDENTIAL INFORMATION
No use or disclosure outside AbbVie is permitted without prior written authorization from AbbVie.

Instructions regarding the collection, processing, and shipping of these samples will be provided by the central laboratory.

A certified local laboratory will be utilized to process and provide results for the clinical laboratory tests as specified in Table 7. Local laboratories will be utilized to process and provide results for clinical laboratory tests allowing for immediate subject medical management. Local laboratory values will be entered by the site directly onto the appropriate eCRF and laboratory normal ranges; certification for the laboratory that is used will be provided to the AbbVie clinical team.

If a laboratory test value is outside the reference range and the investigator considers the laboratory result to be clinically significant, the investigator will:

- repeat the test to verify the out-of-range value;
- follow the out-of-range value to a satisfactory clinical resolution; or
- discontinue the subject from the study
- contact the sponsor to discuss dose reductions; and
- treat the subject as appropriate.

Any laboratory result that results in drug discontinuation or treatment will be recorded as an AE. Other laboratory abnormalities might also be recorded as an AE if the investigator considers the abnormality to be relevant for the study.

CONFIDENTIAL INFORMATION
No use or disclosure outside AbbVie is permitted without prior written authorization from AbbVie.

Table 6. Central Clinical Laboratory Tests

Hematology	Clinical Chemistry	Lab Samples
Hematocrit	Blood urea nitrogen[a]	Serum β2 microglobulin
Hemoglobin	Creatinine	Creatine phosphokinase
White blood cell count	Total bilirubin	Troponin
Neutrophils	Albumin[b]	**Pharmacokinetic Samples**
Lymphocytes	Direct and indirect bilirubin	ABBV-467
Monocytes	ALT	**Biomarker Samples**
Platelet count	AST	Biomarker blood specimens
Peripheral blasts	Alkaline phosphatase	
**Myeloma Testing**	Amylase	
Serum protein immunofixation	Lipase	
Serum protein electrophoresis	Sodium	
Serum quantitative immunoglobulins	Potassium	
Serum free light chains	Calcium	
Urine protein immunofixation	Inorganic phosphorus	
Urine protein electrophoresis	Uric acid	
	Total protein	
	Glucose	
	Bicarbonate	
	Chloride	
	Creatinine clearance (Cockcroft-Gault calculation)	
	Lactate dehydrogenase[b]	

ALT = alanine aminotransferase; AST = aspartate aminotransferase

a. Urea may be reported instead of blood urea nitrogen.

b. Performed only at Screening.

Note: Myeloma testing may also be performed locally, at the discretion of the investigator.

CONFIDENTIAL INFORMATION

No use or disclosure outside AbbVie is permitted without prior written authorization from AbbVie.

Table 7. Local Clinical Laboratory Tests

Local Clinical Laboratory Tests		
**Coagulation**	**TLS Chemistry Panel**	**Other Tests**
PT/INR<br>aPTT[a]	Creatinine<br>Potassium<br>Calcium<br>Inorganic phosphorus<br>Uric acid[f]	Urine and serum pregnancy tests<br>human chorionic gonadotropin[b,c,d]<br>follicle-stimulating hormone[b,c,e]<br>Viral serologies[b,g]<br>HBsAg<br>HBcAb<br>HBsAb<br>HCV antibody (and RNA if HCV antibody is positive)
**Urinalysis**		
Leukocytes<br>Nitrite<br>Protein<br>Ketones<br>Blood<br>Glucose<br>Microscopic examination (as applicable)		

aPTT = activated partial thromboplastin time; HBsAg = hepatitis B surface antigen; HBcAb = hepatitis B core antibody; HBsAb = hepatitis B surface antibody; HCV = hepatitis C virus; INR = international normalized ratio; PT = prothrombin time; TLS = tumor lysis syndrome

a. Partial thromboplastin time (PTT) may be tested if aPTT is not locally available.

b. Performed only at screening.

c. Females only.

d. Pregnancy testing is not required for females of non-childbearing potential.

e. If needed to determine postmenopausal status.

f. At room temperature, rasburicase causes enzymatic degradation of the uric acid in blood/plasma/serum samples potentially resulting in spuriously low plasma uric acid assay readings. The following special sample handling procedure must be followed to avoid ex vivo uric acid degradation. Uric acid must be analyzed in plasma. Blood must be collected into prechilled tubes containing heparin anticoagulant. Immediately immerse plasma samples for uric acid measurement in an ice water bath. Plasma samples must be prepared by centrifugation in a precooled centrifuge (4°C). Finally, the plasma must be maintained in an ice water bath and analyzed for uric acid within 4 hours of collection.

g. Refer to Section 7.1. Japan only: Please see the Section 5.1 for additional details.

Clinical Chemistry and Hematology

The blood samples for serum hematology and chemistry tests will be collected before study drug intake as specified in Section 2; tests may be performed more often if clinically indicated. At the Day 1 visit, blood samples should be collected before the first dose of study drug. The baseline laboratory test results for clinical assessment for a particular test will be defined as the last measurement before the initial dose of study drug.

Additional hematology and chemistry laboratory assessments will be performed based on the clinical indications per institutional guidelines and regional standards. Chemistry and hematology lab results obtained within 48 hours prior to the first dose must be reviewed by the treating investigator prior to administration of that first dose. For other time points, tests may be completed ≤ 3 days before the scheduled visit and must be reviewed before dosing.

CONFIDENTIAL INFORMATION
No use or disclosure outside AbbVie is permitted without prior written authorization from AbbVie.

For chemistry labs performed for TLS prophylaxis and monitoring during Cycle 1, refer to Section 3.14, and Protocol Appendix F for specific requirements.

Tumor Lysis Syndrome Chemistry Panel

Samples for TLS chemistry will be taken at the time points specified in Table 8, while subjects are hospitalized for the first 2 doses of ABBV-467. Samples should also be obtained as clinically indicated in the opinion of the investigator.

Table 8. TLS Chemistry Panel Sampling Times

Cycle 1 Day 1	Cycle 1 Day 8	Cycle 1 Days 15 & 22
0 (pre-infusion)<br>6 – 8 hours post EOI<br>24 hours post-EOI (on Day 2)	0 (pre-infusion)<br>6 – 8 hours post EOI<br>24 hours post-EOI (on Day 9)	0 (pre-infusion)

EOI = end of infusion

Creatine Phosphokinase and Troponin Enzymes

Blood samples will be collected on the days specified in Section 2 for analysis of creatine phosphokinase and troponin serum concentrations. Clinical significance of these enzymes will be evaluated within the context of the study. The sampling times are listed in Table 9. Samples should also be obtained as clinically indicated in the opinion of the investigator.

Table 9. Creatine Phosphokinase and Troponin Sampling Times

Cycle 1		Cycles 2 - 8	Cycles 2, 4, 6, 8
Days 1 – 3	Days 15 – 17	Day 1	Day 15
0 (pre-infusion)<br>4, 8, 24, and 48 hours post-EOI	0 (pre-infusion)<br>4, 8, 24 and 48 hours post-EOI	EOI	EOI

EOI = end of infusion

Note: All samples will be shipped to central lab for processing. Sample times are relative to ABBV-467 infusion.

Pregnancy Tests (Serum and Urine)

Women of childbearing potential must have a negative pregnancy test before initiating therapy. A pregnant or breastfeeding female will not be eligible for participation or continuation in this study. Pregnancy testing should not be performed for postmenopausal women; determination of postmenopausal status will be made during the screening period based on the subject's history.

A qualitative serum pregnancy test will be performed at Screening and a urine pregnancy test will be performed at baseline (or upon confinement in each period) for all female subjects of childbearing potential.

If the serum pregnancy test is positive the subject is considered a screen failure. If the serum pregnancy test is borderline, it should be repeated 2 days later to determine eligibility.

CONFIDENTIAL INFORMATION
No use or disclosure outside AbbVie is permitted without prior written authorization from AbbVie.

If the repeat serum pregnancy test is:

- Positive, the subject is considered a screen failure;
- Negative, the subject can be enrolled into the trial;
- Still borderline, the AbbVie TA MD will be consulted.

Additional urine pregnancy tests will be performed at visits indicated in the Activities Schedule and Section 2. More frequent pregnancy tests can be performed throughout the study at the investigator's discretion or if required per local/country requirements. Urine pregnancy test must be obtained at Cycle 1 Day 1, if it has been > 7 days since obtaining the serum pregnancy results at screening.

A pregnancy test should be repeated on Day 1 of each cycle, evaluated prior to dosing, and at Final Visit.

If the urine pregnancy test (which is performed at the site) is negative, begin or continue dosing. If urine pregnancy test is positive subjects must discontinue from the study.

Coagulation

Prothrombin time (PT) or international normalized ratio (INR) and activated partial thromboplastin time (aPTT) samples will be collected at visits indicated in Section 2. Coagulation panel should be repeated on Day 1 of each cycle for subjects taking vitamin K antagonists or if otherwise clinically indicated.

Urinalysis

Urinalysis tests will be performed as specified in Section 2. Dipstick urinalysis will be completed by the local laboratory at all required visits. Specified abnormal macroscopic urinalyses defined as leukocytes, nitrite, protein, ketones, or blood greater than negative, or glucose greater than normal should be followed up with a microscopic analysis (red blood cells, white blood cells, epithelial cells, bacteria, yeast, parasites, casts, and crystals) at the local laboratory. Urinalysis data may be collected if associated with an AE.

3.16 Disease Assessments

Multiple Myeloma Assessments

Disease assessments will be performed at the visits indicated in Section 2. MM subjects will be evaluated using the adapted International Myeloma Working Group (IMWG) 2016 criteria (see Section 7.3). Note that serum and urine for IMWG assessments do not need to be collected at C1D1 if the Screening samples were collected within 7 days of C1D1.

All subject's IMWG laboratory assessments (serum protein electrophoresis [SPEP], urine protein electrophoresis [UPEP], serum and urine immunofixation, serum free light chains, and serum quantitative immunoglobulins) will be performed by central laboratory, however, local labs may be performed at the investigator's discretion. All local laboratory results must be entered into the eCRF. Imaging and bone marrow assessments will be performed locally. Disease status should continue to be collected for MM subjects after study drug discontinuation for reasons other than disease progression.

No use or disclosure outside AbbVie is permitted without prior written authorization from AbbVie.

abbvie

Serum Protein Electrophoresis, Serum Protein Immunofixation, Serum Quantitative Immunoglobulins

Peripheral blood samples for SPEP, serum protein immunofixation, and serum quantitative immunoglobulin testing will be collected as outlined in Section 2. Serum protein electrophoresis and serum immunofixation will be collected for all subjects at baseline and throughout the study until progressive disease or withdrawal of consent, regardless of SPEP being measurable or M-protein presence at baseline.

The assessment of SPEP M-protein and serum immunofixation at the time of possible VGPR or CR/sCR is mandatory, even in subjects without measurable values at baseline.

Serum Free Light Chains

Blood samples for serum free light chain (FLC) testing will be collected for all subjects as outlined in Section 2.

Subjects with measurable disease in either SPEP and/or UPEP will be assessed for response only based on these 2 tests and not by the FLC assay. FLC response criteria are only applicable to fulfill the requirements of sCR or CR per IMWG criteria.

Urine Protein Immunofixation and Urine Protein Electrophoresis, 24-Hour Urine

Urine samples (24-hour) for urine protein immunofixation and UPEP for M protein testing will be collected for all subjects as outlined in Section 2. Urine protein electrophoresis and urine immunofixation will be collected for all subjects at baseline and throughout the study until progressive disease or withdrawal of consent, regardless of UPEP being measurable or M-protein presence at baseline.

The assessment of UPEP M-protein and urine immunofixation at the time of possible VGPR or CR/sCR is mandatory, even in subjects without measurable values at baseline.

Subjects with measurable disease at baseline by SPEP and UPEP must be followed by both SPEP and UPEP assessment of M-protein for response assessment.

Skeletal Survey

Whole body skeletal imaging will be done at screening using either conventional radiography, low-dose whole body computed tomography (CT), positive emission tomography CT component only (PET-CT), or whole-body magnetic resonance imaging (MRI). The methodology used at baseline should be used at all the follow-up time points for consistent evaluation of any lesions identified. Historical skeletal survey results obtained within 30 days before the first dose may be used for screening (using the above techniques). A skeletal survey will be comprised of the following:

- lateral radiograph of skull.
- antero-posterior and lateral views of the spine.
- antero-posterior views of pelvis, ribs, femora, tibiae, fibulae, humeri, ulnae, and radii.

CONFIDENTIAL INFORMATION
No use or disclosure outside AbbVie is permitted without prior written authorization from AbbVie.

Skeletal surveys should be completed as outlined in Section 2. After the screening procedure, skeletal survey should only be performed if clinically indicated. While the subject is on study, survey (if done) should record any changes to the number of or size of lytic lesions as well as the number and location of any new skeletal or lytic lesions. Changes in measurable lesions over the course of therapy will be assessed using IMWG criteria may be performed at any time during the study if clinically indicated per the investigator.

Plasmacytoma Evaluation

CT, MRI, or PET-CT scans should be performed at baseline, if clinically indicated, to assess for presence of extramedullary plasmacytoma. Plasmacytoma evaluation should be performed to confirm PR/CR or progressive disease, or otherwise as clinically indicated, every 12 - 16 weeks. The same radiological method should be used throughout the study.

Bone Marrow Aspirate and Biopsy

Bone marrow aspirate and biopsy should be completed as outlined in Section 2. Bone marrow aspirates and biopsies performed as standard of care throughout the study should also be captured on an eCRF.

A sufficient bone marrow aspirate or biopsy must be collected for clinical assessment (pathology, plasma cell % and kappa (κ)/lambda (λ) for clonality per IMWG criteria) and cytogenetics performed by a local laboratory as well as for shipment of a portion for the biomarker analyses (refer to Section 3.8). Bone marrow biopsy and aspirate specimens for biomarker analyses are to be split from those collected for locally performed IMWG disease assessments; adequate samples should be obtained to allow for biomarker analysis.

A bone marrow aspirate collection is mandatory at screening, to confirm sCR/CR (including MRD assessments), at 12, 18, and 24 months post confirmation of sCR/CR, and at the Final Visit. Additionally, should an aspirate be performed at any time to confirm a CR or when one is suspected, then an aliquot for biomarker studies should be provided.

Bone marrow core biopsy (fixed formalin paraffin embedded [FFPE] core) should be also collected, unless not recommended per institutional guidelines. However, a bone marrow biopsy is mandatory in the circumstance that an aspirate sample is not available (e.g., due to a dry tap). Fresh biopsies are preferred but archived tissue is acceptable if representative of current disease. Core block or tissue slides are acceptable. Bone marrow core biopsies are optional to confirm sCR/CR, or at the Final Visit.

De-identified copies of all bone marrow biopsy and aspirate reports must be provided to the sponsor and may be submitted for central review to confirm response.

Acute Myeloid Leukemia Assessments

Assessments of AML disease status by the modified International Working Group (IWG) criteria (see Section 7.4) will be performed at the visits indicated in Section 2.

All AML subjects will have response assessment according to the modified IWG criteria for AML. Progressive disease is defined per European LeukemiaNet recommendations. If additional treatments are needed to optimize subjects' medical care, they can be performed following institutional standards and procedures. Subject's disease assessment is based on the most recent physical examination, bone

No use or disclosure outside AbbVie is permitted without prior written authorization from AbbVie.

marrow results and recent hematology values. For subjects who require a delay in next cycle of study treatment for blood count recovery after a bone marrow evaluation, hematology values for up to 2 weeks or pre-dose labs from Day 1 of the next cycle can be used to determine the IWG response.

All AML subjects who completed at least 1 cycle of study treatment will be assessed by the investigators using the modified IWG criteria for AML as described in Section 7.4. Subjects who have discontinued study treatment prior to completion of Cycle 1 will be deemed non-evaluable for response assessment. In addition to the response assessment using the above response criteria, each subject will also be evaluated for complete response with partial hematologic recovery (CRh) based on the hematologic parameters.

Bone marrow aspirate and biopsy should be completed as outlined in Section 2. Bone marrow aspirates and biopsies performed as standard of care throughout the study should also be captured on an eCRF.

A bone marrow aspirate and biopsy must be performed for all subjects during screening to collect mandatory samples for disease and biomarker assessments. Bone marrow aspirate and biopsy will also be collected anytime that progressive disease is suspected.

Bone marrow biopsy materials (i.e., block or slides) are to be submitted from screening and final visits, and aspirate is to be provided from screening, C2D1, Day 1 of every 3 Cycles thereafter (i.e., C5D1, C8D1, C11D1, etc.), and the Final Visit. Additionally, should an aspirate be performed at any time to confirm a CR or when one is suspected, then an aliquot for biomarker studies should be provided.

Bone marrow aspirate and biopsy samples must be collected for all subjects for each of the disease assessments. Bone marrow core biopsy sample collection is considered an optional procedure only for subjects enrolled at sites in countries where an aspirate evaluation by morphologic assessment and flow cytometry is considered standard of care. For these subjects if the aspirate sample is inadequate or unevaluable for disease assessment, repeat bone marrow aspirate and biopsy must be performed within 7 days. A corresponding local laboratory pathology report should be sent to the central laboratory for each local disease assessment which is conducted.

The C2D1 bone marrow aspirate and biopsy should be performed within ± 3 days of Cycle 1 Day 28 and reviewed prior to the administration of study drugs for Cycle 2, if possible. For subjects who require a delay in study treatment for blood count recovery after a bone marrow evaluation, local or central laboratory values obtained up to 2 weeks after C2D1 (i.e., C2D15) can be used to determine the response. Subsequent response assessment should be conducted after every 3 cycles (± 1 week; after C4, C7, etc.) and upon any clinical suspicion of relapse. Although AML response assessments should be completed as soon as possible at the end of relevant cycles, the subsequent cycle does not have to be delayed for bone marrow aspirate/biopsy results that are not yet available. For subjects with resistant disease after the first cycle, and who are remaining on study, a repeat bone marrow aspirate/biopsy should be performed after the second cycle in order to confirm and/or clarify response.

Bone marrow biopsy and aspirate specimens for biomarker analyses are to be split from those collected for locally performed disease assessment; adequate samples should be obtained to allow for biomarker analysis. De-identified copies of all bone marrow biopsy and aspirate reports must be provided to the sponsor and may be submitted for central review to confirm response.

Disease status should continue to be collected for AML subjects after study drug discontinuation for reasons other than disease progression.

3.17 Hospitalization/Inpatient Monitoring

During dose escalation, each subject should be hospitalized the nights prior to C1D1 and C1D8 for hydration, treatment with uric acid-reducing agents, and monitoring. The subject may be discharged once TLS labs are reviewed 24 hours after dosing. See Protocol Section 6.2 for further detail.

Subjects in Japan will be hospitalized for the duration of the DLT period (see Section 5.1).

3.18 Dispense Study Drug

ABBV-467 will not be directly dispensed to subjects. Study drugs will be administered to subjects beginning at baseline (Day 1) and as specified in Section 2.1. The first dose of study drugs will be administered after all other baseline (Day 1) procedures are completed.

Each site will be responsible for maintaining drug accountability records including product description, manufacturer, and lot numbers for all non-investigational products dispensed by the site.

The first dose of ABBV-467 on C1D1 will be administered at half the targeted dose for a given subject; subsequent doses will be administered at the full target dose. ABBV-467 will be administered weekly, for each 28-day cycle, over at least a 30-minute period at approximately the same time each day, with or without food. Alternative dose schedules and infusion times may be explored pending adequate accumulation and thorough review (including with investigators) of clinical safety and PK data.

3.19 Subject Withdrawal from Study

All attempts must be made to determine the date of the last study drug dose and the primary reason for discontinuation of study drug or study participation. The information will be recorded on the appropriate eCRF page. However, these procedures should not interfere with the initiation of any new treatments or therapeutic modalities that the investigator feels are necessary to treat the subject's condition. Following discontinuation of study drug, the subject will be treated in accordance with the investigator's best clinical judgment, irrespective of whether or not the subject decides to continue participation in the study.

4 SAFETY MANUAL

4.1 Methods and Timing of Safety Assessment

All serious and nonserious AEs which could be related to study procedures (e.g., occurrence during screening) will be collected from the time the subject signed the study-specific informed consent until study drug administration and reported to the sponsor. From the time of study drug administration until 30 days after discontinuation of study treatment, all AES and SAEs will be collected whether solicited or spontaneously reported by the subject.

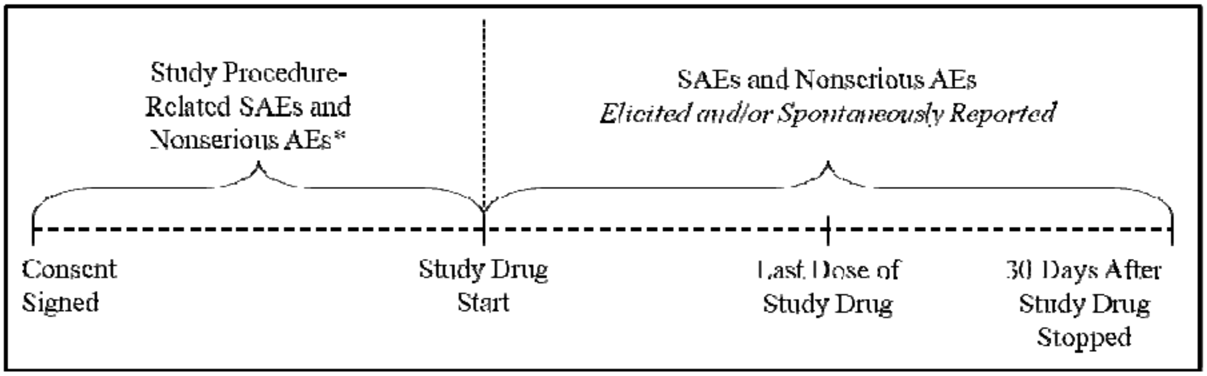

AE = adverse event; SAE = serious adverse event

4.2 Recording Data and Analyses of Safety Findings

Adverse events will be coded using MedDRA. The number and percentage of subjects with treatment-emergent adverse events (i.e., any event that begins or worsens in severity after initiation of study drug through 30 days post-study drug dosing) will be tabulated by primary MedDRA SOC and preferred term and compared between arms using Fisher's exact test. The tabulation of the number of subjects with treatment-emergent AEs by severity grade and relationship to study drug also will be provided. Subjects reporting more than 1 AE for a given MedDRA preferred term will be counted only once for that term using the most severe grade according to the severity grade table and the most related according to the relationship to study drug tables. Subjects reporting more than 1 type of event within an SOC will be counted only once for that SOC.

4.3 Reporting Adverse Events and Intercurrent Illnesses

In the event of an SAE, whether associated with study drug or not, the investigator will notify Clinical Pharmacovigilance within 24 hours of the site being made aware of the SAE by entering the SAE data into the electronic data capture system. SAEs that occur prior to the site having access to the RAVE® system, or if RAVE is not operable, should be documented on the SAE non-eCRF and emailed (preferred route) or faxed to Clinical Pharmacovigilance within 24 hours of the site being made aware of the SAE.

Email: PPDINDPharmacovigilance@abbvie.com<br>FAX to: +1 (847) 938-0660

For safety concerns, contact the Oncology Safety Team at:

AbbVie Oncology Safety Team, Bldg. AP30-3
1 North Waukegan Road
North Chicago, Illinois 60064
Office: (847) 935-2609
Email: SafetyManagement_Oncology@abbvie.com

For any subject safety concerns, please contact the physician listed below:

Primary Therapeutic Area Medical Director
EMERGENCY MEDICAL CONTACT:
[redacted]

[redacted]
1 North Waukegan Road
North Chicago, IL 60064

Contact Information:

Office: [redacted]
Mobile:
Email:

In emergency situations involving study subjects when the primary Therapeutic Area Medical Director is not available by phone, please contact the 24-hour AbbVie Medical Escalation Hotline where your call will be re-directed to a designated backup AbbVie Therapeutic Area Medical Director:

HOTLINE: +1 (973) 784-6402

The sponsor will be responsible for Suspected Unexpected Serious Adverse Reactions (SUSAR) reporting for the Investigational Medicinal Product (IMP) in accordance with Directive 2001/20/EC.

5 COUNTRY-SPECIFIC REQUIREMENTS

5.1 Japan-Specific Information

Study Design:

Special Populations

At least 1 Japanese subject will be enrolled at each dose level in dose escalation Parts A and C. Subjects from sites in Japan will be enrolled sequentially, and will be incorporated into the BOIN dose escalation decisions as they become DLT-evaluable. In Parts B and D (dose expansion), at least 3 Japanese subjects are planned to be enrolled.

CONFIDENTIAL INFORMATION
No use or disclosure outside AbbVie is permitted without prior written authorization from AbbVie.

Hepatitis B virus (HBV) testing

In Japan, if hepatitis B surface antigen (HBsAg) is negative at screening, hepatitis B surface antibody (HBsAb) and hepatitis B core antibody (HBcAb) should be tested to ensure the safety of the subject. If HBsAb and/or HBcAb is positive and hepatitis B virus (HBV) DNA value is < 2.0 IU/mL, monitoring should be conducted following monitoring method described in Figure 1.

Figure 1. Guidelines for the Prevention of Hepatitis B Virus Reactivation in Subjects Receiving Immunosuppressive Therapy or Chemotherapy

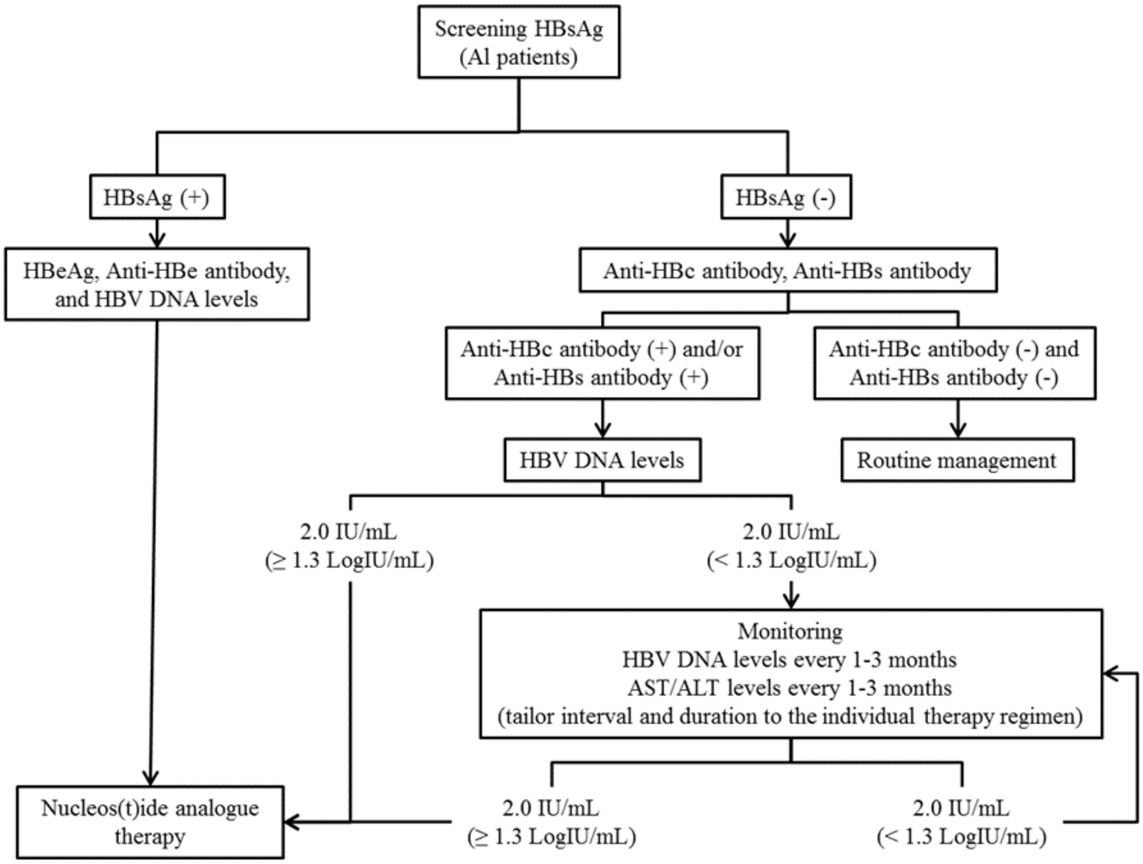

ALT = alanine aminotransferase; AST = aspartate aminotransferase; HBc = hepatitis B core; HBs = hepatitis B surface; HBsAg = hepatitis B surface antigen; HBV = hepatitis B virus; NA = nucleos(t)ide analog

Addendum: Caution is required when administering powerful chemotherapeutic agents for hematological malignancies, as during or following completion of treatment some HBsAg positive or negative patients will develop hepatitis B due to reactivation of HBV, and some of these will go on to suffer fulminant hepatitis. Consideration should also be given to the possibility of HBV reactivation in association with standard chemotherapy for hematological malignancies or solid cancers, and immunosuppressive therapy for autoimmune diseases, such as rheumatic and collagen diseases. The incidences of HBV reactivation, hepatitis and fulminant hepatitis associated with standard chemotherapy and immunosuppressive therapy are not known, and there is a lack of evidence on which to base guidelines. Furthermore, prevention of fulminant hepatitis is not guaranteed with NA therapy.

NB 1) HBV carriers and patients with resolved hepatitis B should be screened prior to immunosuppressive therapy or chemotherapy. First HBsAg testing should be performed to determine whether they are an HBV carrier. HBsAg negative patients should be tested for anti-HBc antibody and anti-HBs antibody, to confirm past infection. Highly sensitive testing

CONFIDENTIAL INFORMATION
No use or disclosure outside AbbVie is permitted without prior written authorization from AbbVie.

methods should be used for measurements of HBsAg, anti-HBc antibody and anti-HBs antibody. Even for only anti-HBs antibody positive patients (i.e., HBsAg negative and anti-HBc antibody negative), reactivation of HBV has been reported, so the action should be taken in accordance with the guidelines unless there is evidence of prior vaccination to HBV.

NB 2) A hepatologist should be consulted concerning HBsAg positive patients. A hepatologist should preferably be consulted for all patients administered NAs.

NB 3) In some patients undergoing retreatment who did not undergo testing for anti-HBc or HBs antibody at the time of their initial chemotherapy, and in patients who have already commenced immunosuppressive therapy, antibody titers may be low, in which case measurement of HBV DNA levels is preferable.

NB 4) Patients with resolved HBV infection should be screened using real-time PCR measurement of HBV DNA levels.

NB 5)

a. Caution is required when treating patients with resolved HBV infection with rituximab + corticosteroid or fludarabine chemotherapy, or when they undergo hematopoietic stem cell transplantation, as these patients are at high risk of HBV reactivation. HBV DNA levels should be monitored on a monthly basis during treatment, and for at least 12 months afterward. Long-term monitoring is required for hematopoietic stem cell transplant recipients.
b. Although the incidence is low, there is a risk of HBV reactivation with standard chemotherapy regimens and combination with molecular targeted therapy with immune-activity. HBV DNA levels should be measured every 1 – 3 months, with the interval and duration tailored to the individual therapy regimen. It is best to err on the side of caution with patients undergoing treatment for hematological malignancies.
c. There is also a risk of HBV reactivation associated with immunosuppressive therapy using corticosteroids, immunosuppressant agents, or molecular targeted therapy with immunosuppressant or immunomodulator activity. HBV DNA levels should be monitored on a monthly basis in patients on immunosuppressive therapy for at least 6 months after commencement or alteration (including cessation) of treatment. After 6 months, the interval and duration should be tailored to the individual therapy regimen.

NB 6) Administration should be commenced as soon as possible, before commencement of immunosuppressive therapy or chemotherapy. However, for HBsAg positive patients with high HBV load, it should be reduced prior to initiation of immunosuppressive therapy or chemotherapy, because fatal cases have been reported due to fulminant hepatitis even on NA therapy.

NB 7) Administration should be commenced as soon as the HBV DNA levels exceed 2.1 log copies/mL, during or after immunosuppressive therapy or chemotherapy. If this occurs during treatment, it is preferable to consult with a hepatologist, and not immediately cease the immunosuppressant or antineoplastic agent with immunosuppressive activity.

NB 8) Entecavir is the recommended NA.

NB 9) Cessation of NA therapy can be considered if the following criteria are met. In patients who were HBsAg positive at the time of screening, when the criteria for cessation of NA therapy in cases with chronic hepatitis B are met. In patients who were anti-HBc antibody and/or anti-HBs antibody positive at the time of screening:

(1) NA therapy has been continued for at least 12 months after completion of immunosuppressive therapy or chemotherapy.
(2) ALT (GPT) levels have been normalized during this period (excluding causes of elevated ALT levels other than HBV).
(3) negative conversion of HBV DNA has occurred during this period.

NB 10) Patients should be carefully monitored, including measurement of HBV DNA levels, for at least 12 months following completion of NA therapy. Monitoring methods depend on package inserts of each NA. NA therapy should be immediately resumed if HBV-DNA levels exceed 2.1 log copies/mL during monitoring period.

Cross reference: Drafting Committee for Hepatitis Management Guidelines and the Japan Society of Hepatology.

Safety reporting

In Japan, the principal investigator will provide documentation of all SAEs to the Director of the investigative site and the Sponsor.

No use or disclosure outside AbbVie is permitted without prior written authorization from AbbVie.

Clinical Expense and Compensation:

Expenditure of the Clinical Expense

The Sponsor will pay the expenses related to this study to the investigative site in accordance with "Special Healthcare Expenditure." The expenses of screening test, etc., will be paid based on the contract concluded with each investigative site. To lighten the burden imposed on the subject with participation to the study, transportation expenses, etc., will be paid to the subjects via participating investigative site in accordance with the rules of the investigative site.

Compensation for Health Impairment and Insurance

1. If a subject suffers some sort of health impairment due to this study, the investigative site will provide treatment and take other necessary measures. Among the expenses required for the treatment, the amount not covered by health insurance that the subject must pay directly will be borne by the Sponsor only when the event is associated with the use of the study drug.
2. When a subject suffers health impairment during this study and a dispute occurs or might occur between the investigative site and the subject, the investigative site will report it to the Sponsors immediately and resolve it. The Sponsor will cooperate with the investigative site in resolving the problem.
3. When the investigative site must compensate to the subject's health impairment caused by this study, the compensation paid by the investigative site and the expenses related to any dispute will be borne in full by the Sponsor, except in cases where the responsibility for the problem is attributed to the investigative site. This shall not apply to cases where the health impairment occurred because the investigative site performed the study with marked deviation from the GCP or the protocol or because of a deliberate action or a major error by the investigative site.
4. When a subject suffers health impairment during this study and liability for compensation arises, the Sponsor will compensate in accordance with the standard operating procedure regarding the compensation prepared in advance.
5. The Sponsor will obtain clinical study insurance and will take other necessary measures to cover the claims and compensation required in such cases.

Consent

Written consent is necessary both from the subject and his/her legal representative if he/she is under the age of 20 years.

If a Japanese subject under the age of 20 years has reached 20 years while participating in the study, new written consent is necessary from the subject regarding continued participation in the study.

Confinement

In Japan, the subjects in Dose Escalation will be hospitalized during the DLT evaluation period for the purpose of safety management.

Protocol Deviations

Investigators must record all protocol deviations in the appropriate medical records. The principal investigator must report protocol deviations which were necessary to eliminate an immediate hazard to

CONFIDENTIAL INFORMATION
No use or disclosure outside AbbVie is permitted without prior written authorization from AbbVie.

study subjects, attaching the reasons for protocol deviations, to both AbbVie and the head of medical institution.

Contraception

- Combined (estrogen and progestogen containing) hormonal birth control (oral, intravaginal,* transdermal,* injectable) associated with inhibition of ovulation initiated at least 1 month prior to study Baseline Day 1.
- Progestogen-only hormonal birth control (oral, injectable,* implantable*) associated with inhibition of ovulation initiated at least 1 month prior to study Baseline Day 1.

* Not approved in Japan.

5.2 SUSAR Reporting

AbbVie will be responsible for SUSAR reporting for the IMP in accordance with global and local guidelines and Appendix A of the Investigator Brochure will serve as the Reference Safety Information (RSI). The RSI in effect at the start of a DSUR reporting period serves as the RSI during the reporting period. For follow-up reports, the RSI in place at the time of occurrence of the 'suspected' Serious Adverse Reaction will be used to assess expectedness.

6 STUDY DRUG

6.1 Treatments Administered

ABBV-467 will be administered as an IV infusion once weekly for each 28-day cycle over at least a 30-minute period, as listed in Section 2.1. Various doses will be administered, as determined by dose-escalation rules. ABBV-467 will be provided by AbbVie as a solution in a glass vial. The vial will contain [redacted] of ABBV-467 (refer to Protocol Section 5.7 for study drug information). After preparation of the dose for infusion and during infusion, the solution must be protected from exposure to light.

Study drug must not be dispensed without contacting the IRT system. Study drug may only be dispensed to subjects enrolled in the study through the IRT system. At the end of the Treatment Period or at the Final Visit, the site will contact the IRT system to provide visit date information and study drug return information for each kit.

6.2 Packaging and Labeling

ABBV-467 will be packaged in vials with quantities sufficient to accommodate study design. Each kit will be labeled per local requirements and this label must remain affixed to the kit. All blank spaces on the labels should be completed by site staff prior to dispensing to subject.

Storage and Disposition of Study Drug

ABBV-467 must be shipped and stored at controlled temperature (–25° to –10°C) and protected from light.

CONFIDENTIAL INFORMATION
No use or disclosure outside AbbVie is permitted without prior written authorization from AbbVie.

The investigational products are for investigational use only and are to be used only within the context of this study. The study drug supplied for this study must be maintained under adequate security and stored under the conditions specified on the label until dispensed for subject use or destroyed on site as appropriate.

6.3 Method of Assigning Subjects to Treatment Groups

This is an open-label, dose-escalation and dose-expansion study. At the screening visit, all subjects will be assigned a unique identification number through the use of the IRT system. For subjects who do not meet the study selection criteria, the site personnel must contact the IRT system and identify the subject as a screen failure.

Subjects who are enrolled will retain their identification number assigned at the screening visit throughout the study. Upon receipt of study drug, the site will acknowledge receipt in the IRT system.

Contact information and user guidelines for IRT use will be provided to each site.

6.4 Selection and Timing of Dose for Each Subject

Selection of the doses for this study is discussed in Protocol Section 4.2. All subjects should take all doses of study medications as detailed in Section 6.1. ABBV-467 will be administered on the designated study days as specified in Section 2.1. The study drug information is provided in Protocol Section 5.7.

6.5 Preparation/Reconsitution of Dosage Form

Written instructions for the preparation of ABBV-467 solutions for infusion will be provided as a separate document from the protocol.

CONFIDENTIAL INFORMATION
No use or disclosure outside AbbVie is permitted without prior written authorization from AbbVie.

7 Appendices

7.1 HEPATITIS B VIRUS TESTING GUIDELINES FOR ELIGIBILTY

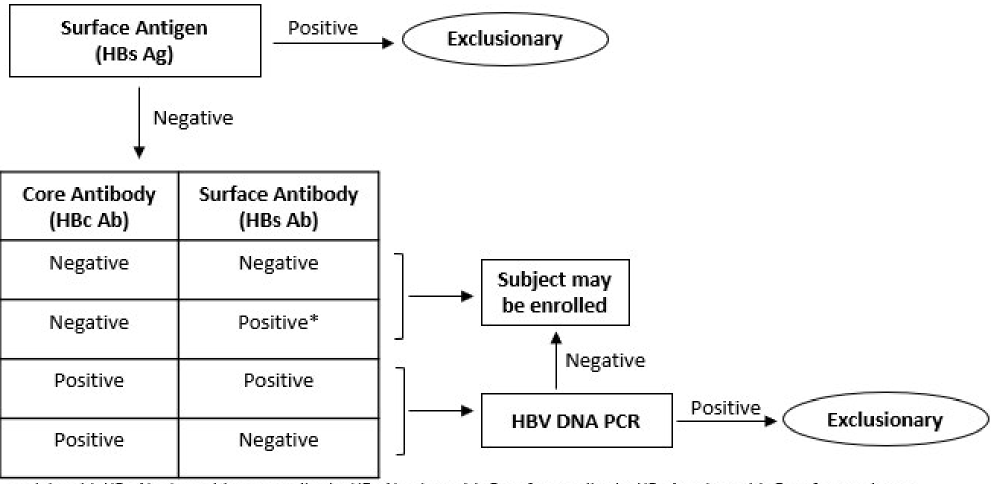

Scenario	Core Antibody (HBc Ab)	Surface Antibody (HBs Ab)
A	Negative	Negative
B	Negative	Positive*
C	Positive	Positive
D	Positive	Negative

DNA= deoxyribonucleic acid; HBc Ab= hepatitis core antibody; HBs Ab = hepatitis B surface antibody; HBs Ag = hepatitis B surface antigen; HBV = hepatitis B virus; PCR = polymerase chain reaction

* A positive test result for HBs Ab is expected for subjects who have had an HBV vaccination. For subjects without a history of HBV vaccination (and where mandated by local requirements), a positive result for HBs Ab requires HBV DNA PCR testing.

7.2 NATIONAL COMPREHENSIVE CANCER NETWORK RISK CATEGORIZATION: GUIDELINES FOR AML (Version 1.2019)

**Risk Category**	**Cytogenetics**
Favorable Risk	Core binding factor: inv(16) or t(16;16) or t(8;21)<br>t(15;17)
Intermediate Risk	Normal cytogenetics<br>+8 alone<br>t(9;11)<br>Other non-defined
Poor Risk	Complex (≥ 3 clonal chromosomal abnormalities)<br>Monosomal karyotype<br>-5,5q-,-7,7q-<br>11q23-non t(9;11)<br>inv(3),t(3;3)<br>t(6;9)<br>t(9;22)

CONFIDENTIAL INFORMATION
No use or disclosure outside AbbVie is permitted without prior written authorization from AbbVie.

7.3 INTERNATIONAL MYELOMA WORKING GROUP RESPONSE CRITERIA

Category	IMWG Response Criteria[a]
Stringent complete response (sCR)	Complete response as defined below, plus the following:<br>• Normal FLC ratio<br>• Absence of clonal cells in bone marrow by immunohistochemistry[b]
Complete response (CR)	• Negative immunofixation on the serum and urine<br>• < 5% plasma cells in bone marrow aspirates<br>• Disappearance of any soft tissue plasmacytomas
Very good partial response (VGPR)	• Serum and urine M-protein detectable by immunofixation but not on electrophoresis, or<br>• ≥ 90% reduction in serum M-protein plus urine M-protein level < 100 mg per 24 hours
Partial response (PR)	• ≥ 50% reduction of serum M-protein plus reduction in 24 hours urinary M-protein by ≥ 90% or to < 200 mg per 24 hours.<br>• If the serum and urine M-protein are not measurable, a ≥ 50% decrease in the difference between involved and uninvolved FLC levels is required in place of the M-protein criteria.<br>• If serum and urine M-protein are not measurable, and serum-free light assay is also not measurable, ≥ 50% reduction in plasma cells is required in place of M-protein, provided baseline bone marrow plasma-cell percentage was ≥ 30%.<br>• In addition to the above criteria, if present at baseline, a ≥ 50% reduction in the size (SPD) of soft tissue plasmacytomas is also required.
Minimal response (MR)	• ≥ 25% but ≤ 49% reduction of serum M-protein and reduction in 24-hour urine M-protein by 50% to 89%.<br>• In addition to the above listed criteria, if present at baseline, a ≥ 50% reduction in the size (SPD) of soft tissue plasmacytomas is also required.
Stable disease (SD)	• Not meeting criteria for CR, VGPR, PR, MR, or progressive disease

CONFIDENTIAL INFORMATION
No use or disclosure outside AbbVie is permitted without prior written authorization from AbbVie.

Category	IMWG Response Criteria[a]
Progressive disease[c]	Any 1 or more of the following criteria:<br>• Increase of 25% from lowest confirmed response value in 1 or more of the following criteria:[d]<br>◦ Serum M-protein (absolute increase must be ≥ 0.5 g/dL);<br>◦ Serum M-protein increase ≥ 1 g/dL, if the lowest M component was ≥ 5 g/dL;<br>◦ Urine M-protein (absolute increase must be ≥ 200 mg/24 hours);<br>◦ In subjects without measurable serum and urine M-protein levels, the difference between involved and uninvolved FLC levels (absolute increase must be > 10 mg/dL);<br>◦ In subjects without measurable serum and urine M-protein levels and without measurable involved FLC levels, bone marrow plasma-cell percentage irrespective of baseline status (absolute increase must be ≥ 10%);<br>• Appearance of a new lesion(s), ≥ 50% increase from nadir in SPD of > 1 lesion, or ≥ 50% increase in the longest diameter of a previous lesion > 1 cm in short axis;<br>• ≥ 50% increase in circulating plasma cells (minimum of 200 cells per µL) if this is the only measure of disease
Clinical Relapse	• Clinical relapse requires 1 or more of the following criteria:<br>◦ Direct indicators of increasing disease and/or end organ dysfunction (CRAB features) related to the underlying clonal plasma-cell proliferative disorder;<br>◦ Development of new soft tissue plasmacytomas or bone lesions (osteoporotic fractures do not constitute progression);<br>◦ Definite increase in the size of existing plasmacytomas or bone lesions. A definite increase is defined as a 50% (and ≥ 1 cm) increase as measured serially by the SPD of the measurable lesion;<br>◦ Hypercalcemia (> 11 mg/dL);<br>◦ Decrease in hemoglobin of ≥ 2 g/dL not related to therapy or other non-myeloma-related conditions;<br>◦ Rise in serum creatinine by 2 mg/dL or more from the start of the therapy and attributable to myeloma;<br>◦ Hyperviscosity related to serum paraprotein
**MRD Criteria (requires a complete response, as defined above)**	
Sustained MRD-negative	MRD negativity in the marrow (NGF or NGS, or both) and by imaging, confirmed minimum of 1 year apart. Subsequent evaluations can be used to further specify the duration of negativity (e.g., MRD-negative at 5 years)[e]
Flow MRD-negative	Absence of phenotypically aberrant clonal plasma cells by NGF[f] on bone marrow aspirates using the EuroFlow standard operation procedure for MRD detection in multiple myeloma (or validated equivalent method) with a minimum sensitivity of 1 in 10^5 nucleated cells or higher

CONFIDENTIAL INFORMATION
No use or disclosure outside AbbVie is permitted without prior written authorization from AbbVie.

Category	IMWG Response Criteria[a]
Sequencing MRD-negative	Absence of clonal plasma cells by NGS on bone marrow aspirate in which presence of a clone is defined as less than 2 identical sequencing reads obtained after DNA sequencing of bone marrow aspirates using the LymphoSIGHT platform (or validated equivalent method) with a minimum sensitivity of 1 in 10^5 nucleated cells[g] or higher
Imaging plus MRD-negative	MRD negativity as defined by NGF or NGS plus disappearance of every area of increased tracer uptake found at baseline or a preceding PET/CT or decrease to less mediastinal blood pool SUV or decrease to less than that of surrounding normal tissue[h]

CRAB features = calcium elevation, renal failure, anemia, lytic bone lesions; FLC = free light chain; IHC = immunohistochemistry; M-protein = myeloma protein; MFC = multiparametric flow cytometry; MRD = minimum residual disease; NGF = next generation flow; NGS = next generation sequencing; SPD = sum of the products of the maximal perpendicular diameters of measured lesions

Adapted from Kumar et al, 2016[5]

a. All response categories require 2 consecutive assessments made at any time before the initiation of any new therapy; All categories of response and MRD also require no known evidence of progressive or new bone lesions if radiographic studies were performed. Radiographic studies are not required to satisfy these response requirements, except for the requirement of FDG PET if imaging MRD-negative status is reported. Bone marrow assessments need not be confirmed.
b. Presence/absence of clonal cells is based upon the κ/λ ratio. An abnormal κ/λ ratio by IHC requires a minimum of 100 plasma cells for analysis. An abnormal ratio reflecting presence of an abnormal clone is κ/λ of > 4:1 or < 1:2.
c. Positive immunofixation alone in a subject previously classified as achieving a complete response will not be considered progression.
d. In the case where a value is felt to be a spurious result per physician discretion (for example, a possible laboratory error), the value will not be considered when determining the lowest value.
e. Sustained MRD negativity when reported should also annotate the method used (for example, sustained flow MRD negative, sustained sequencing MRD negative).
f. Bone marrow MFC should follow NGF guidelines.[18]
g. DNA sequencing assay on bone marrow aspirate should use a validated assay such as LymphoSIGHT (Sequenta).
h. Imaging should be performed once MRD negativity is determined by MFC or NGS following criteria used by Zamagni, et al.[19]

No use or disclosure outside AbbVie is permitted without prior written authorization from AbbVie.

7.4 INTERNATIONAL WORKING GROUP RESPONSE CRITERIA FOR ACUTE MYELOID LEUKEMIA

Response	Definition
Complete Remission (CR)	• < 5% blasts in the BM<br>• no evidence of circulating blasts or extramedullary disease<br>• ANC > 1,000/µL<br>• platelets > 100,000/µL
CR with incomplete blood count recovery CRi[a])	• < 5% blasts in the BM<br>• no evidence of circulating blasts or extramedullary disease<br>• 1 of the following:<br>• ANC > 1,000/µL<br>• platelets > 100,000/µL
CR with partial hematologic recovery (CRh[b])	• < 5% blasts in the BM<br>• no evidence of circulating blasts or extramedullary disease<br>• ANC > 500/µL<br>• platelets > 50,000/µL
Partial Remission (PR)	All of the hematologic values for a CR but with a decrease of at least 50% in the percentage of blasts to 5% to 25% in the bone marrow aspirate
Morphologic Leukemia-Free State (MLFS)	Less than 5% blasts in an aspirate sample with marrow spicules and with a count of at least 200 nucleated cells.
Resistant Disease (RD)	Failure to achieve CR, CRi, PR; in subjects surviving at least 7 days following completion of Cycle 1 treatment, with evidence of persistent leukemia by blood and/or bone marrow examination. Study treatment may continue beyond RD if subject is continuing to derive clinical benefit, in opinion of treating investigator.
Morphologic Relapse	Reappearance of ≥ 5% blasts post CR in peripheral blood or bone marrow
Progressive Disease	Progressive disease per ELN Criteria[c] (ANY of the following):<br>• 50% increase in marrow blasts over baseline (a minimum 15% point increase is required in cases with < 30% blasts at baseline; or persistent marrow blast percentage of > 70% over at least 3 months; without at least a 100% improvement in ANC to an absolute level (> 0.5 × 10^9/L [500/mL], and/or platelet count to > 50 × 10^9/L [50,000/mL] non transfused);<br>• 50% increase in peripheral blasts (WBC × % blasts) to > 25 × 10^9/L (> 25,000/µL);<br>• New extramedullary disease

ANC = absolute neutrophil count; BM = bone marrow; CR = complete remission; CRc = composite CR; CRh = CR with partial hematologic recovery; CRi = CR with incomplete blood count recovery; CRp = CR with incomplete platelet recovery; ELN = European LeukemiaNet; IWG = International Working Group; MLFS = morphologic leukemia-free state; OS = overall survival; PR = partial remission; RD = resistant disease

a. CRp should be categorized as CRi.

No use or disclosure outside AbbVie is permitted without prior written authorization from AbbVie.

b. In addition to the other response categories, subjects should be classified at each disease assessment for presence of CRh (independent of best response). Subjects could, therefore, be classified as achieving CRh as well as other response categories listed above at any given assessment.

c. Progressive disease as defined by ELN criteria.[7]

No use or disclosure outside AbbVie is permitted without prior written authorization from AbbVie.

7.5 INHIBITORS AND SUBSTRATES TO BE USED WITH CAUTION

Class	Drugs
P-glycoprotein inhibitors	amiodarone, carvedilol, clarithromycin, dronedarone, itraconazole, lapatinib, lopinavir and ritonavir, propafenone, quinidine, ranolazine, ritonavir, saquinavir and ritonavir, telaprevir, tipranavir and ritonavir, verapamil
Breast cancer resistance protein inhibitors	curcumin, cyclosporine A, eltrombopag
OATP1B1/B3 inhibitors	atazanavir and ritonavir, clarithromycin, cyclosporine, erythromycin, gemfibrozil, lopinavir and ritonavir, rifampin (single dose), simeprevir
OATP1B1/1B3 substrates	asunaprevir, atorvastatin, bosentan, cerivastatin, danoprevir, docetaxel, fexofenadine, glyburide, nateglinide, paclitaxel, pitavastatin, pravastatin, repaglinide, rosuvastatin, simvastatin acid
Strong CYP3A4 inhibitors	boceprevir, cobicistat, danoprevir and ritonavir, elvitegravir and ritonavir, grapefruit juice, indinavir and ritonavir, itraconazole, ketoconazole, lopinavir and ritonavir, paritaprevir and ritonavir and (ombitasvir and/or dasabuvir), posaconazole, ritonavir, saquinavir and ritonavir, telaprevir, tipranavir and ritonavir, telithromycin, troleandomycin, voriconazole, clarithromycin, idelalisib, nefazodone, nelfinavir

NOTE: This list is based on the FDA-recommended list of clinical substrates and inhibitors and is not intended to be an exhaustive list.

Adapted from: https://www.fda.gov/drugs/drug-interactions-labeling/drug-development-and-drug-interactions-table-substrates-inhibitors-and-inducers

CONFIDENTIAL INFORMATION
No use or disclosure outside AbbVie is permitted without prior written authorization from AbbVie.

7.6 GUIDE OF EXCLUDED MEDICATION THAT AFFECT QT INTERVAL

Generic Name	Brand Names
Alfuzosin	Uroxatral®
Amiodarone	Cordarone®, Pacerone®, Nexterone®
Anagrelide	Agrylin®, Xagrid®
Apomorphine	Apokyn®, Ixense®, Spontane®, Uprima®
Aripiprazole	Abilify®, Aripiprex®
Arsenic trioxide	Trisenox®
Astemizole (Off US mkt)	Hismanal®
Atazanavir	Reyataz®
Azithromycin	Zithromax®, Zmax®
Bedaquiline	Sirturo®
Bepridil (Off US mkt)	Vascor®
Bortezomib	Velcade®, Bortecad®
Bosutinib	Bosulif®
Chloroquine	Aralen®
Chlorpromazine	Thorazine®, Largactil®, Megaphen®
Cisapride (Off US mkt)	Propulsid®
Citalopram	Celexa®, Cipramil®
Clarithromycin	Biaxin®, Prevpac®
Clozapine	Clozaril®, Fazaclo®, Versacloz®
Cocaine	Cocaine
Crizotinib	Xalkori®
Dabrafenib	Tafinlar®
Dasatinib	Sprycel®
Dexmedetomidine	Precedex®, Dexdor®, Dexdomitor®
Dihydroartemisinin + piperaquine	Eurartesim®
Disopyramide	Norpace®
Dofetilide	Tikosyn®
Dolasetron	Anzemet®
Domperidone (Not on US mkt)	Motilium®, Motillium®, Motinorm Costi®, Nomit®
Droperidol	Inapsine®, Droleptan®, Dridol®, Xomolix®
Eribulin	Halaven®

No use or disclosure outside AbbVie is permitted without prior written authorization from AbbVie.

Generic Name	Brand Names
Erythromycin	E.E.S.®, Robimycin®, EMycin®, Erymax®, Ery-Tab®, Eryc Ranbaxy®, Erypar®, Eryped®, Erythrocin Stearate Filmtab®, Erythrocot®, E-Base®, Erythroped®, Ilosone®, MY-E®, Pediamycin®, Zineryt®, Abboticin®, Abboticin-ES®, Erycin®, PCE Dispertab®, Stiemycine®, Acnasol®, Tiloryth®
Escitalopram	Cipralex®, Lexapro®, Nexito®, Anxiset-E® (India), Exodus® (Brazil), Esto® (Israel), Seroplex®, Elicea®, Lexamil®, Lexam®, Entact® (Greece), Losita® (Bangladesh), Reposil® (Chile), Animaxen® (Colombia), Esitalo® (Australia), Lexamil® (South Africa)
Famotidine	Pepcid®, Fluxid®, Quamatel®
Felbamate	Felbatol®
Fingolimod	Gilenya®
Flecainide	Tambocor®, Almarytm®, Apocard®, Ecrinal®, Flécaine®
Foscarnet	Foscavir®
Fosphenytoin	Cerebyx®, Prodilantin®
Gatifloxacin (Off US mkt)	Tequin®
Gemifloxacin	Factive®
Granisetron	Kytril®, Sancuso®, Granisol®
Grepafloxacin (Off market worldwide)	Raxar
Halofantrine	Halfan®
Haloperidol	Haldol® (US & UK), Aloperidin®, Bioperidolo®, Brotopon®, Dozic®, Duraperidol® (Germany), Einalon S®, Eukystol®, Halosten®, Keselan®, Linton®, Peluces®, Serenace®, Serenase®, Sigaperidol®
Ibutilide	Corvert®
Iloperidone	Fanapt®, Fanapta®, Zomaril®
Isradipine	Dynacirc®
Lapatinib	Tykerb®, Tyverb®
Levofloxacin	Levaquin®, Tavanic®
Levomethadyl (Off US mkt)	Orlaam®
Lithium	Eskalith®, Lithobid®
Mesoridazine (Off US mkt)	Serentil®
Methadone	Dolophine®, Symoron®, Amidone®, Methadose®, Physeptone®, Heptadon®
Mifepristone	Korlym®, Mifeprex®
Mirabegron	Myrbetriq®
Mirtazapine	Remeron

No use or disclosure outside AbbVie is permitted without prior written authorization from AbbVie.

Generic Name	Brand Names
Moexipril/HCTZ	Uniretic®, Univasc®
Moxifloxacin	Avelox®, Avalox®, Avelon®
Nicardipine	Cardene®
Nilotinib	Tasigna®
Norfloxacin	Noroxin®, Ambigram®
Ofloxacin	Floxin®
Olanzapine	Zyprexa®, Zydis®, Relprevv®
Ondansetron	Zofran®, Anset®, Ondemet®, Zuplenz®, Emetron®, Ondavell®, Emeset®, Ondisolv®, Setronax®
Oxytocin	Pitocin®, Syntocinon®
Paliperidone	Invega®, Xepilon®
Pasireotide	Signifor®
Pazopanib	Votrient®
Pentamidine	Pentam®
Perflutren lipid microspheres	Definity®
Pimozide	Orap®
Pipamperone (Not on US Mkt)	Dipieron (E.U.), Propitan (Japan)
Probucol (Off US mkt)	Lorelco®
Procainamide (Oral off US mkt)	Pronestyl®, Procan®
Promethazine	Phenergan®
Quetiapine	Seroquel®
Quinidine	Quinaglute®, Duraquin®, Quinact®, Quinidex®, Cin-Quin®, Quinora®
Ranolazine	Ranexa®, Ranozex®
Rilpivirine	Edurant®, Complera®, Eviplera®
Risperidone	Risperdal®
Roxithromycin (Not on US mkt)	Rulide®, Xthrocin®, Roxl-150®, Roxo®, Surlid®, Rulide®, Biaxsig®, Roxar®, Roximycinv®, Roxomycin®, Rulid®, Tirabicin®, Coroxin®
Saquinavir	Invirase® (combo)
Sertindole (Not on US mkt)	Serdolect®, Serlect®
Sevoflurane	Ulane®, Sojourn®
Sorafenib	Nexavar®
Sotalol	Betapace®, Sotalex®, Sotacor®
Sparfloxacin (Off US mkt)	Zagam®

No use or disclosure outside AbbVie is permitted without prior written authorization from AbbVie.

Generic Name	Brand Names
Sulpiride (Not on US mkt)	Dogmatil®, Dolmatil®, Eglonyl®, Espiride®, Modal®, Sulpor®
Sunitinib	Sutent®
Tacrolimus	Prograf®, Advagraf®, Protopic®
Tamoxifen	Nolvadex® (Discontinued June 2013), Istubal®, Valodex®
Telavancin	Vibativ®
Telithromycin	Ketek®
Terfenadine (Off US mkt)	Seldane®
Tetrabenazine (Orphan drug in US)	Nitoman®, Xenazine®
Thioridazine	Mellaril®, Novoridazine®, Thioril®
Tizanidine	Zanaflex®, Sirdalud®
Tolterodine	Detrol®, Detrusitol®
Toremifene	Fareston®
Vandetanib	Caprelsa®
Vardenafil	Levitra®
Vemurafenib	Zelboraf®
Venlafaxine	Effexor®, Efexor®
Vorinostat	Zolinza®
Ziprasidone	Geodon®, Zeldox®

Note: This comprehensive list of medications was obtained from crediblelmeds.org.

Document Approval

Study M19025 - A First-in-Human Study of the MCL-1 Inhibitor, ABBV-467 - Operations Manual for Protocol Version 3-0 - 29Apr2020

Version: 1.0 **Date:** 30-Apr-2020 09:09:26 PM **Company ID:** 04302020-00F9F684636E6D-00001-en

Signed by:	Date:	Meaning Of Signature:
[redacted]	30-Apr-2020 12:52:12 AM	Approver
	30-Apr-2020 02:33:23 AM	Approver
	30-Apr-2020 03:23:17 AM	Subject Matter Expert
	30-Apr-2020 01:07:20 P	Author
	30-Apr-2020 03:03:33 PM	Approver
	30-Apr-2020 05:54:13 PM	Approver
	30-Apr-2020 09:00:08 P	Approver
	30-Apr-2020 09:09:26 PM	Approver

Document Approval

Study M19025 - A First-in-Human Study of the MCL-1 Inhibitor, ABBV-467 - Protocol Version 3-0 - EudraCT 2018-003744-24 - 29Apr2020

Version: 1.0 **Date:** 30-Apr-2020 09:09:31 PM **Company ID:** 04302020-00F9F684636DB2-00001-en

Signed by:	Date:	Meaning Of Signature:
[redacted]	30-Apr-2020 12:52:12 AM	Approver
	30-Apr-2020 02:33:23 AM	Approver
	30-Apr-2020 03:23:17 AM	Subject Matter Expert
	30-Apr-2020 01:07:20 P	Author
	30-Apr-2020 03:03:33 PM	Approver
	30-Apr-2020 05:54:13 PM	Approver
	30-Apr-2020 09:00:08 P	Approver
	30-Apr-2020 09:09:26 PM	Approver
