# Supplementary material for: Selective MCL-1 inhibitor ABBV-467 is efficacious in tumor models but is associated with cardiac troponin increases in patients
Source: Commun Med (Lond). 2023 Oct 25;3:154. doi: 10.1038/s43856-023-00380-z (PMC10600239; doi:10.1038/s43856-023-00380-z)
Supplement: Supplementary file 11 — Description of Additional Supplementary Files [file 43856_2023_380_MOESM11_ESM.pdf]

Description of Additional Supplementary Files

File name: Supplementary Data 1

Description: IC_{50} at or below 1 µM for ABBV-467 against off-target receptors, ion channels, and transporters

File name: Supplementary Data 2

Description: Troponin assay results

File name: Supplementary Data 3

Description: Source data underlying Table 1 and Figures 2, 4, 5, and 6

File name: Supplementary Data 4

Description: Source data underlying Figure 3A

File name: Supplementary Data 5

Description: Source data underlying Figure 3B

File name: Supplementary Data 6

Description: Source data underlying Figure 3C

File name: Supplementary Data 7

Description: Source data underlying Figure 3D

File name: Supplementary Data 8

Description: Source data underlying Figures 3E-H

File name: Supplementary Data 9

Description: Source data underlying Figures 3I-J
