# Supplementary material for: Selective MCL-1 inhibitor ABBV-467 is efficacious in tumor models but is associated with cardiac troponin increases in patients
Source: Commun Med (Lond). 2023 Oct 25;3:154. doi: 10.1038/s43856-023-00380-z (PMC10600239; doi:10.1038/s43856-023-00380-z)
Supplement: Supplementary file 12 — Reporting Summary [file 43856_2023_380_MOESM12_ESM.pdf]

nature portfolio

Corresponding author(s): Andrew J Souers

Last updated by author(s): Jun 24, 2023

Reporting Summary

Nature Portfolio wishes to improve the reproducibility of the work that we publish. This form provides structure for consistency and transparency in reporting. For further information on Nature Portfolio policies, see our Editorial Policies and the Editorial Policy Checklist.

Statistics

For all statistical analyses, confirm that the following items are present in the figure legend, table legend, main text, or Methods section.

n/a	Confirmed	
☐	☒	The exact sample size (*n*) for each experimental group/condition, given as a discrete number and unit of measurement
☐	☒	A statement on whether measurements were taken from distinct samples or whether the same sample was measured repeatedly
☐	☒	The statistical test(s) used AND whether they are one- or two-sided<br>*Only common tests should be described solely by name; describe more complex techniques in the Methods section.*
☐	☒	A description of all covariates tested
☐	☒	A description of any assumptions or corrections, such as tests of normality and adjustment for multiple comparisons
☐	☒	A full description of the statistical parameters including central tendency (e.g. means) or other basic estimates (e.g. regression coefficient) AND variation (e.g. standard deviation) or associated estimates of uncertainty (e.g. confidence intervals)
☐	☒	For null hypothesis testing, the test statistic (e.g. *F*, *t*, *r*) with confidence intervals, effect sizes, degrees of freedom and *P* value noted<br>*Give P values as exact values whenever suitable.*
☐	☒	For Bayesian analysis, information on the choice of priors and Markov chain Monte Carlo settings
☐	☒	For hierarchical and complex designs, identification of the appropriate level for tests and full reporting of outcomes
☐	☒	Estimates of effect sizes (e.g. Cohen's *d*, Pearson's *r*), indicating how they were calculated

Our web collection on statistics for biologists contains articles on many of the points above.

Software and code

Policy information about availability of computer code

Data collection	X-ray diffraction data were collected at the ALBA (Barcelona, Spain) and APS (Argonne, IL, USA) synchrotron sites. X-ray Diffraction data were collected under gaseous nitrogen (100 K) at the APS beamline 17-ID (Advanced Photon Source, Argonne, IL). In vivo data was collected using Study Director. Hydrogen-1 nuclear magnetic resonance spectra were obtained on a Varian UNITY or Inova (500 MHz), Varian UNITY (400 MHz), or Varian UNITY plus or Mercury (300 MHz) instrument. Mass spectral analyses were performed on a Finnigan SSQ 7000 GC/MS mass spectrometer using different techniques, including electrospray ionization, desorption chemical ionization, and atmospheric pressure chemical ionization, as specified for individual compounds. Exact mass measurements were performed on a Finnigan FTMS Newstar T70 mass spectrometer. Analytic liquid chromatography-mass spectrometry was performed on a Finnigan Navigator Mass Spectrometer and Agilent 1100 HPLC system running Xcalibur 1.2 and Open-Access 1.3 software. The mass spectrometer was operated under positive atmospheric pressure chemical ionization conditions. The HPLC system comprised an Agilent Quaternary pump, degasser, column compartment, autosampler, and diode-array detector, with a Sedere Sedex 75 evaporative light-scattering detector. Preparative reverse-phase high-performance liquid chromatography (HPLC) was performed on an automated Gilson HPLC system. For the FRET assays, Fluorescence was measured on the EnVision™ (Perkin Elmer) using a 340/35-nm excitation filter and 520/525- (F-Bak) and 495/510-nm (Tb-labeled anti-glutathione S-transferase [GST] antibody) emission filters.
Data analysis	X-ray diffraction data were processed using Autoproc (Global Phasing) and Molrep (CCP4 suite of programs) then refined using Refmac5 (CCP4 suite of programs), Buster (Global Phasing) and Coot. Figures were prepared using PyMol (Schrodinger). In vivo data was analyzed using GraphPad Prism or Microsoft Excel. TGI and TGDs were calculated using Log-Rank comparison with JMP software (SAS). Clinical pharmacokinetic data was calculated using Phoenix WinNonlin, Certara and figures were prepared using Rstudio. Data from experiments in vivo were analyzed using the student's t-test for TGI values and the Mann-Whitney U test for TGD using Excel and GraphPad Prism. Determination of caspase-3/7 activation, Δψm, and annexin-V positivity was performed using the Intellicyt MultiCyt 4-Plex Kit and high-content flow cytometry (Intellicyt, Albuquerque, NM). For cell viability assays, cell viability was determined using CellTiter-Glo Luminescent

assay (Promega, Madison, WI). For Western blot analysis, proteins were visualized utilizing the Odyssey® infrared imaging system (LI-COR Biosciences). Plasma samples of ABBV-467 were quantified using a validated LC-tandem MS with a lower limit of detection of 3 ng/mL. ABBV-467 PK parameters were calculated using standard non-compartmental approaches with Phoenix, WinNonlin (Version 8.0, Certara).

For manuscripts utilizing custom algorithms or software that are central to the research but not yet described in published literature, software must be made available to editors and reviewers. We strongly encourage code deposition in a community repository (e.g. GitHub). See the Nature Portfolio guidelines for submitting code & software for further information.

Data

Policy information about availability of data

All manuscripts must include a data availability statement. This statement should provide the following information, where applicable:

- Accession codes, unique identifiers, or web links for publicly available datasets
- A description of any restrictions on data availability
- For clinical datasets or third party data, please ensure that the statement adheres to our policy

All data generated or analysed during this study are included in this published article (and its supplementary information files). The atomic coordinates for the structures of Mcl-1 in complex with compound 1, compound 2 and ABBV-467 have been deposited in the Protein Data Bank under the accession codes 8EKX, 8EL0 and 8EL1 respectively. Source data for Table 1 and Figures 2–6 can be found in Supplementary Data 3–9. All other data are available from the corresponding author on reasonable request. The study protocol can be found in the Supplementary Information. All other data are available from the corresponding author (or other sources, as applicable) on reasonable request.

Human research participants

Policy information about studies involving human research participants and Sex and Gender in Research.

Reporting on sex and gender	Sex has been considered as a biological variable for this study. The participants self-reported sex per informed consent. Our statistical analysis of this study included the disaggregation by sex; however, no conclusions can be made based on small sample size (n=8). 75% of the participants were male, 25% were female. Gender was not a consideration as there is no information that suggests gender plays a variable in this type of study.
Population characteristics	Population characteristics collected in this study includes: Age, sex, race, weight, ECOG status, and medical history (including disease type, stage, prior therapies, prior procedures, concomitant medications)
Recruitment	Recruitment of participants was performed by the principle investigators.
Ethics oversight	FDA, Israel National Ethics Committee, and PMDA (Japan)

Note that full information on the approval of the study protocol must also be provided in the manuscript.

Field-specific reporting

Please select the one below that is the best fit for your research. If you are not sure, read the appropriate sections before making your selection.

☒ Life sciences ☐ Behavioural & social sciences ☐ Ecological, evolutionary & environmental sciences

For a reference copy of the document with all sections, see nature.com/documents/nr-reporting-summary-flat.pdf

Life sciences study design

All studies must disclose on these points even when the disclosure is negative.

Sample size	The sample size was determined by the number of participants per dose cohort, as well as the number of dose escalations required to define the RP2D. As well as the expansion of dose levels based on the Boin design and safety at what is ultimately declared the RP2D. For in vivo studies, power analysis based on historical data (variation in treatment and tumor growth) was used to calculate sample size for any specific studies.
Data exclusions	There was no data excluded in this study.
Replication	All in vitro binding and cellular assays were performed at least in triplicate unless otherwise specified. For the in vivo studies, any replications conducted did not refute the original findings.
Randomization	Animals were randomized using match distribution (in methods)
Blinding	Animals were randomized into group allocation. Blinded for tumor measurement, but drug information has to be listed on the cages based on IACUC protocol.

Reporting for specific materials, systems and methods

We require information from authors about some types of materials, experimental systems and methods used in many studies. Here, indicate whether each material, system or method listed is relevant to your study. If you are not sure if a list item applies to your research, read the appropriate section before selecting a response.

Materials & experimental systems

n/a	Involved in the study
☐	☒ Antibodies
☐	☒ Eukaryotic cell lines
☒	☐ Palaeontology and archaeology
☐	☒ Animals and other organisms
☐	☒ Clinical data
☒	☐ Dual use research of concern

Methods

n/a	Involved in the study
☒	☐ ChIP-seq
☒	☐ Flow cytometry
☒	☐ MRI-based neuroimaging

Antibodies

Antibodies used	Anti-BAX (Cell Signaling, catalogue #CS2933), anti-BAK (Ab, catalogue #ab32371), anti-GAPDH (Abcam, catalogue #ab110305), IRDye 680/800CW-conjugated antibodies (LI-COR Biosciences, Lincoln, NE, USA).
Validation	Anti-BAX and anti-BAK antibodies were validated using cell lines deficient in the genes encoding BAX and BAK proteins, respectively. The anti-GAPDH antibody was used as a loading control and their specificity for GAPDH not determined.

Eukaryotic cell lines

Policy information about cell lines and Sex and Gender in Research

Cell line source(s)	For the in vivo studies: AMO-1 (DSMZ), OPM-2 (DSMZ), NCI-H929 (ATCC), OCI-AML2 (DSMZ), and MV4-11 (DSMZ). HERG-1 Transfected HEK293 Cell Line B<br>WARF: P04289US<br>Inventors: Craig January, Zhengfeng Zhou, Qiuming Gong, Gail Robertson, Blake Anson, Matthew Trudeau, Corey Anderson<br>HEK cell line sex: female (no Y chromosome); aneuploid<br>Cloned full-length cDNA of the HERG-1 cardiac potassium channel gene.
Authentication	STR profiling by IDEXX and SoC sensitivity. HEK293 cell lines not authenticated.
Mycoplasma contamination	all cell lines tested negative
Commonly misidentified lines<br>(See ICLAC register)	NA

Animals and other research organisms

Policy information about studies involving animals; ARRIVE guidelines recommended for reporting animal research, and Sex and Gender in Research

Laboratory animals	Female C.B-17 SCID-Beige purchased from Charles River and NSG mice purchased from The Jackson Laboratory. Sexually mature male beagle dogs were used for cardiovascular safety studies. Sexually mature male and female beagle dogs were used for toxicology studies. CD IGS Sprague-Dawley rats, Crl:CD(SD), (~8 weeks old at initiation of dosing) were used for toxicology studies.
Wild animals	NA
Reporting on sex	In vivo efficacy Findings were only tested in female animals. Male animals were used for cardiovascular safety testing, as there was no indication of an effect of sex on pharmacokinetics. Male and female animals were used for toxicology studies and sex effects were not observed on toxicological outcomes.
Field-collected samples	NA
Ethics oversight	All animal studies were conducted in accordance with the guidelines approved by the Institutional Animal Care and Use Committees of AbbVie. All animal studies were conducted in accordance with the guidelines approved by the Institutional Animal Care and Use Committees of AbbVie. All animal studies were conducted in accordance with the guidelines approved by the Institutional Animal Care and Use Committees of AbbVie and Charles River Laboratories, Mattawan, MI.

Note that full information on the approval of the study protocol must also be provided in the manuscript.

Clinical data

Policy information about clinical studies

All manuscripts should comply with the ICMJE guidelines for publication of clinical research and a completed CONSORT checklist must be included with all submissions.

Clinical trial registration	NCT04178902
Study protocol	Redacted protocol is available with the journal entry
Data collection	Data was collected at the investigators sites in Japan and Israel from May, 2020 and June, 2021
Outcomes	Primary and secondary outcomes are predefined for first-in-human studies for oncology drugs.
